# Diversity-oriented synthesis of glycomimetics

**DOI:** 10.1038/s42004-021-00520-3

**Published:** 2021-06-24

**Authors:** Michael Meanwell, Gaelen Fehr, Weiwu Ren, Bharanishashank Adluri, Victoria Rose, Johannes Lehmann, Steven M. Silverman, Rozhin Rowshanpour, Christopher Adamson, Milan Bergeron-Brlek, Hayden Foy, Venugopal Rao Challa, Louis-Charles Campeau, Travis Dudding, Robert Britton

**Affiliations:** 1 Department of Chemistry, Simon Fraser University, Burnaby, BC Canada; 2 Department of Process Research and Development, Merck & Co., Inc, Rahway, NJ USA; 3 Department of Chemistry, Brock University, St. Catharines, ON Canada

**Keywords:** Synthetic chemistry methodology, Diversity-oriented synthesis

## Abstract

Glycomimetics are structural mimics of naturally occurring carbohydrates and represent important therapeutic leads in several disease treatments. However, the structural and stereochemical complexity inherent to glycomimetics often challenges medicinal chemistry efforts and is incompatible with diversity-oriented synthesis approaches. Here, we describe a one-pot proline-catalyzed aldehyde α-functionalization/aldol reaction that produces an array of stereochemically well-defined glycomimetic building blocks containing fluoro, chloro, bromo, trifluoromethylthio and azodicarboxylate functional groups. Using density functional theory calculations, we demonstrate both steric and electrostatic interactions play key diastereodiscriminating roles in the dynamic kinetic resolution. The utility of this simple process for generating large and diverse libraries of glycomimetics is demonstrated in the rapid production of iminosugars, nucleoside analogues, carbasugars and carbohydrates from common intermediates.

## Abstract (web summary)

Glycomimetics are structural mimics of carbohydrates that can replicate their biological activity but have improved drug-like properties. Here, using proline-catalysed α-halogenation/aldol cascades, carbohydrate building blocks are readily assembled and then diversified into glycomimetics including imino- and carbasugars.

## Introduction

**Fig. 1:**
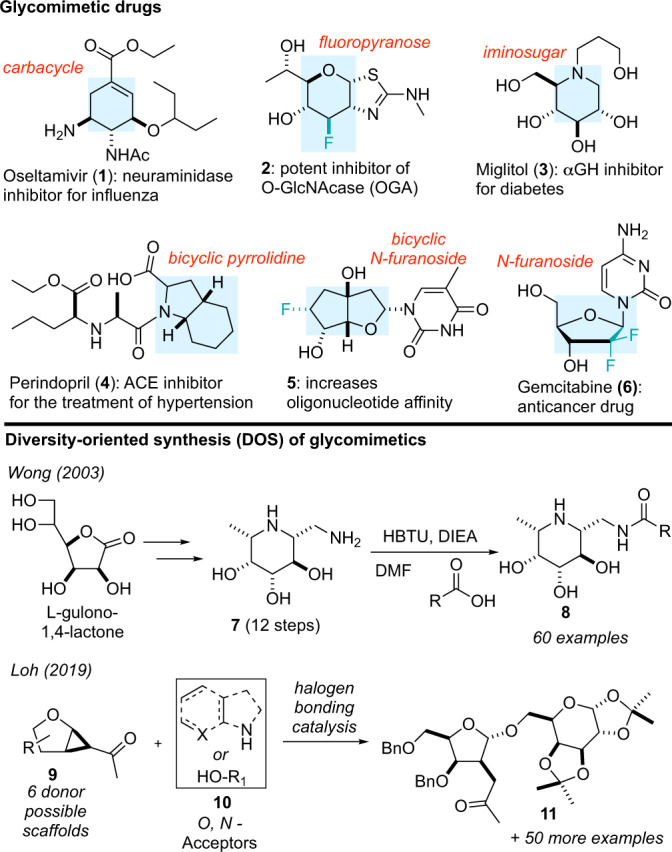
Glycomimetics and DOS approaches used to prepare glycomimetic libraries. Examples of glycomimetic drugs and DOS approaches to prepare DOS libraries of glycomimetics for screening purposes. Shaded regions highlight the glycomimetic structures.

Carbohydrates are essential biomolecules that play critical roles in cell signaling, protein folding, and metabolism^1^. The recognition and regulation of carbohydrate structure are thus strictly controlled processes that rely on the fidelity of an array of carbohydrate-specific enzymes (e.g., glycosyl transferases and glycoside hydrolases) and binding proteins (e.g., lectins)^2,3^. While the use of carbohydrates as therapeutics is limited by generally poor pharmacokinetic properties, structural mimics of carbohydrates, also known as glycomimetics (**1–6**, Fig. 1), that interact in a similar manner with protein targets but have improved drug-like properties (e.g., affinity, stability and bioavailability) represent promising alternatives^2–8^. Common structural changes found in glycomimetics include replacement of the endocyclic oxygen with a carbon (carbasugars: **1**)^9^ or nitrogen (iminosugar: **3** and **4**)^10^ and substitution of a fluorine atom for hydroxyl groups (deoxyfluorosugars: **2**, **5**, and **6**)^11^. For example, Oseltamivir (**1**)^12^ is a carbasugar mimic of sialic acid and a potent neuraminidase inhibitor that became the frontline antiviral drug used during the H1N1 pandemic in 2009. Likewise, Miglitol (**3**)^13^ is a glycomimetic that functions by inhibiting α-glucosidases and decreasing carbohydrate metabolism in patients with type II diabetes. Notably, iminosugars like **3** are typically protonated at physiological pH, which allows them to mimic the oxacarbenium ion transition state traversed during hydrolysis by glycoside hydrolases^10^.

The rational design of glycomimetic drugs often initiates with a structural analysis of the natural carbohydrate substrate bound to the protein target of interest, followed by iterative synthesis campaigns focused on stabilizing the bioactive conformation and removing unnecessary functional groups to improve drug-like properties^5^. Considering the vast array of biologically relevant carbohydrates, diversity-oriented synthesis (DOS)^14,15^ strategies that broadly sample this biologically relevant chemical space would also be well-suited to support drug discovery efforts^16^. However, the functional group density and stereochemical complexity inherent to carbohydrates often requires that DOS strategies rely on the derivatization of naturally occurring carbohydrates, ultimately limiting diversity^16^. For example, Wong has reported a library of potent and selective α-fucosidase inhibitors derived from 1-aminomethyl-fuconojirimycin (**7**) (Fig. 1)^17^. Here, late-stage amide coupling was used to modify the core scaffold **7**, itself generated through a 12-step synthesis. A DOS approach has also been reported by Marcaurelle, where 2,3-unsaturated C-glycoside scaffolds were used to synthesize several new bicyclic carbohydrates^18^. Recently, Loh reported a strategy for the diversification of carbohydrates using hydrogen- and halogen-bond-catalyzed strain release glycosylation to produce complex *O,N*-glycoside analogs **11** (Fig. 1)^19^. Ultimately, this platform enabled the discovery of potential leads for treating acquired cancer resistance. While these limited examples highlight a clear role for DOS in the generation of glycomimetics, they also underscore the need for new synthetic strategies that enable rapid sampling of more diverse regions of carbohydrate-associated chemical space^20^.

**Fig. 2:**
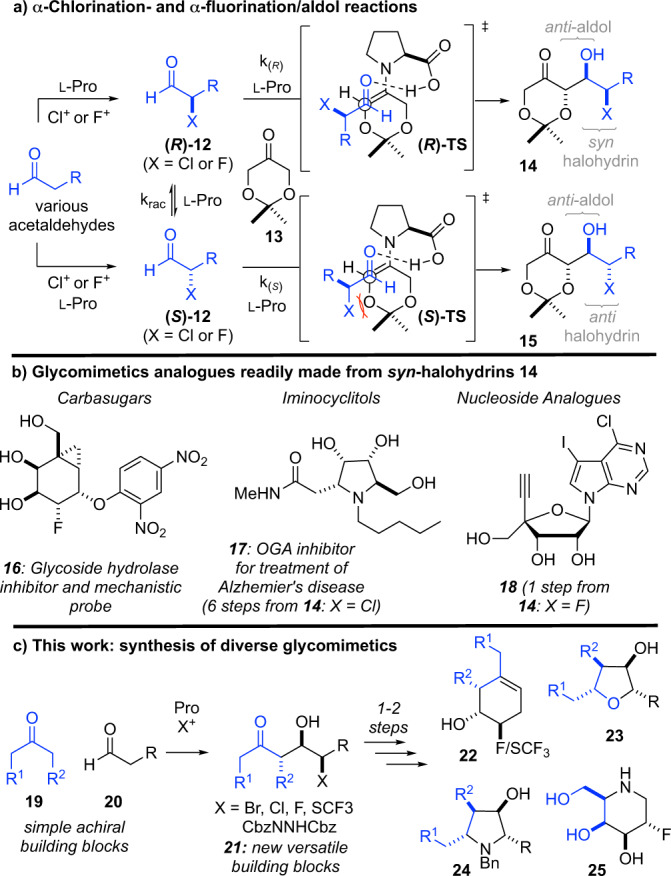
α-Halogenation/aldol reactions used to prepare glycomimetics. **a** Proline-catalyzed α-chlorination- and α-fluorination/aldol reactions used for preparing glycomimetics. The fragments derived from aldehyde coupling partner are colored in blue. **b** Examples of glycomimetics that have been synthesized from α-chlorination- and α-fluorination/aldol reactions. **c** The discovery of new α-functionalization/aldol reactions to diversity-oriented synthesis of glycomimetics. The fragments derived from ketone coupling partner are colored in blue.

As an alternative de novo approach to carbohydrates and glycomimetics, we have reported a one-pot α-chlorination/aldol reaction (αCAR, Fig. 2)^21^. As detailed in Fig. 2 (top panel), here proline catalyzes (i) the racemic α-chlorination of aldehydes, (ii) the interconversion of the resulting racemic α-chloroaldehydes **12**, and (iii) their subsequent aldol reaction with dioxanone **13**. To rationalize the preference for syn-chlorohydrins **14**, we proposed that electrostatic repulsion between the Cl and O atoms in the transition structure (*S*)-TS (X = Cl) disfavors formation of anti-chlorohydrin **15**^21^. The high degree of enantioselectivity results from H-bonding that directs the facial approach of the correctly configured α-chloroaldehyde to the proline-derived enamine through a Houk–List type transition structure^22–24^. Importantly, chlorohydrin scaffolds **14** produced via this dynamic kinetic resolution (DKR) can be readily converted into ribose analogs^21^ as well as other glycomimetics including carbasugars (e.g., **16**^25^) and iminosugars^26^ (e.g. **17**^27^). Recently, we reported a complimentary proline-catalyzed α-fluorination/aldol reaction (αFAR) that supports the rapid synthesis of nucleoside analogs (e.g., **18**)^28^. While these two processes have provided access to a range of useful glycomimetics, both the αCARs and αFARs rely exclusively on dioxanone **13** as the ketone coupling partner^24,29,30^, which prevents wider application of these strategies to library synthesis and DOS pursuits.

## Results and discussion

### α-Functionalization/aldol reactions

Based on the limitations noted above, we envisioned that new α-functionalization/aldol reactions^31,32^, involving a broader selection of (i) electrophiles and (ii) enolizable ketones, would support the construction of diverse collections of glycomimetics. Specifically, we aimed to exploit organocatalytic aldehyde α-functionalization reactions, including α-chlorination^33–35^, α-fluorination^36–38^, α-amination^39,40^, and α-trifluoromethylthiolation^41^ in combination with proline-catalyzed aldol reactions of various cyclic and acyclic ketones^21^. Importantly, these processes would avoid isolation^27,28^ of often unstable and configurationally labile α-functionalized aldehydes and expand their general utility. Toward this goal, we first investigated combinations of different proline-catalyzed α-functionalization reactions with proline-catalyzed aldol reaction of dioxanone **13**. As summarized in Table 1, these reactions were performed as two-step-one-pot sequences (see Supplementary Table 1 for details). Specifically, l-proline-catalyzed α-functionalization using *N*-chlorosuccinimide^34,42^, *N*-bromosuccinimide^43^, *N*-fluorobenzenesulfonimide^36–38^, *N*-trifluoromethylthiophthalimide (PhthN-SCF_3_)^44^, or dibenzyl azodicarboxylate^39,40^ was followed by the direct addition of dioxanone **13**. As summarized in Table 1, we found that these reactions preferentially afford syn-chlorohydrin **27**, syn-fluorohydrin **28**, syn-bromohydrin **29**, syn-trifluoromethylthiohydrin **30**, and syn-aminohydrin **31** with variable diastereoselectivity and in generally excellent enantioselectivity. Considering that the proline-catalyzed α-fluorination of aldehydes is not an enantioselective process (ee’s < 30%)^45^, the selective formation of fluorohydrin **28** was attributed to a DKR of the intermediate α-fluoroaldehyde as observed previously with α-chloroaldehydes^21^. In a separate experiment, involving the αFAR of hydrocinnamaldehyde, the diastereomeric ratio of products (>15:1) and enantiomeric excess of the intermediate α-fluoroaldehyde did not change over the course of the reaction even as the (*R*)-fluoroaldehyde was consumed, further confirming the role of a DKR in these processes (see Supplementary Table 2). Notably, the diastereoselectivity in the production of halohydrins (entries 2–4) correlates with increasing electronegativity of the halogen atom. Thus, despite a smaller van der Waals radius and shorter C–X bond length for X = F, the diastereoselective aldol reaction of the α-fluoroaldehyde was more greatly differentiated. The αCAR was also carried out in CH_2_Cl_2_ (rather than 9:1 CH_2_Cl_2_-DMF) and we observed a coincident increase in diastereoselectivity (2.2:1 to 6:1; entries 1 and 2) consistent with an increased influence of electrostatic interactions in the diastereodifferentiating step (Fig. 2). As summarized in entry 5, the α-trifluoromethylthioaldehyde derived from pentanal also underwent a diastero- and enantioselective aldol reaction with dioxanone **13**. In the case of the α-aza aldehyde generated from the reaction of pentanal and dibenzyl azodicarboxylate (entry 6)^40^, steric hindrance precludes formation of a proline enamine required for racemization^46^. Thus, the ultimate diastereoselectivity (dr = 3:1) reflects the ratio of enantiomeric α-aza aldehydes generated in situ. Notably, when this reaction was repeated in CH_2_Cl_2_ or DMSO, the yield was significantly lower. The l-proline-catalyzed aldol reaction between (*S*)-2-Cbz-aminopentanal and dioxanone yielded the corresponding anti-aminohydrin as the sole product, and both l- and d-proline-catalyzed aldol reactions of (*S*)-*N*-Cbz prolinal (Supplementary Schemes 1 and 2) each gave single products without epimerization of the α-stereocenter, further confirming that α-aminoaldehydes do not racemize under these reaction conditions (*k*_rac_ << *k*_aldol_)^47^. We also examined the l-proline-catalyzed aldol reaction of (±)-2-phenylpropanal and dioxanone **13** (Supplementary Scheme 3), which afforded an equal mixture of syn- and anti- diastereomers again suggesting *k*_rac_ << *k*_aldol_.

**Table 1:** α-Functionalization/aldol reactions with pentanal and dioxanone **13**.


Entry	X^+^	Solvent	dr^a^	%ee	Yield^b^
1^c^	NCS	CH_2_Cl_2_	6:1	94	72
2^d, e^	NCS	CH_2_Cl_2_:DMF	2.2:1	ND	ND
3^d, e^	NFSI	CH_2_Cl_2_:DMF	15:1	96	61
4^d, e, f^	NBS	CH_2_Cl_2_:DMF	1.7:1	ND	17
5^g^	PhthN-SCF_3_	CH_2_Cl_2_:DMSO	6:1	91	52
6^h^	CbzNNCbz	MeNO_2_	3:1	98	45

^a^Diastereomeric ratio determined by ^1^H NMR spectroscopic analysis of crude reaction mixture.

^b^% isolated yield of diastereomer shown.

^c^Pentanal (1.0 equiv), L-pro (0.8 equiv), NCS (1.05 equiv), **13** (1.05 equiv), CH_2_Cl_2_, rt, 24 h.

^d^Pentanal (1.5 equiv.), L-pro (1.5 equiv), X^+^ source (1.5 equiv), NaHCO_3_ (1.5 equiv.), DMF (0.75 M), 1.5 h, −10 °C then add **13** (1.0 equiv) in CH_2_Cl_2_, rt, 48 h.

^e^CH_2_Cl_2_-DMF = 9:1.

^f^rt, 60 h.

^g^Pentanal (2.0 equiv.), L-pro (2.0 equiv), PhthN-SCF_3_ (2.0 equiv), NaHCO_3_ (2.0 equiv.), DMSO (0.75 M), 1.25 h, rt then add **13** (1.0 equiv.) in CH_2_Cl_2_, rt, 48 h (CH_2_Cl_2_-DMSO = 5:1).

^h^Pentanal (1.1 equiv.), L-Pro (0.8 equiv), CbzNNCbz (1.0 equiv), MeNO_2_ (0.50 M), rt then add **13** (2.0 equiv), rt, 48 h.

### Diversification of ketone coupling partners

**Fig. 3:**
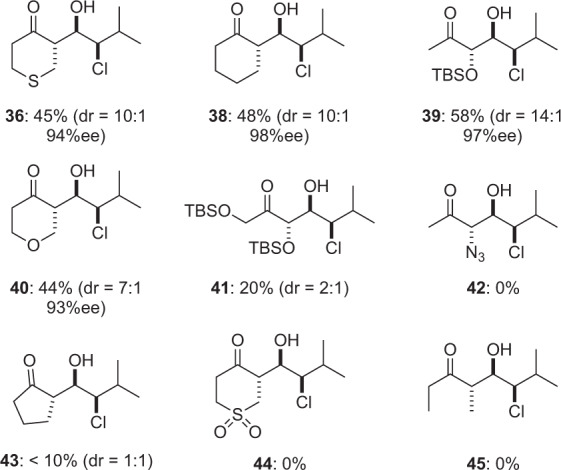
α-Chlorination/aldol reaction products. α-Chlorination/aldol reaction products from a range of ketones. Isolated yields for isolated diastereomer shown, diastereomeric ratio determined by ^1^H NMR spectroscopic analysis of crude reaction mixture.

We next looked to expand the scope of compatible ketones^48^. As summarized in Table 2, we began by examining the l-proline-catalyzed αCAR of tetrahydro-4H-thiopyranone (**35**)^49^ with isovaleraldehyde. Carrying out this reaction using our one-step-one-pot procedure in a variety of solvents afforded the desired syn-chlorohydrin **36**, albeit in low yield (See Table 2, entries 1–3). Suspecting that competitive oxidation of tetrahydro-4H-thiopyran-4-one (**35**) was complicating this process, we adopted a two-step-one-pot sequence. Satisfyingly, when the l-proline-catalyzed α-chlorination of isovaleraldehyde was first carried out in CH_2_Cl_2_ at 0 °C followed by direct addition of thiopyranone **35** in DMSO, the desired aldol adduct **36** was produced in good diastereoselectivity (entry 4). Addition of catalytic amounts of water with the thiopyranone led to modest improvements in yield, and the chlorohydrin **36** was produced in excellent enantiomeric excess (94% ee). Using this straightforward process, several additional ketones (O-TBS-hydroxyacetone, cyclohexanone, and tetrahydro-4H-pyran-4-one)^48^ were engaged in αCARs giving syn-chlorohydrins **36** and **38–40** in good yield and excellent diastereo- and enantioselectivity (Fig. 3). Unfortunately, other simple aliphatic ketones such as acetone, cyclopentanone, and 3-pentanone were incompatible with this process (e.g., **41–45**).

**Table 2:** Optimization of α-chlorination/aldol with **35**.


Entry	Additive	Temp (°C)	Solv. A	Solv. B	dr^a^	Yield^b^
1	None	RT	CH_2_Cl_2_	None	N.D.	<5%
2	None	RT	DMSO	None	N.D.	0%
3	None	0	CH_2_Cl_2_	None	N.D.	<5%
4^c^	None	0	CH_2_Cl_2_	DMSO	10:1	38%
5^c^	H_2_O^d^	0	CH_2_Cl_2_	DMSO	10:1	45%^e^

^a^Diastereomeric ratio determined by ^1^H NMR spectroscopic analysis of crude reaction mixture (stereochemistry of minor diastereomer not assigned).

^b^% isolated yield of **36**.

^c^CH_2_Cl_2_:DMSO = 9:1.

^d^H_2_O (1.0 vol%) was added.

^e^Enantiomeric excess = 94%.

### Computational analysis of proline-catalyzed aldol reactions of α-haloaldehydes

**Fig. 4:**
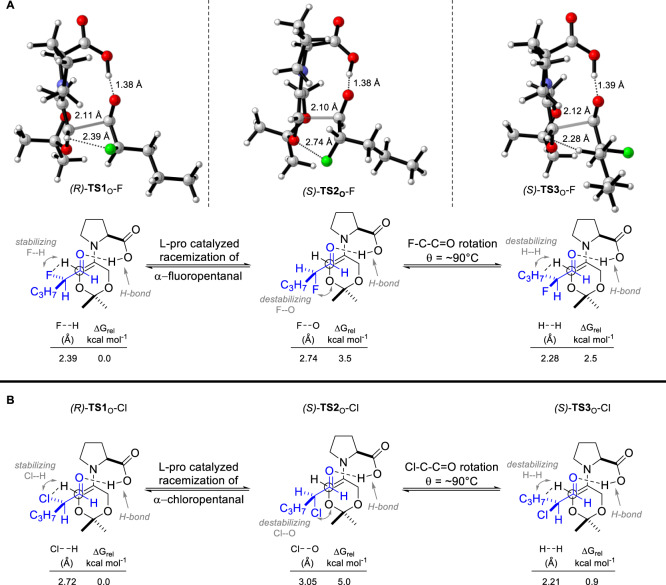
Computational analysis of proline-catalzyed aldol reactions of α-haloaldehydes. **a** Transition state structures (*R*)-**TS1**_O_-F, (*S*)-**TS2**_O_-F and (*S*)-**TS3**_O_-F for l-proline-catalyzed aldol reactions of dioxanone **13** with (*R*)- or (*S*)-2-fluoropentanal. Oxygen atoms are colored in red, fluorine atoms are colored in green. **b** Transition state structures (*R*)-**TS1**_O_-Cl, (*S*)-**TS2**_O_-Cl, and (*S*)-**TS3**_O_-Cl for l-proline-catalyzed aldol reactions involving dioxanone **13** with (*R*)- or (*S*)-2-chloropentanal. DFT calculations were performed using IEFPCM_(DCM)_M06-2X/6-311++G(2d,2p)//IEFPCM_(DCM)_M06-2X/6-31+G(d,p) level of theory. The α-chloro and α-fluoroaldehydes are colored in blue.

To better understand the broad preference for the formation of syn-fluoro- and chlorohydrins (e.g., Tables 1 and 2) regardless of the ketone coupling partner, l-proline-catalyzed aldol reactions involving (*R*)- or (*S*)-2-fluoro- and 2-chloropentanal were examined using density functional theory (DFT) computations employing the program Gaussian 16 with the hybrid meta-GGA M06–2X functional of Truhlar (see Supplementary Data for DFT Calculations pp S146–S695)^50,51^. To start, we analyzed the l-proline-catalyzed aldol reaction between dioxanone **13** and (*R*)- or (*S*)-2-fluoropentanal resulting in the finding of low-energy transition states (*R*)-**TS1**_O_-F, (*S*)-**TS2**_O_-F, and (*S*)-**TS3**_O_-F (Fig. 4a). Notably, (*S*)-**TS2**_O_-F resembled an Evans–Cornforth^52,53^ type carbonyl addition mode (i.e., antiparallel alignment of carbonyl and α-F substituent) while (*S*)-**TS3**_O_-F resembled a Felkin–Anh^54,55^ addition mode (i.e., perpendicular orientation of carbonyl and α-F substituent). Further, each of these transition states possessed a characteristic Houk–List stabilizing O-H···O hydrogen-bonding interaction^22^. Among these structures, transition state (*R*)-**TS1**_O_-F leading to syn-fluorohydrin **28** was energetically favored over the diastereomeric transition states (*S*)-**TS2**_O_-F (ΔG_rel_ = 3.5 kcal mol^−1^) and (*S*)-**TS3**_O_-F (ΔG_rel_ = 2.5 kcal mol^−1^) affording anti-fluorohydrins, which is in agreement with experiment (Table 1, entry 3). The higher energy of the Evans–Cornforth-type transition state (*S*)-**TS2**_O_-F is attributed to a destabilizing interaction between an oxygen on the dioxanone-derived enamine and the aldehyde fluoride substituent with a distance measuring 2.74 Å. By comparison, the corresponding Felkin–Anh-type transition state (*S*)-**TS3**_O_-F is destabilized by unfavorable steric interactions as seen by a close hydrogen–hydrogen contact measuring 2.28 Å. Conversely, the lowest energy transition state (*R*)-**TS1**_O_-F benefited from stabilizing non-covalent interactions (NCIs) and fewer steric contacts as gauged by a NCI surface (see Supplementary Figure 7). Among these NCIs, interestingly, was a fluorine-hydrogen contact measuring 2.39 Å and, in part, deriving from fluorine atom lone pair (*n*) donation into a H–C(*sp*^2^) antibonding orbital (*) of the enamine (*η*_*F*_ → σ*_(C–H)_) contributing ~1.1 kcal mol^−1^ stability based upon natural bond orbital second-order perturbation theory.

The computed trends for reactions of (*R*)- and (*S*)-2-chloropentanal were similar to those discussed above. Namely, three low-energy transition states (*R*)-**TS1**_O_-Cl, (*S*)-**TS2**_O_-Cl, and (*S*)-**TS3**_O_-Cl were found with the latter two possessing structural attributes of prototypical Evans–Cornforth and Felkin–Anh carbonyl addition models (Fig. 4b). Among these structures, the transition state (*R*)-**TS1**_O_-Cl was lower in energy than both of the diastereomeric transition states (*S*)-**TS2**_O_-Cl and (*S*)-**TS3**_O_-Cl, in agreement with the preferential formation of syn-chlorohydrin **27** (Table 1, entry 1). In particular, the Evans–Cornforth-type transition state (*S*)-**TS2**_O_-Cl was destabilized by a repulsive interaction between an oxygen on the dioxanone-derived enamine and the aldehyde chlorine with a distance measuring 3.05 Å. In contrast, Felkin–Anh-type transition state (*S*)-**TS3**_O_-Cl suffered from steric interactions as seen from a close hydrogen–hydrogen contact with a distance of 2.21 Å. On the contrary, the energetically favored transition state (*R*)-**TS1**_O_-Cl contained fewer steric contacts and favorable NCIs, including a chlorine–hydrogen contact measuring 2.72 Å, visible from a NCI surface (see Supplementary Figure 3). Collectively, these studies revealed several conserved features irrespective of fluoro- or chloro-substitution and similar trends were found in the computed reactivity patterns of the cyclic ketones cyclohexanone, tetrahydropyranone, and thiopyranone **35** (Supplementary Tables 1–9). Notably, across this panel of ketones, the preferred transition state structures consistently shared geometries analogous to (*R*)-**TS1**_O_-F and (*R*)-**TS1**_O_-Cl. Moreover, these trends were adhered to with bromo-substitution (see Supplementary Table 6). In all cases, the minimization of repulsive steric interactions and favorable NCIs including a halogen–hydrogen (C–X···H) interaction were identified as key contributors to preferred formation of syn-halohydrin products.

### Scope of α-functionalization/aldol reactions

**Fig. 5:**
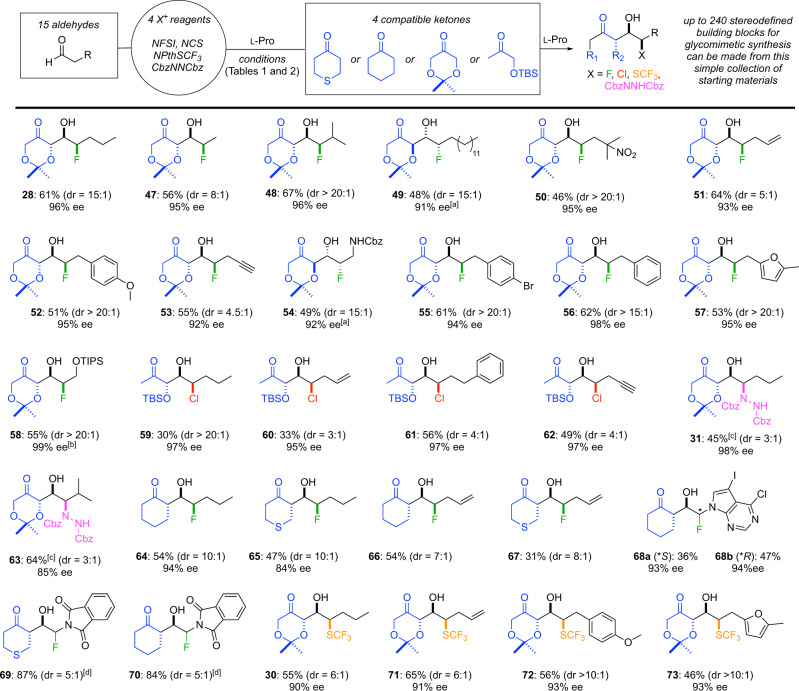
Scope of α-functionalization/aldol reaction. Enantioselective synthesis of chlorohydrins (**59**–**62**), fluorohydrins (**28**, **47**–**58**, **64**–**70**), trifluoromethylthiohydrins (**30**, **71**–**73**), and aminohydrins (**31**, **63**). Yields are for the isolated diastereomer depicted. Diastereomeric ratios (dr) were determined by ^1^H NMR spectroscopic analysis of crude reaction mixtures. Ketones and the fragments of each product that are derived from the ketone are colored in blue. The atoms/groups used to functionalize the aldehyde are colored as follows: fluorine (green), chlorine (red), trifluoromethylthio (orange), and amino group (purple). [a] d-proline was used. [b] Selectfluor was used. [c] Yield over two steps following hydrogenation. [d] Stereochemistry at fluoromethine center not assigned.

With several new α-functionalization/aldol reactions in hand, we examined the reaction of a larger collection of aldehydes with one of the electrophiles demonstrated in Table 1 and ketones demonstrated in Table 2. As detailed in Fig. 5, this strategy allowed for the rapid construction of a collection of fluorohydrins (**28**, **47–58**, and **64–70**), chlorohydrins (**59–62**), trifluoromethylthiohydrins (**30**, **71–73**), and aminohydrins (**31** and **63**) in excellent enantio- and diastereoselectivity. The functional group compatibility of the αFAR is highlighted in the reactions of TIPS-protected 3-hydroxypropanal and Cbz-protected 3-aminopropanal, which delivered the unusual fluorohydrins **54** and **58** bearing a heteroatom at each position in the carbon chain. Overall, the αFAR was tolerant to alkyl, alkene, alkyne, and heteroaryl substituents. Furthermore, simply using d-proline catalysis enables access to enantiomeric syn-fluorohydrins **49** and **54**. Replacing dioxanone **13** with thiopyranone **35** or cyclohexanone provided anti-aldol-syn-fluorohydrin products (**64–70**) in good to excellent yield. We were particularly encouraged by productive reaction of α-fluoro-α-heteroaryl-acetaldehydes, which produced adducts **68–70** that should support the synthesis of nucleoside analogs^28^. Considering the SCF_3_ group is commonly used to increase lipophilicity of drug leads, we were pleased to find that syn-trifluoromethylthiohydrins containing an alkyl (**30**), alkenyl (**71**), aryl (**72**), or heteroaryl (**73**) group could all be prepared in excellent diastereo- and enantioselectivity. To the best of our knowledge, these are the first examples of enantioselective reactions involving α-trifluoromethylthioaldehydes. Finally, we explored the compatibility of a small collection of aldehydes in αCARs with O-TBS-hydroxyacetone, which afforded chlorohydrins **59–62**, and demonstrated that α-amination/aldol reactions deliver enantiomerically enriched syn-aminohydrins **31** and **63**.

### Rapid synthesis of glycomimetics

**Fig. 6:**
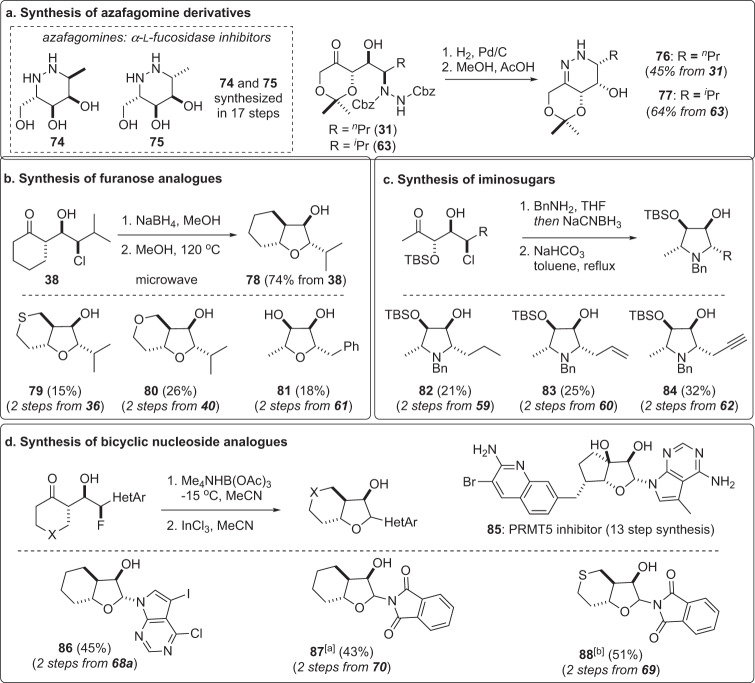
Synthesis of glycomimetics. Rapid diversification of α-functionalization/aldol adducts into glycomimetics including azasugars (**a**), furanose analogs (**b**), iminosugars (**c**), and bicyclic nucleoside analogs (**d**). [a] α:β = 2.5:1; [b] α:β = 3:1.

To demonstrate the potential for this strategy to rapidly generate glycomimetics, several readily available chlorohydrins, fluorohydrins, trifluoromethylthiohydrins, and aminohydrins (Fig. 5) were converted into a diverse collection of iminosugars, furanose analogs, and bicyclic nucleoside analogs (Fig. 6). For example, hydrogenation of syn-aminohydrins allowed access to cyclic hydrazones **76** and **77** in excellent yield over two total steps (panel a). This route compares favorably with reported syntheses of the related azafagomines **74** and **75**, inhibitors of α-fucosidase, that require 17 steps^56^. As depicted in panel b, a series of unusual furanose analogs **78–81** was prepared through 1,3-syn reduction of aldol adducts **36**, **38**, **40**, and **61** with sodium borohydride followed by thermal cyclization^21^. Alternatively, reductive amination of aldol adducts **59**, **60**, and **62** with benzyl amine followed by reflux in toluene under basic conditions gave access to selectively protected 5′-deoxy-iminosugars **82–84** (panel c)^26^. The recent discovery of **85** as a potent and selective PRMT5 inhibitor for cancer treatment highlights bicyclic nucleoside analogs as a relatively unexplored scaffold in drug discovery^57^. Here, we prepared bicyclic nucleoside analogs **86–88** in two steps via a 1,3-syn reduction and indium chloride mediated cyclization^28^ from fluorohydrins **68a**, **69**, and **70** (panel d). Notably, reduction of **68a** afforded preferentially a syn-diol intermediate that was readily cyclized to **86** without epimerization at the anomeric center^28^. Conversely, epimerization of **87** and **88** occurred following cyclization to give a mixture of α-d- and β-d- anomers as shown. Given the synthetic challenges associated with nucleoside analog synthesis, this short sequence provides new opportunities to explore structure activity relationships in this potentially important family of bicyclic nucleoside analogs.

**Fig. 7:**
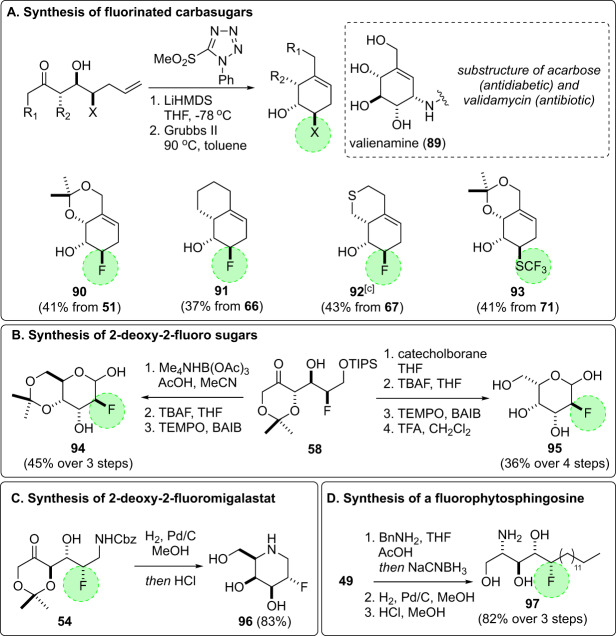
Synthesis of fluorinated glycomimetics. Synthesis of fluorinated glycomimetics including carbasugars (**a**), 2-deoxy-2-fluoro sugars (**b**), a fluorinated iminosugar (**c**), and a fluorinated analog of phytosphingosine (**d**). Fluorine and fluorinated functional groups are highlighted in green. [c] Grela catalyst.

As summarized in Fig. 7, we also explored the utility of fluorinated aldol adducts to serve as precursors to fluorinated glycomimetics. Toward this goal, Julia–Kocienski olefination^58^ and subsequent ring closing metathesis^59^ of **51**, **66**, **67**, and **71** afforded bicyclic fluorinated carbasugars **90–93** in good overall yield (panel a). To access 2-deoxy-2-fluoro sugars, a 1,3-syn or 1,3-anti-selective reduction of the fluorohydrin **58** was followed by removal of the silyl protecting group and oxidation of the resultant primary alcohol (panel b). This short sequence delivered the epimeric 2-deoxy-2-fluoropyranoses **94** and **95** and avoids the iterative alcohol protection–deprotection steps commonly required in fluorosugar synthesis. Hydrogenation of the Cbz-protected amino fluorohydrin **54** (made using d-proline catalysis) gave **96** directly and in excellent yield: a previously undescribed fluorinated analog of the drug migalastat (Galafold)^60^, which is a pharmacological chaperone used to treat Fabry disease (panel c). As an additional target of interest, we also prepared a fluorinated analog of D-ribo-phytosphingosine, a precursor to the potent natural killer T-cell stimulator α-galactosylceramide^61^, in a straightforward manner through the reductive amination of the fluorohydrin **49** with benzyl amine, followed by hydrogenolysis and acid deprotection (panel d).

## Conclusion

In summary, we have developed a flexible and robust DOS approach to glycomimetics that relies on achiral and readily accessible starting materials to access an array of highly relevant carbohydrate-like scaffolds. These processes are enabled by a family of new α-functionalization/aldol reactions that directly transform commercially available aldehydes and ketones into stereochemically rich and densely functionalized aldol adducts in good yield and excellent diastereo- and enantioselectivity. Notably, DFT analysis of aldol reactions of both α-chloro- and α-fluoroaldehydes identified a key stabilizing halogen–hydrogen (C–X···H) interaction in the lowest energy transition structure and support steric and electrostatic interactions playing key diastereodiscriminating roles in these reactions. Ultimately, the demonstration that a broad range of glycomimetics can be readily accessed using these strategies suggests these processes should inspire new efforts in drug discovery.

## Methods

All experimental procedures for α-functionalization/aldol reactions and synthesis of glycomimetics are included in the Supplementary Information (Supplementary Methods).

## Supplementary information

Description of Additional Supplementary Files

Supplemental Material

Supplementary Data 1

Supplementary Data 2

## Data Availability

All characterization data including ^1^H and ^13^C NMR spectral data and sample spectra, IR data, [α]_D_, HRMS, and HPLC chromatograms used to determine enantiomeric purity are included in the Supplementary Information (Supplementary Figs. 1–80). The X-ray crystallographic coordinates for structures reported in this Article have been deposited at the Cambridge Crystallographic Data Centre (CCDC), under deposition numbers CCDC 1556397, 1556396, 1556395, 1556394, and 1556393. These data can be obtained free of charge from The Cambridge Crystallographic Data Centre via www.ccdc.cam.ac.uk/data_request/cif. The crystallographic information of compounds **28**, **47, 56, 58**, and **95** is available in Supplementary Data 2 and is summarized in Supplementary Figs. 81–85 and Supplementary Table 3. The supporting DFT calculations are summarized in Supplementary Fig. 86. Full details for DFT calcuations are available in Supplementary Data File 1 (Supplementary Figs. 87–104 and Supplementary Tables 4–24).
