# Supplementary material for: Diversity-oriented synthesis of glycomimetics
Source: Commun Chem. 2021 Jun 24;4:96. doi: 10.1038/s42004-021-00520-3 (PMC9814271; doi:10.1038/s42004-021-00520-3)
Supplement: Supplementary file 1 — Description of Additional Supplementary Files [file 42004_2021_520_MOESM1_ESM.pdf]

Description of Additional Supplementary Files

File Name: Supplementary Data 1

Description: Full details for DFT calculations

File Name: Supplementary Data 2

Description: The crystallographic information files for compounds 28, 47, 56, 58, and 95
