# Supplementary material for: Diversity-oriented synthesis of glycomimetics
Source: Commun Chem. 2021 Jun 24;4:96. doi: 10.1038/s42004-021-00520-3 (PMC9814271; doi:10.1038/s42004-021-00520-3)
Supplement: Supplementary file 2 — Supplemental Material [file 42004_2021_520_MOESM2_ESM.pdf]

# Supplementary Information

Michael Meanwell[1], Gaelen Fehr[1], Weiwu Ren[1], Bharani Shashank Adluri[1], Victoria Rose[1], Johannes Lehmann[1], Steven M. Silverman[2], Rozhin Rowshanpour[3], Christopher Adamson[1], Milan Bergeron-Brlek[1], Hayden Foy[3], Venugopal Rao Challa[1], Louis-Charles Campeau[2], Travis Dudding[3,*], and Robert Britton[1,*]

*[1] Department of Chemistry, Simon Fraser University, Burnaby, BC, Canada, V5A 1S2*

*[2] Department of Chemistry, Brock University, 1812 Sir Isaac Brock Way, St. Catharines, Ontario, Canada, L2S 3A1*

*[3]Department of Process Research and Development, Merck & Co., Inc. P.O. Box 2000, Rahway, New Jersey 07065, United States. (es).*

* tdudding@brocku.ca

* rbritton@sfu.ca

| | |
|---|---|
| Table of Contents | S1 |
| Supplementary Methods | S1-S52 |
| Supplementary Tables and Figures | S53-S145 |
| Supplementary References | S145 |

## **Suoplementary Methods**

L- and D-proline (99% purity) were purchased from Alfa Aesar. All reactions described were performed at ambient temperature and atmosphere unless otherwise specified. Column chromatography was carried out with 230-400 mesh silica gel (E. Merck, Silica Gel 60). Concentration and removal of trace solvents was done via a Buchi rotary evaporator using acetone-dry-ice condenser and a Welch vacuum pump.

Nuclear magnetic resonance (NMR) spectra were recorded using deuterochloroform ($CDCl_3$), deuteromethanol ($CD_3OD$) or deuterodimethyl sulfoxide (DMSO-$d_6$) as the solvent. Signal

positions (δ) are given in parts per million from tetramethylsilane (δ 0) and were measured relative to the signal of the solvent (**$^{1}$H NMR:** $CDCl_3$: δ 7.26; $CD_3OD$: δ 3.31; DMSO-$d_6$: δ 2.50; **$^{13}$C NMR:** $CDCl_3$: δ 77.16; $CD_3OD$: δ 49.0; DMSO-$d_6$: 39.5). Coupling constants (*J* values) are given in Hertz (Hz) and are reported to the nearest 0.1 Hz. $^{1}$H NMR spectral data are tabulated in the order: multiplicity (*s*, singlet; *d*, doublet; *t*, triplet; *q*, quartet; *sept*, septet; *m*, multiplet; *br* broad), coupling constants, number of protons. NMR spectra were recorded on a Bruker Avance 600 equipped with a QNP or TCI cryoprobe (600 MHz), Bruker 400 (400 MHz) or Bruker 500 (500 MHz). Diastereomeric ratios (dr) are based on analysis of crude $^{1}$H-NMR. Assignments of $^{1}$H are based on analysis of $^{1}$H-$^{1}$H-COSY and nOe spectra. Assignments of $^{13}$C are based on analysis of HSQC spectra.

High performance liquid chromatography (HPLC) analysis was performed on an Agilent 1100 HPLC, equipped with a variable wavelength UV-Vis detector and a chiral column.

High-resolution mass spectra were performed on an Agilent 6210 TOF LC/MS, Bruker MaXis Impact TOF LC/MS, or Bruker micrOTOF-II LC mass spectrometer. For each compound,

Infrared (IR) spectra were recorded neat on a Perkin Elmer Spectrum Two FTIR spectrometer. Only selected, characteristic absorption data are provided for each compound.

Optical rotation was measured on a Perkin-Elmer Polarimeter 341 at 589 nm.

**General Procedure A (α-chlorination/aldol reaction)**

A sample of aldehyde (1.0 equiv.) was added to a stirred suspension of *N*-chlorosuccinimide (1.05 equiv.) and L-proline (0.80 equiv.) in $CH_2Cl_2$ (0.56 M) at 0°C. After 60 minutes, ketone (3.0 equiv.) in DMSO (15.0 M) and $H_2O$ (1% v/v) were added and the resulting reaction mixture was warmed gradually to room temperature. After a total of 24 hrs, or when complete consumption of the aldehyde was observed by $^{1}$H NMR spectroscopic analysis of small reaction aliquots, the reaction mixture concentrated under reduced pressure, and the crude product was purified by flash chromatography as indicated.

**General Procedure B (α-fluorination/aldol reaction with dioxanone)**

A sample of aldehyde (1.5 equiv.) was added to a stirred suspension of *N*-fluorobenzenesulfonimide (1.5 equiv.), L-proline (1.5 equiv.), and $NaHCO_3$ (1.5 equiv.) in DMF (0.75 M) at -10 °C. When complete conversion to the α-fluoroaldehyde was observed by $^{1}$H

NMR spectroscopic analysis, 2,2-Dimethyl-1,3-dioxan-5-one (**13**) (1.0 equiv.) in $CH_2Cl_2$ (0.055 – 0.10 M) was then added and the resulting reaction mixture was allowed to warm gradually to room temperature. After a total of 72 hrs, or when complete consumption of the **13** was observed by $^1$H NMR spectroscopic analysis of small reaction aliquots, the reaction mixture was diluted with $Et_2O$ and the organic layer was washed twice with water and once with brine. The organic layer was then dried over $MgSO_4$, concentrated under reduced pressure and the crude product was purified by flash chromatography as indicated.

**General Procedure C (α-fluorination/aldol reaction with cyclohexanone/thiopyranone 35)**

A sample of aldehyde (1.0 equiv.) was added to a stirred suspension of NFSI (1.0 equiv.), L-proline (1.0 equiv.), and $NaHCO_3$ (1.0 equiv.) in DMF (0.75 M) at -10 °C. When complete conversion to the α-fluoroaldehyde was observed by $^1$H NMR spectroscopic analysis, cyclohexanone or thiopyranone **35** (5.0 – 10.0 equiv.) was then added and the resulting mixture was warmed gradually to room temperature. After a total of 18 hrs, the reaction mixture was diluted with $Et_2O$ and the organic layer was washed twice with water and once with brine. The organic layer was then dried over $MgSO_4$, concentrated under reduced pressure and the crude product was purified by flash chromatography as indicated.

**General Procedure D (α-trifluoromethylthiolation/aldol reaction)**

A sample of aldehyde (2.0 equiv.) was added to a stirred suspension of *N*-trifluoromethylthiophthalimide (2.0 equiv.), L-proline (2.0 equiv.), and $NaHCO_3$ (2.0 equiv.) in DMSO (0.75 M) at room temperature. When complete consumption of aldehyde was observed by $^1$H NMR spectroscopic analysis, 2,2-Dimethyl-1,3-dioxan-5-one (**13**) (1.0 equiv.) in $CH_2Cl_2$ (5 x vol. of DMSO) was then added and the resulting reaction mixture was stirred for a further 48 – 72 hrs. When complete consumption of the **13** was observed by $^1$H NMR spectroscopic analysis of small reaction aliquots, the reaction mixture was diluted with $Et_2O$ and the organic layer was washed twice with water and once with brine. The organic layer was then dried over $MgSO_4$, concentrated under reduced pressure and the crude product was purified by flash chromatography as indicated.

**General Procedure E (olefination/ring-closing metathesis)**

To a stirred solution of 5-(methanesulfonyl)-1-phenyl-1H-tetrazole (2 – 2.2 equiv.) in dry THF (0.80 M) at -78$^o$C was added dropwise a 1 M LiHMDS (2 – 2.2 equiv.) and the resulting reaction

mixture was stirred for 30 minutes. A solution of fluorohydrin (or trifluoromethylthiohydrin) (1.0 equiv.) in dry THF (0.30 – 0.50 M) was then added dropwise and the reaction mixture was stirred for 5 hrs at -78°C. Following complete consumption of fluorohydrin (or trifluoromethylthiohydrin), as monitored by TLC analysis, the reaction mixture was quenched with saturated ammonium chloride solution and diluted with $CH_2Cl_2$. The organic layer was washed twice with water, separated, dried over $MgSO_4$, filtered, and concentrated under reduced pressure. The crude alkene was purified by flash chromatography as indicated. A mixture Grubbs II catalyst (0.05 equiv.) and alkene (1.0 equiv.) in dry toluene (0.025 M) was purged with $N_2$ for 45 minutes in a sealed reaction vessel and subsequently heated to 80 - 90°C for 6 -12 hrs. The reaction mixture was then diluted with $CH_2Cl_2$ and washed twice with water. The organic layer was dried with $MgSO_4$, filtered, and concentrated under reduced pressure. The crude carbacycle was purified by flash chromatography as indicated.

**General Procedure F (reductive amination)**

To a stirred solution of chlorohydrin (1.0 equiv.) in dry THF (0.10 M) was added AcOH (1.0 equiv.) and benzylamine (2 equiv.) and the resulting reaction mixture was allowed to stir for 1 hr. $NaBH_3CN$ (2 equiv.) was added and the reaction mixture stirred for another 1 hr. The reaction mixture was quenched with water and extracted with $CH_2Cl_2$.The organic layer was separated, dried with $Na_2SO_4$, filtered, and concentrated under reduced pressure. The crude product was purified by flash chromatography as indicated.

**General Procedure G (reduction and cyclization)**

To a stirred solution of *syn*-chlorohydrin (1.0 equiv) in MeOH (0.50 M) was added sodium borohydride (2.0 equiv), and the resulting mixture was stirred at 0°C for 1 hour or until no starting material remained (as determined by TLC analysis). The reaction mixture was quenched with saturated $NH_4Cl$, diluted with $CH_2Cl_2$, and washed with $H_2O$. The organic layer was removed, dried over $MgSO_4$, concentrated under reduced pressure, and the crude product was purified by flash chromatography. The resulting diols were dissolved in MeOH (0.50 M) and transferred to a microwave vial. The vial was sealed in a CEM Discover LabMate microwave reactor and subjected to microwave irradiation. The reaction mixture was heated for 5 minutes at 90°C, then 110°C (5 minutes), before heating at 120°C for 108 minutes. The reaction mixture was then cooled and concentrated under reduced pressure. The crude material was purified by flash chromatography as indicated.

**General Procedure H (reduction and benzoylation)**

To a stirred solution of racemic or optically enriched halohydrin (1.0 equiv) in MeOH (0.15 M) was added sodium borohydride (1.5 equiv), and the resulting mixture was stirred at room temperature for 1 hr or until no starting material remained (as determined by TLC analysis). The reaction mixture was then diluted with $CH_2Cl_2$ and washed with $H_2O$. The organic layer was removed, dried over $MgSO_4$, concentrated under reduced pressure, and the crude product was purified by flash chromatography. To a solution of purified diol in $CH_2Cl_2$ (0.10 M) was added triethylamine (6.0 equiv.), either *p*-nitro benzoyl chloride (2.0 - 4.0 equiv.) or *p*-bromo benzoyl chloride (3.0 equiv.), and 4-dimethylaminopyridine (cat.), and left to stir for 1 hr or until no starting material remained (as determined by TLC analysis). The reaction mixture was diluted with $CH_2Cl_2$ and washed with $NaHCO_3$. The organic layer was removed, dried over $MgSO_4$, concentrated under reduced pressure, and the crude product was purified by flash chromatography.

**General Procedure I (hydrogenation)**

Through a solution of fluorohydrin (1.0 equiv) and Pd/C (50 % by weight) in MeOH (0.10 M) was bubbled $H_2$ gas for 1 hr. The reaction vial was then sealed and left over night. The reaction mixture was then filtered, concentrated under reduced pressure, and the crude product was purified by flash chromatography.

**<u>Preparation and characterization of all compounds</u>**

**Preparation of aldol adduct 28**

Following General Procedure B, a solution of pentanal (0.050 mL, 0.470 mmol), NFSI (0.149 g, 0.470 mmol), L-proline (0.054 g, 0.470 mmol), and $NaHCO_3$ (0.039 g, 0.470 mmol) was stirred for 45 minutes at -10 °C in 0.65 mL of DMF. Dioxanone **13** (0.0380 mL, 0.314 mmol) in $CH_2Cl_2$ (5.6 mL) was added and the reaction mixture was left to stir at room temperature for 72 hrs. The ratio of diastereomers was determined to be 15:1 by $^1$H NMR spectroscopic analysis of the crude product. Purification by flash chromatography (pentane:EtOAc – 9:1) afforded *syn*-fluorohydrin **28** (0.045 g, 61 % yield) as a colourless oil.

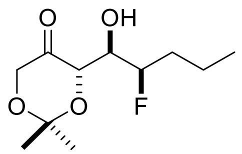

**28**

Data for *syn*-fluorohydrin **28**: **$[\alpha]_D^{20}$** = -6.2 (*c* 2.27 in $CHCl_3$); **IR** (neat): $\upsilon$ = 3429, 2990, 1742, 1376, 1225, 1091, 864 cm$^{-1}$; $^1$**H NMR** (600

MHz, $CDCl_3$): δ 4.69 (dddd, *J* = 47.3, 9.0, 4.0, 1.6 Hz, 1H), 4.39 (dd, *J* = 8.5, 1.1 Hz, 1H), 4.30 (dd, *J* = 17.7, 1.5 Hz, 1H), 4.08 (d, *J* = 17.5 Hz, 1H), 3.80 (ddd, *J* = 27.2, 8.5, 1.5 Hz), 3.29 (s, 1H), 1.94 (m, 1H), 1.61 (m, 1H), 1.52 (m, 1H), 1.50 (s, 3H), 1.43 (s, 3H), 1.42 (m, 1H), 0.97 (t, *J* = 7.0 Hz, 3H); **$^{13}$C NMR** (150 MHz, $CDCl_3$): δ 212.6, 101.8, 91.4 (d, *J* = 175.3), 71.9 (d, *J* = 5.1 Hz), 71.4 (d, *J* = 18.0 Hz), 66.9, 32.6 (d, *J* = 20.8 Hz), 23.8, 23.8, 18.5 (d, *J* = 5.4 Hz), 14.3; **$^{19}$F NMR** (470 MHz, $CDCl_3$): δ –202.4

HRMS ($EI^+$) calcd for $[C_{11}H_{20}FO_4]^+$ 235.1340; found 235.1354

*Determination of the absolute stereochemistry for fluorohydrin* **28**

Following General Procedure H, the fluorohydrin **28** (0.105 g, 0.449 mmol) was converted into the corresponding bis-*p*-bromobenzoate **28-XRD.** Recyrstallization in dichloromethane and ethanol (1:1) allowed for the absolute stereochemistry to be assigned using single X-ray crystallography (see X-ray structures).

*Determination of enantiomeric excess of fluorohydrin* **28**

Following General Procedure B, using a 1:1 mixture of L-:D- proline, a racemic sample of the fluorohydrin **28** was prepared. Following General Procedure H, optically enriched and racemic samples of **28** (0.015 g, 0.064 mmol) were converted into the bis-*p*-nitrobenzoate derivative. The enantiomeric p-nitrobenzoyl diesters were separated by chiral HPLC using a DIACEL CHIRALCEL-OD column; flow rate 1.0 mL/min; eluent: hexanes-*i*PrOH 80:20; detection at 260 nm; retention time = 17.27 min and 22.06 min (see chromatograms).The enantiomeric excess of the optically enriched bis-*p*-nitrobenzoate derivative was determined using the same method (96% ee).

**Preparation of *syn*-bromohydrin 29a and *anti*-bromohydrin 29b**

A solution of pentanal (0.050 mL, 0.47 mmol), NBS (0.092 g, 0.52 mmol), L-proline (0.044 g, 0.38 mmol) and dioxanone **13** (0.059 mL, 0.494 mmol) in $CH_2Cl_2$/DMF (2.82 mL: 0.31 mL) was stirred for 96 hours. The reaction mixture was diluted with $Et_2O$ and the organic layer was washed twice with water and once with brine. The organic layer was then dried over $MgSO_4$, concentrated under reduced pressure Purification of the crude bromohydrins **29** (dr 1.7:1) by flash chromatography (pentane-EtOAc 9:1) afforded *syn*-bromohydrin **29a** (14.4 mg, 11 % yield) as a light brown oil and *anti*-bromohydrin **29b** (8.5 mg, 6 % yield) as a light brown oil.

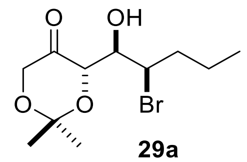

29a

Data for *syn*-bromohydrin **29a**: **$^{1}$H NMR** (600 MHz, $CDCl_3$): δ 4.40 (dd, *J* = 8.7 , 1.2 Hz, 1H), 4.31 (ddd, *J* = 9.4, 5.3, 1.2 Hz, 1H), 4.28 (dd, *J* = 17.6, 1.1 Hz, 1H), 4.07 (d, *J* = 17.6 Hz, 1H), 3.73 (d, *J* = 8.8 Hz, 1H), 3.41 (br s, 1H), 2.10 (m, 1H), 1.85 (m, 1H), 1.61 (m, 1H), 1.54 (s, 3H), 1.46 (m, 1H), 1.42 (s, 3H), 0.96 (t, *J* = 7.4 Hz, 3H); **$^{13}$C NMR** (150 MHz, $CDCl_3$): δ 212.2, 101.8, 74.3, 71.7, 66.6, 57.0, 37.5, 24.2, 23.6, 21.2, 13.6

HRMS ($EI^+$) calcd for $[C_{11}H_{20}{}^{79}BrO_4]^+$ 295.0539; found 295.0556

*Determination of relative stereochemistry for bromohydrin* **29a**

The *syn*-bromohydrin **29a** (10 mg, 0.34 mmol) was converted into a *cis* epoxide to confirm the *syn* stereochemistry of the bromohydrin **29a**. Analysis of the $^{1}$H NMR spectrum indicated a *cis* epoxide as the two epoxide protons resonated at 3.09 and 2.68 ppm.[1]

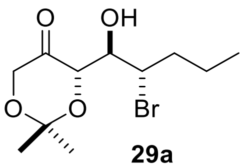

29a

Data for *anti*-bromohydrin **29b**: **$^{1}$H NMR** (600 MHz, $CDCl_3$): δ 4.48 (d, *J* = 7.2, 0.9 Hz, 1H), 4.34 (ddd, *J* = 10.6, 3.5, 3.5 Hz, 1H), 4.29 (dd, *J* = 17.5, 0.9 Hz, 1H), 4.11 (dd, *J* =7.3, 4.2 Hz, 1H), 4.06 (d, *J* = 17.5 Hz, 1H), 3.22 (br s, 1H), 1.93 (m, 1H), 1.80 (m, 1H), 1.67 (m, 1H), 1.50 (s, 3H), 1.44 (s, 3H), 1.43 (m, 1H), 0.94 (t, *J* = 7.4 Hz, 3H); **$^{13}$C NMR** (150 MHz, $CDCl_3$): δ 210.4, 101.5, 74.3, 74.0, 66.8, 57.0, 35.2, 24.0, 23.8, 21.0

HRMS ($EI^+$) calcd for $[C_{11}H_{20}{}^{79}BrO_4]^+$ 295.0539; found 295.0538

*Determination of relative stereochemistry for bromohydrin* **29b**

Following General Procedure B, the *anti*-bromohydrin **29b** (10 mg, 0.34 mmol) was converted into the known epoxide **29b** to confirm *anti* stereochemistry of the bromohydrin.[1]

**Preparation of aldol adduct 30**

Following General Procedure D, a solution of pentanal (0.100 mL, 0.941 mmol), $N(SCF_3)Phth$ (0.232 g, 0.941 mmol), L-proline (0.108 g, 0.941 mmol), and $NaHCO_3$ (0.078 g, 0.941 mmol) was stirred for 50 minutes at RT in DMSO (1.30 mL). Dioxanone **13** (0.044 mL, 0.270 mmol) in $CH_2Cl_2$ (5.6 mL) was stirred for 60 hrs. The ratio of diastereomers was determined to be 6:1 by $^{1}$H NMR spectroscopic analysis of the crude product. Purification by flash chromatography

(pentane:$Et_2O$ – 9:1) afforded *syn*-trifluoromethylthiohydrin **30** (0.082 g, 55 % yield) as a light yellow oil**.**

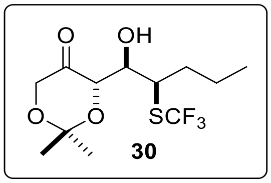

Data for *syn*-trifluoromethylthiohydrin **30**: **$[\alpha]_D^{20}$** = -111.1 (*c* 3.0 in $CH_2Cl_2$); **IR** (neat): $\upsilon$ = 3508, 2961, 1735, 1741, 1377, 1110, 861, 735 $cm^{-1}$; **$^1$H NMR** (600 MHz, $CDCl_3$): δ 4.42 (dd, *J* = 9.1, 1.5 Hz, 1H), 4.30 (dd, *J* = 17.6, 1.5 Hz, 1H), 4.08 (d, *J* = 17.6 Hz, 1H), 4.08 (m, 1H), 3.70 (dd, *J* = 2.2, 1.7 Hz, 1H), 3.46 (m, 1H), 2.00 (m, 1H), 1.87 (m, 1H), 1.48 (s, 3H), 1.42 (s, 3H), 0.95 (dd, *J* = 7.4, 7.4 Hz, 3H); **$^{13}$C NMR** (150 MHz, $CDCl_3$): δ 213.5, 131.6 (q, *J* = 306.6 Hz), 101.7, 72.4, 71.4, 66.5, 47.0, 36.7, 23.8, 23.7, 20.3, 13.8; **$^{19}$F NMR** (470 MHz, $CDCl_3$): δ –39.4

HRMS ($EI^+$) calcd for $[C_{12}H_{19}F_3O_4S + NH_4]^+$ 334.1294; found 334.1303

*Determination of relative stereochemistry for trifluoromethylthiohydrin **30***

Following General Procedure I, trifluoromethylthiohydrin **71** was converted to trifluoromethylthiohydrin **30**. $^1$H NMR analysis revealed identical signals with **30** synthesized using General Procedure D.

*Determination of enantiomeric excess of trifluoromethylthiohydrin **30***

Following General Procedure H, optically enriched and racemic samples of trifluoromethylthiohydrin **30** were converted into their corresponding *p*-nitrobenzoyl diesters. The enantiomeric *p*-nitrobenzoyl diesters were separated by chiral HPLC using a Lux® 3µm Amylose-1 column; flow rate 0.50 mL/min; eluent: hexanes-*i*PrOH 92.5:7.5; detection at 254 nm; retention time = 6.20 min and 9.35 min (see chromatograms). The enantiomeric excess of the optically enriched Bis-PNB ester was determined using the same method (90% ee).

**Preparation of aminohydrin 31 and hydrazone 76**

A solution of pentanal (0.054 mL, 0.55 mmol, 1.1 equiv.), dibenzyl azodicarboxylate (0.149 g, 0.50 mmol, 1.0 equiv.), and L-proline (0.046 g, 0.40 mmol, 0.80 equiv.) in nitromethane (1.2 mL) was stirred until complete consumption of dibenzyl azodicarboxylate was observed by TLC analysis. **13** (0.130 g, 1.0 mmol, 2 equiv.) was then added and the reaction mixture was stirred for 48 hours. The reaction mixture was then diluted with $CH_2Cl_2$, washed with water and brine, dried over MgSO4, filtered, and concentrated under reduced pressure. The crude aldol adduct **31**, without further purification, was dissolved in MeOH (4 mL) containing 1% v/v AcOH. To this

solution, was added Pd/C (100 mg, 25% by weight). H2 gas was bubbled into the reaction mixture until complete consumption of **31** was observed by TLC analysis. The reaction mixture was then filtered through celite and concentrated under reduced pressure. The ratio of diastereomers was determined to be 3:1 by $^{1}$H NMR spectroscopic analysis of the crude product. Purification by flash chromatography (pentane:EtOAc:$NEt_3$ – 60:39:1) afforded hydrazone **76** (0.103 g, 45 % yield over 2 steps) as a white solid.

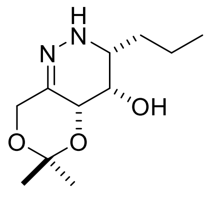

**76**

Data for hydrazone **76**: **$[\alpha]_D^{20}$** = -24.5 (*c* 1.75 in $CHCl_3$); **IR** (neat): $\upsilon$ = 3348, 2989, 2871, 1648, 1455, 1373, 1167, 1074, 862 cm$^{-1}$; **$^{1}$H NMR** (400 MHz, $CDCl_3$): δ 5.13 (br s, 1H), 4.43 (m, 1H), 4.40 (d, *J* = 14.6 Hz, 1H), 4.22 (d, *J* = 14.6 Hz, 1H), 3.87 (m, 1H), 3.14 (dd, *J* = 7.1, 6.8 Hz, 1H), 2.46 (br s, 1H), 1.69 (m, 1H), 1.53 (s, 3H), 1.44 (s, 3H), 1.69 – 1.39 (m, 3H), 0.96 (t, *J* = 7.6 Hz, 3H); **$^{13}$C NMR** (100 MHz, $CDCl_3$): δ 139.6, 99.9, 66.1, 63.1, 62.9, 55.9, 31.7, 27.2, 21.6, 18.8, 14.1

HRMS (EI$^{+}$) calcd for $[C_{11}H_{21}N_2O_3]^+$ 229.1547; found 229.1529

*Determination of enantiomeric excess of hydrazone* **76**

Using a 1:1 mixture of L-:D- proline, a racemic sample of the hydrazone **76** was prepared. The enantiomeric hydrazones were separated by chiral HPLC using a Phenomex Lux Cellulose-3 column; flow rate 0.4 mL/min; eluent: hexanes-*i*PrOH 80:20; detection at 254 nm; retention time = 11.57 min for (+)-**76**; 16.72 min for (-)-**76** (see chromatograms).The enantiomeric excess of the optically enriched **76** was determined using the same method (98% ee).

**Preparation of aldol adduct 36**

Following General Procedure A, isovaleraldehyde (0.054 mL, 0.50 mmol) was added to a mixture of NCS (71 mg, 0.53 mmol) and L-proline (46 mg, 0.40 mmol) in $CH_2Cl_2$ (0.9 mL) at 0°C and the resulting reaction mixture was stirred for 1 hr. Thiopyranone **35** (174 mg, 1.5 mmol) in DMSO (0.1 mL) and $H_2O$ (10 µL) were then added and the resulting reaction mixture was stirred for 48 hrs at room temperature. The ratio of diastereomers was determined to be 10:1 by $^{1}$H NMR spectroscopic analysis of the crude product. Purification by flash chromatography (pentane:EtOAc – 8:2) afforded *syn*-chlorohydrin **36** (0.053 g, 45 % yield) as a white solid.

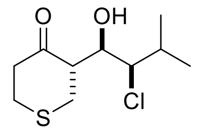

**36**

Data for *syn*-chlorohydrin **36**: **$[\alpha]_D^{20}$** = - 37.4 (*c* = 3.25 in $CHCl_3$); IR (neat): $\upsilon$ = 3539, 2970, 2926, 2872, 1684, 1323, 1307, 1248, 1057, 638, 560, 526 $cm^{-1}$; **$^{1}$H NMR** (400 MHz, $CDCl_3$) δ 4.31 (dd, *J* = 7.4, 3.7 Hz, 1H), 3.68 (dd, *J* = 8.0, 2.5 Hz, 1H), 3.07 (m, 1H) 3.00 - 2.90 (m, 4H), 2.84 - 2.70 (m, 3H), 2.19 (m, *J* = 6.6, 2.0 Hz, 1H), 1.10 (d, *J* = 6.7 Hz, 3H), 1.05 (d, *J* = 6.7 Hz, 3H) **$^{13}$C NMR** (100 MHz, $CDCl_3$) δ 210.8, 70.7, 70.5, 56.5, 44.1, 32.2, 31.9, 30.5, 20.3, 20.1

HRMS (ESI) m/z calcd for $C_{10}H_{21}{}^{35}ClO_2NS$ $[M+NH_4]^+$ 254.0976, found 254.0957

m.p.: 91-93°C

*Determination of relative stereochemistry for chlorohydrin* **36**

Following General Procedure G, the chlorohydrin was converted to **79**. NOE analysis of **94** confirmed relative stereochemistry of chlorohydrin **36**.

*Determination of enantiomeric excess of chlorohydrin* ***36***

Following General Procedure H, *bis*-PNB esters were prepared of both the racemic and enantioenriched samples. The enantiomeric *bis*-PNB esters were separated by chiral HPLC using a Phenomex Lux Amylose-3 column; flow rate 0.4 mL/min; eluent: hexanes-*i*PrOH 90:10; detection at 254 nm; retention time = 10.77 min and 16.02 min (see chromatograms).The enantiomeric excess of the optically enriched **36** was determined using the same method (94% ee).

**Preparation of aldol adduct 38**

Following General Procedure A, isovaleraldehyde (0.108 mL, 1.0 mmol) was added to a mixture of NCS (142 mg, 1.05 mmol) and L-proline (92 mg, 0.80 mmol) in $CH_2Cl_2$ (1.8 mL) at 0°C and the resulting reaction mixture was stirred for 1 hr. Cyclohexanone (0.200 mL, 2.0 mmol) in DMSO (0.2 mL) and $H_2O$ (20 μL) were then added and the resulting reaction mixture was stirred for 48 hrs at room temperature. The reaction mixture was concentrated under reduced pressure. 2 mL of saturated $NH_4Cl$ was added to the resulting crude reaction mixture which was then extracted with diethyl ether. The organic layer was subsequently washed with saturated $NaHCO_3$ and brine, dried over $Na_2SO_4$, filtered, and concentrated under reduced pressure. The ratio of diastereomers was determined to be 10:1 by $^{1}$H NMR spectroscopic analysis of the

crude product. Purification by flash chromatography (pentane:EtOAc – 9:1) afforded *syn*-chlorohydrin **38** (0.104 g, 48 % yield) as a pale yellow oil.

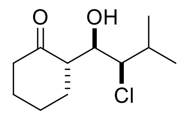
**38**

Data for *syn*-chlorohydrin **38**: **$[\alpha]_D^{20}$** = -13.2 (c = 7.9 in $CHCl_3$) IR (neat): $\upsilon$ = 3530, 2939, 2870, 1695, 1241, 1131, 1603, 732, 531 cm-1 **$^1$H NMR** (500 MHz, $CDCl_3$) δ 4.09 (dd, *J* = 8.4, 2.0 Hz, 1H), 3.58 (dd, *J* = 8.7, 1.8 Hz, 1H), 3.51 (br s, 1H), 2.76 (m, 1H), 2.47 - 2.27 (m, 2H), 2.22 (m, 1H), 2.12 (m, 2H), 1.91 (m, 1H), 1.68 (m, 2H), 1.32 (m, 1H), 1.11 (dd, *J* = 6.8, 1.7 Hz, 3H), 1.02 (dd, *J* = 6.5, 2.0 Hz, 3H) **$^{13}$C NMR** (100 MHz, $CDCl_3$) δ 215.2, 70.9, 70.6, 54.4, 42.6, 32.2, 29.5, 27.5, 24.5, 20.8, 20.1

HRMS: (ESI) m/z calcd for $C_{11}H_{20}{}^{35}ClO_2$ $[M+H]^+$ 219.1146, found 219.1135

*Determination of relative stereochemistry for chlorohydrin* **38**

Following General Procedure G, the chlorohydrin was converted to **78**. NOE analysis of **78** confirmed relative stereochemistry of chlorohydrin **38**.

*Determination of enantiomeric excess of chlorohydrin **38***

Following General Procedure H, *bis*-PNB esters were prepared of both the racemic and enantioenriched samples. The enantiomeric *bis*-PNB esters were separated by chiral HPLC using a Phenomex Lux Amylose-5 column; flow rate 0.25 mL/min; eluent: hexanes-*i*PrOH 97:3; detection at 254 nm; retention time = 6.46 min and 7.08 min (see chromatograms).The enantiomeric excess of the optically enriched **38** was determined using the same method (98% ee).

**Preparation of aldol adduct 39**

Following General Procedure A, isovaleraldehyde (0.054 mL, 0.50 mmol) was added to a mixture of NCS (71 mg, 0.53 mmol) and L-proline (46 mg, 0.40 mmol) in $CH_2Cl_2$ (0.9 mL) at 0°C and the resulting reaction mixture was stirred for 1 hr. O-TBS-hydroxyacetone (282 mg, 1.5 mmol) in DMSO (0.1 mL) and $H_2O$ (10 µL) were then added and the resulting reaction mixture was stirred for 24 hrs at room temperature. The ratio of diastereomers was determined to be 14:1 by $^1$H NMR spectroscopic analysis of the crude product. Purification by flash chromatography (pentane:EtOAc:$NEt_3$ – 95:4:1) afforded *syn*-chlorohydrin **39** (0.108 g, 58 % yield) as a pale yellow oil.

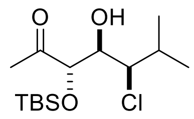

39

Data for *syn*-chlorohydrin **39**: **$[\alpha]_D^{20}$** = -18.9 (c = 6.4 in $CHCl_3$); IR (neat): $\upsilon$ = 3453, 2956, 2930, 1718, 1254, 1102, 835, 777 $cm^{-1}$; **$^1$H NMR** (500 MHz, $CDCl_3$) δ 3.96 (dd, *J* = 7.3, 2.0 Hz, 1H), 3.94 (d, *J* = 8.2 Hz, 1H), 3.82 (d, *J* = 8.2 Hz ,1H), 2.26 (br s, 1H), 2.20 (s, 3H), 2.12 (m, *J* = 6.8 Hz, 1H), 1.07 (d, *J* = 6.6 Hz, 3H), 1.03 (d, *J* = 6.7 Hz, 3H), 0.89 (s, 9H), 0.08 (s, 3H), 0.02 (s, 3H); **$^{13}$C NMR** (125 MHz, $CDCl_3$) δ 209.7, 79.0, 73.0, 70.8, 32.5, 25.6, 25.5, 20.3, 20.0, 18.0, -4.9, -5.2 ppm

HRMS (ESI) m/z calcd for $C_{14}H_{30}{}^{35}ClO_3Si$ $[M+H]^+$ 309.1647, found 309.1666

*Determination of enantiomeric excess of chlorohydrin* ***39***

Following General Procedure H, PNB esters were prepared of both the racemic and enantioenriched samples. The enantiomeric PNB esters were separated by chiral HPLC using a Phenomex Lux i-Cellulose-5 column; flow rate 0.15 mL/min; eluent: hexanes-*i*PrOH 95:5; detection at 254 nm; retention time = 2.89 min and 3.61 min (see chromatograms).The enantiomeric excess of the optically enriched **39** was determined using the same method (97% ee).

**Preparation of chlorohydrin 40**

Following General Procedure A, isovaleraldehyde (0.108 mL, 1.0 mmol) was added to a mixture of NCS (142 mg, 1.05 mmol) and L-proline (90 mg, 0.80 mmol) in $CH_2Cl_2$ (1.8 mL) at 0°C and the resulting reaction mixture was stirred for 1 hr. Cyclohexanone (0.330 mL, 3.0 mmol) in DMSO (0.2 mL) and $H_2O$ (20 µL) were then added and the resulting reaction mixture was stirred for 48 hrs at room temperature. The reaction mixture was concentrated under reduced pressure. 2 mL of saturated $NH_4Cl$ was added to the resulting crude reaction mixture which was then extracted with diethyl ether. The organic layer was subsequently washed with saturated $NaHCO_3$ and brine, dried over $Na_2SO_4$, filtered, and concentrated under reduced pressure. The crude product **40** was then dissolved in MeOH (2 mL) and cooled to 0°C. $NaBH_4$ (74 mg, 2.0 mmol) was added to the reaction mixture and stirred for 1 hr. The reaction mixture was quenched with saturated ammonium chloride and extracted with $CH_2Cl_2$ (10 mL x 3). The combined organic layers were washed with brine, dried over $Na_2SO_4$, filtered, and concentrated under reduced pressure. The ratio of diastereomers was determined to be 7:1 by $^1$H NMR spectroscopic analysis of the crude product. Purification by flash chromatography (pentane:EtOAc – 6:4) afforded *syn*-chlorohydrin **40d** (0.980 g, 44 % yield) as a white solid.

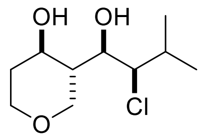
**40d**

Data for *syn*-diol, *syn*-chlorohydrin **40d**: **$[\alpha]_D^{20}$** = - 9.3 (*c* = 6.63 in $CHCl_3$); IR (neat): $\upsilon$ = 3380, 2965, 2872, 1217, 1088, 996, 749, 665, 617 $cm^{-1}$; **1H NMR** (400 MHz, $CDCl_3$) δ 3.99 - 3.86 (m, 3H), 3.78 (ddd, *J* = 7.6, 6.4, 3.2 Hz, 1H), 3.65 (m, *J* = 7.5, 3.2 Hz, 1H), 3.41-3.33 (m, 2H), 3.18 (d, *J* = 6.4 Hz, 1H), 3.08 (dd, *J* = 11.0 Hz, 1H), 2.17 (m, *J* = 6.7 Hz, 1H), 2.01 - 1.89 (m, 2H), 1.65 (m, 1H), 1.06 (d, *J* = 6.7 Hz, 3H), 1.02 (d, *J* = 6.7 Hz, 3H); **$^{13}$C NMR** (100 MHz, $CDCl_3$) δ 74.2, 72.7, 71.5, 67.6, 66.5, 46.5, 34.8, 31.8, 20.4, 19.8.

HRMS (ESI) m/z calcd for $C_{10}H_{20}{}^{35}ClO_3$ $[M+H]^+$ 223.1095, found 223.1082

m.p.: 68 - 72°C

*Determination of relative stereochemistry for chlorohydrin* **40**

Following General Procedure G, the chlorohydrin was converted to **80**. NOE analysis of **80** confirmed relative stereochemistry of chlorohydrin **40**.

*Determination of enantiomeric excess of chlorohydrin **40***

Following General Procedure H, PNB esters were prepared of both the racemic and enantioenriched samples of **80**. The enantiomeric PNB esters were separated by chiral HPLC using a Phenomex Lux Cellulose-3 column; flow rate 0.40 mL/min; eluent: hexanes-*i*PrOH 90:10; detection at 254 nm; retention time = 1.57 min and 2.50 min (see chromatograms).The enantiomeric excess of the optically enriched **80** was determined using the same method (93% ee).

**Preparation of aldol adduct 47**

Following General Procedure B, a solution of propanal (0.050 mL, 0.687 mmol), NFSI (0.217 g, 0.687 mmol), L-proline (0.079 g, 0.687 mmol) and $NaHCO_3$ (0.058 g, 0.687 mmol) was stirred for 45 minutes at -10 °C in DMF (0.92 mL). Dioxanone **13** (0.055 mL, 0.458 mmol) in $CH_2Cl_2$ (8.3 mL) was then added and the reaction mixture was stirred for 48 hrs. The ratio of diastereomers was determined to be 8:1 by $^1$H NMR spectroscopic analysis of the crude product. Purification by flash chromatography (pentane-EtOAc - 4:1) afforded *syn*-fluorohydrin **47** (0.053 g, 56 % yield) as a yellow oil.

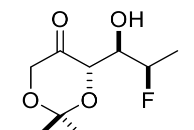

47

Data for *syn*-fluorohydrin **47**: **$[\alpha]_D^{20}$** = -13.5 (*c* 3.42 in $CHCl_3$); **IR** (neat): $\upsilon$ = 3428, 2990, 1742, 1378, 1225, 1091, 864 $cm^{-1}$;**$^1$H NMR** (600 MHz, $CDCl_3$): δ 4.90 (dq, *J* = 47.0 Hz, 6.5 Hz, 1H), 4.38 (dd, *J* = 8.5, 1.4 Hz, 1H), 4.30 (dd, *J* = 17.6, 1.5 Hz, 1H), 4.08 (d, *J* = 17.6, 1H), 3.75 (ddd, *J* = 26.1, 2.5, 2.5, 1H), 3.29 (d, *J* = 2.7), 1.50 (s, 3H), 1.43 (s, 3H), 1.43 (dd, *J* = 24.0, 6.6 Hz, 3H); **$^{13}$C NMR** (150 MHz, $CDCl_3$): δ 212.1, 101.6, 88.0 (d, *J* = 171.0 Hz) , 72.3 (d, *J* = 17.7 Hz), 72.0 (d, *J* = 5.1 Hz), 66.8, 23.7, 23.7, 16.4 (d, *J* = 22.9 Hz); **$^{19}$F NMR** (470 MHz, $CDCl_3$): δ -195.5

HRMS ($EI^+$) calcd for $[C_9H_{16}FO_4]^+$ 207.1027; found 207.1054

*Determination of relative stereochemistry for fluorohydrin* **47**

Following General Procedure H, the fluorohydrin **47** (0.105 g, 0.449 mmol) was converted into the corresponding *bis*-PNB ester (**47**-**XRD**). Recyrstallization in ethanol allowed for the relative stereochemistry to be assigned using single X-ray crystallography (see X-ray structures).

*Determination of enantiomeric excess of fluorohydrin* **47**

Following General Procedure B, using a 1:1 mixture of L-:D- proline, a racemic sample of the fluorohydrin **47** was prepared. Following General Procedure H, optically enriched and racemic samples of **47** (0.040 g, 0.19 mmol) were converted into the corresponding bis-*p*-nitrobenzoate derivative. The enantiomeric *p*-nitrobenzoyl diesters were separated by chiral HPLC using a DIACEL CHIRALCEL-OD column; flow rate 1.0 mL/min; eluent: hexanes-*i*PrOH 80:20; detection at 260 nm; retention time = 28.1 min and 37.4 min (see chromatograms). The enantiomeric excess of the optically enriched *bis*-PNB derivative was determined using the same method (95% ee).

**Preparation of aldol adduct 48**

Following General Procedure B, a solution of 3-methylbutanal (0.050 mL, 0.465 mmol), NFSI (0.147 g, 0.465 mmol), L-proline (0.053 g, 0.465 mmol), and $NaHCO_3$ (0.039 g, 0.465 mmol) was stirred for 45 minutes at -10 °C in 0.60 mL of DMF. Dioxanone **13** (0.037 mL, 0.31 mmol) in $CH_2Cl_2$ (5.4 mL) was stirred for 72 hrs. The ratio of diastereomers was determined to be >20:1 by $^1$H NMR spectroscopic analysis of the crude product. Purification by flash chromatography (pentane-EtOAc - 4:1) afforded *syn*-fluorohydrin **48** (0.049 g, 67 % yield) as a colorless oil**.**

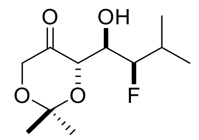

**48**

Data for *syn*-fluorohydrin **48**: **[$\alpha$]$_D^{20}$** = -117 (*c* 2.6 in $CHCl_3$); **IR** (neat): $\upsilon$ = 3526, 2968, 1740, 1377, 864; **$^1$H NMR** (600 MHz, $CDCl_3$): δ 4.39 (d, *J* = 8.7 Hz, 1H), 4.30 (d, *J* = 17.8 Hz, 1H), 4.21 (dd, *J* = 46.5, 9.6 Hz, 1H), 4.08 (d, *J* = 17.6 Hz, 1H), 3.95 (dd, *J* = 28.6, 8.8 Hz, 1H), 3.26 (d, *J* = 2.5 Hz, 1H), 2.26 (m, 1H), 1.50 (s, 3H), 1.43 (s, 3H), 1.07 (d, *J* = 6.6 Hz, 3H), 0.92 (d, *J* =6.6 Hz, 3H); **$^{13}$C NMR** (150 MHz, $CDCl_3$): δ 212.8, 101.6, 96.1 (d, *J* = 178.1 Hz), 71.5 (d, *J* = 6.2 Hz), 69.2 (d, *J* = 18.4 Hz), 66.7, 28.1 (d, *J* = 20.0 Hz), 23.6, 23.6, 19.1 (d, *J* = 4.8 Hz), 18.1 (d, *J* = 9.3 Hz); **$^{19}$F NMR** (470 MHz, $CDCl_3$): δ –203.3

HRMS ($EI^+$) calcd for $[C_{11}H_{20}FO_4]^+$ 235.1340; found 235.1334

*Determination of enantiomeric excess of fluorohydrin* **48**

Following General Procedure B, using a 1:1 mixture of L-: D-proline, a racemic sample of the fluorohydrin **48** was prepared. Following General Procedure H, optically enriched and racemic samples of **48** (0.035 g, 0.15 mmol) were converted into the corresponding bis-*p*-nitrobenzoate derivative. The enantiomeric fluorohydrin were separated by chiral HPLC using a DIACEL CHIRALCEL-OD column; flow rate 1.0 mL/min; eluent: hexanes-*i*PrOH 93.5:6.5; detection at 260 nm; retention time = 24.94 min and 27.31 (see chromatograms). The enantiomeric excess of the optically pure *bis*-PNB derivative was determined using the same method (95 % ee).

**Preparation of aldol adduct 49**

Following General Procedure B, a solution of pentadecanal (0.453 g, 2.0 mmol), NFSI (0.731 g, 2.0 mmol), D-proline (0.23 g, 2.0 mmol) and $NaHCO_3$ (0.168 g, 2.0 mmol) was stirred for 3 hrs at -10 °C in DMF (2.7 mL). Dioxanone **13** (0.287 mL, 1.33 mmol) in $CH_2Cl_2$ (24 mL) was stirred for 48 hours. The ratio of diastereomers was determined to be 15:1 by $^1$H NMR spectroscopic analysis of the crude product. Purification by flash chromatography (pentane-EtOAc - 30:1) afforded *syn*-fluorohydrin **49** (0.239 g, 48% yield) as a clear oil**.**

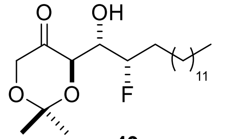

**49**

Data for *syn*-fluorohydrin **49**: **$[\alpha]_D^{20}$** = -83.0 (*c* 1.2 in $CHCl_3$); **IR** (neat): $\upsilon$ = 3530, 2924, 2854, 1740, 1337, 1224, 1090, 865 $cm^{-1}$; **$^1$H NMR** (600 MHz, $CDCl_3$): δ 4.66 (ddd, J = 47.3, 8.9, 4.9 Hz, 1H), 4.38 (dd, J = 8.7, 1.4 Hz, 1H), 4.30 (dd, J = 17.5, 1.4 Hz, 1H), 4.07 (d, J = 17.5 Hz, 1H), 3.79 (dddd, J = 27.1, 8.5, 3.1, 1.7 Hz, 2H), 3.31 (d, J = 3.1 Hz, 1H), 1.92 (m, 1H), 1.25-1.63 (26 H), 0.88 (dd, J =6.6, 6.6 Hz, 3H); **$^{13}$C NMR** (150 MHz, $CDCl_3$): δ 212.3, 101.6, 91.5 (d, J = 174.9 Hz) , 71.7 (d, J = 5.3 Hz), 71.2 (d, J = 18.5 Hz), 66.7, 32.1, 30.4 (d, J = 21.3 Hz), 29.8, 29.8, 29.8, 29.8, 29.6, 29.6, 29.5, 25.4, 25.3, 23.6, 23.6, 22.8, 14.2; **$^{19}$F NMR** (470 MHz, $CDCl_3$): δ –201.9

HRMS ($EI^+$) calcd for $[C_{21}H_{39}FNaO_4]^+$ 397.2725; found 397.2755

*Determination of relative stereochemistry for fluorohydrin* **49**

Analysis of $^1$H-NMR of fluorohydrins **28** and **49** revealed identical signals between 1.60 and 4.70 ppm indicating the two compounds share the same relative stereochemistry.

*Determination of enantiomeric excess of fluorohydrin* **49**

Following General Procedure B, using a 1:1 mixture of L-:D- proline, a racemic sample of the fluorohydrin **49** was prepared. Following General Procedure H, optically enriched and racemic samples of **49** (0.050 g, 0.13 mmol) were converted into the corresponding bis-*p*-nitrobenzoate derivative. The enantiomeric p-nitrobenzoyl esters were separated by chiral HPLC using a DIACEL CHIRALCEL-OD column; flow rate 1.0 mL/min; eluent: hexanes-*i*PrOH 80:20; detection at 260 nm; retention time = 8.76 min and 12.06 min (see chromatograms). The enantiomeric excess of the optically enriched bis-*p*-nitrobenzoate derivative was determined using the same method (91% ee).

**Preparation of aldol adduct 50**

Following General Procedure B, a solution of 4-methyl-4-nitropentanal (0.050 mL, 0.379 mmol), NFSI (0.119 g, 0.379 mmol), L-proline (0.044 g, 0.379 mmol), and $NaHCO_3$ (0.032 g, 0.379 mmol) was stirred for 120 minutes at -10 °C in 0.50 mL of DMF. Dioxanone **13** (0.030 mL, 0.253 mmol) in $CH_2Cl_2$ (5.5 mL) was stirred for 72 hrs. The ratio of diastereomers was determined to be >20:1 by $^1$H NMR spectroscopic analysis of the crude product. Purification by flash chromatography (pentane-EtOAc - 3:1) afforded *syn*-fluorohydrin **50** (0.034 g, 46 % yield) as a colorless oil**.**

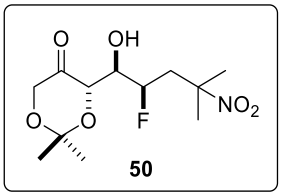

Data for *syn*-fluorohydrin **50**: **$[\alpha]_D^{20}$** = -71.6 (*c* 1.9 in $CHCl_3$); **IR** (neat): $\upsilon$ = 3515, 2989, 1741, 1540, 1376, 861, $cm^{-1}$;**$^1$H NMR** (500 MHz, $CDCl_3$): δ 4.77 (ddd, *J* = 48.7, 10.0, 1.0 Hz, 1H), 4.35 (dd, *J* = 9.0, 1.3 Hz, 1H), 4.30 (dd, *J* = 17.7, 1.4 Hz, 1H), 4.08 (d, *J* = 17.7 Hz, 1H), 3.76 (dddd, *J* = 27.4, 8.9, 2.3, 2.3 Hz, 1H), 3.43 (d, *J* = 2.3 Hz, 1H), 2.57 (m, 1H), 2.30 (m, 1H), 1.69 (s, 3H), 1.67 (s, 3H), 1.48 (s, 3H), 1.40 (s, 3H); **$^{13}$C NMR** (150 MHz, $CDCl_3$): δ 212.5, 101.8, 87.9 (d, *J* = 176.9 Hz), 87.1, 72.2 (d, *J* = 18.6 Hz), 71.2 (d, *J* = 5.5 Hz) 66.5, 41.3 (d, *J* = 20.5 Hz), 28.0, 25.4, 23.6, 23.5; **$^{19}$F NMR** (470 MHz, $CDCl_3$): δ –201.4

HRMS ($EI^+$) calcd for $[C_{12}H_{21}FNO_6]^+$ 294.1347; found 294.1359

*Determination of enantiomeric excess of fluorohydrin* **50**

Following General Procedure B, using a 1:1 mixture of L-:D- proline, a racemic sample of the fluorohydrin **50** was prepared. Following General Procedure H, optically enriched and racemic samples of **50** (0.050 g, 0.171 mmol) were converted into the corresponding bis-*p*-nitrobenzoate derivative. The enantiomeric fluorohydrin were separated by chiral HPLC using a DIACEL CHIRALCEL-OD column; flow rate 1.5 mL/min; eluent: hexanes-*i*PrOH 80:20; detection at 260 nm; retention time = 34.51 min and 37.98 (see chromatograms). The enantiomeric excess of the optically pure bis-*p*-nitrobenzoate derivative was determined using the same method (95% ee).

**Preparation of aldol adduct 51**

Following General Procedure B, a solution of pentenal (0.050 g, 0.60 mmol), NFSI (0.189 g, 0.60 mmol), L-proline (0.069 g, 0.60 mmol) and $NaHCO_3$ (0.055 g, 0.60 mmol) was stirred at -10 °C in DMF (0.80 mL) for 1 hr. Dioxanone **13** (0.048 mL, 0.40 mmol) in $CH_2Cl_2$ (7.2 mL) was stirred for 72 hours. The ratio of diastereomers was determined to be 5:1 by $^1$H NMR spectroscopic analysis of the crude product. Purification by flash chromatography (pentane-EtOAc - 85:15) afforded *syn*-fluorohydrin **51** (0.059 g, 64 % yield) as a light yellow oil**.**

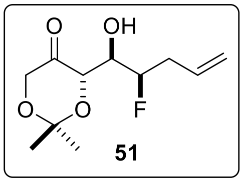

Data for *syn*-fluorohydrin **51**: **$[\alpha]_D^{20}$** = -115.8 (*c* 2.46 in $CHCl_3$); **IR** (neat): $\upsilon$ = 3509, 2989, 1740, 1643, 1422, 1377, 1089, 863 $cm^{-1}$; **$^1$H NMR** (600 MHz, $CDCl_3$): δ 5.83 (m, 1H), 5.20 (d, *J* = 17.4 Hz, 1H), 5.14 (d, *J* = 9.9, 1.0 Hz, 1H), 4.73 (dddd, *J* = 47.0, 7.0, 7.0, 1.3 Hz, 1H), 4.39 (dd, *J* = 8.9, 1.0 Hz, 1H), 4.30 (dd, *J* = 17.7, 1.0 Hz), 4.08 (d, *J* = 17.6), 2.69 (m, 1H), 2.48 (m, 1H) 1.49 (s, 3H), 1.43 (s, 3H);**$^{13}$C NMR** (150 MHz, $CDCl_3$): δ 212.4, 133.0 (d, *J* = 7.6 Hz), 118.5, 101.6, 90.5 (d, *J*

= 178.0 Hz), 71.5 (d, $J$ = 5.3 Hz), 70.5 (d, $J$ = 18.2 Hz), 66.6, 34.9 (d, $J$ = 22.2 Hz), 23.6, 23.6; **$^{19}$F NMR** (470 MHz, $CDCl_3$): δ –201.6

HRMS (EI$^+$) calcd for $[C_{11}H_{18}FO_4]^+$ 233.1184; found 233.1202

*Determination of relative stereochemistry for fluorohydrin* **51**

Following General Procedure I, the fluorohydrin **51** (0.105 g, 0.45 mmol) was converted to the fluorohydrin **28**. Comparison of $^1$H and $^{19}$F NMR with fluorohydrin **28** confirmed relative stereochemistry.

*Determination of the absolute stereochemistry for fluorohydrin* **51**

Following General Procedure I, the fluorohydrin **51** (0.105 g, 0.45 mmol) was converted to the fluorohydrin **28**. Comparison of $[\alpha]_D$ values with fluorohydrin **28** confirmed absolute stereochemistry

*Determination of enantiomeric excess of fluorohydrin* **51**

Following General Procedure I, the optically enriched sample of **51** (0.105 g, 0.45 mmol) was converted into fluorohydrin **28**. Following General Procedure H, the optically enriched and racemic samples of fluorohydrin **28** were converted into their corresponding bis-*p*-nitrobenzoate derivative. The enantiomeric *p*-nitrobenzoyl diesters were separated by chiral HPLC using a DIACEL CHIRALCEL-OD column; flow rate 1.0 mL/min; eluent: hexanes-*i*PrOH 80:20; detection at 260 nm; retention time = 17.27 min and 22.06 min (see chromatograms). The enantiomeric excess of the optically enriched bis-*p*-nitrobenzoate derivative was determined using the same method (93 % ee).

**Preparation of aldol adduct 52**

Following General Procedure B, a solution of 3-(4-methoxyphenyl)propanal (0.050 mL, 0.317 mmol), NFSI (0.100 g, 0.317mmol), L-proline (0.037 g, 0.317 mmol), and $NaHCO_3$ (0.027 g, 0.317 mmol) was stirred for 90 minutes at -10 °C in 0.43 mL of DMF. Dioxanone **13** (0.025 mL, 0.211 mmol) in $CH_2Cl_2$ (3.8 mL) was stirred for 72 hrs. The ratio of diastereomers was determined to be >20:1 by $^1$H NMR spectroscopic analysis of the crude product. Purification by flash chromatography (pentane-EtOAc - 4:1) afforded *syn*-fluorohydrin **52** (0.034 g, 51 % yield) as a white solid**.**

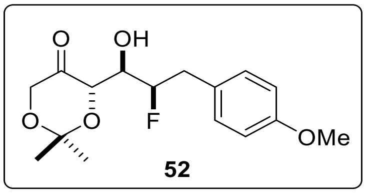

Data for *syn*-fluorohydrin **52**: **[$\alpha$]$_D$$^{20}$** = -233.4 (*c* 3.0 in $CHCl_3$); $^1$**H NMR** (600 MHz, $CDCl_3$): δ 7.20 (d, *J* = 8.8 Hz, 2H), 6.86 (d, *J* = 8.8Hz, 2H) 4.81 (dddd, *J* = 46.8, 7.3, 7.3 Hz, 0.8 Hz, 1H), 4.40 (d, *J* = 8.8 Hz, 1H), 4.26 (d, *J* = 17.8 Hz, 1H), 4.07 (d, *J* = 17.8 Hz, 1H), 3.79 (s, 3H), 3.77 (d, *J* = 24.1, 8.8 Hz, 1H), 3.39 (br s, 1H), 3.15 (m, 1H), 2.99 (m, 1H), 1.46 (s, 3H), 1.39 (s, 3H); $^{13}$**C NMR** (150 MHz, $CDCl_3$): δ 212.7, 158.6, 130.6, 128.9 (d, *J* = 8.0 Hz), 114.1, 101.7, 91.9 (d, *J* = 179.2 Hz), 71.4 (d, *J* = 5.1 Hz), 70.1 (d, *J* =18.1 Hz), 66.6, 55.4, 35.8 (d, *J* = 22.4 Hz), 23.6, 23.5; $^{19}$**F NMR** (470 MHz, $CDCl_3$): δ –200.1

HRMS (EI$^+$) calcd for $[C_{16}H_{22}FO_5]^+$ 313.1446; found 313.1450

*Determination of enantiomeric excess of fluorohydrin* **52**

Following General Procedure B, using a 1:1 mixture of L-:D- proline, a racemic sample of the fluorohydrin **52** was prepared. Following General Procedure H, optically enriched and racemic samples of **52** (0.040 g, 0.128 mmol) were converted into the corresponding bis-*p*-nitrobenzoate derivative. The enantiomeric fluorohydrin were separated by chiral HPLC using a DIACEL CHIRALCEL-OD column; flow rate 1.5 mL/min; eluent: hexanes-*i*PrOH 93.5:6.5; detection at 260 nm; retention time = 74.8 min and 110.4 min (see chromatograms). The enantiomeric excess of the optically pure bis-*p*-nitrobenzoate derivative was determined using the same method (95% ee).

**Preparation of aldol adduct 53**

Following General Procedure B, a solution of pentynal (0.050 g, 0.61 mmol), NFSI (0.192 g, 0.61 mmol), L-proline (0.070 g, 0.61 mmol) and $NaHCO_3$ (0.051 g, 0.61 mmol) was stirred for 1.5 hrs at -10°C in DMF (0.81 mL). Dioxanone **13** (0.049 mL, 0.41 mmol) in $CH_2Cl_2$ (7.3 mL) was stirred for 48 hours. The ratio of diastereomers was determined to be 4.5:1 by $^1$H NMR spectroscopic analysis of the crude product. Purification by flash chromatography (pentane-EtOAc - 9:1) afforded *syn*-fluorohydrin **53** (0.052 g, 55 % yield) as a light yellow oil**.**

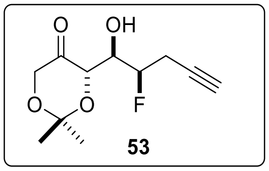

Data for *syn*-fluorohydrin **53**: **[$\alpha$]$_D$$^{20}$** = -62.9 (*c* 3.42 in $CHCl_3$); **IR** (neat): $\upsilon$ = 3512, 3293, 2993, 1743, 1378, 1224, 1033 cm$^{-1}$; $^1$**H NMR** (500 MHz, $CDCl_3$): δ 4.86 (dddd, *J* = 46.4, 7.3, 7.3, 1.5 Hz, 1H), 4.38 (dd, *J* = 8.9, 1.5 Hz, 1H), 4.31 (dd, *J* = 17.6, 1.6 Hz, 1H), 4.10 (d, *J* = 17.7 Hz, 1H), 4.00 (ddd, *J* = 27.9, 9.1, 1.5 Hz, 1H), 2.76 (m, 2H), 2.04 (t, *J* = 2.8 Hz, 1H), 1.49 (s, 3H), 1.43

(s, 3H); **$^{13}$C NMR** (150 MHz, $CDCl_3$): δ 212.2, 101.7, 89.0 (d, *J* = 180.0 Hz), 79.0 (d, *J* = 15.4 Hz), 71.3 (d, *J* = 5.1 Hz), 70.8, 69.7 (d, *J* = 17.4 Hz), 66.6, 23.7, 23.6, 20.4 (d, *J* = 28.9 Hz); **$^{19}$F NMR** (470 MHz, $CDCl_3$): δ –200.1

HRMS ($EI^+$) calcd for $[C_{11}H_{16}FO_4]^+$ 231.1027; found 231.1042

*Determination of relative stereochemistry for fluorohydrin* **53**

Following General Procedure **I**, the fluorohydrin **53** (0.10 g, 0.43 mmol) was converted to the fluorohydrin **28**. Comparison of $^{1}$H and $^{19}$F NMR with fluorohydrin **28** confirmed relative stereochemistry.

*Determination of the absolute stereochemistry for fluorohydrin* **53**

Following General Procedure **F**, the fluorohydrin **53** (0.10 g, 0.43 mmol) was converted to the fluorohydrin **28**. Comparison of **$[\alpha]_D$** values with fluorohydrin **28** confirmed absolute stereochemistry.

*Determination of enantiomeric excess of fluorohydrin* ***53***

Following General Procedure **I**, the optically enriched sample of **53** (0.10 g, 0.43 mmol) was converted into fluorohydrin **28**. Following General Procedure **H**, the optically enriched and racemic samples of fluorohydrin **28** were converted into their corresponding bis-p-nitrobenzoate derivative. The enantiomeric p-nitrobenzoyl diesters were separated by chiral HPLC using a DIACEL CHIRALCEL-OD column; flow rate 1.0 mL/min; eluent: hexanes-iPrOH 80:20; detection at 260 nm; retention time = 17.27 min and 22.06 min (see chromatograms). The enantiomeric excess of the optically enriched bis-p-nitrobenzoate derivative was determined using the same method (92% ee).

**Preparation of aldol adduct 54**

Following General Procedure B, a solution of 3-(4-bromophenyl)propanal (0.050 g, 0.236 mmol), NFSI (0.074 g, 0.236 mmol), L-proline (0.028 g, 0.236 mmol), and $NaHCO_3$ (0.020 g, 0.236 mmol) was stirred for 120 minutes at -10 °C in 0.30 mL of DMF. Dioxanone **13** (0.022 mL, 0.157 mmol) in $CH_2Cl_2$ (2.8 mL) was stirred for 72 hrs. The ratio of diastereomers was determined to be >20:1 by $^{1}$H NMR spectroscopic analysis of the crude product. Purification by

flash chromatography (pentane-EtOAc - 4:1) afforded *syn*-fluorohydrin **55** (0.034 g, 61 % yield) as a white solid**.**

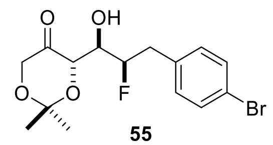
**55**

Data for *syn*-fluorohydrin **55** **[$\alpha$]$_D^{20}$** = -76.4 (*c* 0.8 in $CHCl_3$); **IR** (neat): $\upsilon$ = 3513, 2989, 1740, 1490, 1377, 864cm$^{-1}$;**$^1$H NMR** (600 MHz, $CDCl_3$): δ 7.44 (d, *J* = 8.2 Hz), 7.16 (d, *J* = 8.2 Hz), 4.80 (ddd, *J* = 46.8, 7.2, 7.2, 0.9 Hz, 1H), 4.40 (d, *J* = 18.9 Hz, 1H), 4.27 (d, *J* = 17.7 Hz, 1H), 4.08 (d, *J* = 17.7 Hz, 1H), 3.75 (dd, *J* = 27.6, 9.0 Hz, 1H), 3.42 (br s, 1H), 3.18 (m, 1H), 2.97 (m, 1H), 1.46 (s, 3H), 1.39 (s, 3H); **$^{13}$C NMR** (150 MHz, $CDCl_3$): δ 212.6, 136.0 (d, *J* = 7.2 Hz), 131.8, 131.4, 120.8, 101.7, 91.4 (d, *J* = 179.9 Hz) , 71.3 (d, *J* = 5.0 Hz), 70.2 (d, *J* = 17.1 Hz), 66.6, 36.2 (d, *J* = 22.4 Hz), 23.6, 23.5; **$^{19}$F NMR** (470 MHz, $CDCl_3$): δ –200.6

HRMS (EI$^+$) calcd for $[C_{15}H_{19}{}^{79}BrFO_4]^+$ 361.0445; found 361.0434

*Determination of enantiomeric excess of fluorohydrin* **55**

Following General Procedure B, using a 1:1 mixture of L-:D- proline, a racemic sample of the fluorohydrin **55** was prepared. Following General Procedure H, optically enriched and racemic samples of **55** (0.037 g, 0.103 mmol) were converted into the corresponding bis-*p*-nitrobenzoate derivative. The enantiomeric fluorohydrin were separated by chiral HPLC using a DIACEL CHIRALCEL-OD column; flow rate 1.0 mL/min; eluent: hexanes-*i*PrOH 80:20; detection at 260 nm; retention time = 52.3 min and 63.5 min (see chromatograms). The enantiomeric excess of the optically pure bis-*p*-nitrobenzoate derivative was determined using the same method (95% ee).

**Preparation of aldol adduct 56**

Following General Procedure B, a solution of hydrocinnamaldehyde (0.050 mL, 0.38 mmol), NFSI (0.120 g, 0.38 mmol), L-proline (0.044 g, 0.38 mmol), and $NaHCO_3$ (0.032 g, 0.38 mmol) was stirred for 75 minutes at -10 °C in 0.50 mL of DMF. Dioxanone **13** (0.036 mL, 0.30 mmol) in $CH_2Cl_2$ (4.5 mL) was stirred for 72 hrs. The ratio of diastereomers was determined to be >15:1 by $^1$H NMR spectroscopic analysis of the crude product. Purification by flash chromatography (pentane-EtOAc - 4:1) afforded *syn*-fluorohydrin **56** (0.053 g, 62 % yield, d.r. > 15:1) as a colorless oil**.**

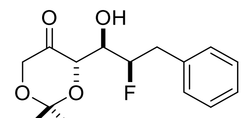

56

Data for *syn*-fluorohydrin **56**: **[$\alpha$]$_D^{20}$** = -3.1 (*c* 2.1 in $CHCl_3$); **IR** (neat): $\upsilon$ = 3511, 2923, 1739, 1705, 1650, 1585, 1453, 863, 698 $cm^{-1}$; **$^1$H NMR** (600 MHz, $CDCl_3$): δ 7.30 (m, 5H), 4.87 (ddd, *J* = 46.5, 7.0, 7.0 Hz, 1H), 4.42 (dd, *J* = 9.0, 0.9 Hz, 1H), 4.27 (dd, *J* = 17.7, 1.0 Hz, 1H), 4.08 (d, *J* = 17.6 Hz, 1H), 3.79 (dd, *J* = 27.6, 9.0 Hz, 1H), 3.42 (br s, 1H), 3.24 (m, 1H), 3.05 (m, 1H), 1.48 (s, 3H), 1.40 (s, 3H); **$^{13}$C NMR** (150 MHz, $CDCl_3$): δ 212.6, 136.9 (d, *J* = 8.1 Hz), 129.6, 128.7, 126.8, 101.7, 91.7 (d, *J* = 177.8 Hz) , 71.4 (d, *J* = 5.2 Hz), 70.2 (d, *J* = 18.1 Hz), 66.6, 36.7 (d, *J* = 22.4 Hz), 23.6, 23.5; **$^{19}$F NMR** (470 MHz, $CDCl_3$): δ –200.1

HRMS ($EI^+$) calcd for $[C_{15}H_{20}FO_4]^+$ 283.1340; found 283.1365

*Determination of relative stereochemistry for fluorohydrin* **56**

Reduction with sodium borohydride of the fluorohydrin **56** (0.105 g, 0.37 mmol) in methanol allowed for conversion to the corresponding *syn*-diol **56-XRD**. Recyrstallization in ethanol (1:1) allowed for the relative stereochemistry to be assigned using single X-ray crystallography (see X-ray structures)

*Determination of enantiomeric excess of fluorohydrin* **56**

Following General Procedure B, using a 1:1 mixture of L-:D- proline, a racemic sample of the fluorohydrin **56** was prepared. The enantiomeric fluorohydrin were separated by chiral HPLC using a DIACEL CHIRALCEL-OD column; flow rate 1.5 mL/min; eluent: hexanes-*i*PrOH 97:3; detection at 260 nm; retention time = 12.80 min and 13.64 min (see chromatograms). The enantiomeric excess of the optically pure **56** was determined using the same method (98% ee).

**Preparation of aldol adduct 57**

Following General Procedure B, a solution of 3-(5-methylfuran-2-yl)propanal (0.050 mL, 0.376 mmol), NFSI (0.119 g, 0.376 mmol), L-proline (0.029 g, 0.376 mmol), and $NaHCO_3$ (0.032 g, 0.376 mmol) was stirred for 90 minutes at -10 °C in 0.50 mL of DMF. Dioxanone **13** (0.030 mL, 0.251 mmol) in $CH_2Cl_2$ (4.5 mL) was stirred for 72 hrs. The ratio of diastereomers was determined to be >20:1 by $^1$H NMR spectroscopic analysis of the crude product. Purification by flash chromatography (pentane-EtOAc - 5:1) afforded *syn*-fluorohydrin **57** (0.038 g, 53 % yield) as a colorless oil.

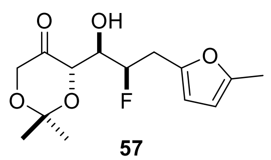

57

Data for *syn*-fluorohydrin **57**: **[$\alpha$]$_D^{20}$** = 16.4 (*c* 2.5 in $CHCl_3$); **$^1$H NMR** (600 MHz, $CDCl_3$): δ 6.02 (d, *J* = 3.1 Hz, 1H), 5.88 (d, *J* = 3.1 Hz, 1H), 4.95 (ddd, *J* = 46.8, 6.9, 6.9 Hz, 1H), 4.41 (d, *J* = 9.0 Hz, 1H), 4.29 (d, *J* = 17.6 Hz, 1H), 4.08 (d, *J* = 17.6 Hz, 1H), 3.82 (dd, *J* = 27.4, 8.8 Hz, 1H), 3.34 (d, *J* = 1.5 Hz, 1H), 3.18 (m, 1H), 3.06 (m, 1H), 2.26 (s, 3H), 1.49 (s, 3H), 1.42 (s, 3H); **$^{13}$C NMR** (150 MHz, $CDCl_3$): δ 212.4, 151.4, 149.0 (d, *J* = 9.5 Hz), 108.3, 106.4, 101.7, 89.4 (d, *J* = 179.3 Hz) , 71.4 (d, *J* = 5.1 Hz), 70.4 (d, *J* = 17.7 Hz), 66.6, 29.5 (d, *J* = 25.2 Hz), 23.6, 23.6, 13.7; **$^{19}$F NMR** (470 MHz, $CDCl_3$): δ –201.2

HRMS ($EI^+$) calcd for $[C_{14}H_{20}FO_5]^+$ 287.1289; found 287.1289

*Determination of enantiomeric excess of fluorohydrin* **57**

Following General Procedure B, using a 1:1 mixture of L-:D- proline, a racemic sample of the fluorohydrin **57** was prepared. Following General Procedure H, optically enriched and racemic samples of **57** (0.043 g, 0.15 mmol) were converted into the corresponding bis-*p*-nitrobenzoate derivative. The enantiomeric fluorohydrin were separated by chiral HPLC using a DIACEL CHIRALCEL-OD column; flow rate 1.5 mL/min; eluent: hexanes-*i*PrOH 93.5:6.5; detection at 260 nm; retention time = 30.00 min and 40.48 min (see chromatograms). The enantiomeric excess of the optically pure bis-*p*-nitrobenzoate derivative was determined using the same method (95% ee).

**Preparation of aldol adduct 58**

A solution of 3-OTIPS-propanal (1.152 g, 5.0 mmol, 1.5 equiv.), Selectfluor (1.77 g, 5.0 mmol, 1.5 equiv.), and L-proline (0.576 g, 5.0 mmol, 1.5 equiv.) were dissolved in 50 mL DMF (0.1 M) and stirred at 4 °C for 3 hrs or until the reaction was complete as determined by TLC analysis. The reaction mixture was diluted with 500 mL of diethyl ether and washed 3 x $H_2O$ (100 mL). The organic layer was removed, dried over $MgSO_4$, and concentrated under reduced pressure. Dioxanone **13** (0.434 g, 3.33 mmol, 1.0 equiv.) and L-proline (0.306 g, 2.7 mmol, 0.8 equiv.) were added to the crude α-fluoroaldehyde in 25 mL of $CH_2Cl_2$ (0.2 M). After 48 hours, the reaction mixture was diluted with $CH_2Cl_2$ and washed with $H_2O$. The ratio of diastereomers was determined to be >20:1 by $^1$H NMR spectroscopic analysis of the crude product. Purification by flash chromatography (pentane-EtOAc - 20:1) afforded *syn*-fluorohydrin **58** (0.692 g, 55 % yield) as a colorless oil**.**

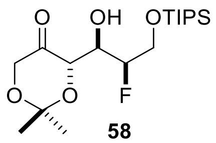

58

Data for *syn*-fluorohydrin **58**: **[$\alpha$]$_D^{20}$** = -25.6 (*c* 5.0 in $CHCl_3$); **IR** (neat): $\upsilon$ = 3503, 2994, 2867, 1741, 1224, 1094, 883 $cm^{-1}$; **$^1$H NMR** (600 MHz, $CDCl_3$): δ 4.75 (dddd, *J* = 47.1, 5.7, 5.7, 1.9 Hz, 1H), 4.41 (dd, *J* = 8.5, 1.4 Hz, 1H), 4.31 (dd, *J* = 17.5, 1.4 Hz, 1H), 4.08 (d, *J* = 17.6 Hz, 1H), 4.05 (ddd, *J* = 27.4, 9.4, 1.7 Hz, 2H), 4.02 (dd, *J* = 18.5, 5.5 Hz, 1H), 3.33 (br s, 1H), 1.50 (s, 3H), 1.44 (s, 3H), 1.07 (m, 21H); **$^{13}$C NMR** (150 MHz, $CDCl_3$): δ 211.7, 101.6, 91.0 (d, *J* = 178.1 Hz) , 71.7 (d, *J* = 5.2 Hz), 69.6 (d, *J* = 18.2 Hz), 66.8, 62.4 (d, *J* = 27.4 Hz), 23.7, 23.7, 18.1, 12.1; **$^{19}$F NMR** (470 MHz, $CDCl_3$): δ –209.0

HRMS ($EI^+$) calcd for $[C_{18}H_{36}FO_5Si]^+$ 379.2311; found 379.2343

*Determination of the absolute stereochemistry for fluorohydrin* **58**

Following General Procedure H, the fluorohydrin **58** (0.050 g, 0.13 mmol) was converted into the corresponding bis-*p*-bromobenzoate derivative **58-XRD**. Recyrstallization in dichloromethane and ethanol (1:1) allowed for the absolute stereochemistry to be assigned using single X-ray crystallography (see X-ray structures).

*Determination of enantiomeric excess of fluorohydrin* **58**

Following General Procedure B, using a 1:1 mixture of L-:D- proline, a racemic sample of the fluorohydrin **58** was prepared. Following General Procedure H, optically enriched and racemic samples of **58** (0.050 g, 0.13 mmol) were converted into the corresponding bis-*p*-nitrobenzoate derivative. The enantiomeric p-nitrobenzoyl diesters were separated by chiral HPLC using a DIACEL CHIRALCEL-OD column; flow rate 1.0 mL/min; eluent: hexanes-*i*PrOH 80:20; detection at 260 nm; retention time = 6.26 min and 8.33 min (see chromatograms). The enantiomeric excess of the optically enriched bis-*p*-nitrobenzoate derivative was determined using the same method (99 % ee).

**Preparation of chlorohydrin 59**

Following General Procedure A, valeraldehyde (0.054 mL, 0.50 mmol) was added to a mixture of NCS (71 mg, 0.53 mmol) and L-proline (46 mg, 0.40 mmol) in $CH_2Cl_2$ (0.9 mL) at 0°C and the resulting reaction mixture was stirred for 1 hr. O-TBS-hydroxyacetone (282 mg, 1.5 mmol) in DMSO (0.1 mL) and $H_2O$ (10 µL) were then added and the resulting reaction mixture was stirred for 24 hrs at room temperature. The ratio of diastereomers was determined to be >20:1 by $^1$H

NMR spectroscopic analysis of the crude product. Purification by flash chromatography (5-10-15% EtOAc in hexanes) afforded *syn*-chlorohydrin **59** (0.044 g, 27 % yield) as a pale yellow oil**.**

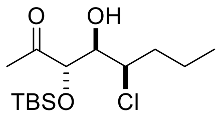

**59**

Data for *syn*-chlorohydrin **59**: **[$\alpha$]$_D^{20}$** = - 0.8 (*c* = 1.3 in $CHCl_3$)**; IR** (neat): $\upsilon$ = 3446, 2958, 2931, 1716, 1254, 1099, 837, 778, 672 $cm^{-1}$; **$^1$H NMR** (400 MHz, CDCl3) δ 4.28 (ddd, *J* = 9.2, 5.2, 1.8 Hz, 1H), 3.97 (d, *J* = 8.5 Hz, 1H), 3.67 (ddd, *J* = 10.3, 8.5, 1.7 Hz, 1H), 2.23 (s, 3H), 2.08 (d, *J* = 10.5 Hz, 1H), 1.92 (m, J = 4.6, 9.3, 14.3 Hz, 1H), 1.78 (dddd, *J* = 14.0, 8.9, 6.7, 5.3 Hz, 1H), 1.54 (m, 2H), 0.96 (dd, *J* = 7.4, 7.4 Hz, 3H), 0.91 (s, 9H), 0.12 (s, 3H), 0.05 (s, 3H) **$^{13}$C NMR** (100 MHz, CDCl3) δ 209.5, 79.1, 74.4, 63.8, 37.0, 25.6, 25.4, 19.9, 18.0, 13.2, -4.8, -5.1

HRMS: (ESI) m/z calcd for $C_{14}H_{33}{}^{35}ClO_3NSi$ $[M+NH_4]^+$ 326.1913, found 326.1889

*Determination of enantiomeric excess of chlorohydrin **59***

Following General Procedure H, PNB esters were prepared of both the racemic and enantioenriched samples. The enantiomeric PNB esters were separated by chiral HPLC using a Phenomex Lux i-Cellulose-5 column; flow rate 0.20 mL/min; eluent: hexanes-*i*PrOH 97:3; detection at 254 nm; retention time = 2.61 min and 3.30 min (see chromatograms).The enantiomeric excess of the optically enriched **59** was determined using the same method (97% ee).

**Preparation of chlorohydrin 60**

Following General Procedure A, 4-pentenal (0.042 mg, 0.50 mmol) was added to a mixture of NCS (71 mg, 0.53 mmol) and L-proline (46 mg, 0.40 mmol) in $CH_2Cl_2$ (0.9 mL) at 0°C and the resulting reaction mixture was stirred for 1 hr. O-TBS-hydroxyacetone (282 mg, 1.5 mmol) in DMSO (0.1 mL) and $H_2O$ (10 μL) were then added and the resulting reaction mixture was stirred for 24 hrs at room temperature. The ratio of diastereomers was determined to be 3:1 by $^1$H NMR spectroscopic analysis of the crude product. Purification by flash chromatography (pentane-EtOAc - 8:2) afforded *syn*-chlorohydrin **60** (0.051 g, 33 % yield) as a clear oil.

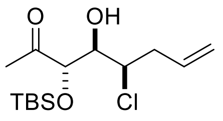

**60**

Data for *syn*-chlorohydrin **60**: **[$\alpha$]$_D^{20}$** = - 27.7 (*c* = 1.84 in $CHCl_3$); **IR** (neat): $\upsilon$ = 3443, 2955, 2929, 2858, 1716, 1641, 1255, 1097, 837, 778 $cm^{-1}$; **$^1$H NMR** (400 MHz, $CDCl_3$): δ 5.83 (dd, *J* = 10.2, 6.9, Hz, 1H), 5.20 (m, 2H), 4.26 (dd, *J* = 7.2, 1.9 Hz, 1H), 3.97 (d, *J* = 8.5 Hz, 1H), 3.73 (m, *J* = 10.3,

8.5 1.9 Hz, 1H), 2.65 (m, 2H), 2.23 (s, 3H), 2.09 (d, *J* = 10.3 Hz, 1H), 0.92 (s, 9H), 0.12 (s, 3H), 0.05 (s, 3H); **$^{13}$C NMR** (100 MHz, $CDCl_3$): δ 209.4, 133.6, 118.5, 78.9, 73.8, 62.8, 39.3, 25.6, 25.4, 18.0, -4.9, -5.1

HRMS (ESI) m/z calcd for $C_{14}H_{28}{}^{35}ClO_3Si$ $[M+H]^+$ 307.1491, found 307.1504

*Determination of enantiomeric excess of chlorohydrin **60***

Following General Procedure H, PNB esters were prepared of both the racemic and enantioenriched samples. The enantiomeric PNB esters were separated by chiral HPLC using a Phenomex Lux i-Cellulose-5 column; flow rate 0.40 mL/min; eluent: hexanes-*i*PrOH 95:5; detection at 254 nm; retention time = 1.18 min and 1.50 min (see chromatograms).The enantiomeric excess of the optically enriched **60** was determined using the same method (95% ee).

**Preparation of aldol adduct 61**

Following General Procedure A, 4-phenylbutanal (0.074 mg, 0.50 mmol) was added to a mixture of NCS (71 mg, 0.53 mmol) and L-proline (46 mg, 0.40 mmol) in $CH_2Cl_2$ (0.9 mL) at 0°C and the resulting reaction mixture was stirred for 1 hr. O-TBS-hydroxyacetone (282 mg, 1.5 mmol) in DMSO (0.1 mL) and $H_2O$ (10 µL) were then added and the resulting reaction mixture was stirred for 24 hrs at room temperature. The ratio of diastereomers was determined to be 4:1 by $^1$H NMR spectroscopic analysis of the crude product. Purification by flash chromatography (pentane-EtOAc - 9:1) afforded *syn*-chlorohydrin **61** (0.108 g, 56 % yield) as a white solid.

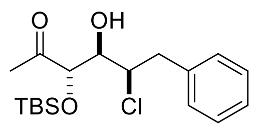


**61**

Data for *syn*-chlorohydrin **61**: $[\alpha]_D^{20}$ = - 9.7 (*c* = 2.0 in $CHCl_3$); **IR** (neat): $\upsilon$ = : 3362, 2958, 2927, 2856, 1708, 1104, 1074, 834, 698 cm$^{-1}$; **$^1$H NMR** (500 MHz, $CDCl_3$): δ 7.28 (m, 2H), 7.21 (m, 3H), 4.15 (ddd, *J* = 10.2, 4.0, 1.6, Hz, 1H), 3.94 (d, *J* = 8.6 Hz , 1H), 3.63 (ddd, J =10.4, 8.5, 1.6 Hz, 1H), 2.91 (ddd, *J* = 12.9, 7.5, 5.0, Hz, 1H), 2.78 (ddd, *J* = 13.9, 8.6, 7.3, Hz, 1H), 2.29 (dddd, J = 14.3, 10.3, 7.4, 5.0 Hz, 1H), 2.20 (s, 3H), 2.13 (d, *J* = 11.0 Hz, 1H), 2.07 (m, 1H), 0.80 (s, 9H), 0.00 (s, 6H); **$^{13}$C NMR** (125 MHz, $CDCl_3$): δ 209.4, 140.3, 128.6, 128.6, 126.3, 79.0, 75.0, 63.1, 36.5, 32.5, 25.6, 25.3, 17.9, -4.9, -5.3

HRMS (ESI) m/z calcd for $C_{19}H_{25}{}^{35}ClO_3NSi$ $[M+NH_4]^+$ 388.2069, found 388.2041

m.p.: 70-73°C

*Determination of relative stereochemistry for chlorohydrin* **61**

Following General Procedure G, the chlorohydrin **61** was converted to THF **81**. NOE analysis of THF **81** confirmed relative stereochemistry of chlorohydrin **61**.

*Determination of enantiomeric excess of chlorohydrin **61***

Following General Procedure H, PNB esters were prepared of both the racemic and enantioenriched samples. The enantiomeric PNB esters were separated by chiral HPLC using a Phenomex Lux Amylose-5 column; flow rate 0.40 mL/min; eluent: hexanes-*i*PrOH 95:5; detection at 254 nm; retention time = 3.24 min and 3.71 min (see chromatograms).The enantiomeric excess of the optically enriched **61** was determined using the same method (97% ee).

**Preparation of chlorohydrin 62**

Following General Procedure A, 4-pentynal (41 mg, 0.5 mmol) was added to a mixture of NCS (71 mg, 0.53 mmol) and L-proline (46 mg, 0.40 mmol) in $CH_2Cl_2$ (0.9 mL) at 0°C and the resulting reaction mixture was stirred for 1 hr. O-TBS-hydroxyacetone (282 mg, 1.5 mmol) in DMSO (0.1 mL) and $H_2O$ (10 μL) were then added and the resulting reaction mixture was stirred for 24 hrs at room temperature. The ratio of diastereomers was determined to be 4:1 by $^1$H NMR spectroscopic analysis of the crude product. Purification by flash chromatography (pentane-EtOAc; 7:3) afforded *syn*-chlorohydrin **62** (0.075 g, 49 % yield) as a clear oil.

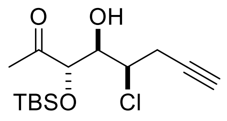

**62**

Data of *syn*-chlorohydrin **62**: $[\alpha]_D^{20}$ **=** - 34.3 (*c* = 0.80 in $CHCl_3$); ); **IR** (neat): $\upsilon$ = 3450, 3311, 2954, 2929, 1716, 1255, 1101, 837, 778, 635, 505 $cm^{-1}$; **$^1$H NMR** (500 MHz, $CDCl_3$): δ 4.30 (m, 1H), 3.97 (br s, 2H), 2.83 (m, 2H), 2.25 (s, 3H), 2.18 (s, 1H), 2.14 (d, *J* = 2.7 Hz, 1H), 0.93 (s, 9H), 0.13 (s, 3H), 0.06 (s, 3H); **$^{13}$C NMR** (125 MHz, $CDCl_3$): δ 209.7, 79.4, 78.7, 73.1, 71.5, 60.4, 25.6, 25.5, 25.1, 18.0, -4.8, -5.1

HRMS: (ESI) m/z calcd for $C_{14}H_{29}{}^{35}ClO_3NSi$ $[M+NH_4]^+$ 322.1600, found 322.1604

*Determination of enantiomeric excess of chlorohydrin **62***

Following General Procedure H, PNB esters were prepared of both the racemic and enantioenriched samples. The enantiomeric PNB esters were separated by chiral HPLC using a

Phenomex Lux i-Cellulose-5 column; flow rate 0.40 mL/min; eluent: hexanes-*i*PrOH 99:1; detection at 254 nm; retention time = 3.06 min and 3.79 min (see chromatograms).The enantiomeric excess of the optically enriched **62** was determined using the same method (97% ee).

**Preparation of aldol adduct 63 and hydrazone 77**

A solution of isovaleraldehyde (54 μL, 0.55 mmol, 1.1 equiv.), dibenzyl azodicarboxylate (0.149 g, 0.50 mmol, 1.0 equiv.), and L-proline (0.046 g, 0.40 mmol, 0.80 equiv.) in nitromethane (1.2 mL) was stirred until complete consumption of dibenzyl azodicarboxylate was observed by TLC analysis. **13** (0.130 g, 1.0 mmol, 2 equiv.) was then added and the reaction mixture was stirred for 48 hours. The reaction mixture was then diluted with $CH_2Cl_2$, washed with water and brine, dried over MgSO4, filtered, and concentrated under reduced pressure. The crude aldol adduct **63**, without further purification, was dissolved in MeOH (4 mL) containing 1% v/v AcOH. To this solution, was added Pd/C (100 mg, 25% by weight). H2 gas was bubbled into the reaction mixture until complete consumption of **63** was observed by TLC analysis. The reaction mixture was then filtered through celite and concentrated under reduced pressure. The ratio of diastereomers was determined to be 3:1 by $^1$H NMR spectroscopic analysis of the crude product. Purification by flash chromatography (pentane:EtOAc:$NEt_3$ – 60:39:1) afforded hydrazone **77** (0.146 g, 64 % yield over 2 steps) as a white solid.

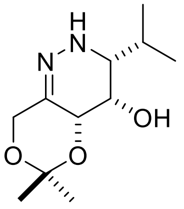

**77**

Data for hydrazone **77**: **$[\alpha]_D^{20}$ =** -22.9 (*c* = 3.19 in $CHCl_3$); ); **IR** (neat): $\upsilon$ = 3359, 2961, 2872, 1643, 1380, 1372, 1165, 1074, 862 cm$^{-1}$; **$^1$H NMR** (400 MHz, $CDCl_3$) δ 5.33 (br s, 1H), 4.41 (m, 1H), 4.39 (d, *J* = 15.6 Hz, 1H), 4.22 (d, *J* = 15.6 Hz, 1H), 4.03 (m, 1H), 2.71 (d, *J* = 9.4 Hz, 1H), 2.47 (br s, 1H), 1.99 (m, 1H), 1.52 (s, 3H), 1.43 (s, 3H), 1.00 (d, *J* = 6.8 Hz, 6H); **$^{13}$C NMR** (100 MHz, $CDCl_3$): δ 139.4, 99.78, 66.4, 62.77, 62.14, 61.8, 27.4, 27.2, 21.6, 19.5, 19.0

HRMS (ESI) m/z calcd for $C_{11}H_{21}N_2O_3$ $[M+H]^+$ 229.1547, found 229.1534

*Determination of relative stereochemistry for* ***63/77***

NOE analysis of hydrazone **77** confirmed relative stereochemistry of **63**.

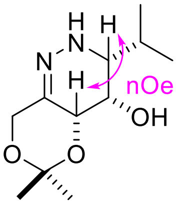


**77**

**Preparation of aldol adduct 64**

Following General Procedure C, a solution of pentanal (0.050 mL, 0.47 mmol), NFSI (0.148 g, 0.47 mmol), L-proline (0.054 g, 0.47 mmol) and $NaHCO_3$ (0.039 g, 0.47 mmol) was stirred at -10 °C in DMF (0.63 mL) for 75 minutes. Cyclohexanone (0.488 mL, 4.70 mmol) was added and the reaction mixture was stirred for 18 hours. The ratio of diastereomers was determined to be 10:1 by $^{1}$H NMR spectroscopic analysis of the crude product. Purification by flash chromatography (pentane:EtOAc – 95:5 → 90:10) afforded *syn*-fluorohydrin **64** (0.051 g, 54% yield) as an off-white solid**.**

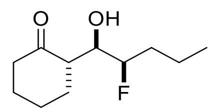
**64**

Data for *syn*-fluorohydrin **64**: $[\alpha]_D^{20}$ = -16.4 (*c* 1.65 in $CH_2Cl_2$); **IR** (neat): $\upsilon$ = 3498, 2957, 2938, 2863, 1698, 1450, cm$^{-1}$; **$^{1}$H NMR** (600 MHz, $CDCl_3$): δ 4.55 (ddd, *J* = 47.8, 8.3, 4.2 Hz, 1H), 3.77 (dddd, *J* = 29.0, 8.3, 4.2, 2.1 Hz, 1H), 3.55 (d, *J* = 4.2 Hz, 1H), 2.45 (m, 1H), 2.36 (dddd, *J* = 13.4, 13.4, 6.2, 1.1 Hz, 1H), 2.23 (m, 1H), 2.12 (m, 1H), 1.85 – 1.97 (2H), 1.55 – 1.78 (3H), 1.36 – 1.55 (3H), 0.96 (dd, *J* = 7.4, 7.4 Hz, 3H); **$^{13}$C NMR** (150 MHz, $CDCl_3$): δ 215.7, 92.7 (d, *J* = 174.1 Hz), 72.2 (d, *J* = 19.3 Hz), 52.8 (d, *J* = 3.5 Hz), 42.9, 32.8 (d, *J* = 21.2), 30.1, 27.8, 24.8; **$^{19}$F NMR** (470 MHz, $CDCl_3$): δ –199.3

HRMS (EI$^{+}$) calcd for $[C_{11}H_{20}FO_2]^{+}$ 203.1442; found 203.1421

*Determination of enantiomeric excess of fluorohydrin* **64**

Following General Procedure H, the optically enriched and racemic samples of fluorohydrin **64** were converted into their corresponding p-nitrobenzoyl diesters. The enantiomeric *p*-nitrobenzoyl diesters were separated by chiral HPLC using a Lux® 3µm Amylose-1 column; flow rate 0.40 mL/min; eluent: hexanes-*i*PrOH 90:10; detection at 254 nm; retention times = 8.96 min and 10.37 min (see chromatograms). The enantiomeric excess of the optically enriched p-nitrobenzoyl diesters was determined using the same method (94 % ee).

**Preparation of aldol adduct 65**

Following General Procedure C, a solution of pentanal (0.0.50 mL, 0.47 mmol), NFSI (0.148 g, 0.47 mmol), L-proline (0.054 g, 0.47 mmol) and $NaHCO_3$ (0.039 g, 0.47 mmol) was stirred for 75 minutes at -10 °C in DMF (0.63 mL). Thiopyranone **35** (0.546 g, 4.70 mmol) was added and the

reaction mixture was stirred for 24 hours at 4°C. The ratio of diastereomers was determined to be 10:1 by $^1$H NMR spectroscopic analysis of the crude product. Purification by flash chromatography (pentane-$Et_2O$ - 4:1) afforded *syn*-fluorohydrin **65** (0.049 g, 47% yield) as a white solid.

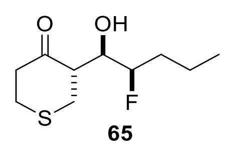

**65**

Data for *syn*-fluorohydrin **65**: **$[\alpha]_D^{20}$** = -16.4° (*c* 1.65 in $CH_2Cl_2$); **IR** (neat): $\upsilon$ = 3353, 2952, 1706, 1428, 510 cm$^{-1}$; **$^1$H NMR** (600 MHz, $CDCl_3$): δ 4.58 (ddd, *J* = 48.0, 8.8, 2.3 Hz, 1H), 3.95 (ddd, *J* = 26.7, 6.6, 2.3 Hz, 1H), 3.07 (m, 1H), 3.06 (m, 1H), 2.94 – 3.02 (3H), 2.72 – 2.86 (3H), 1.88 (m, 1H), 1.64 (m, 1H), 1.60 (m, 1H), 1.51 (m, 1H), 1.42 (m 1H), 0.97 (dd, *J* = 7.4, 7.4 Hz, 3H);**$^{13}$C NMR** (150 MHz, $CDCl_3$): δ 211.7, 92.8 (d, *J* = 173.6 Hz), 71.8 (d, *J* = 20.8 Hz), 55.2 (d, *J* = 3.1 Hz), 44.7, 32.9 (d, *J* = 20.9 Hz), 32.5 (d, *J* = 1.2 Hz), 30.9, 18.7 (d, *J* = 4.9 Hz), 14.0; **$^{19}$F NMR** (470 MHz, $CDCl_3$): δ –197.6

HRMS ($EI^+$) calcd for $[C_{10}H_{18}FO_2S]^+$ 221.1006; found 221.0999

*Determination of enantiomeric excess of fluorohydrin **65***

Using a 1:1 mixture of L-: D-proline, a racemic sample of fluorohydrin **65** was prepared. The enantiomeric fluorohydrins were separated by chiral HPLC using a Lux® 3µm Amylose-1 column; flow rate 0.40 mL/min; eluent: hexanes-*i*PrOH 90:10; detection at 254 nm; retention time = 7.14 min and 8.89 min (see chromatograms). The enantiomeric excess of the optically enriched fluorohydrin **65** was determined using the same method (84% ee).

**Preparation of aldol adduct 66**

Following General Procedure **C**, a solution of pentenal (0.050 g, 0.595 mmol), NFSI (0.188 g, 0.595 mmol), L-proline (0.069 g, 0.595 mmol) and $NaHCO_3$ (0.050 g, 0.595 mmol) was stirred for 1.5 hrs at -10°C in DMF (0.79 mL). Cyclohexanone (0.62 mL, 5.95 mmol) was added and the reaction mixture stirred for 16 hours. The ratio of diastereomers was determined to be 7:1 by $^1$H NMR spectroscopic analysis of the crude product. Purification by flash chromatography (pentane:EtOAc – 95:5 → 90:10) afforded *syn*-fluorohydrin **66** (0.059 g, 50% yield) as a white solid.

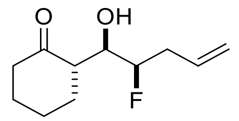
66

Data for *syn*-fluorohydrin **66**: **[α]$_D^{20}$** = +22.2 (*c* 0.60 in $CH_2Cl_2$); **IR** (neat): υ = 3513, 2937, 2863, 1698, 1449, 1132 cm$^{-1}$; **$^1$H NMR** (600 MHz, $CDCl_3$): δ 5.83 (dddd, *J* = 17.2, 10.3, 7.2, 7.0 Hz, 1H), 5.19 (dd, *J* = 17.2, 1.5 Hz, 1H), 5.13 (d, *J* = 10.3 Hz, 1H), 4.57 (ddd, *J* = 47.7, 8.3, 4.7 Hz, 1H), 3.80 (dddd, *J* = 29.7, 8.3, 4.1, 2.0 Hz, 1H), 3.58 (d, *J* = 4.1 Hz, 1H), 2.74 (m, 1H), 2.67 (m, 1H), 2.42 – 2.55 (2H), 2.36 (dddd, *J* = 13.4, 13.4, 6.1, 1.1 Hz, 1H), 2.22 (m, 1H), 2.12 (m, 1H), 1.93 (m, 1H), 1.73 (ddddd, *J* = 13.0, 13.0, 13.0, 3.6, 3.6 Hz, 1H), 1.67 (ddddd, *J* = 13.0, 13.0, 13.0, 3.6, 3.6 Hz, 1H), 1.42 (dddd, *J* = 12.7, 12.7, 12.7, 3.6, 3.6 Hz, 1H); **$^{13}$C NMR** (150 MHz, $CDCl_3$): δ 215.6, 133.3 (d, *J* = 7.5 Hz), 118.4, 92.0 (d, *J* = 177.6 Hz), 71.6 (d, *J* = 18.8 Hz), 52.7 (d, *J* = 3.6 Hz), 42.8, 35.4 (d, *J* = 23.4 Hz), 30.0, 27.8 24.8; **$^{19}$F NMR** (470 MHz, $CDCl_3$): δ –198.7

HRMS (EI$^+$) calcd for $[C_{11}H_{18}FO_2]^+$ 201.1285; found 201.1260

*Determination of relative stereochemistry for fluorohydrin* **66**

Following General Procedure E, the fluorohydrin **66** was converted to carbacycle **91**. NOE analysis of carbacycle **91** confirmed relative stereochemistry of fluorohydrin **66**.

**Preparation of aldol adduct 67**

Following General Procedure C, a solution of pentenal (0.100 mL, 1.02 mmol), NFSI (0.319 g, 1.02 mmol), L-proline (0.118 g, 1.02 mmol) and $NaHCO_3$ (0.086 g, 1.02 mmol) was stirred for 60 minutes at -10 °C in DMF (1.35 mL). Thiopyranone **35** (1.19 g, 10.2 mmol) was then added and the reaction mixture was stirred for 24 hrs at 4°C. The ratio of diastereomers was determined to be 8:1 by $^1$H NMR spectroscopic analysis of the crude product. Purification by flash chromatography (pentane:$Et_2O$ – 4:1) afforded *syn*-fluorohydrin **67** (0.069 g, 31 % yield) as a waxy white solid.

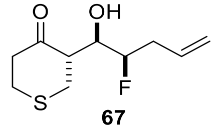
67

Data for *syn*-fluorohydrin **67**: **[α]$_D^{20}$** = -11.0 (*c* 0.30 in $CH_2Cl_2$); **IR** (neat): υ = 3455, 2929, 1703, 1428, 1117, 923 cm$^{-1}$;**$^1$H NMR** (600 MHz, $CDCl_3$): δ 5.82 (dddd, *J* = 17.1, 10.3, 7.0, 6.9 Hz, 1H), 5.21 (dd, *J* = 17.1, 1.4 Hz, 1H), 5.15 (d, *J* = 10.3, 1H), 4.61 (ddd, *J* = 47.7, 5.9, 2.0 Hz, 1H), 3.97 (ddd, *J* = 27.5, 6.4, 2.0 Hz, 1H), 3.09 (m, 1H), 3.05 (m, 1H), 3.04 (m, 1H), 2.99 (m, 1H), 2.97 (m, 1H), 2.81 (m, 2H), 2.76 (m, 1H), 2.64 (m, 1H), 2.51 (m, 1H) ; **$^{13}$C NMR** (150 MHz, $CDCl_3$): δ 211.7,

132.8 (d, $J$ = 7.3 Hz), 118.7, 92.1 (d, $J$ = 176.3 Hz), 71.2 (d, $J$ = 19.3 Hz), 55.1 (d, $J$ = 3.0 Hz), 44.7, 35.4 (d, $J$ = 22.2 Hz), 32.4, 30.8; **$^{19}$F NMR** (470 MHz, $CDCl_3$): δ -197.0

HRMS ($EI^+$) calcd for $[C_{10}H_{16}FO_2S]^+$ 219.0850; 219.0833

*Determination of relative stereochemistry for fluorohydrin* **67**

Following General Procedure E, the fluorohydrin **67** was converted to carbacycle **92**. NOE analysis of carbacycle **92** confirmed relative stereochemistry of fluorohydrin **67**.

**Preparation of syn-fluorohydrin 68a and anti-fluorohydrin 68b**

Following General Procedure C, a solution of aldehyde (2.00 g, 5.86 mmol, 1.0 equiv.), NFSI (1.85 g, 5.86 mmol, 1.0 equiv.), L-proline (0.674 g, 5.86 mmol, 1.0 equiv.) and $NaHCO_3$ (0.984 g, 11.71 mmol, 2 equiv.) was stirred at rt in DMF (10 mL) for 2 hrs. Cyclohexanone (1.15 g, 11.71 mmol) was added and the reaction mixture was stirred for 18 hours. The reaction mixture was then diluted with ethyl acetate (100 mL) and water (30 mL). The organic layer was washed with brine (2 x 30 mL), dried over $MgSO_4$, filtered, and concentrated under reduced pressure. Purification of crude fluorohydrins **68** by flash chromatography (25-75% ethyl acetate in hexanes) afforded *syn*-fluorohydrin **68a** (0.92 g, 36 % yield) and *anti*-fluorohydrin **68b** (1.21 g, 47% yield) as white solids**.**

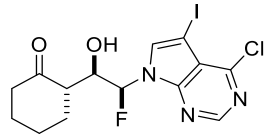


**68a**

Data for *syn*-fluorohydrin **68a**: **$^1$H NMR** (500 MHz, $CDCl_3$): δ 8.73 (s, 1H), 8.27 (s, 1H), 7.02 (dd, $J$ = 50.0, 5.6 Hz, 1H), 5.82 (d, $J$ = 6.9 Hz, 1H), 4.47 (m, 1H), 2.43 (m, 1H), 2.24 (m, 1H), 2.16 (m, 1H), 2.05 (m, 1H), 1.80 – 1.86 (m, 2H), 1.73 (m, 1H), 1.55 – 1.60 (m, 2H); **$^{13}$C NMR** (125 MHz, $CDCl_3$): δ 209.9, 151.5, 151.3, 151.0, 134.0, 116.6, 92.5 (d, $J$ = 205.2 Hz), 69.7 (d, $J$ = 24.4 Hz), 55.3, 51.5, 51.5, 41.5, 29.2, 26.3, 23.5; **$^{19}$F NMR** (470 MHz, $CDCl_3$): δ –147.6

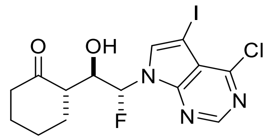


**68b**

Data for *anti*-fluorohydrin **68b**: **$^1$H NMR** (500 MHz, $CDCl_3$): δ 8.75 (s, 1H), 8.34 (s, 1H), 7.05 (dd, J = 47.6, 7.3 Hz, 1H), 5.59 (d, J = 6.7 Hz, 1H), 4.55 (m ,1H), 2.70 (m, 1H), 2.39 (m, 1H), 2.27 (m, 1H), 1.87 – 1.99 (m, 2H), 1.84 (m, 1H), 1.56 – 1.76 (m, 3H); **$^{13}$C NMR** (125 MHz, $CDCl_3$): 210.1, 151.6, 151.4, 151.3, 133.8, 116.6, 91.5 (d, J = 204.6 Hz), 68.9 (d, J = 30.5 Hz), 55.2, 51.1, 41.7, 29.1, 26.4, 23.5

*Determination of relative stereochemistry for syn-fluorohydrin* **68a**

Fluorohydrin **68a** was converted into nucleoside **86**. NOE analysis of nucleoside **86** confirmed relative stereochemistry of fluorohydrin **68a**.

*Determination of enantiomeric excess of fluorohydrin **68a***

Using a 1:1 mixture of L-: D-proline, a racemic sample of fluorohydrin **68a** was prepared. The enantiomeric fluorohydrins were separated by chiral SFC using Daicel OJ-3; 2900 PSI $CO_2$, 40 °C, 3 ml/min, gradient of 20-30% 25mM isobutylamine in isopropanol:CO2 over seven minutes; retention times = 2.57 min and 2.77 min (see chromatograms). The enantiomeric excess of the optically enriched fluorohydrin **68a** was determined using the same method (94% ee).

*Determination of enantiomeric excess of fluorohydrin **68b***

Using a 1:1 mixture of L-: D-proline, a racemic sample of fluorohydrin **68b** was prepared. The enantiomeric fluorohydrins were separated by chiral SFC using Daicel OJ-3; 2900 PSI $CO_2$, 40 °C, 3 ml/min, gradient of 1-20% 25mM diethylamine in methanol:$CO_2$ over five minutes; retention times = 3.10 min and 3.32 min (see chromatograms). The enantiomeric excess of the optically enriched fluorohydrin **68b** was determined using the same method (93% ee).

**Preparation of aldol adduct 69**

Following General Procedure C, a solution of phthalimidoacetaldehyde (0.050 g, 0.265 mmol), NFSI (0.84 g, 0.265 mmol), L-proline (0.031 g, 0.265 mmol) and 2,6-lutidine (0.031 mL, 0.265 mmol) was stirred at 4°C in DMF (0.35 mL) for 15 hrs. Thiopyranone **35** (0.307 g, 2.65 mmol) was added and the reaction mixture was stirred for 18 hours. The ratio of diastereomers was determined to be 5:1 by $^1$H NMR spectroscopic analysis of the crude product. Purification by flash chromatography (pentane:EtOAc – 60:40) afforded an inseparable mixture of *syn*- and *anti*-fluorohydrins **69** (0.075 g, 87% yield, d.r. = 5:1) as a white solid**.**

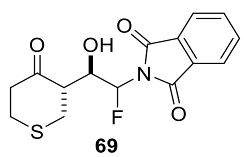

**69**

Data for fluorohydrin **69**: **$^1$H NMR** (600 MHz, $CDCl_3$): δ 7.93, 7.92, 7.79, 7.79, 6.26, 6.11, 5.37, 4.78, 3.44, 3.25, 3.24, 3.16, 3.11, 3.09, 3.03, 2.99, 2.98, 2.85, 2.80, 2.79; **$^{13}$C NMR** (150 MHz, $CDCl_3$): δ 212.8, 210.2, 167.1, 167.1, 135.1, 134.9, 131.6, 131.5, 124.3, 124.2, 89.6, 88.3, 70.1, 66.1, 54.6, 53.6, 45.7, 44.9, 34.6, 31.3, 30.7, 30.1; **$^{19}$F NMR** (470 MHz, $CDCl_3$): δ –155.5, –158.5

HRMS ($EI^+$) calcd for $[C_{15}H_{14}FNO_4S + NH_4]^+$ 341.0966; observed 341.0938

**Preparation of aldol adduct 70**

Following General Procedure C, a solution of phthalimidoacetaldehyde (0.050 g, 0.265 mmol), NFSI (0.84 g, 0.265 mmol), L-proline (0.031 g, 0.265 mmol) and 2,6-lutidine (0.031 mL, 0.265 mmol) was stirred at 4°C in DMF (0.35 mL) for 16 hrs. cyclohexanone (0.275 mL, 2.65 mmol) was added and the reaction mixture was stirred for 18 hours. The ratio of diastereomers was determined to be 5:1 by $^{1}H$ NMR spectroscopic analysis of the crude product. Purification by flash chromatography (pentane:EtOAc – 60:40) afforded an inseparable mixture of *syn*-and *anti* - fluorohydrin **70** (0.068 g, 84% yield, d.r. = 5:1) as a white solid**.**

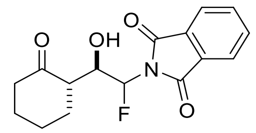

**70**

Data for fluorohydrin **70**: **$^{1}$H NMR** (600 MHz, $CDCl_3$): δ 7.92, 7.91, 7.78, 7.78, 6.29, 6.07, 5.37, 4.63, 3.51, 2.93, 2.92, 2.89, 2.80, 2.44, 2.41, 2.30, 2.25, 2.16, 2.01. 1.99, 1.87, 1.78, 1.71; **$^{13}$C NMR** (150 MHz, $CDCl_3$): δ 215.9, 213.5, 167.1, 167.1, 134.9, 134.8, 131.7, 131.6, 124.1, 124.1, 89.9, 88.3, 69.9, 65.5, 51.8, 51.0, 43.3, 42.7, 32.4, 28.3, 27.8, 26.1, 25.4, 24.8; **$^{19}$F NMR** (470 MHz, $CDCl_3$): δ –156.0, –160.7

HRMS ($EI^+$) calcd for $[C_{16}H_{17}FNO_4]^+$ 306.1136; observed 306.1135

**Preparation of aldol adduct 71**

Following General Procedure D, a solution of pentenal (0.100 mL, 1.01 mmol), $N(SCF_3)Phth$ (0.250 g, 1.01 mmol), L-proline (0.116 g, 1.01 mmol), and $NaHCO_3$ (0.085 g, 1.01 mmol) was stirred for 50 minutes at RT in DMSO (1.35 mL). Dioxanone **13** (0.061 mL, 0.506 mmol) in $CH_2Cl_2$ (6.7 mL) was added and the reaction mixture was stirred for 60 hrs. The ratio of diastereomers was determined to be 6:1 by $^{1}H$ NMR spectroscopic analysis of the crude product. Purification by flash chromatography (pentane-$Et_2O$ 4:1) afforded *syn*-trifluoromethylthiohydrin **71** (0.103 g, 65 % yield) as a light yellow oil**.**

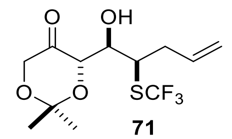

**71**

Data for *syn*-trifluoromethylthiohydrin **71**: **$[\alpha]_D^{20}$** = -100.5° (*c* 1.28 in $CH_2Cl_2$); **IR** (neat): $\upsilon$ = 3650, 3150, 1737, 1377, 1224, 1109 $cm^{-1}$;**$^{1}$H NMR** (600 MHz, $CDCl_3$): δ 5.79 (m, 1H), 5.18 (d, *J* = 17.0 Hz, 1H), 5.14 (d, *J* = 10.3 Hz, 1H), 4.42 (d, *J* = 9.0 Hz, 1H), 4.29 (d, *J* = 17.8 Hz, 1H), 4.12 (d, *J* = 9.0 Hz, 1H), 4.07 (d, *J* = 17.8 Hz, 1H), 3.63 (s, 1H), 3.51 (dd, *J* = 10.1, 4.8 Hz, 1H), 2.78 (ddd, *J* = 14.3, 9.9, 7.9 Hz, 1H), 2.68 (ddd, *J* = 14.3, 6.6, 5.1 Hz, 1H), 1.48 (s, 3H), 1.42 (s, 3H); **$^{13}$C**

**NMR** (150 MHz, $CDCl_3$): δ 213.3, 134.4, 131.6 (q, *J* = 303.5 Hz), 118.7, 101.8, 72.3, 70.4, 66.4, 46.3, 38.9, 23.8, 23.6; **$^{19}$F NMR** (470 MHz, $CDCl_3$): δ –39.7

HRMS ($EI^+$) calcd for $[C_{12}H_{17}F_3O_4S + NH_4]^+$ 332.1138; found 332.1110

*Determination of relative stereochemistry for trifluoromethylthiohydrin **71***

Following General Procedure E, the trifluoromethylthiohydrin **71** was converted to carbacycle **90**. NOE analysis of carbacycle **93** confirmed relative stereochemistry of trifluoromethylthiohydrin **71**.

*Determination of enantiomeric excess of trifluoromethylthiohydrin **71***

Following General Procedure I, trifluoromethylthiohydrin **71** was converted to trifluoromethylthiohydrin **30**. Following General Procedure H, optically enriched and racemic samples of trifluoromethylthiohydrin **30** were converted into their corresponding p-nitrobenzoyl diesters. The enantiomeric p-nitrobenzoyl diesters were separated by chiral HPLC using a Lux® 3µm Amylose-1 column; flow rate 0.50 mL/min; eluent: hexanes-*i*PrOH 92.5:7.5; detection at 254 nm; retention time = 6.92 min and 10.52 min (see chromatograms). The enantiomeric excess of the optically enriched p-nitrobenzoyl diester was determined using the same method (91% ee).

**Preparation of aldol adduct S1**

Following General Procedure D, a solution of isovaleraldehyde (0.050 mL, 0.456 mmol), $N(SCF_3)$Phth (0.113 g, 0.456 mmol), L-proline (0.053 g, 0.456 mmol), and $NaHCO_3$ (0.038 g, 0.456 mmol) was stirred for 50 minutes at RT in DMSO (0.61 mL). Dioxanone **13** (0.027 mL, 0.228 mmol) in $CH_2Cl_2$ (3.04 mL) was added and the reaction mixture was stirred for 60 hrs. The ratio of diastereomers was determined to be 10:1 by $^1$H NMR spectroscopic analysis of the crude product. Purification by flash chromatography (pentane:$Et_2O$ – 88:12) afforded *syn*-trifluoromethylthiohydrin **S1** (0.060 g, 42 % yield) as a colorless oil**.**

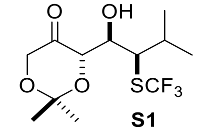

**S1**

Data for *syn*-trifluoromethylthiohydrin **S1**: **$[\alpha]_D^{20}$** = -106.0° (*c* 2.15 in $CH_2Cl_2$); **IR** (neat): $\upsilon$ = 3508, 2967, 173, 1101, 865 cm$^{-1}$;**$^1$H NMR** (600 MHz, $CDCl_3$): δ 4.41 (dd, *J* = 9.0, 1.3 Hz, 1H), 4.30 (dd, *J* = 17.6, 1.5 Hz, 1H), 4.19 (d, *J* = 9.0 Hz, 1H), 4.08 (d, *J* = 17.6 Hz, 1H), 3.73 (dd, *J* = 2.3, 1.3 Hz, 1H), 3.35 (d, *J* = 5.5 Hz, 1H), 2.18 (m, 1H), 1.47 (s, 3H), 1.43 (s, 3H), 1.10 (dd, *J* = 5.8, 5.7 Hz, 3H); **$^{13}$C NMR**

(150 MHz, $CDCl_3$): δ 213.6, 131.6 (q, $J$ = 305.0 Hz), 101.7, 72.2, 70.4, 66.5, 53.5, 33.1, 23.8, 23.7, 20.8, 19; **$^{19}$F NMR** (470 MHz, $CDCl_3$): δ –38.6

HRMS (EI$^+$) calcd for $[C_{12}H_{19}F_3O_4S + NH_4]^+$ 334.1294; found 334.1266

**Preparation of aldol adduct 72**

Following General Procedure D, a solution of 3-(4-methoxyphenyl)propanal (0.050 mL, 0.317 mmol), PhthN($SCF_3$) (0.078 g, 0.317 mmol), L-proline (0.037 g, 0.317 mmol), and $NaHCO_3$ (0.027 g, 0.317 mmol) was stirred for 50 minutes at RT in DMSO (0.42 mL). Dioxanone **13** (0.019 mL, 0.228 mmol) in $CH_2Cl_2$ (2.11 mL) was added and the reaction mixture stirred for 60 hrs. The ratio of diastereomers was determined to be 10:1 by $^1$H NMR spectroscopic analysis of the crude product. Purification by flash chromatography (pentane:$Et_2O$ – 80:20) afforded *syn*-trifluoromethylthiohydrin **72** (0.032 g, 56 % yield) as a yellow oil**.**

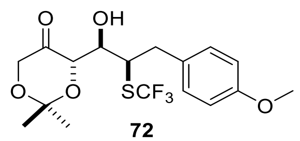

**72**

Data for *syn*-trifluoromethylthiohydrin **72**: **[$\alpha$]$_D^{20}$** = -61.4° ($c$ 1.6 in $CH_2Cl_2$); **IR** (neat): $\upsilon$ = 3518, 1736, 1512, 1108, 863 cm$^{-1}$;**$^1$H NMR** (600 MHz, $CDCl_3$): δ 7.15 (d, $J$ = 8.5 Hz, 2H), 6.85 (d, $J$ = 8.5 Hz, 2H), 4.40 (dd, $J$ = 9.1, 1.3 Hz, 1H), 4.20 (dd, $J$ = 17.6, 1.5 Hz, 1H), 4.01 (d, $J$ = 17.6 Hz, 1H), 3.89 (d, $J$ = 9.1 Hz, 1H), 3.80 (s, 3H), 3.58-3.62 (2H), 3.26 (dd, $J$ = 13.7, 11.0 Hz, 1H), 3.21 (dd, $J$ = 13.7, 5.5 Hz, 1H), 1.46 (s, 3H), 1.34 (s, 3H;**$^{13}$C NMR** (150 MHz, $CDCl_3$): δ 213.6, 158.6, 131.6 (q, $J$ = 306.0 Hz), 130.5, 130.0, 114.1, 101.7, 72.4, 69.2, 66.4, 55.4, 48.2, 39.2, 23.8, 23.6; **$^{19}$F NMR** (470 MHz, $CDCl_3$): δ –39.7

HRMS (EI$^+$) calcd for $[C_{17}H_{21}F_3O_5S + NH_4]^+$ 412.1400.1340; found 412.1369

*Determination of enantiomeric excess of trifluoromethylthiohydrin* **72**

Using sodium borohydride in methanol, optically enriched and racemic samples of trifluoromethylthiohydrin **72** were converted into their corresponding diols. The enantiomeric diols were separated by chiral HPLC using a Lux® 3µm Amylose-1 column; flow rate 0.50 mL/min; eluent: hexanes-*i*PrOH 95:5; detection at 254 nm; retention time = 6.01 min and 8.49 min (see chromatograms). The enantiomeric excess of the optically enriched diol was determined using the same method (93% ee).

**Preparation of aldol adduct 73**

Following General Procedure D, a solution of 3-(5-methylfuran-2-yl)propanal (0.050 mL, 0.376 mmol), N($SCF_3$)Phth (0.093 g, 0.376 mmol), L-proline (0.043 g, 0.376 mmol), and $NaHCO_3$ (0.032 g, 0.376 mmol) was stirred for 50 minutes at RT in DMSO (0.50 mL). Dioxanone **13** (0.023 mL, 0.188 mmol) in $CH_2Cl_2$ (2.50 mL) was added and the reaction mixture stirred for 60 hrs. The ratio of diastereomers was determined to be >10:1 by $^1$H NMR spectroscopic analysis of the crude product. Purification by flash chromatography (pentane:$Et_2O$ – 85:15) afforded *syn*-trifluoromethylthiohydrin **73** (0.032 g, 46 % yield) as a colorless oil**.**

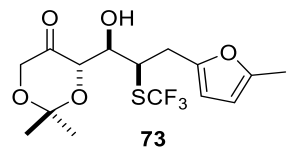

**73**

Data for *syn*-trifluoromethylthiohydrin **73**: **$[\alpha]_D^{20}$** = -101.7° (*c* 1.0 in $CH_2Cl_2$); **IR** (neat): $\upsilon$ = 3517, 2995, 1736, 1386, 1113, 737 cm$^{-1}$;$^1$**H NMR** (600 MHz, $CDCl_3$): δ 6.00 (d, *J* = 2.8 Hz, 1H), 5.86 (d, *J* = 2.8 Hz, 1H), 4.42 (dd, *J* = 9.0, 1.3 Hz, 1H), 4.26 (dd, *J* = 17.6, 1.3 Hz, 1H), 4.05 (d, *J* = 17.6 Hz, 1H), 4.03 (d, *J* = 9.0 Hz, 1H), 3.76 (dd, *J* = 10.6, 5.1 Hz, 1H), 3.62 (dd, *J* = 2.3, 1.3 Hz, 1H), 3.31 (dd, *J* = 15.1, 10.6 Hz, 1H), 3.18 (dd, *J* = 15.1, 5.1 Hz, 1H), 2.26 (s, 3H), 1.48 (s, 3H), 1.40 (s, 3H); $^{13}$**C NMR** (150 MHz, $CDCl_3$): δ 213.6, 151.6, 149.9, 131.4 (q, *J* = 306.0 Hz), 108.6, 106.2, 101.7, 72.4, 70.3, 66.4, 45.8, 33.2, 23.8, 23.6, 13; $^{19}$**F NMR** (470 MHz, $CDCl_3$): δ –39.8

HRMS (EI$^+$) calcd for $[C_{15}H_{19}F_3O_5S + NH_4]^+$ 386.1244; found 386.1227

*Determination of enantiomeric excess of trifluoromethylthiohydrin **73***

Following General Procedure H, optically enriched and racemic samples of trifluoromethylthiohydrin **73** were converted into their corresponding *p*-nitrobenzoyl diesters. The enantiomeric p-nitrobenzoyl diesters were separated by chiral HPLC using a Lux® 3µm Amylose-1 column; flow rate 0.50 mL/min; eluent: hexanes-*i*PrOH 95:5; detection at 254 nm; retention time = 20.83 min and 22.46 min (see chromatograms). The enantiomeric excess of the optically enriched p-nitrobenzoyl diester was determined using the same method (93% ee).

**Preparation of THF 78**

Following General Procedure G, to a stirred solution of chlorohydrin **38** (100 mg, 0.45 mmol) in MeOH (2 mL) was added $NaBH_4$ (74 mg, 2.0 mmol) in MeOH (2 mL). Once TLC analysis indicated complete consumption of **36**, the reaction mixture was quenched with aqueous $NH_4Cl$ (5 mL), extracted 3x with 10 mL of $CH_2Cl_2$, and concentrated under reduced pressure to afford diol **S38**. Without further purification, the crude reduction product dissolved in MeOH (2 mL) and

subjected to microwave irradiation. Purification of crude **78** by flash column chromatography (hexanes:EtOAc – 80:20) afforded **78** (60 mg, 74% yield) as a white solid.

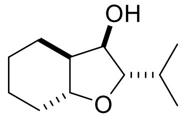

**78**

Data for THF **78**: **[$\alpha$]$_D^{20}$** = +4.7 (*c* = 4.2 in $CHCl_3$); **IR** (neat): $\upsilon$ = 3368, 2931, 2865, 1446, 1081, 1070, 978, 641 $cm^{-1}$; **$^1$H NMR** (600 MHz, $CDCl_3$) δ 3.97 (m, 1H), 3.47 (m, *J* = 6.5 Hz, 1H), 3.39 (m, *J* = 11.0, 4.9 Hz, 1H), 2.15 (m, 1H), 1.84-1.70 (m, 5H), 1.37-1.17 (m, 4H), 1.13 (m, 1H), 0.95 (d, *J* = 6.7 Hz, 3H), 0.92 (d, *J* = 6.7 Hz, 3H); **$^{13}$C NMR** (150 MHz, CDCl3) δ 93.1, 79.4, 76.6, 50.2, 31.5, 31.1, 25.5, 23.9, 23.3, 18.49, 18.47

HRMS: (ESI) m/z calcd for $C_{11}H_{21}O_2$ $[M+H]^+$ 185.1536, found 185.1541

***Determination of relative stereochemistry for THF 78***

Analysis of 2D NOESY of THF **78** supported the indicated stereochemistry.

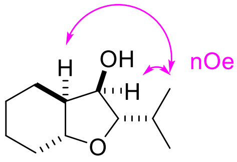


**78**

**Preparation of THF 79**

Following General Procedure G, to a stirred solution of chlorohydrin **36** (250 mg, 1.0 mmol) in MeOH (2 mL) was added $NaBH_4$ (74 mg, 2.0 mmol) in MeOH (2 mL). Once TLC analysis indicated complete consumption of **36**, the reaction mixture was quenched with aqueous $NH_4Cl$ (5 mL), extracted 3x with 10 mL of $CH_2Cl_2$, and concentrated under reduced pressure to afford diol **S36**. Without further purification, the crude reduction product dissolved in MeOH (2 mL) and subjected to microwave irradiation. Purification of crude **79** by flash column chromatography (pentane:EtOAc – 80:20) afforded **79** (30 mg, 15% yield) as a white solid.

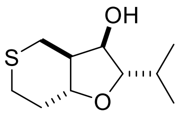

**79**

Data for THF **79**: **[$\alpha$]$_D^{20}$** = + 4.7 (*c* = 2.7 in $CHCl_3$); **IR** (neat): $\upsilon$ = 3398, 2924, 1437, 1382, 1072, 1015, 945, 880, 634 $cm^{-1}$; **$^1$H NMR** (600 MHz, $CDCl_3$) δ 4.06 (m, 1H), 3.43 (d, *J* = 6.5 Hz, 1H), 3.38 (ddd, *J* = 10.9, 10.9, 3.7 Hz, 1H), 2.84 (dd, *J* = 13.0, 11.6 Hz, 1H), 2.73-2.61 (m, 3H), 2.46 (dd, *J* = 11.1, 3.5 Hz, 1H), 1.74 (m, 1H), 1.66 (m, 1H), 1.60 (m, 1H), 0.95 (d, *J* = 6.8 Hz, 3H), 0.92 (d, *J* = 6.7 Hz, 3H); **$^{13}$C NMR** (150 MHz, $CDCl_3$) δ 91.9, 78.7, 74.7, 49.9, 33.6, 31.0, 27.3, 18.4, 18.3.

HRMS: (ESI) m/z calcd for $C_{10}H_{19}O_2S$ $[M+H]^+$ 203.1100, found 203.1098

***Determination of relative stereochemistry for THF 79***

Analysis of 2D NOESY of THF **79** supported the indicated stereochemistry.

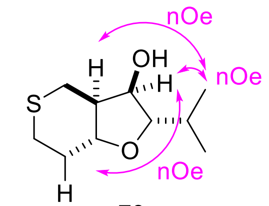


**79**

**Preparation of THF 80**

Following General Procedure G, **40d** (20 mg, 0.09 mmol) was dissolved in MeOH (0.18 mL) and subjected to microwave irradiation. The cyclized product **80** was purified by flash chromatography ($CH_2Cl_2$:MeOH – 97:3) to yield **80** (11.1 mg, 60% yield) as a clear oil.

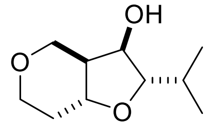


**80**

Data for THF **80**: **$[\alpha]_D^{20}$** = +8.9 (*c* = 1.1 in $CHCl_3$); **IR** (neat): $\upsilon$ = 3349, 2969, 2924, 1084, 1046, 880, 635 $cm^{-1}$; $^1$H NMR (600 MHz, $CDCl_3$) δ 4.17 (m, 2H), 4.03 (dd, *J* = 11.7, 4.5 Hz, 1H), 3.70 (dd, *J* = 11.0, 4.2 Hz, 1H), 3.52 (m, 1H), 3.30 (m, *J* = 12.0, 2.2 Hz, 1H), 2.11 (m, 1H), 1.76 (m, 1H), 1.69 (m, 1H), 1.39 (bs, 1H), 0.97 (d, *J* = 6.0 Hz, 3H), 0.94 (d, *J* = 6.0 Hz, 3H); $^{13}$C NMR (150 MHz, $CDCl_3$) δ 93.0, 77.2, 73.6, 67.2, 65.5, 48.9, 33.1, 31.0, 18.6, 18.4

HRMS: (ESI) m/z calcd for $C_{10}H_{22}O_3N$ $[M+NH_4]$ + 204.1594, found 204.1600

***Determination of relative stereochemistry for THF 80***

Analysis of 2D NOESY of THF **80** supported the indicated stereochemistry.

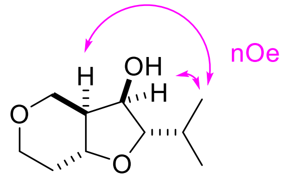


**80**

**Preparation of THF 81**

Following General Procedure G, to a stirred solution of chlorohydrin **61** (194 mg, 0.50 mmol) in MeOH (1 mL) was added $NaBH_4$ (37 mg, 1.0 mmol) in MeOH (2 mL). Once TLC analysis indicated complete consumption of **61**, the reaction mixture was quenched with aqueous $NH_4Cl$ (5 mL), extracted 3x with 10 mL of $CH_2Cl_2$, and concentrated under reduced pressure to afford diol **S61**. Without further purification, the crude reduction product dissolved in MeOH (1 mL) and subjected to microwave irradiation. Purification of crude **S81** by flash column chromatography (pentane:EtOAc – 60:40) afforded a white solid (20 mg, 18% yield). **S81** was stirred in acetone (0.90 mL) with TsOH (0.1 eq, 30 mg) and two heaping spatula tips of $MgSO_4$ to form the acetonide protected THF. The crude product **81** was purified by flash column chromatography (hexanes:EtOAc – 60:40) to afford **81** (18 mg) as a clear oil.

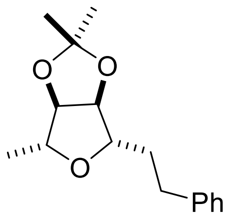

**81**

Data for THF **81**: $[\alpha]_D^{20}$ = -14.7 (*c* = 2.2 in $CHCl_3$); **IR** (neat): $\upsilon$ = 3027, 2957, 2929, 2871, 1708, 1381, 1210, 1074, 865, 699, 512 $cm^{-1}$; **$^1$H NMR** (600 MHz, $CDCl_3$) δ 7.29 (m, 2H), 7.21 (m, 3H), 4.37 (dd, *J* = 7.2, 4.9 Hz, 1H), 4.27 (dd, *J* = 7.2, 5.0 Hz, 1H), 3.92 (m, 1H), 3.83 (td, *J* = 6.7, 4.9 Hz, 1H), 2.78 (m, 1H), 2.72 (m, 1H), 1.93 (m, 2H), 2.53 (s, 3H), 1.34 (s, 3H), 1.33 (d, *J* = 7.7 Hz, 3H); **$^{13}$C NMR** (150 MHz, $CDCl_3$) δ 141.6, 128.4, 128.3, 125.8, 114.9, 86.2, 85.3, 83.3, 79.9, 35.4, 31.8, 29.7, 27.3, 25.4, 19.0

HRMS: (ESI) m/z calcd for $C_{16}H_{23}O_3[M+H]^+$ 263.1642, found 263.1635

***Determination of relative stereochemistry for THF 81***

Analysis of 2D NOESY of THF **81** supported the indicated stereochemistry.

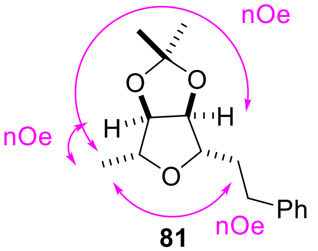

**81**

**Preparation of iminosugar 82**

Following General Procedure F, chlorohydrin **59** (95 mg, 0.29 mmol) was dissolved in dry THF (3 mL) with AcOH (17 µL, 0.29 mmol) and benzylamine (84 µL, 0.80 mmol). The resulting reaction was stirred for 1 hr. $NaBH_3CN$ (49 mg, 0.8 mmol) was added and the reaction mixture stirred for another 1hr. The crude product was purified by flash chromatography (hexanes:$Et_2O$ – 90:10) to afford **82** (24 mg, 21% yield) as a clear oil. Stereochemistry was assigned by analogy to the other iminosugars made by this process.

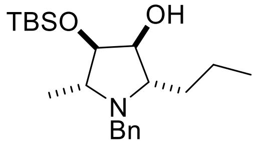


**82**

Data for iminosugar **82**: **$[\alpha]_D^{20}$** = +12.3 (*c* = 2.3 in $CHCl_3$); **IR** (neat): $\upsilon$ = 3549, 2956, 2929, 1253, 1123, 835, 776, 732, 698 $cm^{-1}$; **$^1$H NMR** (500 MHz, $CDCl_3$) δ 7.29 (m, 4H), 7.22 (m, 1H), 3.76 (d, J = 14.2 Hz, 1H), 3.72 (d, J = 14.2 Hz, 1H), 3.64 (m, J = 3.3, 5.7 Hz, 1H), 3.60 (m, J = 5.5, 6.7 Hz, 1H), 2.67 (m, 2H), 2.61 (d, J = 3.3 Hz, 1H), 1.41 (bs, 1H), 1.49-1.36 (m, 2H), 1.31-1.16 (m, 2H), 1.02 (d, J = 6.2 Hz, 3H), 0.91 (s, 9H), 0.85 (t, J = 7.1 Hz, 3H), 0.10 (s, 3H), 0.09 (s, 3H); **$^{13}$C NMR** (125 MHz, $CDCl_3$) δ 139.5, 129.0, 127.0, 126.7, 77.8, 74.6, 70.1, 62.9, 56.8, 35.8, 25.7, 18.9, 18.1, 18.0, 14.3, -4.6, -4.8

HRMS: (ESI) m/z calcd for $C_{21}H_{38}NO_2Si$ $[M+H]^+$ 364.2666, found 364.2668

**Preparation of iminosugar 83**

Following General Procedure F, chlorohydrin 60 (54 mg, 0.18 mmol) was dissolved in dry THF (2 mL) with AcOH (10 µL, 0.18 mmol) and benzylamine (49 µL, 81 0.46 mmol). The resulting reaction mixture was stirred for 1 hr. $NaBH_3CN$ (28 mg, 0.46 mmol) was added and stirred for another 1 hr. The crude product **83** was purified by flash chromatography (hexanes:EtOAc – 90:10) to afford **83** (16 mg, 25% yield) a clear oil. Stereochemistry was assigned by analogy to the other iminosugars made by this process.

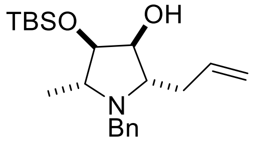


**83**

Data for iminosugar **83**: **$[\alpha]_D^{20}$** = +21.4 (*c* = 1.4 in $CHCl_3$); **IR** (neat): $\upsilon$ = 3550, 3028, 2065, 2955, 2928, 1253, 1124, 1092, 835, 776, 698 $cm^{-1}$; **$^1$H NMR** (500 MHz, $CDCl_3$) δ 7.29 (m, 4H), 7.23 (m, 1H), 5.85 (m, 1H), 5.02 (m, J = 1.7, 13.8 Hz, 2H), 3.80 (d, *J* = 14.1 Hz, 1H), 3.71 (d, *J* = 14.1 Hz, 1H), 3.68 (m, 1H), 3.60 (dd, *J* = 6.8, 5.3 Hz, 1H), 2.80 (dd, J = 7.4, 3.5 Hz, 1H), 2.72 (m, *J* = 6.3 Hz, 1H), 2.57 (d, *J* = 3.6 Hz, 1H), 2.13 (dddd, *J* = 13.8, 7.3, 3.5, 1.1 Hz, 1H), 2.00 (m, *J* = 14.3, 7.8, 1.3 Hz, 1H), 1.01 (d, *J* = 6.2 Hz, 3H), 0.91 (s, 9H), 0.11

(s, 3H), 0.09 (s, 3H); **$^{13}$C NMR** (125 MHz, $CDCl_3$) δ 139.8, 135.6, 128.9, 128.0, 126.8, 116.4, 77.8, 74.1, 69.7, 63.3, 57.4, 37.7, 25.8, 18.3, 18.0, -4.6, -4.8

HRMS: (ESI) m/z calcd for $C_{21}H_{36}NO_2Si$ $[M+H]^+$ 362.2510, found 362.2501

**Preparation of iminosugar 84**

Following General Procedure F, chlorohydrin **62** (24 mg, 0.07 mmol) was dissolved in dry THF (1 mL) with AcOH (4.5 μL, 0.07 mmol) and benzylamine (22 μL, 0.21 mmol). The resulting reaction mixture was stirred for 1 hr. $NaBH_3CN$ (12.5 mg, 0.21 mmol) was added and stirred for another 1 hr. The crude product **84** was purified by flash chromatography (hexanes:$Et_2O$ – 80:20) to afford **84** (9 mg, 32% yield) a clear oil.

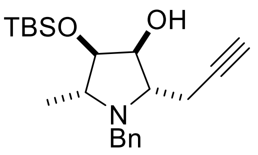

**84**

Data for iminosugar **84**: **$[\alpha]_D^{20}$** = +12.6 (*c* = 11 mg/mL in $CHCl_3$); **IR** (neat): $\upsilon$ = 3547, 3310, 2955, 2928, 2119, 1253, 1130, 835, 777, 699, 632 cm$^{-1}$; **$^1$H NMR** (500 MHz, $CDCl_3$) δ 7.36 - 7.27 (m, 4H), 7.23 (m, 1H), 3.89-3.81 (m, 2H), 3.75 (dd, *J* = 6.2, 4.8 Hz, 1H), 3.71 (d, *J* = 13.8 Hz, 1H), 2.91 (ddd, *J* = 6.9, 4.2, 2.8 Hz, 1H), 2.80 (m, 1H), 2.62 (d, *J* = 3.3 Hz, 1H), 2.19 (ddd, *J* = 16.9, 4.1, 2.7 Hz, 1H), 2.05 (ddd, *J* = 16.9, 6.8, 2.6 Hz, 1H), 1.94 (t, *J* = 2.6 Hz, 1H), 1.04 (d, *J* = 6.2 Hz, 3H), 0.92 (s, 9H), 0.12 - 0.11 (s, 6H); **$^{13}$C NMR** (125 MHz, $CDCl_3$) δ 139.7, 128.9, 128.1, 126.9, 82.2, 77.8, 74.6, 69.2, 68.7, 63.3, 57.5, 25.8, 23.5, 18.2, 18.0, -4.6, -4.7

HRMS: (ESI) m/z calcd for $C_{21}H_{34}NO_2Si$ $[M+H]^+$ 360.2353, found 360.2348

***Determination of relative stereochemistry for iminosugar 84***

Analysis of 2D NOESY of iminosugar **84** supported the indicated stereochemistry.

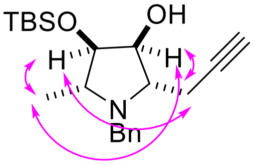

**84**

**Preparation of nucleoside analogue 86**

To a suspension of **68a** (100 mg, 0.228 mmol) in MeCN (2.0 mL) at 0 °C was added acetic acid (131 μl, 2.285 mmol), followed by sodium triacetoxyborohydride (242 mg, 1.142 mmol). The mixture was stirred at room temperature for 16h, at which time LCMS indicated complete conversion to the reduced product in approximately 2.5:1 selectivity. The reaction mixture was then diluted with water and ethyl acetate. The organic layer was washed with brine, dried over $MgSO_4$, filtered, and concentrated under reduced pressure. The crude reduced product was then diluted with MeCN (2.0 mL) and indium chloride (50.5 mg, 0.228 mmol) was added. The resulting reaction mixture was stirred overnight at 50 °C. The reaction mixture was then concentrated under reduced pressure and purified by flash column chromatography (25-100% ethyl acetate in hexanes) to afford nucleoside **86** (43 mg, 45%) as a white solid.

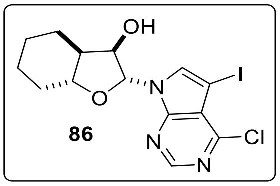

**86**

Data for nucleoside analogue **86**: $[\alpha]_D^{20}$ = -15.0 (*c* 0.17 in MeOH); **IR** (neat): $\upsilon$ = 3298, 2938, 2852, 1537, 1442, 1204, 1108 $cm^{-1}$; $^1$**H NMR** (600 MHz, $CDCl_3$): δ 8.68 (s, 1H), 7.98 (s, 1H), 6.11 (s, 1H), 5.59 (d, J = 4.7 Hz, 1H), 4.23 (dd, J = 4.7, 4.4 Hz, 1H), 3.64 (ddd, J = 11.1, 11.1, 4.0 Hz, 1H), 2.08 (m, 1H), 1.72 – 1.82 (4H), 1.49 (m, 1H), 1.19 – 1.40 (m, 3H); $^{13}$**C NMR** (150 MHz, $CDCl_3$): δ 151.1, 150.7, 150.1, 133.3, 116.5, 91.0, 80.9, 76.1, 53.4, 47.7, 40.8, 24.8, 23.6, 23.3

HRMS ($EI^+$) calcd for $C_{14}H_{16}ClIN_3O_2^+$ 419.9970; Found 419.9952.

***Determination of relative stereochemistry for nucleoside 86***

Analysis of 2D NOESY of nucleoside **86** supported the indicated stereochemistry

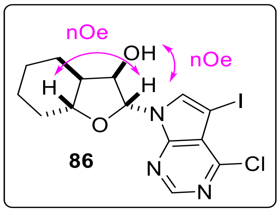

**86**

**Preparation of nucleoside analogue 87**

To a stirred solution of fluorohydrins **70** (0.105 g, 0.344 mmol, 1.0 equiv) in MeCN (3.00 mL) at -15˚C was added tetramethylammoniumtriacetoxyborohydride (0.453 g, 1.72 mmol, 5.0 equiv) and acetic acid (0.190 mL, 3.44 mmol, 10 equiv). The resulting mixture was stirred 16 hours. The reaction mixture was then diluted with a saturated solution of Rochelle salt and washed three times with $CH_2Cl_2$. The organic layer was separated, dried over $MgSO_4$, filtered, and

concentrated under reduced pressure. The crude product **S70** was purified by flash chromatography (EtOAc:pentane – 70:30) to afford **S70** as a white solid (0.076 g, 72%)

To a stirred solution of *syn*-diol-fluorohydrins **S70** (0.076, 0.248 mmol, 1.0 equiv.) in MeCN (2.50 mL) was added $InCl_3$ (0.014 g, 0.062 mmol, 0.25 equiv.) and the reaction mixture was stirred for 24 hours. The reaction mixture was diluted with $CH_2Cl_2$ and was washed with saturated sodium bicarbonate solution. The organic layer was separated, dried over $MgSO_4$, filtered, and concentrated under reduced pressure. The ratio of anomers (α:β) was determined to be 2.5:1 by $^1$H NMR spectroscopic analysis of the crude product. The crude product **87** was purified by flash chromatography (EtOAc:pentane – 25:75) to afford nucleoside **87** (α-anomer) as a colorless oil (42.7 mg, 60%)

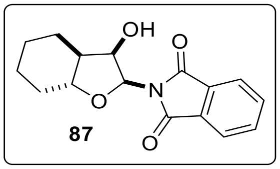


Data for nucleoside analogue **87** (α-anomer): **$[\alpha]_D^{20}$** = +46.6 (*c* 0.38 in $CH_2Cl_2$); **IR** (neat): $\upsilon$ = 3475, 2935, 1708, 1370, 720 $cm^{-1}$; **$^1$H NMR** (600 MHz, $CDCl_3$): δ 7.88 (m, 2H), 7.77 (m, 2H), 6.13 (d, *J* = 5.0 Hz, 1H), 4.40 (ddd, *J* = 11.8, 5.0, 4.8 Hz, 1H), 4.03 (ddd, *J* = 10.6, 10.6, 4.1 Hz, 1H), 3.13 (d, *J* =11.9 Hz, 1H), 2.22 (m, 1H), 1.94 (m, 1H), 1.85 (m, 2H), 1.62 (dddd, *J* = 11.9, 11.9, 4.6, 3.2 Hz, 1H), 1.51 (m, 1H), 1.23 – 1.40(3H); **$^{13}$C NMR** (150 MHz, $CDCl_3$): δ 169.1, 134.6, 132.1, 123.8, 84.4, 81.1, 75.3, 51.4, 31.7, 25.4, 24.0, 24.0

HRMS ($EI^+$) calcd for $C_{16}H_{18}NO_4$ [M + $H^+$] 288.1230; found 288.1246

***Determination of relative stereochemistry for nucleoside 87***

Analysis of 2D NOESY of nucleoside **87** (α-anomer) supported the indicated stereochemistry

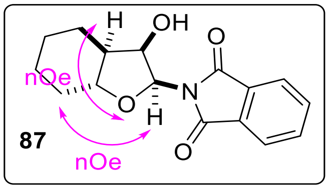


**Preparation of nucleoside analogue 88**

To a stirred solution of fluorohydrins **69** (0.097 g, 0.30 mmol, 1.0 equiv) in MeCN (3.00 mL) at -15˚C was added tetramethylammoniumtriacetoxyborohydride (0.395 g, 1.50 mmol, 5.0 equiv) and acetic acid (0.172 mL, 1.50 mmol, 10 equiv). The resulting mixture was stirred 16 hours. The reaction mixture was then diluted with a saturated solution of Rochelle salt and washed

three times with $CH_2Cl_2$. The organic layer was separated, dried over $MgSO_4$, filtered, and concentrated under reduced pressure. The crude product **S69** was purified by flash chromatography (EtOAc:pentane – 70:30) to afford **S69** as a white solid (0.068 g, 70%)

To a stirred solution of *syn*-diol-fluorohydrins **S69** (0.047, 0.143 mmol, 1.0 equiv.) in MeCN (1.43 mL) was added $InCl_3$ (7.9 mg, 0.036 mmol, 0.25 equiv.) and the reaction mixture was stirred for 24 hours. The reaction mixture was diluted with $CH_2Cl_2$ and was washed with saturated sodium bicarbonate solution. The organic layer was separated, dried over $MgSO_4$, filtered, and concentrated under reduced pressure. The ratio of anomers (α:β) was determined to be 3:1 by $^1$H NMR spectroscopic analysis of the crude product. The crude product **88** was purified by flash chromatography (EtOAc:pentane – 40:60) to afford **88** (α-anomer) as a colorless oil (23.7 mg, 73%).

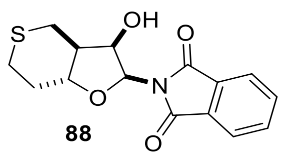

88

Data for nucleoside analogue **88** (α-anomer): **$[\alpha]_D^{20}$** = +18.6 (*c* 2.37 in $CH_2Cl_2$); **IR** (neat): $\upsilon$ = 3475, 2923, 1774, 1709, 1373, 719 $cm^{-1}$; **$^1$H NMR** (600 MHz, $CDCl_3$): δ 7.88 (m, 2H), 7.77 (m, 2H), 6.13 (d, *J* = 4.9 Hz, 1H), 4.40 (ddd, *J* = 11.5, 4.7, 4.7 Hz, 1H), 4.03 (ddd, *J* = 11.2, 11.2, 3.6 Hz, 1H), 3.35 (d, *J* = 11.9 Hz, 1H), 2.98 (dd, *J* = 13.1 11.9 Hz, 1H), 2.82 (m ,2H), 2.69 (m, 1H), 2.50 (m, 1H), 2.10 (m ,1H), 1.74 (m , 1H); **$^{13}$C NMR** (150 MHz, $CDCl_3$): δ 169.2, 134.8, 131.9, 124.0, 83.0, 80.2, 75.2, 51.3, 33.5, 27.6, 27.4

HRMS (EI$^+$) calcd for $C_{15}H_{19}N_2O_4S$ [M + $NH_4^+$] 323.1060; found 323.1037

***Determination of relative stereochemistry for nucleoside 88***

Analysis of 2D NOESY of nucleoside **88** (α-anomer) supported the indicated stereochemistry

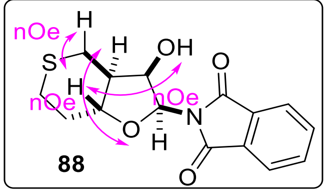

88

**Preparation of carbocycle 90**

Following General Procedure E, to a stirred solution of 5-(methanesulfonyl)-1-phenyl-1H-tetrazole (0.126 g, 0.560 mmol) in dry THF (0.70 mL) at -78ºC was added dropwise a 1 M LiHMDS (0.560 mL, 0.560 mmol) and the resulting reaction mixture was stirred for 30 minutes.

A solution of fluorohydrin **51** (0.052 g, 0.224 mmol) in dry THF (0.45 mL) was then added dropwise and the reaction mixture was allowed to stir for 5 hrs at -78°C. Purification of crude alkene **S51** by flash chromatography (pentane:EtOAc – 80:20) afforded alkene **S51** (0.034 g, 65 % yield) as a colorless oil**.** A mixture Grubbs II catalyst (2.9 mg) and alkene **S51** (0.034 g, 0.148 mmol) in dry toluene (5.91 mL) was purged with $N_2$ for 45 minutes in a sealed reaction vessel and subsequently heated to 80°C for 6 hrs. Purification of crude carbacycle **90** by flash chromatography (pentane:EtOAc – 75:25) afforded carbacycle **90** (0.019 g, 63 % yield) as a white solid.

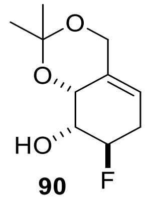

**90**

Data for carbacycle **90**: **$[\alpha]_D^{20}$** = -32.8 (*c* 0.50 in $CH_2Cl_2$); **$^1$H NMR** (600 MHz, $CDCl_3$): δ 5.45 (s, 1H), 4.96 (m, 1H), 4.61 (s, 1H), 4.51 (d, *J* = 13.5 Hz, 1H), 4.13 (m, 1H), 4.10 (d, *J* = 13.5 Hz, 1H), 2.71 (dd, J = 3.8, 1.5 Hz, 1H), 2.62 (dddd, *J* = 43.7, 19.2, 5.6, 2.6 Hz, 1H), 2.31 (dd, *J* = 21.2, 19.2 Hz, 1H), 1.57 (s, 3H), 1.44 (s, 3H); **$^{13}$C NMR** (150 MHz, $CDCl_3$): δ 128.2, 117.8, 99.4, 88.5 (d, *J* = 166.5 Hz), 66.1 (d, *J* = 2.7 Hz), 65.9 (d, *J* = 27.7 Hz), 63.7, 29.0, 27.4 (d, *J* = 22.1), 20.0.

HRMS ($EI^+$) calcd for $[C_{10}H_{16}FO_3]^+$ 203.1078; found 203.1058

**Preparation of carbacycle 91**

Following General Procedure E, to a stirred solution of 5-(methanesulfonyl)-1-phenyl-1H-tetrazole (0.134 g, 0.60 mmol) in dry THF (0.75 mL) at -78$^o$C was added dropwise a 1 M LiHMDS (0.60 mL, 0.60 mmol) and the resulting reaction mixture was stirred for 30 minutes. A solution of fluorohydrin **66** (0.060 g, 0.30 mmol) in dry THF (1.20 mL) was then added dropwise and the reaction mixture was allowed to stir for 5 hrs at -78°C. Purification of the crude alkene **S66** by flash chromatography (pentane:EtOAc – 90:10) afforded alkene **S66** (0.031 g, 52 % yield) as a white solid**.** A mixture Grubbs II catalyst (5.5 mg, 0.05 equiv.) and alkene **S66** (0.026 g, 0.13 mmol) in dry toluene (5.30 mL) was purged with $N_2$ for 30 minutes in a sealed reaction vessel and subsequently heated to 80°C for 8 hrs. Purification of the crude carbacycle **91** by flash chromatography (pentane:$Et_2O$ – 85:15) afforded carbacycle **91** (15.9 mg, 72 % yield) as a colorless oil.

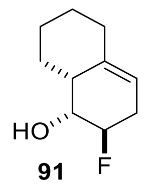

**91**

Data for carbacycle **91**: **$[\alpha]_D^{20}$** = -111.3 (*c* 0.3 in $CH_2Cl_2$); **IR** (neat): $\upsilon$ = 3418, 2925, 2853, 1447, 1003 cm$^{-1}$;**$^1$H NMR** (600 MHz, $CDCl_3$): δ 5.17 (m, 1H), 4.68 (dddd, J = 52.2, 13.8, 8.2, 5.6 Hz, 1H), 3.99 (m, 1H), 2.50 (m, 1H), 2.44 (m,

1H), 2.26 (m, 1H), 2.23 (m, 1H), 2.09 (d, J = 3.7 Hz), 2.06 (m, 1H), 1.95 (m, 1H), 1.87 (m ,1H), 1.81 (m ,1H), 1.39 (ddd, J = 13.2, 3.8, 3.8 Hz, 1H), 1.24 (ddd, J = 13.0, 3.8, 3.8 Hz, 1H), 1.13 (dd, J = 12.8, 3.7 Hz, 1H); **$^{13}$C NMR** (150 MHz, $CDCl_3$): δ 136.9 (d, J = 1.8 Hz), 113.0 (d, J = 8.8 Hz), 90.6 (d, J = 171.2 Hz) , 71.2 (d, J = 19.2 Hz), 42.4 (d, J = 4.8 Hz), 35.4, 29.8 (d, J = 19.8 Hz), 28.7, 28.3; **$^{19}$F NMR** (470 MHz, $CDCl_3$): δ –191.8

HRMS ($EI^+$) calcd for $C_{10}H_{16}FO_4$ 171.1180; found 171.1154

***Determination of relative stereochemistry for carbacycle 91***

Analysis of 2D NOESY of carbacycle **91** supported the indicated stereochemistry

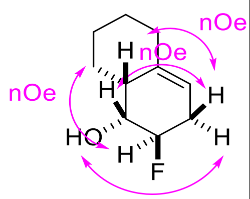

**91**

**Preparation of carbocycle 92**

Following General Procedure E, to a stirred solution of 5-(methanesulfonyl)-1-phenyl-1H-tetrazole (0.099 g, 0.44 mmol) in dry THF (0.55 mL) at -78$^o$C was added dropwise a 1 M LiHMDS (0.440 mL, 0.440 mmol) and the resulting reaction mixture was stirred for 30 minutes. A solution of fluorohydrin **67** (0.048 g, 0.220 mmol) in dry THF (2.20 mL) was then added dropwise and the reaction mixture was allowed to stir for 3 hrs at -78°C. Purification of the crude alkene **S67** by flash chromatography (pentane:EtOAc – 85:15) afforded alkene **S67** (0.029 g, 61 % yield) as a off-white solid**.** A mixture Grela catalyst (5.9 mg, 0.10 equiv.) and alkene **S67** (0.019 g, 0.088 mmol) in dry toluene (3.52 mL) was purged with $N_2$ for 30 minutes in a sealed reaction vessel and subsequently heated to 80°C for 8 hrs. Purification of the crude carbacycle **92** by flash chromatography (pentane:EtOAc – 90:10) afforded carbacycle **92** (0.013 g, 71 % yield) as a white solid.

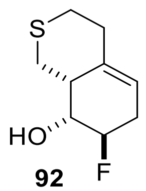

**92**

Data for carbacycle **92**: **$[\alpha]_D^{20}$** = -77.2 (*c* 0.53 in $CH_2Cl_2$); **IR** (neat): $\upsilon$ = 3424, 2924, 1426, 1290, 1088, 1057, 1021 cm$^{-1}$;**$^1$H NMR** (600 MHz, $CDCl_3$): δ 5.27 (m, 1H), 4.64 (dddd, J = 52.7, 15.5, 9.3, 6.3 Hz, 1H), 4.00 (ddd, J = 16.3, 9.3, 7.1 Hz, 1H), 3.01 (dd, J = 12.9, 2.9 Hz, 1H), 2.85 (m, 1H), 2.63 (m, 2H), 2.55 (m, 1H), 2.53 (m, 1H), 2.43 (dd, J = 12.8, 12.4 Hz, 1H), 2.35 (m, 1H), 2.32 (m, 1H), 2.27 (m, 1H); **$^{13}$C NMR** (150 MHz, $CDCl_3$): δ 138.9, 116.2 (d, J = 10.4 Hz), 90.3 (d, J =

172.2 Hz), 71.5 (d, J = 17.8 Hz), 45.3 (d, J = 5.6 Hz), 31.8, 30.8, 30.3 (d, J = 20.0 Hz); **$^{19}$F NMR** (470 MHz, $CDCl_3$): δ –191.4

HRMS (EI$^+$) calcd for $[C_9H_{14}FOS]^+$ 189.0744; found 189.0757

***Determination of relative stereochemistry for carbacycle 92***

Analysis of 2D NOESY of carbacycle **92** supported the indicated stereochemistry

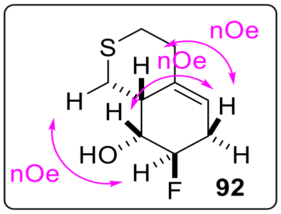


**Preparation of carbocycle 93**

Following General Procedure E, to a stirred solution of 5-(methanesulfonyl)-1-phenyl-1H-tetrazole (0.066 g, 0.295 mmol) in dry THF (0.37 mL) at -78ºC was added dropwise a 1 M LiHMDS (0.295 mL, 0.295 mmol) and the resulting reaction mixture was stirred for 30 minutes. A solution of fluorohydrin **71** (0.042 g, 0.134 mmol) in dry THF (0.54 mL) was then added dropwise and the reaction mixture was allowed to stir for 3 hrs at -78°C. Purification of the crude alkene **S71** by flash chromatography (pentane:$Et_2O$ – 80:20) afforded alkene **S71** (0.023 g, 56 % yield) as a colorless oil**.** A mixture Grubbs II catalyst (2.9 mg) and alkene **S71** (0.021 g, 0.067 mmol) in dry toluene (2.70 mL) was purged with $N_2$ for 30 minutes in a sealed reaction vessel and subsequently heated to 90°C for 6 hrs. Purification of the crude carbacycle **93** by flash chromatography (pentane:EtOAc – 80:20) afforded carbacycle **93** (0.013 g, 74 % yield) as a white solid.

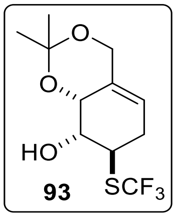


Data for carbacycle **93**: **$[\alpha]_D^{20}$** = -54.3 (*c* 0.83 in $CH_2Cl_2$); **IR** (neat): $\upsilon$ = 3470, 1430, 1111, 879, cm$^{-1}$;**$^1$H NMR** (600 MHz, $CDCl_3$): δ 5.52 (m, 1H), 4.62 (m, 1H), 4.48 (dd, *J* = 13.4, 2.6 Hz, 1H), 4.11 (m, 1H), 4.06 (d, *J* = 13.4 Hz, 1H), 3.75 (m, 1H), 3.00 (d, *J* = 18.8 Hz, 1H), 2.94 (d, *J* = 1.4 Hz, 1H), 2.21 (ddd, *J* = 18.8, 4.4, 2.1 Hz, 1H), 1.58 (s, 3H), 1.43 (s, 3H); **$^{13}$C NMR** (150 MHz, $CDCl_3$): δ 130.9 (q, *J* = 307.7 Hz), 128.8, 119.6, 99.6, 67.6, 65.7, 63.8, 41.5 (q, *J* = 1.6 Hz), 29.0, 27.4, 20.1; **$^{19}$F NMR** (470 MHz, $CDCl_3$): δ –39.9

HRMS (EI$^+$) calcd for $[C_{11}H_{16}F_3O_3S]^+$ 285.0767; found 285.0780

### *Determination of relative stereochemistry for carbacycle 93*

Analysis of 2D NOESY for carbacycle **93** supported the indicated stereochemistry

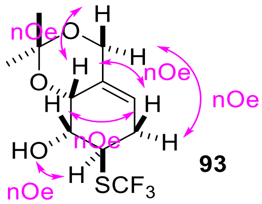

**93**

### Synthesis of 2-fluoro-2-deoxy- D-altrose acetonide (94)

The fluorohydrin **58** (0.150 g, 0.397 mmol, 1 equiv.) in MeCN (0.45 mL, 0.9 M) was added to a stirred solution of $Me_4NBH(OAc)_3$ (0.520 g, 1.98 mmol, 5 equiv.) and AcOH (0.23 mL, 3.97 mmol, 10 equiv.) in MeCN (2.0 mL, 0.2 M) at -25°C and the resulting mixture was stirred for 24 hrs. The reaction mixture was then quenched by addition of a saturated aqueous solution of sodium tartrate. The aqueous layer was removed and extracted four times with $CH_2Cl_2$, and the combined organic layers were dried over $MgSO_4$ and concentrated under reduced pressure. Purification of the crude product by flash chromatography (hexanes:EtOAc – 75:25) afforded the 1,3 *syn*-fluorodiol (0.114 g, 76 %).

To a cold solution (0 °C) of the 1,3-*syn*-fluorodiol (0.060 g, 0.16 mmol) in THF (1.6 mL) was added a solution of tetrabutylammonium fluoride in THF (1 M, 0.18 mL, 0.18 mmol), and the reaction mixture was stirred for 30 minutes. The reaction mixture was then diluted with $Et_2O$ (2 mL) and was washed with a solution of saturated aqueous ammonium chloride. The organic layer was dried over $MgSO_4$ and concentrated under reduced pressure. Purification of the crude product by flash chromatography ($CH_2Cl_2$-MeOH 95:5) afforded the deprotected 1,3-*syn*-fluorotriol (32 mg, 91% yield). To a cold (0°C) solution of bis-(acetoxy)iodobenzene (35 mg, 0.108 mmol) and the 1,3-*syn*-fluorotriol (23 mg, 0.103 mmol), in $CH_2Cl_2$ (1.1 mL), was added 2,2,6,6-tetramethylpiperidinyloxy (1 mg, cat.) and the reaction mixture was allowed to gradually warm to room temperature and stirred for 5 hrs and the reaction mixture was concentrated under reduced pressure. Purification of the crude fluorohydrin **94** (dr = 1:1) by flash chromatography (pentane-EtOAc 5:5) afforded **94** (15 mg, 65% yield) as a clear oil.

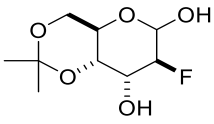

**94**

Data for 2-fluoro-2-deoxy- L-altrose acetonide (**94**) $[\alpha]_D^{20}$ = + 2.5 (*c* 0.83 in $CHCl_3$); **IR** (neat)**:** $\upsilon$ = 3413, 2918, 1078, 1043 $cm^{-1}$; **$^1$H NMR** (600 MHz, $CDCl_3$): $\delta$ 5.12 (2H), 4.68 (2H), 4.38 (1H), 4.27 (2H), 4.16 (1H), 3.91 (7H), 3.27 (1H), 2.68 (1H), 2.30 (1H), 1.49 (12H); **$^{13}$C NMR** (150 MHz, $CDCl_3$): $\delta$

100.4, 100.3, 92.9, 91.8, 89.2, 86.5, 69.0, 68.7, 67.7, 67.6, 64.4, 62.6, 62.3, 59.4, 29.2, 29.1, 19.5, 19.4 **$^{19}$F NMR** (470 MHz, $CDCl_3$): δ -194.9, -216.5

**HRMS** (ESI) *m/z* calcd for $C_9H_{16}FO_5$ $[M + H]^+$ 223.0976, found 223.0965

**Synthesis of 2-fluoro-2-deoxy-L-galactose (95)**

To a cold (0 °C), stirred solution of fluorohydrin **58** (0.189 g, 0.500 mmol, 1.0 equiv.) in dry THF (0.1 M) was added a solution of catechol borane in THF (1.1 mL, 1.0 M, 2.2 equiv.). The resulting mixture was allowed to warm gradually to room temperature and was then stirred for an additional 45 minutes or until complete consumption of starting chlorohydrin was observed by TLC analysis. The mixture was then diluted with MeOH (to 0.05 M) and a solution of saturated aqueous sodium tartrate was added. The biphasic mixture was stirred vigorously for 2 hours, after which time the aqueous layer was removed and extracted three times with $Et_2O$. The combined organic layers were dried over $MgSO_4$, concentrated under reduced pressure, and the crude product was purified by flash chromatography (pentane- EtOAc; 2:1) to yield the 1,3-*anti*-fluorodiol (0.056 g, 82 %)

To a cold solution (0 °C) of the 1,3-*anti*-fluorodiol (0.076 g, 0.20 mmol, 1 equiv.) in THF (2.0 mL) was added a solution of tetrabutylammonium fluoride in THF (1 M, 0.22 mL, 0.22 mmol, 1.1 equiv.), and the reaction mixture was stirred for 4 hrs. The reaction mixture was then diluted with $Et_2O$ (2 mL) and was washed with a solution of saturated aqueous ammonium chloride. The organic layer was dried over $MgSO_4$ and concentrated under reduced pressure. Purification of the crude product by flash chromatography ($CH_2Cl_2$-MeOH 95:5) afforded the deprotected 1,3-*anti*-fluorotriol (41 mg, 91% yield) (See **Pre95**-**XRD** for X-ray). To a cold (0°C) solution of bis-(acetoxy)iodobenzene (12.9 mg, 0.040 mmol) and the 1,3-*anti*-fluorotriol (9 mg, 0.04 mmol), in $CH_2Cl_2$ (0.40 mL), was added 2,2,6,6-tetramethylpiperidinyloxy (1 mg, cat.) and the reaction mixture was allowed to gradually warm to room temperature and stirred for 8 hrs and the reaction mixture was concentrated under reduced pressure. Purification of the crude fluorohydrin (dr 1:0.1.0) by flash chromatography (pentane-EtOAc 5:5) afforded (4.6 mg, 51 % yield) as a clear oil. The purified product (4.6 mg, 0.020 mmol) was then dissolved in $CH_2Cl_2$ (0.20 mL) and and 0.05 mL of TFA was added. The reaction mixture was left to stir for 24 hrs and the solvent was removed under reduced pressure to give pure **95** (4.3 mg, 100%) as a colorless oil. The data for **95** matched those of previously reported for 2-fluoro-2-deoxy-D-galactose.[2]

**Synthesis of fluorohydrin 54 and 2-fluoro-2-deoxy migalastat (96)**

Following General Procedure B, a solution of 3-*N-Cbz*-aminopropanal (0.10 g, 0.483 mmol), NFSI (0.152 g, 0.483 mmol), D-proline (0.056 g, 0.483 mmol) and $NaHCO_3$ (0.041 g, 0.483 mmol) was stirred for 3 hours at -10 °C in DMF (0.65 mL). Dioxanone **13** (0.039 mL, 0.322 mmol) in $CH_2Cl_2$ (5.8 mL) was stirred for 24 hours. Purification of the crude fluorohydrin **54** by flash chromatography (pentane-EtOAc 3:1) afforded fluorohydrin **54** (0.056 g, 49 % yield) as a yellow oil. $^1$H-NMR spectroscopic analysis of this material indicated that it exists as a complicated 1:1 mixture of fluorohydrin **54**:hemiminals (1:1 mixture of diastereomers).

Following General Procedure I, the fluorohydrin **54** (0.051 g, 0.144 mmol) and Pd/C powder were stirred in MeOH (1.4 mL) with bubbling in $H_2$ gas for 18 hrs. The Pd/C was filtered off, the solvent was removed under reduced pressure, and the crude product (dr 7:1) was purified with flash chromatography (EtOAc: pentanes; 40:60) to give a white powder (0.024 g, 83 %). The purified product (0.028 g, 0.139 mmol) was then dissolved in MeOH (1.4 mL) and 0.5 mL of 1 M HCl was added. The reaction mixture was left to stir for 24 hrs and the solvent was removed under reduced pressure to give pure **96** (0.022 g, 97%).

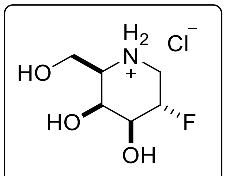
**96**

Data for 2-fluoro-2-deoxy migalastat (**96**): $[\alpha]_D^{20}$ = + 10.7 (*c* 1.88 in MeOH); **IR** (neat)**:** $\upsilon$ = 3307, 2952, 2464, 1406, 1111, 1055 cm$^{-1}$; **$^1$H NMR** (500 MHz, MeOD): $\delta$ 5.25 (dddd, *J* = 49.2, 10.8, 9.3, 5.6 Hz, 1H), 4.06 (m, 1H), 3.80 (m, 3H), 3.57 (ddd, *J* = 12.4, 5.6, 2.3 Hz, 1H), 3.40 (dd, *J* = 8.1, 5.3 Hz, 1H), 3.08 (m, 1H); **$^{13}$C NMR** (150 MHz, MeOD): $\delta$ 88.5 (*J* = 175.5 Hz), 73.1 (d, *J* = 17.7 Hz), 68.9 (d, *J* = 10.1 Hz), 62.0, 60.4, 45.0 (d, *J* = 32.6); **$^{19}$F NMR** (470 MHz, MeOD):δ -204.5

HRMS (ESI) *m/z* calcd for $C_6H_{13}FNO_3^+$ $[M + H]^+$ 166.0874, found 166.0893

*Determination of enantiomeric excess of fluorohydrin **54***

Following General Procedure B, using a 1:1 mixture of L: D - proline, a racemic sample of the fluorohydrin **54** was prepared. Following General Procedure **I**, the optically enriched and racemic samples of fluorohydrin **54** (0.055 g, 0.155 mmol) were converted into their corresponding cyclized products. These were then diacylated with (*R*)-(+)-MTPA-OH (3 equiv.), DIC (6 equiv.), pyridine (3 equiv.), and 4-dimethylaminopyridine (cat.) in $CH_2Cl_2$ (0.10 M). By analysis of $^{19}$F NMR it was determined that the enantiomeric excess was 92 %.

**Synthesis of (*5R*)-5-D-ribo-fluorophytosphingosine (97)**

To a stirred solution of the fluorohydrin **54** (0.187 g, 0.50 mmol, 1.0 equiv.) in 5.0 mL of THF (0.1 M) was added to benzylamine (0.137 mL, 1.25 mmol, 2.5 equiv.) and glacial acetic acid (0.030 g, 0.50 mmol, 1.0 equiv.), and the resulting mixture was stirred at 20°C for 2 hrs or until complete conversion into the corresponding imine was accomplished (as determined by 1H NMR spectroscopic analysis of small samples removed from the reaction mixture). $NaCNBH_3$ (0.080 g, 1.25 mmol, 2.5 equiv.) was then added and the mixture was stirred for a further 1 hr. The reaction mixture was then diluted with $CH_2Cl_2$ to a concentration of 0.05 M and treated with water. The layers were separated and the organic layer was washed with brine, dried ($MgSO_4$), and concentrated under reduced pressure. The crude product was purified by flash chromatography ($CH_2Cl_2$-MeOH; 15:1) to afford the reductive amination product (0.206 g, 88 % yield). Pd/C (2 mg) was added to a stirred solution of purified product (9.3 mg, 0.02 mmol, 1.0 equiv.) in 0.20 mL of MeOH (0.1 M) under a $H_2$ atmosphere. After 24 hrs the reaction was filtered, concentrated under reduced pressure, and purified by flash column chromatography ($CH_2Cl_2$-MeOH; 20:1) to give the debenzylated product (7 mg, 93 %). A solution of the debenzylated product (9 mg, 0.024 mmol) in 0.25 mL MeOH (0.1 M) was added 0.05 mL of 1 M HCl and left for 24 hrs. The reaction mixture was then concentrated under reduced pressure to afford pure **97** (8.7 mg, 98 %).

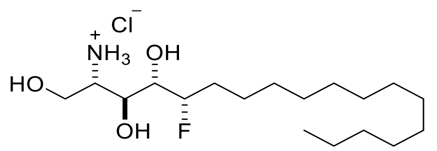


**97**

Data for (5*R*)-5-D-ribo-fluorophytosphingosine (**97**): **[$\alpha$]$_D^{20}$** = -4.5 (*c* 0.75 in DMSO-$d_6$); **IR** (neat)**:** $\upsilon$ = 3425, 2924, 1025, 1005, 822, 760, 614 cm$^{-1}$; **$^1$H NMR** (600 MHz, DMSO-$d_6$): $\delta$ = 7.84 (br s), 4.69 (ddd, *J* = 47.5, 8.6, 4.7 Hz, 1H), 3.80 (dd, *J* = 9.8, 2.7 Hz, 1H), 3.75 (dd, *J* = 11.2, 3.8 Hz, 1H), 3.58 (dd, *J* = 11.0, 9.4 Hz, 1H), 3.28 (dd, *J* = 29.6, 9.8 Hz, 1H), 1.77 (m, 1H), 1.56 (m, 1H), 1.26 (m, 26 H), 0.87 (dd, J = 6.8, 6.8 Hz, 3H); **$^{13}$C NMR** (150 MHz, DMSO-$d_6$): $\delta$ = 91.8 (*J* = 173.1 Hz), 71.1 (d, *J* = 18.1 Hz), 67.8 (d, *J* = 5.0 Hz), 56.9, 54.5, 31.3, 30.4 (d, *J* = 21.4 Hz), 29.0, 29.0, 29.0, 29.0, 28.9, 28.9, 28.7, 24.8, 24.8, 22.1, 13.9; **$^{19}$F NMR** (470 MHz, DMSO-$d_6$):δ -201.0

**HRMS** (ESI) *m/z* calcd for $C_{18}H_{39}FNO_3^+$ [M + H]$^+$ 336.2908, found 336.2920

## Supplementary Tables and Figures

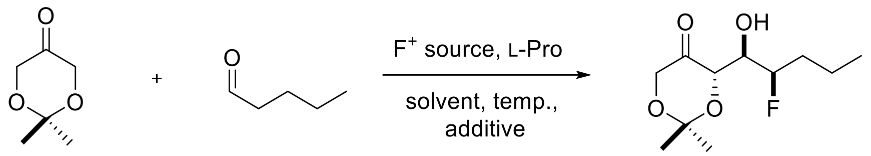


| Entry | (*S*)-Pro equiv. | Temp. (˚C) | Solvent (M) | Additive | $F^+$ source | Yield |
|---|---|---|---|---|---|---|
| 1 | 0.8 | RT | $CH_3CN$ | None | Selectfluor | 19 % |
| 2 | 0.8 | 0 | $CH_2Cl_2$ | None | NFSI | < 5% |
| 3 | 0.8 | 0 | $CH_2Cl_2$ | TFA | NFSI | < 5 % |
| 4 | 0.3 | RT | $CH_3CN$ /$CH_2Cl_2$ | None | Selectfluor | 32 % |
| 5 | 0.8 | RT | THF/IPA | None | Selectfluor | < 5% |
| 6 | 1.5 | 4 | DMF/$CH_2Cl_2$ | TFA | NFSI | 21% |
| 7 | 1.0 | 4 | DMF/$CH_2Cl_2$ | $NaHCO_3$ | NFSI | 54% |
| 8 | 1.0 | -10 | DMF/$CH_2Cl_2$ | None | NFSI | 18% |

**Supplementary Table 1.** Optimization of αFAR reaction.

*Determination of DKR in αFAR*

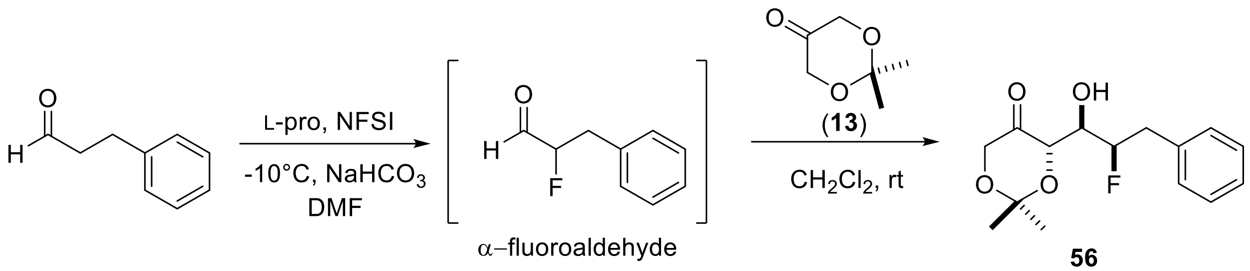


| Entry | Time (hrs) | % ee | Conversion to 56[c] |
|---|---|---|---|
| **1** | 0[a] | 23 | - |
| **2** | 1[b] | 23 | Not detected |
| **3** | 2[b] | 23 | 46% |
| **4** | 4[b] | 23 | 53% |
| **5** | 8[b] | 23 | 83% |

**Supplementary Table 2.** Monitoring %ee of α-fluoroaldehyde.[a]Before addition of ketone.[b]After addition of ketone.[c]Based on remaining ketone.

Following General Procedure B, the aFAR was stopped at different time intervals to monitor the enantiopurity of the α-fluoroaldehyde over the course of the reaction. The enantiomeric α-fluoroaldehydes were reduced in situ with $NaBH_4$ and separated by chiral HPLC using a CHIRALPAK IG column; flow rate 0.6 mL/min; eluent: hexanes-*i*PrOH 98:2; detection at 260 nm; retention time = 33.57 min and 35.10 min.The enantiomeric excess of the intermediate α-fluoroaldehydes were determined using the same method for each time interval shown above (Table S1).

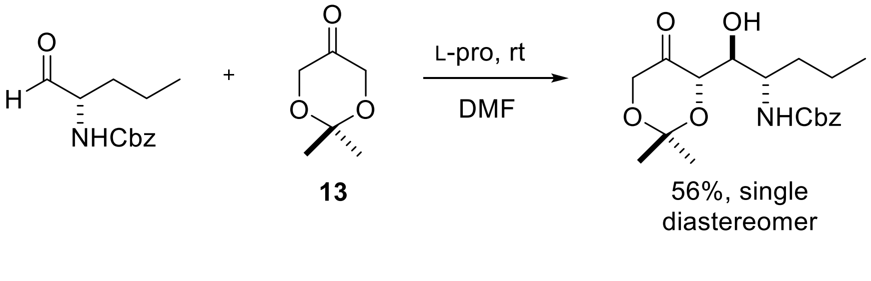


**Supplementary Scheme 1**. L-proline catalyzed aldol reaction between (*S*)-2-Cbz-aminopentanal and dioxanone (**13**).

L-proline catalyzed aldol reaction

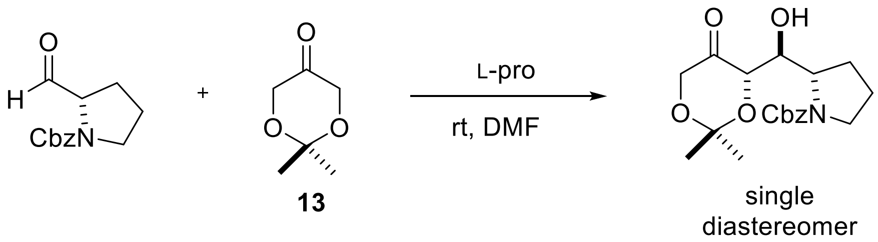


D-proline catalyzed aldol reaction

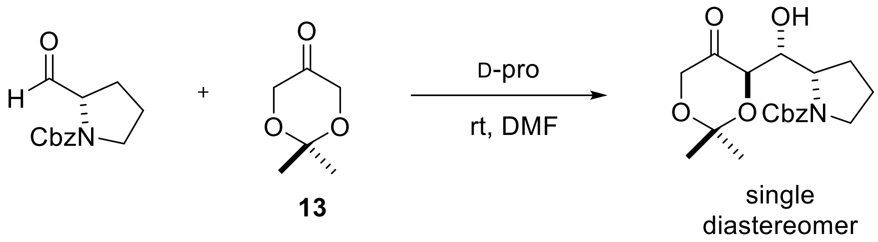


**Supplementary Scheme 2** L- and D-proline catalyzed aldol reactions between (*S*)-N-Cbz-prolinal and dioxanone (**13**).

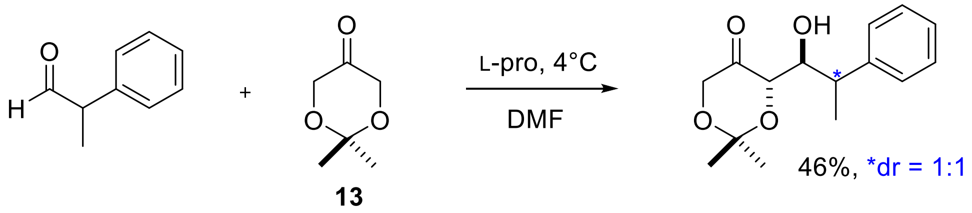


**Supplementary Scheme 3**. L-proline catalyzed aldol reaction between 2-phenylpropanal and dioxanone (**13**).

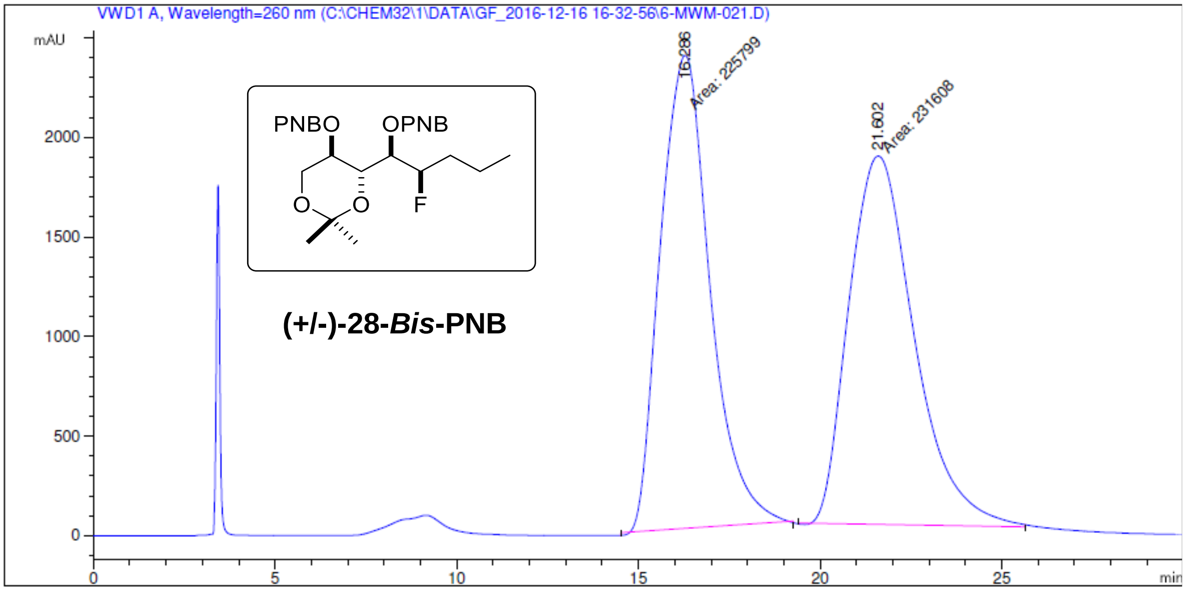


| Peak # | RetTime [min] | Type | Width [min] | Area mAU *s | Height [mAU] | Area % |
|---|---|---|---|---|---|---|
| 1 | 16.286 | MM | 1.5841 | 2.25799e5 | 2375.75269 | 49.3650 |
| 2 | 21.602 | MM | 2.0865 | 2.31608e5 | 1850.00977 | 50.6350 |

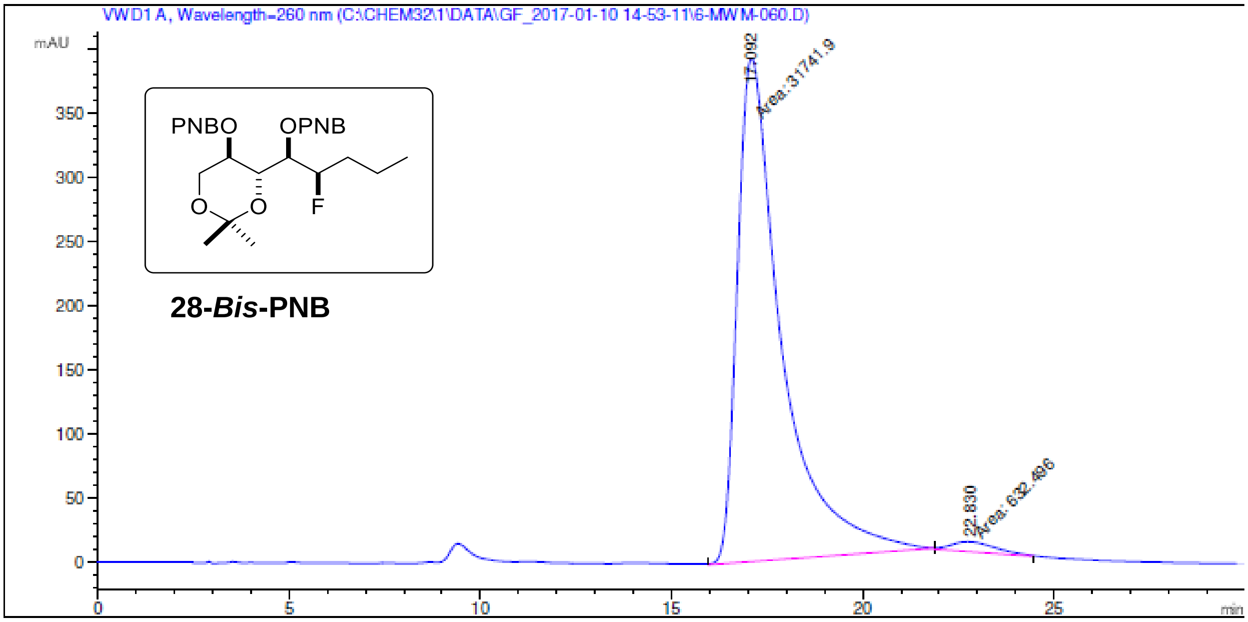


| Peak # | RetTime [min] | Type | Width [min] | Area mAU *s | Height [mAU] | Area % |
|---|---|---|---|---|---|---|
| 1 | 17.092 | MM | 1.3494 | 3.17419e4 | 392.04599 | 98.0463 |
| 2 | 22.830 | MM | 1.3567 | 632.49640 | 7.76991 | 1.9537 |

**Supplementary Figure 1**. Determination of enantiomeric excess of **28**.

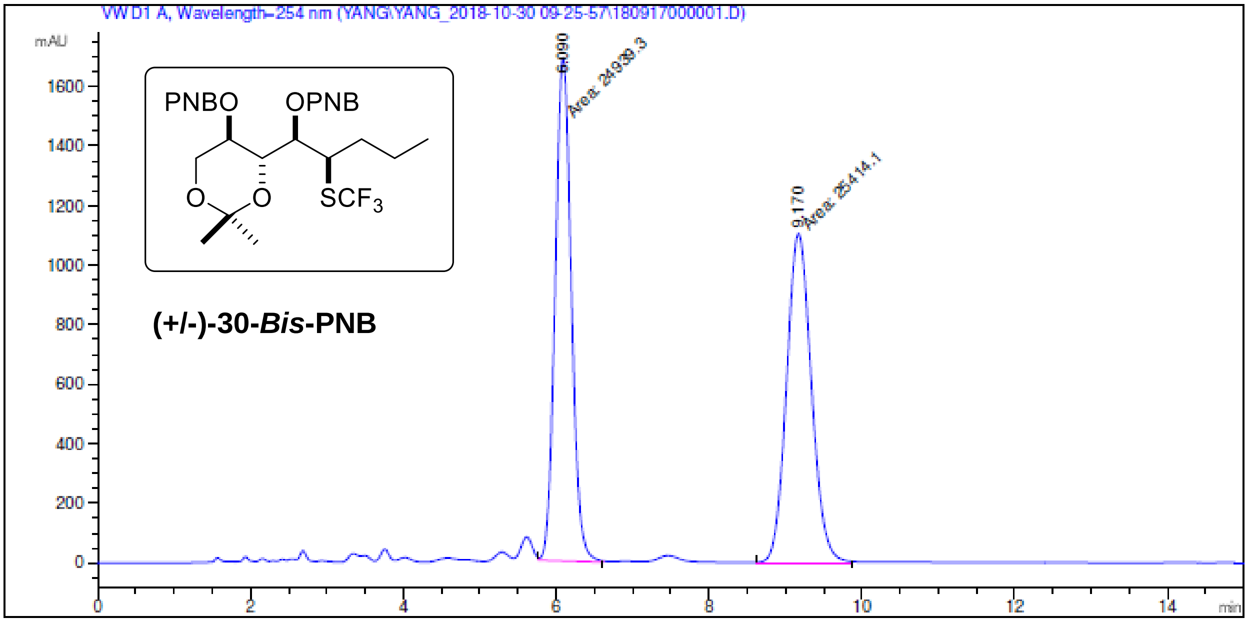


| Peak # | RetTime [min] | Type | Width [min] | Area mAU *s | Height [mAU ] | Area % |
|---|---|---|---|---|---|---|
| 1 | 6.090 | MM | 0.2459 | 2.49393e4 | 1690.62378 | 49.5286 |
| 2 | 9.170 | MM | 0.3815 | 2.54141e4 | 1110.22144 | 50.4714 |

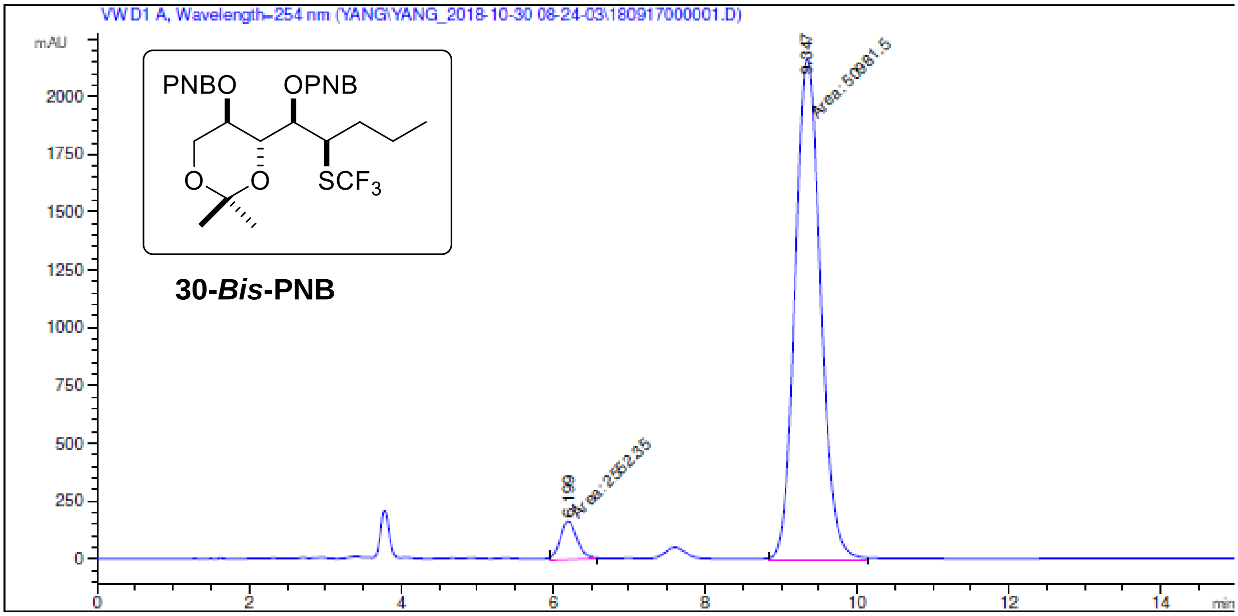


| Peak # | RetTime [min] | Type | Width [min] | Area mAU *s | Height [mAU ] | Area % |
|---|---|---|---|---|---|---|
| 1 | 6.199 | MM | 0.2574 | 2552.35303 | 165.24725 | 4.7677 |
| 2 | 9.347 | MM | 0.3910 | 5.09815e4 | 2172.87427 | 95.2323 |

**Supplementary Figure 2**. Determination of enantiomeric excess of **30**.

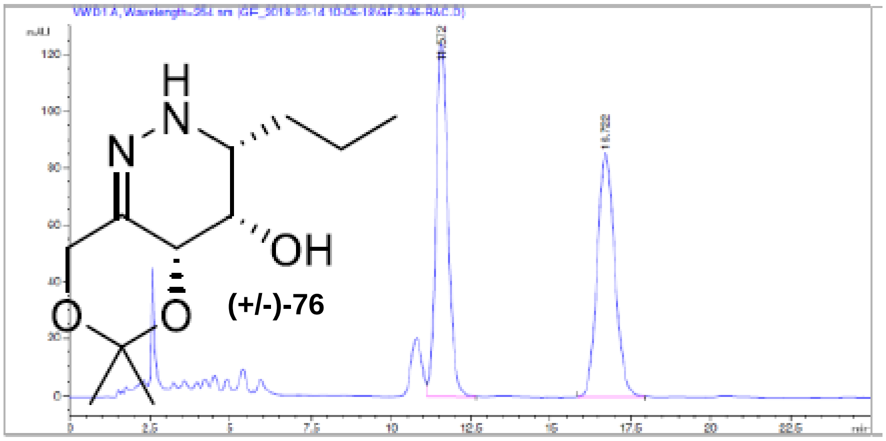


| Peak # | RetTime [min] | Type | Width [min] | Area mAU *s | Height [mAU ] | Area % |
|---|---|---|---|---|---|---|
| 1 | 11.572 | VB | 0.3993 | 3222.68481 | 123.99079 | 49.7858 |
| 2 | 16.722 | BB | 0.6037 | 3250.41846 | 85.20957 | 50.2142 |

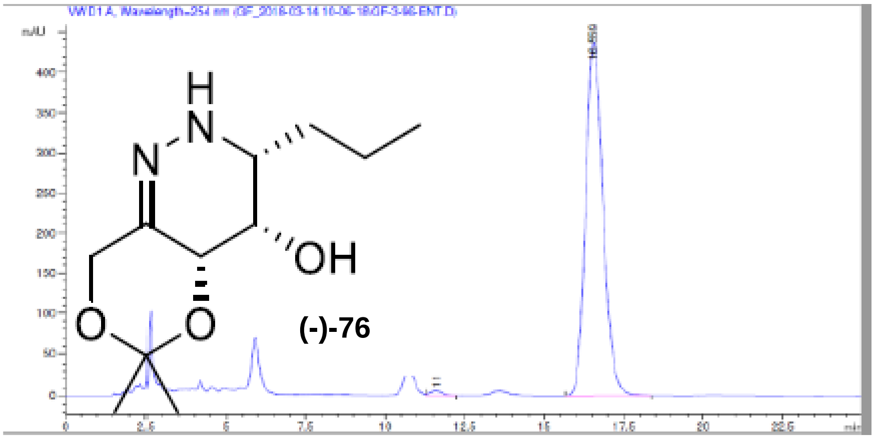


| Peak # | RetTime [min] | Type | Width [min] | Area mAU *s | Height [mAU ] | Area % |
|---|---|---|---|---|---|---|
| 1 | 11.596 | VB | 0.4016 | 154.58374 | 5.84761 | 0.9159 |
| 2 | 16.559 | BB | 0.5943 | 1.67232e4 | 436.26291 | 99.0841 |

**Supplementary Figure 3**. Determination of enantiomeric excess of **76**.

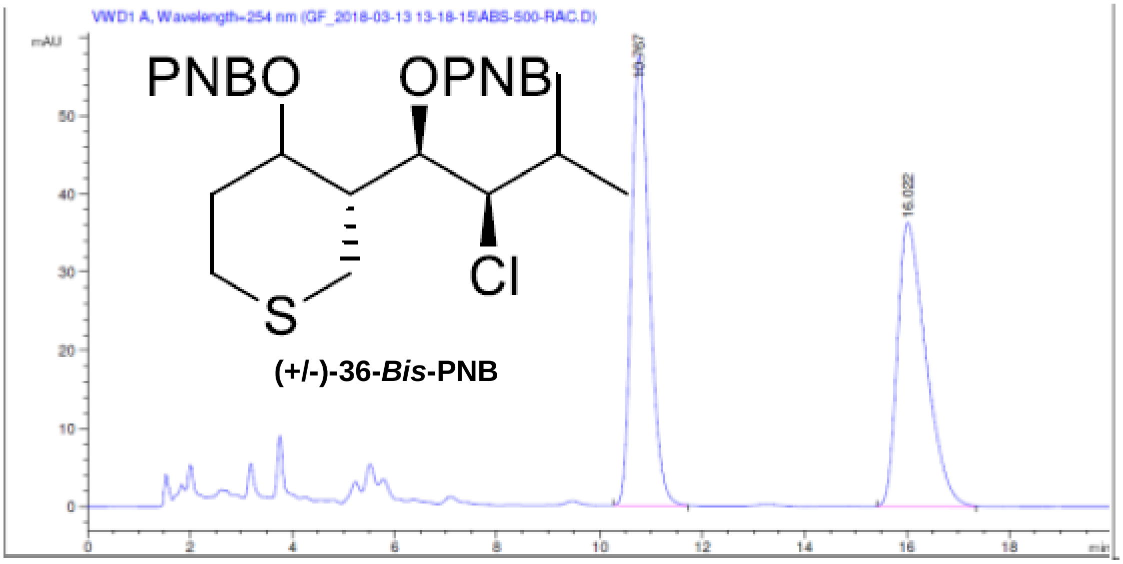


| Peak # | RetTime [min] | Type | Width [min] | Area mAU *s | Height [mAU ] | Area % |
|---|---|---|---|---|---|---|
| 1 | 10.767 | BB | 0.3784 | 1406.09375 | 57.52106 | 50.0987 |
| 2 | 16.022 | BB | 0.5694 | 1400.55212 | 36.31229 | 49.9013 |

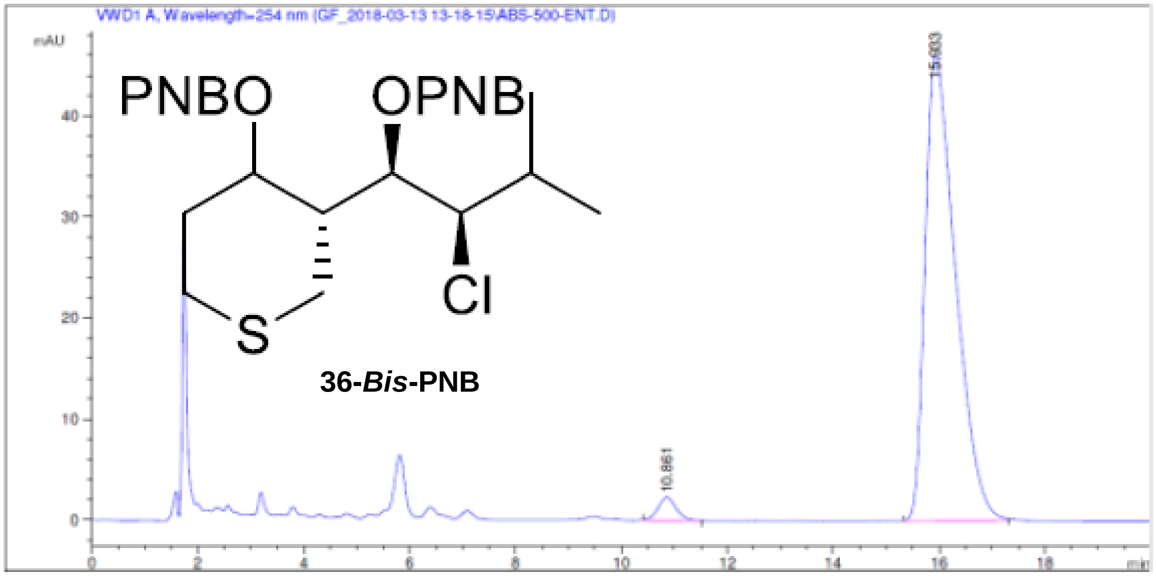


| Peak # | RetTime [min] | Type | Width [min] | Area mAU *s | Height [mAU ] | Area % |
|---|---|---|---|---|---|---|
| 1 | 10.861 | BB | 0.3750 | 54.24590 | 2.26922 | 2.8605 |
| 2 | 15.933 | BB | 0.6066 | 1842.12866 | 46.03395 | 97.1395 |

**Supplementary Figure 4**. Determination of enantiomeric excess of **36**.

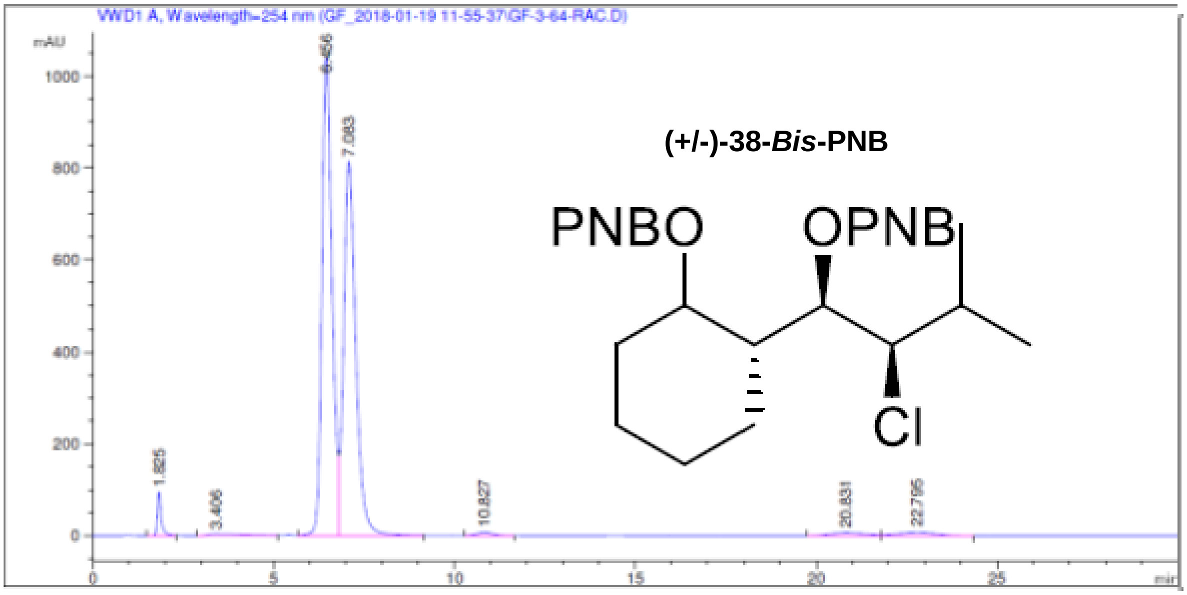


| Peak # | RetTime [min] | Type | Width [min] | Area mAU *s | Height [mAU ] | Area % |
|---|---|---|---|---|---|---|
| 1 | 1.825 | BV | 0.1105 | 715.49103 | 95.71639 | 1.7298 |
| 2 | 3.406 | BB | 0.8502 | 254.80144 | 3.87467 | 0.6160 |
| 3 | 6.456 | VV | 0.2971 | 2.02611e4 | 1040.47473 | 48.9826 |
| 4 | 7.083 | VB | 0.3514 | 1.90217e4 | 813.52588 | 45.9864 |

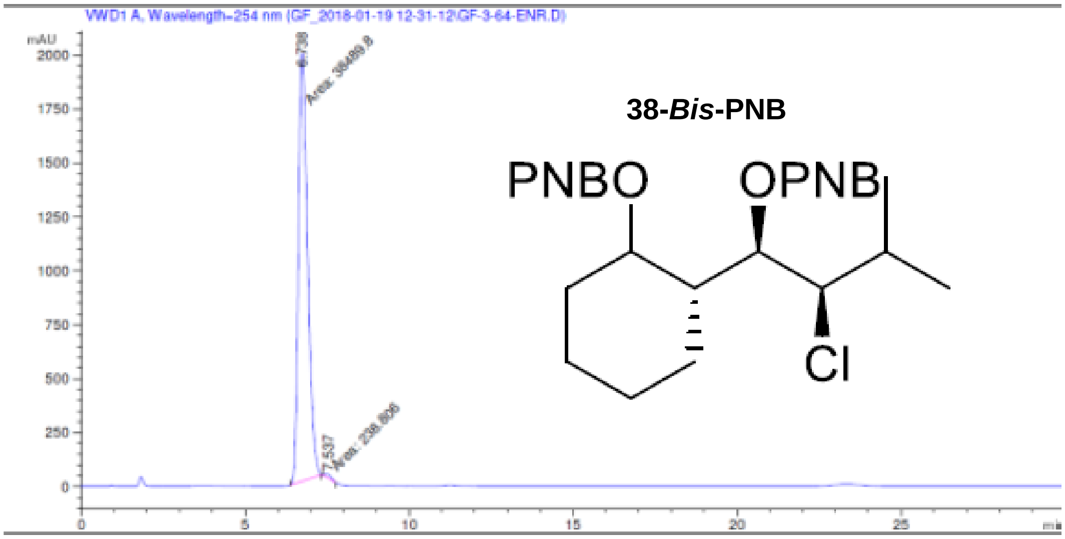


| Peak # | RetTime [min] | Type | Width [min] | Area mAU *s | Height [mAU ] | Area % |
|---|---|---|---|---|---|---|
| 1 | 6.738 | MM | 0.3231 | 3.84898e4 | 1985.21973 | 99.3834 |
| 2 | 7.537 | MM | 0.2245 | 238.80635 | 17.72810 | 0.6166 |

**Supplementary Figure 5**. Determination of enantiomeric excess of **38**.

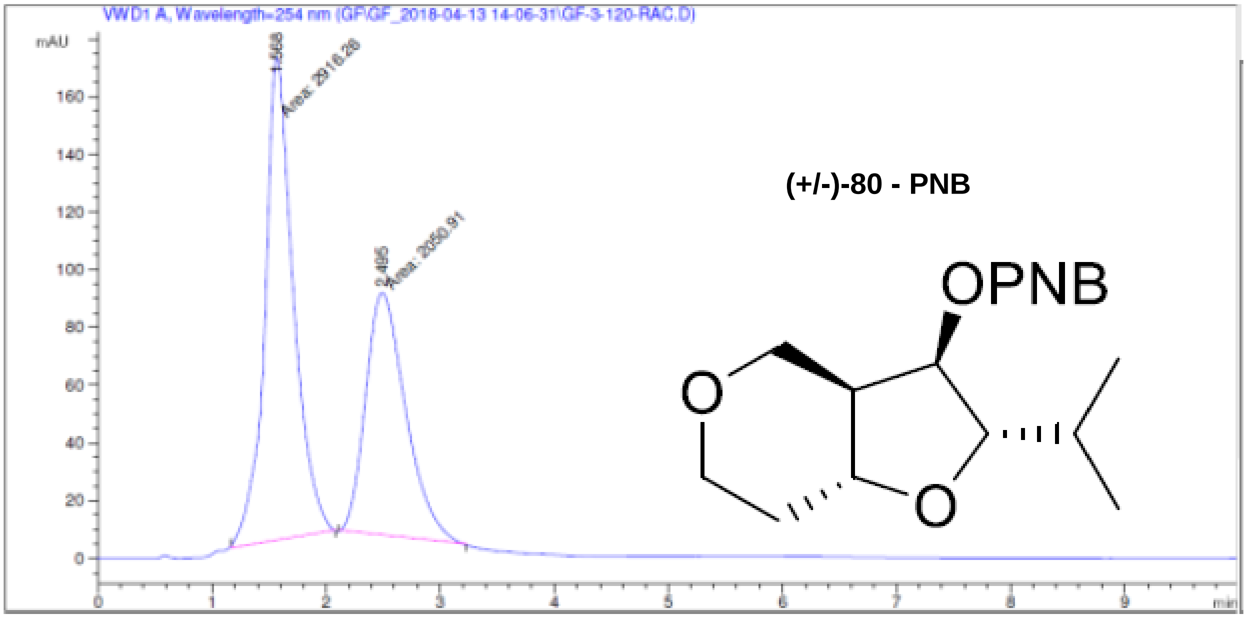


| Peak # | RetTime [min] | Type | Width [min] | Area mAU *s | Height [mAU ] | Area % |
|---|---|---|---|---|---|---|
| 1 | 1.568 | MM | 0.2907 | 2916.25854 | 167.17921 | 58.7107 |
| 2 | 2.495 | MM | 0.4077 | 2050.90723 | 83.83390 | 41.2893 |

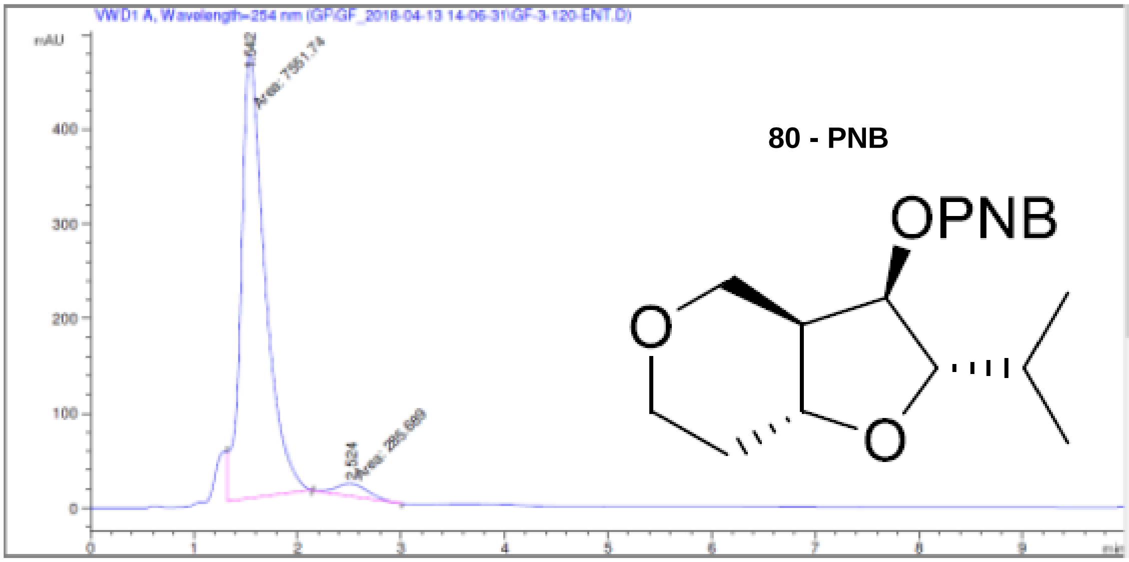


| Peak # | RetTime [min] | Type | Width [min] | Area mAU *s | Height [mAU ] | Area % |
|---|---|---|---|---|---|---|
| 1 | 1.542 | MM | 0.2686 | 7551.74268 | 468.56067 | 96.3548 |
| 2 | 2.524 | MM | 0.3519 | 285.68860 | 13.53053 | 3.6452 |

**Supplementary Figure 6**. Determination of enantiomeric excess of **80**.

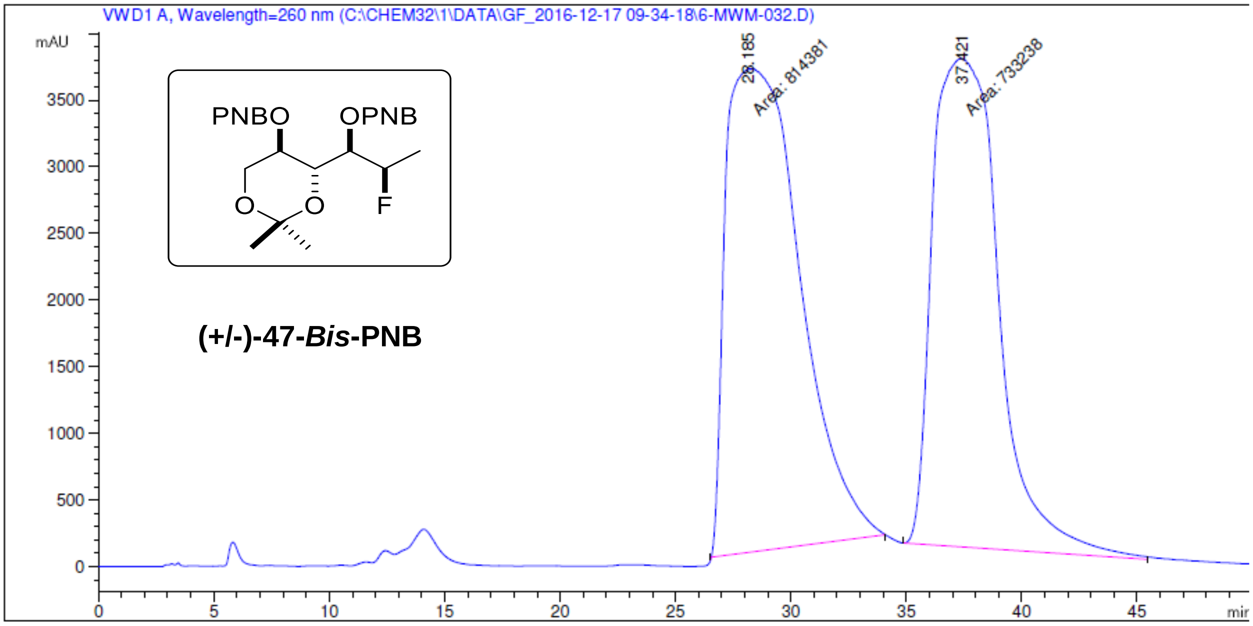


| Peak # | RetTime [min] | Type | Width [min] | Area mAU *s | Height [mAU] | Area % |
|---|---|---|---|---|---|---|
| 1 | 28.185 | MM | 3.7371 | 8.14381e5 | 3631.99609 | 52.6215 |
| 2 | 37.421 | MM | 3.3336 | 7.33238e5 | 3665.93579 | 47.3785 |

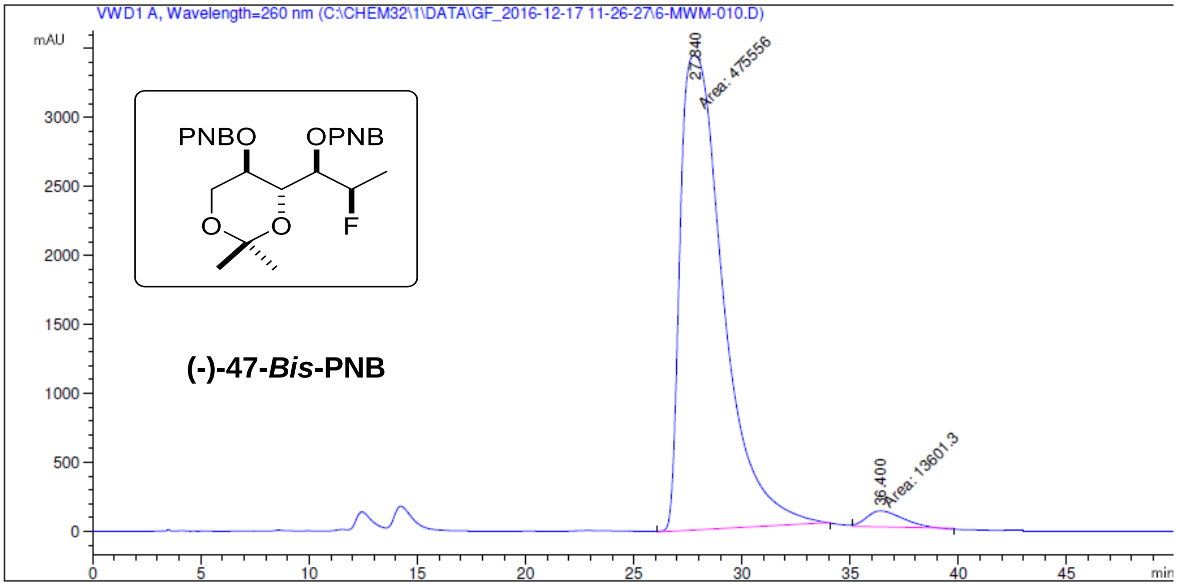


| Peak # | RetTime [min] | Type | Width [min] | Area mAU *s | Height [mAU] | Area % |
|---|---|---|---|---|---|---|
| 1 | 27.840 | MM | 2.3335 | 4.82563e5 | 3446.58838 | 97.2990 |
| 2 | 36.405 | MM | 1.9430 | 1.33957e4 | 114.90332 | 2.7010 |

**Supplementary Figure 7**. Determination of enantiomeric excess of **47**.

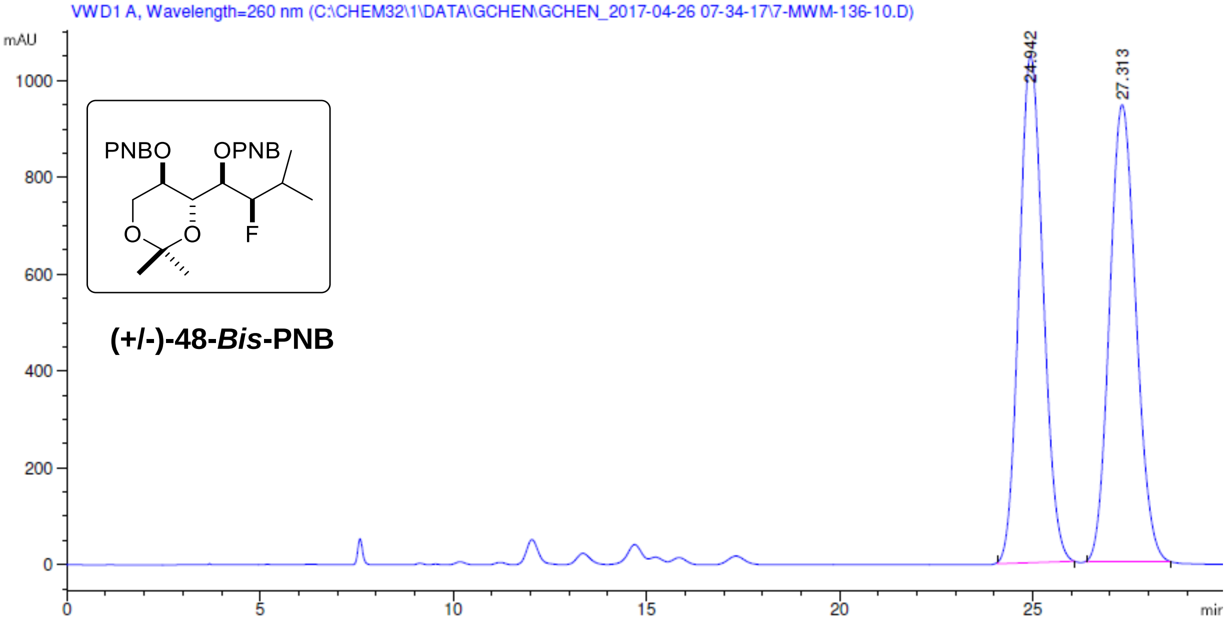


| Peak # | RetTime [min] | Type | Width [min] | Area mAU *s | Height [mAU] | Area % |
|---|---|---|---|---|---|---|
| 1 | 24.942 | BB | 0.6736 | 4.51681e4 | 1047.10242 | 50.1981 |
| 2 | 27.313 | BB | 0.7400 | 4.48115e4 | 944.25000 | 49.8019 |

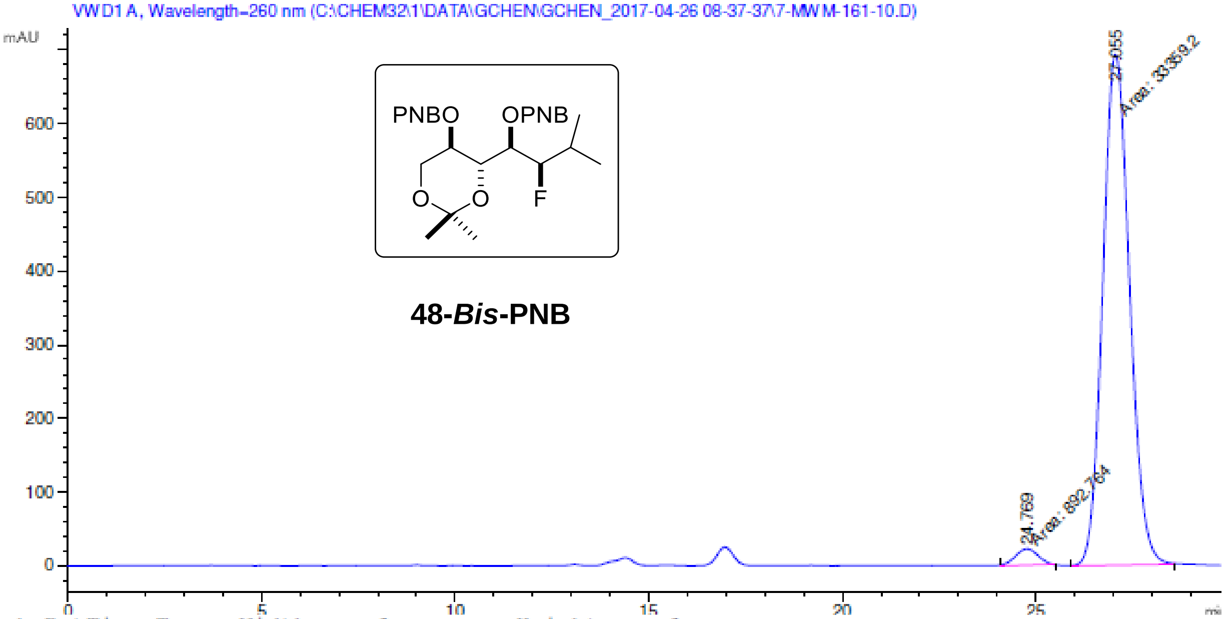


| Peak # | RetTime [min] | Type | Width [min] | Area mAU *s | Height [mAU] | Area % |
|---|---|---|---|---|---|---|
| 1 | 24.769 | MM | 0.6788 | 892.76361 | 21.91918 | 2.6065 |
| 2 | 27.055 | MM | 0.8022 | 3.33592e4 | 693.12097 | 97.3935 |

**Supplementary Figure 8**. Determination of enantiomeric excess of **48**.

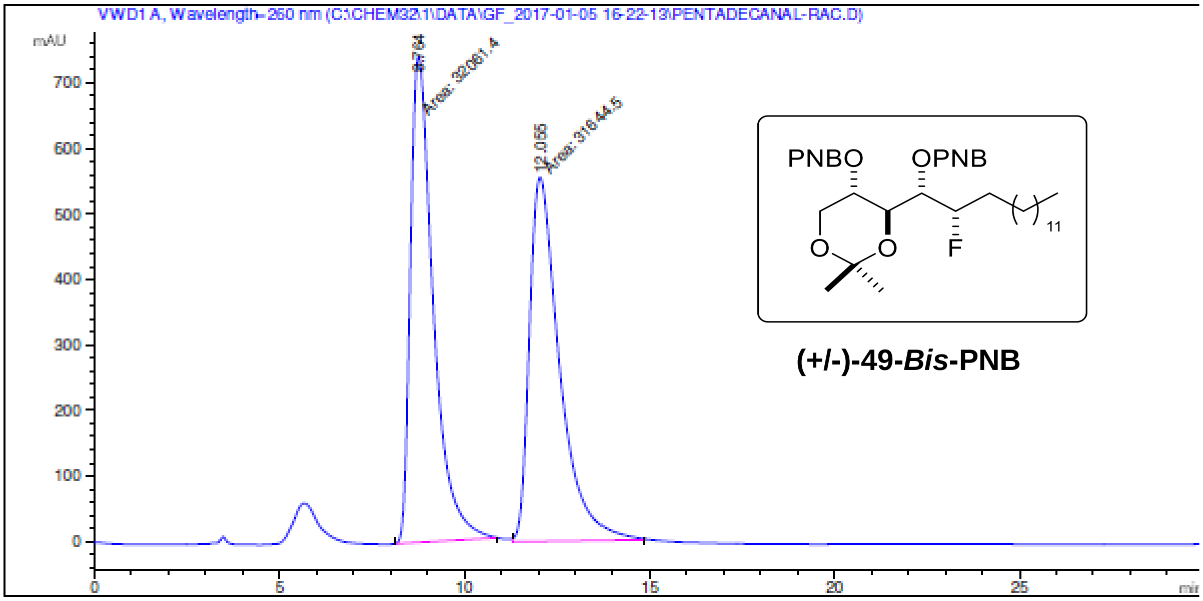


| Peak # | RetTime [min] | Type | Width [min] | Area mAU *s | Height [mAU ] | Area % |
|---|---|---|---|---|---|---|
| 1 | 8.764 | MM | 0.7202 | 3.20614e4 | 741.96558 | 50.3272 |
| 2 | 12.055 | MM | 0.9481 | 3.16445e4 | 556.26318 | 49.6728 |

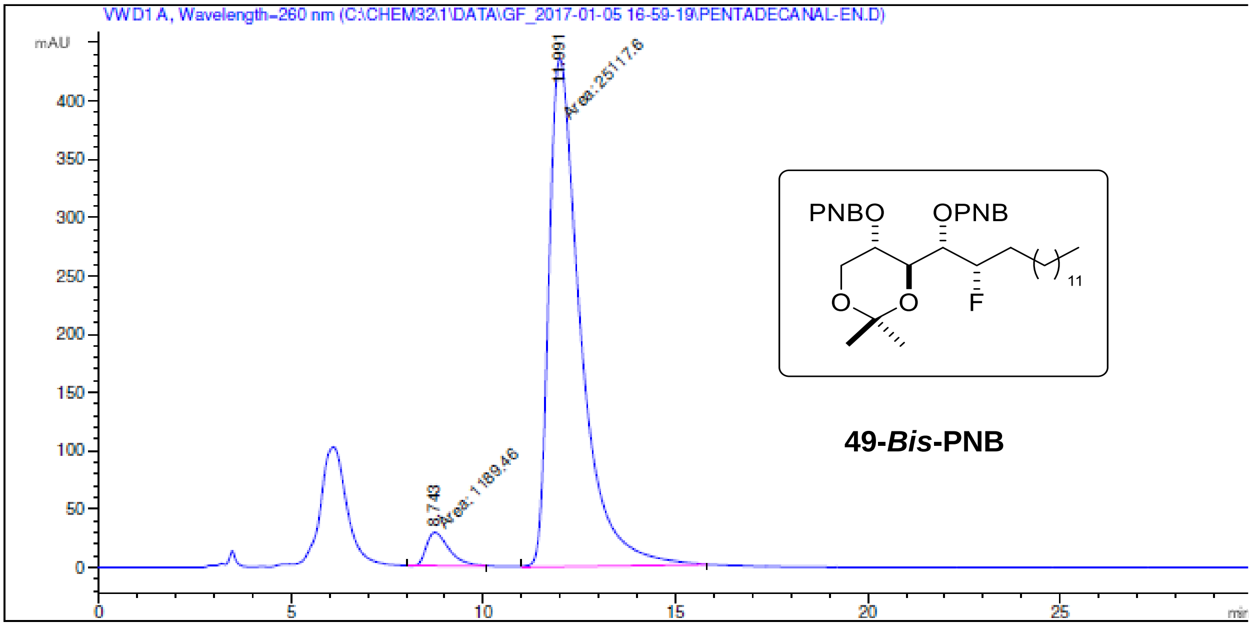


| Peak # | RetTime [min] | Type | Width [min] | Area mAU *s | Height [mAU ] | Area % |
|---|---|---|---|---|---|---|
| 1 | 8.743 | MM | 0.6916 | 1189.46448 | 28.66516 | 4.5215 |
| 2 | 11.991 | MM | 0.9600 | 2.51176e4 | 436.05441 | 95.4785 |

**Supplementary Figure 9**. Determination of enantiomeric excess of **49**.

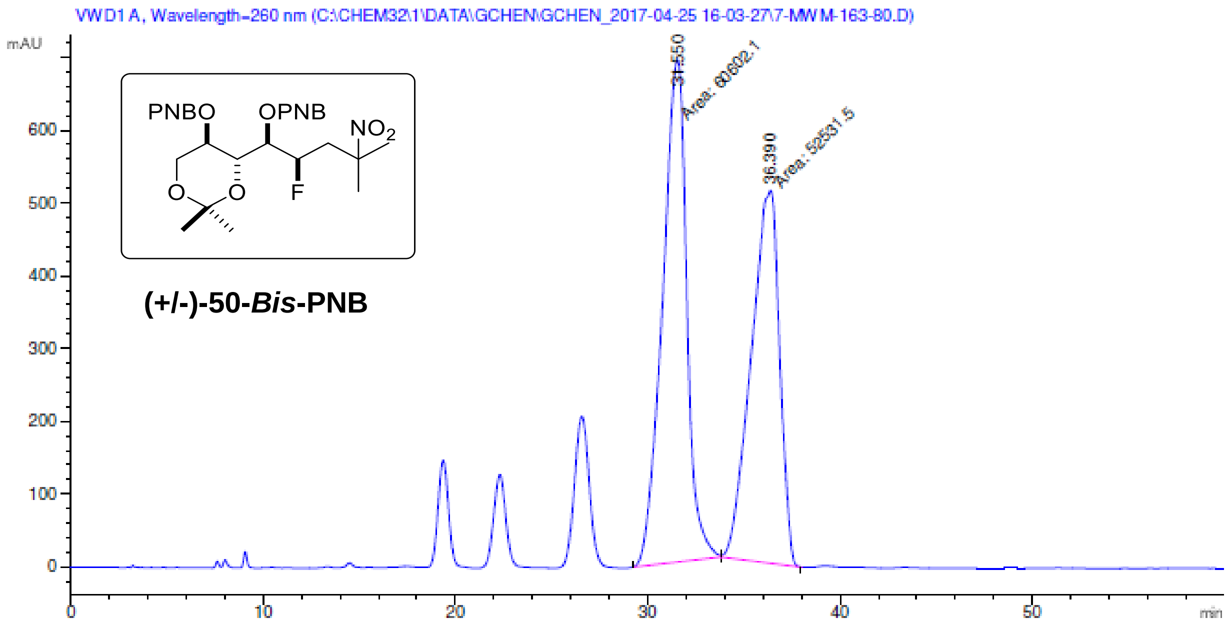


| Peak # | RetTime [min] | Type | Width [min] | Area mAU *s | Height [mAU ] | Area % |
|---|---|---|---|---|---|---|
| 1 | 31.550 | MM | 1.4641 | 6.06021e4 | 689.87817 | 53.5668 |
| 2 | 36.390 | MM | 1.7093 | 5.25315e4 | 512.21033 | 46.4332 |

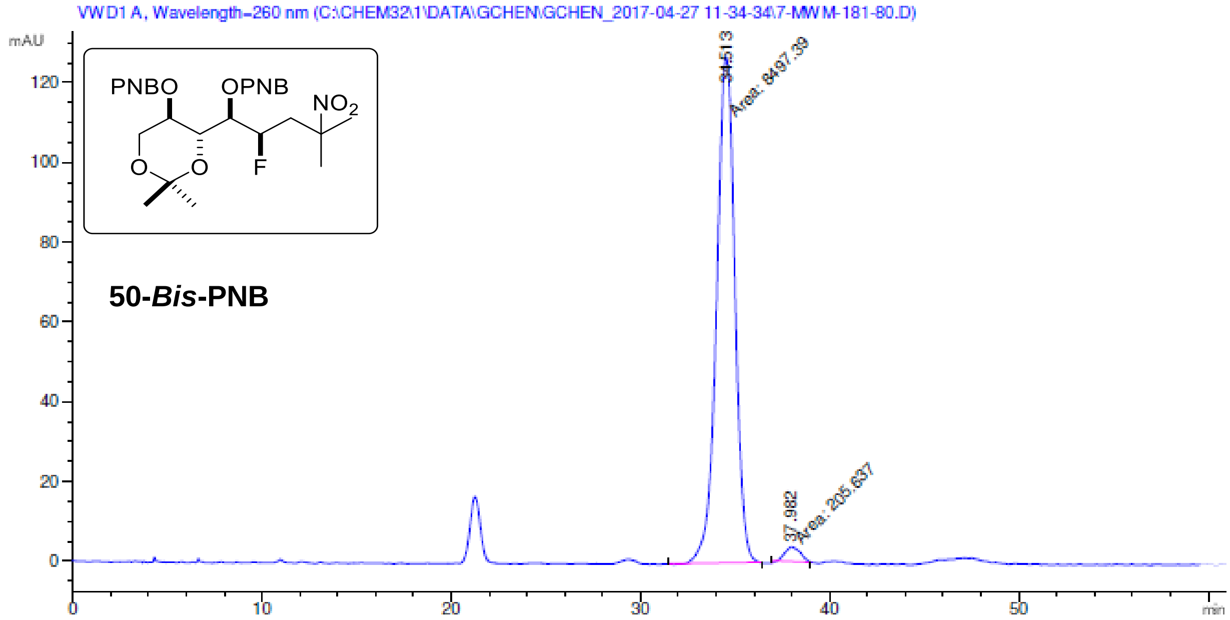


| Peak # | RetTime [min] | Type | Width [min] | Area mAU *s | Height [mAU ] | Area % |
|---|---|---|---|---|---|---|
| 1 | 34.513 | MM | 1.1163 | 8497.39453 | 126.87410 | 97.6372 |
| 2 | 37.982 | MM | 0.9360 | 205.63655 | 3.66148 | 2.3628 |

**Supplementary Figure 10**. Determination of enantiomeric excess of **50**.

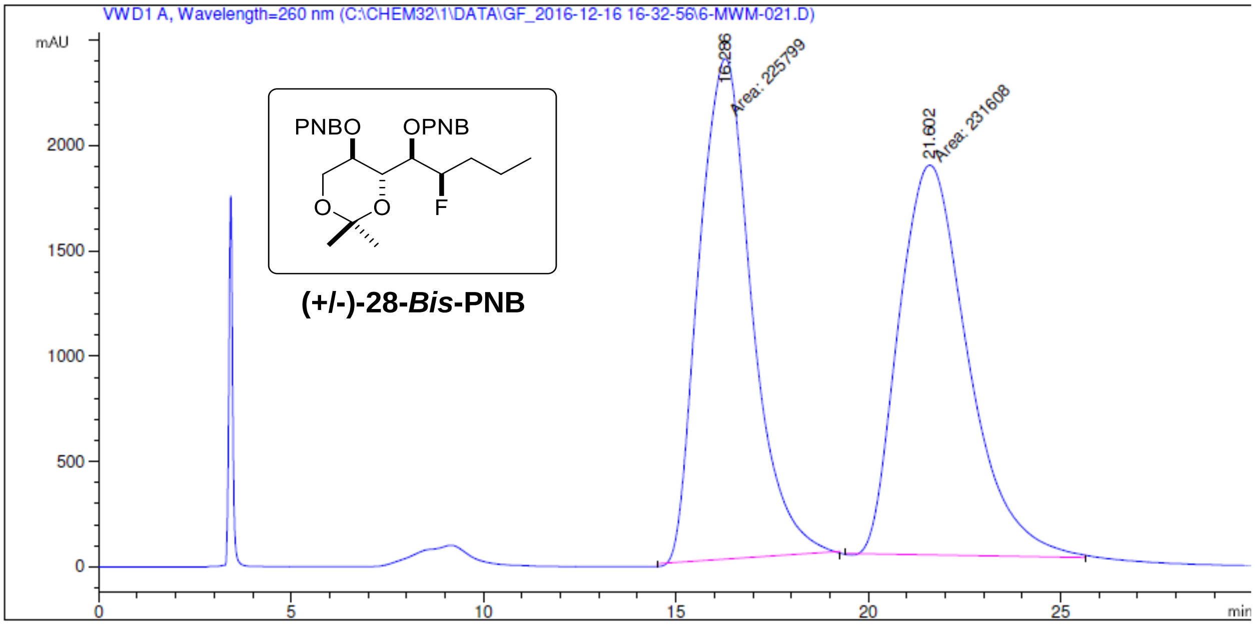


| Peak # | RetTime [min] | Type | Width [min] | Area mAU *s | Height [mAU ] | Area % |
|---|---|---|---|---|---|---|
| 1 | 16.286 | MM | 1.5841 | 2.25799e5 | 2375.75269 | 49.3650 |
| 2 | 21.602 | MM | 2.0865 | 2.31608e5 | 1850.00977 | 50.6350 |

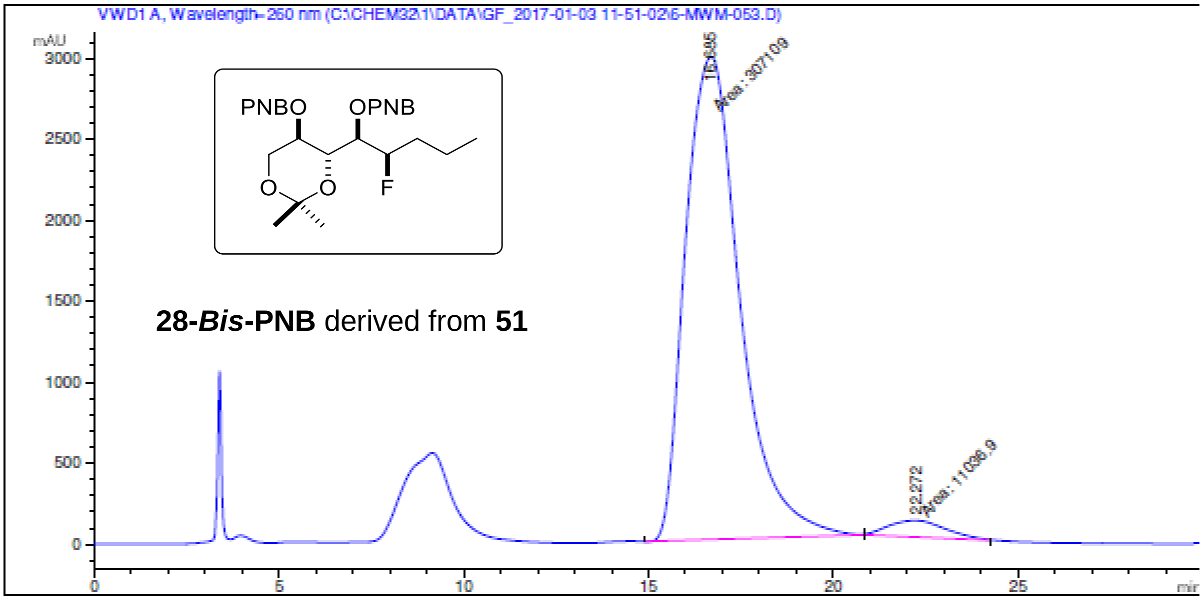


| Peak # | RetTime [min] | Type | Width [min] | Area mAU *s | Height [mAU ] | Area % |
|---|---|---|---|---|---|---|
| 1 | 16.685 | MM | 1.7169 | 3.07109e5 | 2981.22705 | 96.5309 |
| 2 | 22.272 | MM | 1.7879 | 1.10369e4 | 102.88731 | 3.4691 |

**Supplementary Figure 11**. Determination of enantiomeric excess of **51**.

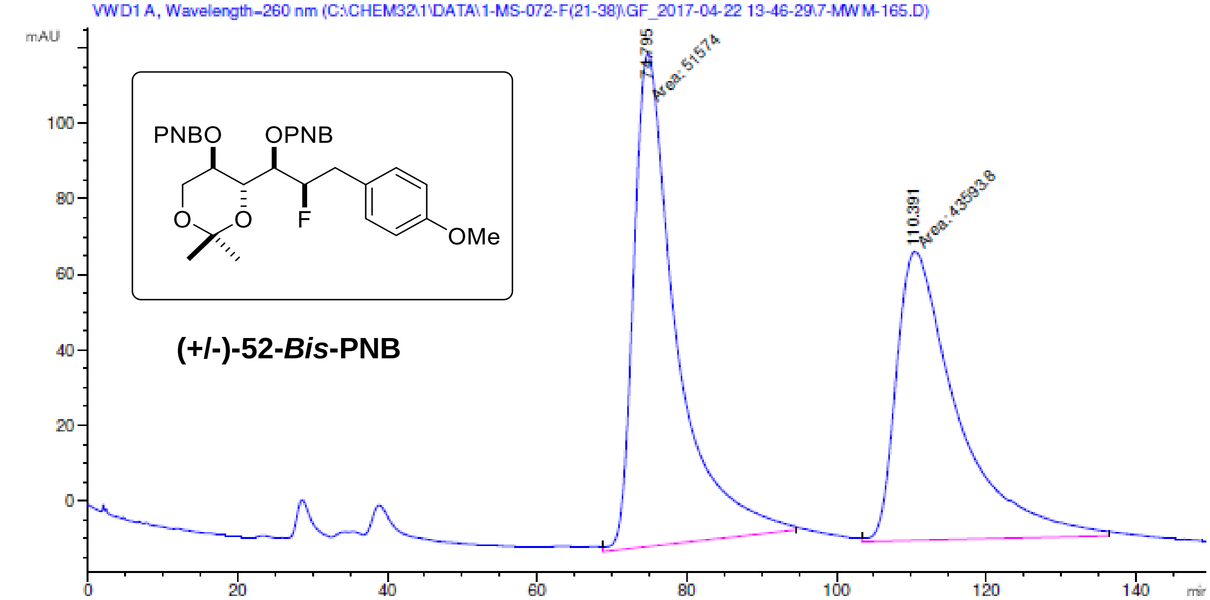


| Peak # | RetTime [min] | Type | Width [min] | Area mAU *s | Height [mAU ] | Area % |
|---|---|---|---|---|---|---|
| 1 | 74.795 | MM | 6.5718 | 5.15740e4 | 130.79599 | 54.1927 |
| 2 | 110.391 | MM | 9.4860 | 4.35938e4 | 76.59305 | 45.8073 |

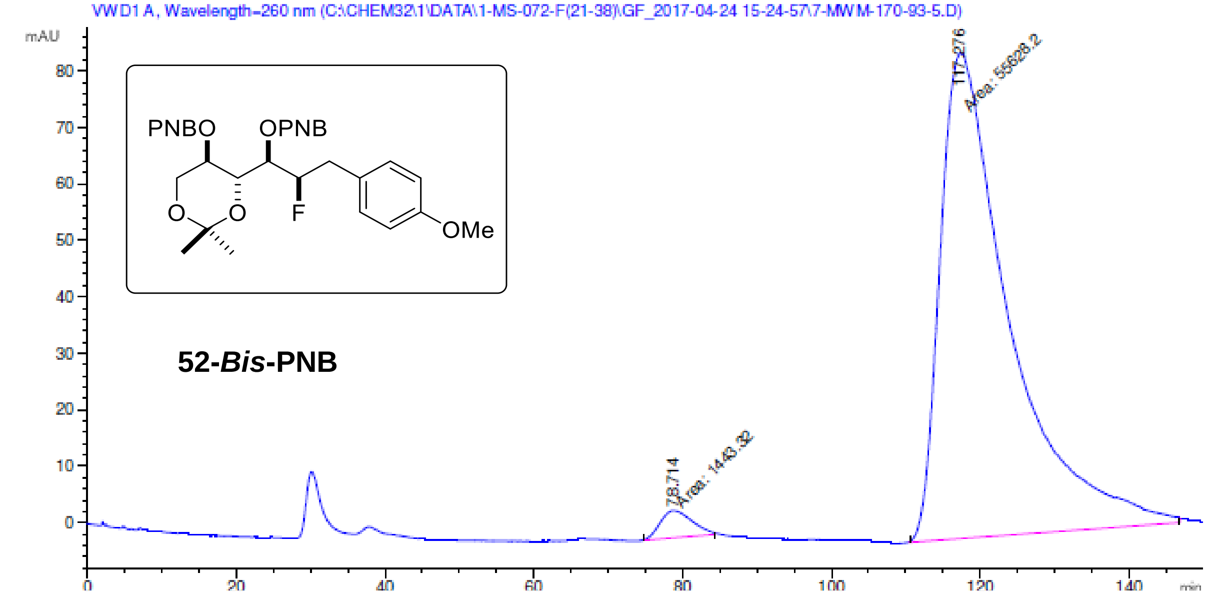


| Peak # | RetTime [min] | Type | Width [min] | Area mAU *s | Height [mAU ] | Area % |
|---|---|---|---|---|---|---|
| 1 | 78.714 | MM | 4.9697 | 1443.32019 | 4.84036 | 2.5290 |
| 2 | 117.276 | MM | 10.7686 | 5.56282e4 | 86.09621 | 97.4710 |

**Supplementary Figure 12**. Determination of enantiomeric excess of **52**.

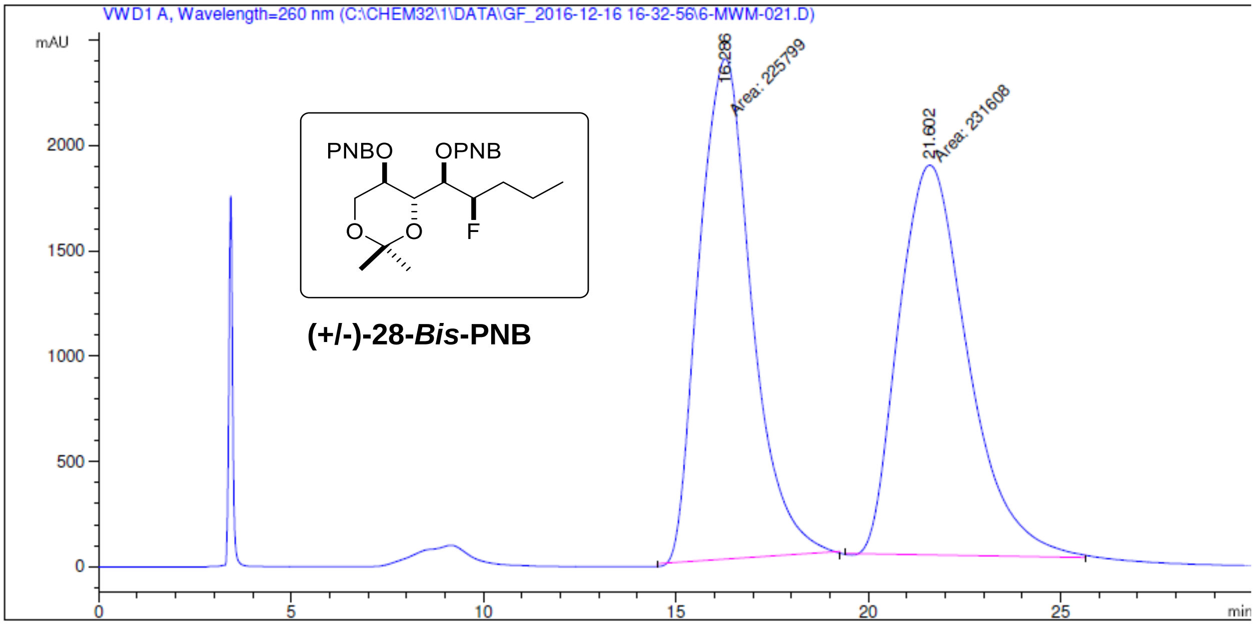


| Peak # | RetTime [min] | Type | Width [min] | Area mAU *s | Height [mAU ] | Area % |
|---|---|---|---|---|---|---|
| 1 | 16.286 | MM | 1.5841 | 2.25799e5 | 2375.75269 | 49.3650 |
| 2 | 21.602 | MM | 2.0865 | 2.31608e5 | 1850.00977 | 50.6350 |

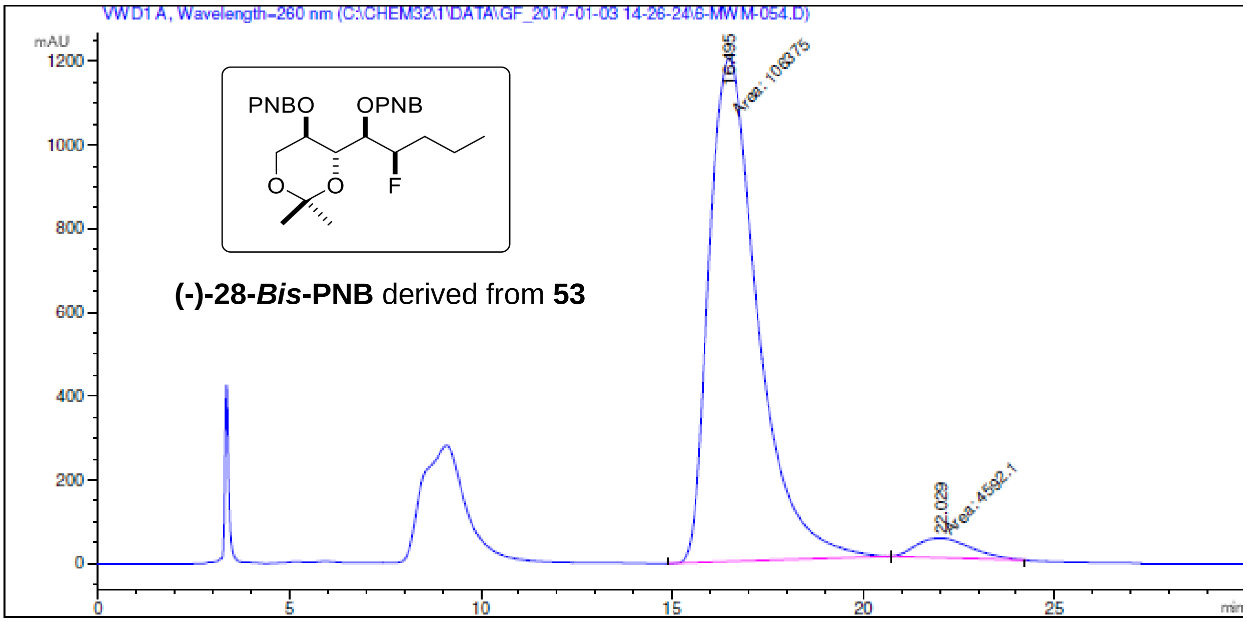


| Peak # | RetTime [min] | Type | Width [min] | Area mAU *s | Height [mAU ] | Area % |
|---|---|---|---|---|---|---|
| 1 | 16.495 | MM | 1.4751 | 1.06375e5 | 1201.89160 | 95.8617 |
| 2 | 22.029 | MM | 1.6457 | 4592.09717 | 46.50721 | 4.1383 |

**Supplementary Figure 13**. Determination of enantiomeric excess of **53**.

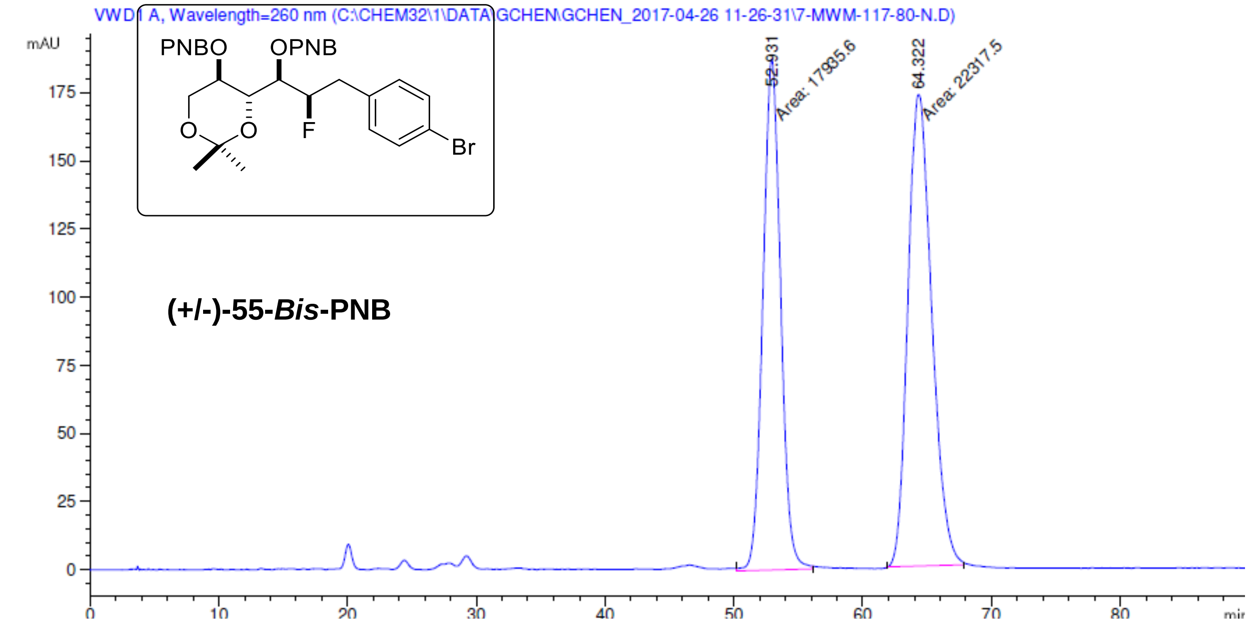


| Peak # | RetTime [min] | Type | Width [min] | Area mAU *s | Height [mAU] | Area % |
|---|---|---|---|---|---|---|
| 1 | 52.931 | MM | 1.5954 | 1.79356e4 | 187.36581 | 44.5571 |
| 2 | 64.322 | MM | 2.1503 | 2.23175e4 | 172.98358 | 55.4429 |

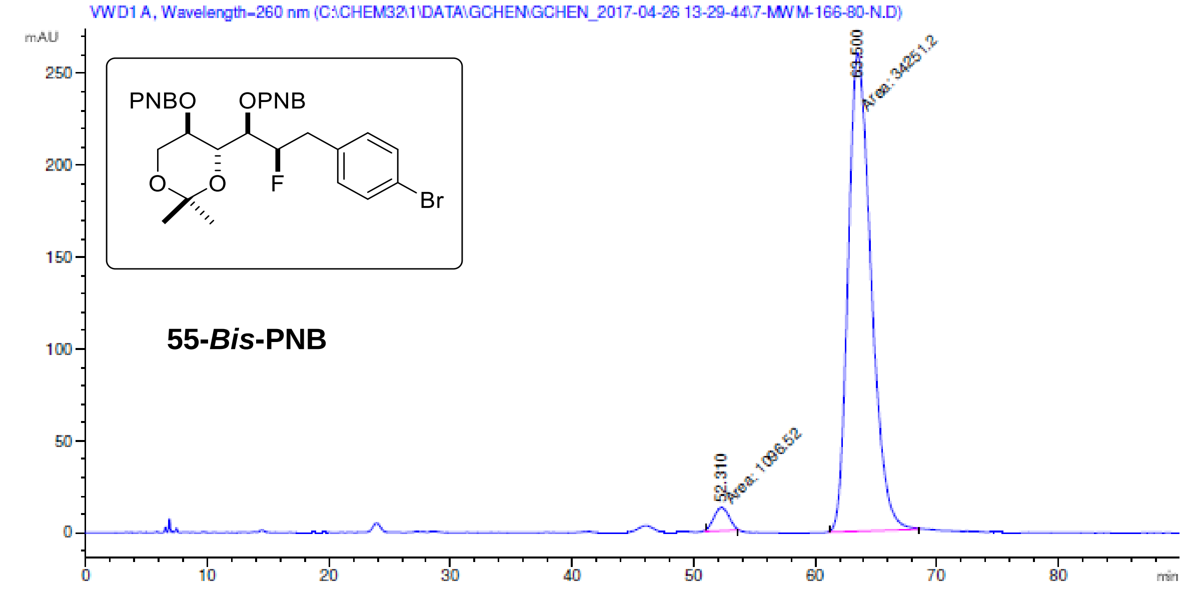


| Peak # | RetTime [min] | Type | Width [min] | Area mAU *s | Height [mAU] | Area % |
|---|---|---|---|---|---|---|
| 1 | 52.310 | MM | 1.4265 | 1096.51550 | 12.81092 | 3.1021 |
| 2 | 63.500 | MM | 2.1938 | 3.42512e4 | 260.21017 | 96.8979 |

**Supplementary Figure 14**. Determination of enantiomeric excess of **55**.

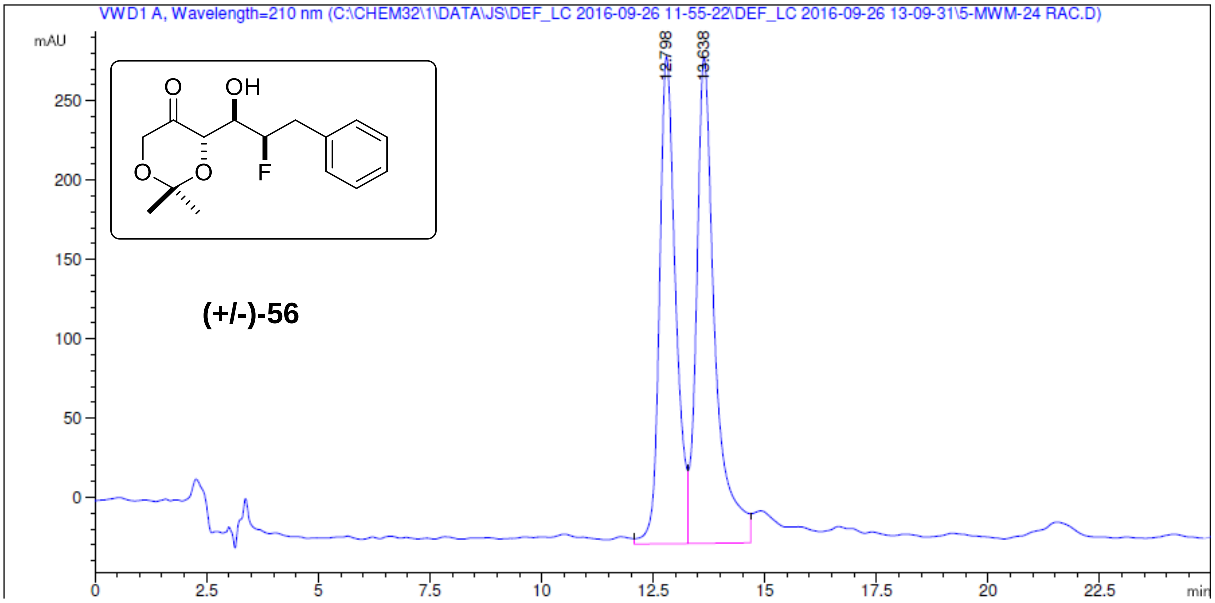


| Peak # | RetTime [min] | Type | Width [min] | Area mAU *s | Height [mAU ] | Area % |
|---|---|---|---|---|---|---|
| 1 | 12.798 | VV | 0.3747 | 7717.89990 | 307.11288 | 46.8503 |
| 2 | 13.638 | VV | 0.4151 | 8755.62305 | 306.19824 | 53.1497 |

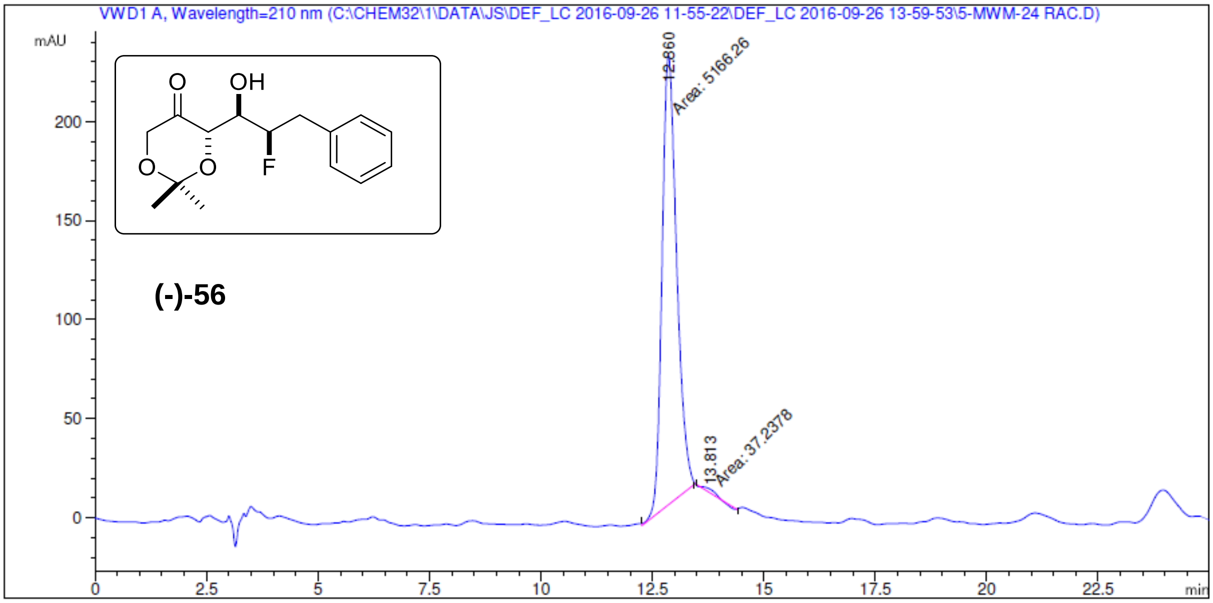


| Peak # | RetTime [min] | Type | Width [min] | Area mAU *s | Height [mAU ] | Area % |
|---|---|---|---|---|---|---|
| 1 | 12.860 | MM | 0.3800 | 5166.26025 | 226.59462 | 99.2844 |
| 2 | 13.813 | MM | 0.3651 | 37.23775 | 1.97158 | 0.7156 |

**Supplementary Figure 15**. Determination of enantiomeric excess of **56**.

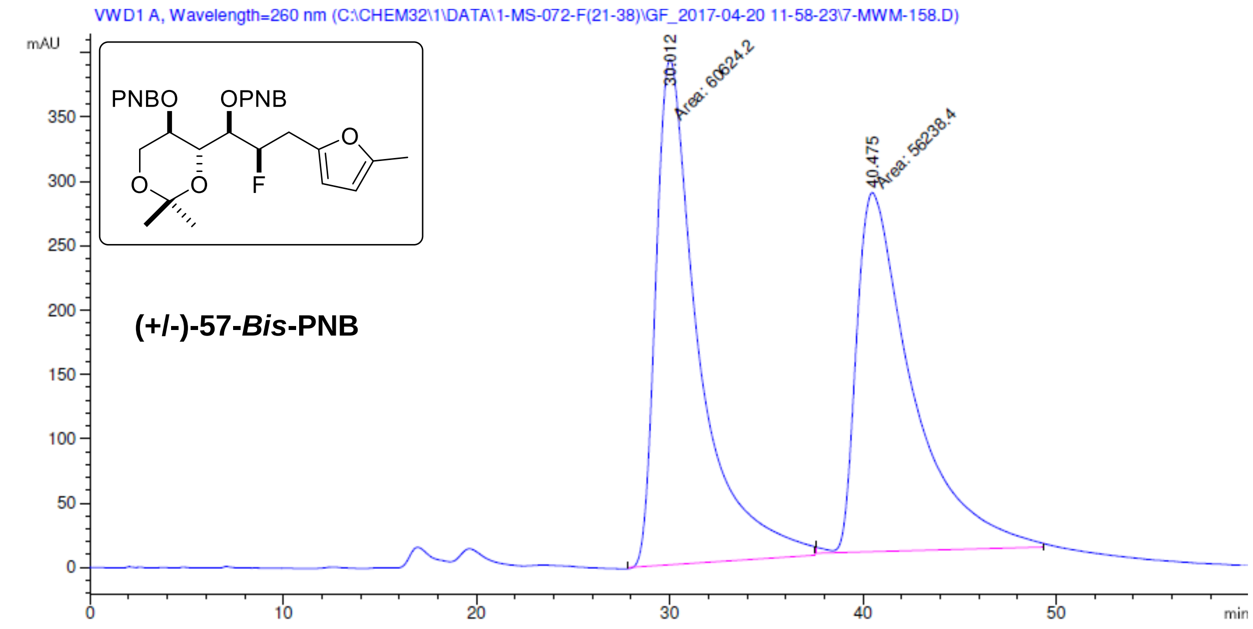


| Peak # | RetTime [min] | Type | Width [min] | Area mAU *s | Height [mAU ] | Area % |
|---|---|---|---|---|---|---|
| 1 | 30.012 | MM | 2.5741 | 6.06242e4 | 392.52161 | 51.8765 |
| 2 | 40.475 | MM | 3.3601 | 5.62384e4 | 278.94772 | 48.1235 |

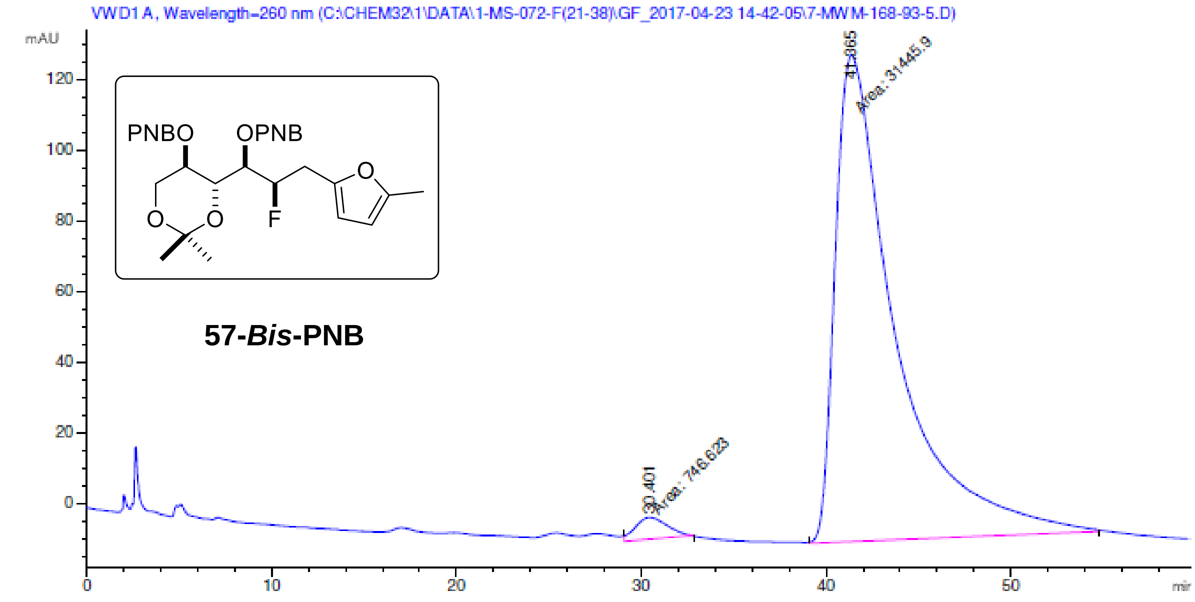


| Peak # | RetTime [min] | Type | Width [min] | Area mAU *s | Height [mAU ] | Area % |
|---|---|---|---|---|---|---|
| 1 | 30.401 | MM | 1.9979 | 746.62323 | 6.22836 | 2.3192 |
| 2 | 41.365 | MM | 3.8032 | 3.14459e4 | 137.80399 | 97.6808 |

**Supplementary Figure 16**. Determination of enantiomeric excess of **57**.

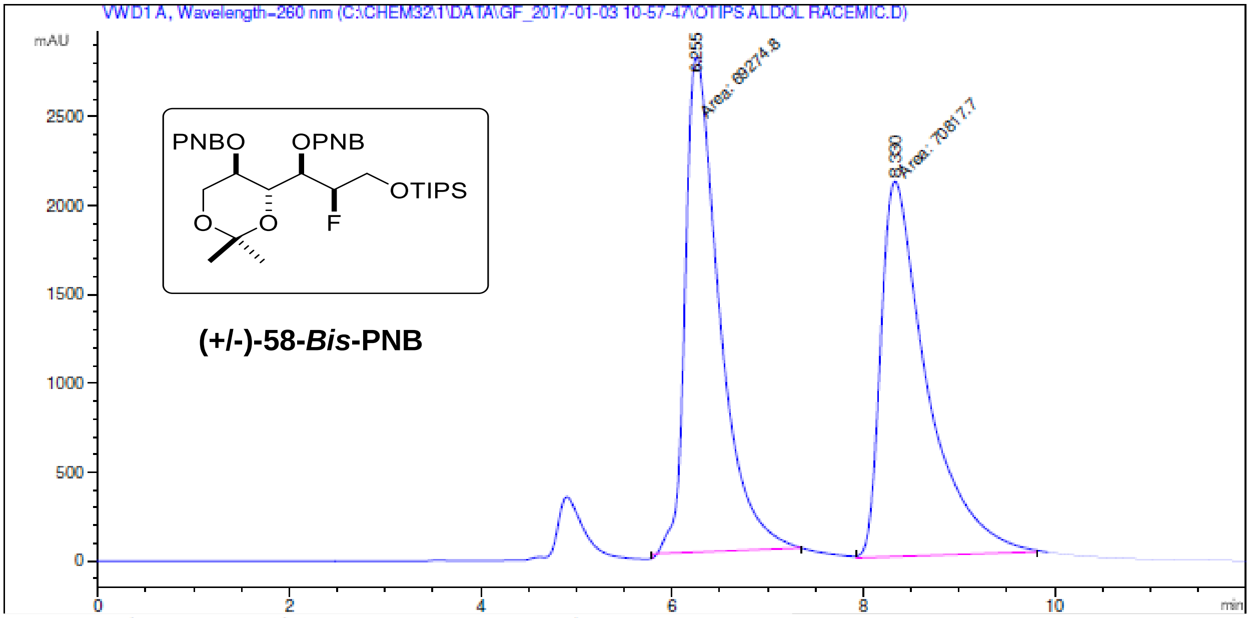


| Peak # | RetTime [min] | Type | Width [min] | Area mAU *s | Height [mAU ] | Area % |
|---|---|---|---|---|---|---|
| 1 | 6.255 | MM | 0.4144 | 6.92748e4 | 2785.93945 | 49.4493 |
| 2 | 8.330 | MM | 0.5587 | 7.08177e4 | 2112.64746 | 50.5507 |

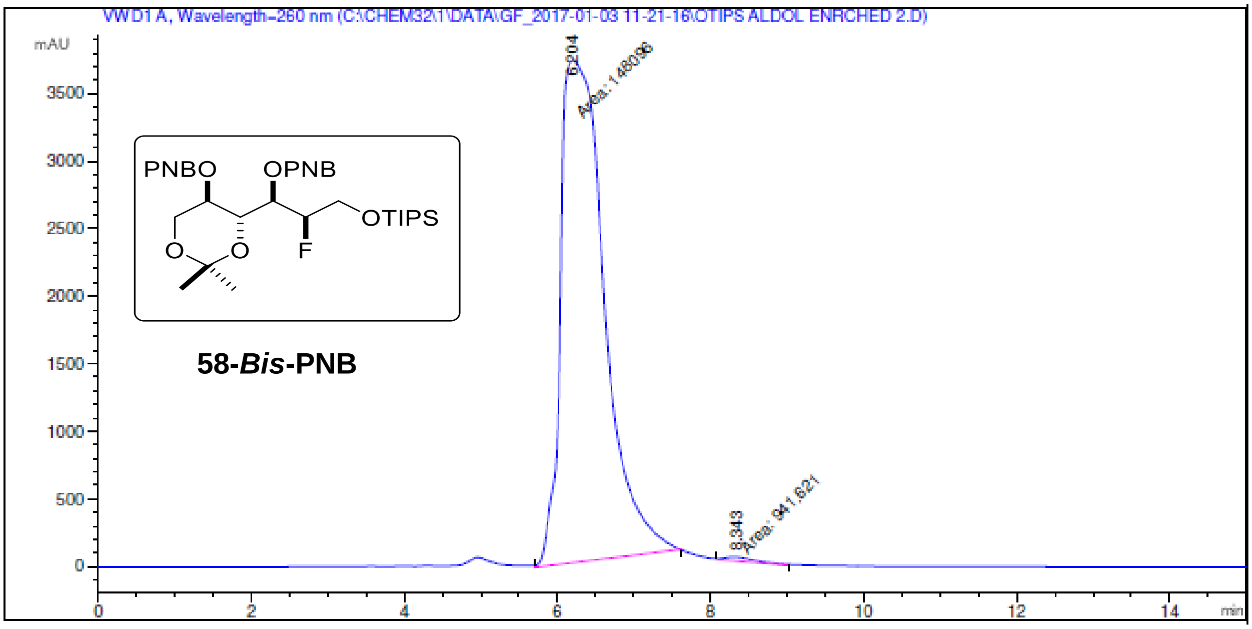


| Peak # | RetTime [min] | Type | Width [min] | Area mAU *s | Height [mAU ] | Area % |
|---|---|---|---|---|---|---|
| 1 | 6.204 | MM | 0.6642 | 1.48096e5 | 3716.41406 | 99.3682 |
| 2 | 8.343 | MM | 0.4924 | 941.62128 | 31.87027 | 0.6318 |

**Supplementary Figure 17**. Determination of enantiomeric excess of **58**.

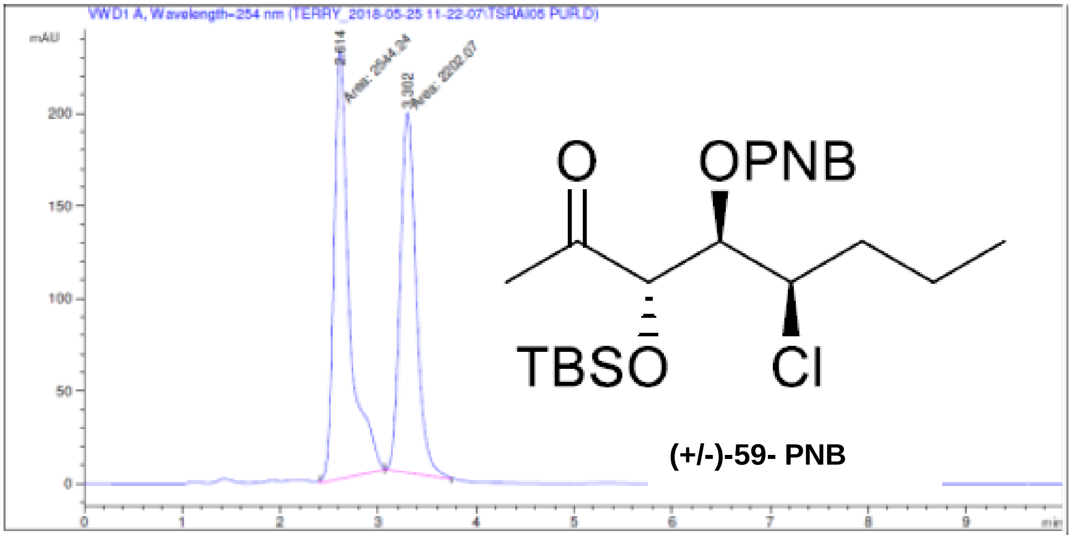


| Peak # | RetTime [min] | Type | Width [min] | Area mAU *s | Height [mAU ] | Area % |
|---|---|---|---|---|---|---|
| 1 | 2.614 | MM | 0.1835 | 2544.23975 | 231.07678 | 53.6046 |
| 2 | 3.302 | MM | 0.1887 | 2202.07080 | 194.44383 | 46.3954 |

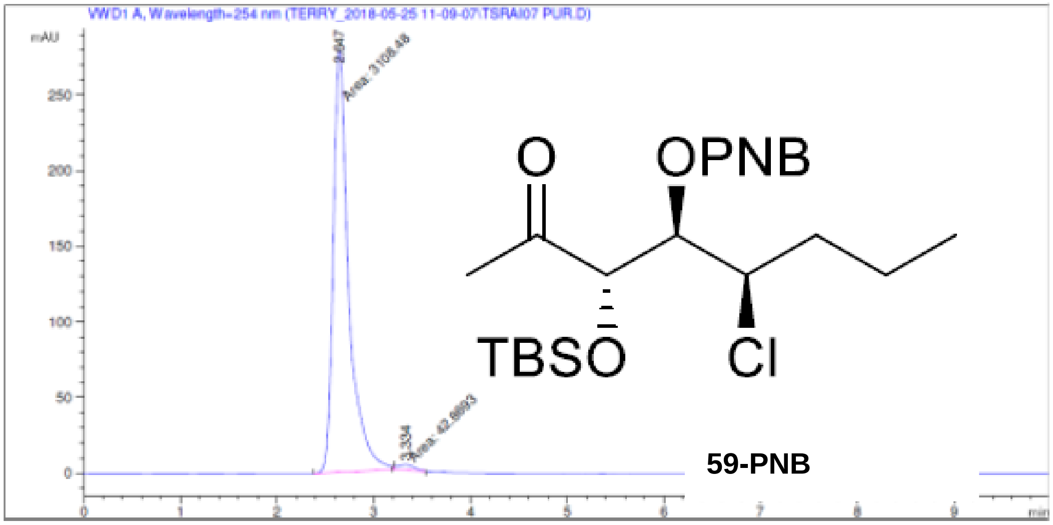


| Peak # | RetTime [min] | Type | Width [min] | Area mAU *s | Height [mAU ] | Area % |
|---|---|---|---|---|---|---|
| 1 | 2.647 | MM | 0.1856 | 3108.48022 | 279.20975 | 98.6397 |
| 2 | 3.334 | MM | 0.1951 | 42.86931 | 3.66210 | 1.3603 |

**Supplementary Figure 18**. Determination of enantiomeric excess of **59**.

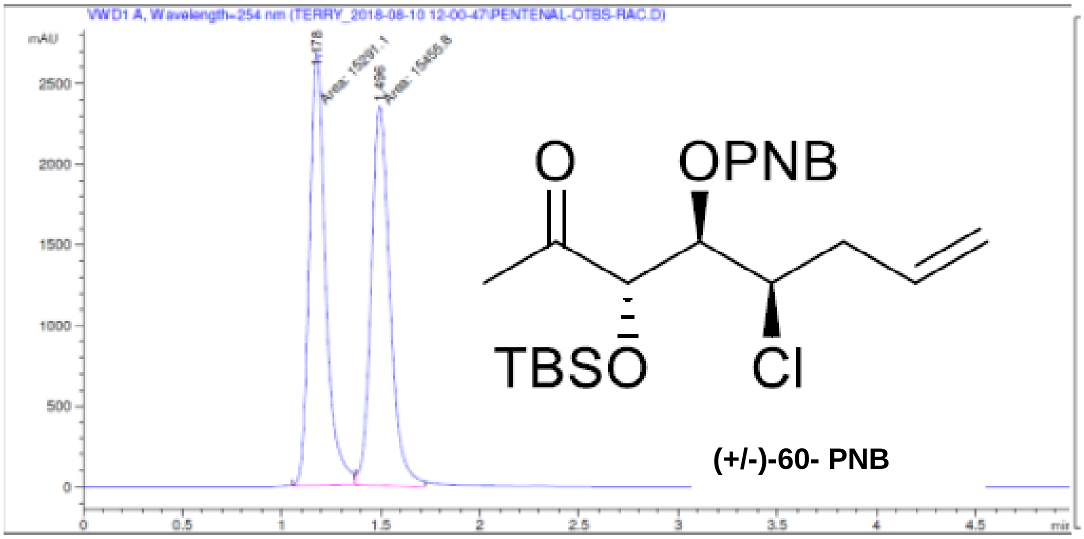


| Peak # | RetTime [min] | Type | Width [min] | Area mAU *s | Height [mAU ] | Area % |
|---|---|---|---|---|---|---|
| 1 | 1.178 | MM | 0.0948 | 1.52911e4 | 2687.56836 | 49.7322 |
| 2 | 1.496 | MM | 0.1094 | 1.54558e4 | 2354.75977 | 50.2678 |

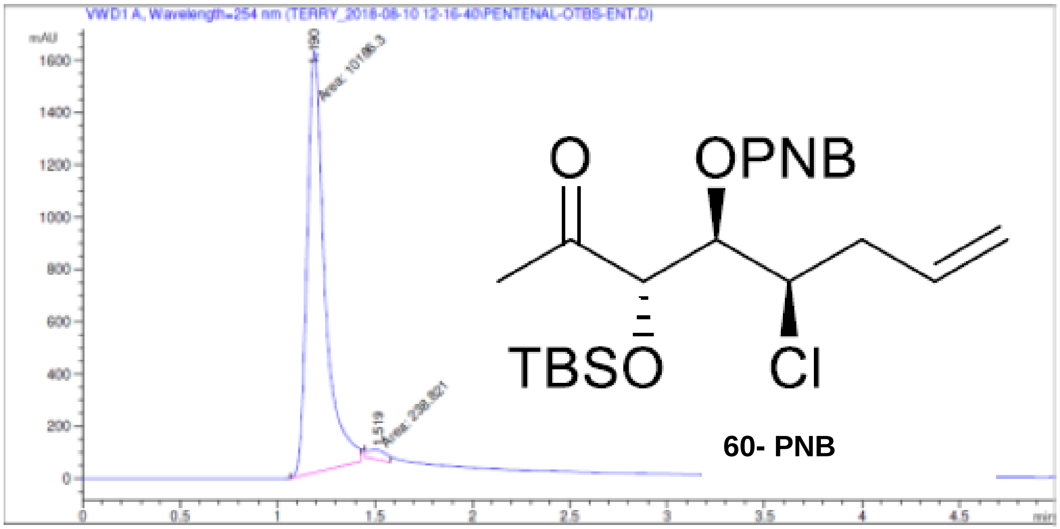


| Peak # | RetTime [min] | Type | Width [min] | Area mAU *s | Height [mAU ] | Area % |
|---|---|---|---|---|---|---|
| 1 | 1.190 | MM | 0.1051 | 1.01863e4 | 1615.77222 | 97.7092 |
| 2 | 1.519 | MM | 0.1092 | 238.82076 | 36.45218 | 2.2908 |

**Supplementary Figure 19**. Determination of enantiomeric excess of **60**.

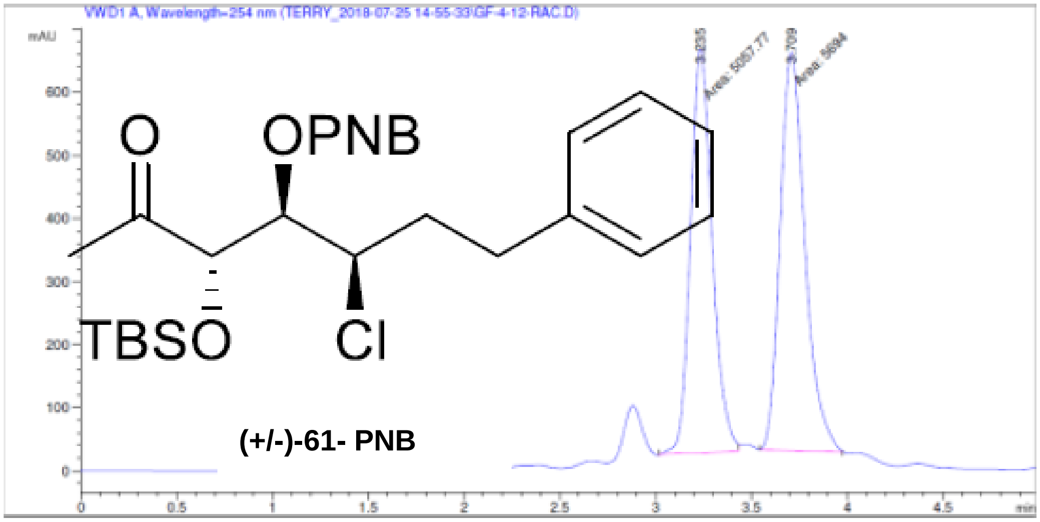


| Peak # | RetTime [min] | Type | Width [min] | Area mAU *s | Height [mAU ] | Area % |
|---|---|---|---|---|---|---|
| 1 | 3.235 | MM | 0.1316 | 5057.76611 | 640.45544 | 47.0413 |
| 2 | 3.709 | MM | 0.1504 | 5693.99561 | 631.12048 | 52.9587 |

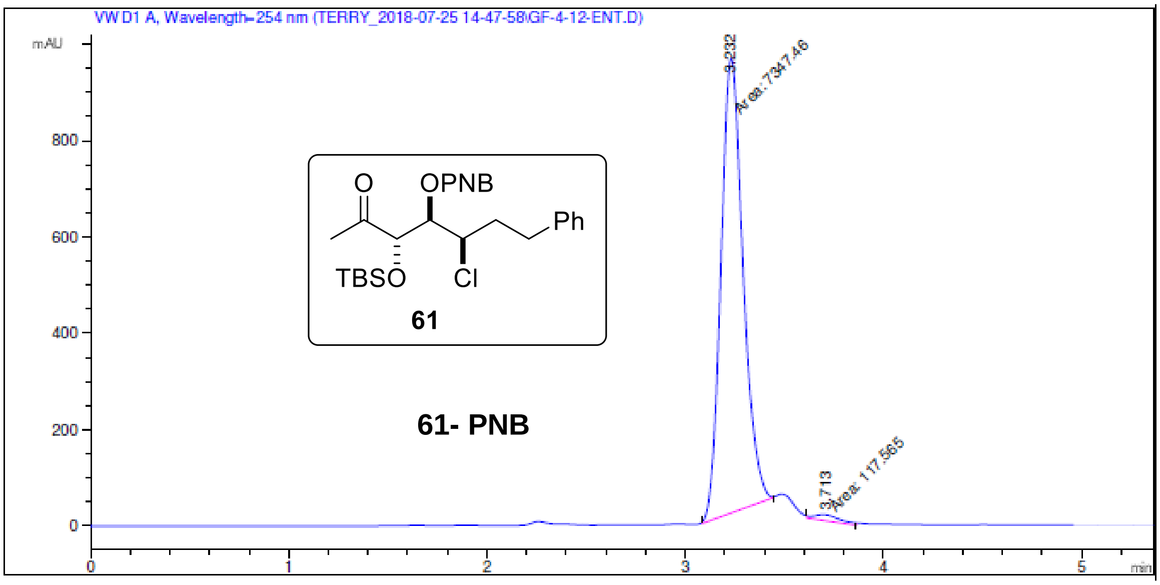


| Peak # | RetTime [min] | Type | Width [min] | Area mAU *s | Height [mAU ] | Area % |
|---|---|---|---|---|---|---|
| 1 | 3.232 | MM | 0.1295 | 7347.46143 | 945.41620 | 98.4251 |
| 2 | 3.713 | MM | 0.1557 | 117.56458 | 12.58270 | 1.5749 |

**Supplementary Figure 20**. Determination of enantiomeric excess of **61**.

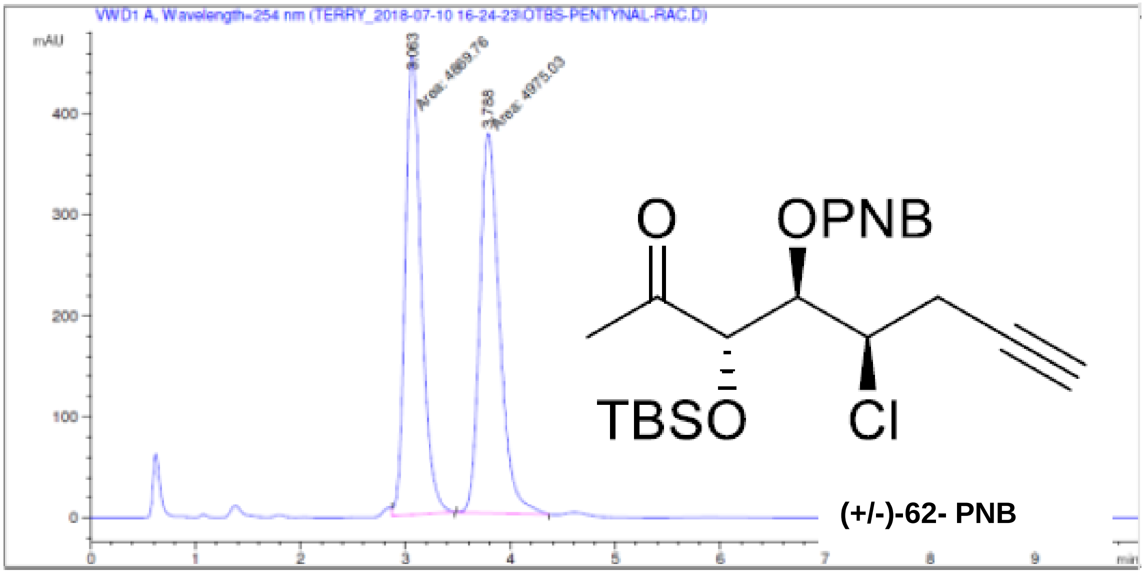


| Peak # | RetTime [min] | Type | Width [min] | Area mAU *s | Height [mAU ] | Area % |
|---|---|---|---|---|---|---|
| 1 | 3.063 | MM | 0.1783 | 4869.75928 | 455.25430 | 49.4653 |
| 2 | 3.788 | MM | 0.2202 | 4975.03027 | 376.62704 | 50.5347 |

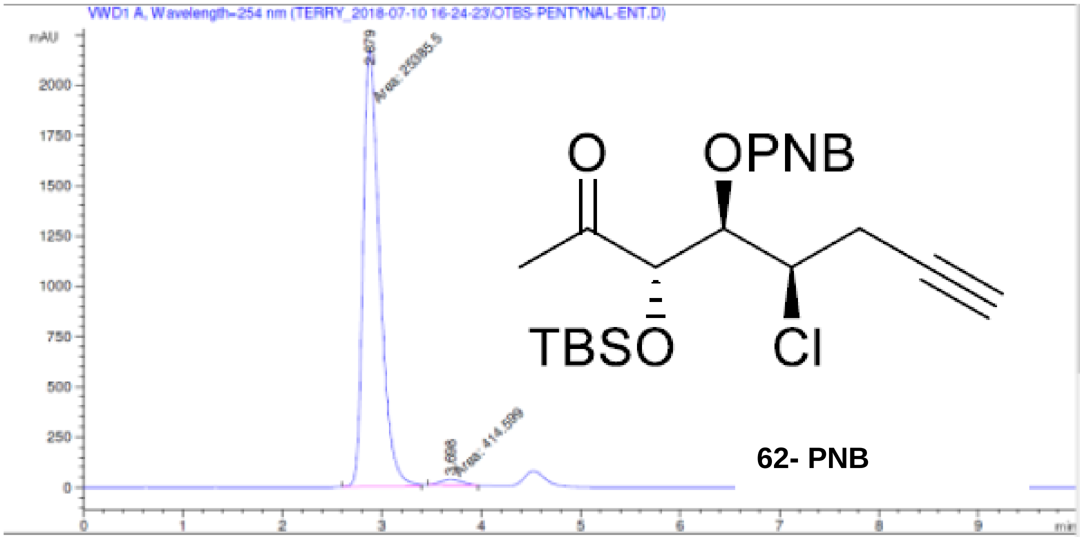


| Peak # | RetTime [min] | Type | Width [min] | Area mAU *s | Height [mAU ] | Area % |
|---|---|---|---|---|---|---|
| 1 | 2.879 | MM | 0.1952 | 2.53855e4 | 2167.88501 | 98.3930 |
| 2 | 3.698 | MM | 0.2541 | 414.59915 | 27.19168 | 1.6070 |

**Supplementary Figure 21**. Determination of enantiomeric excess of **62**.

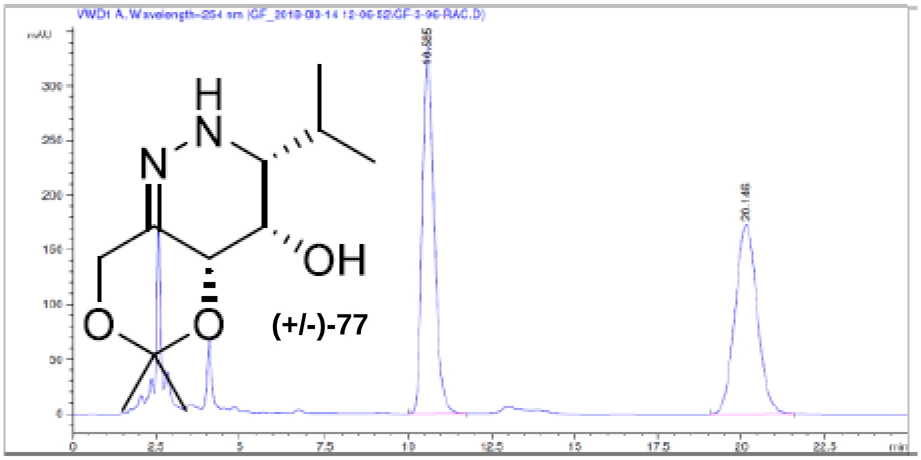

| Peak # | RetTime [min] | Type | Width [min] | Area mAU *s | Height [mAU ] | Area % |
|---|---|---|---|---|---|---|
| 1 | 10.585 | BB | 0.3739 | 8127.26758 | 336.03912 | 50.3763 |
| 2 | 20.146 | BB | 0.7194 | 8005.85010 | 172.87167 | 49.6237 |

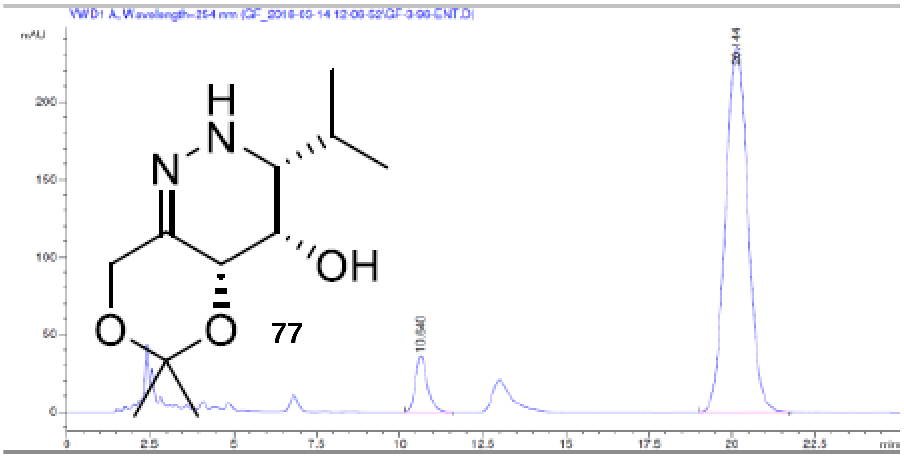

| Peak # | RetTime [min] | Type | Width [min] | Area mAU *s | Height [mAU ] | Area % |
|---|---|---|---|---|---|---|
| 1 | 10.640 | BB | 0.3770 | 888.06750 | 35.76637 | 7.4649 |
| 2 | 20.144 | BB | 0.7259 | 1.10085e4 | 235.51138 | 92.5351 |

**Supplementary Figure 22**. Determination of enantiomeric excess of **77**.

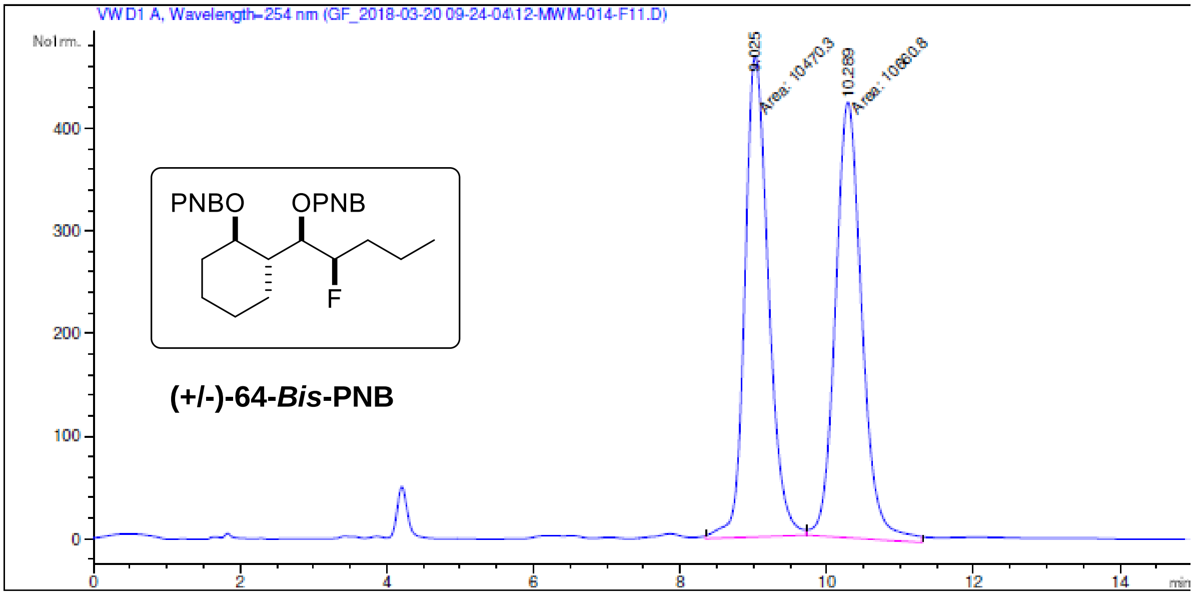


| Peak # | RetTime [min] | Type | Width [min] | Area mAU *s | Height [mAU ] | Area % |
|---|---|---|---|---|---|---|
| 1 | 9.025 | MM | 0.3722 | 1.04703e4 | 468.78464 | 49.5493 |
| 2 | 10.289 | MM | 0.4186 | 1.06608e4 | 424.43393 | 50.4507 |

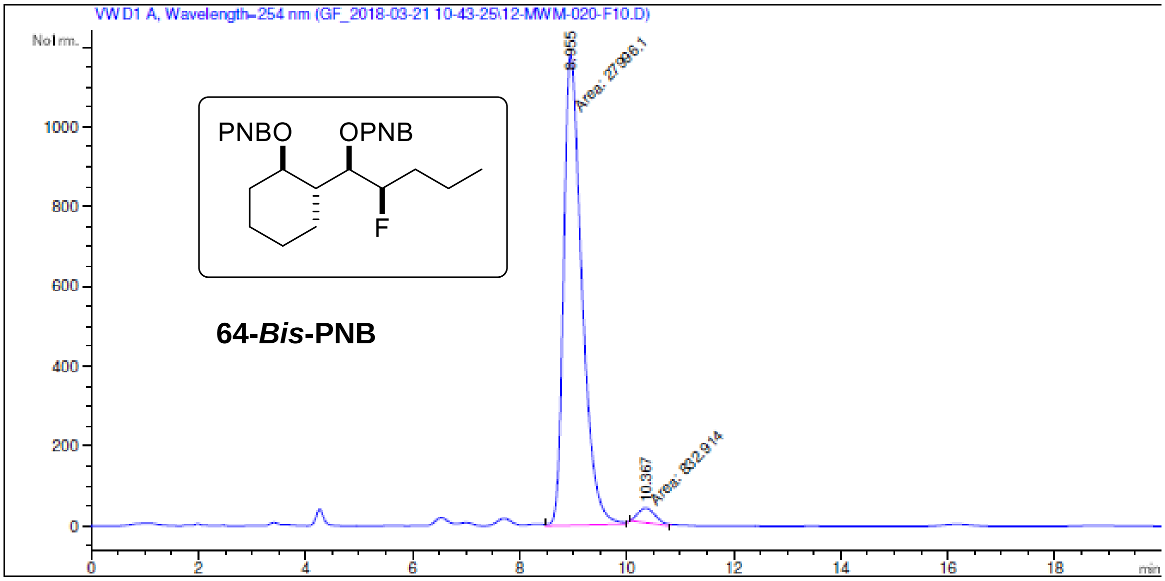


| Peak # | RetTime [min] | Type | Width [min] | Area mAU *s | Height [mAU ] | Area % |
|---|---|---|---|---|---|---|
| 1 | 8.955 | MM | 0.3957 | 2.79961e4 | 1179.06384 | 97.1108 |
| 2 | 10.367 | MM | 0.3787 | 832.91437 | 36.66050 | 2.8892 |

**Supplementary Figure 23**. Determination of enantiomeric excess of **64**.

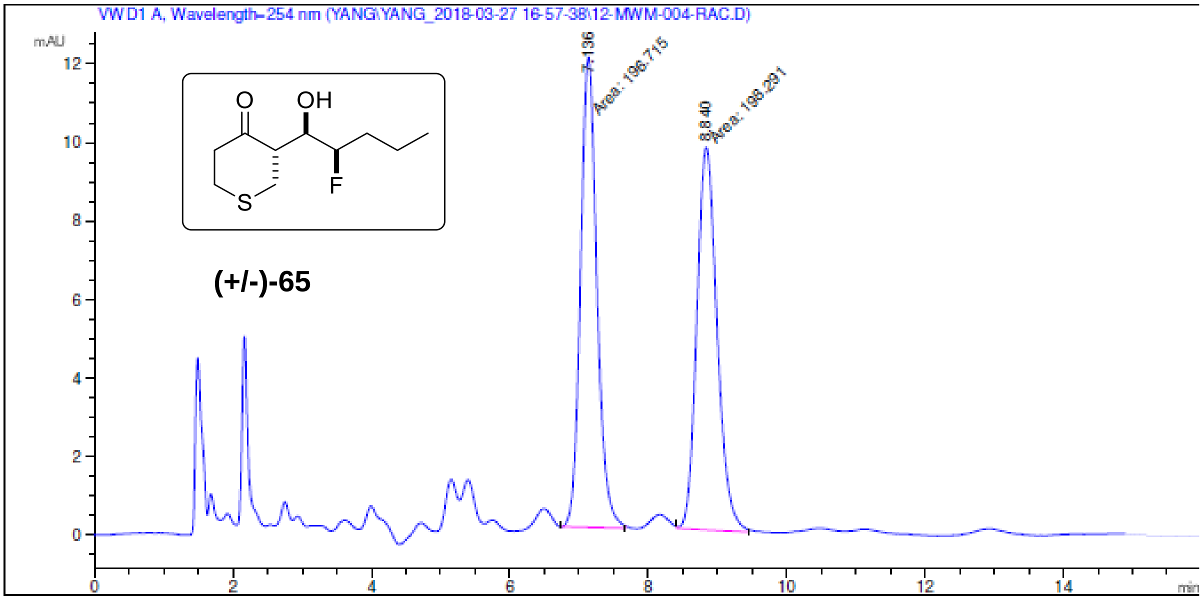


| Peak # | RetTime [min] | Type | Width [min] | Area mAU *s | Height [mAU ] | Area % |
|---|---|---|---|---|---|---|
| 1 | 7.136 | MM | 0.2735 | 196.71518 | 11.98946 | 49.8005 |
| 2 | 8.840 | MM | 0.3388 | 198.29149 | 9.75485 | 50.1995 |

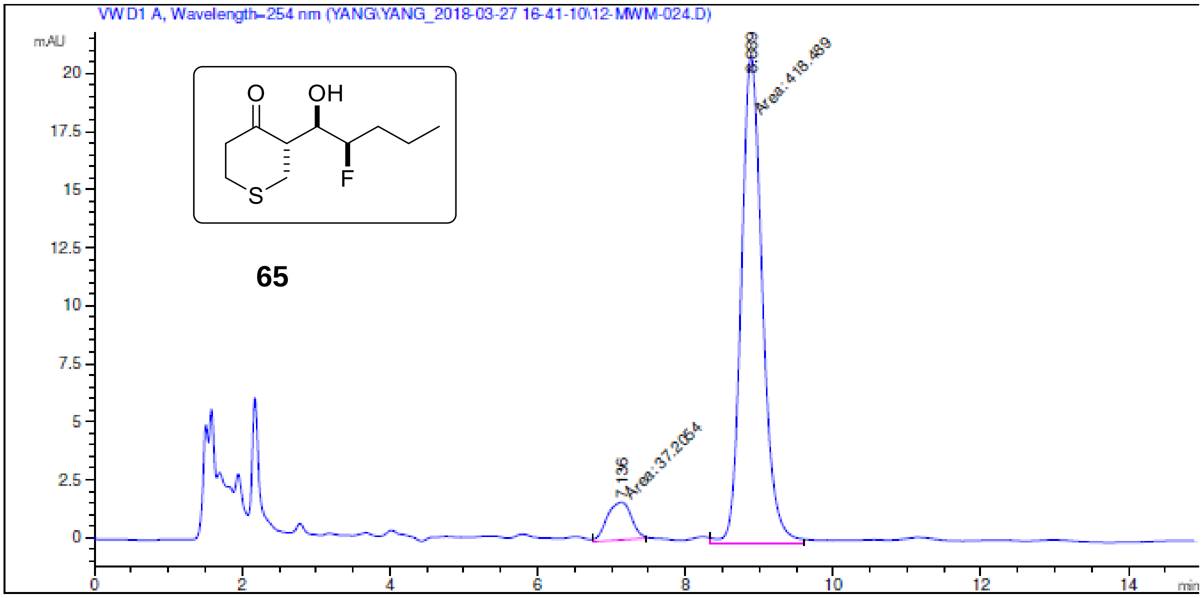


| Peak # | RetTime [min] | Type | Width [min] | Area mAU *s | Height [mAU ] | Area % |
|---|---|---|---|---|---|---|
| 1 | 7.136 | MM | 0.3817 | 37.20538 | 1.62468 | 8.1645 |
| 2 | 8.889 | MM | 0.3323 | 418.48904 | 20.98671 | 91.8355 |

**Supplementary Figure 24**. Determination of enantiomeric excess of **65**.

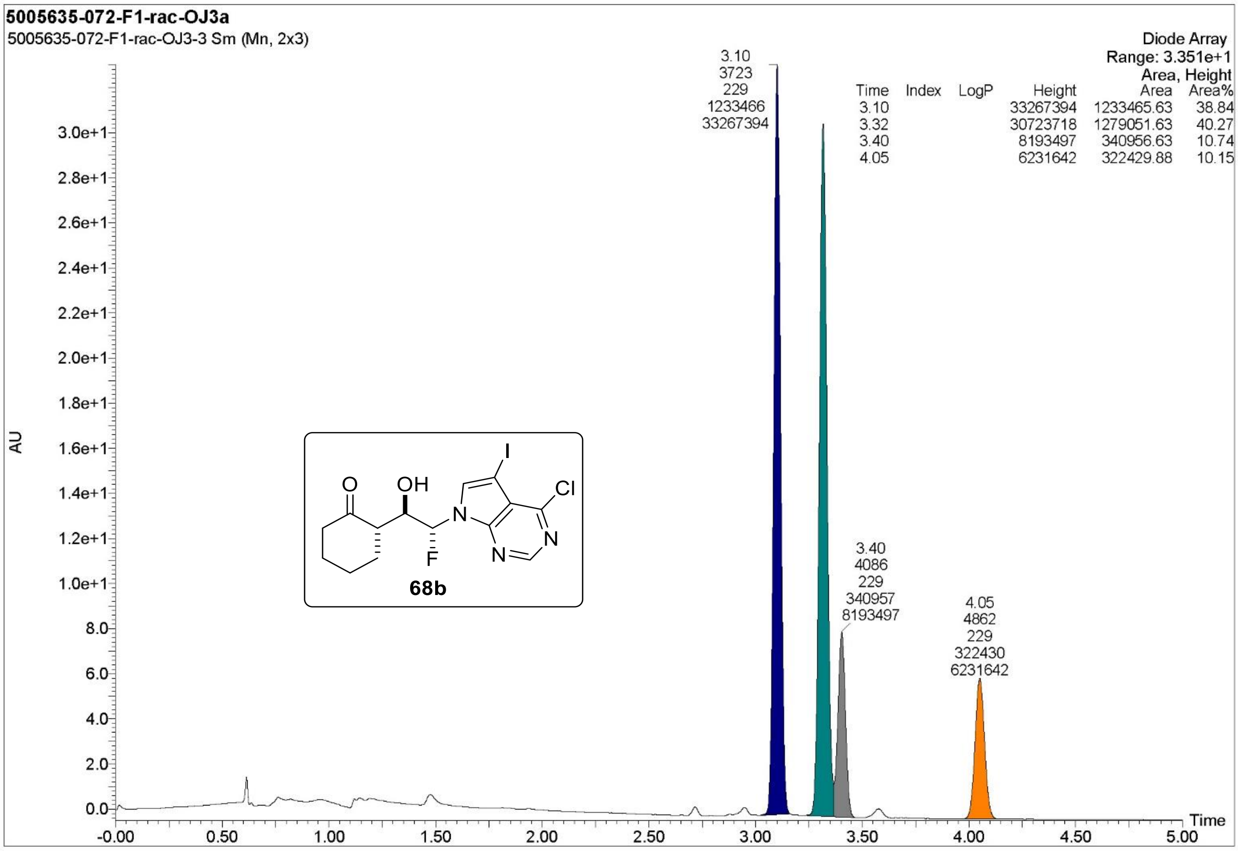


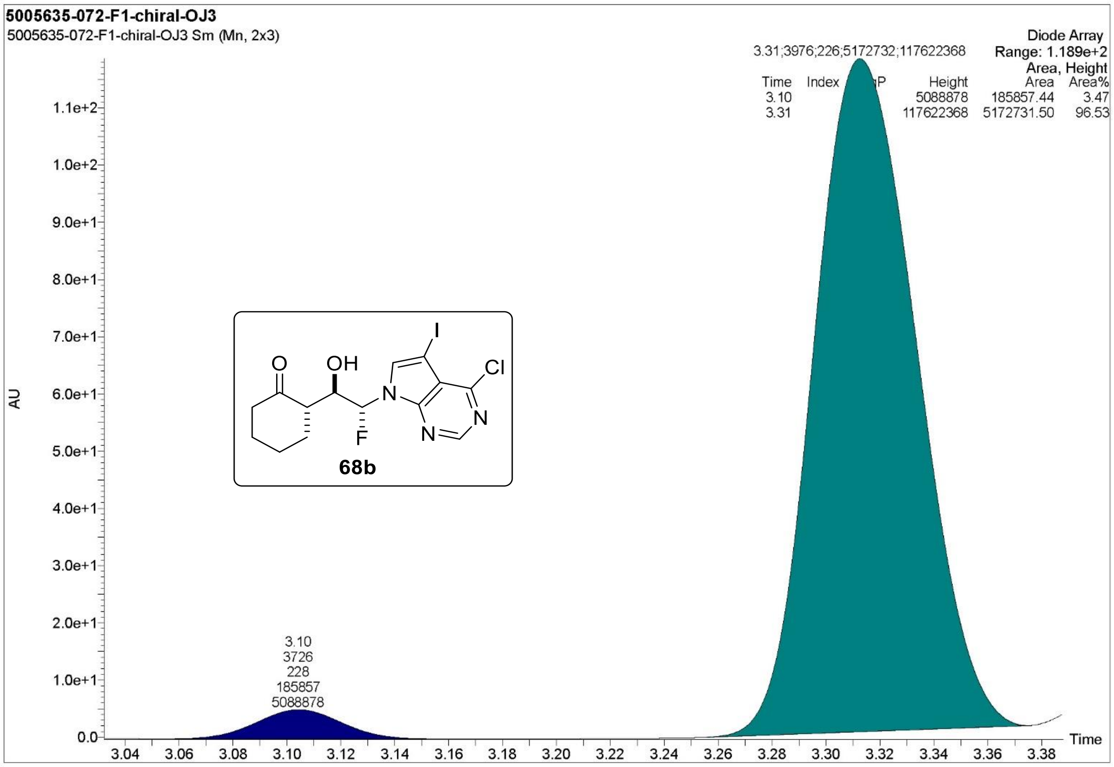


**Supplementary Figure 25**. Determination of enantiomeric excess of **68b**.

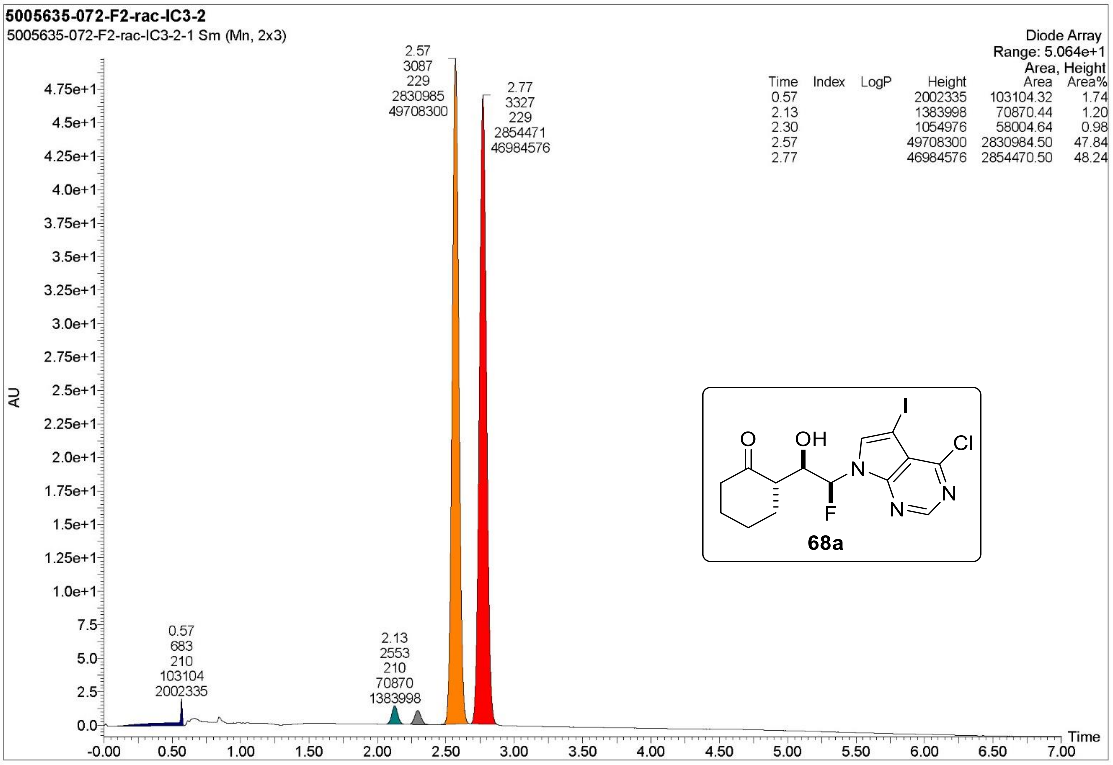


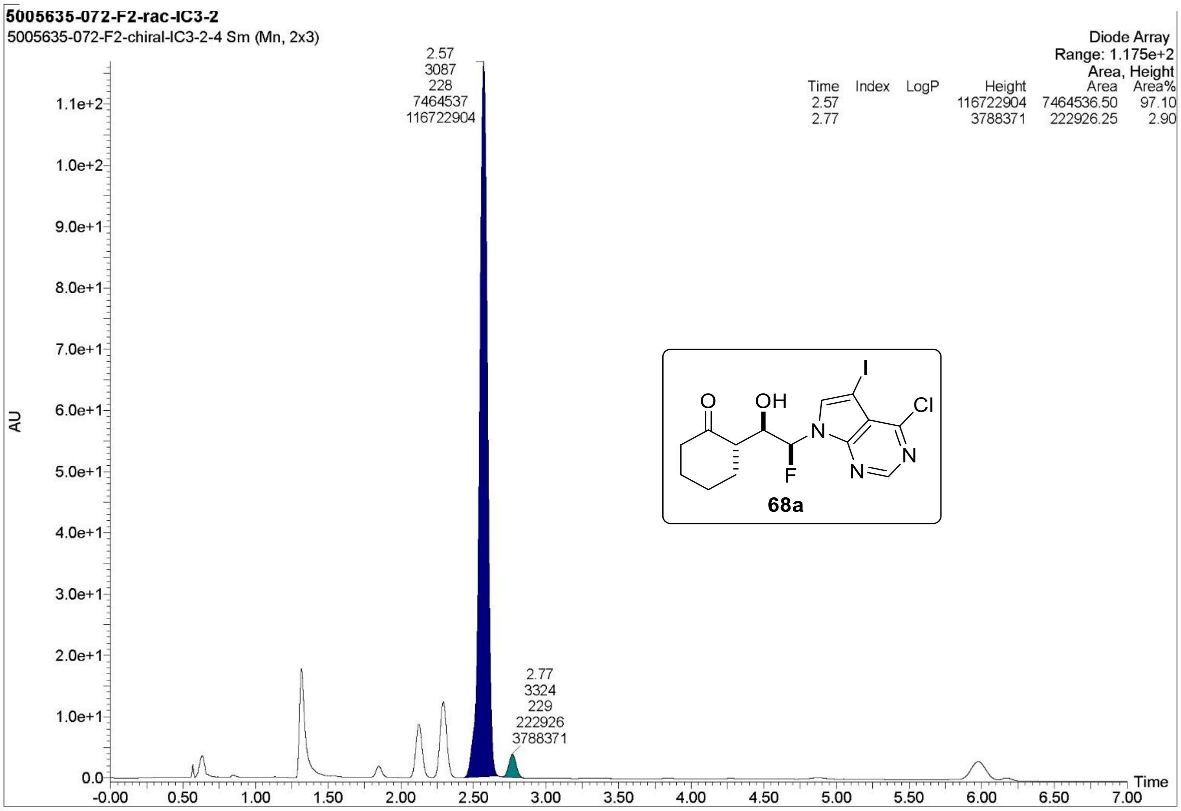


**Supplementary Figure 26**. Determination of enantiomeric excess of **68a**.

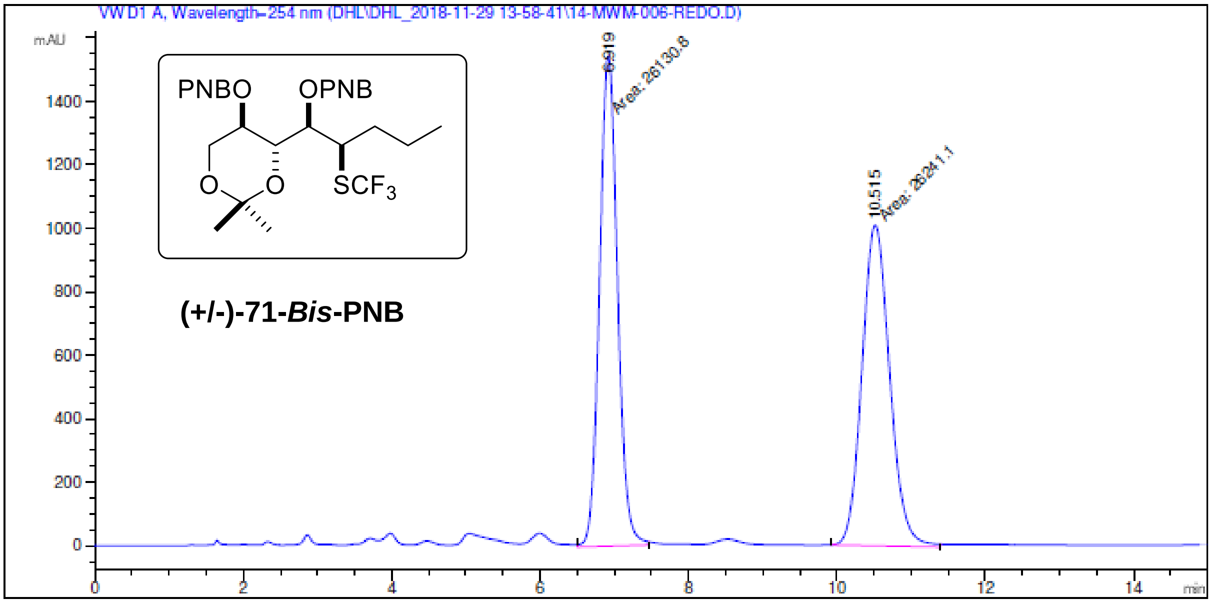


| Peak # | RetTime [min] | Type | Width [min] | Area mAU *s | Height [mAU ] | Area % |
|---|---|---|---|---|---|---|
| 1 | 6.919 | MM | 0.2820 | 2.61308e4 | 1544.30896 | 49.8947 |
| 2 | 10.515 | MM | 0.4330 | 2.62411e4 | 1009.98901 | 50.1053 |

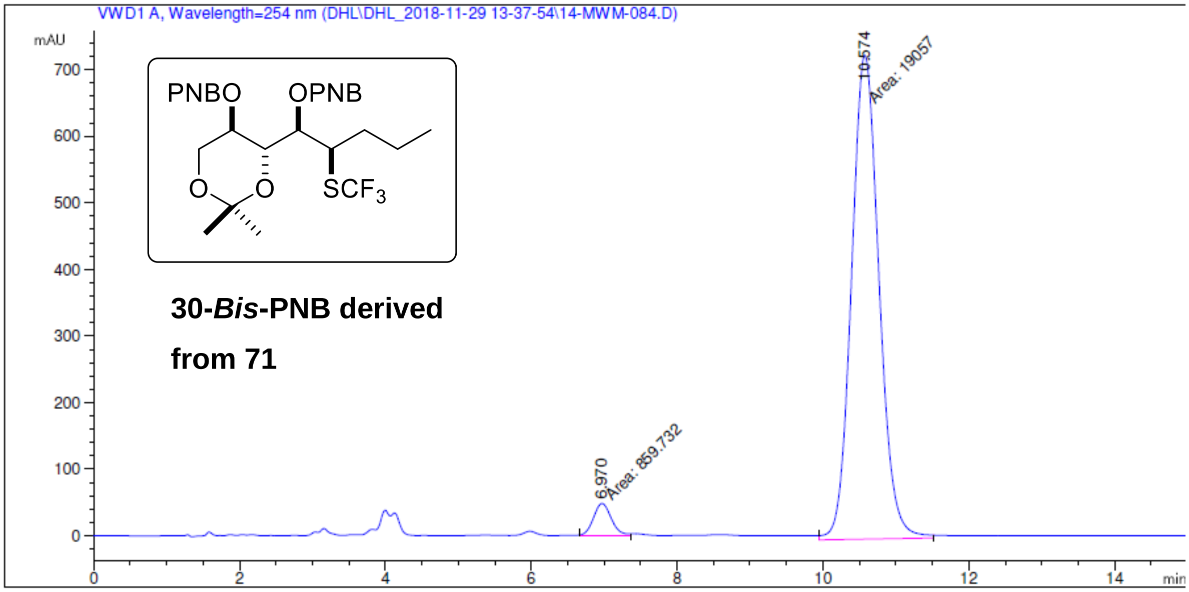


| Peak # | RetTime [min] | Type | Width [min] | Area mAU *s | Height [mAU ] | Area % |
|---|---|---|---|---|---|---|
| 1 | 6.970 | MM | 0.2922 | 859.73206 | 49.03386 | 4.3166 |
| 2 | 10.574 | MM | 0.4371 | 1.90570e4 | 726.57654 | 95.6834 |

**Supplementary Figure 27**. Determination of enantiomeric excess of **71**.

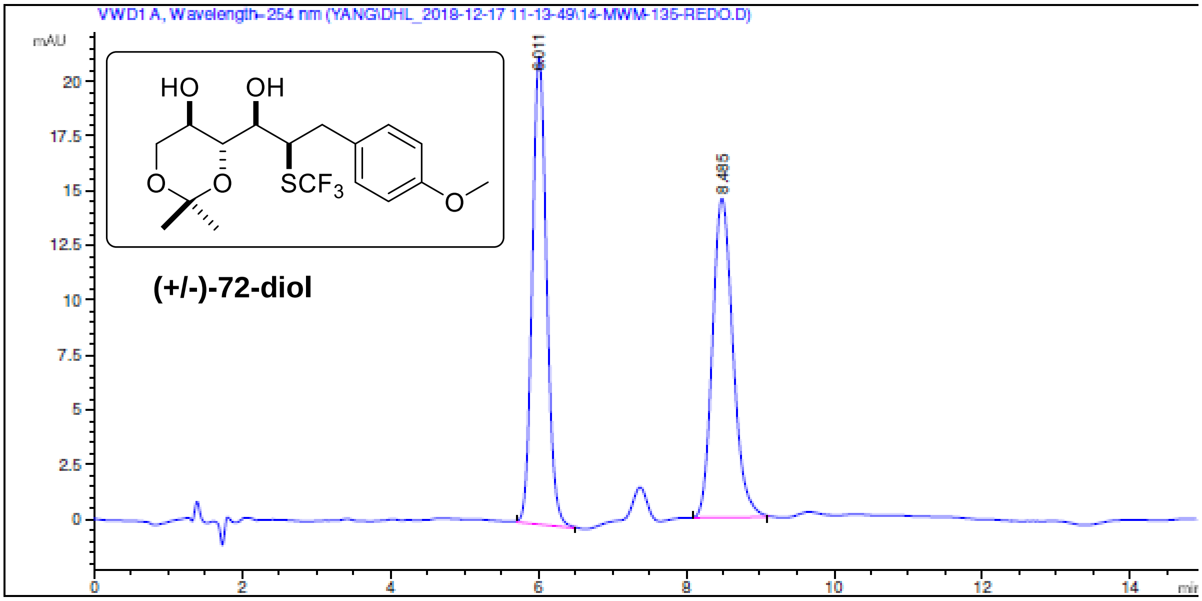


| Peak # | RetTime [min] | Type | Width [min] | Area mAU *s | Height [mAU ] | Area % |
|---|---|---|---|---|---|---|
| 1 | 6.011 | BB | 0.2080 | 287.88373 | 21.38168 | 50.5259 |
| 2 | 8.485 | BB | 0.3015 | 281.89120 | 14.56703 | 49.4741 |

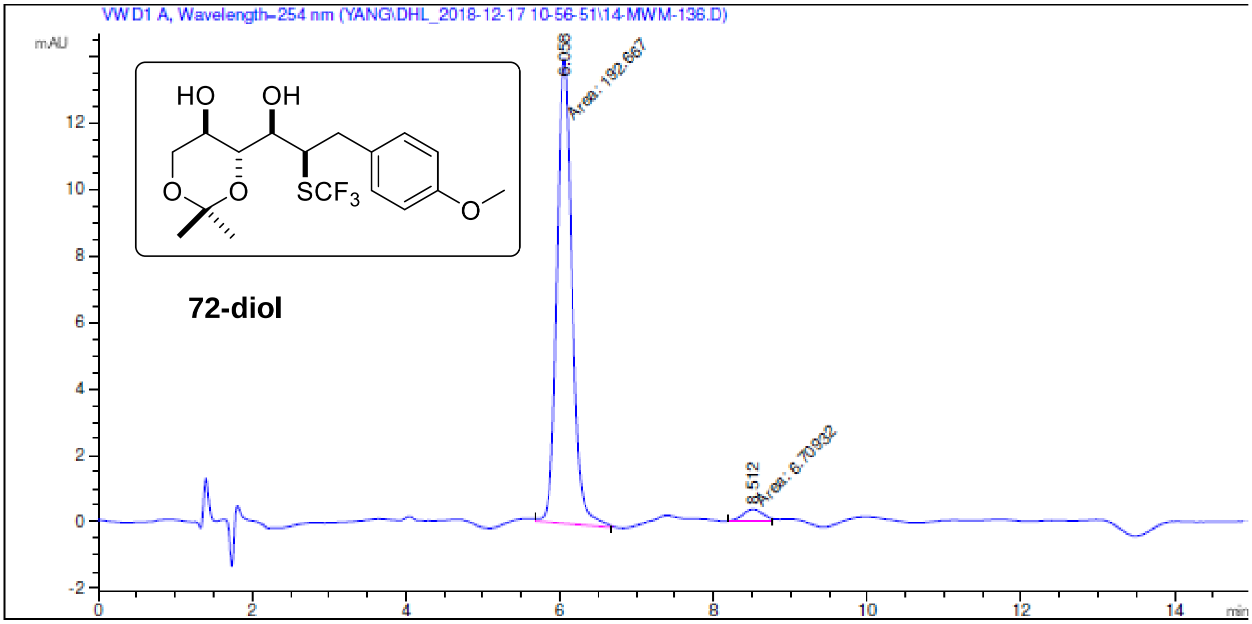


| Peak # | RetTime [min] | Type | Width [min] | Area mAU *s | Height [mAU ] | Area % |
|---|---|---|---|---|---|---|
| 1 | 6.058 | MM | 0.2298 | 192.66653 | 13.97408 | 96.6348 |
| 2 | 8.512 | MM | 0.3107 | 6.70932 | 3.59961e-1 | 3.3652 |

**Supplementary Figure 28**. Determination of enantiomeric excess of **72**.

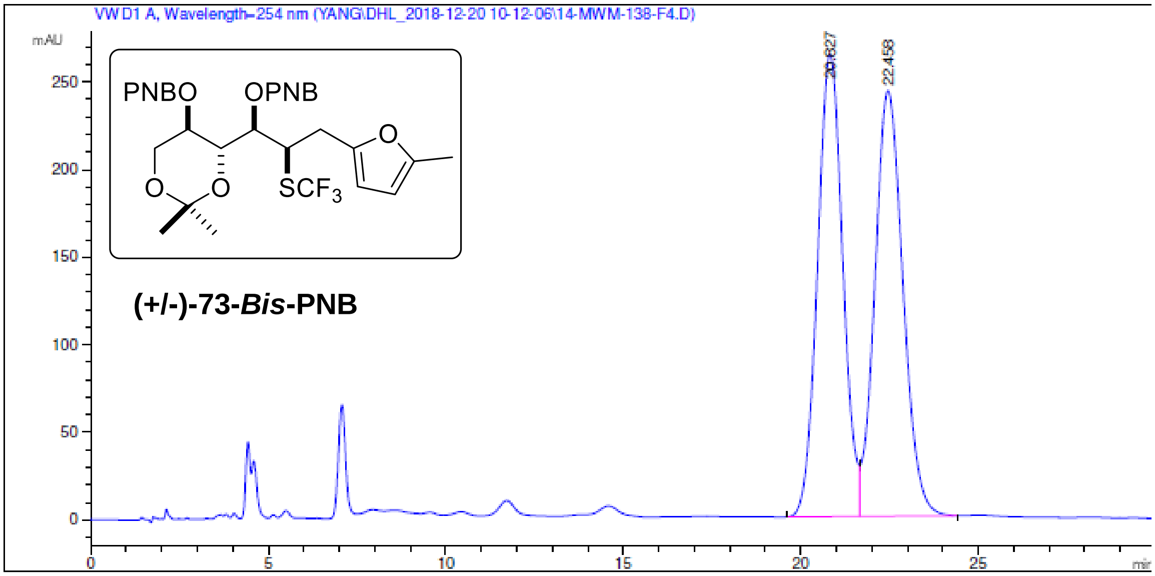


| Peak # | RetTime [min] | Type | Width [min] | Area mAU *s | Height [mAU ] | Area % |
|---|---|---|---|---|---|---|
| 1 | 20.827 | BV | 0.7663 | 1.30500e4 | 263.85162 | 49.2945 |
| 2 | 22.458 | VB | 0.8541 | 1.34235e4 | 243.01193 | 50.7055 |

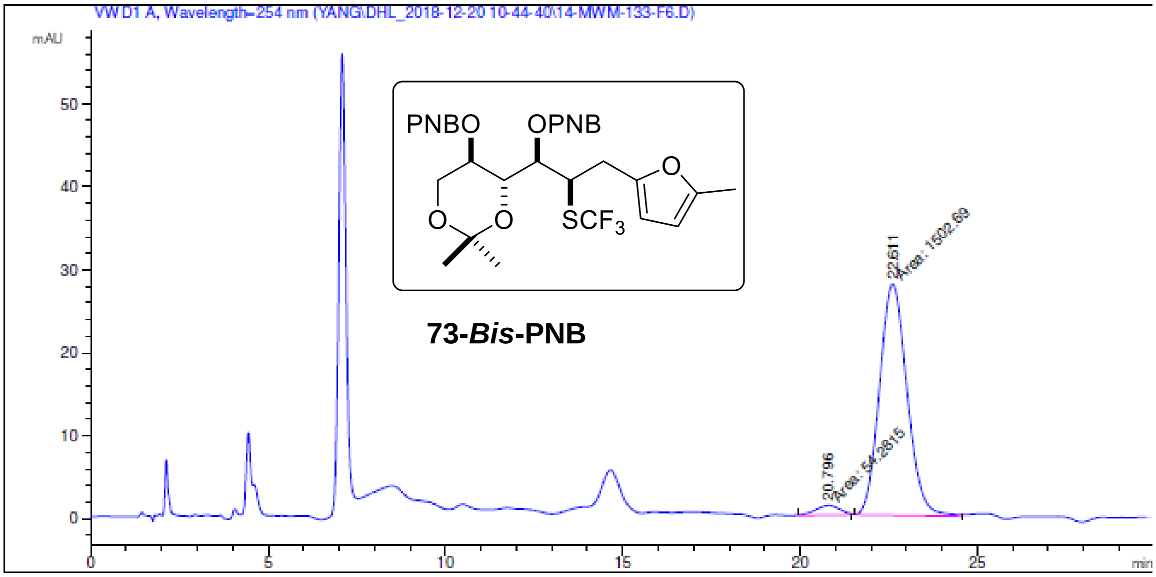


| Peak # | RetTime [min] | Type | Width [min] | Area mAU *s | Height [mAU ] | Area % |
|---|---|---|---|---|---|---|
| 1 | 20.796 | MM | 0.7802 | 54.28146 | 1.15951 | 3.4864 |
| 2 | 22.611 | MM | 0.8990 | 1502.68835 | 27.85789 | 96.5136 |

**Supplementary Figure 29**. Determination of enantiomeric excess of **73**.

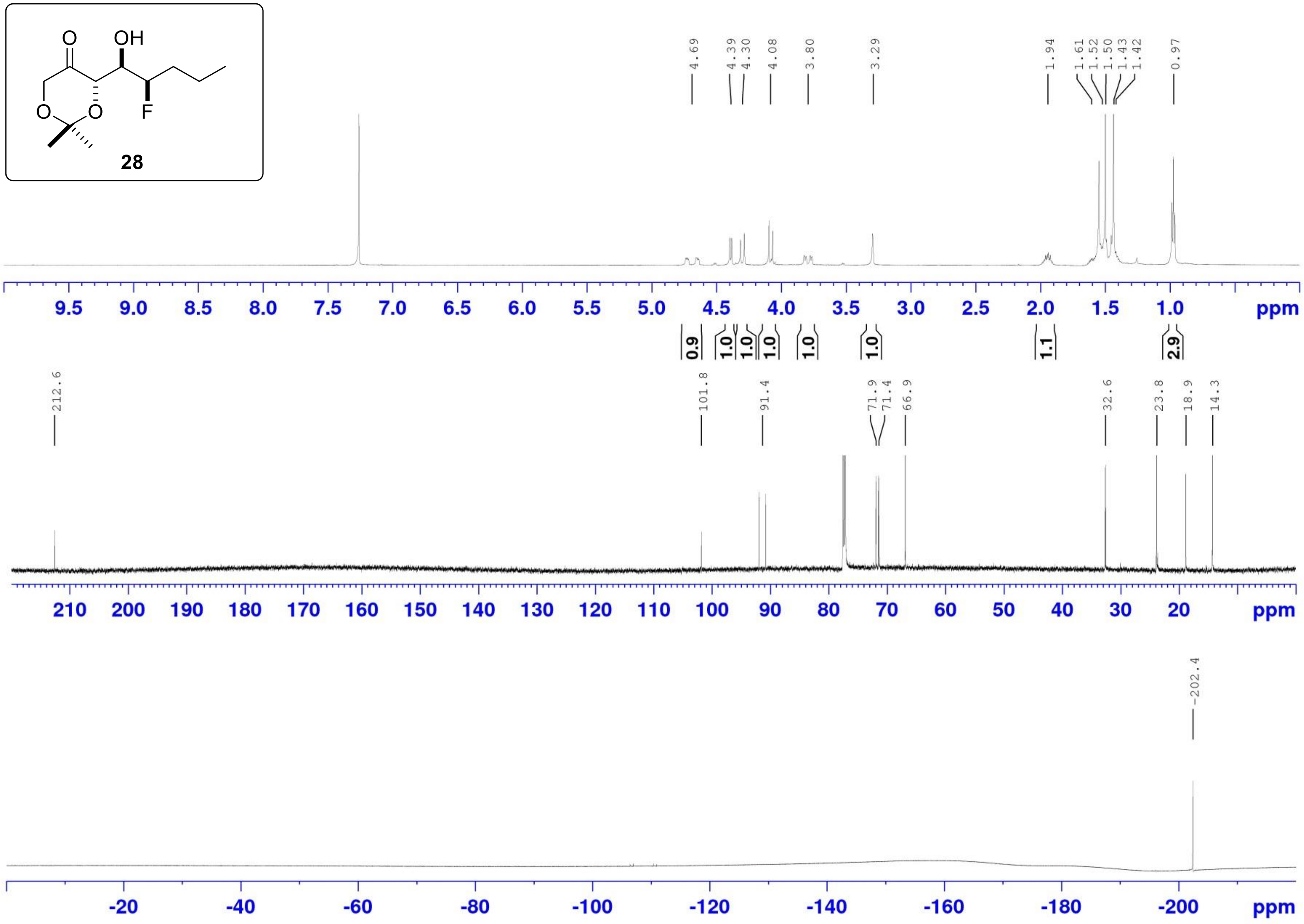


**Supplementary Figure 30**. NMR spectra of **28**.

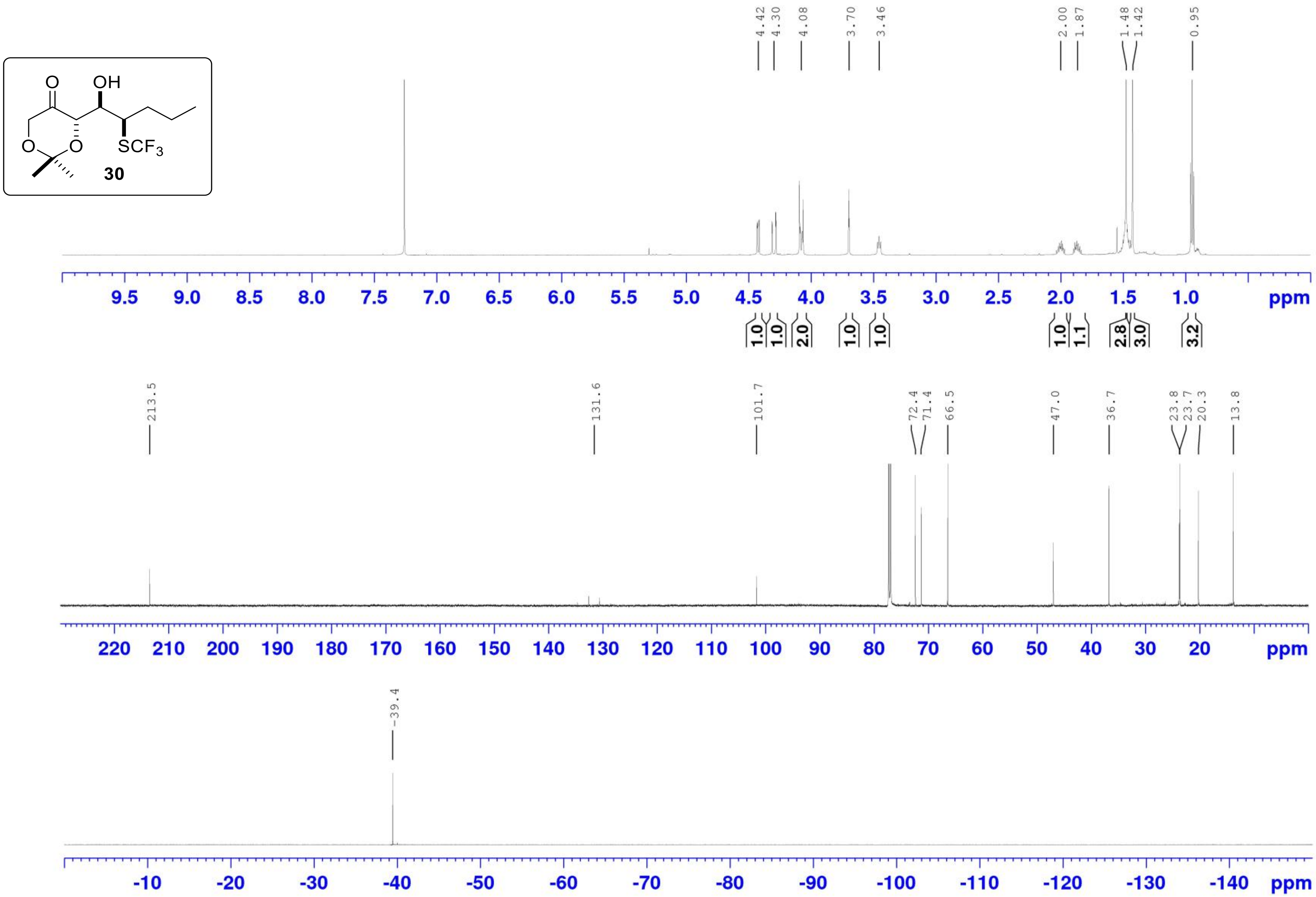


**Supplementary Figure 31**. NMR spectra of **30**.

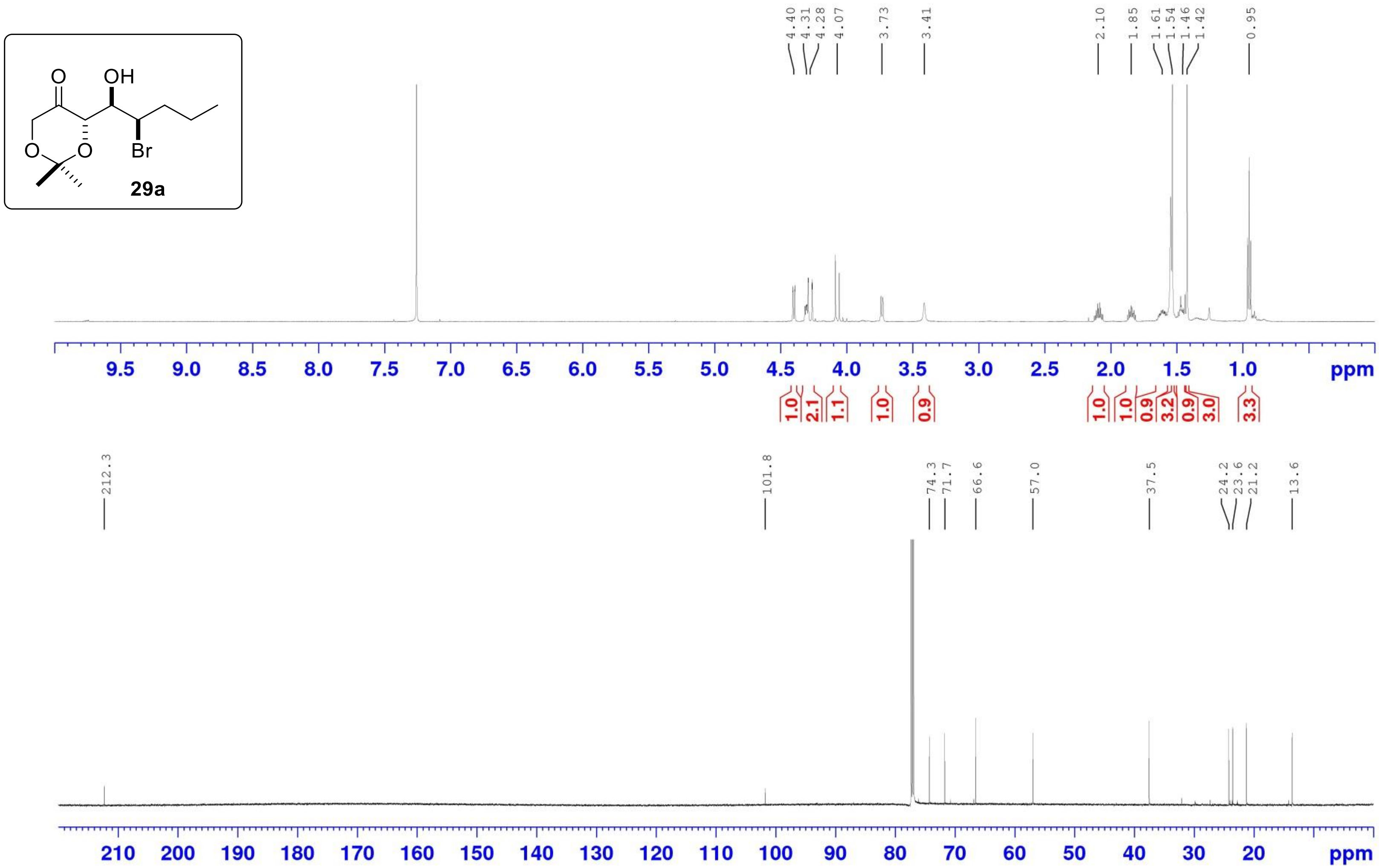


**Supplementary Figure 32**. NMR spectra of **29a**.

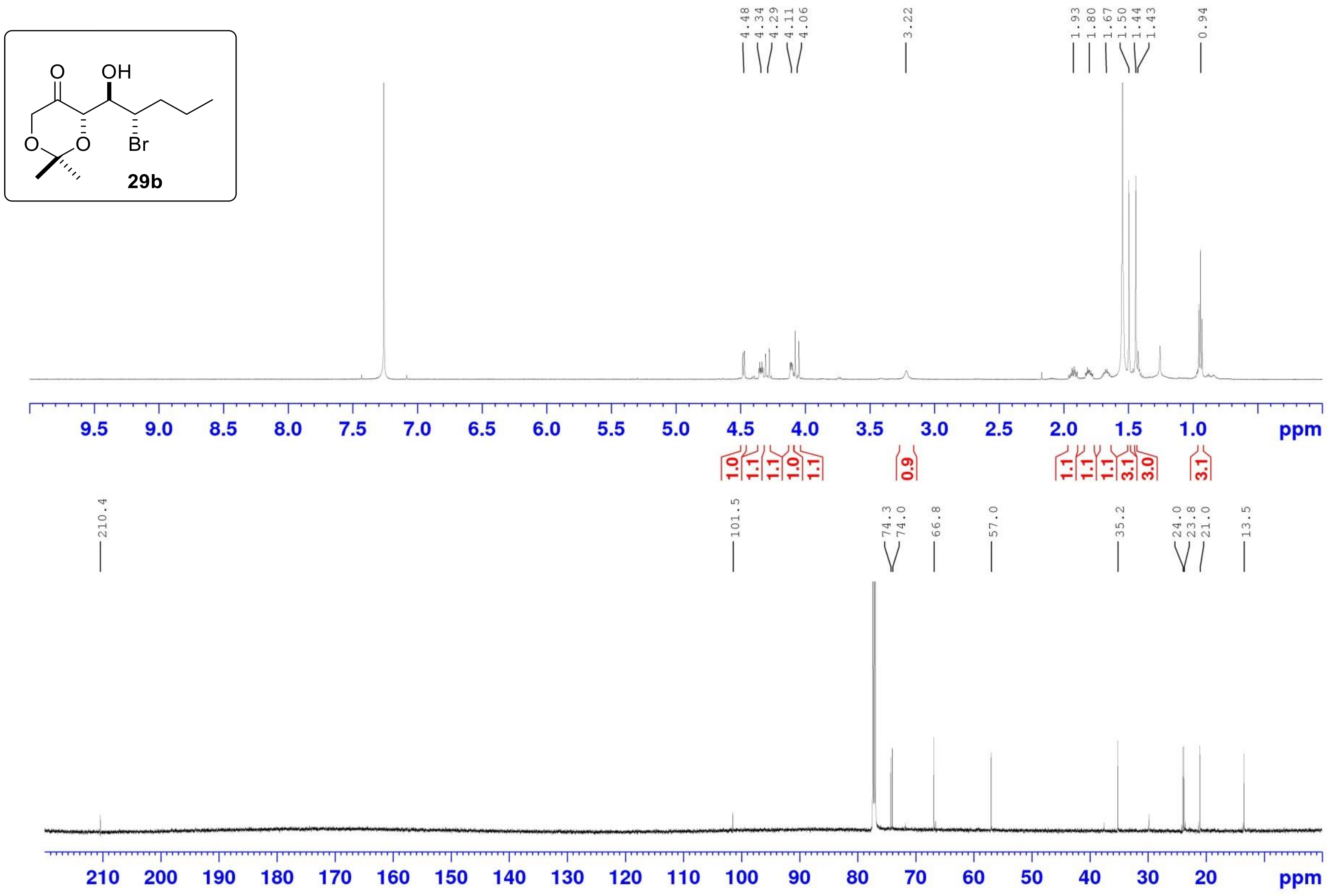


**Supplementary Figure 33**. NMR spectra of **29b**.

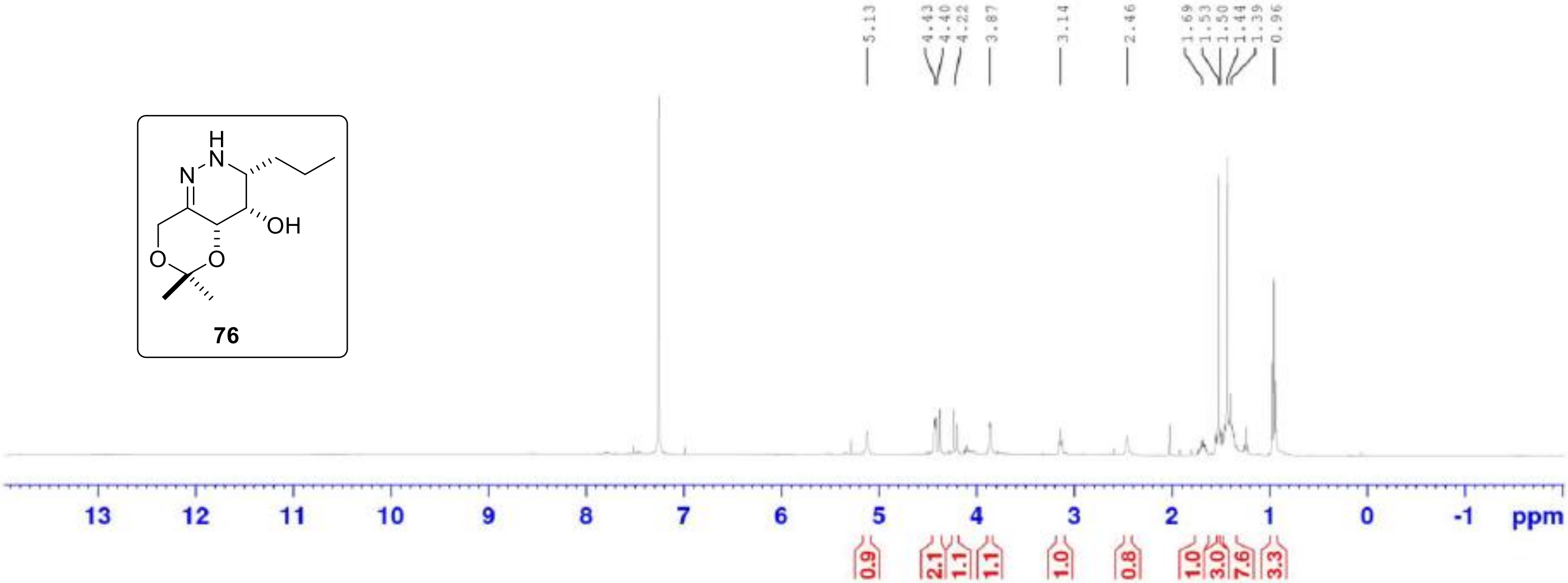


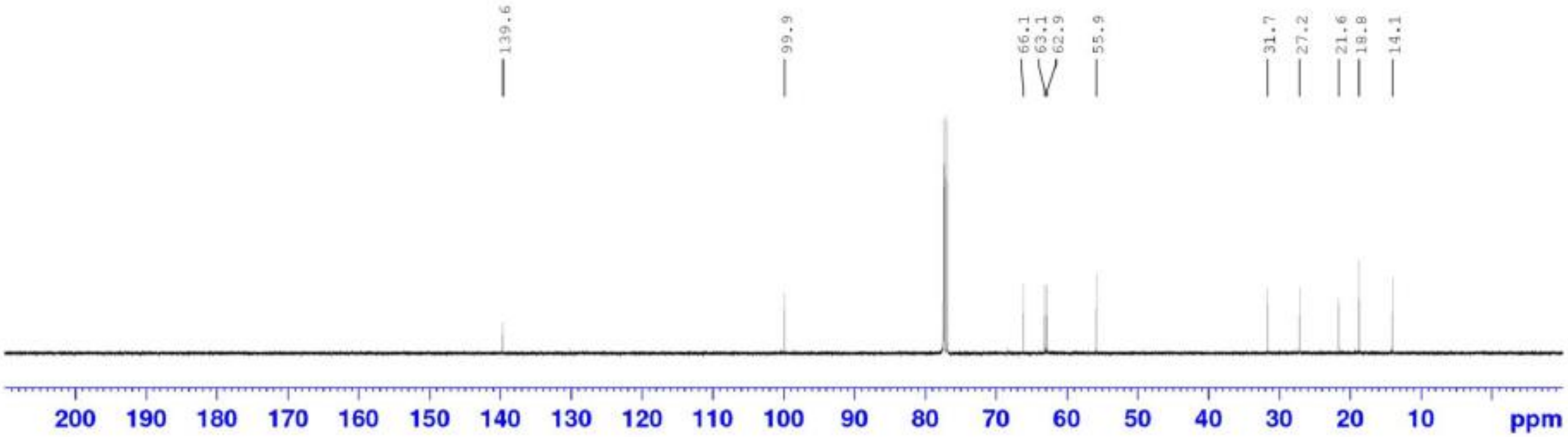


**Supplementary Figure 33**. NMR spectra of **76**.

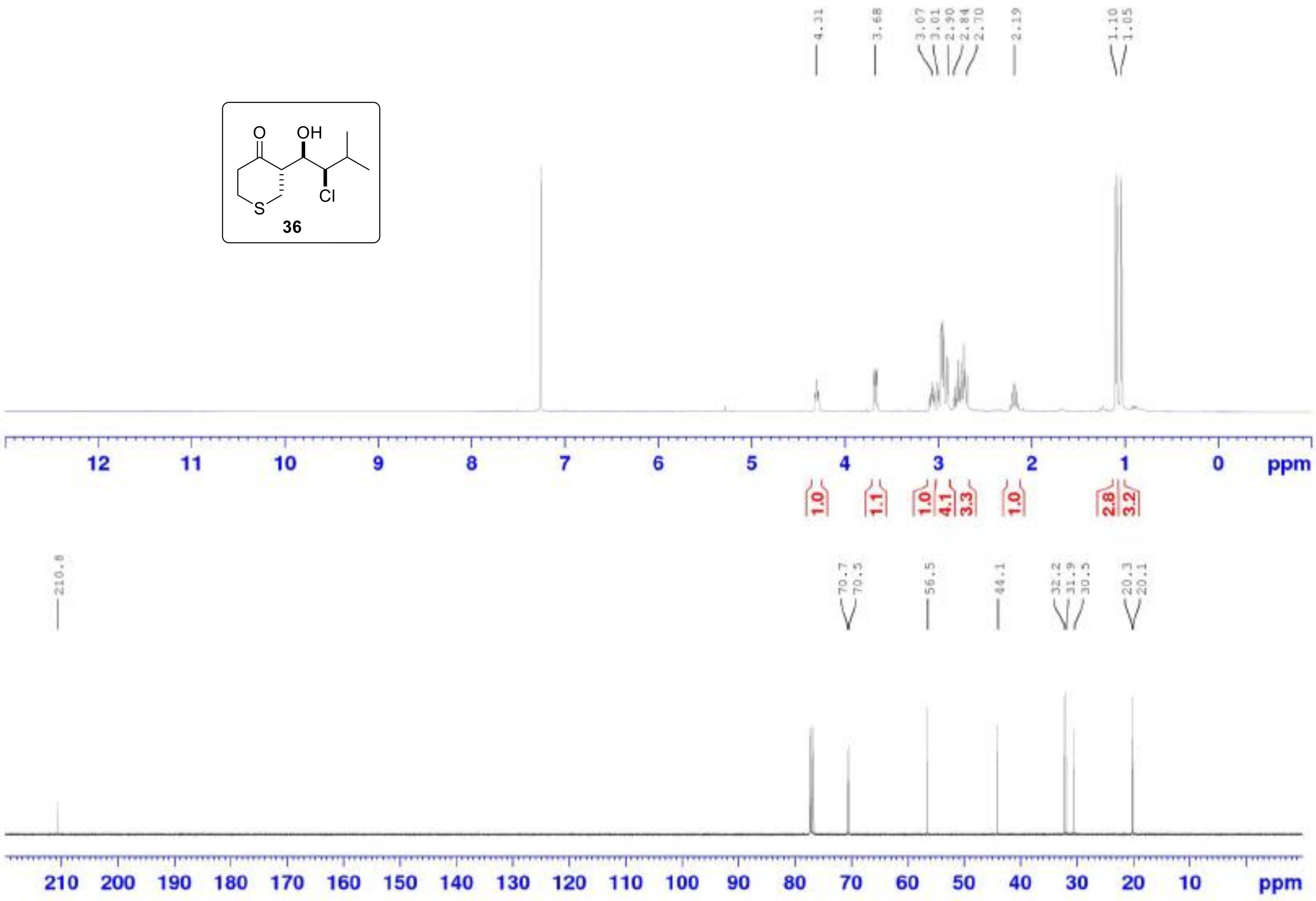


**Supplementary Figure 33**. NMR spectra of **36**.

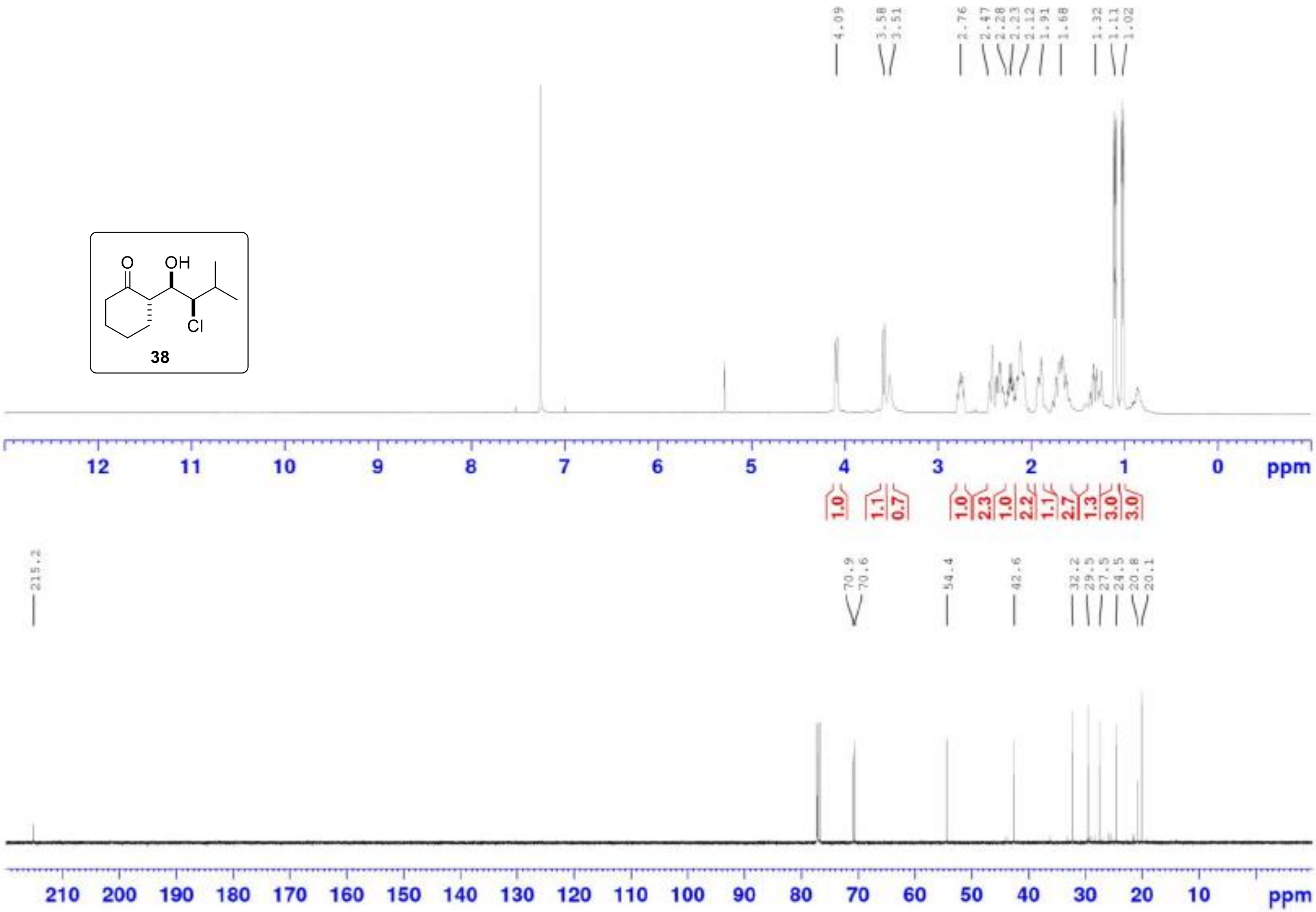


**Supplementary Figure 34**. NMR spectra of **38**.

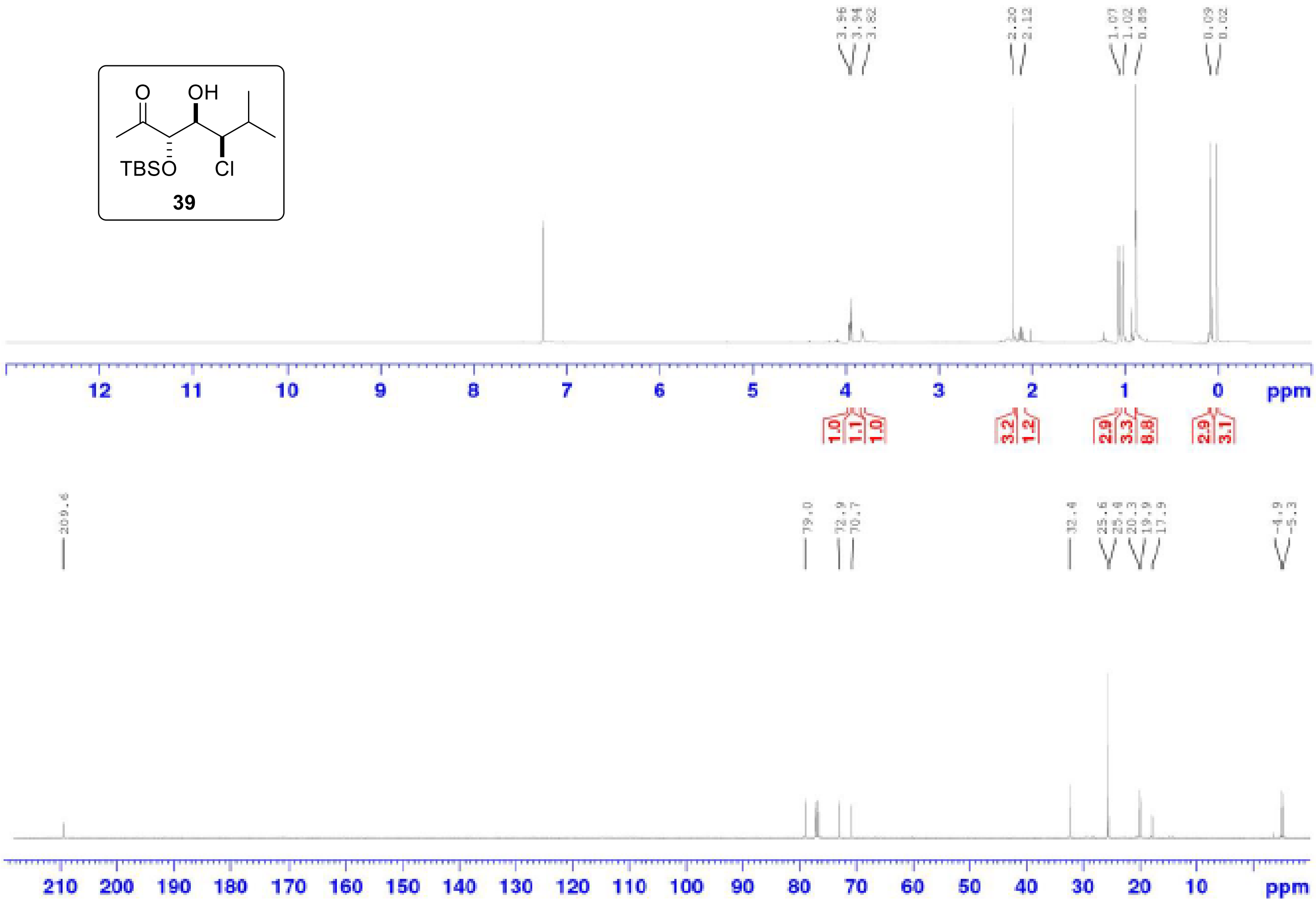


**Supplementary Figure 35**. NMR spectra of **39**.

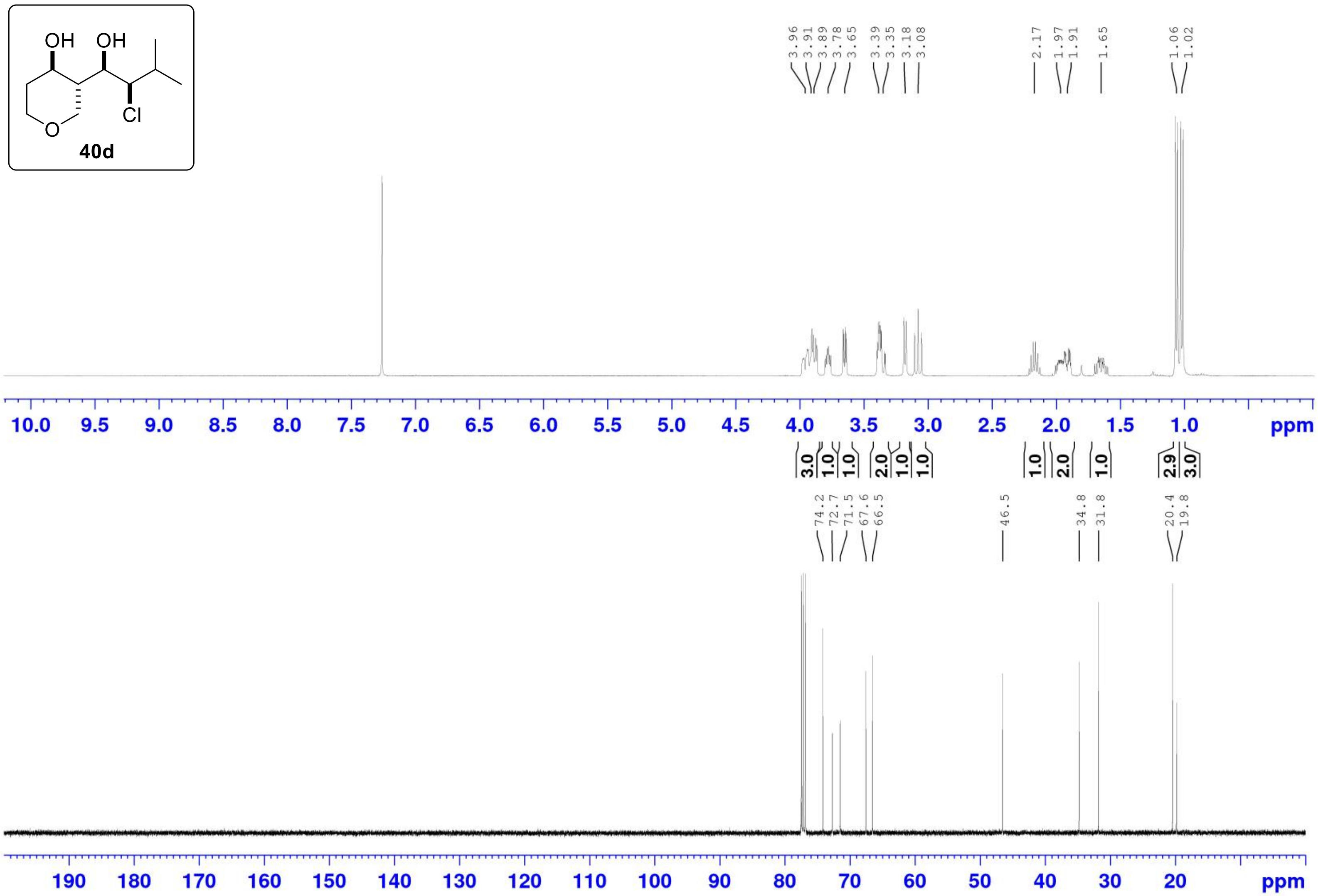


**Supplementary Figure 36**. NMR spectra of **40d**.

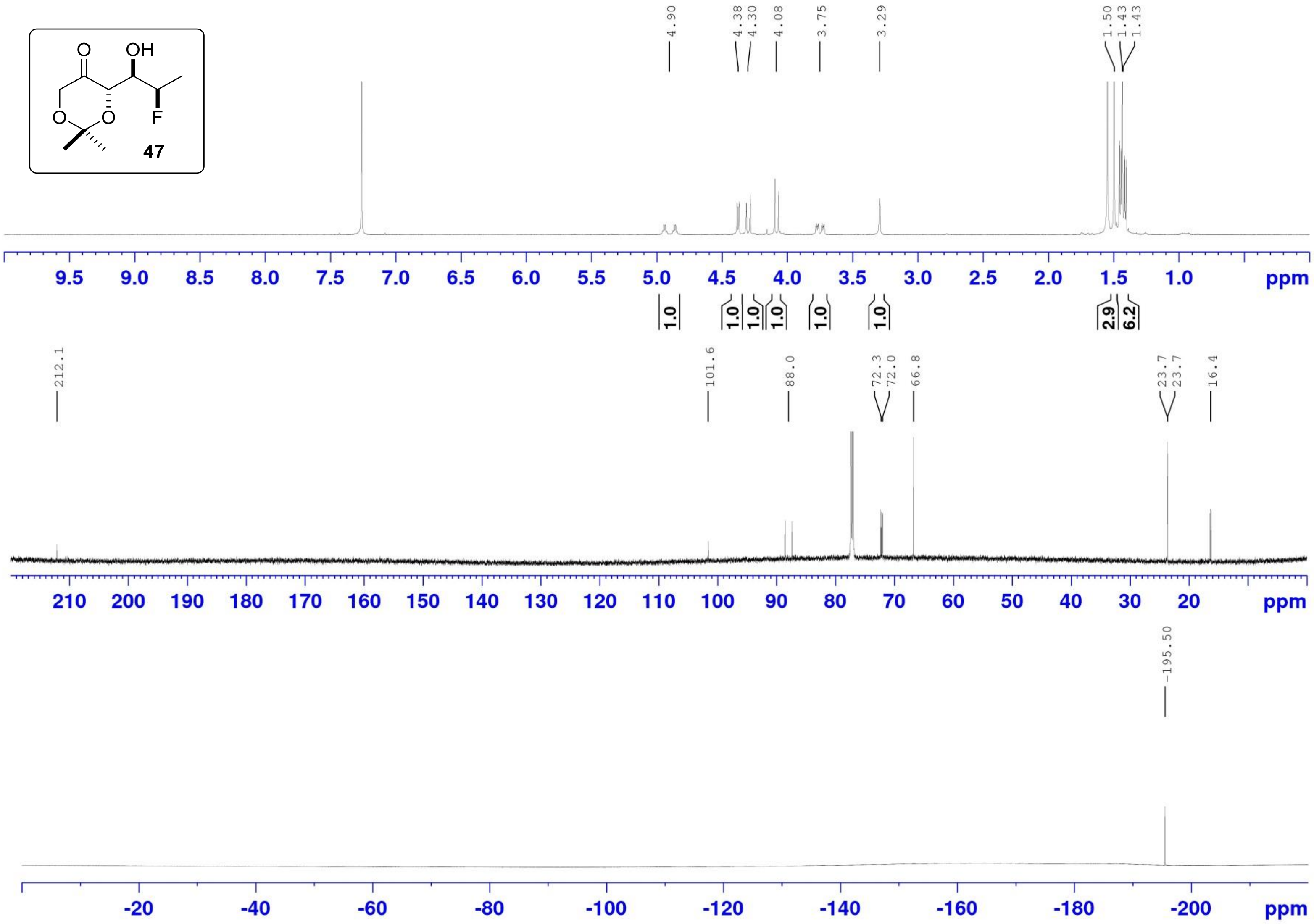


**Supplementary Figure 37**. NMR spectra of **47**.

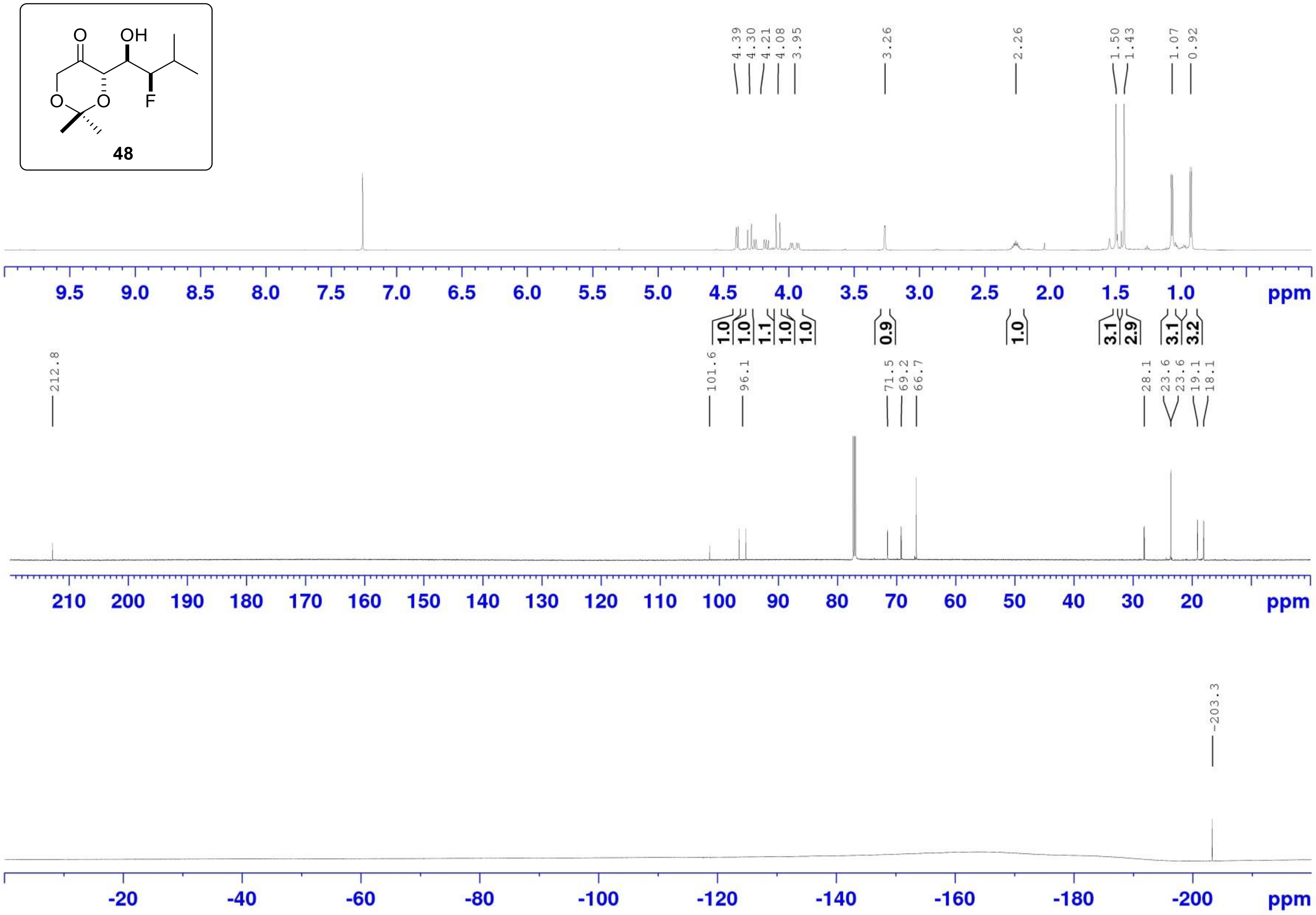


**Supplementary Figure 38**. NMR spectra of **48**.

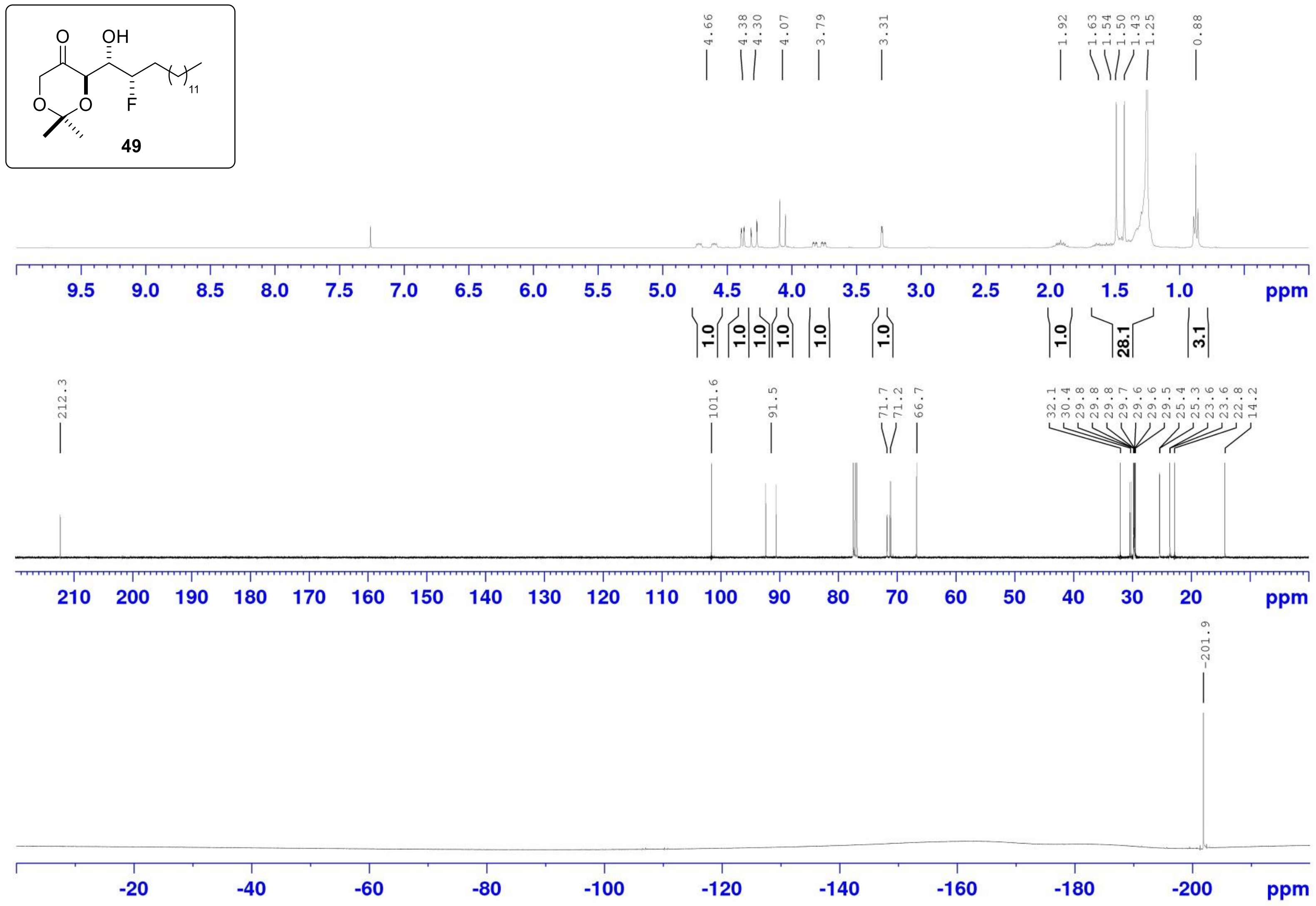

**Supplementary Figure 39**. NMR spectra of **49**.

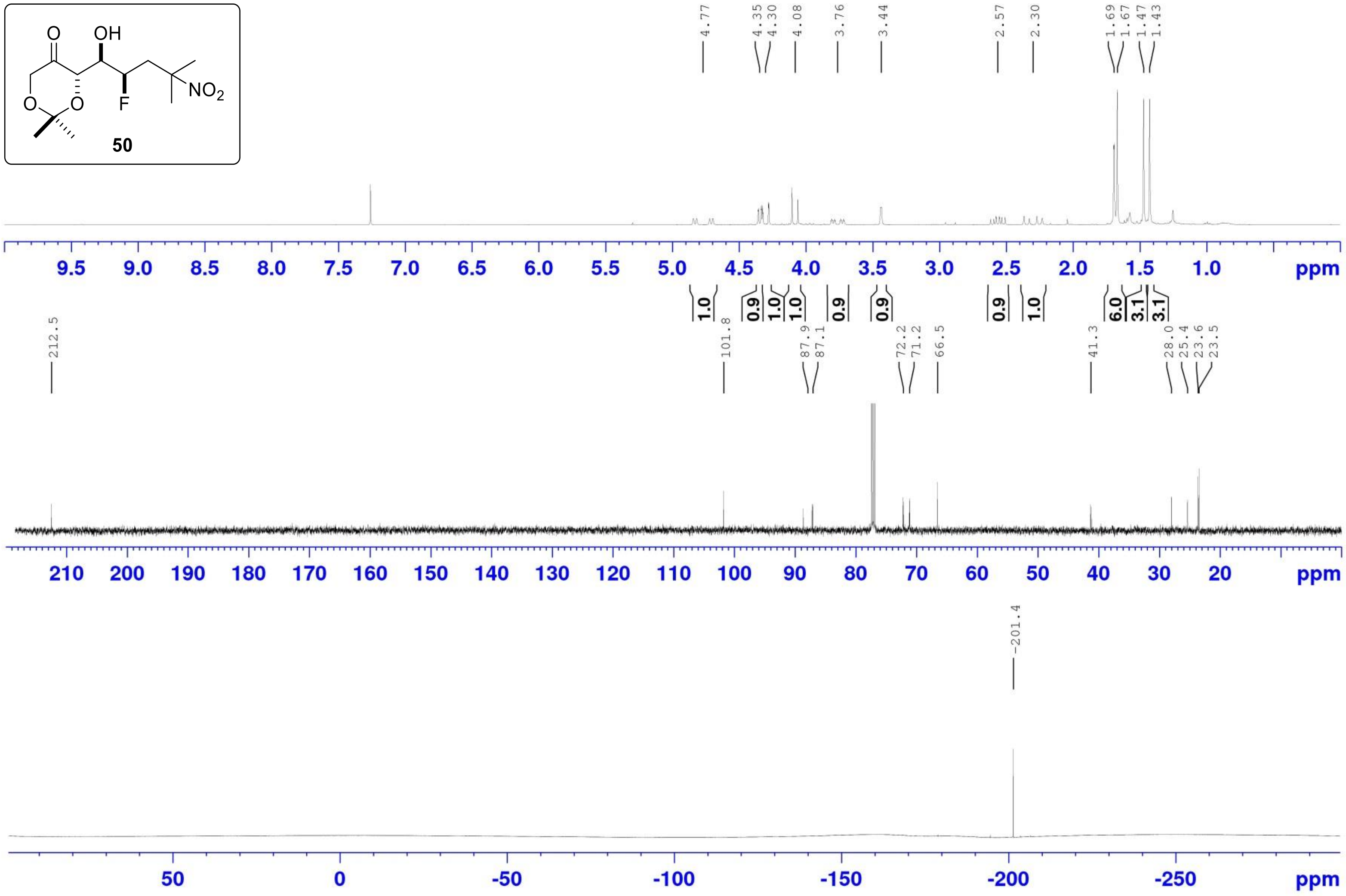


**Supplementary Figure 40**. NMR spectra of **50**.

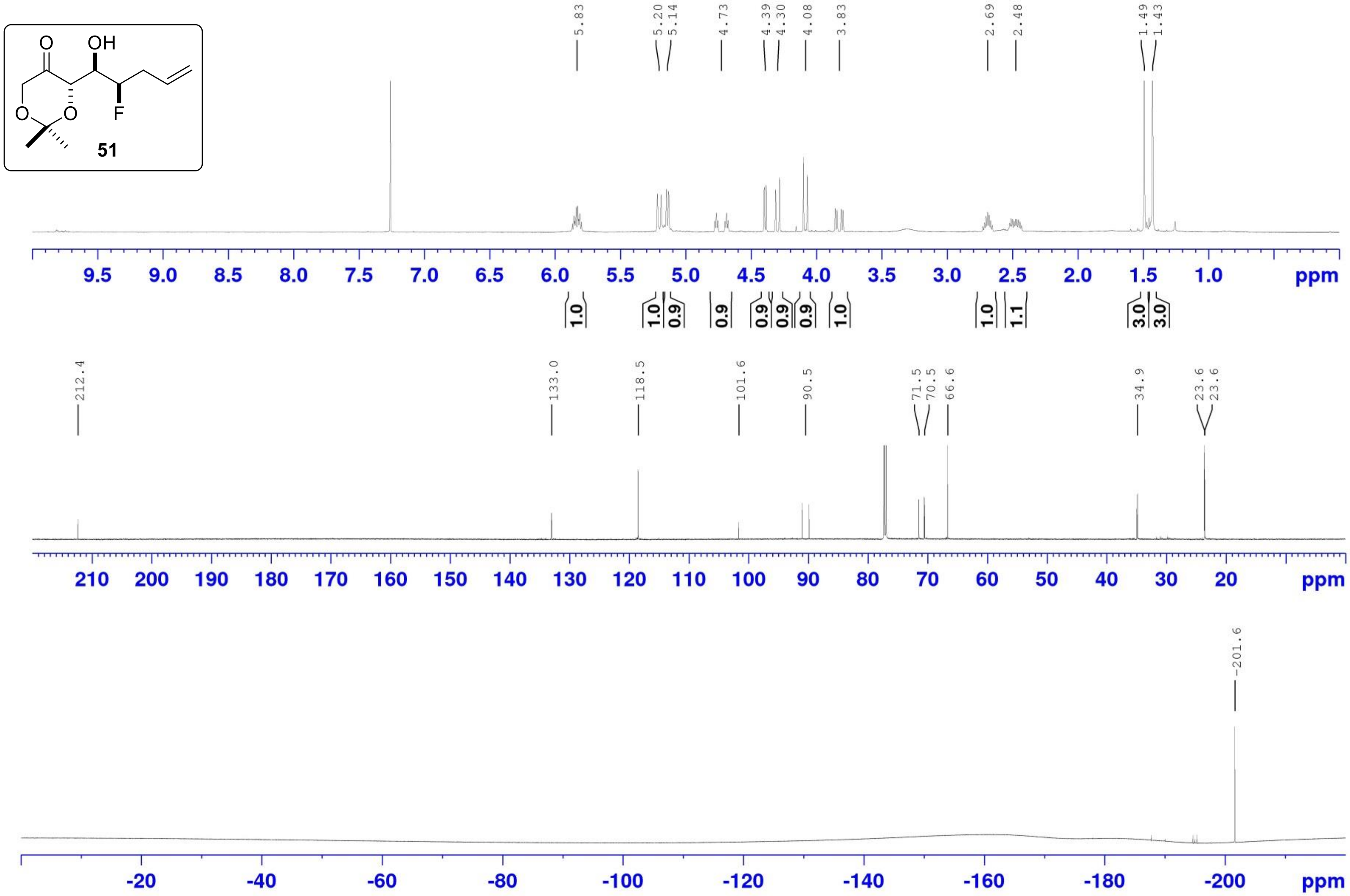


**Supplementary Figure 41**. NMR spectra of **51**.

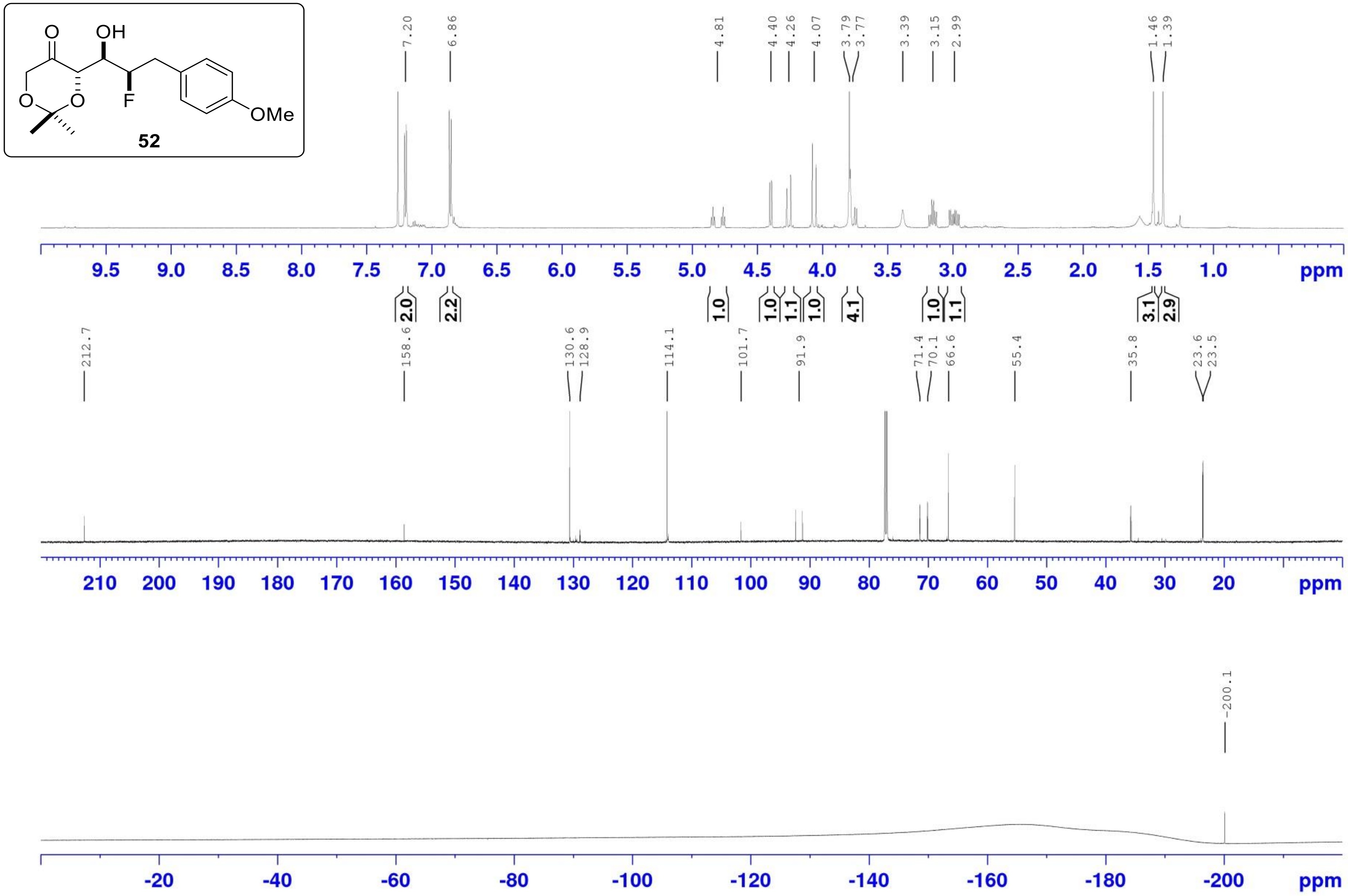


**Supplementary Figure 42**. NMR spectra of **52**.

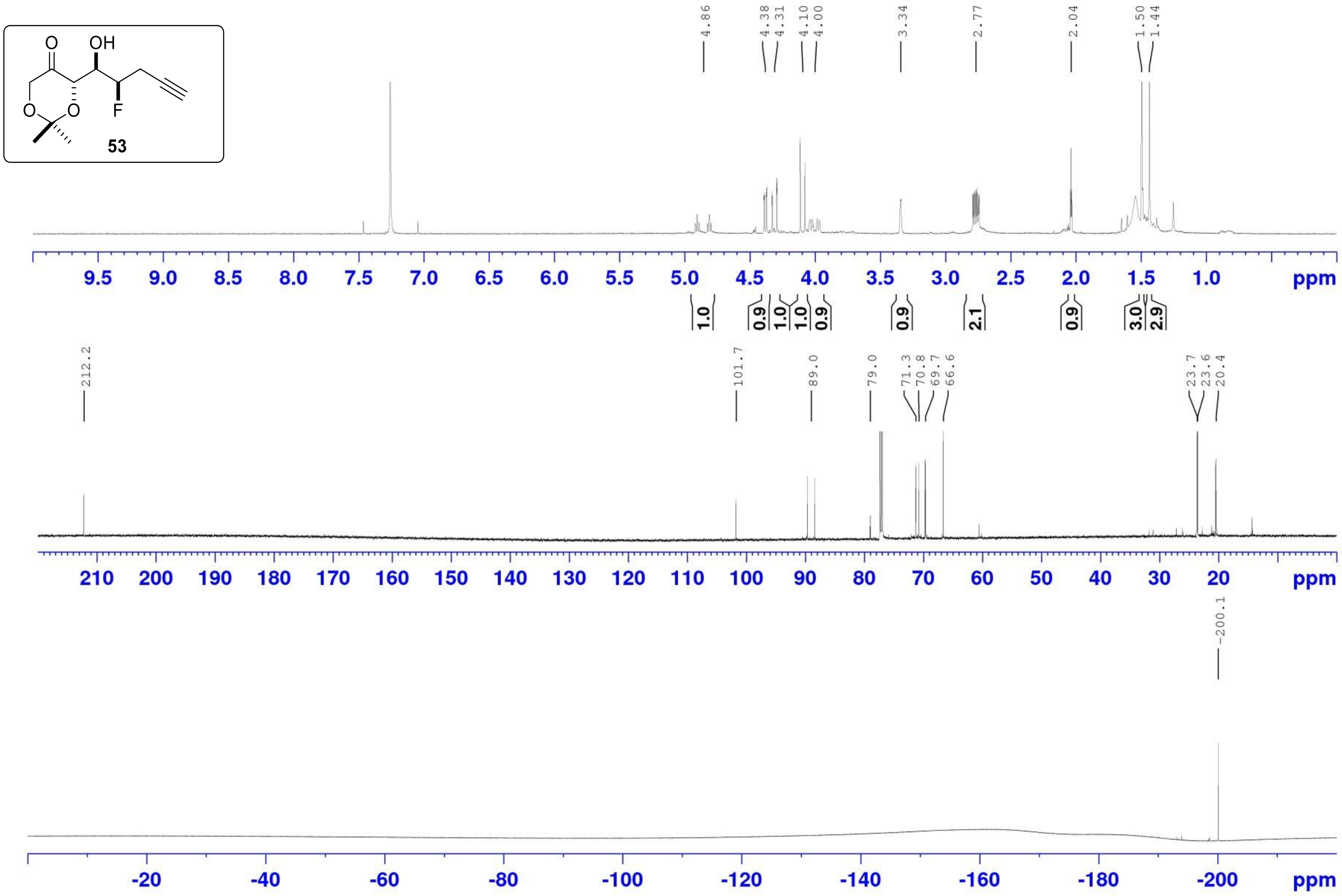


**Supplementary Figure 43**. NMR spectra of **53**.

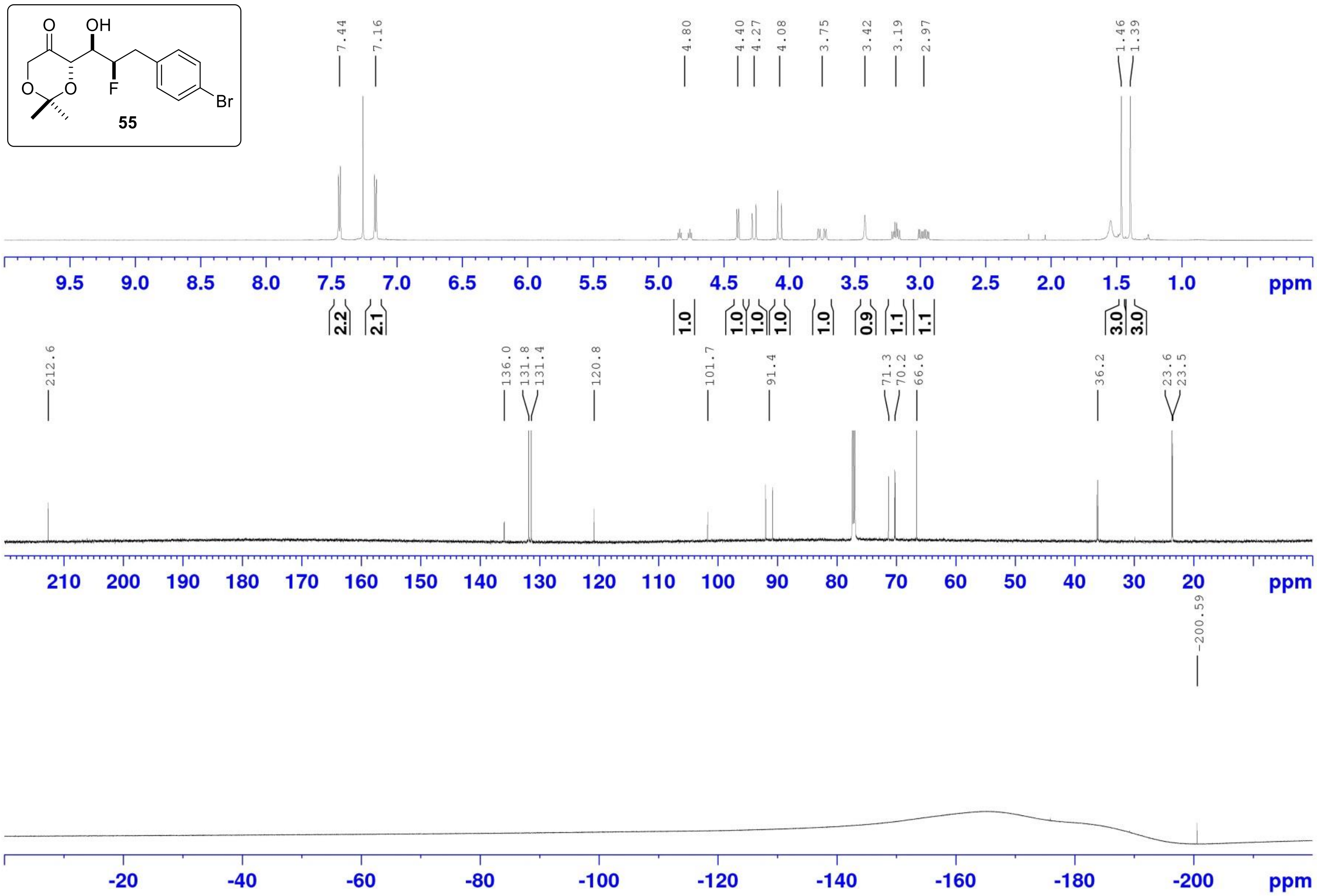


**Supplementary Figure 44**. NMR spectra of **55**.

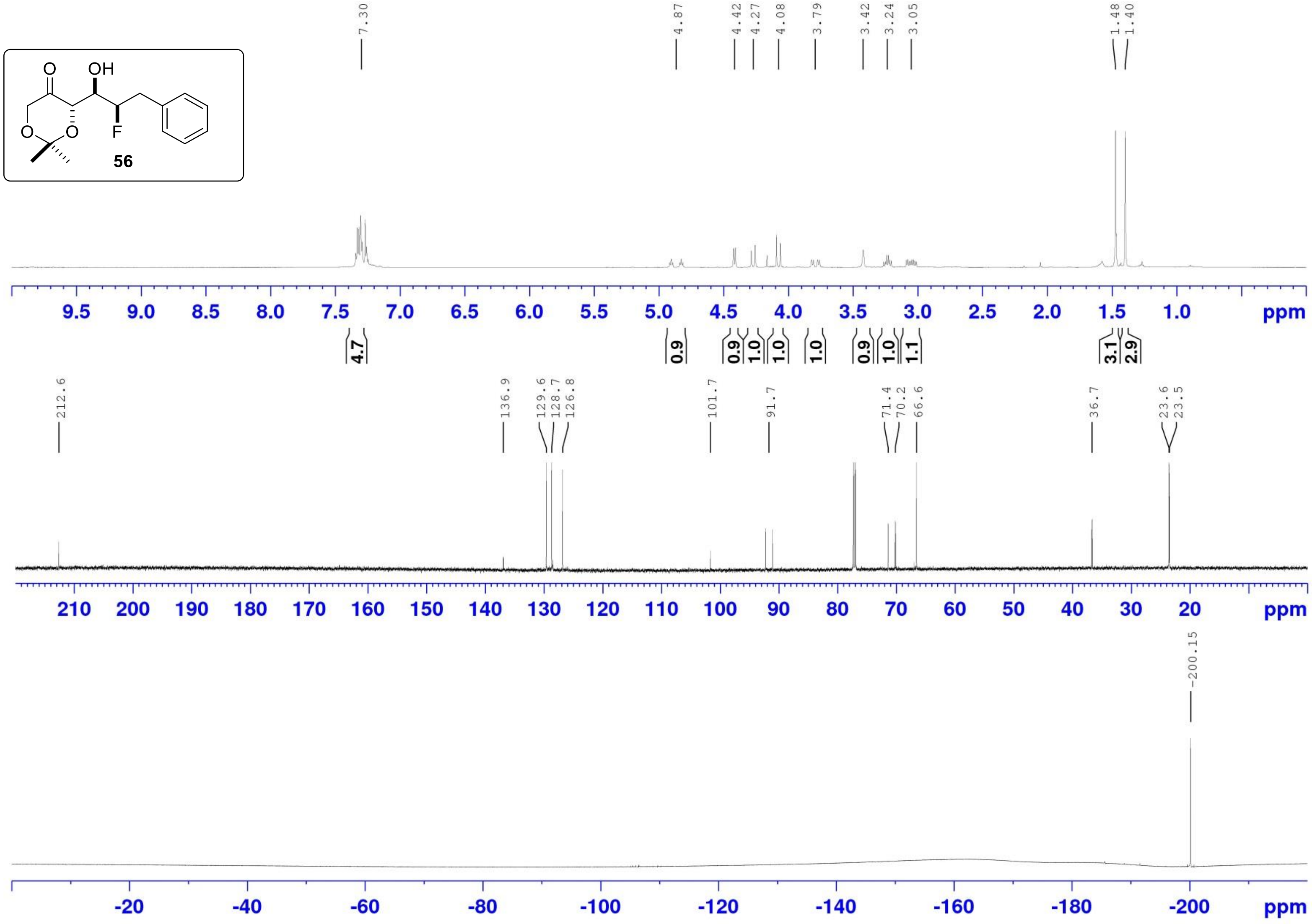


**Supplementary Figure 45**. NMR spectra of **56**.

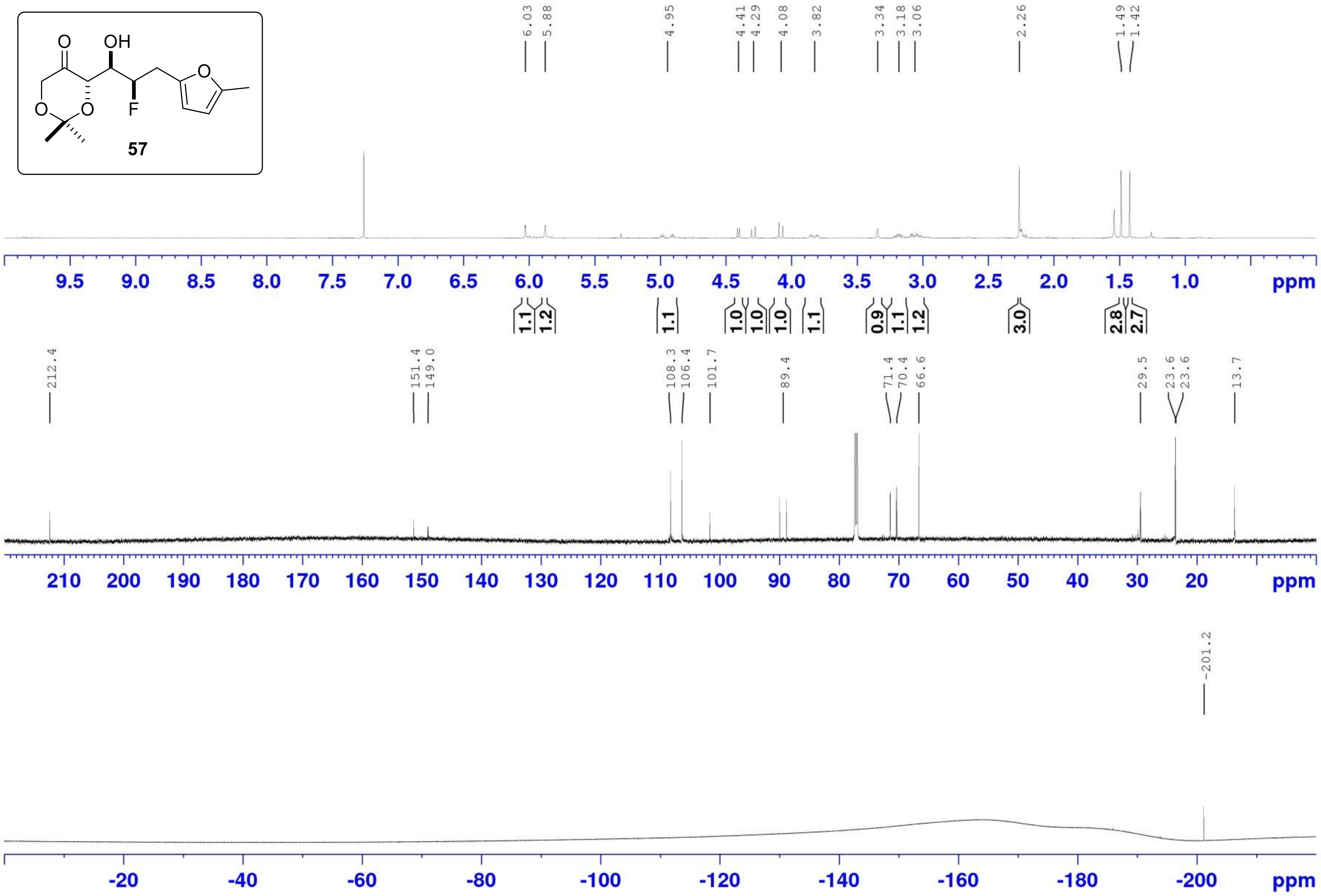


**Supplementary Figure 46**. NMR spectra of **57**.

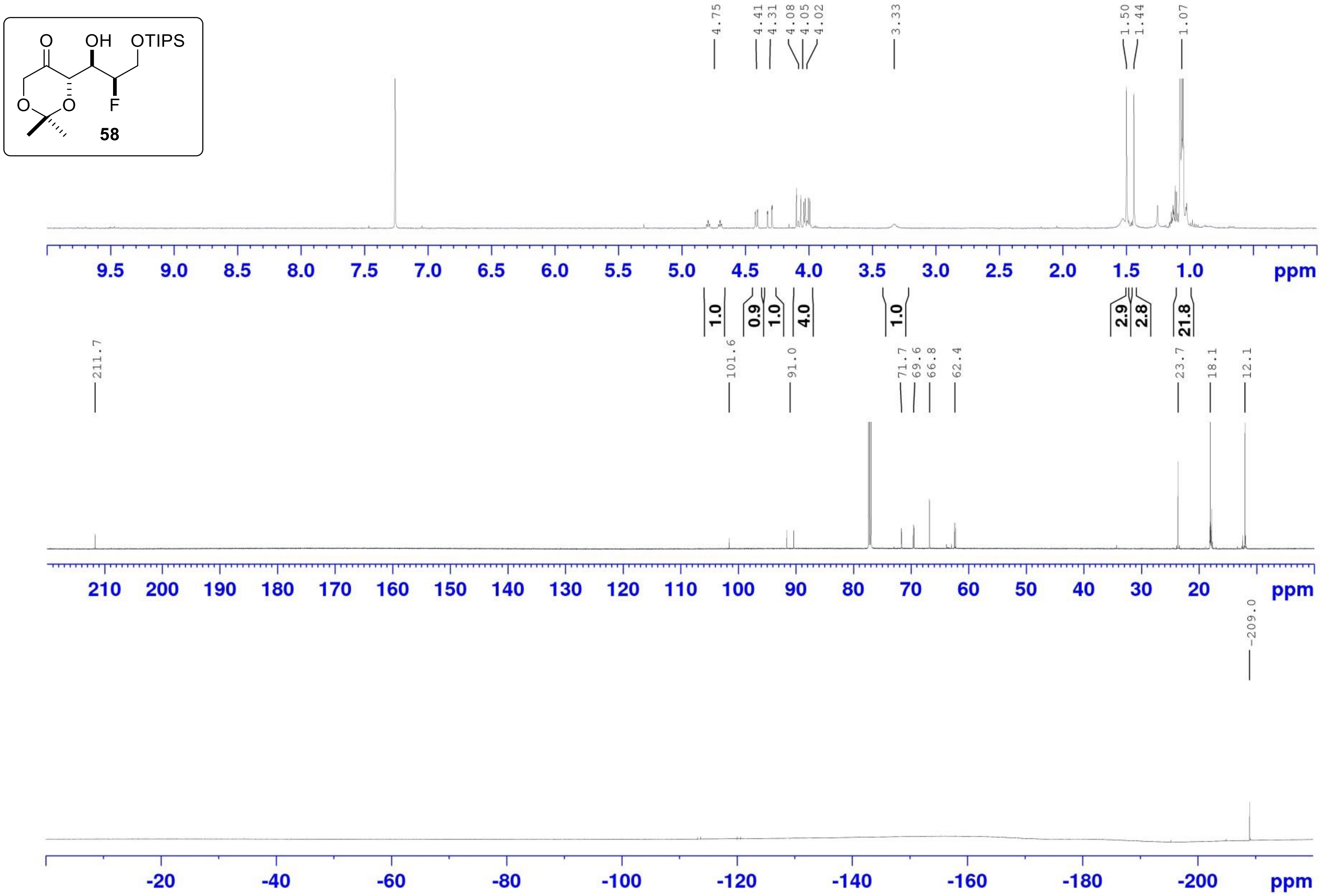


**Supplementary Figure 47**. NMR spectra of **58**.

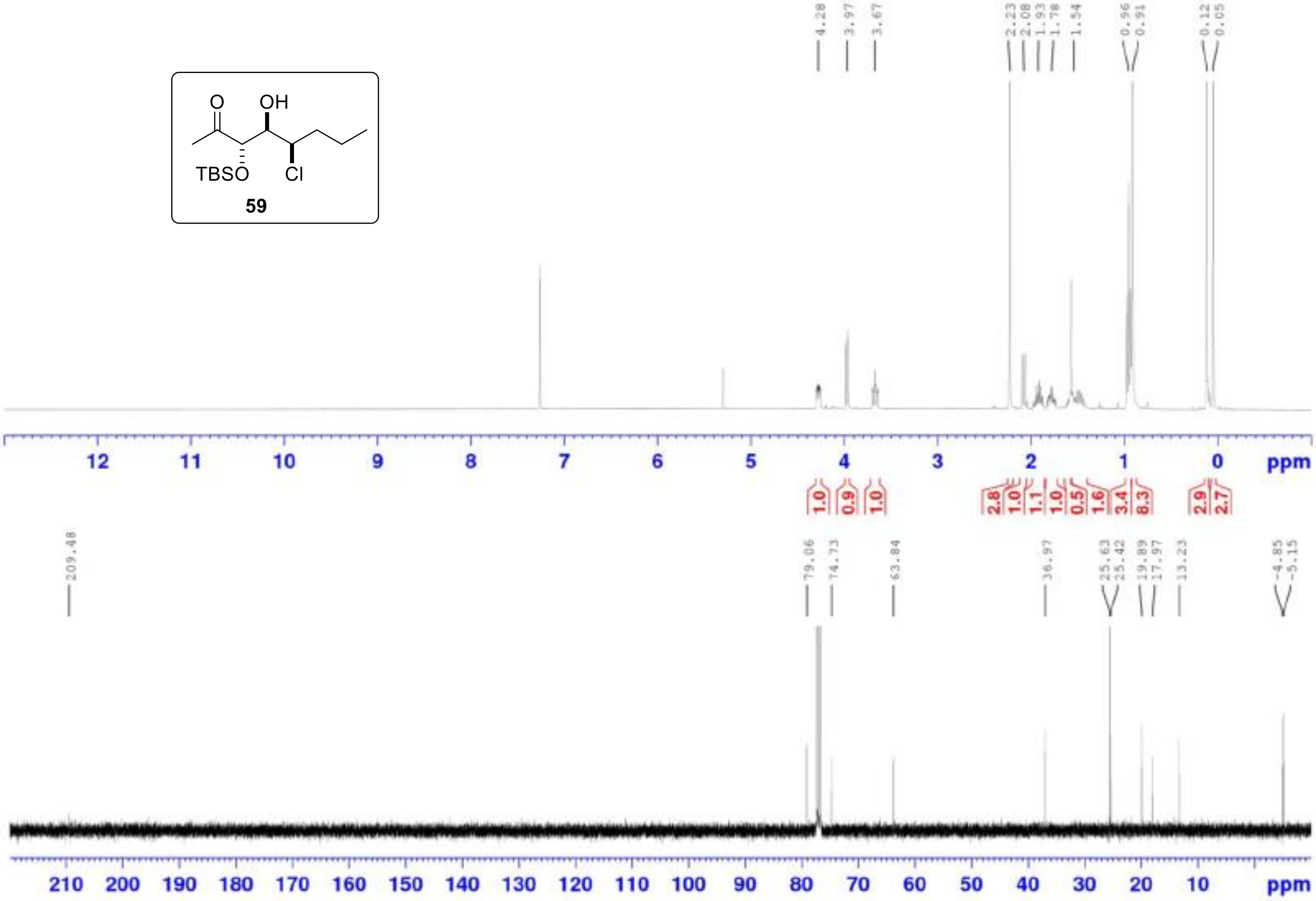


**Supplementary Figure 48**. NMR spectra of **59**.

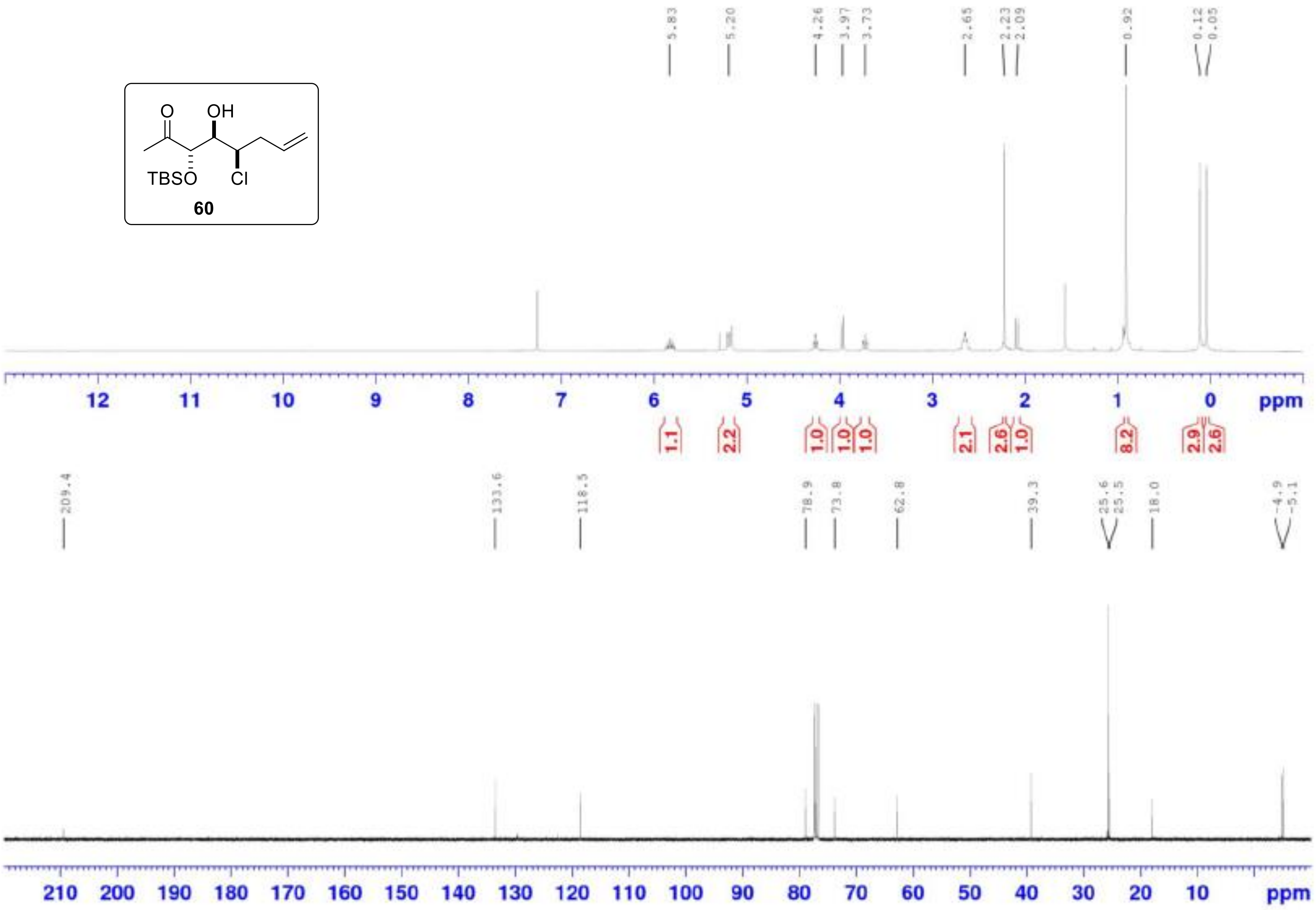


**Supplementary Figure 49**. NMR spectra of **60**.

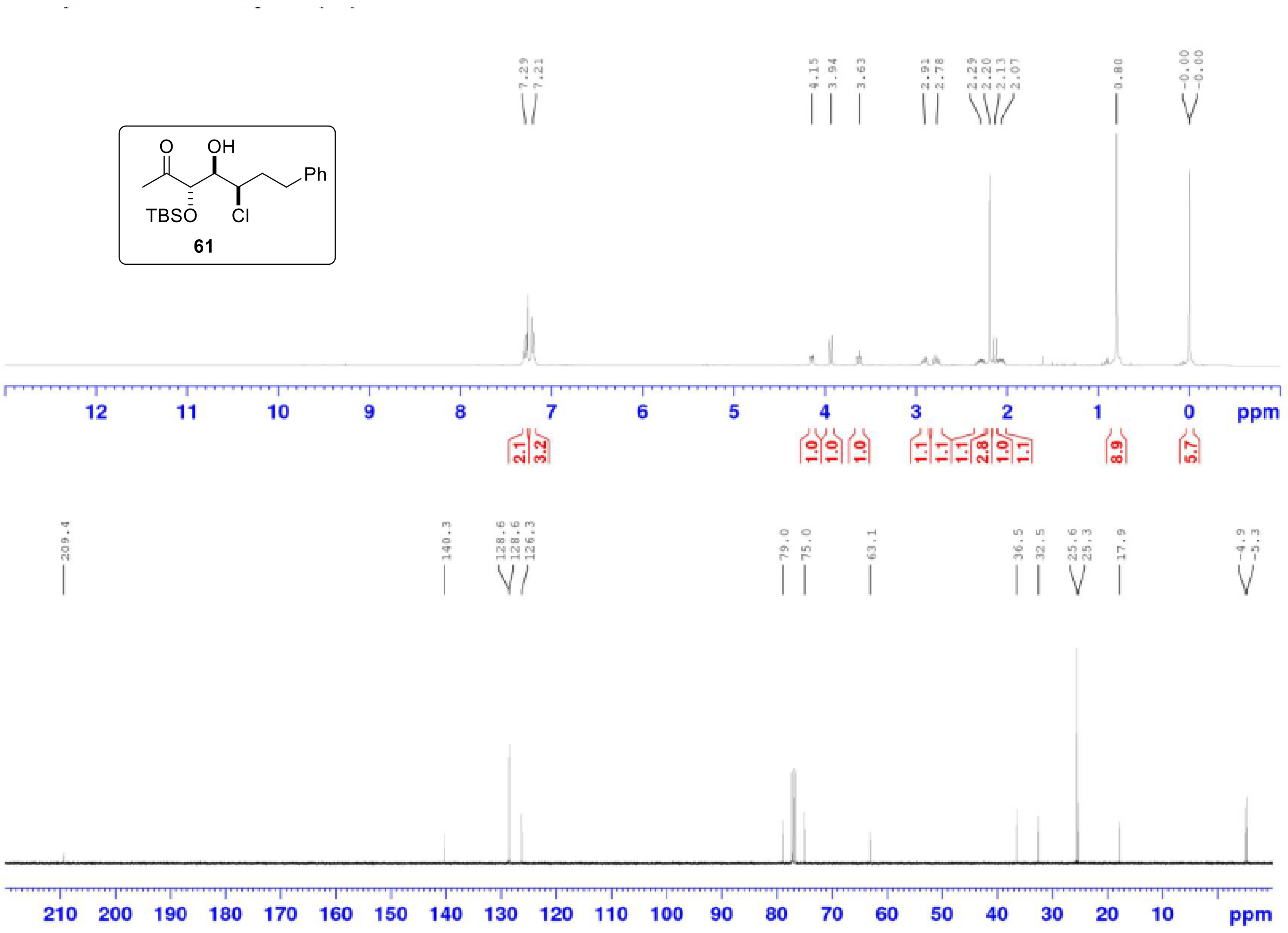


**Supplementary Figure 50**. NMR spectra of **61**.

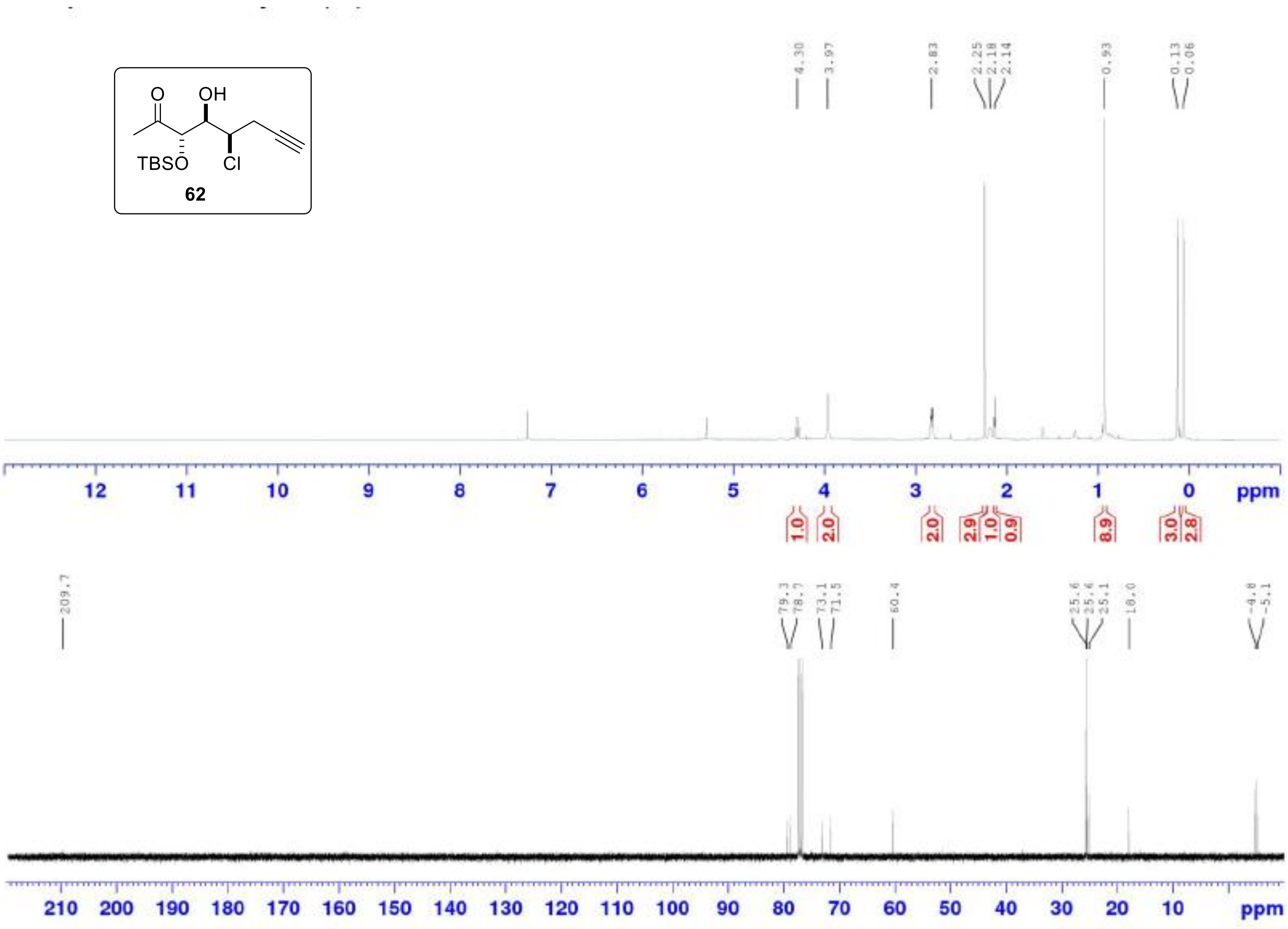


**Supplementary Figure 51**. NMR spectra of **62**.

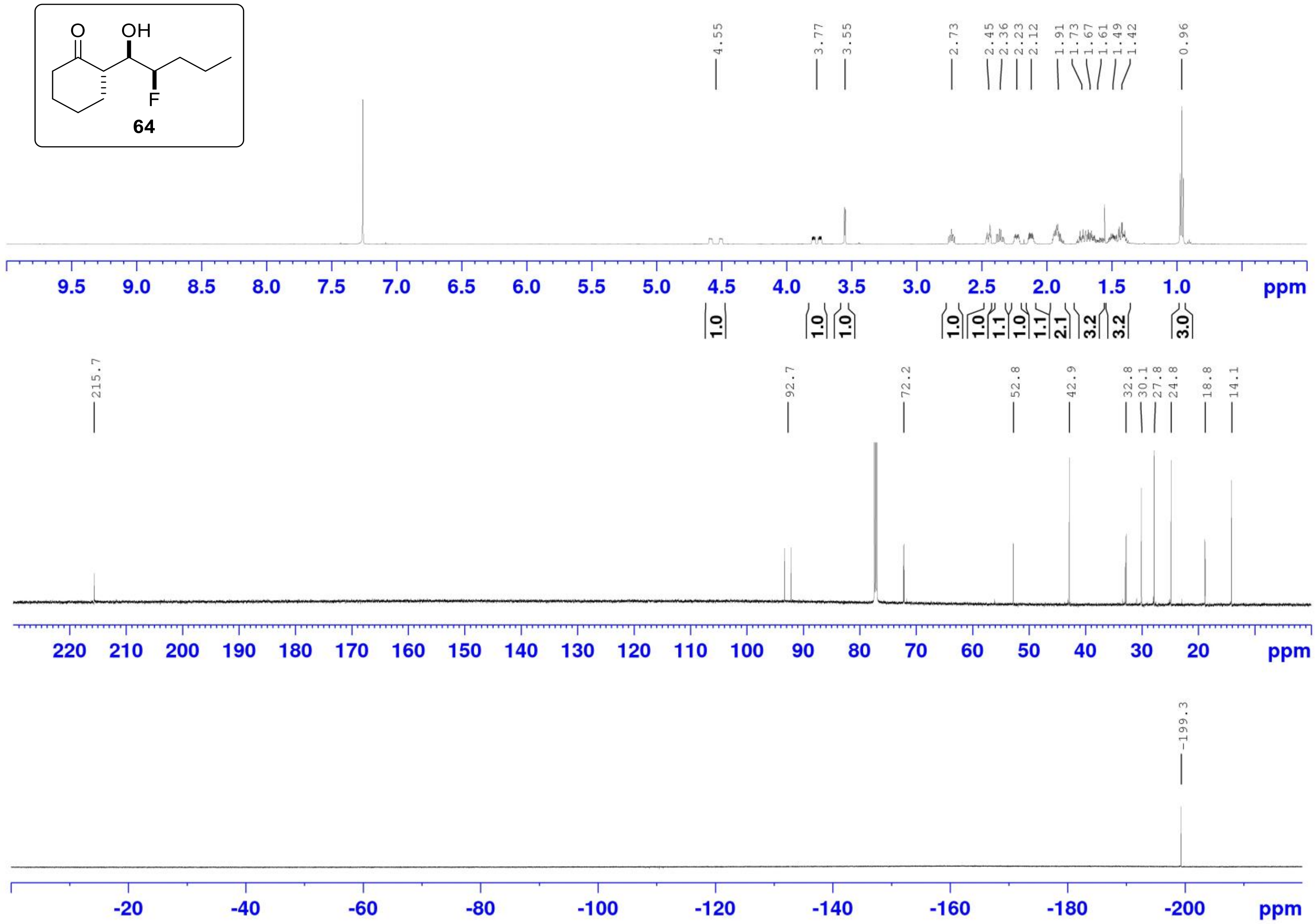

**Supplementary Figure 52**. NMR spectra of **64**.

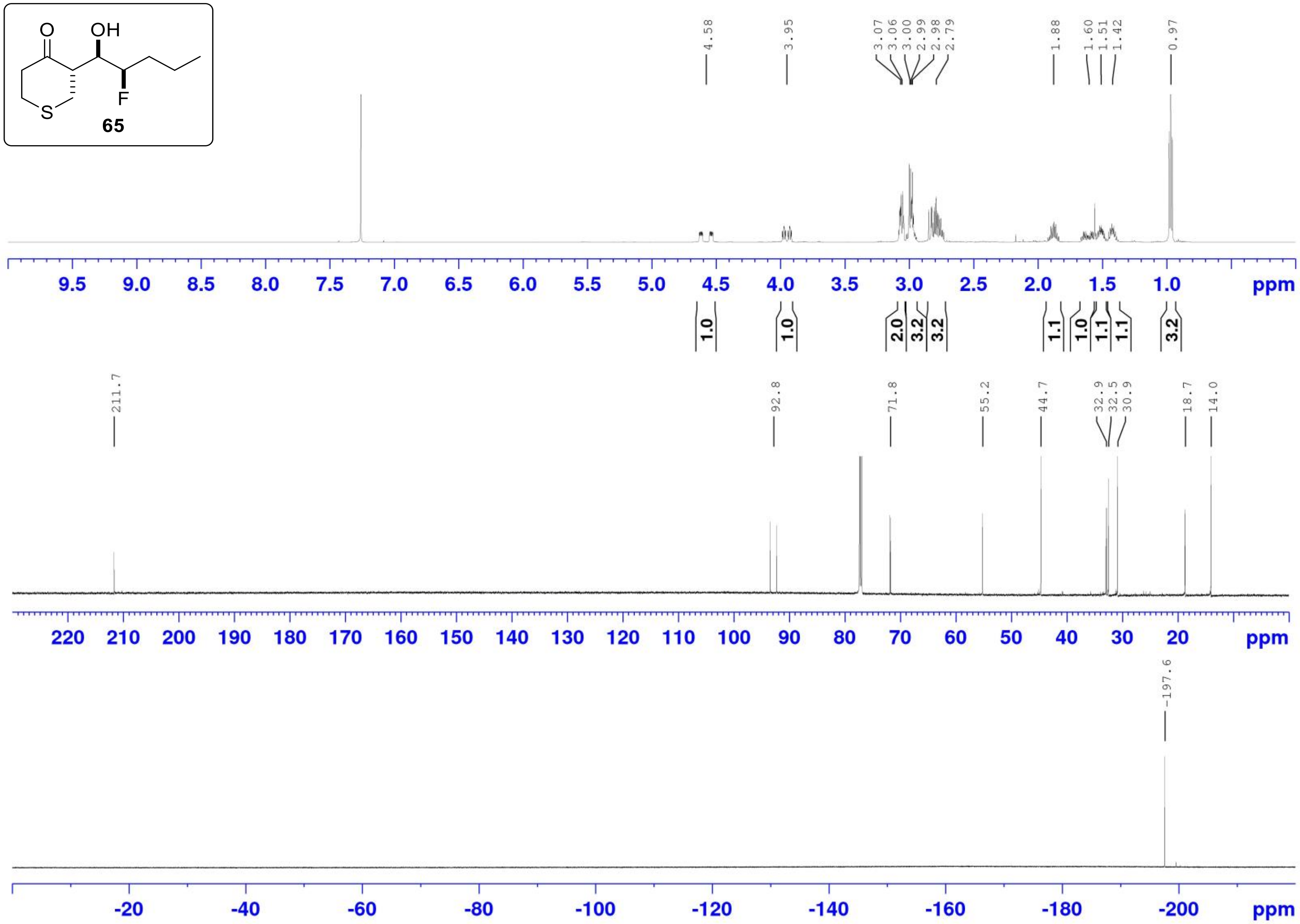


**Supplementary Figure 53**. NMR spectra of **65**.

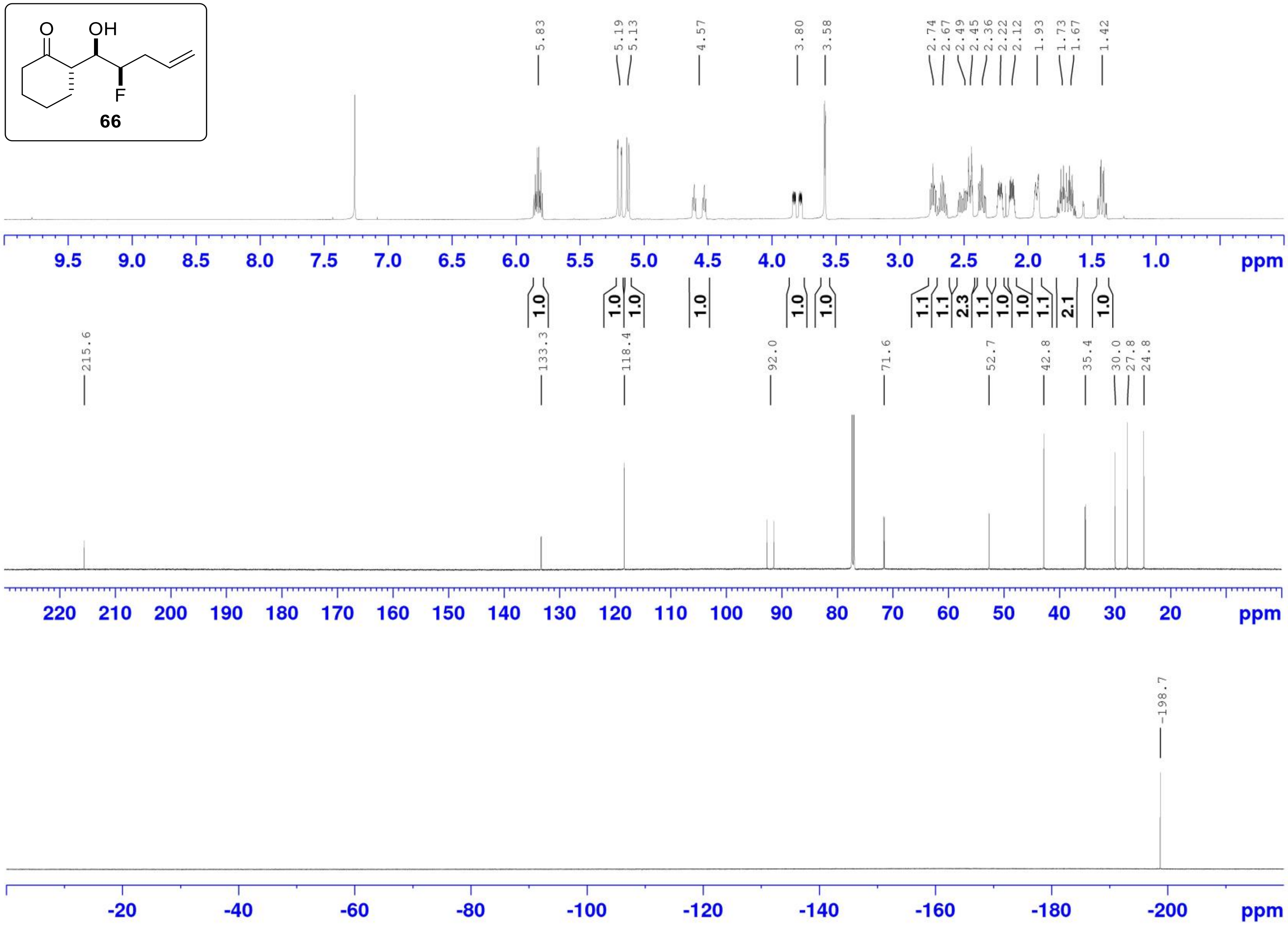


**Supplementary Figure 54**. NMR spectra of **66**.

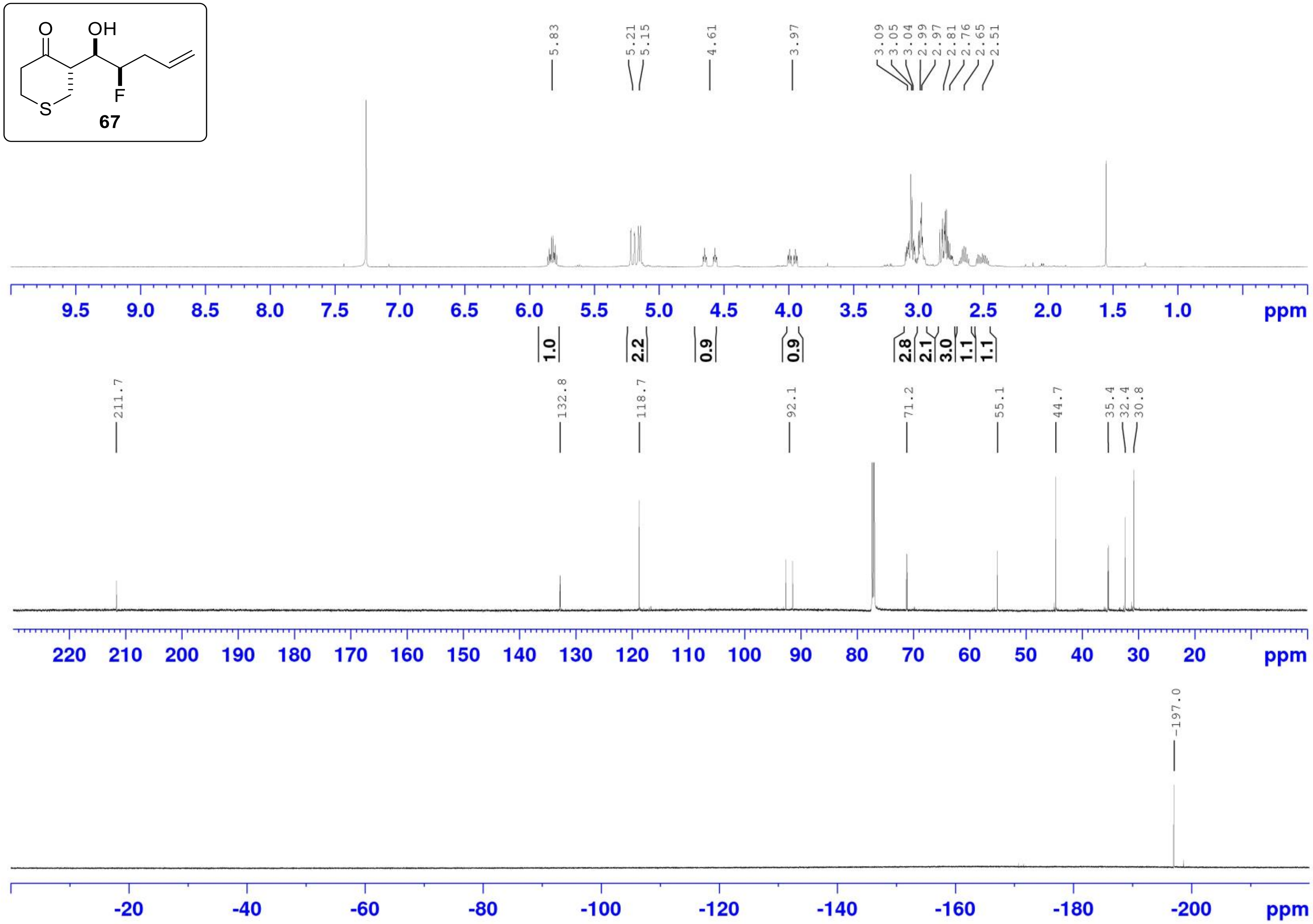


**Supplementary Figure 55**. NMR spectra of **67**.

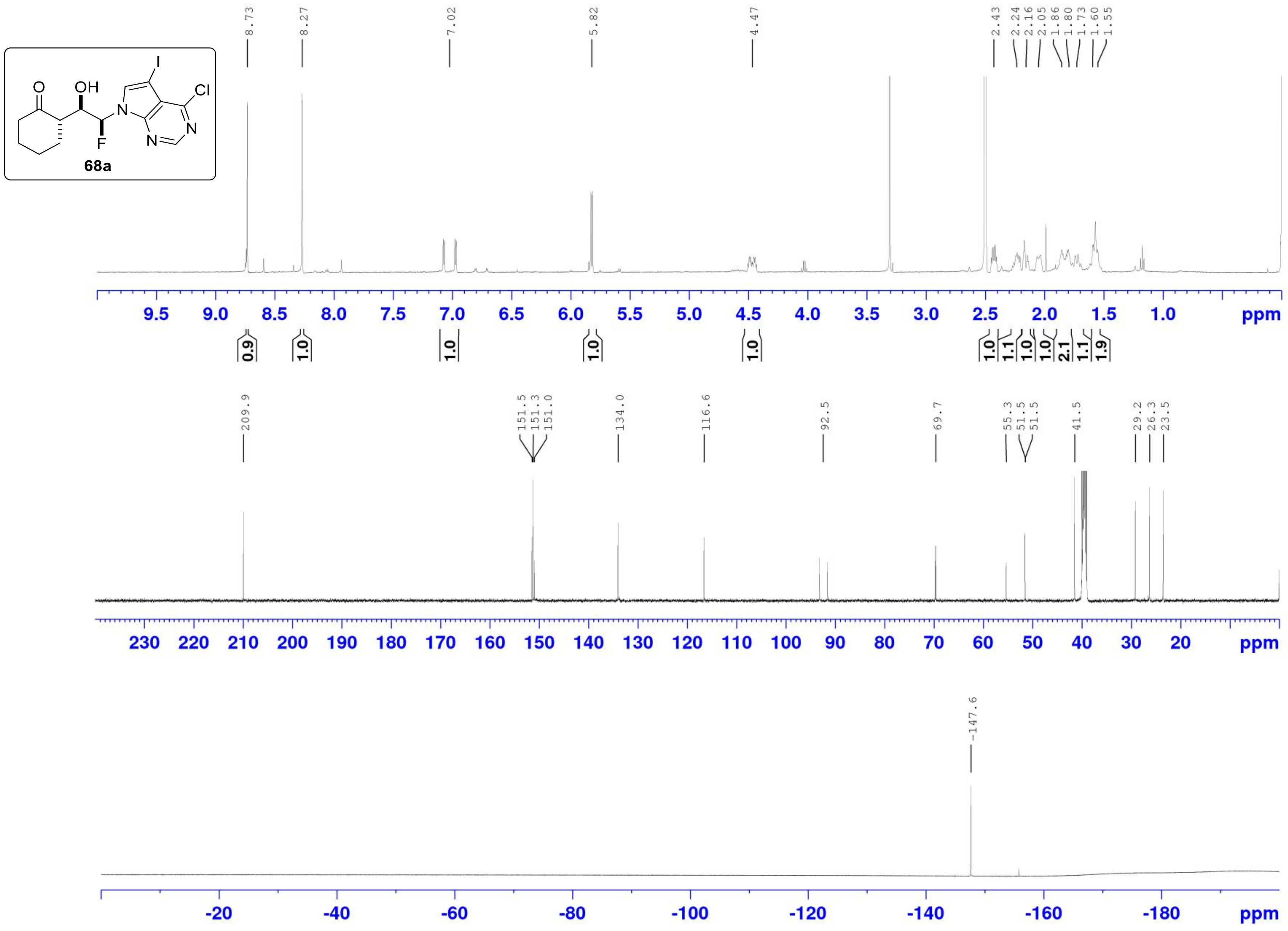

**Supplementary Figure 56**. NMR spectra of **68a**.

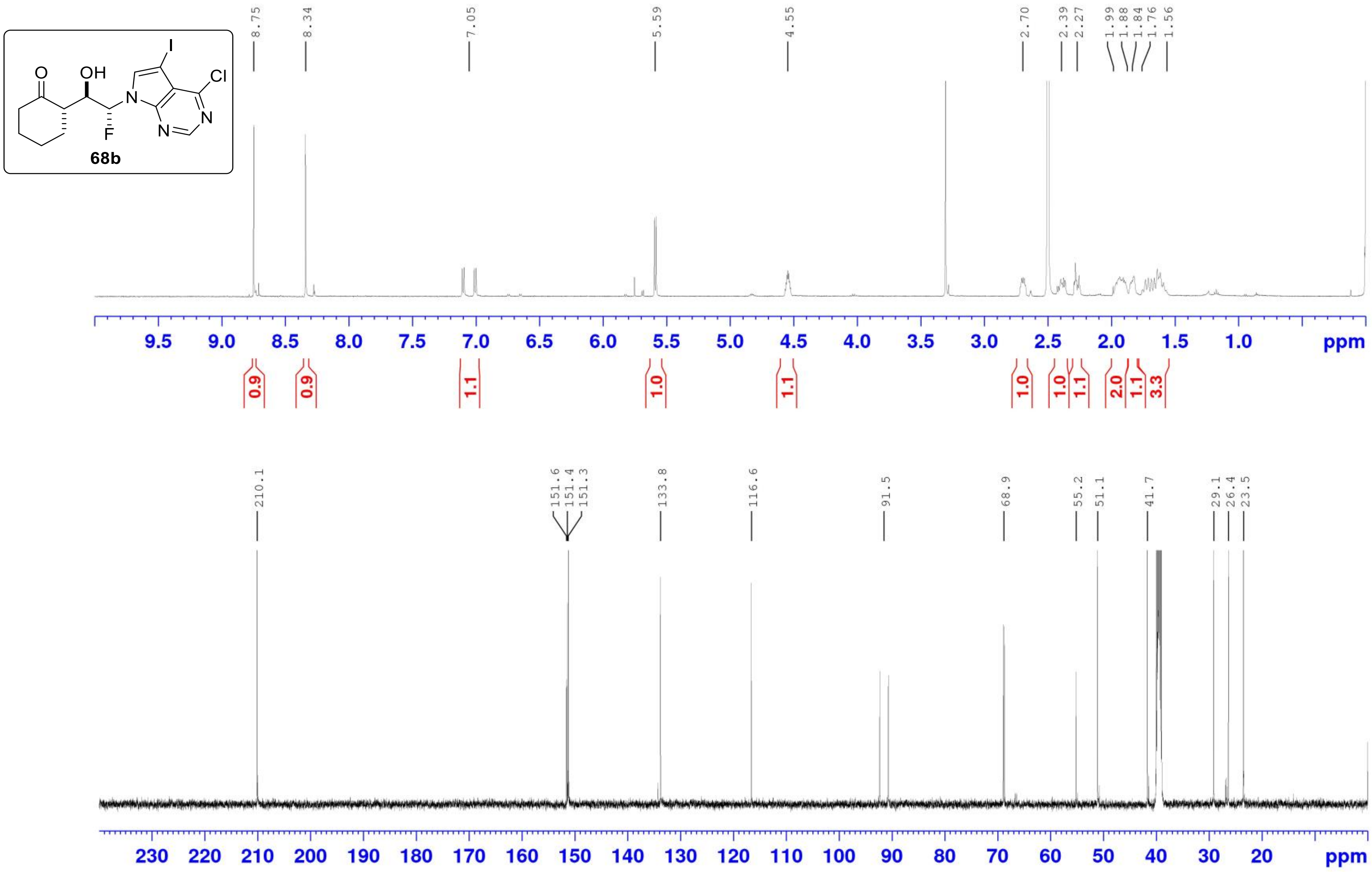


**Supplementary Figure 57**. NMR spectra of **68b**.

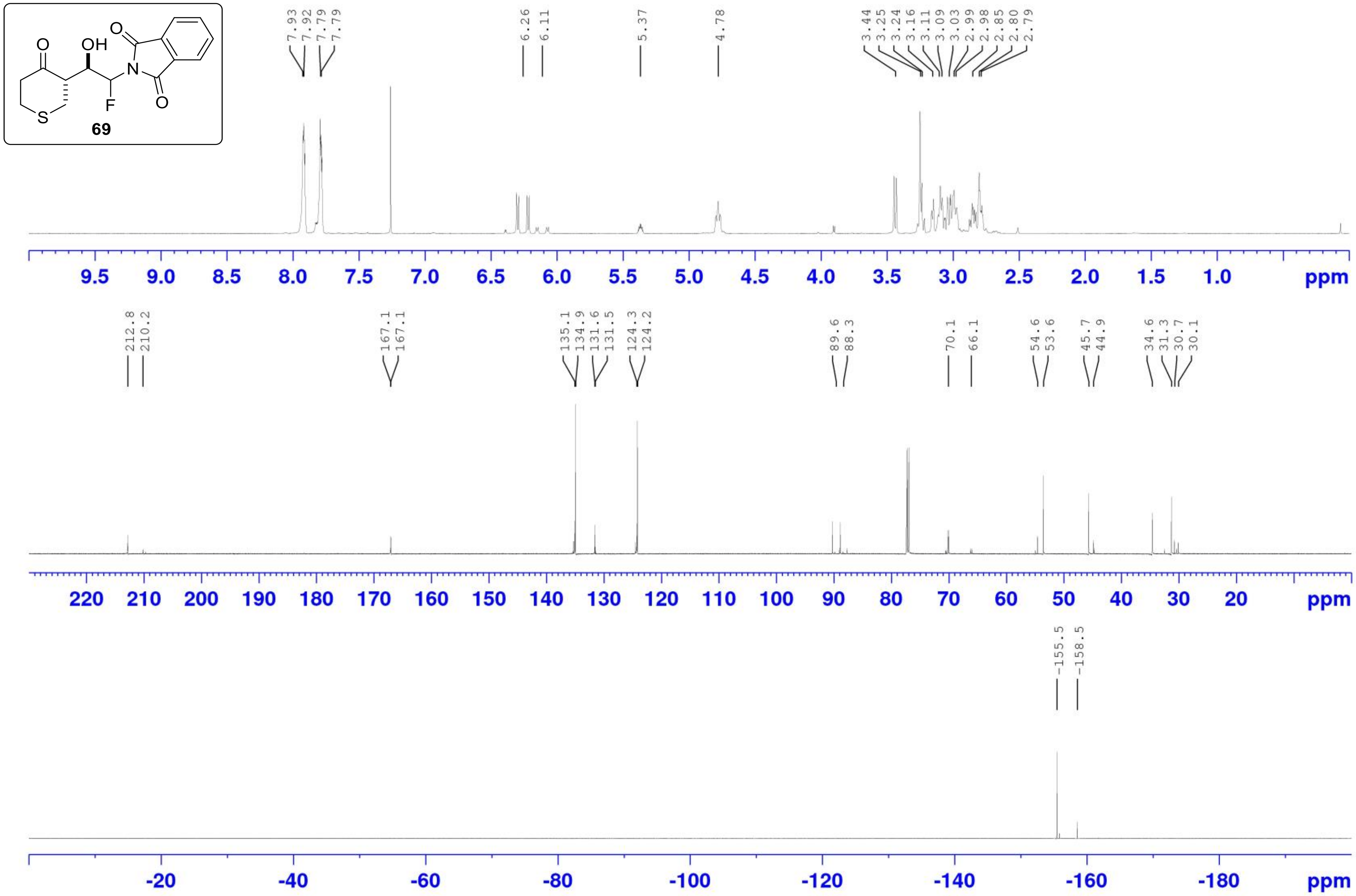


**Supplementary Figure 58**. NMR spectra of **69**.

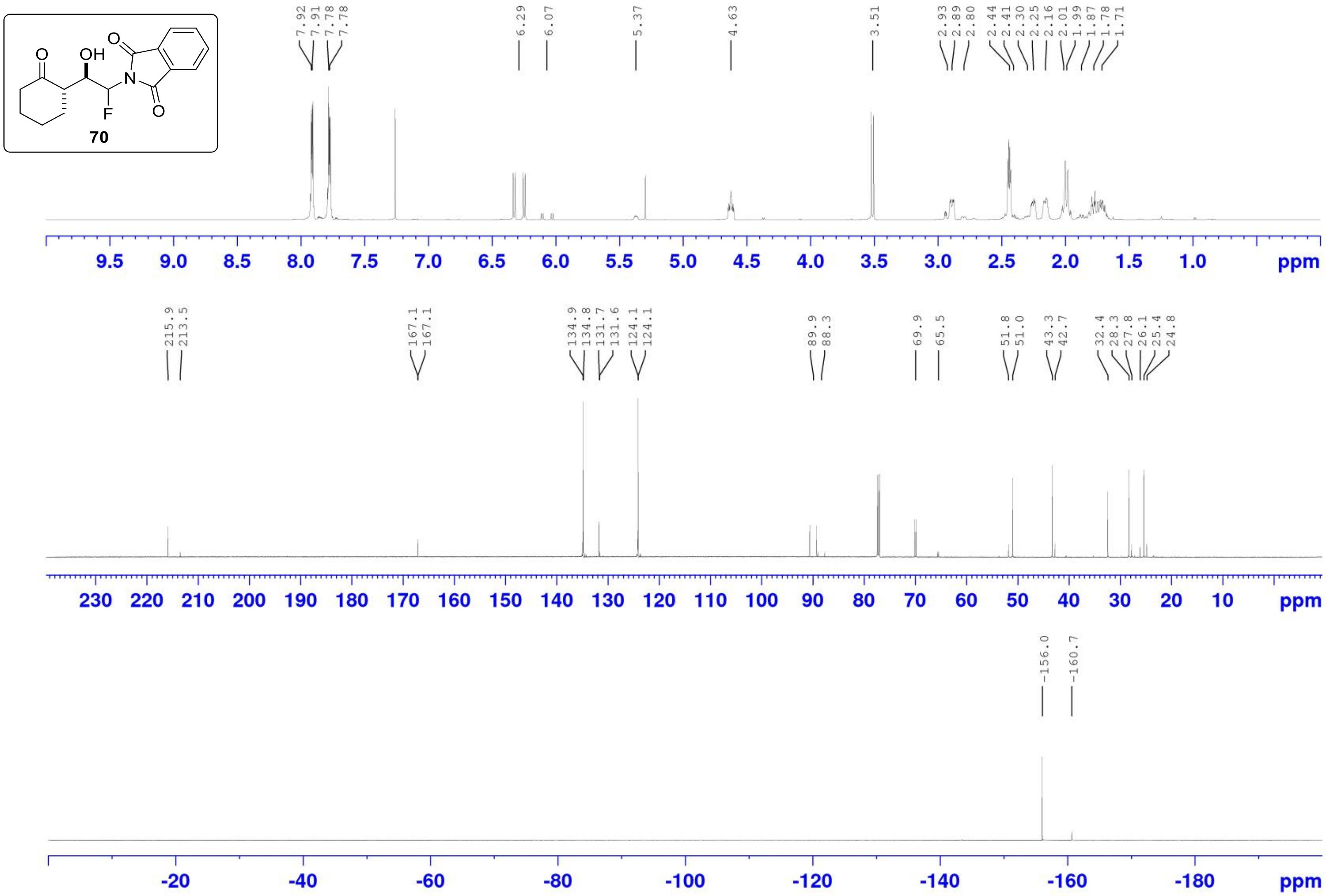


**Supplementary Figure 59**. NMR spectra of **70**.

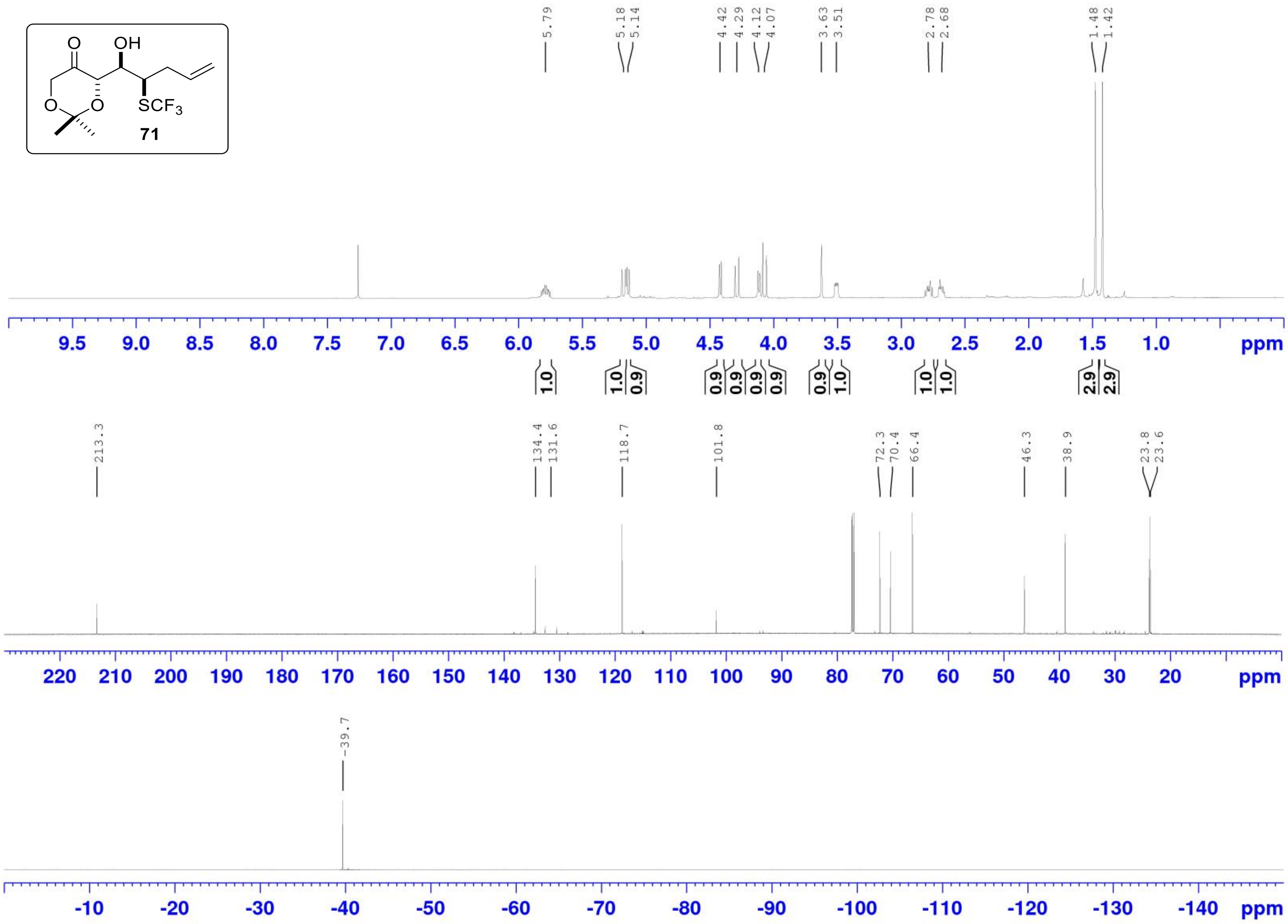


Supplementary Figure 60. NMR spectra of **71**.

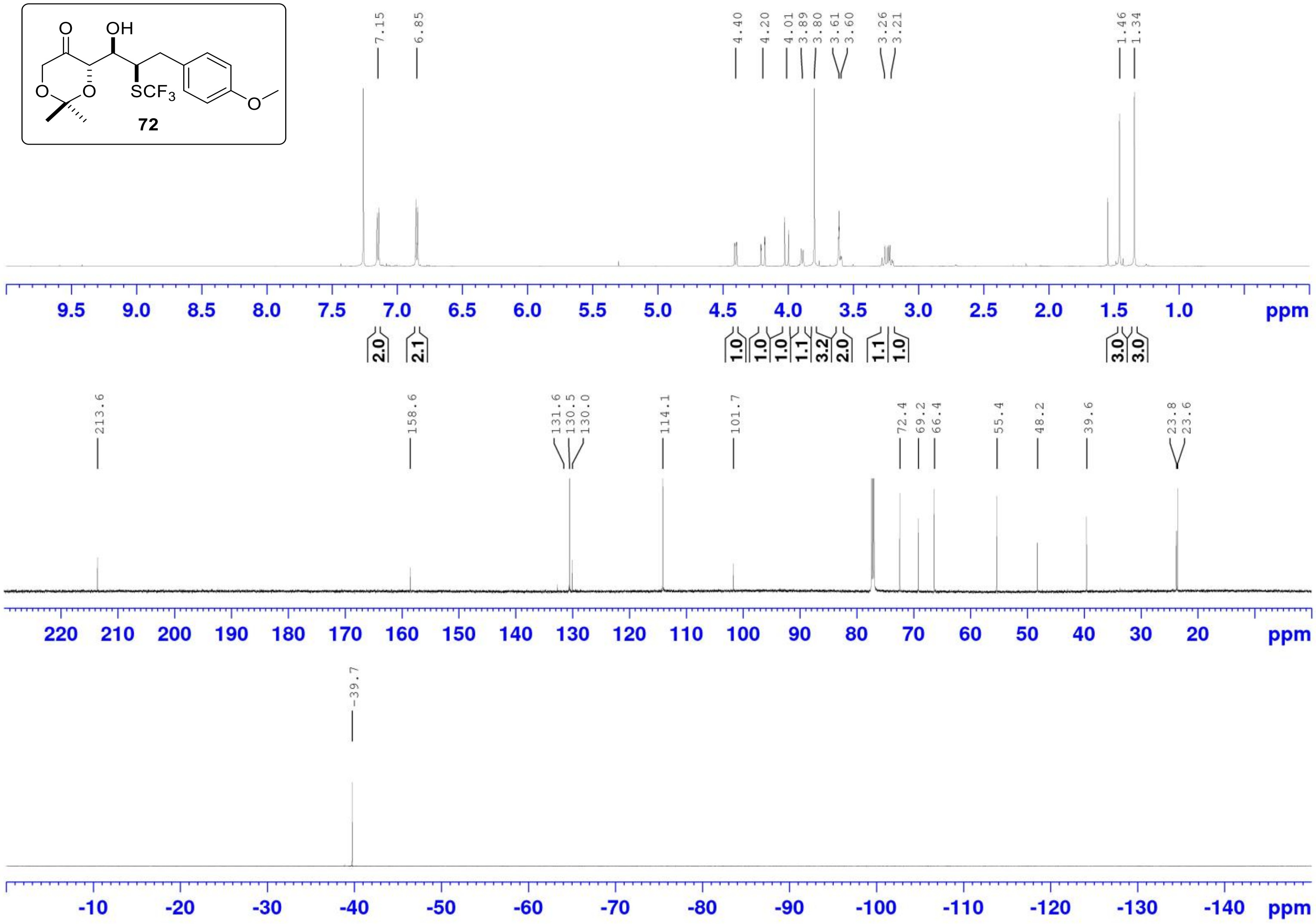

**Supplementary Figure 61**. NMR spectra of **72**.

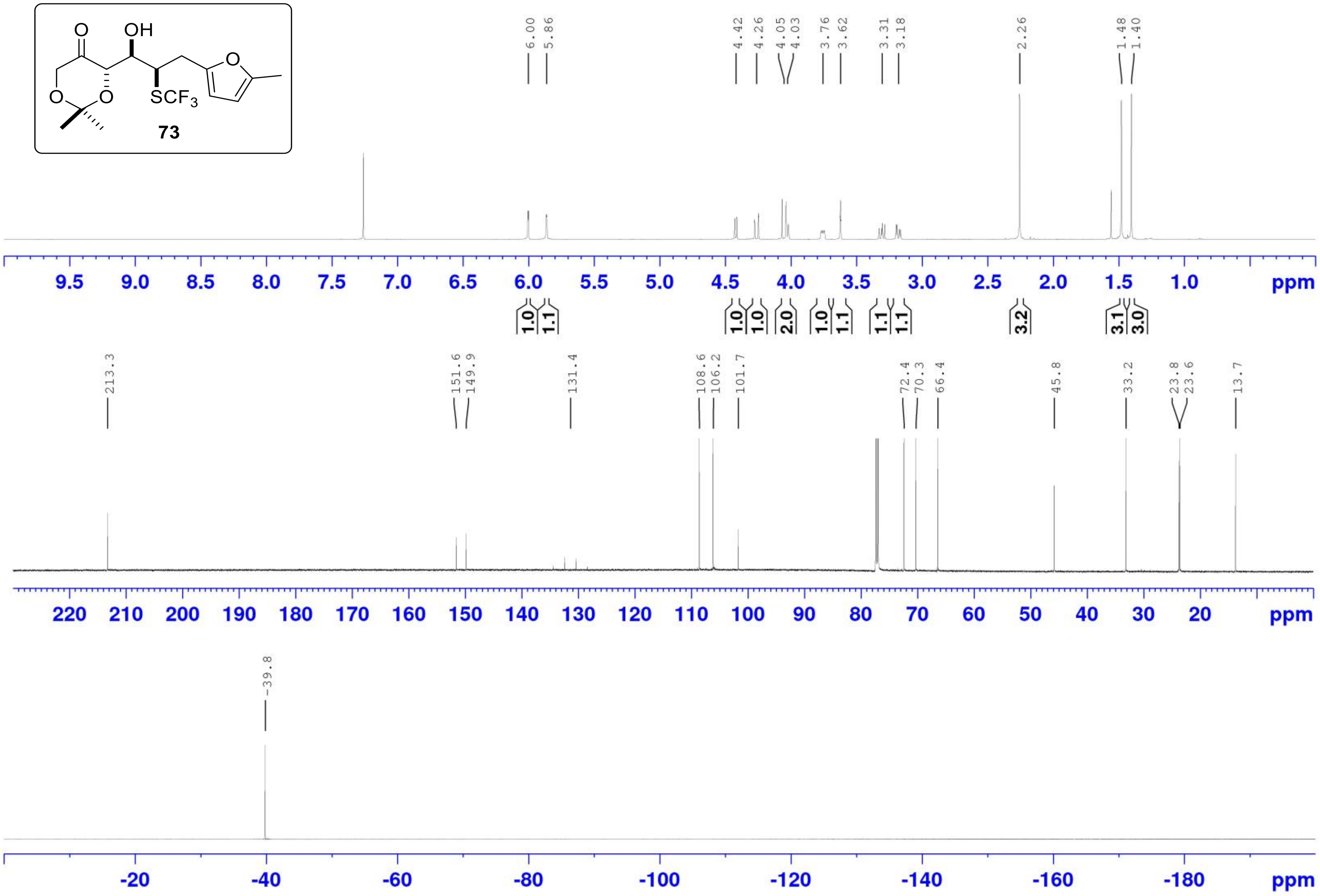


**Supplementary Figure 62**. NMR spectra of **73**.

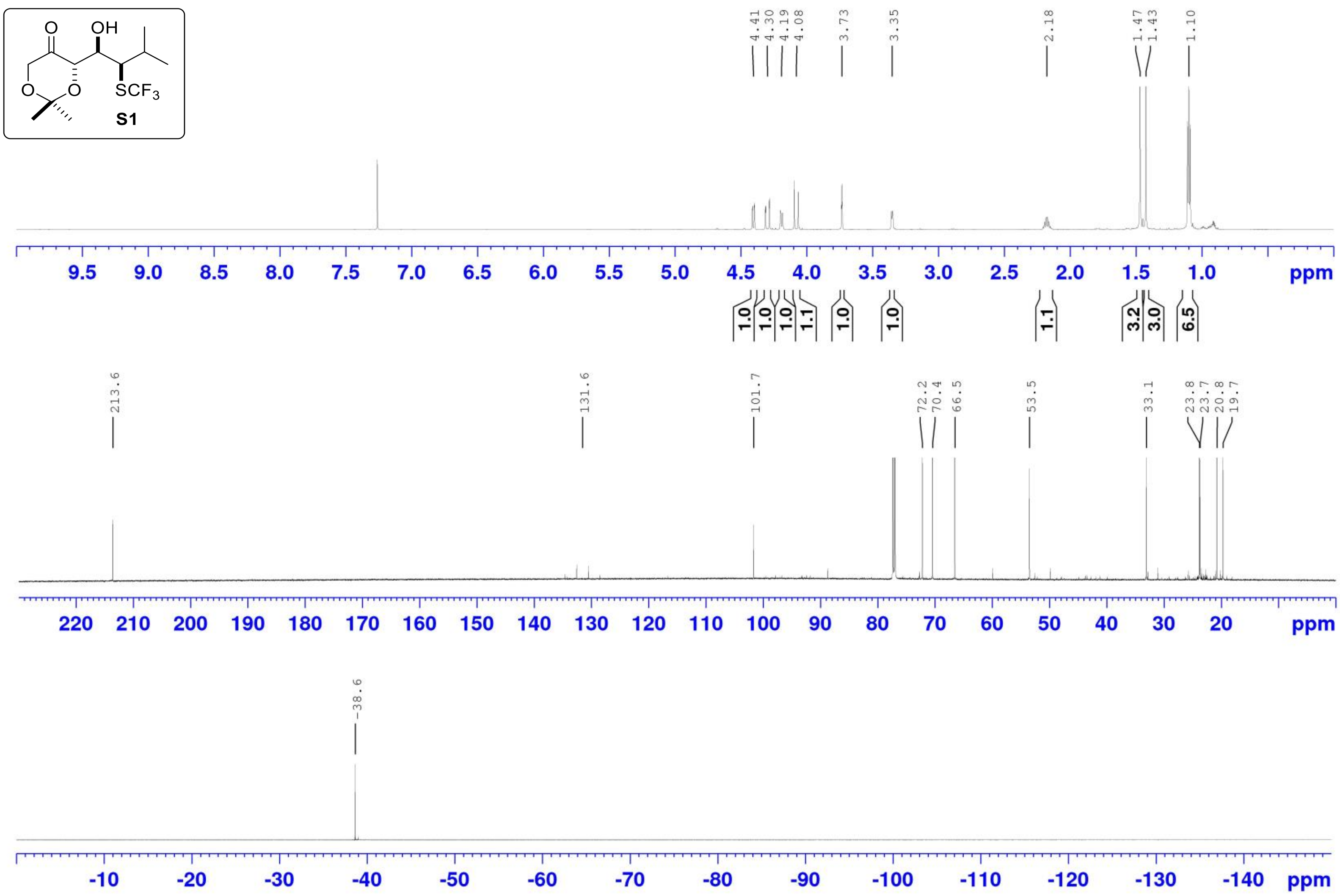


**Supplementary Figure 63**. NMR spectra of **S1**.

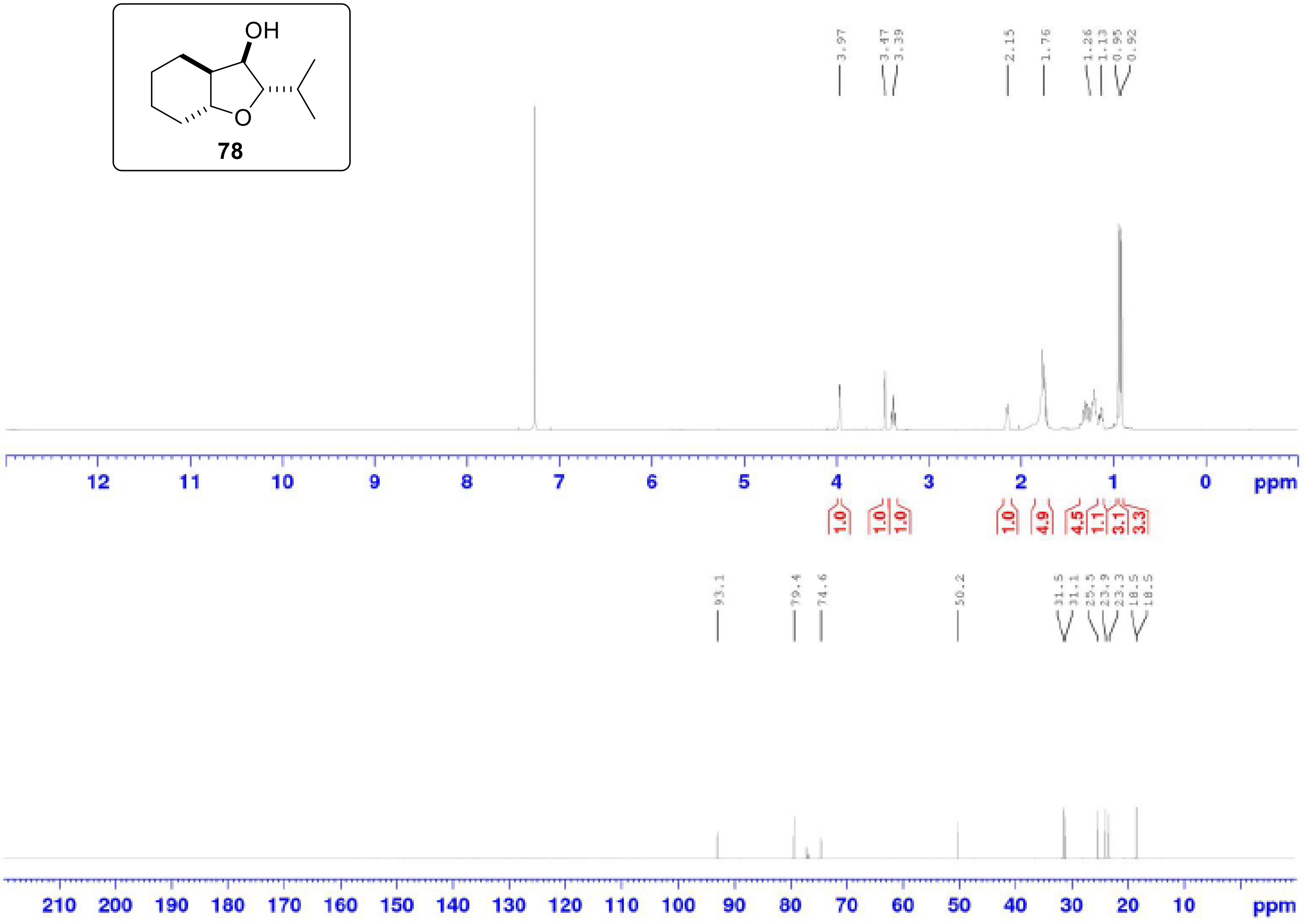


**Supplementary Figure 64**. NMR spectra of **78**.

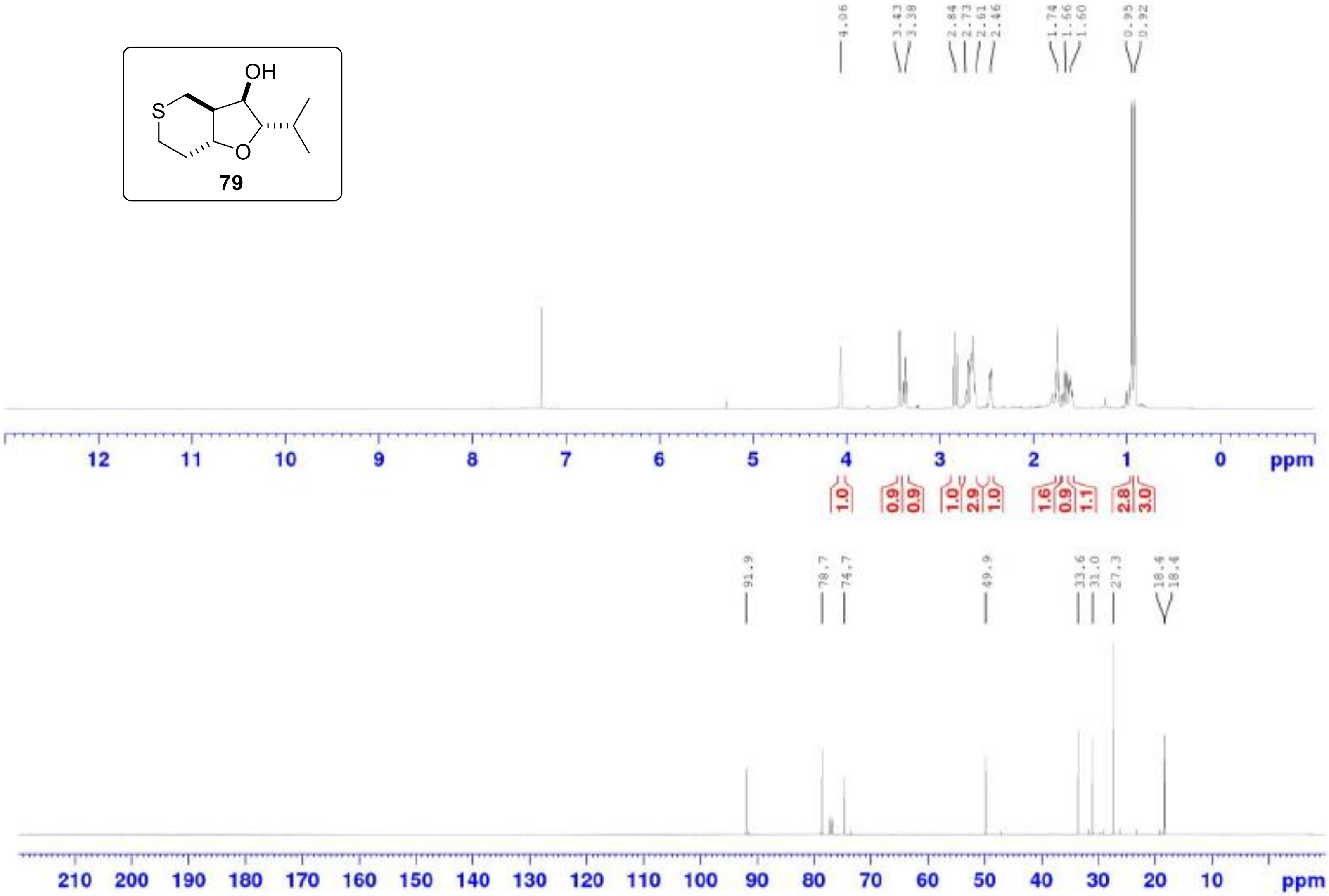


**Supplementary Figure 65**. NMR spectra of **79**.

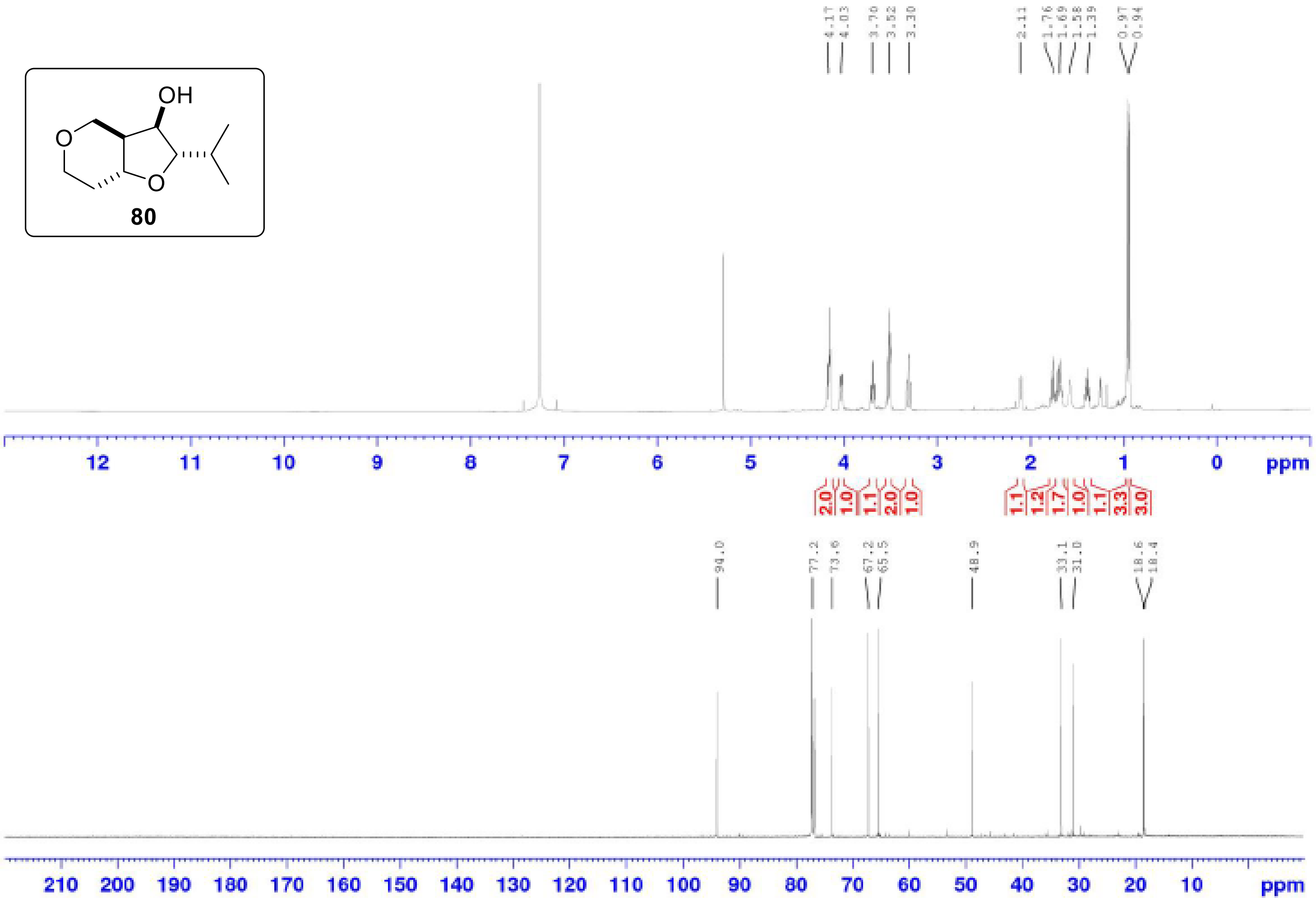


**Supplementary Figure 66**. NMR spectra of **80**.

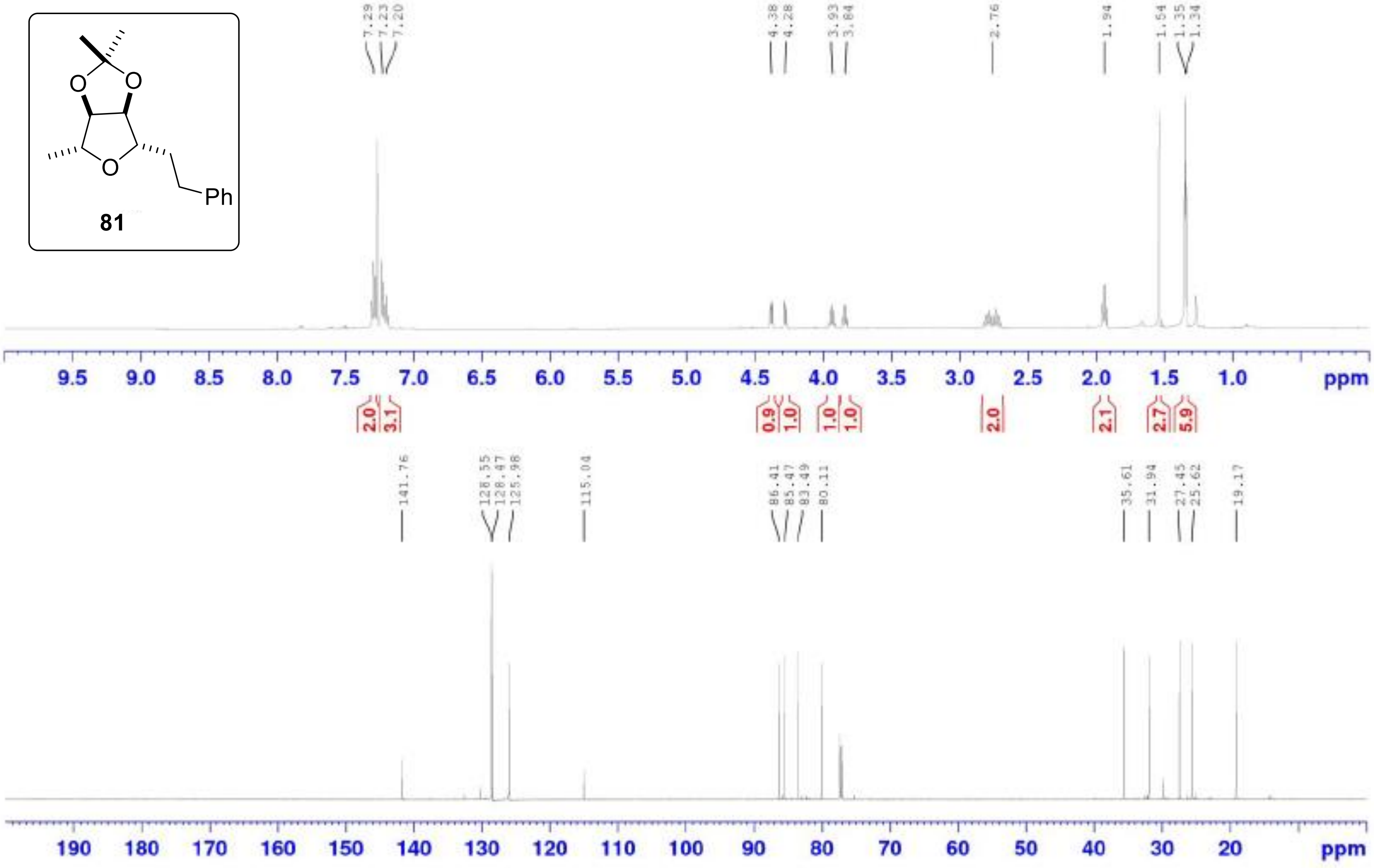


**Supplementary Figure 67**. NMR spectra of **81**.

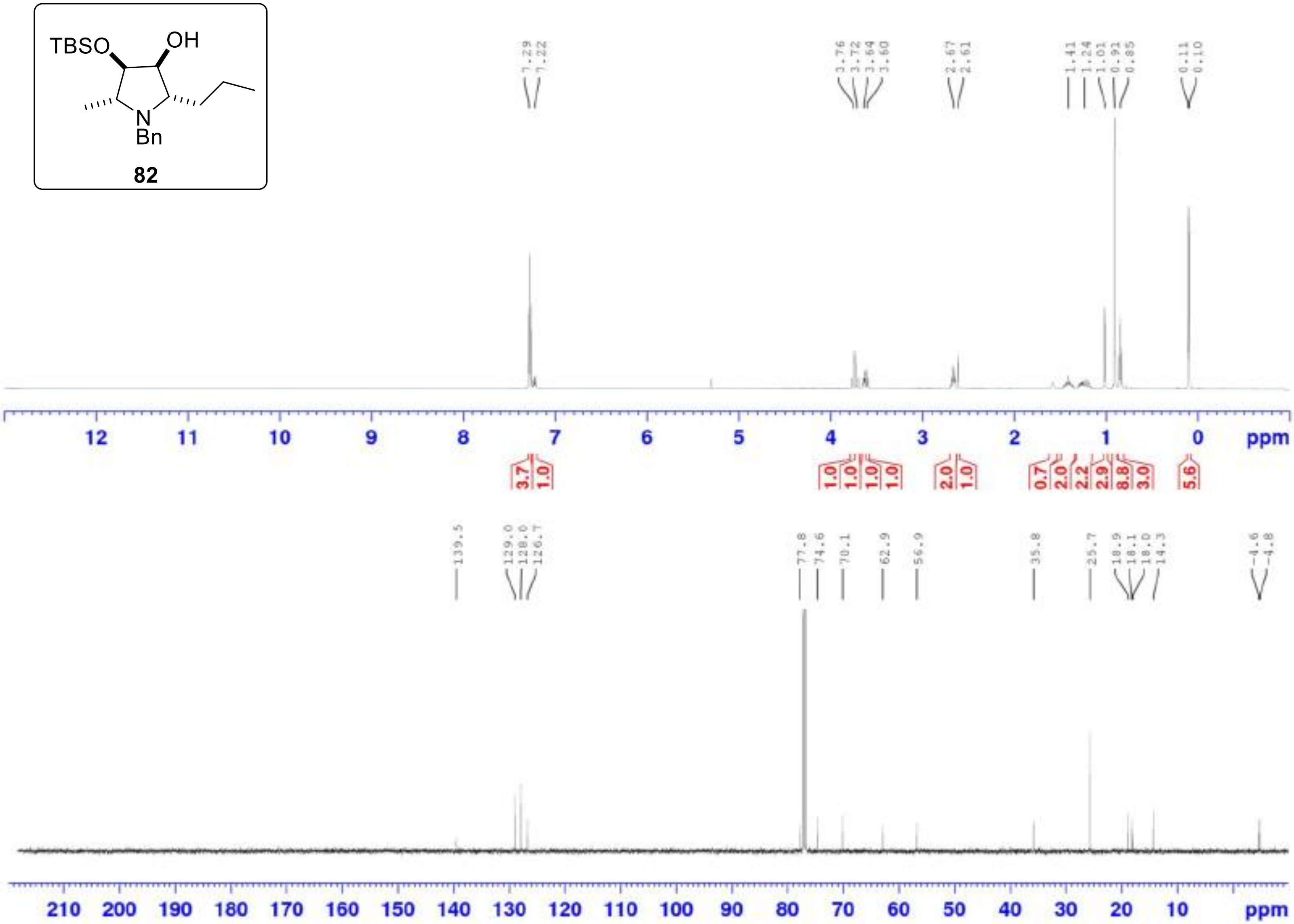

**Supplementary Figure 68**. NMR spectra of **82**.

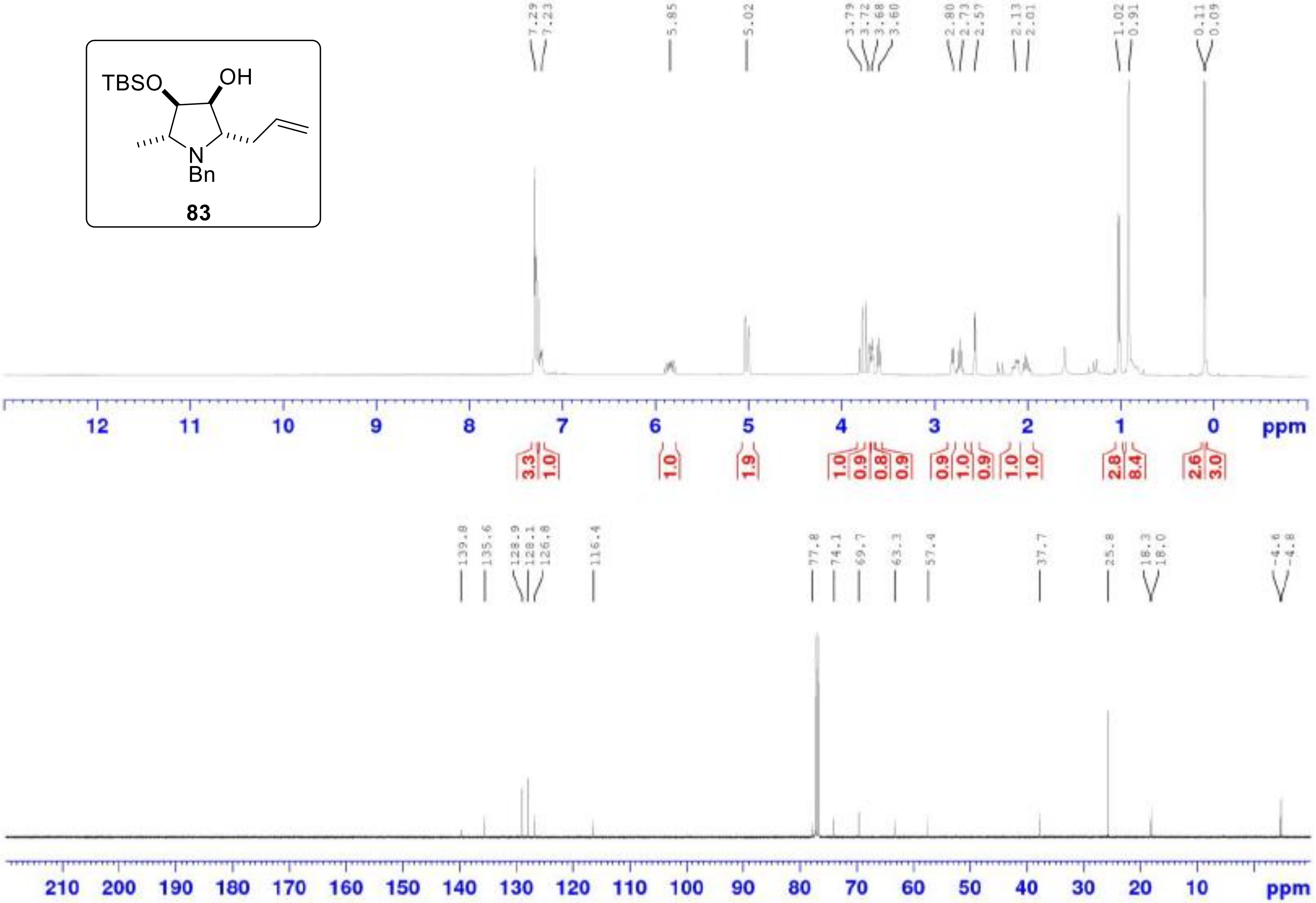


**Supplementary Figure 69**. NMR spectra of **83**.

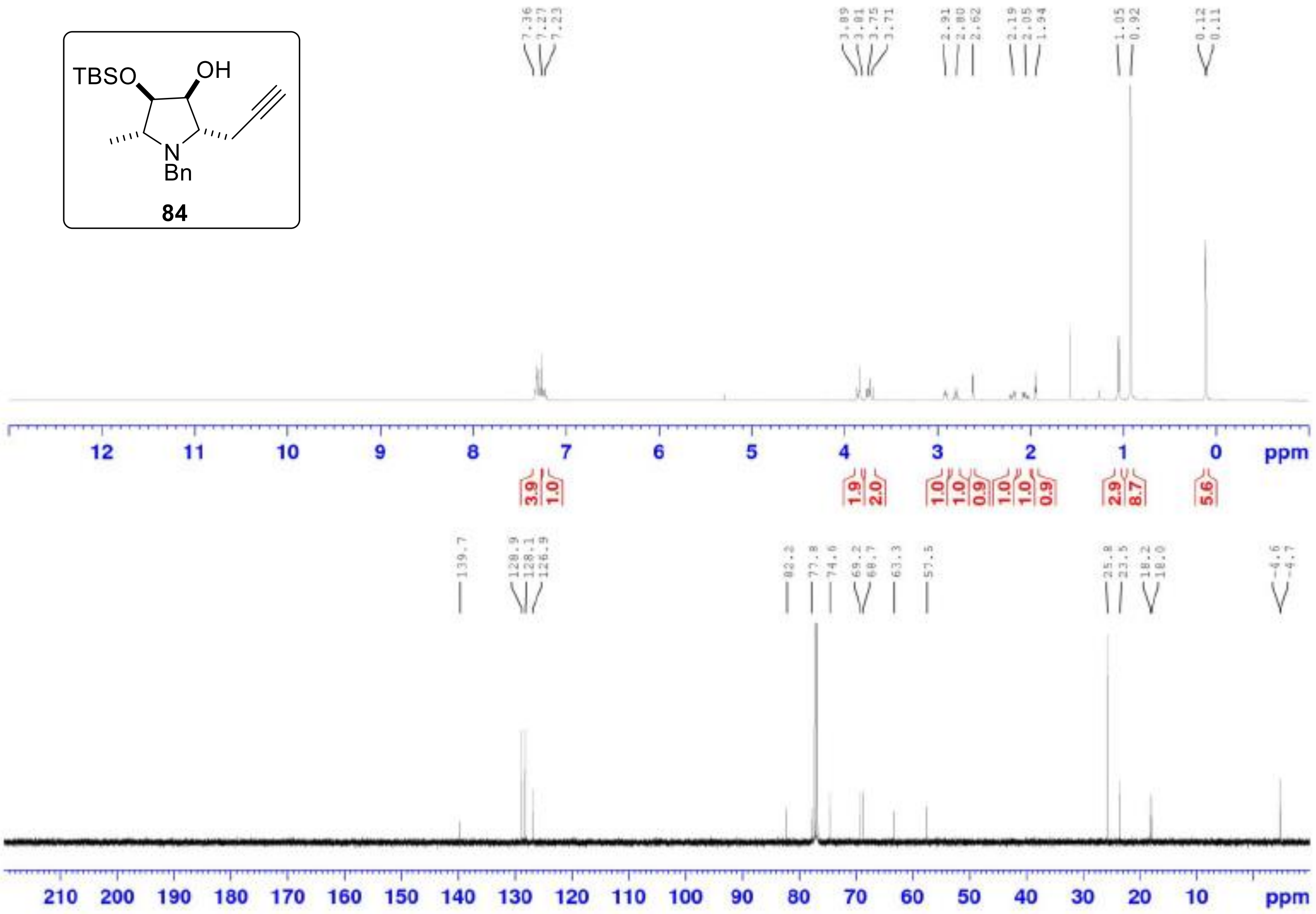


**84**

**Supplementary Figure 70**. NMR spectra of **84**.

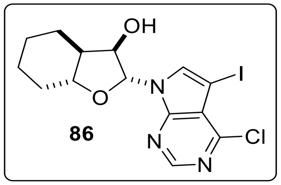


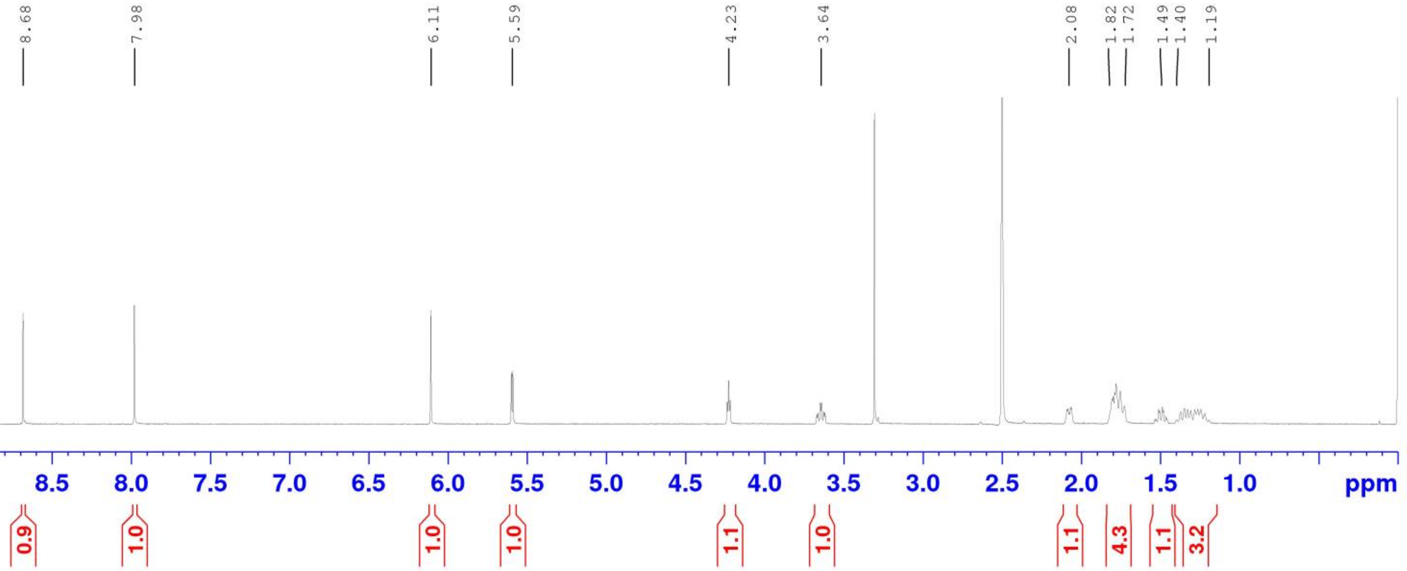

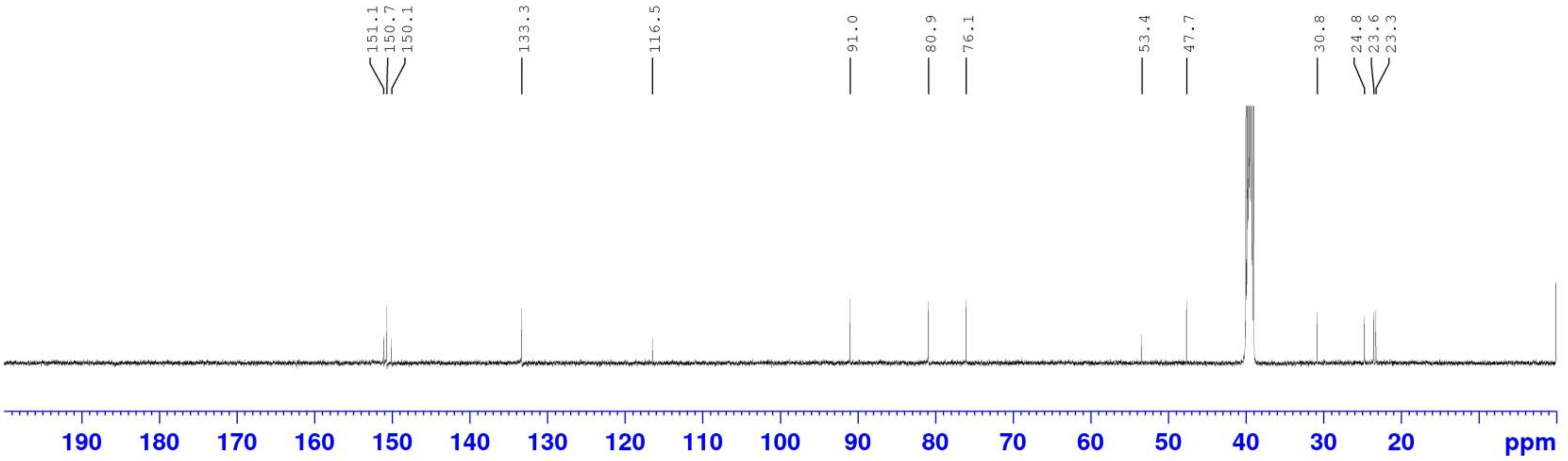

**Supplementary Figure 71**. NMR spectra of **86**.

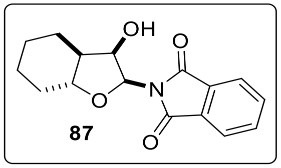


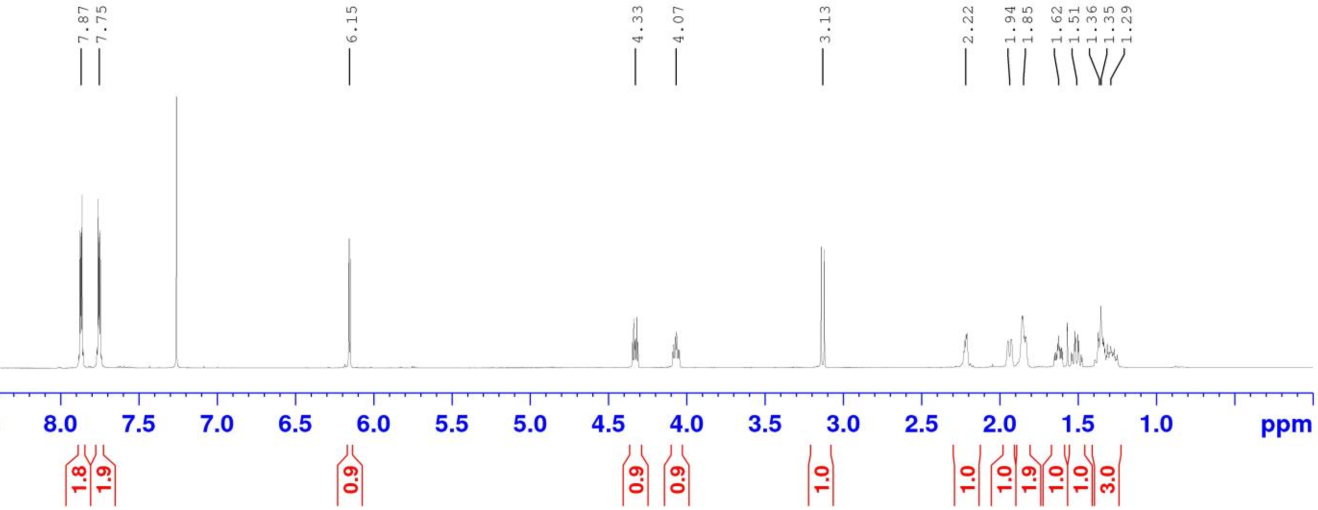


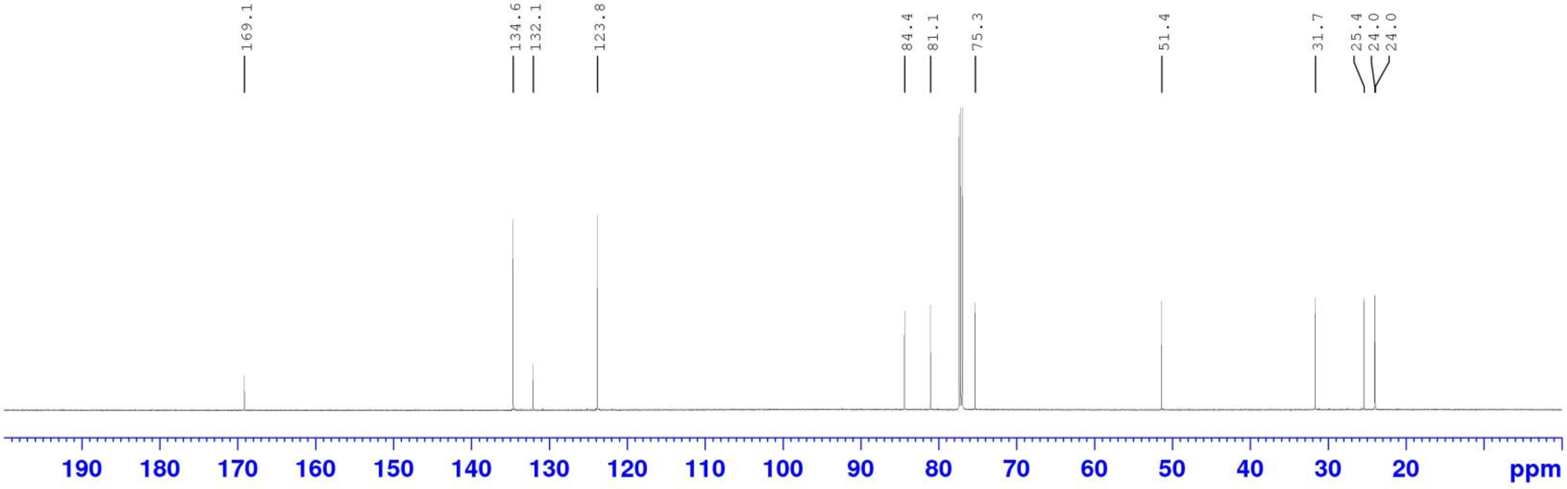


**Supplementary Figure 72**. NMR spectra of **87**.

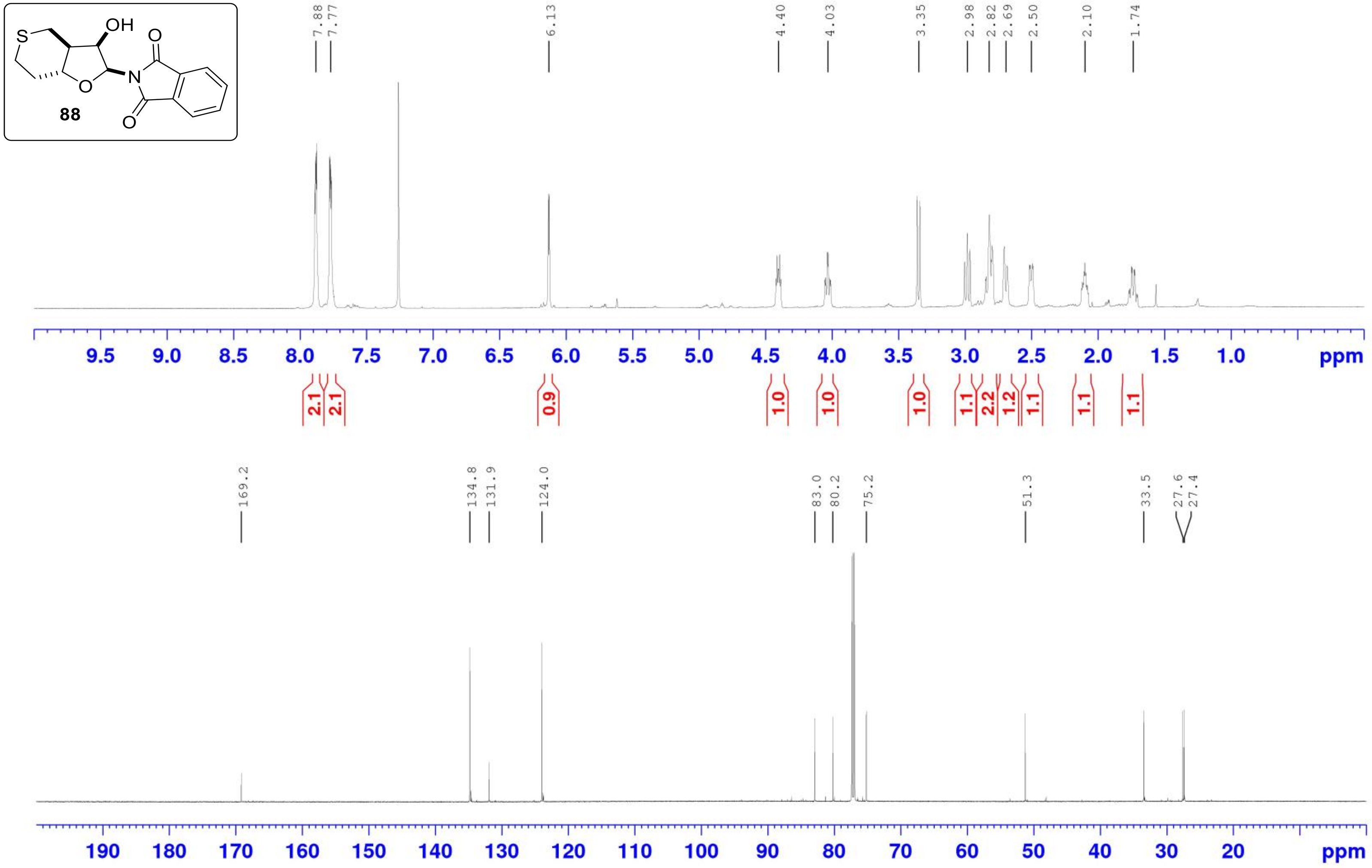


**Supplementary Figure 73**. NMR spectra of **88**.

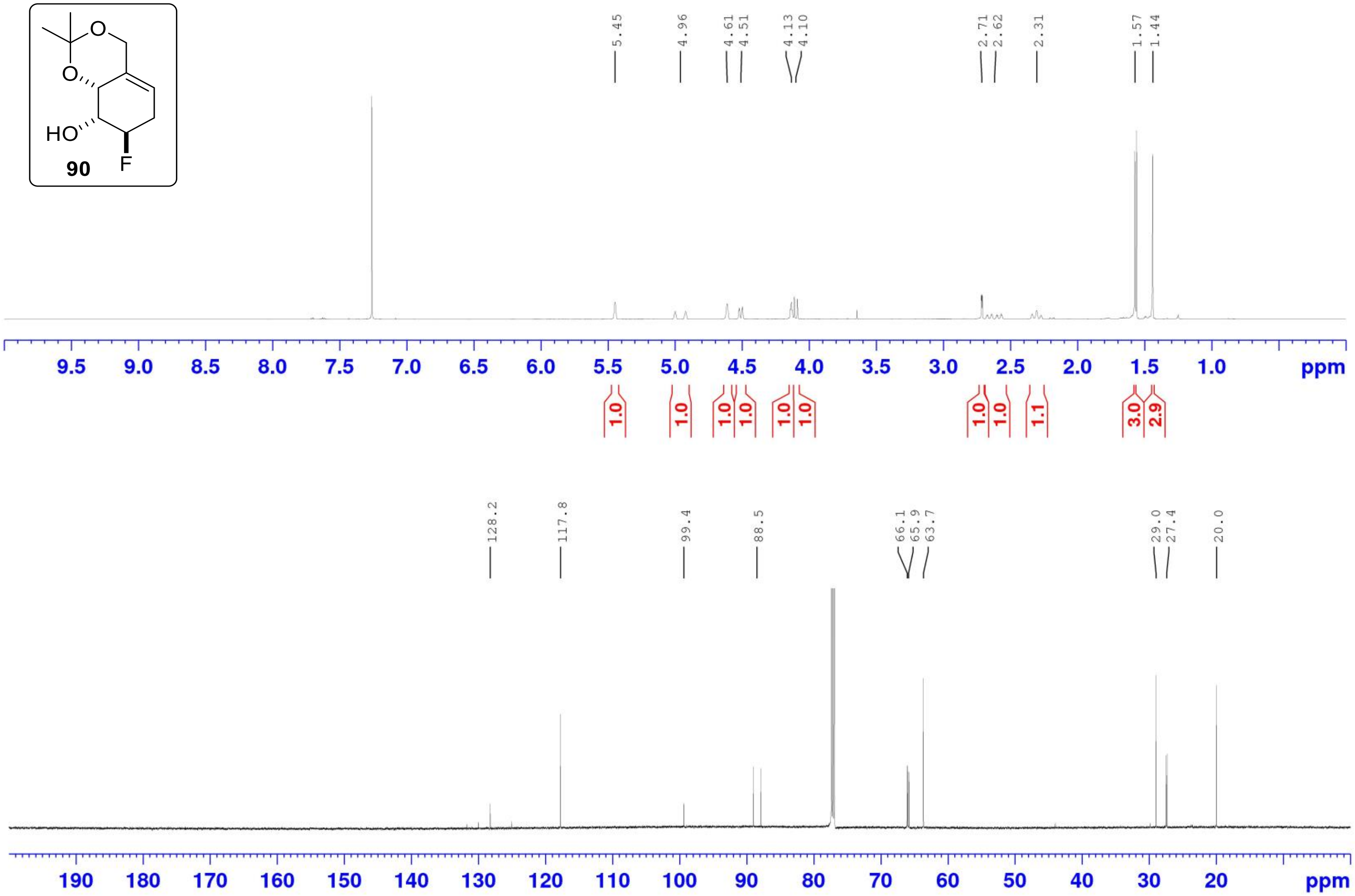


**Supplementary Figure 74**. NMR spectra of **90**.

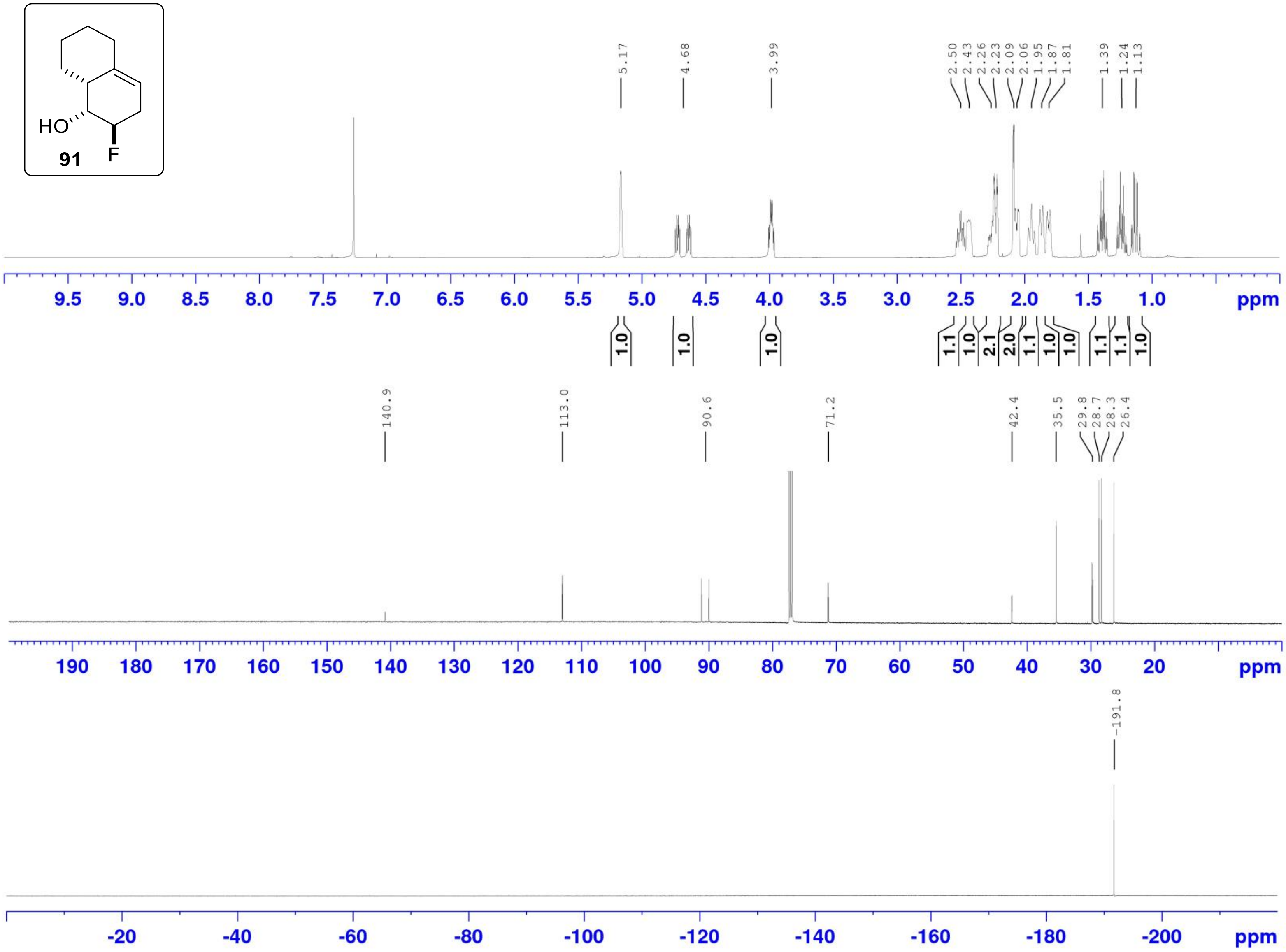


**Supplementary Figure 75**. NMR spectra of **91**.

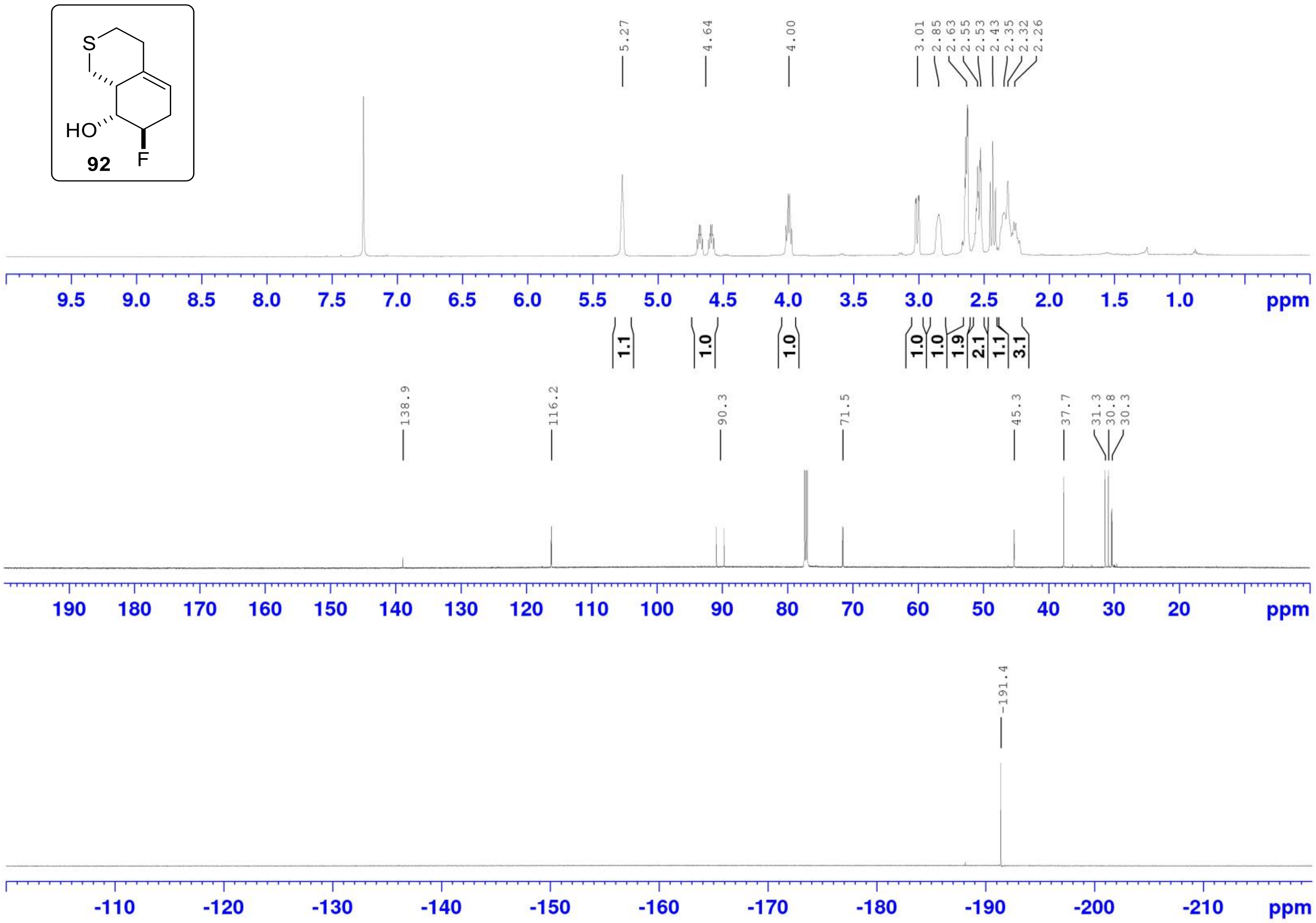


**Supplementary Figure 76**. NMR spectra of **92**.

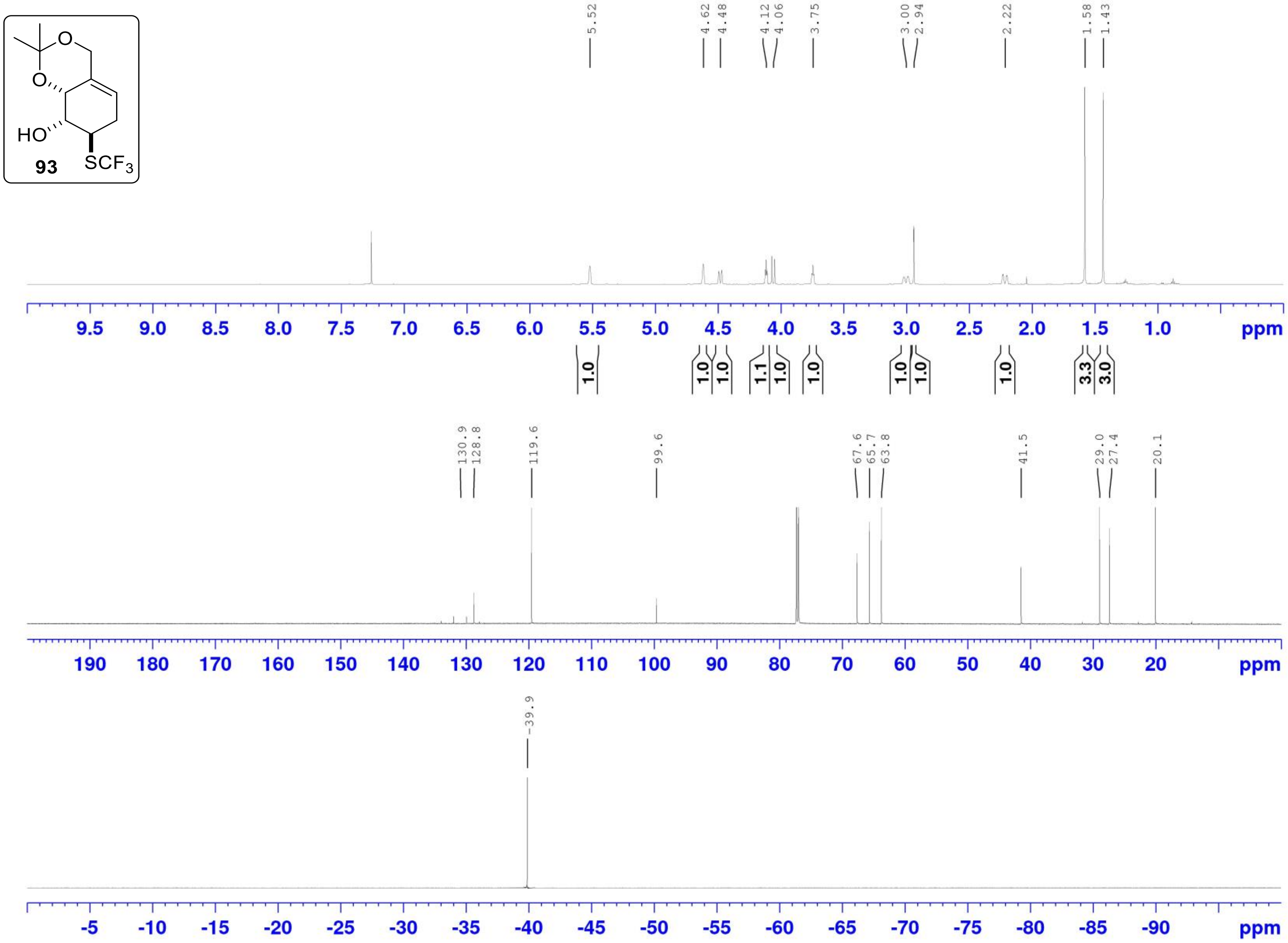


**Supplementary Figure 77**. NMR spectra of **93**.

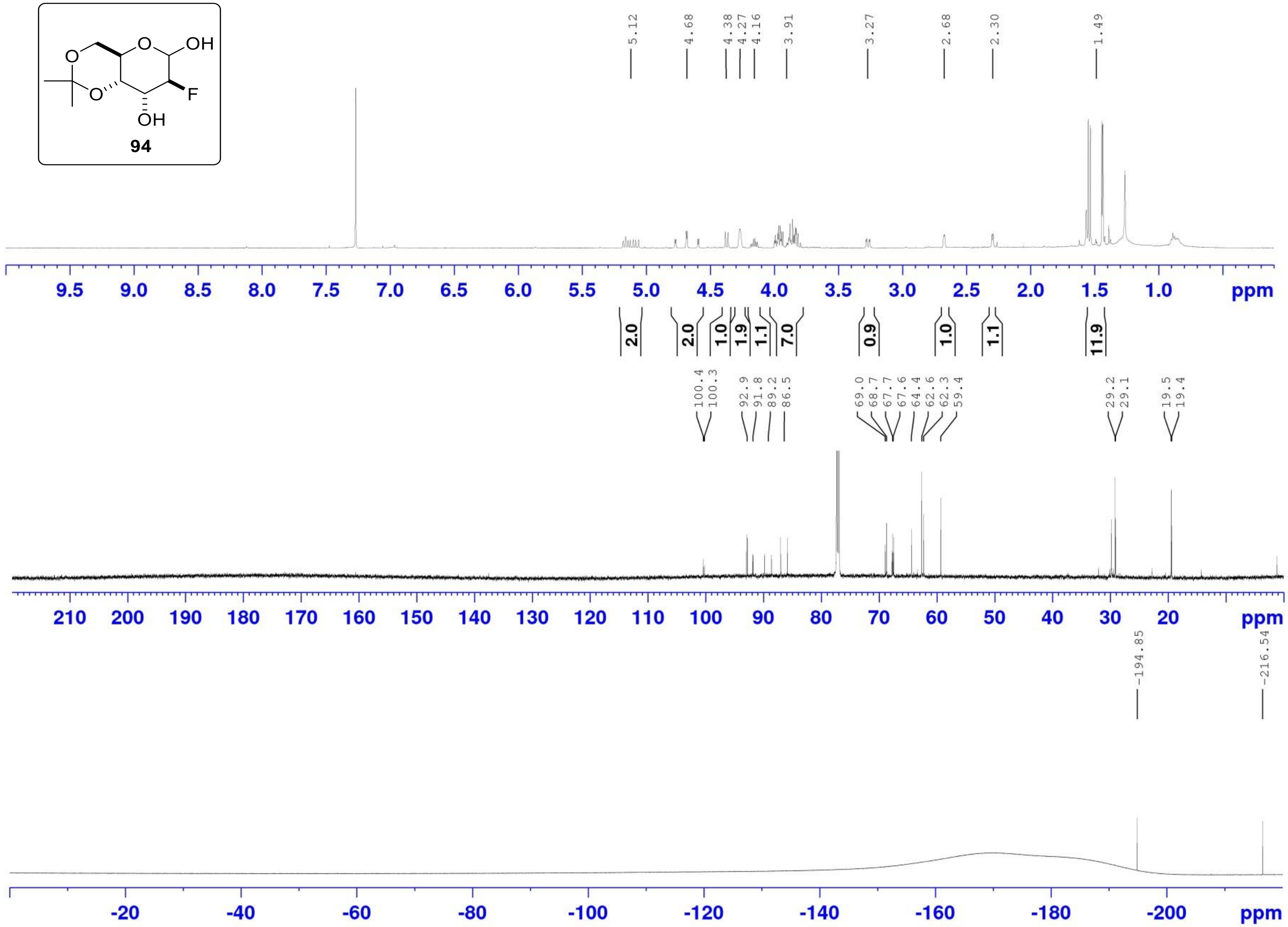


**Supplementary Figure 78**. NMR spectra of **94**.

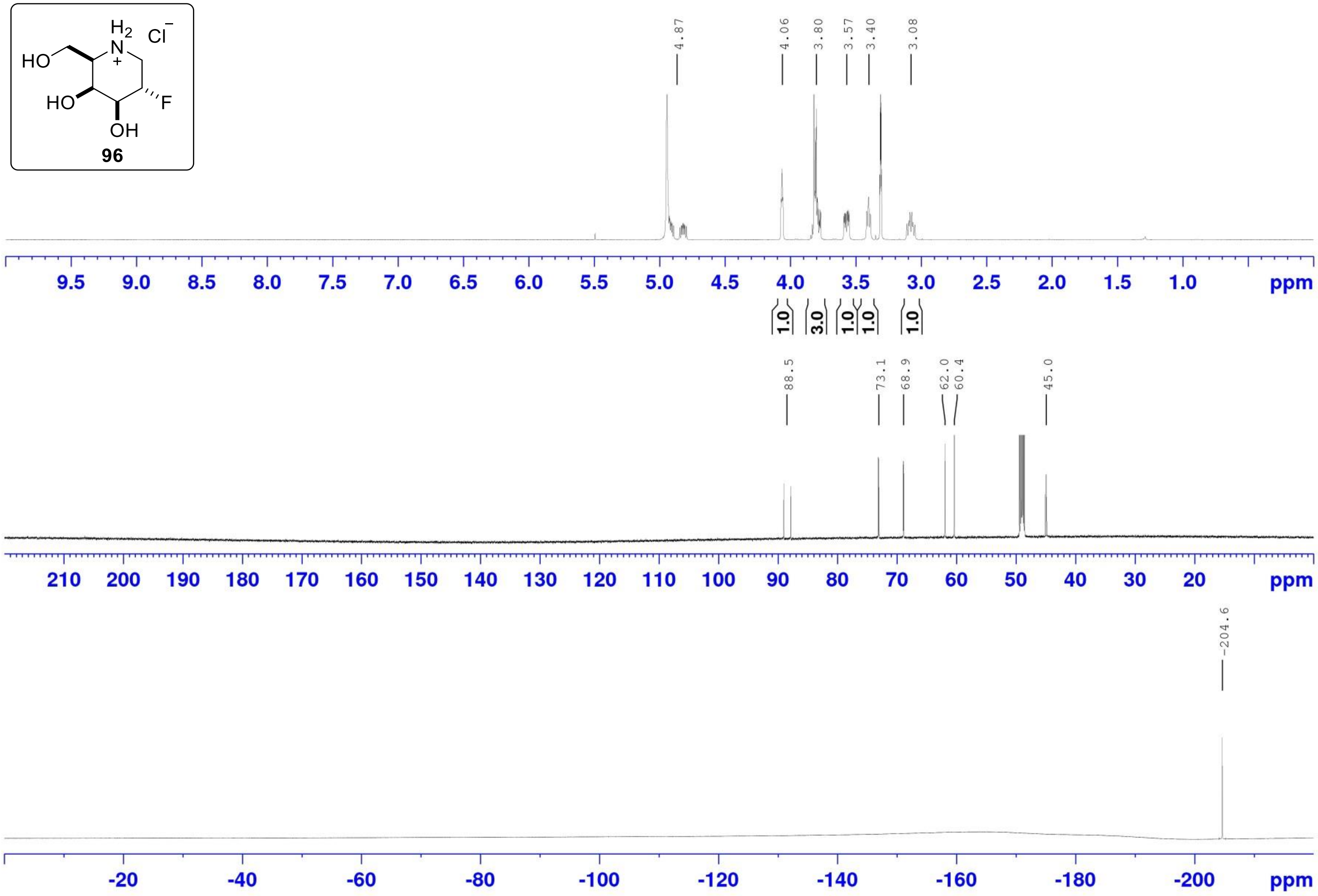


**Supplementary Figure 79**. NMR spectra of **95**.

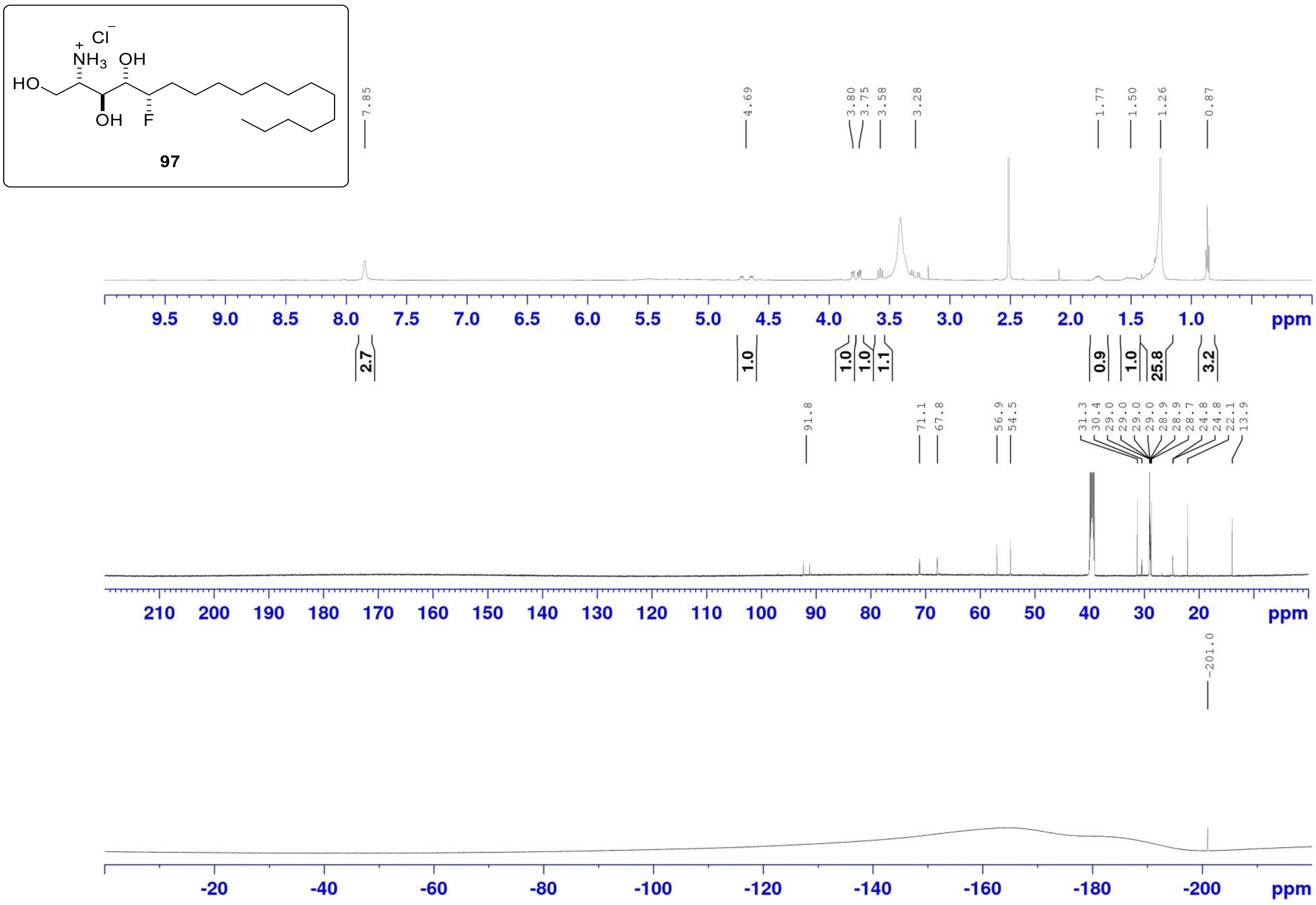


**Supplementary Figure 80**. NMR spectra of **97**.

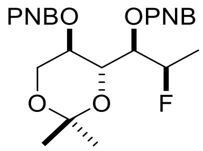


**47-XRD**

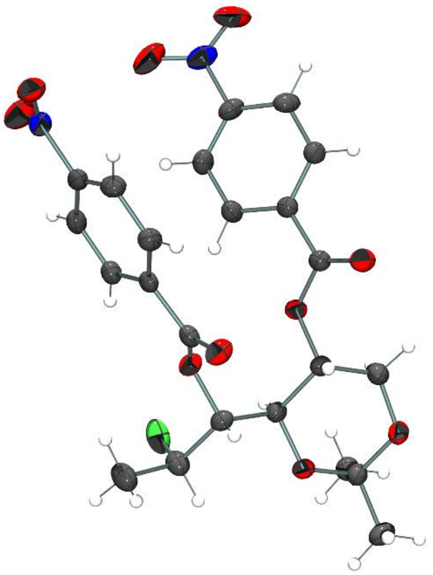

**Supplementary Figure 81**. XRD structure of compound **47-XRD (CCDC number: 1556393; CIF label: 6-MWM-010)**

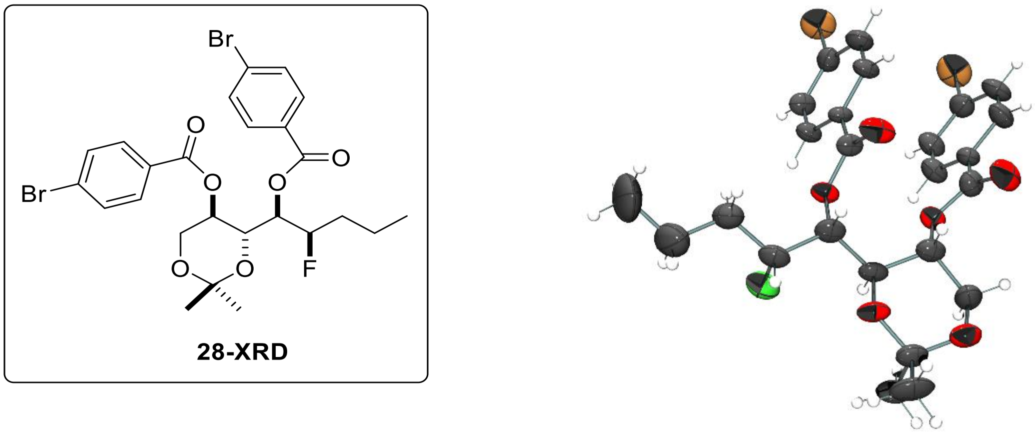


**Supplementary Figure 82**. XRD structure of compound **28-XRD (CCDC number: 1556394; CIF label: 6-MWM-110)**

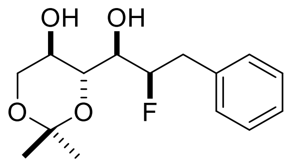


**56-XRD**

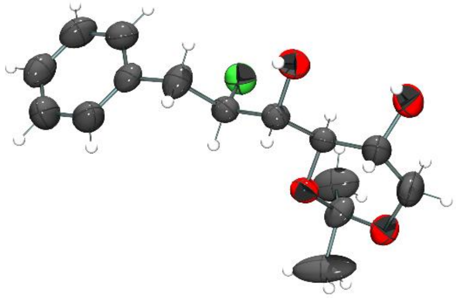

**Supplementary Figure 83**. XRD structure of compound **56-XRD (CCDC number: 1556395; CIF label: 5-MWM-110)**

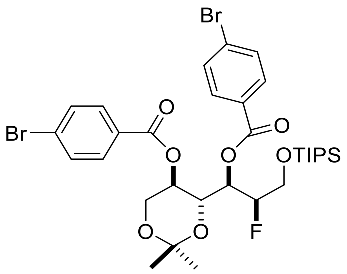


**58-XRD**

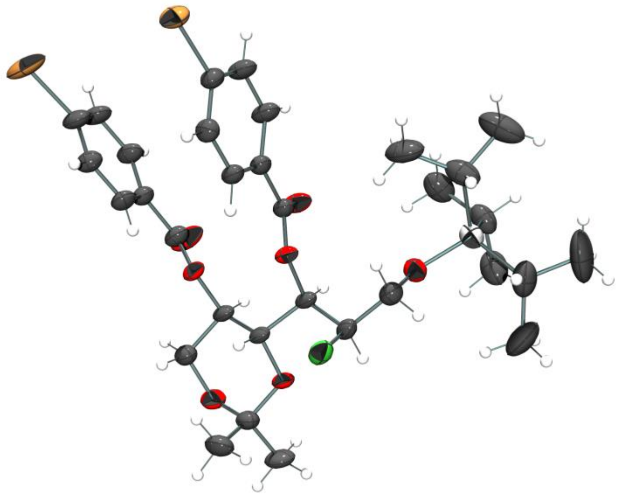

**Supplementary Figure 84**. XRD structure of compound **58-XRD (CCDC number: 1556396; CIF label: 5-MWM-135)**

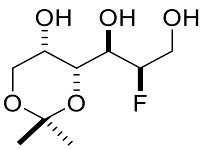


**Pre95-XRD**

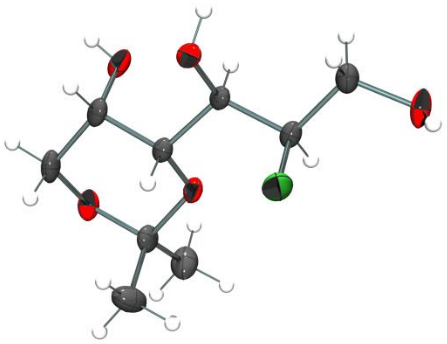

**Supplementary Figure 85**. XRD structure of compound **Pre95-XRD (CCDC number: 1556397; CIF label: MWM-565)**

| Compound Reference | **47-XRD** | **28-XRD** | **56-XRD** | **58-XRD** | **Pre95-XRD** |
|---|---|---|---|---|---|
| Chemical Formula | $C_{23}H_{23}N_2O_{10}F$ | $C_{25}H_{27}O_6Br_2F$ | $C_{15}H_{21}O_4F$ | $C_{32}H_{43}Br_2FO_7Si$ | $C_9H_{17}FO_5$ |
| FW | 506.43 | 602.28 | 284.32 | 746.57 | 224.22 |
| Crystal System | Orthorhombic | Orthorhombic | Orthorhombic | Monoclinic | Triclinic |
| Space group | $P2_12_12_1$ | $P2_12_12_1$ | $P2_12_12_1$ | $P2_1$ | P1 |
| a/Å | 7.8910(2) | 5.5445(4) | 5.60440(10) | 12.6483(4) | 9.3287(6) |
| b/Å | 11.8417(3) | 18.6627(13) | 13.0932(3) | 11.3201(4) | 9.3568(5) |
| c/Å | 24.9605(6) | 24.3453(19) | 20.2022(4) | 13.4868(4) | 25.3029(15) |
| α/° | 90 | 90 | 90 | 90 | 91.911(2) |
| β/° | 90 | 90 | 90 | 115.6810(10) | 97.536(2) |
| γ/° | 90 | 90 | 90 | 90 | 90.195(3) |
| Unit cell volume/$Å^3$ | 2332.38(10) | 2519.1(3) | 1482.43(5) | 1740.29(10) | 2188.2(2) |
| Z | 4 | 4 | 4 | 2 | 8 |
| Temperature/K | 150(2) | 150(2) | 296(2) | 150(2) | 150(2) |
| Radiation type | Cu Kα | Cu Kα | Cu Kα | Cu Kα | Cu Kα |
| Absorption coefficient, μ/$mm^{-1}$ | 1.023 | 4.476 | 0.83 | 3.689 | 1.038 |
| All Reflections | 16537 | 16215 | 9652 | 25308 | 60788 |
| Unique Reflections | 4273 | 4631 | 2610 | 6336 | 14635 |
| Flack parameter | 0.03(4) | 0.002(8) | 0.07(5) | 0.001(5) | 0.04(3) |
| $R_{int}$ | 0.0337 | 0.0389 | 0.0205 | 0.0305 | 0.0532 |

| | | | | | |
|---|---|---|---|---|---|
| Final $R_1$ values ($I>2\sigma(I)$) | 0.0281 | 0.0251 | 0.0368 | 0.0351 | 0.0436 |
| Final $wR(F^2)$ values ($I>2\sigma(I)$) | 0.0735 | 0.0642 | 0.1023 | 0.0916 | 0.1146 |
| Final $R_1$ values (all data) | 0.0286 | 0.0258 | 0.0388 | 0.0361 | 0.0437 |
| Final $wR(F^2)$ (all data) | 0.074 | 0.0648 | 0.1048 | 0.0927 | 0.1147 |
| Goodness of fit | 1.049 | 1.034 | 1.085 | 1.031 | 1.044 |

**Supplementary Table 3.** Summary of parameters from XRD analysis

(*R*)-**TS₁**-X

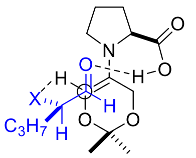

| X | X--H (Å) | H--H (Å) |
|---|---|---|
| Cl | 2.68 | - |
| F | 2.37 | - |

(*R*)-**TS₁**-X

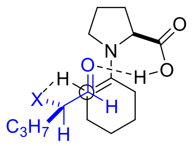

| X | X--H (Å) | H--H (Å) |
|---|---|---|
| Cl | 2.69 | 2.14 |
| F | 2.36 | 2.18 |

(*R*)-**TS₁**-X

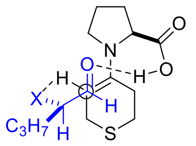

| X | X--H (Å) | H--H (Å) |
|---|---|---|
| Cl | 2.73 | 2.22 |
| F | 2.34 | 2.24 |

(*R*)-**TS₁**-X

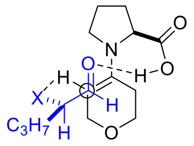

| X | X--H (Å) | H--H (Å) |
|---|---|---|
| Cl | 2.68 | 2.19 |
| F | 2.33 | 2.24 |

(*S*)-**TS₁**-X

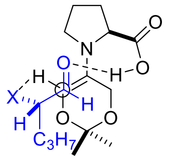

| X | X--H (Å) | H--H (Å) | ΔΔG ‡ kcal/mol |
|---|---|---|---|
| Cl | 2.72 | - | 2.7 |
| F | 2.37 | - | 2.9 |

(*S*)-**TS₁**-X

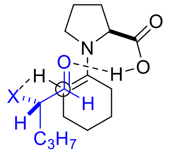

| X | X--H (Å) | H--H (Å) | ΔΔG ‡ kcal/mol |
|---|---|---|---|
| Cl | 2.74 | 2.22, 2.36 2.39 | 3.3 |
| F | 2.40 | 2.35, 2.36 | 3.5 |

(*S*)-**TS₁**-X

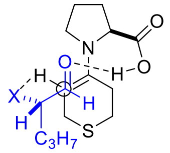

| X | X--H (Å) | H--H (Å) | ΔΔG ‡ kcal/mol |
|---|---|---|---|
| Cl | 2.73 | 2.22, 2.29 | 3.1 |
| F | 2.40 | 2.25 | 3.5 |

(*S*)-**TS₁**-X

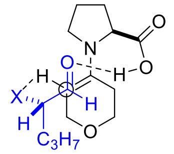

| X | X--H (Å) | H--H (Å) | ΔΔG ‡ kcal/mol |
|---|---|---|---|
| Cl | 2.73 | 2.22, 2.38 | 2.7 |
| F | 2.38 | 2.36, 2.37 2.39 | 2.9 |

**Supplementary Figure 86.** Intermolecular interactions of (*R*) or (*S*)-2-fluoro- and chloropentanal with **13**, cyclohexanone, **35**, and tetrahydropyranone observed in (*R*)- and (*S*)-$TS_1$ transition state structures. DFT calculations were performed using $IEFPCM_{(DCM)}$M06-2X/6-311++G(2d,2p)//$IEFPCM_{(DCM)}$M06-2X/6-31+G(d,p) level of theory

## Supplementary References


1. Bergeron-Brlek, M.; Teoh, T.; Britton, R. *Org. Lett.* **2013**, *15* (14), 3554

2. J.N. Barlow, J.S.Blanchard, *Carbohydrate Research*, **2000**, *328*, 473
