# Supplementary material for: Diversity-oriented synthesis of glycomimetics
Source: Commun Chem. 2021 Jun 24;4:96. doi: 10.1038/s42004-021-00520-3 (PMC9814271; doi:10.1038/s42004-021-00520-3)
Supplement: Supplementary file 3 — Supplementary Data 1 [file 42004_2021_520_MOESM3_ESM.pdf]

Supplementary Data 1

Density functional theory (DFT) computations were performed using the software package Gaussian 16, Revision C. 01.[1] All DFT optimizations were performed using the M06-2X functional[2] with a 6-31+G(d,p) basis set with solvent dichloromethane (DCM) accounted for using the integral equation formalism polarizable continuum model (IEFPCM) and the default parameters of DCM (ε = 8.93).[3] The use of the hybrid meta-GGA M06-2X functional of Truhlar was selected based on its proven robustness. This functional accounts for nonlocal effects of electronic dispersion and has been found to give good estimates for reaction enthalpies in bond-forming reactions. The optimized geometries were verified as transition state structures (one imaginary frequency) or minima (zero imaginary frequencies) by frequency calculations. Intrinsic reaction coordinate (IRC) calculations were performed to confirm that all transition state structures were linked to relevant minima.[4,5] The energies of the M06-2X/6-31+G(d,p) optimized structures were further refined by single point calculations performed at the M06-2X/6-311++G(2d,2p) level with solvent DCM accounted for using the IEFPCM solvation model. The thermal corrections to the Gibbs free energies (temperature = 273.15 K) computed at the lower level of theory (M06-2X/6-31+G(d,p)) were added to the electronic energies obtained from the single point calculations to provide the final reported Gibbs free energies. The keyword (integral=grid=ultrafine) was used for all calculations. The reported activation barriers were computed with respect to the separate reagents and associated transition state. The 3D images of all optimized geometries were generated with CYLview[6] and GaussView 6[7] was used to construct all structures prior to optimization and to visualize the output from the Gaussian 16 calculations. The program NBO 6 was used for the reported second-order perturbation theory NBO analyses performed at the M06-2X/6-31+G(d,p) level of theory. The reported Noncovalent Interaction (NCI) surfaces (isovalue = 0.3 min = -0.05 and max = 0.05) were computed with the M06-2X-D3 functional and a LACV3P++** basis set using the program Jaguar of the Schrödinger software package.[8]

Protocol for Conformational Searches Providing Structures for DFT Optimizations: Using the MacroModel program of the Schrödinger software package[9] models were constructed for *re*-stereofacial enamine addition to (*R*)- or (*S*)-2-fluoro-, 2-chloro-, 2-bromopentanal located in a cis-orientation to the L-proline carboxylic acid. The enamine component of these models was derived from the respective ketones, i.e., dioxanone, cyclohexanone, tetrahydropyranone and thiopyranone. The carbon-carbon bond forming distances of these enamine addition models was set to 2.1 Å and the dihedral angle between the aldehyde carbonyl and carbon-halogen bond locked into three different geometries, corresponding to 90°, 180°, and 310° degrees. This resulted in three unique structures for each aldehyde and enamine combination, respectively, resembling (1) Evans-Cornforth (i.e., anti-parallel alignment of carbonyl and α-halogen substituent) model, (2) Felkin-Anh (i.e., perpendicular orientation of carbonyl and α-halogen substituent) and (3) a unique model (i.e., gauche orientation of the carbonyl and α-halogen substituent) termed the Dudding-Britton model. The internal coordinates of the carbons of the enamine alkene and aldehyde carbonyl, as well as, the carbon and halogen atoms of each these transition state models were then frozen, i.e., fixed (see Figures S13 – S18 of Supporting Information for representative images with the noted frozen (fixed) atoms highlighted). Each of these transition state models was next subjected to Monte Carlo conformational searches (MCCS) using the OPLS3 force field. From

these conformational searches, an array of conformers for enamine aldol addition were generated. These conformers were then exported to the program Gaussian 16 and optimized at the $IEFPCM_{(DCM)}$ M06-2X/6-31+G(d,p) level of theory as outlined in the above computational methods section. From these optimizations, transition states for enamine addition were located, e.g., labeled as (*R*)-**TS1**$_{O}$-F, (*S*)-**TS2**$_{O}$-F and (*S*)-**TS3**$_{O}$-F. As noted in the above computational methods the energies of these M06-2X/6-31+G(d,p) optimized structures were further refined by single point calculations performed at the M06-2X/6-311++G(2d,2p) level with solvent DCM accounted for using the IEFPCM solvation model. The thermal corrections to the Gibbs free energies (temperature = 273.15 K) computed at the lower level of theory (M06-2X/6-31+G(d,p)) were added to the electronic energies obtained from the single point calculations to provide the final reported Gibbs free energies. The keyword (integral=grid=ultrafine) was used for all calculations. The reported activation barriers were computed with respect to the separate reagents and associated transition state.

---

[1] Gaussian 16, Revision C.01, Frisch, M. J.; Trucks, G. W.; Schlegel, H. B.; Scuseria, G. E.; Robb, M. A.; Cheeseman, J. R.; Scalmani, G.; Barone, V.; Petersson, G. A.; Nakatsuji, H.; Li, X.; Caricato, M.; Marenich, A. V.; Bloino, J.; Janesko, B. G.; Gomperts, R.; Mennucci, B.; Hratchian, H. P.; Ortiz, J. V.; Izmaylov, A. F.; Sonnenberg, J. L.; Williams-Young, D.; Ding, F.; Lipparini, F.; Egidi, F.; Goings, J.; Peng, B.; Petrone, A.; Henderson, T.; Ranasinghe, D.; Zakrzewski, V. G.; Gao, J.; Rega, N.; Zheng, G.; Liang, W.; Hada, M.; Ehara, M.; Toyota, K.; Fukuda, R.; Hasegawa, J.; Ishida, M.; Nakajima, T.; Honda, Y.; Kitao, O.; Nakai, H.; Vreven, T.; Throssell, K.; Montgomery, J. A., Jr.; Peralta, J. E.; Ogliaro, F.; Bearpark, M. J.; Heyd, J. J.; Brothers, E. N.; Kudin, K. N.; Staroverov, V. N.; Keith, T. A.; Kobayashi, R.; Normand, J.; Raghavachari, K.; Rendell, A. P.; Burant, J. C.; Iyengar, S. S.; Tomasi, J.; Cossi, M.; Millam, J. M.; Klene, M.; Adamo, C.; Cammi, R.; Ochterski, J. W.; Martin, R. L.; Morokuma, K.; Farkas, O.; Foresman, J. B.; Fox, D. J. Gaussian, Inc., Wallingford CT, 2016.

[2] Zhao, Y.; Truhlar, D. G. The M06 Suite of Density Functionals for Main Group Thermochemistry, Thermochemical Kinetics, Noncovalent Interactions, Excited States, and Transition Elements: Two New Functionals and Systematic Testing of Four M06-class Functionals and 12 Other Functionals. *Theor. Chem. Acc*. **2008**, *120*, 215-241.

[3] Cancès, E.; Mennucci, B.; Tomasi, J. A New Integral Equation Formalism for the Polarizable Continuum Model: Theoretical Background and Applications to Isotropic and Anistropic Dielectrics. *J. Chem. Phys.* **1997**, *107*, 3032–3041.

[4] C. González and H. B. Schlegel, Reaction path following in mass-weighted internal coordinates, *J. Phys. Chem.* **1990**, *94*, 5523–5527.

[5] K. Fukui, The Path of Chemical Reactions – The IRC Approach, *Acc. Chem. Res.* **1981**, *14*, 363–368.

[6] Legault, C. Y. CYLview, version 1.0b; Universitéde Sherbrooke: Quebec, Canada, **2009**; http://www.cylview.org.

[7] GaussView, Version 6, Dennington, Roy; Keith, Todd A.; Millam, John M. Semichem Inc., Shawnee Mission, KS, 2016.

[8] Schrödinger Release 2019–2: Jaguar; Schrödinger, LLC: New York, 2019.

[9] Schrödinger Release 2019–2: MacroModel; Schrödinger, LLC: New York, 2019.

Energies of Calculated Structures and Cartesian Coordinates

Supplementary Table 4. Energies for enamine addition to 2-chloropentanal. Reported relative Gibbs free energy energies for structures optimized at the IEFPCM$_{(DCM)}$M06-2X/6-311++G(2d,2p)//IEFPCM$_{(DCM)}$M06-2X/6-31+G(d,p) level of theory represent the sum of the thermal correction to Gibbs free energy computed at the IEFPCM$_{(DCM)}$M06-2X/6-31+G(d,p) level of theory and single point energies computed at the IEFPCM$_{(DCM)}$M06-2X/6-311++G(2d,2p). All energies are reported in Hartrees.

**Structure**	**Single Point Energies, E IEFPCM$_{(DCM)}$M06-2X/6-311++G(2d,2p)**	**Thermal Corrections to Gibbs Free Energies, IEFPCM$_{(DCM)}$M06-2X/6-31+G(d,p)**	**Gibbs Free Energies (G), IEFPCM$_{(DCM)}$M06-2X/6-31+G(d,p)**	**Gibbs Free Energies (G), IEFPCM$_{(DCM)}$M06-2X/6-311++G(2d,2p)// IEFPCM$_{(DCM)}$M06-2X/6-31+G(d,p)**
2-Chloropentanal	-731.33393130	0.102879	-731.121809	-731.2310523
Enamine of Cyclohexanone (G)	-634.55147535	0.238392	-634.142903	-634.3130834
Enamine of Dioxane (O)	-784.98483791	0.244077	-784.522619	-784.7407609
Enamine of Tetrahydro-4H-thiopyranone (T)	-993.43210840	0.210205	-993.028735	-993.2219034
Enamine ofTetrahydro-4H-pyranone (P)	-670.45469021	0.214793	-670.055312	-670.2398972
(*R*)-**TS1**$_P$-Cl-Pre	-1401.806718	0.33727	-1401.176087	-1401.469448
(*R*)-**TS1**$_P$-Cl	-1401.797805	0.340985	-1401.164861	-1401.45682
(*R*)-**TS1**$_P$-Cl-P	-1401.829317	0.344987	-1401.192882	-1401.48433
(*S*)-**TS1**$_P$-Cl-Pre	-1401.805905	0.337081	-1401.175625	-1401.468824
(*S*)-**TS1**$_P$-Cl	-1401.791734	0.341063	-1401.159001	-1401.450671
(*S*)-**TS1**$_P$-Cl-P	-1401.825061	0.345083	-1401.188431	-1401.479978
(*R*)-**TS1**$_G$-Cl-Pre	-1365.903561	0.361196	-1365.263491	-1365.542365
(*R*)-**TS1**$_G$-Cl	-1365.894619	0.36506	-1365.252009	-1365.529559
(*R*)-**TS1**$_G$-Cl-P	-1365.927951	0.368557	-1365.282361	-1365.559394
(*S*)-**TS1**$_G$-Cl-Pre	-1365.902931	0.361683	-1365.262572	-1365.541248
(*S*)-**TS1**$_G$-Cl	-1365.888191	0.364994	-1365.245974	-1365.523197
(*S*)-**TS1**$_G$-Cl-P	-1365.923664	0.369188	-1365.277488	-1365.554476
(*R*)-**TS1**$_O$-Cl-Pre	-1516.33886	0.367316	-1515.644924	-1515.971544
(*R*)-**TS1**$_O$-Cl	-1516.327244	0.369496	-1515.632299	-1515.957748
(*R*)-**TS1**$_O$-Cl-P	-1516.354	0.372133	-1515.656854	-1515.981867
(*S*)-**TS1**$_O$-Cl-Pre	-1516.336956	0.366549	-1515.644002	-1515.970407
(*S*)-**TS1**$_O$-Cl	-1516.322975	0.369358	-1515.628417	-1515.953617
(*S*)-**TS1**$_O$-Cl-P	-1516.35131	0.372586	-1515.653803	-1515.978724
(*R*)-**TS1**$_T$-Cl-Pre	-1724.786027	0.333886	-1724.150088	-1724.452141
(*R*)-**TS1**$_T$-Cl	-1724.774436	0.336803	-1724.137252	-1724.437633
(*R*)-**TS1**$_T$-Cl-P	-1724.806482	0.341091	-1724.165365	-1724.465391
(*S*)-**TS1**$_T$-Cl-Pre	-1724.785259	0.333383	-1724.150079	-1724.451876
(*S*)-**TS1**$_T$-Cl	-1724.768234	0.337056	-1724.131053	-1724.431178
(*S*)-**TS1**$_T$-Cl-P	-1724.802266	0.341112	-1724.161176	-1724.461154

Pre – Precomplex

P – Product

2-Chloropentanal

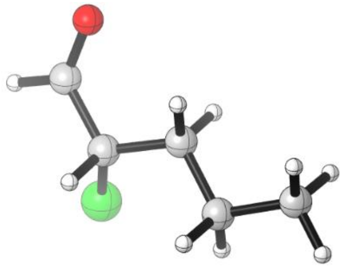

- Thermochemistry -

Zero-point correction=	0.133590 (Hartree/Particle)
Thermal correction to Energy=	0.141018
Thermal correction to Enthalpy=	0.141883
Thermal correction to Gibbs Free Energy=	0.102879
Sum of electronic and zero-point Energies=	-731.091098
Sum of electronic and thermal Energies=	-731.083671
Sum of electronic and thermal Enthalpies=	-731.082806
Sum of electronic and thermal Free Energies=	-731.121809

Number of Imaginary Frequencies = 0

E (Single Point Energy) [IEFPCM$_{(DCM)}$M06-2X/6-311++G(2d,2p)] = -731.3339313

C	1.85100300	-0.87752800	0.16285700
H	2.83568300	-0.41605600	0.35801200
C	0.64361300	-0.00294100	0.46470400
O	1.75342600	-2.00654500	-0.25203400
C	-0.65978200	-0.54897700	-0.08583300
H	-0.58573500	-0.62038200	-1.17752500
C	-1.89068300	0.25927800	0.31780000
H	-1.92701900	0.33945500	1.41147400
H	-1.80392700	1.27950400	-0.07116600
H	-0.74936600	-1.57511100	0.29135100
C	-3.17803900	-0.38083200	-0.19765600
H	-3.30122900	-1.39166600	0.20411100
H	-4.05406200	0.20596500	0.09049200
H	-3.16572500	-0.45356800	-1.28993000
H	0.59195400	0.13274400	1.55109400

Cl	1.03149200	1.63867500	-0.19546300

Cyclohexanone Enamine

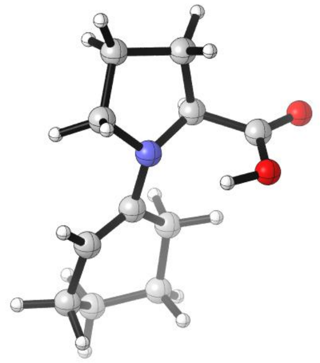

- Thermochemistry -

Zero-point correction=	0.273667 (Hartree/Particle)
Thermal correction to Energy=	0.284624
Thermal correction to Enthalpy=	0.285489
Thermal correction to Gibbs Free Energy=	0.238392
Sum of electronic and zero-point Energies=	-634.107628
Sum of electronic and thermal Energies=	-634.096671
Sum of electronic and thermal Enthalpies=	-634.095806
Sum of electronic and thermal Free Energies=	-634.142903

Number of Imaginary Frequencies = 0

E (Single Point Energy) [IEFPCM$_{(DCM)}$M06-2X/6-311++G(2d,2p)] = -634.55147535

C	-1.82195600	1.11295500	0.60857400
H	-1.53065100	2.00743300	1.15254600
C	-0.86893400	0.31843700	0.08450900
N	0.50219300	0.64457600	0.17935600
C	1.52533600	-0.13928100	-0.50793400
H	1.26454400	-0.39338700	-1.54236100
C	0.90391500	2.05917500	0.29321800
H	1.09601800	2.32552600	1.33963000
H	0.09895100	2.70108000	-0.07729900
C	2.16670900	2.16745400	-0.56331200
H	2.84630600	2.94751600	-0.21495100
H	1.89270700	2.38510000	-1.60048900
C	2.77115200	0.76866800	-0.46975400
H	3.46659400	0.52461600	-1.27363200

H	3.29080200	0.64607400	0.48753700
C	-1.20813400	-0.97782600	-0.62247900
H	-1.03547100	-0.85379700	-1.70047700
H	-0.53671900	-1.78009000	-0.29623000
C	-3.60717500	-0.23343600	-0.54792400
C	1.84596700	-1.43833300	0.22161200
O	1.50430300	-1.46277000	1.51338400
O	2.41933300	-2.36724700	-0.29892800
H	1.02403400	-0.63129700	1.70301300
C	-2.65327600	-1.41167600	-0.37333700
C	-3.30092600	0.83560900	0.49995500
H	-2.90726500	-2.23166200	-1.05172400
H	-2.74331500	-1.79790900	0.65020300
H	-3.69314100	0.51417800	1.47602000
H	-3.82836000	1.76531900	0.25643500
H	-4.64847900	-0.56023100	-0.46503100
H	-3.47705100	0.18915800	-1.55310100

Dioxane Enamine

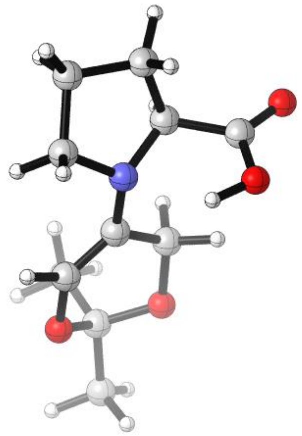

- Thermochemistry -

Zero-point correction=	0.281659 (Hartree/Particle)
Thermal correction to Energy=	0.294503
Thermal correction to Enthalpy=	0.295368
Thermal correction to Gibbs Free Energy=	0.244077
Sum of electronic and zero-point Energies=	-784.485037
Sum of electronic and thermal Energies=	-784.472193
Sum of electronic and thermal Enthalpies=	-784.471328
Sum of electronic and thermal Free Energies=	-784.522619

Number of Imaginary Frequencies = 0

E (Single Point Energy) [IEFPCM$_{(DCM)}$M06-2X/6-311++G(2d,2p)] = -784.98483791

C	1.22503300	1.21865900	-0.76338000
H	1.00442800	2.16629800	-1.23735600
C	0.30007300	0.36278500	-0.30177000
N	-1.08975000	0.61880300	-0.35293300
C	-1.98615800	-0.14099200	0.52122700
H	-1.60401700	-0.27174100	1.54163100
C	-1.53015800	2.02677100	-0.45187600
H	-1.77966400	2.27044100	-1.49000200
H	-0.72493500	2.69425100	-0.12831800
C	-2.74187500	2.12835100	0.47919800
H	-3.48394400	2.84537400	0.12365600
H	-2.41667800	2.43674400	1.47746700
C	-3.27731200	0.69943900	0.53063000
H	-3.88699500	0.48231200	1.40835600
H	-3.86759100	0.48039200	-0.36634500
C	0.75393400	-0.97387300	0.23585400
H	0.60125300	-1.04343900	1.32333700
H	0.20111700	-1.79321800	-0.23344900
O	2.57637300	0.96536100	-0.72609700
O	2.11077200	-1.21055500	-0.09341900
C	2.96379300	-0.10522500	0.13306500
C	4.34641200	-0.53267600	-0.30857900
H	4.71319700	-1.32809000	0.34357000
H	5.02916200	0.31780500	-0.25614200
C	2.93046000	0.35712800	1.58718100
H	3.70484200	1.11143200	1.74177900
H	3.12643700	-0.49369600	2.24479000
H	1.96638700	0.79812300	1.85011200
C	-2.30638100	-1.51802100	-0.04765400
O	-2.15580900	-1.63013200	-1.37052300
O	-2.72550100	-2.43111200	0.62417000
H	4.30228500	-0.89834500	-1.33654400
H	-1.77064900	-0.78883700	-1.69243300

Tetrahydro-4H-thiopyranone Enamine

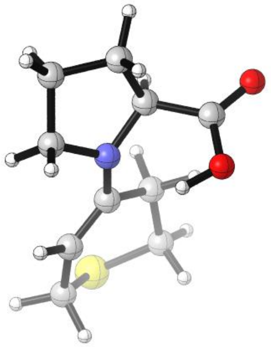

- Thermochemistry -

Zero-point correction=	0.246271 (Hartree/Particle)
Thermal correction to Energy=	0.257547
Thermal correction to Enthalpy=	0.258412
Thermal correction to Gibbs Free Energy=	0.210205
Sum of electronic and zero-point Energies=	-992.992669
Sum of electronic and thermal Energies=	-992.981393
Sum of electronic and thermal Enthalpies=	-992.980528
Sum of electronic and thermal Free Energies=	-993.028735

Number of Imaginary Frequencies = 0

E (Single Point Energy) [IEFPCM$_{(DCM)}$M06-2X/6-311++G(2d,2p)] = -993.43210840

C	-1.49100900	1.13942000	0.73968100
H	-1.13785700	2.01687300	1.27476400
C	-0.58847400	0.33055500	0.15152200
N	0.78358500	0.65217600	0.16756000
C	1.78155700	-0.15905300	-0.52510400
H	1.48906500	-0.44159900	-1.54324900
C	1.20425000	2.06361500	0.24844400
H	1.44165800	2.34092500	1.28276000
H	0.39152700	2.70890800	-0.09819200
C	2.43336000	2.14324000	-0.65756200
H	3.13020900	2.92630600	-0.35305500
H	2.12090300	2.33830600	-1.68821100
C	3.03247200	0.74297900	-0.55227900
H	3.69713800	0.47529100	-1.37434000

H	3.58529000	0.63967000	0.38855400
C	-0.95257000	-0.96013700	-0.55231000
H	-0.82514400	-0.82984500	-1.63398100
H	-0.26322800	-1.75787300	-0.25035900
C	2.11859800	-1.43900800	0.23105100
O	1.82486100	-1.42603900	1.53523200
O	2.66301700	-2.38761100	-0.28395800
H	1.36333100	-0.58592300	1.72756700
C	-2.36301500	-1.45150000	-0.25442500
C	-2.98471500	0.95811700	0.75543500
H	-2.61529200	-2.29150000	-0.90407700
H	-2.44404600	-1.78851900	0.78398000
H	-3.32967400	0.59122400	1.72980300
H	-3.47666900	1.91963000	0.58466200
S	-3.60362800	-0.15795400	-0.53852800

Tetrahydropyranone Enamine

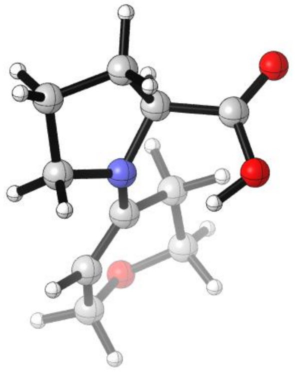

- Thermochemistry -

Zero-point correction=	0.250021 (Hartree/Particle)
Thermal correction to Energy=	0.260784
Thermal correction to Enthalpy=	0.261649
Thermal correction to Gibbs Free Energy=	0.214793
Sum of electronic and zero-point Energies=	-670.020084
Sum of electronic and thermal Energies=	-670.009321
Sum of electronic and thermal Enthalpies=	-670.008456
Sum of electronic and thermal Free Energies=	-670.055312

Number of Imaginary Frequencies = 0

E (Single Point Energy) [IEFPCM$_{(DCM)}$M06-2X/6-311++G(2d,2p)] = -670.45469021

C	-1.84748200	1.11944800	0.54926200

H	-1.61858700	2.03104300	1.09210100
C	-0.87250100	0.32722400	0.06917600
N	0.49187200	0.64484200	0.18716000
C	1.51644600	-0.13172600	-0.50807100
H	1.25648400	-0.36663000	-1.54708400
C	0.89707900	2.05592400	0.33087600
H	1.08649400	2.29825900	1.38325100
H	0.09573100	2.70754100	-0.02999100
C	2.16230300	2.17571300	-0.52049600
H	2.84295500	2.94733000	-0.15611600
H	1.89128100	2.41279100	-1.55407900
C	2.76348100	0.77376900	-0.45068200
H	3.45972400	0.54304500	-1.25765200
H	3.28107200	0.63272200	0.50509500
C	-1.22541200	-0.97606500	-0.61290100
H	-1.06621500	-0.88887500	-1.69451400
H	-0.58924600	-1.79542500	-0.25926400
C	1.83030100	-1.44411100	0.20009500
O	1.49164400	-1.48983700	1.49242700
O	2.39584100	-2.36725200	-0.33791100
H	1.01892200	-0.66002600	1.70340000
C	-2.67637800	-1.34381300	-0.32458700
C	-3.30733600	0.78945400	0.38104700
H	-3.00941200	-2.15333000	-0.97653600
H	-2.77984800	-1.67071000	0.72142900
H	-3.75364800	0.49936300	1.34734900
H	-3.85665200	1.66193200	0.01545700
O	-3.53063000	-0.24139000	-0.56517600

(*R*)-**TS1**$_{P}$-Cl-Pre

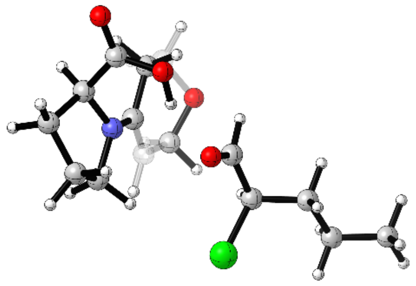

- Thermochemistry -

Zero-point correction= 0.385419 (Hartree/Particle)
Thermal correction to Energy= 0.404775
Thermal correction to Enthalpy= 0.405640

Thermal correction to Gibbs Free Energy= 0.337270
Sum of electronic and zero-point Energies= -1401.127937
Sum of electronic and thermal Energies= -1401.108582
Sum of electronic and thermal Enthalpies= -1401.107717
Sum of electronic and thermal Free Energies= -1401.176087

Number of Imaginary Frequencies = 0

E (Single Point Energy) [IEFPCM$_{(DCM)}$M06-2X/6-311++G(2d,2p)] = -1401.806718

C	-0.20397600	1.31198900	-1.49299500
H	0.05171200	0.61107300	-2.28157100
C	-1.21064800	1.06121000	-0.62808400
N	-1.89028100	-0.14796900	-0.59004800
C	-3.23899300	-0.27292800	-0.04565300
H	-3.86139500	0.60637300	-0.23759300
C	-1.61105400	-1.18237700	-1.58675700
H	-0.56018100	-1.48079500	-1.52804800
H	-1.81428200	-0.80918300	-2.60326600
C	-2.57916000	-2.29469300	-1.20147000
H	-2.17064500	-2.87067100	-0.36393400
H	-2.77954500	-2.98119300	-2.02577300
C	-3.82152600	-1.51139600	-0.76851700
H	-4.37520300	-1.17152900	-1.64803700
H	-4.50632900	-2.07452600	-0.13166000
C	-1.55723400	2.06413700	0.44981600
H	-2.63841400	2.23406500	0.49349300
H	-1.26071600	1.66398400	1.42931000
C	1.12757900	-0.30719700	0.77272800
H	0.96844300	0.62496500	1.34567600
C	2.41510900	-0.31424000	-0.01787800
O	0.36208700	-1.24269800	0.86113900
C	-3.25891400	-0.48774500	1.46984100

O	-2.13940300	-0.91753100	2.04385000
O	-4.26753300	-0.31430900	2.11954600
C	3.59669700	-0.46879000	0.94553400
H	3.47036300	0.27902600	1.74013000
C	4.95701900	-0.26970900	0.28023600
H	5.08719600	-1.01495300	-0.51135600
H	4.97877300	0.71570000	-0.20114200
H	2.49394100	0.63697400	-0.55163800
H	3.53891100	-1.45659000	1.41831700
C	6.09709600	-0.38187200	1.29009500
H	5.99957400	0.37232500	2.07746500
H	6.09956500	-1.36729000	1.76669300
H	7.06637000	-0.24018800	0.80535100
H	-1.39092700	-0.97865000	1.40800300
C	-0.86380200	3.39846800	0.19333800
C	0.60652400	2.57964700	-1.41419800
H	-1.36978700	3.93652900	-0.62225600
H	-0.88413800	4.02440000	1.08700100
H	1.67127800	2.37502000	-1.56710900
H	0.30300200	3.29359300	-2.19813000
O	0.49818600	3.20435100	-0.14654600
Cl	2.39761200	-1.60460000	-1.26292500

(*R*)-**TS1**P-Cl

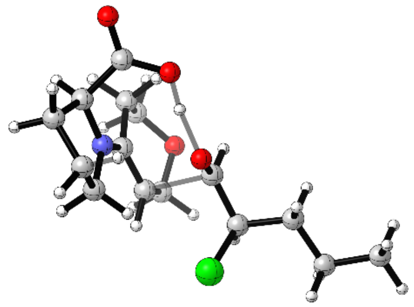

- Thermochemistry -

Zero-point correction=	0.385333 (Hartree/Particle)
Thermal correction to Energy=	0.403086
Thermal correction to Enthalpy=	0.403951
Thermal correction to Gibbs Free Energy=	0.340985
Sum of electronic and zero-point Energies=	-1401.120513
Sum of electronic and thermal Energies=	-1401.102760
Sum of electronic and thermal Enthalpies=	-1401.101895
Sum of electronic and thermal Free Energies=	-1401.164861

Number of Imaginary Frequencies = 1

E (Single Point Energy) [IEFPCM$_{(DCM)}$M06-2X/6-311++G(2d,2p)] = -1401.797805

C	-0.01721200	1.26192200	-1.16558600
H	0.31339200	0.64254500	-1.99428700
C	-1.25845400	1.02311600	-0.59006900
N	-1.87326400	-0.16200300	-0.70079200
C	-3.10967800	-0.52428800	-0.00377300
H	-3.87573500	0.24511100	-0.12410800
C	-1.44157600	-1.21153000	-1.64311000
H	-0.36322000	-1.35133000	-1.58583200
H	-1.71877400	-0.91325400	-2.66312100
C	-2.22088900	-2.43487900	-1.17515500
H	-1.68879200	-2.91120100	-0.34478400
H	-2.35295000	-3.17035700	-1.97005500

C	-3.54282800	-1.83310400	-0.69482500
H	-4.17886700	-1.58693700	-1.54978300
H	-4.10841900	-2.47649500	-0.01940500
C	-1.83503500	2.03626800	0.36965200
H	-2.92413600	2.07788400	0.27988400
H	-1.60629800	1.72061900	1.39722000
C	0.99635400	0.13886200	0.42867500
H	0.93290100	1.03312100	1.07077300
C	2.37194900	-0.01671600	-0.20628200
O	0.33709200	-0.89403400	0.71047300
C	-2.95858100	-0.73223900	1.51301300
O	-1.76466600	-0.89530200	2.03084200
O	-3.96776400	-0.76703300	2.19339300
C	3.37212400	-0.40242600	0.88563300
H	3.26753600	0.33739000	1.69111000
C	4.82540400	-0.43005300	0.41694900
H	4.93921000	-1.17818300	-0.37454600
H	5.07999300	0.54170700	-0.02428300
H	2.68887300	0.90854300	-0.68945300
H	3.07941900	-1.37382700	1.30125100
C	5.78098600	-0.74440200	1.56642800
H	5.70424800	0.00839100	2.35756900
H	5.55091100	-1.71941200	2.00777700
H	6.81817100	-0.76729000	1.22201700
H	-0.93459900	-0.85523500	1.40286200
C	-1.25317900	3.42315800	0.11762500
C	0.57117200	2.65399800	-1.10950300
H	-1.65612900	3.84166900	-0.81636500

H	-1.50361800	4.09782300	0.93742500
H	1.66376300	2.62061300	-1.06924100
H	0.29952600	3.22541100	-2.01167300
O	0.15851500	3.36467500	0.04279800
Cl	2.34692300	-1.26916900	-1.50608700

(*R*)-**TS1**$_{P}$-Cl-P

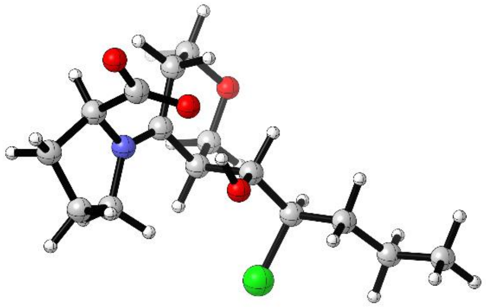

- Thermochemistry -

Zero-point correction= 0.389647 (Hartree/Particle)
Thermal correction to Energy= 0.407384
Thermal correction to Enthalpy= 0.408249
Thermal correction to Gibbs Free Energy= 0.344987
Sum of electronic and zero-point Energies= -1401.148222
Sum of electronic and thermal Energies= -1401.130485
Sum of electronic and thermal Enthalpies= -1401.129620

Number of Imaginary Frequencies = 0

E (Single Point Energy) [IEFPCM$_{(DCM)}$M06-2X/6-311++G(2d,2p)] = -1401.829317

C	0.16571600	1.02749700	-0.80302600
H	0.24865100	0.53896100	-1.77545100
C	-1.23826200	0.94891400	-0.28206800
N	-2.03544100	0.00873700	-0.65943500
C	-3.30273200	-0.31966200	0.03120900
H	-3.92013200	0.57285400	0.13708200

C	-1.72372400	-1.01815800	-1.70011100
H	-0.68857400	-1.33896900	-1.60312700
H	-1.88913900	-0.55084600	-2.67573100
C	-2.73431600	-2.12693500	-1.42187600
H	-2.33731900	-2.80724000	-0.66211900
H	-2.95142800	-2.70149900	-2.32300700
C	-3.94527800	-1.36825200	-0.87755800
H	-4.49103100	-0.87978100	-1.69079200
H	-4.63909700	-1.99059700	-0.31237700
C	-1.60056200	1.91949600	0.80175400
H	-2.67849600	1.99076600	0.94914600
H	-1.17327600	1.52191900	1.73148200
C	1.03924900	0.23688600	0.23008100
H	1.05789300	0.81918300	1.16498800
C	2.50265700	0.11793000	-0.19552100
O	0.53488400	-1.05206200	0.44681100
C	-2.99976600	-0.88751100	1.46546000
O	-1.79280500	-0.98668100	1.81412100
O	-4.01679300	-1.17887500	2.11150600
C	3.32782000	-0.66957800	0.81314400
H	3.12072700	-0.23168900	1.79957800
C	4.83171800	-0.63213600	0.55120200
H	5.04361500	-1.07527300	-0.42787900
H	5.16644200	0.41201700	0.50579000
H	2.93171700	1.11345700	-0.32816800
H	2.96345800	-1.70124400	0.84166900
C	5.61081500	-1.37808300	1.63282500
H	5.43484300	-0.93520200	2.61854100

H	5.30391700	-2.42795900	1.67843800
H	6.68604500	-1.34979200	1.43755000
H	-0.29875600	-1.02528200	0.97869900
C	-1.01196700	3.30819500	0.52154200
C	0.59086200	2.49596800	-0.95597200
H	-1.54036600	3.78589500	-0.31573800
H	-1.11963100	3.93620800	1.40602100
H	1.65439400	2.56644100	-1.18900600
H	0.03359200	2.95086300	-1.78739300
O	0.36947300	3.22779500	0.23081900
Cl	2.61756400	-0.66254100	-1.83733500

(*S*)-**TS1**$_{P}$-Cl-Pre

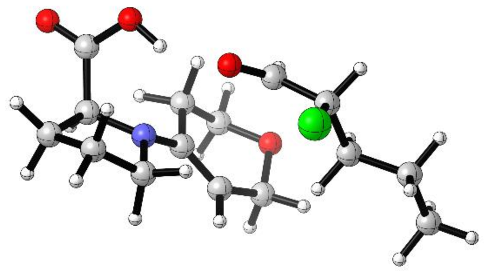

- Thermochemistry -

Zero-point correction=	0.385404 (Hartree/Particle)
Thermal correction to Energy=	0.404710
Thermal correction to Enthalpy=	0.405575
Thermal correction to Gibbs Free Energy=	0.337081
Sum of electronic and zero-point Energies=	-1401.127303
Sum of electronic and thermal Energies=	-1401.107997
Sum of electronic and thermal Enthalpies=	-1401.107132
Sum of electronic and thermal Free Energies=	-1401.175625

Number of Imaginary Frequencies = 0

E (Single Point Energy) [IEFPCM$_{(DCM)}$M06-2X/6-311++G(2d,2p)] = -1401.805905

C	0.23958100	1.43001400	-1.21841800

H	0.54893400	0.80916400	-2.05331000
C	-0.88396900	1.16087000	-0.52397000
N	-1.64696900	0.01202300	-0.72046600
C	-3.09817700	0.02171900	-0.54240300
H	-3.55543600	0.97600100	-0.82411800
C	-1.24229500	-0.96921900	-1.72536900
H	-0.25715200	-1.37456200	-1.47054800
H	-1.18451200	-0.51331300	-2.72688400
C	-2.36396700	-1.99820800	-1.66013700
H	-2.21712600	-2.65506900	-0.79592800
H	-2.42209900	-2.61578300	-2.55800600
C	-3.59919400	-1.11294200	-1.47026400
H	-3.89549100	-0.67500800	-2.42729800
H	-4.46330000	-1.63373500	-1.05323600
C	-1.31034500	2.05766800	0.61669000
H	-2.37676300	2.30132900	0.54736400
H	-1.17108100	1.52584400	1.56790100
C	0.97694500	-0.75688800	1.32078500
H	0.89212800	0.26167200	1.74374600
C	2.39811900	-1.15740800	0.99405300
O	0.00527500	-1.46354600	1.17820900
C	-3.52819200	-0.25124700	0.89988700
O	-2.63974000	-0.82854100	1.70165200
O	-4.64783300	0.00669500	1.28722200
C	3.10253600	-0.07557200	0.17939700
H	2.63996200	-0.02760200	-0.81410000
C	4.61109300	-0.27218800	0.05707200
H	5.04790500	-0.33517500	1.06129200

H	4.81618700	-1.22600600	-0.44033800
H	2.91164900	-1.28668100	1.95544400
H	2.89900500	0.88331800	0.67672200
C	5.25856800	0.87028400	-0.72267400
H	5.08674900	1.83067200	-0.22555700
H	6.33812500	0.72556400	-0.81233100
H	4.84275200	0.93603700	-1.73354800
H	-1.76489400	-0.93812100	1.26433900
C	-0.50959700	3.35599500	0.61557000
C	1.10703100	2.61701500	-0.88758600
H	-0.86993700	4.02204500	-0.18282100
H	-0.60865200	3.87441300	1.57080700
H	2.16759800	2.35276100	-0.93546800
H	0.94314400	3.43488200	-1.60919900
O	0.87116100	3.09878200	0.42435500
Cl	2.44243300	-2.75078200	0.17594300

(*S*)-**TS1**$_{P}$-Cl

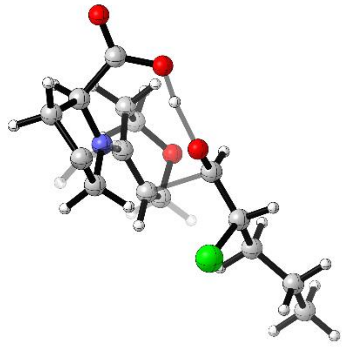

- Thermochemistry -

Zero-point correction= 0.385331 (Hartree/Particle)
Thermal correction to Energy= 0.403019
Thermal correction to Enthalpy= 0.403884

Thermal correction to Gibbs Free Energy= 0.341063
Sum of electronic and zero-point Energies= -1401.114733
Sum of electronic and thermal Energies= -1401.097045
Sum of electronic and thermal Enthalpies= -1401.096180
Sum of electronic and thermal Free Energies= -1401.159001

Number of Imaginary Frequencies = 1

E (Single Point Energy) [IEFPCM$_{(DCM)}$M06-2X/6-311++G(2d,2p)] = -1401.791734

C	0.44342800	0.86301500	-0.97364200
H	0.79525700	0.06742600	-1.62652000
C	-0.91873400	0.94483300	-0.68591000
N	-1.73418800	-0.10922900	-0.78732200
C	-3.14459600	-0.11356900	-0.38357500
H	-3.67814500	0.74205600	-0.80356500
C	-1.34989400	-1.36895900	-1.45054400
H	-0.36379500	-1.68504200	-1.11665900
H	-1.33662600	-1.21045400	-2.53708200
C	-2.45303000	-2.33158200	-1.02853400
H	-2.22599500	-2.73495000	-0.03590300
H	-2.56421400	-3.16400900	-1.72485800
C	-3.68738400	-1.43012000	-0.97554000
H	-4.05791100	-1.23695300	-1.98622600
H	-4.50863600	-1.82944800	-0.37890600
C	-1.45677300	2.18714900	-0.01491200
H	-2.48939300	2.37956900	-0.31701100
H	-1.46368200	2.01657300	1.07080300
C	0.86351200	-0.14971200	0.93989700
H	0.96432500	0.84399000	1.40550200
C	2.21276400	-0.84093000	0.72624300
O	-0.11405700	-0.89203900	1.22192400

C	-3.38629100	-0.05495500	1.13465600
O	-2.39758100	-0.29716200	1.96001700
O	-4.51159500	0.19415400	1.52773500
C	3.39051200	0.01049000	0.28371900
H	3.31216900	0.22034100	-0.78882300
C	4.74444900	-0.63509000	0.57823100
H	4.81856500	-0.83785500	1.65366600
H	4.80476800	-1.60277800	0.06810500
H	2.43058400	-1.26465600	1.71519900
H	3.32768000	0.96868300	0.81763600
C	5.90369400	0.25548200	0.13692600
H	5.87360200	1.22125800	0.65177000
H	6.86701200	-0.21262800	0.35497400
H	5.85908400	0.44776400	-0.93996400
H	-1.45329400	-0.49820300	1.56543700
C	-0.60145400	3.40811100	-0.33105500
C	1.22842300	2.16147400	-1.04412700
H	-0.75379100	3.72191500	-1.37443600
H	-0.86643500	4.23938100	0.32394000
H	2.28220200	2.01015500	-0.82019100
H	1.16803500	2.58124000	-2.06195000
O	0.76617300	3.12383100	-0.11515500
Cl	2.04250900	-2.28722700	-0.34299100

(*S*)-**TS1**$_{P}$-Cl-P

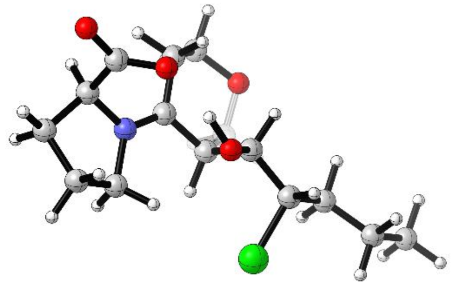

- Thermochemistry -

Zero-point correction=	0.389760 (Hartree/Particle)
Thermal correction to Energy=	0.407413
Thermal correction to Enthalpy=	0.408278
Thermal correction to Gibbs Free Energy=	0.345083
Sum of electronic and zero-point Energies=	-1401.143755
Sum of electronic and thermal Energies=	-1401.126102
Sum of electronic and thermal Enthalpies=	-1401.125237
Sum of electronic and thermal Free Energies=	-1401.188431

Number of Imaginary Frequencies = 0

E (Single Point Energy) [IEFPCM$_{(DCM)}$M06-2X/6-311++G(2d,2p)] = -1401.825061

C	0.44699100	0.52665400	-0.71285200
H	0.58001000	-0.26396500	-1.45565600
C	-1.01457200	0.81878100	-0.51557700
N	-1.90598500	-0.08379000	-0.74224000
C	-3.32538400	0.03511100	-0.33731900
H	-3.74276100	0.97214700	-0.70791400
C	-1.62057700	-1.45486400	-1.26276500
H	-0.71438800	-1.84078700	-0.79849700
H	-1.48757600	-1.37195100	-2.34598500
C	-2.87708400	-2.24146200	-0.90177900
H	-2.78996700	-2.63011500	0.11756200
H	-3.02962400	-3.08056600	-1.58145500

C	-3.98267000	-1.18796100	-0.97768400
H	-4.24101900	-0.97607900	-2.01988900
H	-4.88975600	-1.45946200	-0.43788900
C	-1.35854700	2.14527500	0.09260700
H	-2.41556300	2.38956900	-0.01402900
H	-1.15874100	2.04834700	1.16799500
C	0.98403800	0.01775900	0.67138700
H	1.15527800	0.90106000	1.30680500
C	2.32575400	-0.72381600	0.60661800
O	0.09567300	-0.88224500	1.28367800
C	-3.45132200	0.02531500	1.22900400
O	-2.38910200	-0.04393900	1.90228000
O	-4.62069900	0.09872400	1.63451000
C	3.56636100	0.07539400	0.22734400
H	3.60183300	0.22873000	-0.85679600
C	4.86292500	-0.59808900	0.67807300
H	4.83758100	-0.72916000	1.76656700
H	4.92155400	-1.60030700	0.23878200
H	2.45664400	-1.15092700	1.60423900
H	3.49000900	1.06508200	0.69851700
C	6.09331900	0.21417200	0.28115300
H	6.06205200	1.21458700	0.72492600
H	7.01289600	-0.27348100	0.61465700
H	6.14946100	0.33098700	-0.80596000
H	-0.77505800	-0.48194900	1.52727200
C	-0.48674500	3.26689600	-0.47954300
C	1.15147600	1.79692900	-1.22656700
H	-0.76594000	3.48090300	-1.52083900

H	-0.61976500	4.17286700	0.11200100
H	2.23081700	1.66628300	-1.23857500
H	0.82336900	1.99513200	-2.25730700
O	0.88080200	2.91754300	-0.41321000
Cl	2.17843400	-2.17868400	-0.47844300

(*R*)-**TS1**$_G$-Cl-Pre

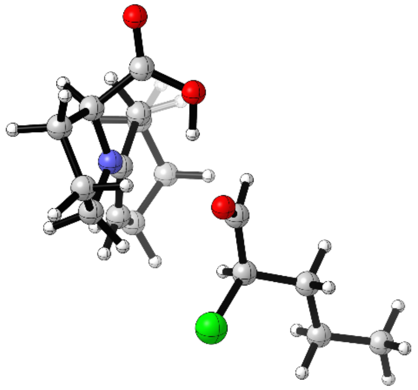

- Thermochemistry -

Zero-point correction= 0.409291 (Hartree/Particle)
Thermal correction to Energy= 0.428788
Thermal correction to Enthalpy= 0.429653
Thermal correction to Gibbs Free Energy= 0.361196
Sum of electronic and zero-point Energies= -1365.215397
Sum of electronic and thermal Energies= -1365.195900
Sum of electronic and thermal Enthalpies= -1365.195035
Sum of electronic and thermal Free Energies= -1365.263491

Number of Imaginary Frequencies = 0

E (Single Point Energy) [IEFPCM$_{(DCM)}$M06-2X/6-311++G(2d,2p)] = -1365.903561

C	-0.16740300	1.27862100	-1.50042600
H	0.05084400	0.53442300	-2.26105600
C	-1.17615400	1.06372100	-0.62591000
N	-1.87834800	-0.14169000	-0.58495800

C	-3.23261200	-0.23831900	-0.04865900
H	-3.84377200	0.64551300	-0.25475100
C	-1.62635200	-1.17665800	-1.58808100
H	-0.58038100	-1.49281700	-1.54467100
H	-1.83605200	-0.79846000	-2.60194300
C	-2.60338700	-2.27745700	-1.19436800
H	-2.19387600	-2.85707000	-0.35967100
H	-2.81815700	-2.96291300	-2.01609100
C	-3.83312800	-1.47964200	-0.75310500
H	-4.39492400	-1.14323700	-1.62876200
H	-4.51553800	-2.03224500	-0.10411100
C	-1.53723900	2.07682300	0.44155400
H	-2.62399700	2.19725800	0.49699800
H	-1.22775600	1.68362700	1.42192300
C	0.57588700	3.29488200	-0.18844700
C	1.07772000	-0.47012400	0.80541600
H	0.89939400	0.40026500	1.46494700
C	2.36523500	-0.37477600	0.02106600
O	0.32342900	-1.41757600	0.80514000
C	-3.25510900	-0.43171000	1.46880000
O	-2.15582200	-0.92544500	2.03351600
O	-4.24280700	-0.19196700	2.12945500
C	3.55475000	-0.53648000	0.97273600
H	3.39904200	0.15793700	1.80969700
C	4.90332900	-0.24015200	0.31984000
H	5.06615800	-0.93542300	-0.51016400
H	4.87900000	0.76929900	-0.10902800
H	2.39902800	0.61183000	-0.45139400

H	3.54165200	-1.55151700	1.38751900
C	6.04912600	-0.35307900	1.32307500
H	6.09680300	-1.36086700	1.74765100
H	7.01023000	-0.14279200	0.84708300
H	5.91900100	0.35379300	2.14873500
H	-1.42813600	-1.03758400	1.38135800
Cl	2.39955500	-1.58063100	-1.30443200
C	-0.89300600	3.44318100	0.19907500
C	0.68396800	2.52472700	-1.50348700
H	-1.42466300	3.95482700	-0.61325300
H	-1.00474500	4.06002400	1.09557700
H	1.72962700	2.25980100	-1.70895600
H	0.38378900	3.18029000	-2.33382300
H	1.10721700	2.75224800	0.60701200
H	1.05406300	4.27490000	-0.28014600

(*R*)-**TS1**$_G$-Cl

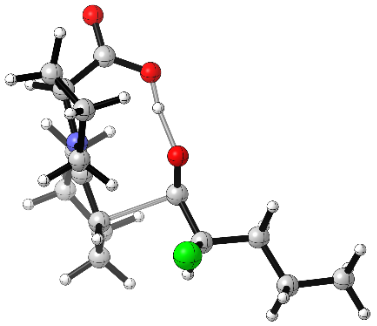

- Thermochemistry -

Zero-point correction=	0.409354 (Hartree/Particle)
Thermal correction to Energy=	0.427259
Thermal correction to Enthalpy=	0.428124
Thermal correction to Gibbs Free Energy=	0.365060
Sum of electronic and zero-point Energies=	-1365.207715

Sum of electronic and thermal Energies= -1365.189810
Sum of electronic and thermal Enthalpies= -1365.188945
Sum of electronic and thermal Free Energies= -1365.252009

Number of Imaginary Frequencies = 1

E (Single Point Energy) [IEFPCM$_{(DCM)}$M06-2X/6-311++G(2d,2p)] = -1365.894619

C	-0.02497100	1.27173900	-1.14946500
H	0.29999900	0.62051000	-1.95584500
C	-1.26142500	1.01955300	-0.56907500
N	-1.86151600	-0.17845700	-0.69570800
C	-3.09794100	-0.56403300	-0.01241300
H	-3.87883700	0.18931000	-0.13796100
C	-1.42024300	-1.20905700	-1.65411200
H	-0.34070300	-1.33846700	-1.60692600
H	-1.70714300	-0.90231700	-2.66940100
C	-2.17612700	-2.45116500	-1.19864100
H	-1.63262600	-2.92671300	-0.37514600
H	-2.29743600	-3.18028600	-2.00127500
C	-3.50662000	-1.87888200	-0.70802100
H	-4.15215200	-1.64135200	-1.55832700
H	-4.05610400	-2.53793300	-0.03406900
C	-1.87577700	2.01270500	0.39219600
H	-2.96582300	1.95116200	0.35108800
H	-1.58804800	1.72060200	1.41421900
C	0.08977600	3.53017300	0.01082600
C	0.99380400	0.09717000	0.44476200
H	0.92123700	0.96493200	1.12029900
C	2.37253000	-0.01850200	-0.18927800
O	0.33923500	-0.94610500	0.68922600

C	-2.95543100	-0.77058900	1.50419300
O	-1.76261800	-0.94733400	2.02292800
O	-3.96411100	-0.79560400	2.18598500
C	3.37682500	-0.41995700	0.89341800
H	3.25789600	0.29280400	1.72110200
C	4.83145300	-0.40731000	0.42816800
H	4.96054500	-1.12951000	-0.38472400
H	5.06883800	0.58152500	0.01612900
H	2.67243900	0.92673600	-0.64343000
H	3.10083900	-1.40861700	1.27878000
C	5.79052600	-0.73771600	1.57021400
H	5.57800300	-1.72956300	1.98186300
H	6.82866500	-0.73085100	1.22798100
H	5.69812100	-0.01040700	2.38325500
H	-0.93945700	-0.90998000	1.39077900
Cl	2.37214700	-1.23310900	-1.52463800
C	-1.42857800	3.44947500	0.12012700
C	0.57695300	2.66177200	-1.14921100
H	-1.88093000	3.79826000	-0.81655100
H	-1.80088000	4.09640300	0.91939500
H	1.67138000	2.59980000	-1.13601300
H	0.32721200	3.15740300	-2.09768800
H	0.53852800	3.19398300	0.95468000
H	0.41197400	4.56470700	-0.14064900

(*R*)-**TS1**$_G$-Cl-P

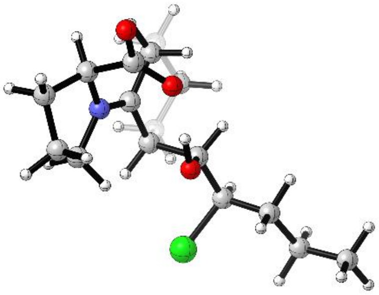

- Thermochemistry -

Zero-point correction=	0.413433 (Hartree/Particle)
Thermal correction to Energy=	0.431419
Thermal correction to Enthalpy=	0.432284
Thermal correction to Gibbs Free Energy=	0.368557
Sum of electronic and zero-point Energies=	-1365.237484
Sum of electronic and thermal Energies=	-1365.219499
Sum of electronic and thermal Enthalpies=	-1365.218634
Sum of electronic and thermal Free Energies=	-1365.282361

Number of Imaginary Frequencies = 0

E (Single Point Energy) [IEFPCM$_{(DCM)}$M06-2X/6-311++G(2d,2p)] = -1365.927951

C	0.16577300	1.02418400	-0.76361800
H	0.21054600	0.53565700	-1.73739600
C	-1.23100500	0.95862900	-0.21316700
N	-2.06197800	0.06616100	-0.63727500
C	-3.31994600	-0.28482900	0.05905400
H	-3.90580000	0.61075800	0.26562600
C	-1.80368400	-0.89248900	-1.75663500
H	-0.78448400	-1.26690600	-1.69329700
H	-1.95172900	-0.34511300	-2.69255000
C	-2.85775400	-1.97801600	-1.55721500
H	-2.47775100	-2.74174900	-0.87169300

H	-3.11583400	-2.45849200	-2.50172400
C	-4.02443300	-1.22395200	-0.91930500
H	-4.56708200	-0.64492000	-1.67312800
H	-4.73051700	-1.86585300	-0.39249400
C	-1.55530600	1.85906600	0.94405300
H	-2.62205900	1.86185700	1.16401300
H	-1.06199100	1.41359300	1.81908800
C	0.43420200	3.31278800	0.34180900
C	1.02292600	0.15778500	0.21987500
H	1.00995000	0.63305300	1.21377300
C	2.50089100	0.10552100	-0.16372400
O	0.52658900	-1.15162400	0.29919800
C	-2.99096600	-0.99082500	1.42339100
O	-1.77698800	-1.14932500	1.72335800
O	-3.99527100	-1.31970900	2.07259100
C	3.30516300	-0.79057900	0.76767600
H	3.05272600	-0.48704500	1.79338100
C	4.81643800	-0.69418800	0.57121500
H	5.07310500	-1.00232100	-0.44810500
H	5.12967400	0.35267100	0.67387300
H	2.91355300	1.11718100	-0.15547100
H	2.96243400	-1.82346600	0.65131600
C	5.57280300	-1.56073300	1.57634800
H	5.28766600	-2.61275100	1.47412000
H	6.65344700	-1.48880100	1.42759700
H	5.35211800	-1.25294900	2.60364400
H	-0.30175200	-1.17323600	0.83866900
Cl	2.68903400	-0.45911400	-1.88529900

C	-1.04199100	3.29229100	0.73106300
C	0.64530200	2.47787300	-0.91990600
H	-1.63299000	3.77503100	-0.05645800
H	-1.21423300	3.85543000	1.65177000
H	1.69200100	2.47887100	-1.23631200
H	0.07962600	2.93097800	-1.74293700
H	1.04553800	2.92914700	1.16852600
H	0.75899200	4.34129000	0.16051600

(*S*)-**TS1**$_{G}$-Cl-Pre

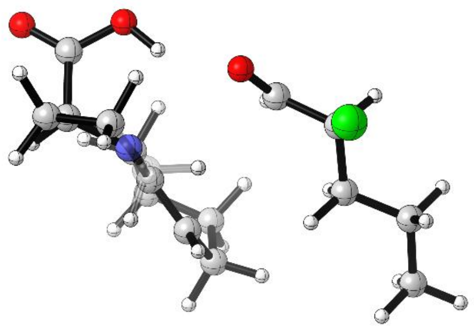

- Thermochemistry -

Zero-point correction=	0.409146 (Hartree/Particle)
Thermal correction to Energy=	0.428587
Thermal correction to Enthalpy=	0.429452
Thermal correction to Gibbs Free Energy=	0.361683
Sum of electronic and zero-point Energies=	-1365.215109
Sum of electronic and thermal Energies=	-1365.195668
Sum of electronic and thermal Enthalpies=	-1365.194803
Sum of electronic and thermal Free Energies=	-1365.262572

Number of Imaginary Frequencies = 0

E (Single Point Energy) [IEFPCM$_{(DCM)}$M06-2X/6-311++G(2d,2p)] = -1365.902931

C	0.26134200	1.44482700	-1.19536100
H	0.58501000	0.77357100	-1.98586300

C	-0.87412200	1.17400400	-0.51747100
N	-1.59481100	-0.01243900	-0.70220200
C	-3.05221400	-0.03911300	-0.58171000
H	-3.52686100	0.88937600	-0.91596300
C	-1.13344200	-1.00375700	-1.67220600
H	-0.14208200	-1.37145900	-1.38726300
H	-1.06737400	-0.57212900	-2.68431600
C	-2.21914300	-2.06987300	-1.60682400
H	-2.07440700	-2.69366600	-0.71794500
H	-2.22915100	-2.71670600	-2.48588700
C	-3.48854600	-1.22282800	-1.48090700
H	-3.77348200	-0.82959100	-2.46058600
H	-4.34582700	-1.75933700	-1.06922000
C	-1.39078300	2.08868700	0.57414500
H	-2.47501700	2.21648500	0.48245700
H	-1.22610100	1.60092300	1.54696200
C	0.95845000	-0.82179400	1.36218700
H	0.86218700	0.17797000	1.82570000
C	2.38171600	-1.18983000	1.00603100
O	-0.00542900	-1.53563600	1.20749200
C	-3.52467300	-0.27152900	0.85377800
O	-2.65583200	-0.82601400	1.69318900
O	-4.65494100	-0.00765600	1.20522900
C	3.06349500	-0.08023900	0.20864000
H	2.59632500	-0.01974800	-0.78132200
C	4.57480400	-0.24888100	0.07831100
H	5.01698000	-0.31975700	1.07966800
H	4.79408300	-1.19139400	-0.43443500

H	2.90906400	-1.33601500	1.95753700
H	2.84741100	0.86780500	0.72158000
C	5.19926400	0.91603300	-0.68674900
H	5.01163200	1.86605900	-0.17541300
H	6.28097800	0.79209500	-0.78148600
H	4.77878700	0.98903600	-1.69514100
H	-1.77345900	-0.94450600	1.27398600
C	-0.71663500	3.46139200	0.55773200
C	1.11254000	2.66593300	-0.94607300
H	-1.11062400	4.04960500	-0.28091400
H	-0.96807100	4.00047000	1.47591200
H	2.17461400	2.39574900	-0.99852100
H	0.95320900	3.39729300	-1.75173400
Cl	2.43766300	-2.76162200	0.14790900
C	0.79487100	3.31963900	0.39769900
H	1.28746500	4.29402100	0.47322300
H	1.18619700	2.69692900	1.21533200

(*S*)-**TS1**$_{G}$-Cl

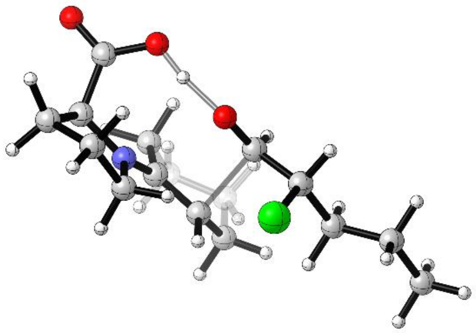

- Thermochemistry -

Zero-point correction= 0.409269 (Hartree/Particle)

Thermal correction to Energy= 0.427110
Thermal correction to Enthalpy= 0.427975
Thermal correction to Gibbs Free Energy= 0.364994
Sum of electronic and zero-point Energies= -1365.201698
Sum of electronic and thermal Energies= -1365.183858
Sum of electronic and thermal Enthalpies= -1365.182993
Sum of electronic and thermal Free Energies= -1365.245974

Number of Imaginary Frequencies = 1

E (Single Point Energy) [IEFPCM$_{(DCM)}$M06-2X/6-311++G(2d,2p)] = -1365.888191

C	0.43962000	0.86969500	-0.97787000
H	0.76146100	0.04701800	-1.61280700
C	-0.91952600	0.95328600	-0.67007700
N	-1.73219200	-0.10823900	-0.77862900
C	-3.14173500	-0.12580700	-0.37203800
H	-3.68552400	0.72438800	-0.78953700
C	-1.34851900	-1.35734000	-1.46220600
H	-0.35766300	-1.67504800	-1.14647600
H	-1.35027300	-1.18635300	-2.54730600
C	-2.43725400	-2.33410900	-1.03720100
H	-2.19444400	-2.74220800	-0.05019300
H	-2.54793200	-3.16236700	-1.73876100
C	-3.67893900	-1.44531000	-0.96326500
H	-4.06449800	-1.25091300	-1.96812400
H	-4.48906900	-1.85603300	-0.35887800
C	-1.47677000	2.18431300	0.01254500
H	-2.53620800	2.30368900	-0.22387700
H	-1.42074300	2.01199800	1.09867200
C	0.85571600	-0.19809700	0.93318500
H	0.95568900	0.77710100	1.43517700
C	2.20834300	-0.87718500	0.71020000

O	-0.12203400	-0.94789100	1.19233700
C	-3.37905800	-0.07053900	1.14611700
O	-2.39023600	-0.33493600	1.96511100
O	-4.49858700	0.19285900	1.54743900
C	3.38178600	-0.00814400	0.29183100
H	3.32111100	0.19678400	-0.78207700
C	4.73788600	-0.63330200	0.61779400
H	4.79627500	-0.82240600	1.69673700
H	4.81775800	-1.60650500	0.12053200
H	2.42376100	-1.32188400	1.69055800
H	3.29158600	0.95273800	0.81764300
C	5.89447800	0.26493200	0.18520400
H	5.84371100	1.23701000	0.68656400
H	6.85938600	-0.18832100	0.42649200
H	5.86665700	0.44264500	-0.89474300
H	-1.45311400	-0.54357500	1.55792900
C	-0.72161900	3.46521500	-0.34045900
C	1.25298700	2.14877100	-1.10750900
H	-0.94012100	3.74228200	-1.37925300
H	-1.08533600	4.27829400	0.29415100
H	2.31198300	1.95259000	-0.94112800
H	1.17821900	2.50356800	-2.14586700
Cl	2.05327600	-2.30188500	-0.39000500
C	0.77935900	3.26333600	-0.17769100
H	1.32017100	4.18767100	-0.40259400
H	1.00313700	3.00900700	0.86720600

(*S*)-**TS1**$_{G}$-Cl-P

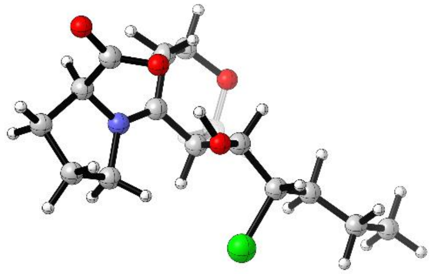

- Thermochemistry -

Zero-point correction=	0.413725 (Hartree/Particle)
Thermal correction to Energy=	0.431652
Thermal correction to Enthalpy=	0.432517
Thermal correction to Gibbs Free Energy=	0.369188
Sum of electronic and zero-point Energies=	-1365.232950
Sum of electronic and thermal Energies=	-1365.215023
Sum of electronic and thermal Enthalpies=	-1365.214158
Sum of electronic and thermal Free Energies=	-1365.277488

Number of Imaginary Frequencies = 0

E (Single Point Energy) [IEFPCM$_{(DCM)}$M06-2X/6-311++G(2d,2p)] = -1365.923664

C	0.33703900	0.18028900	-0.87230900
H	0.26887000	-0.71546700	-1.48971700
C	-1.03974500	0.70547200	-0.56662800
N	-2.07803900	-0.05154000	-0.68717200
C	-3.40315900	0.27472800	-0.11401000
H	-3.71507600	1.27431700	-0.41633100
C	-2.07332700	-1.45084700	-1.21704400
H	-1.20461500	-1.98481800	-0.83941500
H	-2.03506200	-1.38382000	-2.30847900
C	-3.39706500	-2.03065200	-0.72656900
H	-3.26826100	-2.45213500	0.27514300
H	-3.75499300	-2.81878300	-1.39010200

C	-4.31846400	-0.81220200	-0.67603800
H	-4.65371100	-0.54045100	-1.68190600
H	-5.19166200	-0.94477400	-0.03720900
C	-1.12623000	2.09082200	0.00740500
H	-2.15593800	2.44034700	0.06491300
H	-0.76949000	2.01390900	1.04357900
C	0.90297100	-0.27743900	0.51327100
H	0.96399800	0.59067100	1.18614700
C	2.32025600	-0.86968900	0.49158200
O	0.05565000	-1.25704900	1.06905700
C	-3.32337000	0.22765700	1.45466900
O	-2.21020100	-0.05218000	1.97503200
O	-4.39807800	0.48265000	2.01892300
C	3.46625400	0.13163400	0.58737500
H	3.52809600	0.75387200	-0.31096700
C	4.82446000	-0.51660400	0.85351000
H	4.75165300	-1.14801300	1.74770100
H	5.08406900	-1.17728200	0.01969000
H	2.36140200	-1.53715400	1.35570400
H	3.22457300	0.80238600	1.42345500
C	5.92097200	0.52938900	1.04380500
H	5.69577700	1.18451000	1.89159200
H	6.88891100	0.05734200	1.23082100
H	6.01963300	1.15690800	0.15198800
H	-0.76620700	-0.83890500	1.42385400
C	-0.25534000	3.09434200	-0.76745300
C	1.16411300	1.23199500	-1.63048400
H	-0.69684100	3.26839800	-1.75601600

H	-0.27859900	4.04753800	-0.23332200
H	2.16959800	0.84110400	-1.79867300
H	0.71708500	1.35080200	-2.62529800
Cl	2.54727400	-1.98166900	-0.93112300
C	1.17698000	2.59063900	-0.93352500
H	1.75826300	3.30412500	-1.52479000
H	1.66410000	2.51994900	0.04748400

(*R*)-**TS1**$_{O}$-Cl-Pre

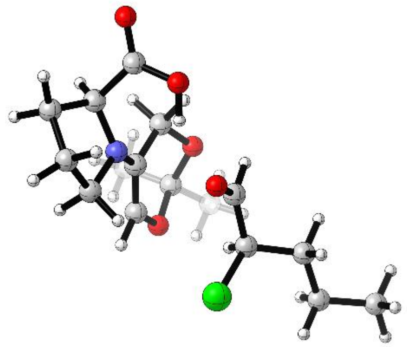

- Thermochemistry -

Zero-point correction=	0.416833 (Hartree/Particle)
Thermal correction to Energy=	0.438270
Thermal correction to Enthalpy=	0.439135
Thermal correction to Gibbs Free Energy=	0.367316
Sum of electronic and zero-point Energies=	-1515.595407
Sum of electronic and thermal Energies=	-1515.573970
Sum of electronic and thermal Enthalpies=	-1515.573105
Sum of electronic and thermal Free Energies=	-1515.644924

Number of Imaginary Frequencies = 0

E (Single Point Energy) [IEFPCM$_{(DCM)}$M06-2X/6-311++G(2d,2p)] = -1516.338860

C	-0.07822600	0.98047700	-1.32356900
H	0.03153800	0.38575100	-2.22177600
C	-1.04756200	0.80339500	-0.40757200

N	-2.00395600	-0.20974100	-0.49395500
C	-3.32924600	-0.03225200	0.09839700
H	-3.62015300	1.01935400	0.19225800
C	-2.06687600	-1.02106800	-1.71107500
H	-1.17159300	-1.64734700	-1.78568900
H	-2.12220200	-0.37757000	-2.60438400
C	-3.35808200	-1.81167600	-1.52972300
H	-3.18736700	-2.66701900	-0.86700200
H	-3.75527300	-2.18362400	-2.47548800
C	-4.27721800	-0.78215000	-0.86473000
H	-4.64813400	-0.07438700	-1.61128700
H	-5.13400200	-1.21418800	-0.34470700
C	-1.02187400	1.63909800	0.84853500
H	-1.79819600	2.41734800	0.84528800
H	-1.18619000	1.01165300	1.73152000
O	0.94531600	1.88788600	-1.17750300
O	0.26458800	2.21301500	1.01703400
C	0.76084000	2.85699900	-0.14263500
C	2.13890900	3.37058500	0.21192300
H	2.61949200	3.78116400	-0.67820400
H	2.05885800	4.15178800	0.97066200
C	-0.17237800	3.95669100	-0.63707200
H	0.32613700	4.51866600	-1.42943100
H	-0.40931700	4.63532000	0.18633200
H	-1.09938900	3.54549100	-1.04327600
C	0.87837800	-1.12508900	0.76081500
H	0.77277800	-0.37923500	1.57227000
C	2.15859600	-0.95706400	-0.02126500

O	0.05059200	-1.99348300	0.59292200
C	-3.41955700	-0.63776300	1.50367200
O	-2.43275500	-1.44704100	1.87947400
O	-4.36200600	-0.41562700	2.23191100
H	2.74419800	2.54843100	0.60238200
C	3.36049700	-1.28680900	0.86844000
H	3.23523800	-0.72650300	1.80531000
C	4.70164800	-0.91500200	0.23925300
H	4.82997100	-1.46785700	-0.69721100
H	4.69072300	0.15135300	-0.01888500
H	3.33597700	-2.35318200	1.12325000
C	5.86698700	-1.21192400	1.18037200
H	5.77067800	-0.64897500	2.11433200
H	5.90277800	-2.27665000	1.43209800
H	6.82160300	-0.94274900	0.72113500
H	-1.73572600	-1.50865900	1.18769600
H	2.21057700	0.08908700	-0.34525600
Cl	2.13234100	-1.95505700	-1.51162100

(*R*)-**TS1**$_{O}$-Cl

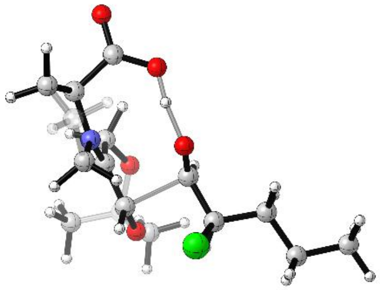

- Thermochemistry -

Zero-point correction= 0.415898 (Hartree/Particle)
Thermal correction to Energy= 0.435797

Thermal correction to Enthalpy= 0.436662
Thermal correction to Gibbs Free Energy= 0.369496
Sum of electronic and zero-point Energies= -1515.585898
Sum of electronic and thermal Energies= -1515.565999
Sum of electronic and thermal Enthalpies= -1515.565134
Sum of electronic and thermal Free Energies= -1515.632299

Number of Imaginary Frequencies = 1

E (Single Point Energy) [IEFPCM$_{(DCM)}$M06-2X/6-311++G(2d,2p)] = -1516.327244

C	0.07380400	0.93626500	-0.94021000
H	0.33080000	0.42941900	-1.86404000
C	-1.17196800	0.76942500	-0.35005500
N	-1.92941200	-0.29760500	-0.60604100
C	-3.16688000	-0.61449600	0.10944400
H	-3.77960200	0.28046700	0.25090100
C	-1.68503900	-1.20785200	-1.74285300
H	-0.64926100	-1.55001300	-1.73889200
H	-1.89138600	-0.66947100	-2.67728400
C	-2.68367500	-2.33571400	-1.49676300
H	-2.24637300	-3.07323100	-0.81557200
H	-2.96456800	-2.84318800	-2.42048300
C	-3.85994700	-1.61944200	-0.82776600
H	-4.44253600	-1.06912700	-1.57209000
H	-4.53139100	-2.28032900	-0.27875600
C	-1.57710300	1.71079900	0.75660400
H	-2.48413700	2.26063000	0.46612400
H	-1.79570900	1.15547700	1.67474900
O	0.81359500	2.08744800	-0.74421500
O	-0.51884700	2.58761500	1.07882000
C	0.14808300	3.13982000	-0.04275700
C	1.23106000	4.04145400	0.50541700

H	1.85377600	4.40748800	-0.31314900
H	0.77870800	4.89109900	1.02079100
C	-0.80711000	3.86686700	-0.98369100
H	-0.22562200	4.39845700	-1.73942900
H	-1.40115600	4.58935600	-0.41823300
H	-1.48123900	3.17855000	-1.49953800
C	0.92567200	-0.41585400	0.42134800
H	0.89290400	0.34314400	1.22250500
C	2.30604900	-0.47854500	-0.21863800
O	0.25017700	-1.48275700	0.52983500
C	-2.96595700	-1.21813200	1.51493300
O	-1.76740400	-1.55204900	1.91315300
O	-3.95430300	-1.36196900	2.21366500
H	1.84952200	3.47831200	1.20773600
C	3.29837900	-1.06535100	0.78493300
H	3.20811300	-0.47249000	1.70557700
C	4.75062100	-1.03725600	0.31261400
H	4.85039100	-1.64110200	-0.59543300
H	5.02085100	-0.00884500	0.04196600
H	2.98957900	-2.08848000	1.02989700
C	5.70268300	-1.55900400	1.38711900
H	5.64078700	-0.95072700	2.29526100
H	5.45603900	-2.59079700	1.65737800
H	6.73877500	-1.54091000	1.03901100
H	-0.93592400	-1.46797600	1.25064800
H	2.61985700	0.51988600	-0.53057000
Cl	2.25923100	-1.48862200	-1.71911300

(*R*)-**TS1**$_{O}$-Cl-P

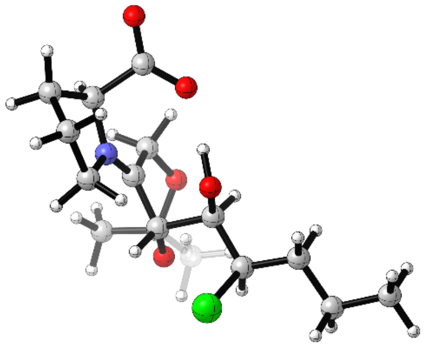

- Thermochemistry -

Zero-point correction=	0.419814 (Hartree/Particle)
Thermal correction to Energy=	0.439965
Thermal correction to Enthalpy=	0.440830
Thermal correction to Gibbs Free Energy=	0.372133
Sum of electronic and zero-point Energies=	-1515.609173
Sum of electronic and thermal Energies=	-1515.589022
Sum of electronic and thermal Enthalpies=	-1515.588157
Sum of electronic and thermal Free Energies=	-1515.656854

Number of Imaginary Frequencies = 0

E (Single Point Energy) [IEFPCM$_{(DCM)}$M06-2X/6-311++G(2d,2p)] = -1516.354000

C	0.17745800	0.70838300	-0.70155200
H	0.21378600	0.33326200	-1.72718100
C	-1.21963100	0.66624600	-0.16093100
N	-2.06779100	-0.20564800	-0.57214300
C	-3.31472400	-0.51731400	0.15266900
H	-3.81285300	0.40611100	0.45746600
C	-1.88023200	-1.13474900	-1.73033500
H	-0.88957000	-1.58395300	-1.68399500
H	-1.98663900	-0.53404800	-2.63873000
C	-3.02107300	-2.13786400	-1.56814200
H	-2.69703800	-2.97039100	-0.93630000

H	-3.33465400	-2.53810500	-2.53268300
C	-4.11407200	-1.33093500	-0.86239000
H	-4.62178700	-0.66546000	-1.56724600
H	-4.85620900	-1.94753900	-0.35627900
C	-1.52856800	1.57948300	0.99378100
H	-2.44667500	2.14765000	0.78943400
H	-1.68326100	0.97577100	1.89407100
O	0.77401600	1.98749300	-0.68753900
O	-0.43212900	2.42728100	1.24355600
C	0.13126500	3.00956800	0.08070300
C	1.22427500	3.94106900	0.55540800
H	1.78273500	4.31813000	-0.30334500
H	0.78642600	4.78161200	1.09773100
C	-0.90999400	3.71570000	-0.78273600
H	-0.39485300	4.26064400	-1.57584400
H	-1.48313900	4.42285800	-0.17763000
H	-1.60142700	3.01578000	-1.26082900
C	1.00379100	-0.25103300	0.22739500
H	1.01471200	0.21614000	1.22558300
C	2.46072300	-0.30972800	-0.22433200
O	0.46511000	-1.53761600	0.25915600
C	-2.98234000	-1.34780400	1.45375600
O	-1.76769700	-1.49011100	1.75716000
O	-3.98546300	-1.75685000	2.05103000
H	1.90129100	3.39436900	1.21541400
C	3.31403700	-1.15381400	0.71081100
H	3.13852000	-0.77238500	1.72646100
C	4.81006700	-1.10304000	0.40848100

H	4.99346800	-1.49533600	-0.59750700
H	5.14353500	-0.05755600	0.40643100
H	2.94925000	-2.18631200	0.69116500
C	5.61908100	-1.90254300	1.42806600
H	5.47315300	-1.50972700	2.43958400
H	5.31178700	-2.95331700	1.42996000
H	6.68815400	-1.86495700	1.20275900
H	-0.33352800	-1.55923300	0.84277300
H	2.84724700	0.70877600	-0.29934500
Cl	2.54829700	-0.97262200	-1.91927900

(*S*)-**TS1**$_{O}$-Cl-Pre

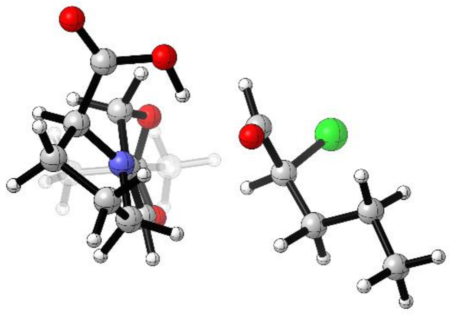

- Thermochemistry -

Zero-point correction= 0.416527 (Hartree/Particle)
Thermal correction to Energy= 0.438072
Thermal correction to Enthalpy= 0.438937
Thermal correction to Gibbs Free Energy= 0.366549
Sum of electronic and zero-point Energies= -1515.594023
Sum of electronic and thermal Energies= -1515.572478
Sum of electronic and thermal Enthalpies= -1515.571613
Sum of electronic and thermal Free Energies= -1515.644002

Number of Imaginary Frequencies = 0

E (Single Point Energy) [IEFPCM$_{(DCM)}$M06-2X/6-311++G(2d,2p)] = -1516.336956

C	0.30527400	0.75188600	-1.27007500
H	0.41255200	0.06420900	-2.09979600
C	-0.75495300	0.78731200	-0.44314000
N	-1.85059700	-0.07494400	-0.55132800
C	-3.18855300	0.40627200	-0.20324000
H	-3.29817000	1.49039700	-0.31485100
C	-1.90211900	-1.00947300	-1.67574300
H	-1.11348300	-1.76126000	-1.56744400
H	-1.75806900	-0.48074200	-2.63282700
C	-3.31336000	-1.57669500	-1.57946300
H	-3.35791800	-2.34449000	-0.79944800
H	-3.65223400	-2.01837700	-2.51800000
C	-4.12799100	-0.34278100	-1.17849500
H	-4.30085800	0.29088300	-2.05268500
H	-5.09452100	-0.57119800	-0.72545800
C	-0.72543700	1.72678700	0.73683700
H	-1.41597000	2.57365100	0.61128600
H	-1.01367600	1.19706600	1.65158600
O	1.40122300	1.56977600	-1.13035900
O	0.59734600	2.18727300	0.95719300
C	1.24058900	2.65448200	-0.21406500
C	2.63364300	3.07463600	0.20099800
H	3.22172500	3.32578400	-0.68422100
H	3.11795800	2.25285000	0.73389400
C	0.46228100	3.77917600	-0.88986100
H	0.23393700	4.55873500	-0.15844300
H	-0.46867400	3.41648400	-1.33178300

H	1.07298100	4.20740700	-1.68736600
C	0.57126600	-1.55619100	1.23279300
H	0.61284100	-0.69957700	1.93291300
C	1.92752900	-2.01373500	0.73219500
O	-0.47940000	-2.09130100	0.96117600
C	-3.56302300	0.07466200	1.24409800
O	-2.82524400	-0.84350900	1.86143500
O	-4.51006100	0.59462300	1.79329900
H	2.57770200	3.94721200	0.85531200
C	2.91522000	-0.86059500	0.60752400
H	2.57071100	-0.16637700	-0.16664800
C	4.35115000	-1.29881100	0.33193700
H	4.67683700	-1.99295600	1.11690100
H	4.38981400	-1.84893400	-0.61426700
H	2.88224000	-0.31265700	1.56031200
C	5.29543900	-0.09998200	0.26927800
H	5.29360500	0.45092400	1.21583200
H	6.32207800	-0.41436600	0.06458100
H	4.98984200	0.59253100	-0.52222800
H	-2.08201600	-1.14948700	1.29177600
H	2.29482300	-2.74214100	1.46731700
Cl	1.74161700	-2.92103300	-0.80696300

(*S*)-**TS1**$_O$-Cl

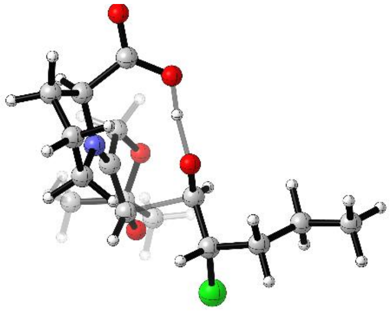

- Thermochemistry -

Zero-point correction=	0.415626 (Hartree/Particle)
Thermal correction to Energy=	0.435516
Thermal correction to Enthalpy=	0.436381
Thermal correction to Gibbs Free Energy=	0.369358
Sum of electronic and zero-point Energies=	-1515.582149
Sum of electronic and thermal Energies=	-1515.562258
Sum of electronic and thermal Enthalpies=	-1515.561393
Sum of electronic and thermal Free Energies=	-1515.628417

Number of Imaginary Frequencies = 1

E (Single Point Energy) [IEFPCM$_{(DCM)}$M06-2X/6-311++G(2d,2p)] = -1516.322975

C	0.45629400	0.48268000	-0.75121600
H	0.70898300	-0.25488700	-1.50756100
C	-0.86799000	0.77346700	-0.44311300
N	-1.85696000	-0.08693400	-0.68446200
C	-3.24092100	0.11393700	-0.24749000
H	-3.55766300	1.14805800	-0.41123500
C	-1.71268600	-1.25546600	-1.57411300
H	-0.84983000	-1.85186600	-1.27746700
H	-1.57814500	-0.89990100	-2.60432900
C	-3.03649500	-1.99124600	-1.38870400
H	-2.97535000	-2.64384100	-0.51133700
H	-3.29195800	-2.60124900	-2.25604900

C	-4.03287600	-0.85529800	-1.14299700
H	-4.27659500	-0.35356000	-2.08379000
H	-4.96110900	-1.17034700	-0.66548400
C	-1.16791200	2.00911700	0.37004200
H	-1.82288200	2.68200100	-0.20296900
H	-1.68495500	1.74126500	1.29733600
O	1.43979000	1.44522300	-0.59884800
O	0.02157100	2.65635000	0.76323700
C	0.99169600	2.75643800	-0.26447400
C	2.18044900	3.47176200	0.33807400
H	3.00870300	3.47098100	-0.37328900
H	2.48730900	2.95623100	1.25087300
C	0.44814900	3.45665600	-1.50650000
H	-0.01011500	4.40755200	-1.22255300
H	-0.29213100	2.84842900	-2.03232200
H	1.27354400	3.65132100	-2.19408600
C	0.63912700	-0.77835600	0.95025200
H	0.81568200	0.11102400	1.57824800
C	1.93576800	-1.52764400	0.63376100
O	-0.40246500	-1.48034200	1.11206400
C	-3.49952700	-0.17405300	1.24572200
O	-2.54724600	-0.68170900	1.98294200
O	-4.60192800	0.09378800	1.69033800
H	1.91142500	4.50285200	0.57647600
C	3.18120900	-0.70094500	0.35535900
H	3.12668700	-0.26554800	-0.64703100
C	4.47486100	-1.49812200	0.51478000
H	4.52114000	-1.91318100	1.52940000

H	4.46743400	-2.34983700	-0.17478300
H	3.17976500	0.14212200	1.05886600
C	5.70629600	-0.63370000	0.25268500
H	5.74512200	0.21264800	0.94636400
H	6.62793600	-1.20942300	0.37158000
H	5.68948200	-0.23118900	-0.76537100
H	-1.62689300	-0.96151300	1.53486300
H	2.10115400	-2.13997100	1.52921400
Cl	1.66185600	-2.74008600	-0.68250600

(*S*)-**TS1**$_{O}$-Cl-P

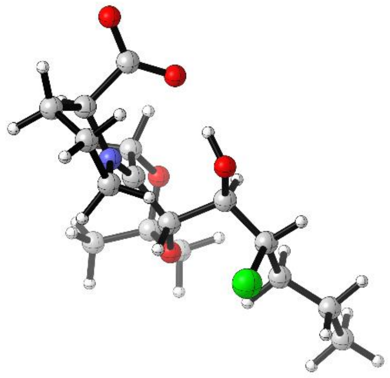

- Thermochemistry -

Zero-point correction= 0.419984 (Hartree/Particle)
Thermal correction to Energy= 0.440008
Thermal correction to Enthalpy= 0.440873
Thermal correction to Gibbs Free Energy= 0.372586
Sum of electronic and zero-point Energies= -1515.606404
Sum of electronic and thermal Energies= -1515.586381
Sum of electronic and thermal Enthalpies= -1515.585516
Sum of electronic and thermal Free Energies= -1515.653803

Number of Imaginary Frequencies = 0

E (Single Point Energy) [IEFPCM$_{(DCM)}$M06-2X/6-311++G(2d,2p)] = -1516.3513

C	0.36112900	0.18791000	-0.56946000
H	0.38034600	-0.43928800	-1.46473500
C	-1.03486900	0.66185200	-0.28396400
N	-2.06015900	-0.02631000	-0.63472900
C	-3.41690300	0.22560200	-0.11071300
H	-3.63553000	1.29594400	-0.13589600
C	-2.04048200	-1.25298800	-1.49131200
H	-1.24498500	-1.91827700	-1.15970200
H	-1.85888100	-0.91991100	-2.51767200
C	-3.44412500	-1.82942300	-1.31513600
H	-3.46319500	-2.50548400	-0.45490100
H	-3.75580200	-2.38619100	-2.19927700
C	-4.30765800	-0.59572800	-1.03983600
H	-4.50221200	-0.04429200	-1.96471000
H	-5.25591400	-0.82339400	-0.55405200
C	-1.17309000	1.90234600	0.55574200
H	-1.84955800	2.61623500	0.06522500
H	-1.59748500	1.62852100	1.52704700
O	1.29223700	1.23076800	-0.76129300
O	0.09598000	2.46234900	0.79795700
C	0.91907000	2.54963900	-0.35186100
C	2.19254100	3.24050700	0.08349200
H	2.92499900	3.20073700	-0.72521900
H	2.59661400	2.73145800	0.96116400
C	0.23015100	3.26274500	-1.51175800
H	-0.15533100	4.23148100	-1.18333400
H	-0.59199600	2.67633400	-1.93213000
H	0.96055500	3.42093600	-2.30730100

C	0.77482800	-0.68080200	0.67123900
H	0.90488300	0.01291000	1.51650000
C	2.11824600	-1.39244000	0.48206800
O	-0.18188400	-1.66488700	0.94703800
C	-3.51285600	-0.25813500	1.38915300
O	-2.44536100	-0.61986200	1.95196400
O	-4.65730400	-0.20336700	1.85565600
H	1.98463200	4.28357800	0.33091600
C	3.36064800	-0.51490800	0.41762000
H	3.37405500	0.05058700	-0.51836100
C	4.66054300	-1.29961300	0.58763700
H	4.62498700	-1.86079500	1.52980800
H	4.74986200	-2.03799000	-0.21685000
H	3.27447900	0.22222600	1.22777300
C	5.87923100	-0.37903200	0.57909600
H	5.82373000	0.35176500	1.39266300
H	6.80599800	-0.94629800	0.69879200
H	5.94233200	0.17402800	-0.36379000
H	-0.99020400	-1.27408600	1.36289800
H	2.19562100	-2.08805000	1.32210400
Cl	2.03243200	-2.47492500	-0.98096900

(*R*)-**TS1**$_T$-Cl-Pre

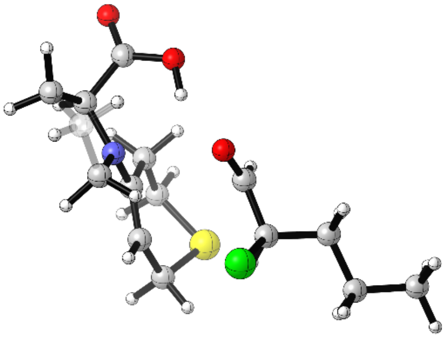

- Thermochemistry -

Zero-point correction=	0.381900 (Hartree/Particle)
Thermal correction to Energy=	0.401652
Thermal correction to Enthalpy=	0.402517
Thermal correction to Gibbs Free Energy=	0.333886
Sum of electronic and zero-point Energies=	-1724.102074
Sum of electronic and thermal Energies=	-1724.082322
Sum of electronic and thermal Enthalpies=	-1724.081457
Sum of electronic and thermal Free Energies=	-1724.150088

Number of Imaginary Frequencies = 0

E (Single Point Energy) [IEFPCM$_{(DCM)}$M06-2X/6-311++G(2d,2p)] = -1724.786027

C	-0.20294900	1.09364800	-1.57221400
H	-0.06839100	0.32878100	-2.33095200
C	-1.19593800	0.95538600	-0.66461600
N	-1.96408600	-0.20532500	-0.60168900
C	-3.30817900	-0.23253400	-0.03173700
H	-3.87817800	0.68038200	-0.22835700
C	-1.77532900	-1.27523800	-1.58296700
H	-0.74609100	-1.64332700	-1.53908900
H	-1.97522800	-0.91023200	-2.60317500
C	-2.80531800	-2.31437200	-1.15768000
H	-2.41856400	-2.90079800	-0.31697000

H	-3.06552100	-3.00128000	-1.96476500
C	-3.98466800	-1.44351100	-0.71841300
H	-4.53185300	-1.08426800	-1.59431000
H	-4.69301500	-1.94810300	-0.05838300
C	-1.48396900	1.96890600	0.42458200
H	-2.55829400	2.18492900	0.45026600
H	-1.23666800	1.52294200	1.39768500
C	0.99966400	-0.62628300	0.80313900
H	0.85699900	0.26044700	1.44827800
C	2.29405800	-0.59375800	0.02706500
O	0.20242300	-1.53902700	0.79827600
C	-3.31260200	-0.42269800	1.48683200
O	-2.23178700	-0.96815300	2.03858300
O	-4.27777700	-0.13262800	2.16012900
C	3.47011900	-0.79764600	0.98688600
H	3.33588100	-0.09234600	1.81849700
C	4.83235800	-0.55592800	0.33996200
H	4.97163300	-1.25847600	-0.48830300
H	4.84872800	0.45322000	-0.09006100
H	2.37318200	0.38964000	-0.44531300
H	3.41548600	-1.80926200	1.40670200
C	5.96865800	-0.71120300	1.34828500
H	5.86226300	0.00187700	2.17196700
H	5.97579700	-1.71928500	1.77483700
H	6.93915600	-0.53885900	0.87605600
H	-1.51131300	-1.11835300	1.38540100
C	-0.75633500	3.29918000	0.26959200
C	0.76855600	2.24050000	-1.65313300

H	-1.18428200	3.88327600	-0.55107800
H	-0.84812800	3.88640800	1.18488500
H	1.75092600	1.88012800	-1.97492900
H	0.45520300	2.98747600	-2.39243300
Cl	2.28299500	-1.80708000	-1.29243100
S	1.01727100	3.08340500	-0.06016900

(*R*)-**TS1**$_T$-Cl

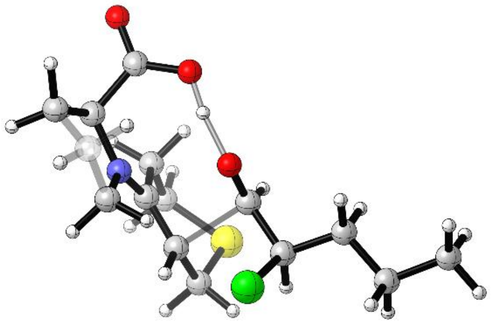

- Thermochemistry -

Zero-point correction=	0.381793 (Hartree/Particle)
Thermal correction to Energy=	0.399942
Thermal correction to Enthalpy=	0.400807
Thermal correction to Gibbs Free Energy=	0.336803
Sum of electronic and zero-point Energies=	-1724.092262
Sum of electronic and thermal Energies=	-1724.074114
Sum of electronic and thermal Enthalpies=	-1724.073249
Sum of electronic and thermal Free Energies=	-1724.137252

Number of Imaginary Frequencies = 1

E (Single Point Energy) [IEFPCM$_{(DCM)}$M06-2X/6-311++G(2d,2p)] = -1724.774436

C	-0.02605600	1.06081600	-1.19138300
H	0.24953700	0.38239100	-1.99360500
C	-1.27469000	0.87205200	-0.60035400
N	-1.89977000	-0.31032400	-0.70555500

C	-3.12941900	-0.67099900	0.00799800
H	-3.89544100	0.09937600	-0.10135500
C	-1.48539800	-1.37001600	-1.64950500
H	-0.40973700	-1.52715400	-1.60135500
H	-1.76853800	-1.06865200	-2.66685200
C	-2.27045100	-2.58628400	-1.17420500
H	-1.73254400	-3.06896500	-0.35134500
H	-2.41671100	-3.31858900	-1.96957600
C	-3.58079200	-1.97539800	-0.67783300
H	-4.22602200	-1.72400200	-1.52433800
H	-4.14264500	-2.61418000	0.00509600
C	-1.87046300	1.87653900	0.36301000
H	-2.96112500	1.85116200	0.28709300
H	-1.61797000	1.55972200	1.38496200
C	0.96120400	-0.05725600	0.40313900
H	0.88771200	0.81361400	1.07356400
C	2.35247700	-0.19454000	-0.19794800
O	0.30328200	-1.10398900	0.65232900
C	-2.95505300	-0.88087800	1.52232000
O	-1.75515600	-1.06271100	2.01598200
O	-3.95367300	-0.89808000	2.21970600
C	3.31253100	-0.66221700	0.89762500
H	3.18871400	0.02579600	1.74529600
C	4.77989600	-0.67723800	0.47476400
H	4.91235000	-1.37503600	-0.35875600
H	5.05853700	0.31743300	0.10477300
H	2.69610400	0.75594400	-0.60824700
H	2.99566900	-1.65425600	1.23992900

C	5.69426800	-1.07507000	1.63194700
H	5.59936200	-0.37260300	2.46622400
H	5.43972000	-2.07294200	2.00319900
H	6.74190400	-1.08909800	1.32023900
H	-0.92885200	-1.04576000	1.36829500
C	-1.41993100	3.31788700	0.15118100
C	0.62955800	2.41732300	-1.30687100
H	-1.81042000	3.71252700	-0.79182200
H	-1.79919700	3.94326800	0.96099300
H	1.70902100	2.31354800	-1.44113600
H	0.26079300	2.95112800	-2.19057700
Cl	2.35410800	-1.36379900	-1.57487000
S	0.38666400	3.48200600	0.14522100

(*R*)-**TS1**$_{T}$-Cl-P

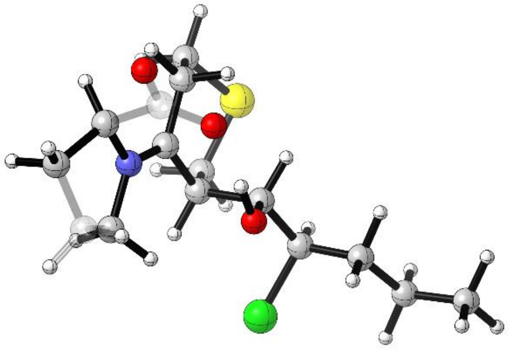

- Thermochemistry -

Zero-point correction= 0.386258 (Hartree/Particle)
Thermal correction to Energy= 0.404439
Thermal correction to Enthalpy= 0.405304
Thermal correction to Gibbs Free Energy= 0.341091
Sum of electronic and zero-point Energies= -1724.120199
Sum of electronic and thermal Energies= -1724.102017
Sum of electronic and thermal Enthalpies= -1724.101152
Sum of electronic and thermal Free Energies= -1724.165365

Number of Imaginary Frequencies = 0

E (Single Point Energy) [IEFPCM$_{(DCM)}$M06-2X/6-311++G(2d,2p)] = -1724.806482

C	0.15411500	0.79540200	-0.85218600
H	0.17852400	0.22399000	-1.78104100
C	-1.24512500	0.80996600	-0.29852400
N	-2.08816200	-0.09958600	-0.65744900
C	-3.35813000	-0.37710600	0.05345300
H	-3.93487200	0.53925100	0.17802400
C	-1.84143700	-1.14777100	-1.69879600
H	-0.82727500	-1.52922800	-1.60731500
H	-1.98544400	-0.67298100	-2.67411900
C	-2.90690200	-2.20183000	-1.41453000
H	-2.53792500	-2.90824600	-0.66456500
H	-3.16425400	-2.75734200	-2.31706700
C	-4.06772500	-1.38448600	-0.85023600
H	-4.59884900	-0.86309600	-1.65265800
H	-4.78487200	-1.97193700	-0.27689000
C	-1.57098000	1.78477600	0.80074600
H	-2.64003400	1.79653000	1.01140900
H	-1.07883700	1.40601600	1.70556700
C	1.00045800	0.00314200	0.20074600
H	1.00477000	0.57489200	1.14097300
C	2.47238500	-0.11842500	-0.18811000
O	0.47215600	-1.28053700	0.39556100
C	-3.04941200	-0.97202100	1.47612200
O	-1.84006100	-1.11139800	1.80162400
O	-4.06402500	-1.23885200	2.13630500

C	3.26277400	-0.94683600	0.81501100
H	3.03051700	-0.54115100	1.80960400
C	4.77362100	-0.91111900	0.59630900
H	5.01013100	-1.32235600	-0.39102900
H	5.11555600	0.13167600	0.59557500
H	2.91040500	0.87950600	-0.26949000
H	2.89080600	-1.97602200	0.79843800
C	5.51804000	-1.69821700	1.67312600
H	5.31769000	-1.28723800	2.66795200
H	5.20371100	-2.74686700	1.67504400
H	6.59838800	-1.67083900	1.50832600
H	-0.35199800	-1.23797400	0.93924700
C	-1.10565900	3.21840200	0.52173000
C	0.68371300	2.19674600	-1.18421300
H	-1.63811300	3.64156200	-0.33530200
H	-1.32775300	3.83571300	1.39344400
H	1.70691900	2.12788800	-1.55867500
H	0.08011400	2.63160300	-1.98668200
Cl	2.62825800	-0.83845100	-1.85385700
S	0.68102900	3.33687300	0.22732100

(*S*)-**TS1**$_T$-Cl-Pre

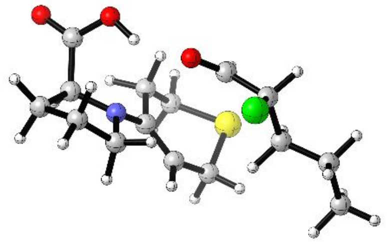

- Thermochemistry -

Zero-point correction=	0.381822 (Hartree/Particle)
Thermal correction to Energy=	0.401559
Thermal correction to Enthalpy=	0.402424
Thermal correction to Gibbs Free Energy=	0.333383
Sum of electronic and zero-point Energies=	-1724.101640
Sum of electronic and thermal Energies=	-1724.081903
Sum of electronic and thermal Enthalpies=	-1724.081038
Sum of electronic and thermal Free Energies=	-1724.150079

Number of Imaginary Frequencies = 0

E (Single Point Energy) [IEFPCM$_{(DCM)}$M06-2X/6-311++G(2d,2p)] = -1724.785259

C	0.24980000	1.21338500	-1.31143000
H	0.50799700	0.49287800	-2.08204500
C	-0.88987900	1.04237000	-0.60824000
N	-1.67130200	-0.10861600	-0.74460400
C	-3.12823300	-0.06689900	-0.61611200
H	-3.56213300	0.86899300	-0.98308700
C	-1.25425800	-1.17931500	-1.64930400
H	-0.28653100	-1.57980500	-1.32893300
H	-1.15691100	-0.81373800	-2.68413200
C	-2.39634600	-2.18088600	-1.53768600
H	-2.29378700	-2.76342100	-0.61563600
H	-2.43320600	-2.87202200	-2.38144700
C	-3.61827400	-1.26123200	-1.47105100

H	-3.86527900	-0.89570700	-2.47158900
H	-4.51025700	-1.72943500	-1.05034200
C	-1.36075600	1.99678000	0.46990400
H	-2.43225900	2.19567800	0.34670700
H	-1.24864500	1.50385600	1.44542900
C	0.89765900	-0.82355500	1.36770000
H	0.88124800	0.22958700	1.70604300
C	2.28483600	-1.33267100	1.04396700
O	-0.11534100	-1.48249400	1.30094700
C	-3.61087600	-0.22889400	0.82645600
O	-2.76219100	-0.76518700	1.69681400
O	-4.73726300	0.07589800	1.15592400
C	3.02880600	-0.36435500	0.12774700
H	2.54576800	-0.37079900	-0.85721500
C	4.52062000	-0.65592900	-0.00840200
H	4.97783100	-0.66442900	0.98868200
H	4.66136900	-1.65591300	-0.43223200
H	2.81575800	-1.41352600	2.00115800
H	2.88986100	0.64160400	0.54895500
C	5.20981800	0.38384000	-0.88955300
H	4.77510300	0.39170000	-1.89463700
H	5.10009100	1.38871900	-0.46813800
H	6.27797100	0.17438600	-0.98740200
H	-1.87410100	-0.91667900	1.30090600
C	-0.64233800	3.33998700	0.48883900
C	1.21998900	2.35531100	-1.16391100
H	-0.92807400	3.94817900	-0.37511100
H	-0.90143600	3.89379200	1.39286600

H	2.24593800	2.00138400	-1.30205200
H	1.04728800	3.12554000	-1.92521100
Cl	2.21768600	-2.98777300	0.35960900
S	1.16373700	3.14489200	0.47276900

(*S*)-**TS1**$_{T}$-Cl

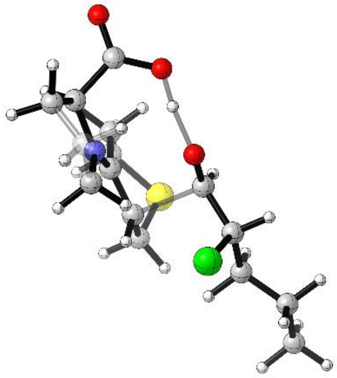

- Thermochemistry -

Zero-point correction= 0.381811 (Hartree/Particle)
Thermal correction to Energy= 0.399850
Thermal correction to Enthalpy= 0.400715
Thermal correction to Gibbs Free Energy= 0.337056
Sum of electronic and zero-point Energies= -1724.086298
Sum of electronic and thermal Energies= -1724.068259
Sum of electronic and thermal Enthalpies= -1724.067394
Sum of electronic and thermal Free Energies= -1724.131053

Number of Imaginary Frequencies = 1

E (Single Point Energy) [IEFPCM$_{(DCM)}$M06-2X/6-311++G(2d,2p)] = -1724.768234

C	0.41136000	0.64516000	-1.01382900
H	0.66493800	-0.20383800	-1.64541600
C	-0.94356800	0.82452100	-0.70570100
N	-1.80345700	-0.19643300	-0.78847900
C	-3.20841300	-0.14848800	-0.36046900

H	-3.71864000	0.72069100	-0.78048100
C	-1.47975900	-1.48278600	-1.43976800
H	-0.50575100	-1.84017400	-1.11411500
H	-1.47471600	-1.33627600	-2.52792500
C	-2.61320100	-2.39593100	-0.99129500
H	-2.38651900	-2.79616400	0.00267400
H	-2.76505200	-3.23131000	-1.67633500
C	-3.81012800	-1.44774000	-0.93142900
H	-4.18714600	-1.25000900	-1.93887100
H	-4.63789800	-1.80824500	-0.31922000
C	-1.45242500	2.08104400	-0.02739900
H	-2.49697500	2.25025900	-0.30142800
H	-1.43794300	1.89713200	1.05631800
C	0.79263000	-0.35315600	0.89562300
H	0.93174600	0.63052800	1.37046200
C	2.11579900	-1.09373300	0.69792400
O	-0.21227700	-1.06317500	1.18256900
C	-3.42118700	-0.06876700	1.16161600
O	-2.43138500	-0.35315800	1.96961400
O	-4.52767000	0.23684700	1.56957800
C	3.32690400	-0.28231400	0.26886800
H	3.30767800	-0.14347500	-0.81709600
C	4.65211300	-0.92931600	0.67064500
H	4.67761200	-1.04677700	1.76082300
H	4.70697700	-1.93640300	0.24162300
H	2.30581900	-1.52348800	1.68983900
H	3.25055500	0.71052800	0.73461400
C	5.85052000	-0.10351600	0.20908200

H	5.85637000	-0.00071300	-0.88091300
H	5.82205900	0.90229500	0.64079100
H	6.79206500	-0.57152300	0.50760800
H	-1.49751500	-0.60743900	1.55874600
C	-0.67782600	3.35892000	-0.32879800
C	1.31110200	1.84612000	-1.23610800
H	-0.81745100	3.66399200	-1.37045100
H	-1.04247900	4.16459100	0.31051300
H	2.36119200	1.56472400	-1.20720600
H	1.13179100	2.27576900	-2.22911000
Cl	1.90958300	-2.53683500	-0.37063700
S	1.09798100	3.17202000	-0.01457000

(*S*)-**TS1**$_T$-Cl-P

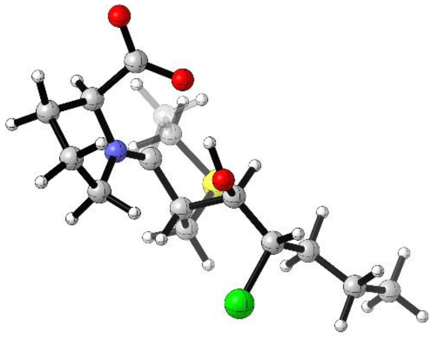

- Thermochemistry -

Zero-point correction=	0.386197 (Hartree/Particle)
Thermal correction to Energy=	0.404426
Thermal correction to Enthalpy=	0.405291
Thermal correction to Gibbs Free Energy=	0.341112
Sum of electronic and zero-point Energies=	-1724.116090
Sum of electronic and thermal Energies=	-1724.097861
Sum of electronic and thermal Enthalpies=	-1724.096996
Sum of electronic and thermal Free Energies=	-1724.161176

Number of Imaginary Frequencies = 0

E (Single Point Energy) [IEFPCM$_{(DCM)}$M06-2X/6-311++G(2d,2p)] = -1724.802266

C	0.28484600	0.12929700	-0.82136200
H	0.19732400	-0.71677700	-1.50420000
C	-1.08604900	0.66464500	-0.49631700
N	-2.12903600	-0.07324500	-0.67952800
C	-3.46235000	0.22368100	-0.10596000
H	-3.76360300	1.24333300	-0.34489000
C	-2.12963500	-1.43486500	-1.30415100
H	-1.27184900	-2.00287700	-0.95192700
H	-2.07618500	-1.29238300	-2.38761700
C	-3.46479000	-2.03418900	-0.87284300
H	-3.35435100	-2.52322100	0.09986300
H	-3.81762000	-2.77258100	-1.59374400
C	-4.37674400	-0.81412200	-0.75461900
H	-4.69414400	-0.47179000	-1.74449500
H	-5.26051700	-0.98082300	-0.13883200
C	-1.18630200	2.00040200	0.19048600
H	-2.22098900	2.33709900	0.24700900
H	-0.85184100	1.84458800	1.22372900
C	0.83410100	-0.43461400	0.53141400
H	0.92748200	0.39246600	1.24919300
C	2.22751800	-1.07711200	0.46575300
O	-0.04720700	-1.41809100	1.02082400
C	-3.40766500	0.07085200	1.45851100
O	-2.30195300	-0.24142200	1.97570800
O	-4.49151900	0.28784800	2.01933600
C	3.42301400	-0.13698300	0.57980300

H	3.54762700	0.46500500	-0.32590800
C	4.73240300	-0.86385200	0.88590800
H	4.60542600	-1.46236700	1.79640700
H	4.96016600	-1.56446200	0.07526800
H	2.24915000	-1.77765400	1.30409000
H	3.20083700	0.56359100	1.39561900
C	5.89210700	0.11358900	1.06503500
H	6.04700100	0.70538600	0.15701000
H	5.69553900	0.80813300	1.88815800
H	6.82344800	-0.41461200	1.28487600
H	-0.85837300	-1.00148400	1.40110500
C	-0.34409800	3.10252500	-0.46294700
C	1.16228900	1.17106200	-1.52521500
H	-0.71111400	3.32809000	-1.46835300
H	-0.42698700	4.00998900	0.13689100
H	2.13581300	0.73716500	-1.75461200
H	0.70302900	1.44417700	-2.48076600
Cl	2.38505800	-2.14293300	-1.00186000
S	1.41909800	2.68350100	-0.55646700

Supplementary Table 5. Energies for enamine addition to 2-fluoropentanal. Reported energies for structures optimized at the IEFPCM$_{(DCM)}$M06-2X/6-311++G(2d,2p)//IEFPCM$_{(DCM)}$M06-2X/6-31+G(d,p) level of theory represent the sum of the thermal correction to Gibbs Free Energy computed at the IEFPCM$_{(DCM)}$M06-2X/6-31+G(d,p) level of theory and single point energies computed at the IEFPCM$_{(DCM)}$M06-2X/6-311++G(2d,2p). All energies are reported in Hartrees.

**Structure**	**Single Point Energies, E IEFPCM$_{(DCM)}$M06-2X/6-311++G(2d,2p)**	**Thermal Corrections to Gibbs Free Energies, IEFPCM$_{(DCM)}$M06-2X/6-31+G(d,p)**	**Gibbs Free Energies (G), IEFPCM$_{(DCM)}$ M06-2X/6-31+G(d,p)**	**Gibbs Free Energies (G), IEFPCM$_{(DCM)}$M06-2X/6-311++G(2d,2p)// IEFPCM$_{(DCM)}$M06-2X/6-31+G(d,p)**
2-Fluoropentanal	-370.97333955	0.104800	-370.761392	-370.8685396
Enamine of Cyclohexanone (G)	-634.55147535	0.238392	-634.142903	-634.3130834
Enamine of Dioxane (O)	-784.98483791	0.244077	-784.522619	-784.7407609
Enamine of Tetrahydro-4H-thiopyranone (T)	-993.43210840	0.210205	-993.028735	-993.2219034
Enamine of Tetrahydro-4H-pyranone (P)	-670.45469021	0.214793	-670.055312	-670.2398972
(*R*)-**TS1**$_P$-F-Pre	-1041.445897	0.339385	-1040.814963	-1041.106512
(*R*)-**TS1**$_P$-F	-1041.438205	0.342953	-1040.805151	-1041.095252
(*R*)-**TS1**$_P$-F-P	-1041.468899	0.346922	-1040.832562	-1041.121977
(*S*)-**TS1**$_P$-F-Pre	-1041.445551	0.339716	-1040.814143	-1041.105835
(*S*)-**TS1**$_P$-F	-1041.432614	0.343526	-1040.79923	-1041.089088
(*S*)-**TS1**$_P$-F-P	-1041.465035	0.347826	-1040.827766	-1041.117209
(*R*)-**TS1**$_G$-F-Pre	-1005.542301	0.363097	-1004.902179	-1005.179204
(*R*)-**TS1**$_G$-F	-1005.535067	0.366742	-1004.892614	-1005.168325
(*R*)-**TS1**$_G$-F-P	-1005.56741	0.370131	-1004.922232	-1005.197279
(*S*)-**TS1**$_G$-F-Pre	-1005.542275	0.363781	-1004.901524	-1005.178494
(*S*)-**TS1**$_G$-F	-1005.52915	0.36724	-1004.886483	-1005.16191
(*S*)-**TS1**$_G$-F-P	-1005.562739	0.371678	-1004.915989	-1005.191061
(*R*)-**TS1**$_O$-F-Pre	-1155.977106	0.36792	-1155.284337	-1155.609186
(*R*)-**TS1**$_O$-F	-1155.966865	0.371031	-1155.272322	-1155.595834

(*R*)-**TS1**$_O$-F-P	-1155.993515	0.374432	-1155.296115	-1155.619083
(*S*)-**TS1**$_O$-F-Pre	-1155.976614	0.369748	-1155.281921	-1155.606866
(*S*)-**TS1**$_O$-F	-1155.962553	0.371627	-1155.267711	-1155.590926
(*S*)-**TS1**$_O$-F-P	-1155.990751	0.374972	-1155.293024	-1155.615779
(*R*)-**TS1**$_T$-F-Pre	-1364.424378	0.334681	-1363.789529	-1364.089697
(*R*)-**TS1**$_T$-F	-1364.414632	0.338653	-1363.777503	-1364.075979
(*R*)-**TS1**$_T$-F-P	-1364.445963	0.342962	-1363.804973	-1364.103001
(*S*)-**TS1**$_T$-F-Pre	-1364.424621	0.335908	-1363.78861	-1364.088713
(*S*)-**TS1**$_T$-F	-1364.408701	0.339218	-1363.771275	-1364.069483
(*S*)-**TS1**$_T$-F-P	-1364.440624	0.342683	-1363.799902	-1364.097941

Pre – Precomplex

P – Product

2-Fluoropentanal

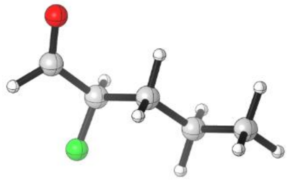

- Thermochemistry -

Zero-point correction= 0.134820 (Hartree/Particle)
Thermal correction to Energy= 0.142059
Thermal correction to Enthalpy= 0.142924
Thermal correction to Gibbs Free Energy= 0.104800
Sum of electronic and zero-point Energies= -370.731371
Sum of electronic and thermal Energies= -370.724132
Sum of electronic and thermal Enthalpies= -370.723267
Sum of electronic and thermal Free Energies= -370.761392

Number of Imaginary Frequencies = 0

E (Single Point Energy) [IEFPCM(DCM)M06-2X/6-311++G(2d,2p)] = -370.97333955

C	-2.05152900	-0.21022600	-0.16519200
H	-2.69577600	0.44411600	-0.78260500
C	-0.75208400	0.40682200	0.31057700
O	-2.36292400	-1.34621100	0.10454700
C	0.46296200	-0.35915200	-0.18435800
H	0.34683400	-1.39863500	0.14420100
C	1.77752800	0.22096200	0.33419300
H	1.85726800	1.26922600	0.02723400
H	1.76581600	0.21102400	1.43102300
H	0.45389800	-0.36131900	-1.28168900
C	2.98317100	-0.56298700	-0.17959400
H	3.02320900	-0.54321400	-1.27342200
H	3.91856400	-0.14272900	0.19888700
H	2.93120500	-1.61025400	0.13508900
H	-0.76438900	0.46487000	1.40609300
F	-0.71705800	1.71823300	-0.17054900

(*R*)-**TS1**$_{\mathrm{P}}$-F-Pre

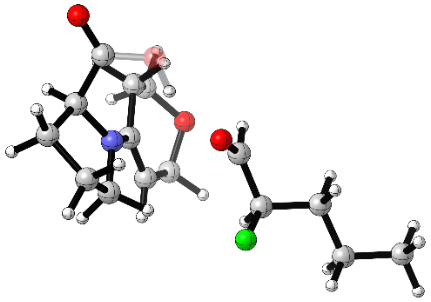

- Thermochemistry -

Zero-point correction=	0.386815 (Hartree/Particle)
Thermal correction to Energy=	0.405898
Thermal correction to Enthalpy=	0.406763
Thermal correction to Gibbs Free Energy=	0.339385
Sum of electronic and zero-point Energies=	-1040.767533
Sum of electronic and thermal Energies=	-1040.748450
Sum of electronic and thermal Enthalpies=	-1040.747585
Sum of electronic and thermal Free Energies=	-1040.814963

Number of Imaginary Frequencies = 0

E (Single Point Energy) [IEFPCM$_{(DCM)}$M06-2X/6-311++G(2d,2p)] = -1041.445897

C	-0.06323800	1.42534700	-1.36994400
H	0.26933300	0.79589400	-2.18977000
C	-1.09896000	1.06356600	-0.58269900
N	-1.69844400	-0.18522000	-0.65054400
C	-3.07666300	-0.41960300	-0.22931500
H	-3.73565000	0.42923800	-0.43687200
C	-1.26582900	-1.15912600	-1.65306400
H	-0.20642900	-1.39352200	-1.50910900
H	-1.40355100	-0.76129800	-2.67102000
C	-2.18981200	-2.34365000	-1.39529000
H	-1.82132800	-2.92426800	-0.54256700
H	-2.27024700	-3.00875200	-2.25668200
C	-3.51341800	-1.65455800	-1.05266500
H	-4.00231300	-1.30781300	-1.96729400
H	-4.21868300	-2.28339300	-0.50623700
C	-1.55934900	1.97486300	0.53229700
H	-2.65155500	2.05942600	0.54588500
H	-1.26028100	1.54616300	1.49878500
C	1.30808600	-0.22309700	0.75418300
H	1.23965000	0.77061900	1.23289700

C	2.51756700	-0.43441100	-0.12579500
O	0.50088000	-1.10102800	0.97962100
C	-3.21265800	-0.70936100	1.26812500
O	-2.11349000	-1.04861400	1.93328200
O	-4.29088100	-0.67052800	1.82103400
C	3.78228700	-0.53447500	0.71791900
H	3.84912600	0.36256400	1.34618600
C	5.04300700	-0.66761800	-0.13563900
H	4.94964400	-1.54785400	-0.78021200
H	5.12210100	0.20465100	-0.79590200
H	2.59372900	0.39471900	-0.84043200
H	3.68124800	-1.39623700	1.38949600
C	6.29900400	-0.78436700	0.72475100
H	6.42007700	0.09753600	1.36198200
H	6.24541300	-1.66350800	1.37470100
H	7.19432400	-0.87822800	0.10481100
H	-1.30635800	-0.99624800	1.37408900
C	-0.95760600	3.36552000	0.36982900
C	0.68358700	2.71687400	-1.15573600
H	-1.44652200	3.89722400	-0.46017600
H	-1.08275100	3.95289200	1.28099400
H	1.76588800	2.55419700	-1.20946000
H	0.42914500	3.45445700	-1.93500700
O	0.43491200	3.27834200	0.12156300
F	2.34529900	-1.60252300	-0.85567100

(*R*)-**TS1**$_{P}$-F

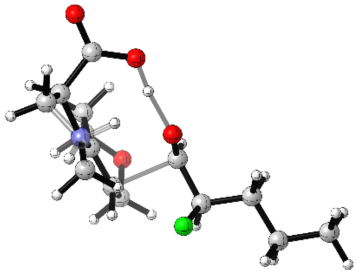

- Thermochemistry -

Zero-point correction=	0.386786 (Hartree/Particle)
Thermal correction to Energy=	0.404308
Thermal correction to Enthalpy=	0.405173
Thermal correction to Gibbs Free Energy=	0.342953
Sum of electronic and zero-point Energies=	-1040.761317
Sum of electronic and thermal Energies=	-1040.743796
Sum of electronic and thermal Enthalpies=	-1040.742931
Sum of electronic and thermal Free Energies=	-1040.805151

Number of Imaginary Frequencies = 1

E (Single Point Energy) [IEFPCM$_{(DCM)}$M06-2X/6-311++G(2d,2p)] = -1041.438205

C	0.09190600	1.38198100	-1.05563300
H	0.50552200	0.84771500	-1.90550800
C	-1.14918500	1.01594700	-0.55895100
N	-1.66374300	-0.20341100	-0.77534100
C	-2.91052000	-0.69597500	-0.18662500
H	-3.72626600	0.01819000	-0.32284100
C	-1.09034300	-1.15829900	-1.74148400
H	-0.00895800	-1.21470300	-1.61986600
H	-1.32776500	-0.82469800	-2.76069400
C	-1.79598000	-2.46315000	-1.39117800

H	-1.28088100	-2.94190100	-0.55162100
H	-1.81732400	-3.16102200	-2.22954600
C	-3.19045400	-1.99461100	-0.97090600
H	-3.79000600	-1.75201200	-1.85284400
H	-3.74266000	-2.71744200	-0.36860400
C	-1.84970400	1.91935300	0.42707200
H	-2.93197800	1.89376800	0.27113000
H	-1.65921400	1.55188700	1.44473100
C	1.12656100	0.18746300	0.49311700
H	1.06056600	1.05070000	1.17459500
C	2.45015700	0.05590300	-0.24130000
O	0.48957000	-0.87004200	0.73033200
C	-2.84836600	-0.97511900	1.32490400
O	-1.68389100	-1.07682300	1.92071800
O	-3.89562100	-1.12238300	1.92829200
C	3.50566300	-0.53500500	0.68106500
H	3.57585800	0.10220500	1.57214600
C	4.87601200	-0.64936100	0.01453600
H	4.78744300	-1.26337700	-0.88804200
H	5.20387300	0.34632800	-0.30918700
H	2.78417900	1.01673300	-0.64508200
H	3.15206900	-1.51859300	1.01298100
C	5.91552400	-1.25630700	0.95453500
H	6.03280200	-0.64470700	1.85504200
H	5.61583400	-2.26111600	1.26906900
H	6.89205000	-1.33260500	0.46904300
H	-0.82361000	-0.94189800	1.35545900
C	-1.35142000	3.35417200	0.29806700

C	0.59866300	2.79075800	-0.84835500
H	-1.72147800	3.80452300	-0.63484700
H	-1.69879700	3.95796700	1.13761200
H	1.68550900	2.80139900	-0.71881800
H	0.37041500	3.41870900	-1.72449500
O	0.06307400	3.39023300	0.31660600
F	2.27254300	-0.80517200	-1.32924300

(*R*)-**TS1**$_{P}$-F-P

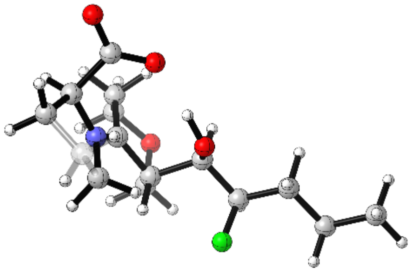

- Thermochemistry -

Zero-point correction=	0.391208 (Hartree/Particle)
Thermal correction to Energy=	0.408695
Thermal correction to Enthalpy=	0.409560
Thermal correction to Gibbs Free Energy=	0.346922
Sum of electronic and zero-point Energies=	-1040.788276
Sum of electronic and thermal Energies=	-1040.770789
Sum of electronic and thermal Enthalpies=	-1040.769924
Sum of electronic and thermal Free Energies=	-1040.832562

Number of Imaginary Frequencies = 0

E (Single Point Energy) [IEFPCM$_{(DCM)}$M06-2X/6-311++G(2d,2p)] = -1041.468899

F	2.60949200	-0.29652700	-1.61182700
C	0.30195200	1.04818100	-0.79056300
H	0.40439300	0.59509700	-1.77824900
C	-1.10435200	0.93553600	-0.28293300

N	-1.89584700	0.00918100	-0.70420700
C	-3.16411300	-0.35506500	-0.03391500
H	-3.78413600	0.52969500	0.11325200
C	-1.57584200	-0.96898200	-1.78778800
H	-0.54190400	-1.29340800	-1.69548100
H	-1.73260000	-0.45818400	-2.74276200
C	-2.58750300	-2.09024900	-1.56878800
H	-2.19409700	-2.80651700	-0.84088700
H	-2.79991700	-2.62062000	-2.49772100
C	-3.80158300	-1.35968600	-0.99405100
H	-4.34494100	-0.83366700	-1.78522600
H	-4.49637600	-2.00904200	-0.46154500
C	-1.47813200	1.85617600	0.84020500
H	-2.55674300	1.90904500	0.98937900
H	-1.04652600	1.42594000	1.75302500
C	1.18076300	0.22564500	0.21457100
H	1.20189000	0.75962300	1.17650600
C	2.62259900	0.14342600	-0.27455800
O	0.68670900	-1.07947700	0.36500000
C	-2.86342900	-0.98991600	1.37267300
O	-1.65779600	-1.09054200	1.72436900
O	-3.88131600	-1.32516400	1.99626900
C	3.48535800	-0.79907100	0.53942400
H	3.39992600	-0.50750700	1.59441400
C	4.95151400	-0.77727400	0.10934000
H	5.02009600	-1.03591300	-0.95308900
H	5.34601600	0.24144100	0.21302700
H	3.06661300	1.14523800	-0.28860700

H	3.07088300	-1.80857400	0.45256800
C	5.79995600	-1.74466900	0.93202700
H	5.76010000	-1.49070600	1.99639900
H	5.43716000	-2.77151900	0.82017700
H	6.84717000	-1.72128700	0.61881200
H	-0.14857100	-1.08490700	0.89270500
C	-0.90631100	3.26188800	0.61711000
C	0.70997200	2.52680600	-0.88567800
H	-1.43845300	3.76566700	-0.20239900
H	-1.02423800	3.85318500	1.52537300
H	1.77296100	2.61862800	-1.11127800
H	0.14951100	3.00707100	-1.70052500
O	0.47642700	3.21008500	0.32755800

(*S*)-**TS1**$_{P}$-F-Pre

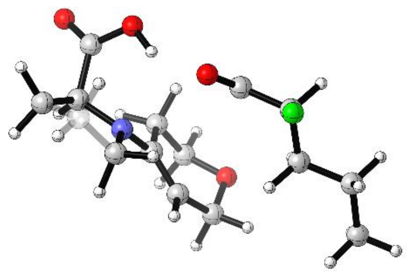

- Thermochemistry -

Zero-point correction= 0.386939 (Hartree/Particle)
Thermal correction to Energy= 0.405957
Thermal correction to Enthalpy= 0.406822
Thermal correction to Gibbs Free Energy= 0.339716
Sum of electronic and zero-point Energies= -1040.766920
Sum of electronic and thermal Energies= -1040.747902
Sum of electronic and thermal Enthalpies= -1040.747037
Sum of electronic and thermal Free Energies= -1040.814143

Number of Imaginary Frequencies = 0

E (Single Point Energy) [IEFPCM$_{(DCM)}$M06-2X/6-311++G(2d,2p)] = -1041.445551

C	0.44643300	1.33270600	-1.24283000
H	0.63331900	0.75145800	-2.14010300
C	-0.65964100	1.13660500	-0.49758600
N	-1.56702100	0.10687700	-0.73798700
C	-2.98134700	0.23865200	-0.39633800
H	-3.36009100	1.25801600	-0.52069100
C	-1.37430600	-0.78442800	-1.87987300
H	-0.42367200	-1.31734400	-1.77751700
H	-1.35819500	-0.21932700	-2.82580500
C	-2.59256200	-1.69662600	-1.80503700
H	-2.43226500	-2.47044000	-1.04638300
H	-2.81074700	-2.18663600	-2.75541100
C	-3.69724900	-0.72726800	-1.37344600
H	-4.04777400	-0.15207800	-2.23461400
H	-4.56187200	-1.21245700	-0.91585300
C	-0.91685100	1.98498400	0.72769500
H	-1.94162500	2.37338700	0.72540500
H	-0.81901700	1.36812000	1.63154000
C	0.88037000	-1.30277300	1.10747800
H	0.68142200	-0.35832000	1.64769700
C	2.33936500	-1.63672900	0.93416500
O	-0.02277000	-2.00233900	0.70224300
C	-3.27750000	-0.15813900	1.04992700
O	-2.42958600	-1.00342400	1.62998300
O	-4.26364400	0.23734100	1.63321900
C	3.06812200	-0.53515700	0.18054700

H	2.64454500	-0.47832900	-0.83071600
C	4.57787500	-0.75563100	0.11664600
H	4.97706800	-0.80800400	1.13681900
H	4.78330300	-1.72182800	-0.35634000
H	2.77460000	-1.77276100	1.93471700
H	2.84496300	0.41917700	0.67652400
C	5.27260400	0.36245900	-0.65725400
H	5.09513900	1.33368500	-0.18321000
H	6.35280100	0.20195900	-0.70230200
H	4.89713700	0.41639300	-1.68461700
H	-1.66427200	-1.22047400	1.04898900
C	0.04889700	3.16545400	0.78328800
C	1.47769700	2.36549100	-0.87080000
H	-0.26089600	3.93893200	0.06452900
H	0.06550800	3.60655100	1.78137300
H	2.48958600	1.97000100	-1.00298200
H	1.39112100	3.25883300	-1.51184400
O	1.37314100	2.75581800	0.48804100
F	2.45449900	-2.84038100	0.25770200

(*S*)-**TS1**$_{\mathrm{P}}$-F

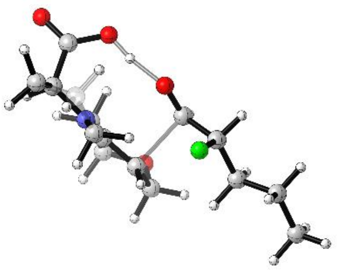

- Thermochemistry -

Zero-point correction=	0.386991 (Hartree/Particle)
Thermal correction to Energy=	0.404417
Thermal correction to Enthalpy=	0.405282
Thermal correction to Gibbs Free Energy=	0.343526
Sum of electronic and zero-point Energies=	-1040.755765
Sum of electronic and thermal Energies=	-1040.738339
Sum of electronic and thermal Enthalpies=	-1040.737474
Sum of electronic and thermal Free Energies=	-1040.799230

Number of Imaginary Frequencies = 1

E (Single Point Energy) [IEFPCM$_{(DCM)}$M06-2X/6-311++G(2d,2p)] = -1041.432614

C	0.60798700	0.86316300	-0.86105400
H	1.02766800	0.08599800	-1.49431000
C	-0.76664600	0.89775400	-0.66077400
N	-1.53301400	-0.19137300	-0.80649700
C	-2.96831100	-0.24347600	-0.51525200
H	-3.49964200	0.58607200	-0.98771600
C	-1.04966600	-1.44065300	-1.42134500
H	-0.08004600	-1.71389900	-1.00837100
H	-0.95844100	-1.29387500	-2.50609800
C	-2.14601600	-2.44069600	-1.07399300
H	-1.98179800	-2.82497700	-0.06168200
H	-2.17202600	-3.28388900	-1.76586300
C	-3.41404900	-1.58703800	-1.12810000
H	-3.71260100	-1.41936900	-2.16678900
H	-4.26330800	-2.01126700	-0.59077300
C	-1.40043700	2.12635900	-0.05215600
H	-2.40132100	2.28918300	-0.46124000
H	-1.51266900	1.96619500	1.02874400
C	0.95664600	-0.20562100	1.05887100

H	1.09573600	0.77961400	1.53063800
C	2.23989200	-0.98016000	0.75866900
O	-0.04878000	-0.91238900	1.32455600
C	-3.33402200	-0.17437100	0.97737500
O	-2.40622700	-0.36227900	1.88571800
O	-4.49569400	0.03307200	1.27648100
C	3.46904700	-0.23360900	0.28790700
H	3.31183600	0.11115400	-0.74044500
C	4.72628500	-1.10330300	0.34339100
H	4.89063800	-1.43192600	1.37683100
H	4.56437800	-2.00717200	-0.25418200
H	2.46579500	-1.49784300	1.70322900
H	3.60720700	0.65185200	0.92155200
C	5.95794300	-0.35814100	-0.16527900
H	6.14572900	0.53972600	0.43258900
H	6.85023400	-0.98767300	-0.11688500
H	5.82265100	-0.04545600	-1.20578000
H	-1.43586200	-0.53419800	1.56790400
C	-0.54498100	3.36239200	-0.29650100
C	1.38838100	2.16214500	-0.79672200
H	-0.58984200	3.65706000	-1.35552900
H	-0.89486300	4.19750300	0.31215100
H	2.39650800	2.00270400	-0.41382000
H	1.48443200	2.59943400	-1.80422300
O	0.79615900	3.10945700	0.07180400
F	1.94163400	-1.98741000	-0.16590700

(*S*)-**TS1**$_P$-F-P

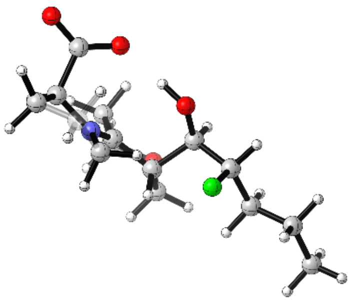

- Thermochemistry -

Zero-point correction=	0.391238 (Hartree/Particle)
Thermal correction to Energy=	0.408554
Thermal correction to Enthalpy=	0.409419
Thermal correction to Gibbs Free Energy=	0.347826
Sum of electronic and zero-point Energies=	-1040.784353
Sum of electronic and thermal Energies=	-1040.767037
Sum of electronic and thermal Enthalpies=	-1040.766172
Sum of electronic and thermal Free Energies=	-1040.827766

Number of Imaginary Frequencies = 0

E (Single Point Energy) [IEFPCM$_{(DCM)}$M06-2X/6-311++G(2d,2p)] = -1041.465035

C	0.62691600	0.59738700	-0.56646400
H	0.86933400	-0.13496400	-1.34182600
C	-0.86097900	0.79507800	-0.50110600
N	-1.65987800	-0.17510300	-0.78884200
C	-3.11661700	-0.15880100	-0.51871900
H	-3.56680400	0.73142900	-0.96010700
C	-1.22657200	-1.52340100	-1.26353100
H	-0.32564600	-1.82790000	-0.73385900
H	-1.02904300	-1.44210300	-2.33733900

C	-2.43798400	-2.40394600	-0.97609400
H	-2.40054300	-2.75660000	0.05937000
H	-2.46642300	-3.27031800	-1.63791200
C	-3.61526700	-1.44847300	-1.17357800
H	-3.80100800	-1.28439600	-2.23959700
H	-4.53966300	-1.78179500	-0.70220200
C	-1.36405600	2.09241000	0.05623800
H	-2.41167800	2.26649800	-0.19111600
H	-1.30275000	1.99775000	1.14860200
C	1.09043100	0.02660600	0.82586100
H	1.31637900	0.88355100	1.47856000
C	2.35057000	-0.83062400	0.69091000
O	0.15437200	-0.83802100	1.41823000
C	-3.39918400	-0.13400900	1.02645600
O	-2.41057500	-0.12086300	1.80600700
O	-4.60685300	-0.12205300	1.30907200
C	3.62621500	-0.17591600	0.20010500
H	3.51367000	0.08662000	-0.85926400
C	4.84027000	-1.09227900	0.36222000
H	4.96765300	-1.33195400	1.42476800
H	4.64735500	-2.03854200	-0.15503400
H	2.51108800	-1.29724900	1.66945800
H	3.78871300	0.75445700	0.75925100
C	6.11551100	-0.45325300	-0.18199400
H	6.33199400	0.48793700	0.33392400
H	6.97650600	-1.11405400	-0.05219200
H	6.01695300	-0.23462600	-1.25028600
H	-0.73704200	-0.44359800	1.57762000

C	-0.49764600	3.27070100	-0.38879100
C	1.32449600	1.92623200	-0.91898000
H	-0.64019200	3.48011000	-1.45821500
H	-0.76648600	4.16080900	0.18034400
H	2.39665500	1.85389700	-0.74676300
H	1.16279900	2.14835600	-1.98348500
O	0.86413700	3.00008100	-0.12902200
F	2.04022900	-1.88300300	-0.19225600

(*R*)-**TS1**$_G$-F-Pre

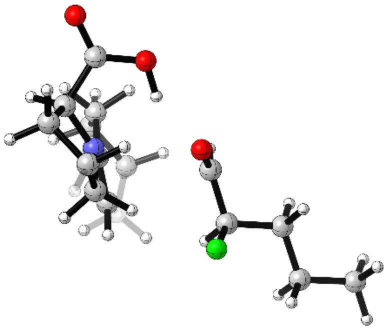

- Thermochemistry -

Zero-point correction= 0.410530 (Hartree/Particle)
Thermal correction to Energy= 0.429844
Thermal correction to Enthalpy= 0.430709
Thermal correction to Gibbs Free Energy= 0.363097
Sum of electronic and zero-point Energies= -1004.854747
Sum of electronic and thermal Energies= -1004.835433
Sum of electronic and thermal Enthalpies= -1004.834568
Sum of electronic and thermal Free Energies= -1004.902179

Number of Imaginary Frequencies = 0

E (Single Point Energy) [IEFPCM$_{(DCM)}$M06-2X/6-311++G(2d,2p)] = -1005.542301

C	-0.04818100	1.43036000	-1.33126600
H	0.30109000	0.76327400	-2.11484000

C	-1.09587900	1.06123600	-0.56021800
N	-1.66531800	-0.20923700	-0.63986100
C	-3.04739500	-0.47164200	-0.24817700
H	-3.72434400	0.35743800	-0.47614300
C	-1.20901700	-1.16144600	-1.65296800
H	-0.14484800	-1.37301700	-1.51609700
H	-1.35902900	-0.75629100	-2.66692200
C	-2.09915200	-2.37320400	-1.40644300
H	-1.71682100	-2.94791000	-0.55569000
H	-2.15700500	-3.03554600	-2.27198400
C	-3.44320000	-1.72470900	-1.06615200
H	-3.94320700	-1.39795600	-1.98210400
H	-4.12872000	-2.37408700	-0.51811300
C	-1.63805200	1.96495100	0.52815600
H	-2.73259300	1.94176500	0.53252200
H	-1.32183400	1.56796100	1.50487300
C	0.33921500	3.46086700	0.11771000
C	1.29625300	-0.32312500	0.81234400
H	1.20443200	0.59831600	1.41518100
C	2.51100300	-0.38824500	-0.08247900
O	0.51040000	-1.23972700	0.92925100
C	-3.20032600	-0.74969900	1.24896400
O	-2.10702800	-1.10190900	1.91857400
O	-4.27968900	-0.69464600	1.79872000
C	3.77476000	-0.58696000	0.74523000
H	3.82696900	0.21454900	1.49297800
C	5.03948000	-0.58127100	-0.11281400
H	4.95990500	-1.36279000	-0.87561100

H	5.10810400	0.37631900	-0.64323400
H	2.57272000	0.53551700	-0.67139700
H	3.68497000	-1.53533700	1.28960400
C	6.29488100	-0.80174600	0.72789500
H	6.25241700	-1.76520200	1.24572500
H	7.19294700	-0.79382200	0.10487500
H	6.40176100	-0.01811500	1.48490400
H	-1.30327200	-1.06534500	1.35288500
C	-1.16419000	3.41063600	0.37461900
C	0.66709000	2.75273200	-1.19676100
H	-1.68882000	3.87622400	-0.46948800
H	-1.42964100	3.97690900	1.27223000
H	1.75110000	2.59914600	-1.28127300
H	0.39833700	3.40543700	-2.03988700
H	0.86173300	2.96574200	0.94781800
H	0.69512000	4.49534200	0.08922200
F	2.36028300	-1.44511300	-0.96991100

(*R*)-**TS1**$_{G}$-F

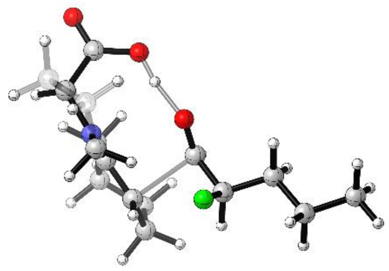

- Thermochemistry -

Zero-point correction= 0.410618 (Hartree/Particle)
Thermal correction to Energy= 0.428352
Thermal correction to Enthalpy= 0.429217

Thermal correction to Gibbs Free Energy= 0.366742
Sum of electronic and zero-point Energies= -1004.848738
Sum of electronic and thermal Energies= -1004.831004
Sum of electronic and thermal Enthalpies= -1004.830139
Sum of electronic and thermal Free Energies= -1004.892614

Number of Imaginary Frequencies = 1

E (Single Point Energy) [IEFPCM$_{(DCM)}$M06-2X/6-311++G(2d,2p)] = -1005.535067

C	0.08143300	1.38599300	-1.04452200
H	0.50130000	0.81239300	-1.86569100
C	-1.15653500	1.00488600	-0.55116400
N	-1.64302100	-0.23293000	-0.76871700
C	-2.88984500	-0.74773800	-0.20105100
H	-3.72087500	-0.05620900	-0.35892000
C	-1.04508600	-1.17691900	-1.73019900
H	0.03595900	-1.21604500	-1.60474700
H	-1.28241500	-0.84964500	-2.75203600
C	-1.72342800	-2.49676000	-1.38404500
H	-1.20423200	-2.96183200	-0.53926700
H	-1.72160700	-3.19674900	-2.22111200
C	-3.13112700	-2.05963000	-0.97670800
H	-3.73001100	-1.83833600	-1.86473400
H	-3.67052600	-2.79176700	-0.37370100
C	-1.91596500	1.89572800	0.40678200
H	-2.99147700	1.76036200	0.26768100
H	-1.69253300	1.56847500	1.43357800
C	-0.04185800	3.55711300	0.27036400
C	1.13445500	0.16355700	0.51415300
H	1.07234300	1.01805500	1.20602100
C	2.45441600	0.05088300	-0.22861200

O	0.49879000	-0.89366400	0.74345500
C	-2.84984400	-1.00931100	1.31329000
O	-1.69133300	-1.10074400	1.92520000
O	-3.90251800	-1.15520300	1.90752900
C	3.52679900	-0.51583600	0.69003900
H	3.59324600	0.12820600	1.57652300
C	4.89377500	-0.61088700	0.01351900
H	4.80881000	-1.23282100	-0.88392800
H	5.20108900	0.38808000	-0.32014200
H	2.76590400	1.01615700	-0.63923200
H	3.19349800	-1.50346900	1.03115200
C	5.95161000	-1.19247900	0.94906600
H	5.67239300	-2.20012400	1.27323100
H	6.92530100	-1.25510800	0.45596800
H	6.06559200	-0.57243100	1.84422600
H	-0.83365400	-0.96611400	1.36434600
C	-1.55454000	3.37218600	0.24496200
C	0.59934200	2.80011100	-0.89331200
H	-1.95243200	3.74139900	-0.70853800
H	-2.03538000	3.94762700	1.04099800
H	1.68947300	2.78786200	-0.76924700
H	0.41801800	3.35139200	-1.82627200
H	0.34904200	3.18670200	1.22715200
H	0.22096800	4.61751000	0.21111100
F	2.28691300	-0.82017500	-1.30980600

(*R*)-**TS1**$_{G}$-F-P

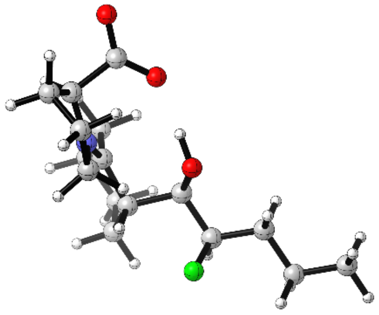

- Thermochemistry -

Zero-point correction=	0.414958 (Hartree/Particle)
Thermal correction to Energy=	0.432734
Thermal correction to Enthalpy=	0.433599
Thermal correction to Gibbs Free Energy=	0.370131
Sum of electronic and zero-point Energies=	-1004.877404
Sum of electronic and thermal Energies=	-1004.859628
Sum of electronic and thermal Enthalpies=	-1004.858763
Sum of electronic and thermal Free Energies=	-1004.922232

Number of Imaginary Frequencies = 0

E (Single Point Energy) [IEFPCM$_{(DCM)}$M06-2X/6-311++G(2d,2p)] = -1005.567410

C	0.30575600	1.02837100	-0.78242900
H	0.37707500	0.53752900	-1.75362200
C	-1.09470400	0.93339000	-0.24683900
N	-1.90900600	0.03191200	-0.68394500
C	-3.17402500	-0.33387100	-0.00806000
H	-3.77195900	0.55567800	0.19010700
C	-1.62307300	-0.92122700	-1.80057400
H	-0.60159800	-1.28518300	-1.71993000

H	-1.76016500	-0.37426000	-2.73831400
C	-2.66927600	-2.01787400	-1.62085800
H	-2.29288800	-2.77935200	-0.93086200
H	-2.90725300	-2.49865100	-2.57048600
C	-3.85340400	-1.27712400	-0.99997400
H	-4.38958900	-0.70143800	-1.76099400
H	-4.56142100	-1.92723200	-0.48600800
C	-1.44386300	1.82383400	0.91067800
H	-2.50824700	1.79100600	1.13814300
H	-0.92891900	1.39874300	1.78334100
C	0.49972000	3.33297500	0.31274600
C	1.17857300	0.18283900	0.20728300
H	1.18850000	0.66783100	1.19537300
C	2.62940700	0.13415100	-0.25790800
O	0.68954300	-1.13084800	0.30437500
C	-2.86081000	-1.03841000	1.36066800
O	-1.65103200	-1.17376900	1.68603800
O	-3.87269700	-1.38839300	1.98736200
C	3.48376200	-0.84721300	0.51824200
H	3.37350900	-0.61948900	1.58663900
C	4.95840500	-0.78924200	0.12201200
H	5.05133800	-0.98123600	-0.95262400
H	5.34262000	0.22380400	0.29646200
H	3.06320400	1.13957500	-0.20611100
H	3.08039800	-1.85324500	0.36398100
C	5.79724500	-1.79990900	0.90140100
H	5.44509200	-2.82023000	0.71841700
H	6.85066700	-1.75020900	0.61297600

H	5.73281100	-1.61292100	1.97839200
H	-0.14609300	-1.15481000	0.83028600
C	-0.97824700	3.27173900	0.68893000
C	0.74897900	2.49465000	-0.93978000
H	-1.57807600	3.72769500	-0.10787800
H	-1.17595000	3.83711000	1.60316000
H	1.79946200	2.52176600	-1.24020600
H	0.18255000	2.92797200	-1.77296700
H	1.11168400	2.97338300	1.14941400
H	0.79700200	4.36888700	0.12616800
F	2.64845600	-0.22827900	-1.61785300

(*S*)-**TS1**$_G$-F-Pre

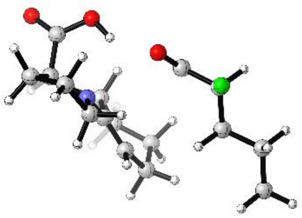

- Thermochemistry -

Zero-point correction= 0.410599 (Hartree/Particle)
Thermal correction to Energy= 0.429803
Thermal correction to Enthalpy= 0.430668
Thermal correction to Gibbs Free Energy= 0.363781
Sum of electronic and zero-point Energies= -1004.854705
Sum of electronic and thermal Energies= -1004.835502
Sum of electronic and thermal Enthalpies= -1004.834636
Sum of electronic and thermal Free Energies= -1004.901524

Number of Imaginary Frequencies = 0

E (Single Point Energy) [IEFPCM$_{(DCM)}$M06-2X/6-311++G(2d,2p)] = -1005.542275

C	0.46370500	1.31742000	-1.19485900
H	0.72077700	0.65295800	-2.01488500
C	-0.68050700	1.11461900	-0.50807600
N	-1.49894900	-0.00056900	-0.72666200
C	-2.94811000	0.07546100	-0.55070800
H	-3.36347300	1.05187300	-0.82037000
C	-1.14737400	-0.97412200	-1.75819300
H	-0.18049100	-1.43125800	-1.52549400
H	-1.07727600	-0.49719500	-2.74955800
C	-2.31044900	-1.95645400	-1.70703900
H	-2.18444400	-2.63824300	-0.85891500
H	-2.40073500	-2.55099800	-2.61786600
C	-3.50579400	-1.02494100	-1.48797300
H	-3.79636400	-0.56091800	-2.43441700
H	-4.38545600	-1.51774400	-1.06869300
C	-1.10682800	2.02727600	0.62360600
H	-2.17514600	2.25673800	0.54524200
H	-0.98450200	1.48865100	1.57571200
C	0.96855700	-1.11183200	1.31927200
H	0.84330200	-0.12435900	1.80115800
C	2.39749900	-1.51948000	1.05630400
O	0.01532200	-1.80871600	1.04840500
C	-3.38118400	-0.19549900	0.89014000
O	-2.53869900	-0.88597200	1.65271200
O	-4.46221400	0.15714900	1.31160400
C	3.13201000	-0.49235300	0.20949500
H	2.63864000	-0.43411400	-0.76880900
C	4.61655800	-0.81004800	0.04344300

H	5.08670600	-0.86364600	1.03298700
H	4.72553100	-1.79866000	-0.41484700
H	2.89400700	-1.63842500	2.03046800
H	3.01143400	0.48882800	0.68784400
C	5.31994900	0.24157600	-0.81152300
H	5.24003100	1.23351200	-0.35431500
H	6.38137000	0.01002200	-0.93131500
H	4.87166100	0.29540400	-1.80907400
H	-1.69126600	-1.07085600	1.18621400
C	-0.31195500	3.33377700	0.65552900
C	1.42248400	2.44656200	-0.90777600
H	-0.66080400	3.99028300	-0.15181200
H	-0.50448000	3.85287500	1.59909800
H	2.45515500	2.08422300	-0.98717100
H	1.32150100	3.22439100	-1.67863100
C	1.17906100	3.06579200	0.46748800
H	1.75740200	3.98829200	0.57846100
H	1.52102900	2.37475400	1.25155500
F	2.40750300	-2.75429100	0.42750100

(*S*)-**TS1**$_G$-F

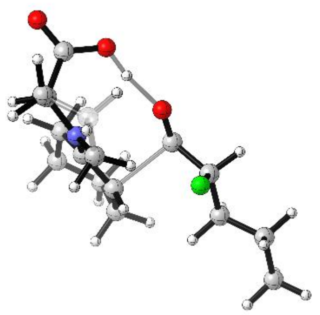

- Thermochemistry -

Zero-point correction= 0.410799 (Hartree/Particle)
Thermal correction to Energy= 0.428426
Thermal correction to Enthalpy= 0.429291
Thermal correction to Gibbs Free Energy= 0.367240
Sum of electronic and zero-point Energies= -1004.842923
Sum of electronic and thermal Energies= -1004.825296
Sum of electronic and thermal Enthalpies= -1004.824431
Sum of electronic and thermal Free Energies= -1004.886483

Number of Imaginary Frequencies = 1

E (Single Point Energy) [IEFPCM$_{(DCM)}$M06-2X/6-311++G(2d,2p)] = -1005.529150

C	0.60367800	0.89308500	-0.84361600
H	1.01458400	0.09625700	-1.45893100
C	-0.77290800	0.90813100	-0.64504400
N	-1.51670400	-0.20139500	-0.80229800
C	-2.95320100	-0.28684400	-0.52578800
H	-3.50201900	0.52492200	-1.00870400
C	-1.00668000	-1.43543400	-1.42544200
H	-0.03173200	-1.69350900	-1.01742200
H	-0.92024100	-1.28095200	-2.50993200
C	-2.07647400	-2.46426800	-1.08161400
H	-1.90139200	-2.84574800	-0.07001500
H	-2.08069100	-3.30659900	-1.77523100
C	-3.36540500	-1.64364900	-1.13367300
H	-3.67112300	-1.48623100	-2.17191900
H	-4.20273700	-2.08849900	-0.59394000
C	-1.45906100	2.11730800	-0.04682900
H	-2.48397500	2.18853000	-0.41854100
H	-1.53307700	1.96100800	1.03990700
C	0.95969200	-0.22884800	1.06949900
H	1.10411700	0.74330300	1.56510100

C	2.24598000	-0.99491700	0.76174700
O	-0.04612500	-0.93820000	1.32286000
C	-3.33385200	-0.21616000	0.96212900
O	-2.41242600	-0.40315100	1.87804200
O	-4.49826700	-0.01240500	1.25403100
C	3.46773700	-0.24140300	0.28401800
H	3.30107300	0.10122900	-0.74325600
C	4.73110900	-1.10210900	0.33673200
H	4.90322100	-1.42637700	1.37038300
H	4.57287400	-2.00917700	-0.25702900
H	2.47985500	-1.50941200	1.70649200
H	3.60154300	0.64729900	0.91411600
C	5.95512200	-0.35032400	-0.18071200
H	6.13971200	0.55097800	0.41307000
H	6.85218600	-0.97328700	-0.13484100
H	5.81214700	-0.04206900	-1.22149800
H	-1.44182600	-0.56612200	1.56204600
C	-0.71533700	3.42162400	-0.32613700
C	1.40213500	2.18439200	-0.80834800
H	-0.80314800	3.67247000	-1.39064600
H	-1.18826100	4.23060100	0.23776200
H	2.41403100	1.99568600	-0.44327800
H	1.52119800	2.55730600	-1.83573100
C	0.75477000	3.27583900	0.04265900
H	1.28896100	4.21991500	-0.10097700
H	0.83507400	3.02329100	1.10857700
F	1.95192000	-2.00706100	-0.15849000

(*S*)-**TS1**$_G$-F-P

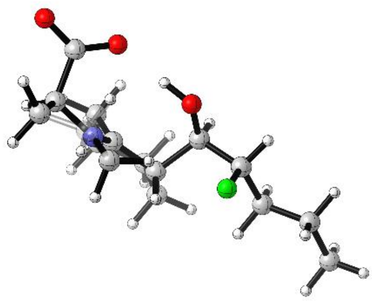

- Thermochemistry -

Zero-point correction=	0.415163 (Hartree/Particle)
Thermal correction to Energy=	0.432659
Thermal correction to Enthalpy=	0.433524
Thermal correction to Gibbs Free Energy=	0.371678
Sum of electronic and zero-point Energies=	-1004.872504
Sum of electronic and thermal Energies=	-1004.855008
Sum of electronic and thermal Enthalpies=	-1004.854143
Sum of electronic and thermal Free Energies=	-1004.915989

Number of Imaginary Frequencies = 0

E (Single Point Energy) [IEFPCM$_{(DCM)}$M06-2X/6-311++G(2d,2p)] = -1005.562739

C	0.62617700	0.62009200	-0.55443300
H	0.84742500	-0.12729600	-1.32061500
C	-0.86595700	0.80699300	-0.47357100
N	-1.65190500	-0.17087700	-0.77935700
C	-3.11055400	-0.18194200	-0.51520600
H	-3.57166400	0.71090400	-0.93923800
C	-1.20049800	-1.50594200	-1.27653700
H	-0.30300800	-1.81398700	-0.74380500
H	-0.99165400	-1.40258900	-2.34636500

C	-2.40346700	-2.40695300	-1.01952600
H	-2.36964800	-2.78366800	0.00760500
H	-2.41659600	-3.25807700	-1.70146600
C	-3.59010900	-1.46131900	-1.20270900
H	-3.77056800	-1.27452300	-2.26597000
H	-4.51377500	-1.81600800	-0.74574900
C	-1.39793100	2.08297700	0.11096500
H	-2.47305300	2.17355700	-0.03893600
H	-1.24619800	1.99095400	1.19673900
C	1.09794200	0.02815100	0.82760100
H	1.34112700	0.86778300	1.49691000
C	2.35210300	-0.83765100	0.67913900
O	0.16410900	-0.83957800	1.42195600
C	-3.40000000	-0.19564800	1.02741600
O	-2.41476600	-0.17883700	1.81126300
O	-4.60901500	-0.21312800	1.30739300
C	3.63525400	-0.19369200	0.19374300
H	3.52819300	0.06869200	-0.86572300
C	4.84083800	-1.11972900	0.36371700
H	4.96273000	-1.35765700	1.42737300
H	4.64187900	-2.06598900	-0.15143500
H	2.50923200	-1.31476300	1.65339900
H	3.80232200	0.73705000	0.75109800
C	6.12358400	-0.49359600	-0.17794800
H	6.34652900	0.44724000	0.33599800
H	6.97860200	-1.16125800	-0.04352700
H	6.03035700	-0.27699600	-1.24713600
H	-0.73354100	-0.45621100	1.57255800

C	-0.65834100	3.32406600	-0.39856000
C	1.34561900	1.93299900	-0.93414700
H	-0.87108300	3.47357400	-1.46380500
H	-1.04495100	4.19636900	0.13440800
H	2.41798900	1.81368500	-0.77851500
H	1.20520700	2.09360500	-2.00969700
C	0.84092400	3.16499800	-0.18639000
H	1.37605200	4.04789600	-0.54770600
H	1.05358100	3.08271600	0.88720400
F	2.03241400	-1.88103900	-0.21159200

(*R*)-**TS1**$_{\mathrm{O}}$-F-Pre

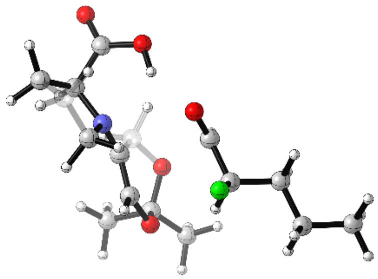

- Thermochemistry -

Zero-point correction=	0.418142 (Hartree/Particle)
Thermal correction to Energy=	0.439414
Thermal correction to Enthalpy=	0.440279
Thermal correction to Gibbs Free Energy=	0.367920
Sum of electronic and zero-point Energies=	-1155.234115
Sum of electronic and thermal Energies=	-1155.212844
Sum of electronic and thermal Enthalpies=	-1155.211979
Sum of electronic and thermal Free Energies=	-1155.284337

Number of Imaginary Frequencies = 0

E (Single Point Energy) [IEFPCM$_{\mathrm{(DCM)}}$M06-2X/6-311++G(2d,2p)] = -1155.977106

C	-0.01969800	1.02725200	1.28218500

H	-0.15913200	0.48780400	2.21087100
C	0.96443300	0.77938100	0.39865500
N	1.89250500	-0.24942700	0.55674800
C	3.22526900	-0.15118400	-0.03622700
H	3.54687000	0.88287400	-0.19907600
C	1.92065000	-0.99274400	1.81760200
H	1.01149800	-1.59510500	1.91514800
H	1.97807600	-0.30109400	2.67392300
C	3.19466000	-1.82273800	1.69998700
H	3.01015300	-2.71256300	1.08839700
H	3.57240800	-2.14547800	2.67142800
C	4.14504100	-0.85827500	0.98384500
H	4.52362200	-0.11296200	1.68889900
H	4.99713900	-1.34127800	0.50256400
C	0.98595100	1.54750900	-0.90019500
H	1.79280000	2.29375500	-0.92534700
H	1.13690900	0.86606700	-1.74495900
O	-1.01326100	1.95428400	1.06730900
O	-0.27313500	2.16137100	-1.12163800
C	-0.77225000	2.87069200	-0.00212100
C	-2.12333000	3.41513400	-0.41039600
H	-2.60741600	3.88211700	0.44957900
H	-1.99956500	4.15815800	-1.20096400
C	0.18824400	3.95897800	0.46532200
H	-0.30522800	4.56964200	1.22412800
H	0.46244600	4.59448800	-0.38060000
H	1.09384800	3.53509400	0.90540200
C	-1.04040600	-1.14593400	-0.61062100

H	-0.95999200	-0.37698100	-1.40213300
C	-2.29547100	-1.05693300	0.22113100
O	-0.20098400	-2.01183900	-0.47394800
C	3.30980300	-0.85169400	-1.39755500
O	2.29102100	-1.63122000	-1.74673000
O	4.27724000	-0.72264200	-2.11585400
H	-2.74832100	2.59703500	-0.77698100
C	-3.51858500	-1.44190500	-0.60219900
H	-3.53875700	-0.81484800	-1.50272200
C	-4.82104800	-1.26951800	0.17850800
H	-4.77534100	-1.87140400	1.09216400
H	-4.91389700	-0.22212100	0.49113500
H	-3.40356600	-2.48219000	-0.93157600
C	-6.03715700	-1.67534900	-0.65079900
H	-6.11100200	-1.06937500	-1.55962600
H	-5.96986500	-2.72553200	-0.95229000
H	-6.96255200	-1.54682300	-0.08338500
H	1.57391500	-1.62067000	-1.07200400
H	-2.39052200	-0.02976300	0.59910100
F	-2.17619500	-1.90539100	1.31398200

(*R*)-**TS1**$_{\mathrm{O}}$-F

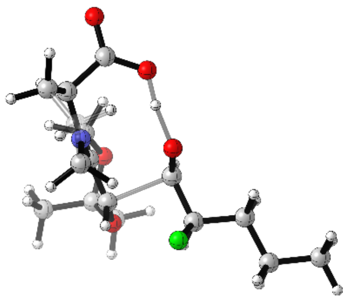

- Thermochemistry -

Zero-point correction=	0.417229 (Hartree/Particle)
Thermal correction to Energy=	0.436919
Thermal correction to Enthalpy=	0.437784
Thermal correction to Gibbs Free Energy=	0.371031
Sum of electronic and zero-point Energies=	-1155.226124
Sum of electronic and thermal Energies=	-1155.206435
Sum of electronic and thermal Enthalpies=	-1155.205570
Sum of electronic and thermal Free Energies=	-1155.272322

Number of Imaginary Frequencies = 1

E (Single Point Energy) [IEFPCM$_{(DCM)}$M06-2X/6-311++G(2d,2p)] = -1155.966865

C	-0.19474600	0.95633800	0.92177900
H	-0.50136200	0.48215700	1.84731700
C	1.05748800	0.72646700	0.37057700
N	1.76938200	-0.35882400	0.67901800
C	3.01714800	-0.73676700	0.01354300
H	3.66685000	0.13222300	-0.12607100
C	1.45505700	-1.22908500	1.82925300
H	0.40748900	-1.53133600	1.79912100
H	1.65258000	-0.67575800	2.75688700
C	2.41679000	-2.39932000	1.64231900
H	1.97358700	-3.13549200	0.96351800
H	2.64816500	-2.89495900	2.58600800
C	3.64057900	-1.74408100	0.99641700
H	4.21950100	-1.19816100	1.74684800
H	4.30377800	-2.44309500	0.48594800
C	1.52835800	1.62088400	-0.74934000
H	2.44604500	2.14597100	-0.44676900
H	1.75309900	1.03208200	-1.64461600
O	-0.88931000	2.12363200	0.66709600

O	0.51273400	2.52535900	-1.12750400
C	-0.16647900	3.13164700	-0.04219800
C	-1.20004500	4.05515900	-0.64662200
H	-1.83252000	4.46552700	0.14294400
H	-0.70257400	4.87366800	-1.17073200
C	0.78630000	3.85179800	0.90654400
H	0.20243600	4.42183300	1.63179800
H	1.41901500	4.53947500	0.33963200
H	1.42309500	3.15580200	1.45817600
C	-1.05536200	-0.42123200	-0.42033700
H	-1.01376500	0.31223400	-1.24297800
C	-2.40066200	-0.46289400	0.28352900
O	-0.39802300	-1.50447900	-0.49235800
C	2.84324800	-1.36456400	-1.38523400
O	1.64812200	-1.66244700	-1.81916700
O	3.85006200	-1.55959500	-2.04467000
H	-1.81785300	3.49496300	-1.35174500
C	-3.41629800	-1.22611200	-0.55138800
H	-3.47467600	-0.74793800	-1.53780900
C	-4.80264300	-1.26150400	0.09066400
H	-4.72788100	-1.71589000	1.08439100
H	-5.15888400	-0.23425700	0.23706700
H	-3.03509600	-2.24266100	-0.70594000
C	-5.80295800	-2.03999700	-0.76143000
H	-5.90675400	-1.58782100	-1.75319800
H	-5.47420800	-3.07518400	-0.89854800
H	-6.79132400	-2.05952100	-0.29453400
H	0.79665600	-1.53876600	-1.18396300

H	-2.74894000	0.55089900	0.50627400
F	-2.23727400	-1.12604600	1.50691400

(*R*)-**TS1**$_{O}$-F-P

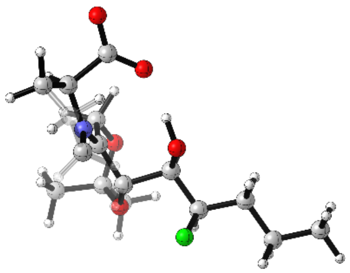

- Thermochemistry -

Zero-point correction=	0.421340 (Hartree/Particle)
Thermal correction to Energy=	0.441228
Thermal correction to Enthalpy=	0.442093
Thermal correction to Gibbs Free Energy=	0.374432
Sum of electronic and zero-point Energies=	-1155.249207
Sum of electronic and thermal Energies=	-1155.229319
Sum of electronic and thermal Enthalpies=	-1155.228454
Sum of electronic and thermal Free Energies=	-1155.296115

Number of Imaginary Frequencies = 0

E (Single Point Energy) [IEFPCM$_{(DCM)}$M06-2X/6-311++G(2d,2p)] = -1155.993515

C	-0.30733000	0.68729300	0.73125200
H	-0.35126700	0.31882900	1.75863800
C	1.09009900	0.64137000	0.19238700
N	1.94855600	-0.21250100	0.61963900
C	3.19791300	-0.52479100	-0.10052300
H	3.68180200	0.39735800	-0.43089700
C	1.77370000	-1.11996900	1.79636400
H	0.79255200	-1.58883100	1.75325800
H	1.86236600	-0.49918300	2.69275200

C	2.93389800	-2.10519300	1.66160800
H	2.62640400	-2.96082200	1.05293700
H	3.25454000	-2.47275200	2.63678600
C	4.01203100	-1.29794400	0.93390700
H	4.50862400	-0.60561900	1.62064300
H	4.76422800	-1.91468300	0.44308300
C	1.38722300	1.52920100	-0.98522400
H	2.30344400	2.10691900	-0.80087900
H	1.53947400	0.90485200	-1.87174400
O	-0.89810800	1.96946500	0.70804600
O	0.28475300	2.36496300	-1.24781600
C	-0.27086400	2.97404200	-0.09512000
C	-1.37446700	3.88593700	-0.58355200
H	-1.92683400	4.28249200	0.27036000
H	-0.94762000	4.71432600	-1.15272800
C	0.77469400	3.70955200	0.73830100
H	0.26416300	4.27258100	1.52171800
H	1.33724400	4.40368500	0.10860200
H	1.47497000	3.02657200	1.22805000
C	-1.13370800	-0.28499000	-0.18558500
H	-1.15708000	0.15683800	-1.19362000
C	-2.56701100	-0.34055700	0.32596000
O	-0.58892200	-1.57254600	-0.18279700
C	2.87393300	-1.39414800	-1.37797600
O	1.66087100	-1.55773800	-1.67552800
O	3.88129900	-1.80836300	-1.96517900
H	-2.05457900	3.31735800	-1.22156600
C	-3.44458100	-1.30111200	-0.44922100

H	-3.40125600	-1.01708600	-1.50886900
C	-4.89526300	-1.29464700	0.03119200
H	-4.92321000	-1.54752900	1.09669000
H	-5.30446800	-0.28096800	-0.06399100
H	-3.01540300	-2.30550100	-0.36777800
C	-5.76028400	-2.27682600	-0.75589000
H	-5.76183400	-2.02840900	-1.82234100
H	-5.38099200	-3.29854200	-0.65177300
H	-6.79583100	-2.26466400	-0.40538000
H	0.21117300	-1.60662400	-0.76154000
H	-2.98579200	0.67123500	0.32902600
F	-2.52374900	-0.75376700	1.67133500

(*S*)-**TS1**$_{O}$-F-Pre

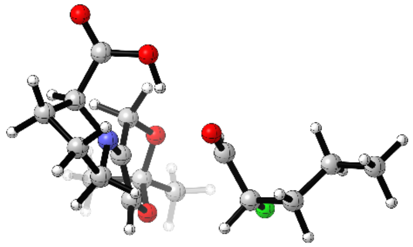

- Thermochemistry -

Zero-point correction=	0.421340 (Hartree/Particle)
Thermal correction to Energy=	0.441228
Thermal correction to Enthalpy=	0.442093
Thermal correction to Gibbs Free Energy=	0.374432
Sum of electronic and zero-point Energies=	-1155.249207
Sum of electronic and thermal Energies=	-1155.229319
Sum of electronic and thermal Enthalpies=	-1155.228454
Sum of electronic and thermal Free Energies=	-1155.296115

Number of Imaginary Frequencies = 0

E (Single Point Energy) [IEFPCM$_{(DCM)}$M06-2X/6-311++G(2d,2p)] =

(*S*)-**TS1**$_{O}$-F

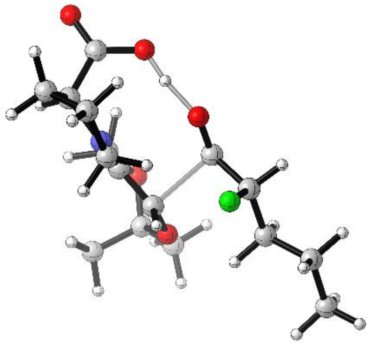

- Thermochemistry -

Zero-point correction=	0.417306 (Hartree/Particle)
Thermal correction to Energy=	0.436926
Thermal correction to Enthalpy=	0.437791
Thermal correction to Gibbs Free Energy=	0.371627
Sum of electronic and zero-point Energies=	-1155.222032
Sum of electronic and thermal Energies=	-1155.202412
Sum of electronic and thermal Enthalpies=	-1155.201547
Sum of electronic and thermal Free Energies=	-1155.267711

Number of Imaginary Frequencies = 1

E (Single Point Energy) [IEFPCM$_{(DCM)}$M06-2X/6-311++G(2d,2p)] = -1155.962553

C	-0.58752700	0.40228300	0.70680200
H	-0.86389100	-0.33337100	1.45527400
C	0.74214500	0.69983000	0.44498600
N	1.72448000	-0.15779100	0.72740000
C	3.12704100	0.05108300	0.36163200
H	3.43648800	1.08082300	0.56414700
C	1.53647700	-1.33654300	1.59399600
H	0.68113400	-1.92173000	1.25410100

H	1.36364300	-0.99552600	2.62360000
C	2.86313600	-2.07630200	1.45131100
H	2.83646700	-2.71019100	0.55866800
H	3.07862800	-2.70502500	2.31620300
C	3.87414900	-0.94054400	1.27235700
H	4.07701100	-0.45875800	2.23306100
H	4.82206700	-1.25081300	0.83166300
C	1.06605400	1.93615500	-0.35729900
H	1.68014200	2.62068800	0.24620800
H	1.63328800	1.67406200	-1.25600500
O	-1.57897300	1.34276200	0.48868300
O	-0.11184900	2.56406400	-0.81341500
C	-1.13403600	2.65803000	0.16290200
C	-2.30126200	3.34939000	-0.50576300
H	-3.16166100	3.35107100	0.16627000
H	-2.55976100	2.81547500	-1.42291500
C	-0.66419900	3.37837100	1.42325000
H	-0.21343100	4.33669900	1.15272000
H	0.06394400	2.79017100	1.98735200
H	-1.52455000	3.56033800	2.07030600
C	-0.68670800	-0.89982600	-0.98786700
H	-0.91121900	-0.03112700	-1.62701200
C	-1.90128300	-1.72825800	-0.57392200
O	0.38761700	-1.55370900	-1.13865000
C	3.46052500	-0.20303400	-1.12286400
O	2.55240700	-0.70902700	-1.91532100
O	4.57907500	0.08768800	-1.50908500
H	-2.03203000	4.37939300	-0.74852100

C	-3.21229800	-1.01639000	-0.31679100
H	-3.14095100	-0.43825800	0.60962600
C	-4.38817200	-1.99064700	-0.23740300
H	-4.46841400	-2.53982300	-1.18374100
H	-4.18854600	-2.73428400	0.54256400
H	-3.37767200	-0.29296400	-1.12546400
C	-5.70356500	-1.27325300	0.05673700
H	-5.92825000	-0.53640300	-0.72149100
H	-6.53865900	-1.97708400	0.10562600
H	-5.65260200	-0.74349000	1.01354500
H	1.61749600	-1.00529600	-1.51464600
H	-2.02676800	-2.45985800	-1.38563000
F	-1.54914900	-2.47627500	0.55918000

(*S*)-**TS1**$_{O}$-F-P

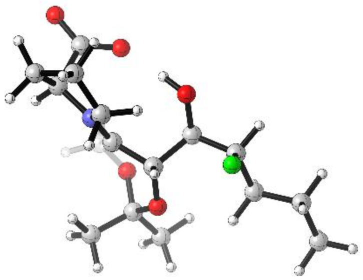

- Thermochemistry -

Zero-point correction= 0.421565 (Hartree/Particle)
Thermal correction to Energy= 0.441354
Thermal correction to Enthalpy= 0.442219
Thermal correction to Gibbs Free Energy= 0.374972
Sum of electronic and zero-point Energies= -1155.246431
Sum of electronic and thermal Energies= -1155.226642
Sum of electronic and thermal Enthalpies= -1155.225777
Sum of electronic and thermal Free Energies= -1155.293024

Number of Imaginary Frequencies = 0

E (Single Point Energy) [IEFPCM$_{(DCM)}$M06-2X/6-311++G(2d,2p)] = -1155.990751

C	-0.45145900	0.08783400	0.60117100
H	-0.44421300	-0.48930500	1.52865600
C	0.92327800	0.59332800	0.26879600
N	1.98325400	-0.00794000	0.67077100
C	3.32380800	0.26614500	0.11760700
H	3.48410800	1.34422300	0.03995500
C	2.02832500	-1.15785500	1.62639100
H	1.27622500	-1.89286500	1.34545300
H	1.81711100	-0.75389600	2.62092200
C	3.46454400	-1.66503600	1.50443900
H	3.52782700	-2.41540300	0.71063000
H	3.80352600	-2.11856600	2.43625100
C	4.25736900	-0.41445900	1.11557800
H	4.41771600	0.22957000	1.98551500
H	5.21848400	-0.63219900	0.65081700
C	0.99407100	1.75583400	-0.68347600
H	1.63376600	2.54733500	-0.26973300
H	1.42720200	1.41325800	-1.62888400
O	-1.40532300	1.11630100	0.75585900
O	-0.30437600	2.22215900	-0.96527800
C	-1.11900600	2.39621100	0.18009000
C	-2.43600900	2.95537100	-0.31186600
H	-3.15565400	2.97084100	0.50915800
H	-2.81935200	2.32196200	-1.11480700
C	-0.46220500	3.28121000	1.23531300

H	-0.14191900	4.22604300	0.78840900
H	0.39936700	2.79809500	1.70511400
H	-1.19122300	3.48792000	2.02104100
C	-0.84558200	-0.87209300	-0.57549800
H	-0.99389500	-0.25884200	-1.47622500
C	-2.14900600	-1.61176800	-0.27744200
O	0.14215300	-1.85310800	-0.76036500
C	3.44256300	-0.35270400	-1.33024500
O	2.39427800	-0.82096400	-1.84766600
O	4.58235800	-0.28354100	-1.80715900
H	-2.29365000	3.97147500	-0.68583200
C	-3.43625900	-0.81172100	-0.31419900
H	-3.43103700	-0.07512100	0.49451100
C	-4.67230700	-1.70595700	-0.21398400
H	-4.66917900	-2.42425200	-1.04334200
H	-4.61983600	-2.29144800	0.71071700
H	-3.46088000	-0.25062600	-1.25754100
C	-5.96518900	-0.89365300	-0.23844100
H	-6.04542000	-0.31532700	-1.16490800
H	-6.84381000	-1.54042000	-0.16818100
H	-5.99764000	-0.18898300	0.59902000
H	0.94076200	-1.47868100	-1.20478000
H	-2.19582900	-2.44255700	-0.99155600
F	-2.01996400	-2.20417300	0.99285500

(*R*)-**TS1**$_{T}$-F-Pre

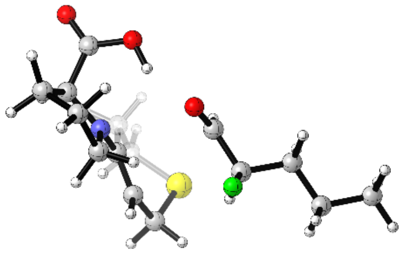

- Thermochemistry -

Zero-point correction=	0.383204 (Hartree/Particle)
Thermal correction to Energy=	0.402801
Thermal correction to Enthalpy=	0.403666
Thermal correction to Gibbs Free Energy=	0.334681
Sum of electronic and zero-point Energies=	-1363.741005
Sum of electronic and thermal Energies=	-1363.721408
Sum of electronic and thermal Enthalpies=	-1363.720543
Sum of electronic and thermal Free Energies=	-1363.789529

Number of Imaginary Frequencies = 0

E (Single Point Energy) [IEFPCM$_{(DCM)}$M06-2X/6-311++G(2d,2p)] = -1364.424378

C	-0.09201800	1.19348300	-1.51712000
H	0.04868600	0.50188800	-2.34230800
C	-1.08327000	0.96460900	-0.62651400
N	-1.83686500	-0.20664600	-0.66356000
C	-3.19013900	-0.29034200	-0.12129200
H	-3.76562000	0.62999800	-0.25925400
C	-1.61923700	-1.19780700	-1.71804800
H	-0.58651700	-1.55735700	-1.68326100
H	-1.80700600	-0.76167700	-2.71217700

C	-2.64350000	-2.27668200	-1.38860900
H	-2.26444200	-2.91709100	-0.58463800
H	-2.88043600	-2.90642200	-2.24781300
C	-3.84075400	-1.45214200	-0.91018200
H	-4.37387400	-1.03325300	-1.76813800
H	-4.55688900	-2.01078200	-0.30443700
C	-1.38207000	1.87479500	0.54759100
H	-2.45997400	2.06794100	0.59763000
H	-1.11974200	1.35014000	1.47638300
C	1.13237500	-0.71984100	0.70138000
H	1.03195900	0.15350000	1.37056400
C	2.39078100	-0.74228800	-0.12951900
O	0.30826500	-1.61071700	0.68364400
C	-3.22185100	-0.59490700	1.37838600
O	-2.14270800	-1.15829100	1.91364500
O	-4.20824800	-0.37537400	2.04805800
C	3.61375400	-0.97593000	0.74817300
H	3.62532200	-0.20667600	1.53081200
C	4.91781300	-0.92762300	-0.04728000
H	4.88025000	-1.67586700	-0.84586200
H	5.00477800	0.05251800	-0.53197000
H	2.47663900	0.21380800	-0.66147500
H	3.50410700	-1.94749500	1.24600800
C	6.13312100	-1.17771300	0.84254600
H	6.19799300	-0.42671600	1.63645200
H	6.07293000	-2.16273400	1.31619000
H	7.05987300	-1.13836200	0.26433100
H	-1.40188300	-1.24420500	1.27085900

C	-0.67716400	3.22540300	0.50574000
C	0.86558200	2.35493600	-1.49517700
H	-1.11345500	3.86888400	-0.26461600
H	-0.77924800	3.73252600	1.46671400
H	1.85430300	2.03714100	-1.84010200
H	0.54425600	3.15729400	-2.17025800
S	1.09976000	3.06317600	0.16348500
F	2.28787500	-1.74821600	-1.07929900

(*R*)-**TS1**$_T$-F

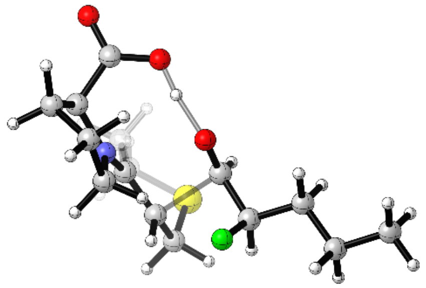

- Thermochemistry -

Zero-point correction= 0.383176 (Hartree/Particle)
Thermal correction to Energy= 0.401127
Thermal correction to Enthalpy= 0.401992
Thermal correction to Gibbs Free Energy= 0.338653
Sum of electronic and zero-point Energies= -1363.732980
Sum of electronic and thermal Energies= -1363.715029
Sum of electronic and thermal Enthalpies= -1363.714164
Sum of electronic and thermal Free Energies= -1363.777503

Number of Imaginary Frequencies = 1

E (Single Point Energy) [IEFPCM$_{(DCM)}$M06-2X/6-311++G(2d,2p)] = -1364.414632

C	0.08134300	1.15049800	-1.12315300
H	0.47068800	0.53827400	-1.93191900
C	-1.17308000	0.81690100	-0.61974100

N	-1.65918300	-0.42377600	-0.78693100
C	-2.89563400	-0.92522500	-0.17905400
H	-3.73096300	-0.24254200	-0.34946000
C	-1.06125700	-1.41343900	-1.70624400
H	0.01943000	-1.44754400	-1.58071600
H	-1.30333400	-1.12994500	-2.73939800
C	-1.73773400	-2.71670300	-1.29975600
H	-1.21359100	-3.14554600	-0.43913300
H	-1.73896700	-3.45058000	-2.10708500
C	-3.14291400	-2.26348500	-0.90369900
H	-3.74615300	-2.07541100	-1.79640200
H	-3.67962300	-2.97032200	-0.26923700
C	-1.94727500	1.72926500	0.30776900
H	-3.01866300	1.59578800	0.13182700
H	-1.75437800	1.41449200	1.34250600
C	1.11866300	0.03795800	0.45709000
H	1.05665500	0.91720100	1.11645500
C	2.44888500	-0.12043500	-0.25974100
O	0.48587200	-1.01705400	0.73270200
C	-2.82984700	-1.13132200	1.34463700
O	-1.66554200	-1.17314300	1.94492400
O	-3.87626200	-1.27480500	1.95136400
C	3.49203000	-0.68926900	0.69002900
H	3.55520000	-0.02728000	1.56341900
C	4.86895100	-0.82826300	0.04205500
H	4.78741300	-1.46727500	-0.84369000
H	5.20507900	0.15636700	-0.30618700
H	2.79229400	0.82726600	-0.68577200

H	3.13031000	-1.66146600	1.04574500
C	5.89537600	-1.41292100	1.01018500
H	6.00568700	-0.77665000	1.89432400
H	5.58744900	-2.40706700	1.34967300
H	6.87677800	-1.50730000	0.53787500
H	-0.79970500	-1.06031100	1.37065800
C	-1.62818200	3.21180600	0.15934000
C	0.63339100	2.55659000	-1.11490700
H	-1.96564100	3.58896200	-0.81093900
H	-2.14067600	3.77847900	0.93845100
H	1.72668600	2.53770900	-1.11144200
H	0.33644400	3.09660000	-2.02139800
S	0.14583900	3.54310000	0.33037200
F	2.28151100	-1.01075400	-1.32626200

(*R*)-**TS1**$_{\mathrm{T}}$-F-P

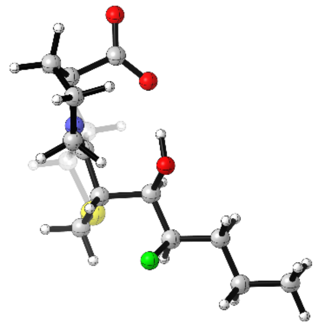

- Thermochemistry -

Zero-point correction=	0.387768 (Hartree/Particle)
Thermal correction to Energy=	0.405714
Thermal correction to Enthalpy=	0.406579
Thermal correction to Gibbs Free Energy=	0.342962
Sum of electronic and zero-point Energies=	-1363.760167
Sum of electronic and thermal Energies=	-1363.742222
Sum of electronic and thermal Enthalpies=	-1363.741357

Sum of electronic and thermal Free Energies= -1363.804973

Number of Imaginary Frequencies = 0

E (Single Point Energy) [IEFPCM$_{(DCM)}$M06-2X/6-311++G(2d,2p)] = -1364.445963

C	0.28875200	0.79294200	-0.85772200
H	0.32538200	0.24326800	-1.79934800
C	-1.11260900	0.79355300	-0.31036400
N	-1.96061900	-0.09841200	-0.70060300
C	-3.23189200	-0.39471200	-0.00030600
H	-3.80109800	0.51987300	0.16472600
C	-1.71808100	-1.11152900	-1.77653600
H	-0.70973400	-1.50791000	-1.68800000
H	-1.84617300	-0.60070700	-2.73566900
C	-2.79815400	-2.16296700	-1.53986600
H	-2.44113400	-2.90492600	-0.81903000
H	-3.05920800	-2.67707600	-2.46559500
C	-3.95099500	-1.35592700	-0.94508600
H	-4.47396700	-0.79610900	-1.72672400
H	-4.67604200	-1.95768000	-0.39715100
C	-1.43636400	1.73323500	0.82005500
H	-2.50513400	1.73968100	1.03168600
H	-0.94427300	1.32554500	1.71197100
C	1.13203700	-0.02995200	0.17530100
H	1.13270000	0.49530900	1.14090600
C	2.58810900	-0.11431100	-0.26570600
O	0.60840600	-1.32659600	0.30590500
C	-2.92454600	-1.05197900	1.39531100
O	-1.71551300	-1.20091400	1.71671600
O	-3.93987300	-1.35123900	2.04080400

C	3.40619900	-1.11181000	0.52758900
H	3.28042000	-0.87866900	1.59297800
C	4.88874400	-1.08578000	0.15840300
H	4.99763600	-1.28499800	-0.91342500
H	5.28998000	-0.07989600	0.33552900
H	3.04253200	0.88202500	-0.20594800
H	2.98361400	-2.10909600	0.36791100
C	5.69213900	-2.10919200	0.95816000
H	5.61226500	-1.91454600	2.03271700
H	5.32239600	-3.12311500	0.77428200
H	6.75132200	-2.08263700	0.68846600
H	-0.21566000	-1.30993100	0.84903600
C	-0.97063000	3.17477100	0.58588000
C	0.81804600	2.20328200	-1.15078600
H	-1.50416500	3.62477800	-0.25674700
H	-1.19175700	3.76446500	1.47677500
H	1.84147400	2.14334400	-1.52455500
H	0.21457200	2.66066900	-1.94083900
S	0.81558800	3.30230100	0.29301200
F	2.62225300	-0.47859400	-1.62569900

(*S*)-**TS1**$_{T}$-F-Pre

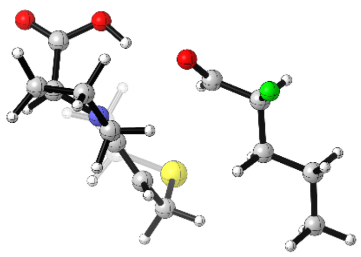

- Thermochemistry -

Zero-point correction= 0.383296 (Hartree/Particle)
Thermal correction to Energy= 0.402792
Thermal correction to Enthalpy= 0.403657
Thermal correction to Gibbs Free Energy= 0.335908
Sum of electronic and zero-point Energies= -1363.741222
Sum of electronic and thermal Energies= -1363.721726
Sum of electronic and thermal Enthalpies= -1363.720861
Sum of electronic and thermal Free Energies= -1363.788610

Number of Imaginary Frequencies = 0

E (Single Point Energy) [IEFPCM$_{(DCM)}$M06-2X/6-311++G(2d,2p)] = -1364.424621

C	0.41756400	1.05646300	-1.33289900
H	0.61072800	0.32604600	-2.11295100
C	-0.72746600	0.97158000	-0.62260900
N	-1.60135700	-0.10921400	-0.76794700
C	-3.04414700	0.03374000	-0.57885900
H	-3.42541400	1.00587100	-0.90776300
C	-1.29702500	-1.18308800	-1.71240600
H	-0.35129800	-1.66158400	-1.43884300
H	-1.20860300	-0.79695300	-2.74069600
C	-2.50412100	-2.10277600	-1.58044300
H	-2.40716000	-2.71864800	-0.67972800
H	-2.62476400	-2.76416100	-2.44008300
C	-3.65338700	-1.10180900	-1.43758100
H	-3.91773100	-0.69389300	-2.41700400
H	-4.55700000	-1.51850200	-0.98825500
C	-1.11506600	1.95281300	0.46462300
H	-2.16365900	2.24805500	0.33909500
H	-1.05291200	1.44471900	1.43664300
C	0.88471900	-1.09883300	1.36480600
H	0.83713000	-0.05342700	1.72189300

C	2.27761700	-1.63352300	1.14263800
O	-0.11624400	-1.75714700	1.17824500
C	-3.47569000	-0.12857600	0.87955100
O	-2.64939100	-0.78262700	1.68926500
O	-4.54729600	0.27888700	1.27429100
C	3.05886900	-0.76647400	0.16850200
H	2.55100100	-0.79822300	-0.80432300
C	4.51729200	-1.19542700	0.02404300
H	5.00164000	-1.16039900	1.00750300
H	4.55721500	-2.23631300	-0.31400100
H	2.78393800	-1.66471400	2.11831300
H	3.00421600	0.26957700	0.52898300
C	5.26743000	-0.29856500	-0.95810600
H	4.80407900	-0.33427300	-1.94966000
H	5.25804000	0.74324500	-0.62039300
H	6.31008700	-0.60922200	-1.06278300
H	-1.80117900	-1.01873700	1.24707100
C	-0.28114300	3.22725400	0.49792600
C	1.48579600	2.10503200	-1.17266700
H	-0.51404800	3.86847200	-0.35795900
H	-0.48937900	3.79078800	1.40907000
H	2.47534100	1.66033900	-1.31525000
H	1.38498300	2.89752200	-1.92399600
S	1.50090600	2.87553100	0.47417800
F	2.19619400	-2.93437200	0.67115300

(*S*)-**TS1**$_T$-F

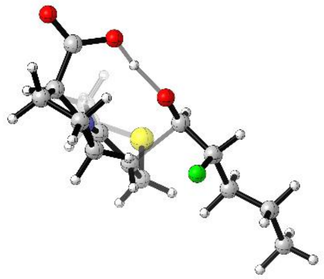

- Thermochemistry -

Zero-point correction=	0.383310 (Hartree/Particle)
Thermal correction to Energy=	0.401139
Thermal correction to Enthalpy=	0.402004
Thermal correction to Gibbs Free Energy=	0.339218
Sum of electronic and zero-point Energies=	-1363.727183
Sum of electronic and thermal Energies=	-1363.709355
Sum of electronic and thermal Enthalpies=	-1363.708490
Sum of electronic and thermal Free Energies=	-1363.771275

Number of Imaginary Frequencies = 1

E (Single Point Energy) [IEFPCM$_{(DCM)}$M06-2X/6-311++G(2d,2p)] = -1364.408701

C	0.57604500	0.65114800	-0.90257500
H	0.92044200	-0.17977500	-1.51465700
C	-0.80229700	0.75691300	-0.69940200
N	-1.59008900	-0.32083100	-0.81581900
C	-3.02596100	-0.34688600	-0.51120100
H	-3.55025700	0.47438800	-1.00446700
C	-1.13421700	-1.60079000	-1.39383700
H	-0.17059000	-1.88474600	-0.97661100
H	-1.04590900	-1.48618600	-2.48252300
C	-2.24482800	-2.57076700	-1.01135000

H	-2.07950600	-2.92798600	0.01060300
H	-2.28767300	-3.43288400	-1.67861500
C	-3.49799100	-1.69891300	-1.08229500
H	-3.80149100	-1.55621000	-2.12327200
H	-4.35040700	-2.09223400	-0.52674800
C	-1.44773300	1.99730700	-0.11526200
H	-2.45992300	2.10221200	-0.51575700
H	-1.55075500	1.84389200	0.96762400
C	0.90185200	-0.36793200	1.01691600
H	1.08469400	0.61415900	1.47845600
C	2.15440200	-1.20235000	0.75324400
O	-0.12867100	-1.03550300	1.30967300
C	-3.37791700	-0.23045000	0.98237200
O	-2.44730900	-0.41283400	1.88643700
O	-4.53395000	0.00904000	1.28158200
C	3.40809900	-0.52226900	0.24845800
H	3.27493300	-0.27257500	-0.81040100
C	4.64753100	-1.40446600	0.40678100
H	4.79502500	-1.62877000	1.47016000
H	4.47309800	-2.36157400	-0.09747200
H	2.36083000	-1.68250300	1.72176800
H	3.54942700	0.41589200	0.80105300
C	5.89872900	-0.73896800	-0.16102900
H	5.78081200	-0.53510000	-1.23025200
H	6.09673900	0.21337800	0.34174000
H	6.77844100	-1.37542900	-0.03518200
H	-1.47499200	-0.61703000	1.56677100
C	-0.70552400	3.30312200	-0.36774400

C	1.45989100	1.87888200	-0.99402200
H	-0.73134000	3.57131300	-1.42837600
H	-1.17886600	4.10848100	0.19624500
H	2.49835700	1.62906100	-0.78172500
H	1.43843000	2.29354600	-2.00902500
S	1.02330600	3.20526200	0.16545500
F	1.82301800	-2.24208900	-0.12352600

(*S*)-**TS1**$_{T}$-F-P

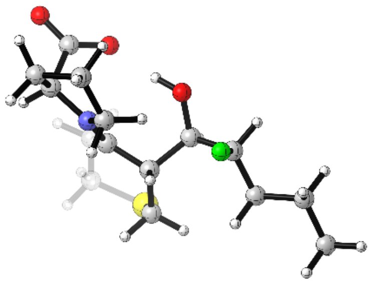

- Thermochemistry -

Zero-point correction= 0.387482 (Hartree/Particle)
Thermal correction to Energy= 0.405341
Thermal correction to Enthalpy= 0.406206
Thermal correction to Gibbs Free Energy= 0.342683
Sum of electronic and zero-point Energies= -1363.755102
Sum of electronic and thermal Energies= -1363.737244
Sum of electronic and thermal Enthalpies= -1363.736379
Sum of electronic and thermal Free Energies= -1363.799902

Number of Imaginary Frequencies = 0

E (Single Point Energy) [IEFPCM$_{(DCM)}$M06-2X/6-311++G(2d,2p)] = -1364.440624

C	0.57321900	0.39685200	-0.59771400
H	0.72367700	-0.37445800	-1.35789700
C	-0.90361400	0.67736600	-0.47887600
N	-1.74581900	-0.25594100	-0.77343500

C	-3.19890600	-0.19789700	-0.48200900
H	-3.62182200	0.72531000	-0.87951500
C	-1.37393200	-1.61165300	-1.28667300
H	-0.49183700	-1.97421700	-0.76251300
H	-1.16773400	-1.50905400	-2.35682600
C	-2.62238700	-2.44797600	-1.02787900
H	-2.59858600	-2.84097300	-0.00667600
H	-2.68867600	-3.28673000	-1.72178600
C	-3.75626100	-1.43611400	-1.18443600
H	-3.93674100	-1.22224900	-2.24256300
H	-4.69375000	-1.74496000	-0.72222200
C	-1.37206300	1.96401700	0.14573200
H	-2.44519200	2.09497700	0.00710900
H	-1.21071000	1.84479600	1.22549400
C	1.04829700	-0.19369800	0.78119700
H	1.30931300	0.64800900	1.43954800
C	2.27817800	-1.09179500	0.63447900
O	0.08961600	-1.03434500	1.37617800
C	-3.45614700	-0.22928800	1.06792400
O	-2.45328100	-0.25808400	1.82827600
O	-4.65831100	-0.20974200	1.37231700
C	3.58591800	-0.47781800	0.17314000
H	3.52536700	-0.26996100	-0.90192800
C	4.77521000	-1.40443000	0.43257500
H	4.86021800	-1.58339000	1.51109700
H	4.58014000	-2.37607100	-0.03520800
H	2.40983400	-1.57902400	1.60737900
H	3.73993800	0.47798100	0.69076700

C	6.08181200	-0.82333700	-0.10162900
H	6.02637400	-0.66902500	-1.18414600
H	6.29822700	0.14378200	0.36395700
H	6.92412700	-1.49010700	0.10020800
H	-0.78237800	-0.61178900	1.56339000
C	-0.64601900	3.22042800	-0.33604400
C	1.36617500	1.63112100	-1.06527200
H	-0.83330400	3.40528300	-1.39785600
H	-1.01646200	4.07829900	0.22696700
H	2.43253300	1.41588900	-1.06867100
H	1.08375000	1.86853300	-2.09575200
S	1.13920300	3.11608300	-0.05256800
F	1.94067800	-2.11932300	-0.26760900

Supplementary Table 6. Energies for enamine addition to 2-bromopentanal. Reported energies for structures optimized at the $IEFPCM_{(DCM)}$M06-2X/6-311++G(2d,2p)//$IEFPCM_{(DCM)}$M06-2X/6-31+G(d,p) level of theory represent the sum of the thermal correction to Gibbs Free Energy computed at the $IEFPCM_{(DCM)}$M06-2X/6-31+G(d,p) level of theory and single point energies computed at the $IEFPCM_{(DCM)}$M06-2X/6-311++G(2d,2p). All energies are reported in Hartrees.

**Structure**	**Single Point Energies, E $IEFPCM_{(DCM)}$M06-2X/6-311++G(2d,2p)**	**Thermal Corrections to Gibbs Free Energies, $IEFPCM_{(DCM)}$M06-2X/6-31+G(d,p)**	**Gibbs Free Energies (G), $IEFPCM_{(DCM)}$M06-2X/6-31+G(d,p)**	**Gibbs Free Energies (G), $IEFPCM_{(DCM)}$M06-X/6-311++G(2d,2p)// $IEFPCM_{(DCM)}$M06-2X/6-31+G(d,p)**
2-Bromopentanal	-2845.302023	0.102041	-2842.756584	-2845.199982
Enamine of Cyclohexanone (G)	-634.55147535	0.238392	-634.142903	-634.3130834
Enamine of Dioxane (O)	-784.98483791	0.244077	-784.522619	-784.7407609
Enamine of Tetrahydro-4H-thiopyranone (T)	-993.43210840	0.210205	-993.028735	-993.2219034
Enamine of Tetrahydro-4H-pyranone (P)	-670.45469021	0.214793	-670.055312	-670.2398972
(*R*)-**TS1**$_P$-Br-Pre	-3515.772806	0.33702	-3512.817561	-3515.435786
(*R*)-**TS1**$_P$-Br	-3515.764676	0.339202	-3512.807029	-3515.425474
(*R*)-**TS1**$_P$-Br-P	-3515.796502	0.343987	-3512.8352	-3515.452515
(*S*)-**TS1**$_P$-Br-Pre	-3515.773376	0.336209	-3512.816466	-3515.437167
(*S*)-**TS1**$_P$-Br	-3515.755215	0.339792	-3512.797441	-3515.415423
(*S*)-**TS1**$_P$-Br-P	-3515.791108	0.343838	-3512.829276	-3515.44727
(*R*)-**TS1**$_G$-Br-Pre	-3479.870718	0.360463	-3476.904554	-3479.510255
(*R*)-**TS1**$_G$-Br	-3479.861538	0.363361	-3476.894285	-3479.498177
(*R*)-**TS1**$_G$-Br-P	-3479.895319	0.367485	-3476.924713	-3479.527834
(*S*)-**TS1**$_G$-Br-Pre	-3479.870083	0.359689	-3476.903689	-3479.510394
(*S*)-**TS1**$_G$-Br	-3479.855148	0.363834	-3476.887892	-3479.491314
(*S*)-**TS1**$_G$-Br-P	-3479.890591	0.367929	-3476.919777	-3479.522662

(*R*)-**TS1**$_O$-Br-Pre	-3630.3055	0.366152	-3627.287997	-3629.939348
(*R*)-**TS1**$_O$-Br	-3630.294358	0.367844	-3627.274362	-3629.926514
(*R*)-**TS1**$_O$-Br-P	-3630.321486	0.366131	-3627.288017	-3629.955355
(*S*)-**TS1**$_O$-Br-Pre	-3630.304491	0.366172	-3627.285373	-3629.938319
(*S*)-**TS1**$_O$-Br	-3630.290158	0.367855	-3627.270827	-3629.922303
(*S*)-**TS1**$_O$-Br-P	-3630.318696	0.372541	-3627.295449	-3629.946155
(*R*)-**TS1**$_T$-Br-Pre	-3838.753211	0.332629	-3835.791695	-3838.420582
(*R*)-**TS1**$_T$-Br	-3838.741514	0.335333	-3835.779609	-3838.406181
(*R*)-**TS1**$_T$-Br-P	-3838.773806	0.339578	-3835.808206	-3838.43422
(*S*)-**TS1**$_T$-Br-Pre	-3838.752357	0.33267	-3835.789922	-3838.419687
(*S*)-**TS1**$_T$-Br	-3838.735267	0.335793	-3835.77367	-3838.399474
(*S*)-**TS1**$_T$-Br-P	-3838.769222	0.339134	-3835.804351	-3838.430088

Pre – Precomplex

P – Product

2-Bromopentanal

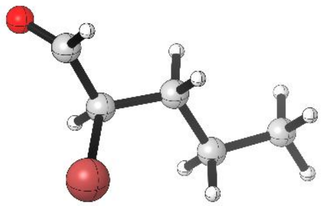

- Thermochemistry -

Zero-point correction= 0.133479 (Hartree/Particle)
Thermal correction to Energy= 0.140970
Thermal correction to Enthalpy= 0.141835
Thermal correction to Gibbs Free Energy= 0.102041
Sum of electronic and zero-point Energies= -2842.725145
Sum of electronic and thermal Energies= -2842.717655
Sum of electronic and thermal Enthalpies= -2842.716789
Sum of electronic and thermal Free Energies= -2842.756584

Number of Imaginary Frequencies = 0

E (Single Point Energy) [IEFPCM$_{(DCM)}$M06-2X/6-311++G(2d,2p)] = -2845.302023

C	1.23693100	1.54795200	-0.34621000
H	1.33300600	1.41009900	-1.43993900
C	0.25532500	0.62139900	0.33151200
O	1.86828400	2.38983000	0.24705500
C	-1.13902800	0.76859800	-0.25746500
H	-1.43667100	1.81367500	-0.09180100
C	-2.18180100	-0.15516700	0.36870400
H	-1.92606000	-1.19509100	0.14064600
H	-2.14420900	-0.05117200	1.45983700
H	-1.09415100	0.61296000	-1.34243600
C	-3.58582700	0.16153200	-0.14039800
H	-3.63780100	0.05803700	-1.22904700
H	-4.32494700	-0.51457200	0.29680400
H	-3.87364000	1.18651500	0.11378600
H	0.28586600	0.75505500	1.41314000
Br	0.98167800	-1.16743000	-0.02955100

(R)-**TS1**$_P$-Br-Pre

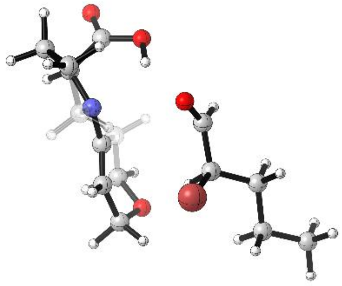

- Thermochemistry -

Zero-point correction=	0.384883 (Hartree/Particle)
Thermal correction to Energy=	0.404397
Thermal correction to Enthalpy=	0.405262
Thermal correction to Gibbs Free Energy=	0.337020
Sum of electronic and zero-point Energies=	-3512.769697
Sum of electronic and thermal Energies=	-3512.750183
Sum of electronic and thermal Enthalpies=	-3512.749318
Sum of electronic and thermal Free Energies=	-3512.817561

Number of Imaginary Frequencies = 0

E (Single Point Energy) [IEFPCM$_{(DCM)}$M06-2X/6-311++G(2d,2p)] = -3515.772806

C	-0.22947700	0.59828800	-1.86474200
H	-0.22583200	-0.31834000	-2.44407100
C	-1.15552000	0.82340600	-0.91501300
N	-2.09640100	-0.13447000	-0.52583200
C	-3.43259700	0.25565000	-0.07844900
H	-3.80921700	1.15605700	-0.57400600
C	-2.11815800	-1.43874600	-1.18866500
H	-1.16847900	-1.95277100	-1.01104500
H	-2.26409000	-1.32921900	-2.27542000
C	-3.31873200	-2.12135700	-0.54518700
H	-3.04506100	-2.50944400	0.44213300
H	-3.70366100	-2.94870300	-1.14379900
C	-4.31934100	-0.96754200	-0.42238800
H	-4.80021900	-0.78607800	-1.38737100
H	-5.10253300	-1.13279400	0.32041900
C	-1.15339500	2.12214700	-0.14086300
H	-2.16079600	2.55125600	-0.09184300
H	-0.83833800	1.94353600	0.89682600
C	0.66603300	-0.51047100	1.48835400
H	0.52066000	0.25922500	2.27260100

C	1.88075700	-0.26007100	0.63844900
O	-0.13192900	-1.40929200	1.35364000
C	-3.47762400	0.53110600	1.42480200
O	-2.53861500	-0.06001400	2.16440700
O	-4.34211800	1.20803800	1.93554500
C	3.14691200	-0.01573000	1.45383600
H	2.88564400	0.69341100	2.25136700
C	4.28161200	0.57026700	0.61559100
H	4.56684000	-0.15213200	-0.15672200
H	3.91145700	1.46292800	0.09538300
H	1.64641100	0.64801900	0.06707500
H	3.46315900	-0.94756900	1.93686700
C	5.49522800	0.92571200	1.47000800
H	5.23677100	1.67255400	2.22748300
H	5.87928100	0.04093000	1.98793000
H	6.30296200	1.33291000	0.85652900
H	-1.90206000	-0.53819100	1.59181000
C	-0.22336900	3.12949900	-0.80720400
C	0.90333900	1.55598700	-2.12356800
H	-0.67598300	3.50592400	-1.73634500
H	-0.02483100	3.97620500	-0.14803200
H	1.85870500	1.01626800	-2.14476700
H	0.78665700	2.05784700	-3.09740600
O	1.02944400	2.53179400	-1.09951100
Br	2.10083100	-1.66038900	-0.68334900

(*R*)-**TS1**$_P$-Br

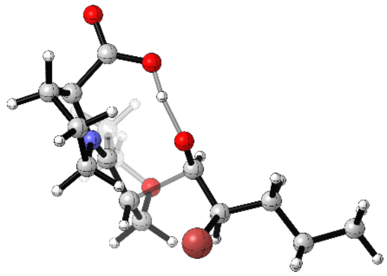

- Thermochemistry -

Zero-point correction=	0.384683 (Hartree/Particle)
Thermal correction to Energy=	0.402729
Thermal correction to Enthalpy=	0.403594
Thermal correction to Gibbs Free Energy=	0.339202
Sum of electronic and zero-point Energies=	-3512.761549
Sum of electronic and thermal Energies=	-3512.743502
Sum of electronic and thermal Enthalpies=	-3512.742637
Sum of electronic and thermal Free Energies=	-3512.807029

Number of Imaginary Frequencies = 1

E (Single Point Energy) [IEFPCM$_{(DCM)}$M06-2X/6-311++G(2d,2p)] = -3515.764676

C	-0.26041800	1.15309400	-1.26771500
H	0.15277600	0.40877400	-1.94194700
C	-1.51527600	0.94805900	-0.71454900
N	-2.06447100	-0.27279700	-0.63048100
C	-3.31705000	-0.57117500	0.06648600
H	-4.11474800	0.11254600	-0.23336900
C	-1.53093100	-1.44975700	-1.34105200

H	-0.45194400	-1.51466100	-1.21255100
H	-1.76871800	-1.36253600	-2.40983800
C	-2.26920200	-2.60996000	-0.68394900
H	-1.75640600	-2.89134900	0.24212000
H	-2.32214300	-3.48685500	-1.33089000
C	-3.64363500	-2.01172000	-0.37846500
H	-4.24899600	-1.97067400	-1.28837500
H	-4.20673900	-2.55123000	0.38450500
C	-2.19477600	2.08297400	0.01399500
H	-3.27777500	2.04343400	-0.13205900
H	-2.01018200	1.97245300	1.09184100
C	0.71020200	0.39240100	0.57699900
H	0.53156600	1.37388000	1.04706900
C	2.12845000	0.25527100	0.05199600
O	0.10522700	-0.62757200	0.98816600
C	-3.23904300	-0.47710100	1.59958200
O	-2.06782300	-0.48876400	2.19185200
O	-4.27888900	-0.42235300	2.23034800
C	3.07694600	0.11885900	1.24310700
H	2.84660300	0.94529100	1.93070100
C	4.55618000	0.18664500	0.87133200
H	4.81738600	-0.68060900	0.25567300
H	4.73170600	1.07738300	0.25535400
H	2.41464400	1.10009200	-0.57330100
H	2.84800800	-0.81366200	1.77093600
C	5.44300000	0.23072300	2.11366800
H	5.22475200	1.11578100	2.71973900
H	5.27972100	-0.65260600	2.73950700

H	6.50166100	0.25970400	1.84293900
H	-1.21177900	-0.53284900	1.60515800
C	-1.67083200	3.43441700	-0.46087500
C	0.24641900	2.56470800	-1.45509700
H	-2.04882600	3.65515900	-1.47007400
H	-1.99532300	4.22852300	0.21301900
H	1.33641600	2.60915700	-1.37658300
H	-0.01841400	2.94030300	-2.45672000
O	-0.25593700	3.44820900	-0.47003100
Br	2.28734900	-1.32104700	-1.09128400

(*R*)-**TS1**$_{P}$-Br-P

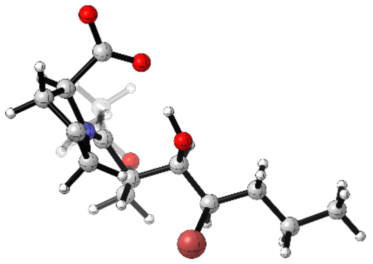

- Thermochemistry -

Zero-point correction=	0.389140 (Hartree/Particle)
Thermal correction to Energy=	0.407022
Thermal correction to Enthalpy=	0.407887
Thermal correction to Gibbs Free Energy=	0.343987
Sum of electronic and zero-point Energies=	-3512.790047
Sum of electronic and thermal Energies=	-3512.772164
Sum of electronic and thermal Enthalpies=	-3512.771299
Sum of electronic and thermal Free Energies=	-3512.835200

Number of Imaginary Frequencies = 0

E (Single Point Energy) [IEFPCM$_{(DCM)}$M06-2X/6-311++G(2d,2p)] = -3515.796502

C	-0.06786900	0.95761600	-0.90300000

H	0.12297800	0.22523600	-1.69044000
C	-1.50427200	0.90155900	-0.48031700
N	-2.17388600	-0.19350500	-0.59695000
C	-3.48552800	-0.43305300	0.04332200
H	-4.17369900	0.38001300	-0.19125600
C	-1.65538100	-1.44857900	-1.22203100
H	-0.62238300	-1.61172200	-0.91970600
H	-1.71932900	-1.32066500	-2.30730100
C	-2.61548400	-2.52224600	-0.71881700
H	-2.27019800	-2.90303800	0.24748000
H	-2.67920600	-3.35746000	-1.41712800
C	-3.93483300	-1.76733000	-0.55249600
H	-4.41054600	-1.60521300	-1.52470900
H	-4.64537200	-2.26086400	0.11039800
C	-2.05393100	2.11208000	0.21044200
H	-3.14380500	2.10975200	0.24467000
H	-1.69872100	2.06229000	1.24825200
C	0.77457100	0.57731100	0.36286200
H	0.77250700	1.45654900	1.02829800
C	2.24015600	0.32284700	0.02319000
O	0.28662500	-0.55810800	1.02158300
C	-3.32147300	-0.50157500	1.60538300
O	-2.17283600	-0.30236700	2.08366700
O	-4.37830900	-0.74015300	2.20759100
C	3.07104100	0.01481400	1.25888000
H	2.83643100	0.79769800	1.99477200
C	4.57740600	0.00344300	1.01195900
H	4.82882800	-0.81312600	0.32618200

H	4.87120700	0.93617000	0.51417600
H	2.66490000	1.16261500	-0.52852900
H	2.73817500	-0.93470900	1.68860900
C	5.35707100	-0.15611300	2.31534600
H	5.14883300	0.67178200	3.00072800
H	5.08040500	-1.08666100	2.82156100
H	6.43466700	-0.18015100	2.13303700
H	-0.59682400	-0.40236000	1.43985300
C	-1.53943600	3.40133900	-0.43980800
C	0.27833400	2.35273700	-1.44596300
H	-1.99784100	3.54419800	-1.42857700
H	-1.79235100	4.25525200	0.18887000
H	1.35662300	2.45595900	-1.57932800
H	-0.19666500	2.48842200	-2.42786900
O	-0.13131700	3.37539000	-0.56386200
Br	2.36437300	-1.19436100	-1.23425500

(*S*)-TS1$_P$-Br-Pre

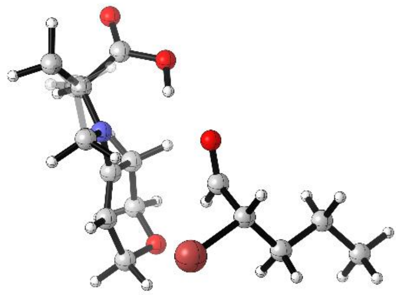

- Thermochemistry -

Zero-point correction= 0.384990 (Hartree/Particle)
Thermal correction to Energy= 0.404613
Thermal correction to Enthalpy= 0.405478
Thermal correction to Gibbs Free Energy= 0.336209

Sum of electronic and zero-point Energies= -3512.767685
Sum of electronic and thermal Energies= -3512.748062
Sum of electronic and thermal Enthalpies= -3512.747197
Sum of electronic and thermal Free Energies= -3512.816466

Number of Imaginary Frequencies = 0

E (Single Point Energy) [IEFPCM$_{(DCM)}$M06-2X/6-311++G(2d,2p)] = -3515.773376

C	0.47508000	-0.52059900	1.95928600
H	0.42906800	-1.60346700	1.91770500
C	1.34351400	0.17402800	1.20255300
N	2.13940500	-0.41512100	0.21736000
C	3.48530500	0.07693200	-0.07345600
H	3.98151500	0.50674400	0.80319400
C	2.03723500	-1.85318300	-0.03195100
H	1.02805300	-2.09656400	-0.38038300
H	2.23673900	-2.43153200	0.88445800
C	3.12898900	-2.09666000	-1.06676100
H	2.78296300	-1.78789900	-2.05918500
H	3.43493000	-3.14300800	-1.11726800
C	4.24659700	-1.17497200	-0.57007500
H	4.75775400	-1.63405800	0.28070500
H	4.99492800	-0.92736900	-1.32502900
C	1.38904400	1.68209500	1.28618300
H	2.42145300	2.04123700	1.37344200
H	0.98073900	2.11409200	0.36165400
C	-1.13581800	0.33111500	-0.78864100
H	-1.10191000	0.91697500	0.14949200
C	-2.41223200	-0.44240900	-1.01653800
O	-0.21072900	0.31928700	-1.57247100
C	3.49831900	1.16206800	-1.15368000

O	2.38342700	1.33209500	-1.85634400
O	4.49318000	1.81491400	-1.38618000
C	-3.63716500	0.38060100	-0.65910200
H	-4.52708600	-0.25278700	-0.73094300
C	-3.77912100	1.59307700	-1.58423500
H	-2.91104400	2.25439400	-1.46852100
H	-3.77923400	1.25456700	-2.62723300
H	-2.44152300	-0.84625000	-2.02882600
H	-3.55347500	0.70789500	0.38530700
C	-5.05943900	2.37277000	-1.29193600
H	-5.06934000	2.73188000	-0.25815200
H	-5.15181700	3.23890200	-1.95179300
H	-5.94000700	1.73950500	-1.43747600
H	1.65568600	0.76895100	-1.51065000
C	0.58838800	2.17115900	2.48730000
C	-0.55284500	0.16129400	2.82360200
H	1.12853300	1.95653200	3.42137700
H	0.40991300	3.24615700	2.42705300
H	-1.54004700	-0.28143900	2.65183500
H	-0.31691900	0.04397900	3.89404600
O	-0.68192600	1.54132400	2.51859800
Br	-2.24149800	-1.99361300	0.17313000

(*S*)-TS1$_{P}$-Br

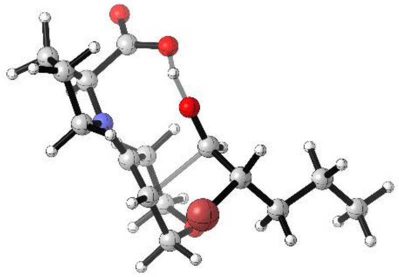

- Thermochemistry -

Zero-point correction=	0.385076 (Hartree/Particle)
Thermal correction to Energy=	0.402978
Thermal correction to Enthalpy=	0.403843
Thermal correction to Gibbs Free Energy=	0.339792
Sum of electronic and zero-point Energies=	-3512.752157
Sum of electronic and thermal Energies=	-3512.734254
Sum of electronic and thermal Enthalpies=	-3512.733389
Sum of electronic and thermal Free Energies=	-3512.797441

Number of Imaginary Frequencies = 1

E (Single Point Energy) [IEFPCM$_{(DCM)}$M06-2X/6-311++G(2d,2p)] = -3515.755215

C	-0.11217600	-0.09546500	1.40909700
H	-0.20143500	-1.16698400	1.53779100
C	1.13618800	0.43840700	1.11271300
N	2.11350000	-0.32165000	0.60589500
C	3.39271400	0.18898500	0.10997800
H	3.84136200	0.89303900	0.81428200
C	2.06055000	-1.79942300	0.58966600
H	1.10215200	-2.14271700	0.20233900
H	2.19589900	-2.16941500	1.61437700
C	3.23044200	-2.17643100	-0.31246200
H	2.91411200	-2.13867000	-1.36025100
H	3.60675700	-3.17837500	-0.10131000
C	4.25735100	-1.08012800	-0.02129900

H	4.75322700	-1.26980700	0.93490500
H	5.02253900	-0.96856100	-0.79076500
C	1.31369400	1.93657800	1.14297900
H	2.31762400	2.20438500	1.48390400
H	1.21264600	2.32038300	0.11727800
C	-0.64660000	0.03665200	-0.73599000
H	-0.63090700	1.13771200	-0.67856700
C	-2.07317100	-0.50994000	-0.79265000
O	0.25336600	-0.56417100	-1.38450100
C	3.30173900	0.93020500	-1.23487500
O	2.23266000	0.78300900	-1.97878000
O	4.24653800	1.61517100	-1.58336000
C	-3.13839800	0.54393000	-0.50815300
H	-4.07341500	0.05710000	-0.21523100
C	-3.38747300	1.41362800	-1.74569900
H	-2.43981200	1.83314900	-2.10645600
H	-3.77595000	0.78240400	-2.55383300
H	-2.18868100	-0.86857600	-1.81899100
H	-2.82625700	1.18588900	0.32143700
C	-4.36841300	2.54440100	-1.44618700
H	-3.97539600	3.20327800	-0.66542800
H	-4.55806400	3.15001700	-2.33611400
H	-5.32653000	2.14525600	-1.09796700
H	1.44471200	0.17635700	-1.63674300
C	0.28276700	2.60416400	2.05661500
C	-1.09091700	0.73498600	2.20170900
H	0.55835000	2.44532800	3.10936200
H	0.24906000	3.67764400	1.86585100

H	-2.11554500	0.39690600	2.03389600
H	-0.89340000	0.63954400	3.28205300
O	-1.02314500	2.10143600	1.83341700
Br	-2.33236200	-2.16687400	0.22160100

(*S*)-TS1$_P$-Br-P

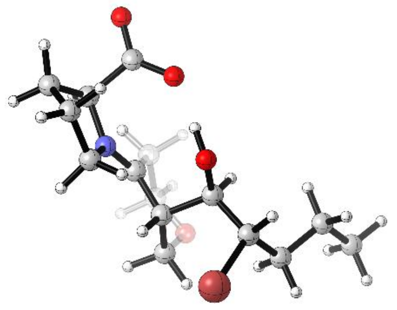

```
-------------------
- Thermochemistry -
-------------------
Zero-point correction=                           0.389442 (Hartree/Particle)
Thermal correction to Energy=                    0.407418
Thermal correction to Enthalpy=                  0.408283
Thermal correction to Gibbs Free Energy=         0.343838
Sum of electronic and zero-point Energies=       -3512.783672
Sum of electronic and thermal Energies=          -3512.765695
Sum of electronic and thermal Enthalpies=        -3512.764830
Sum of electronic and thermal Free Energies=     -3512.829276
Number of Imaginary Frequencies = 0
```

E (Single Point Energy) [IEFPCM$_{(DCM)}$M06-2X/6-311++G(2d,2p)] = -3515.791108

C	-0.17280200	-0.19360500	0.89572100
H	-0.13154100	-1.26955800	1.06757700
C	1.20400600	0.40000500	0.87499900
N	2.23815800	-0.32001100	0.60497700

C	3.55268700	0.24658000	0.23346200
H	3.87653800	0.98085600	0.97121200
C	2.23111800	-1.80207800	0.40282900
H	1.34545300	-2.09454100	-0.15662200
H	2.22387100	-2.26170400	1.39578700
C	3.53744700	-2.07041800	-0.33894500
H	3.38244600	-1.95769900	-1.41636800
H	3.90306000	-3.07945800	-0.14510100
C	4.46931500	-0.97564700	0.18248700
H	4.82762500	-1.22044400	1.18727900
H	5.32820800	-0.78417700	-0.46081100
C	1.28157300	1.88842700	1.04497000
H	2.28963700	2.22290500	1.29101000
H	1.02459000	2.33008900	0.07374300
C	-0.74372700	0.04996700	-0.54058500
H	-0.76598900	1.13536300	-0.71752700
C	-2.18565100	-0.42248500	-0.75724300
O	0.07168900	-0.58372300	-1.49575900
C	3.43817700	0.95789700	-1.16483400
O	2.31823100	0.93421100	-1.74217200
O	4.49667800	1.47617200	-1.54902200
C	-3.26513500	0.60404900	-0.42238900
H	-4.22472000	0.09015300	-0.31006300
C	-3.39641100	1.65797300	-1.52778400
H	-2.42813700	2.13616300	-1.71716700
H	-3.68811300	1.16207600	-2.46106500
H	-2.26049700	-0.69465900	-1.81094000
H	-3.05038500	1.10147300	0.52887300

C	-4.42606400	2.72426300	-1.15986400
H	-4.12878000	3.25424600	-0.24938800
H	-4.53407100	3.46120000	-1.95981100
H	-5.40723800	2.27286100	-0.98106300
H	0.89329300	-0.05373300	-1.63682900
C	0.29353900	2.38064200	2.11420700
C	-0.99063700	0.44530400	2.02470600
H	0.64982000	2.10272900	3.11606000
H	0.21211800	3.46641100	2.06156600
H	-2.02171400	0.09322900	1.99728600
H	-0.56219400	0.15151900	2.99401300
O	-1.00314000	1.85288800	1.90872600
Br	-2.52721900	-2.14533800	0.14168300

(*R*)-TS1$_G$-Br-Pre

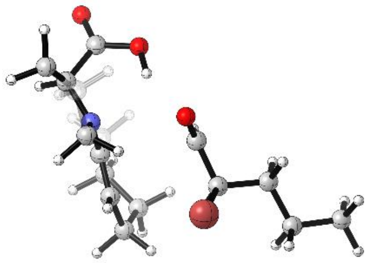

- Thermochemistry -

Zero-point correction= 0.408700 (Hartree/Particle)
Thermal correction to Energy= 0.428388
Thermal correction to Enthalpy= 0.429253
Thermal correction to Gibbs Free Energy= 0.360463
Sum of electronic and zero-point Energies= -3476.856317
Sum of electronic and thermal Energies= -3476.836629
Sum of electronic and thermal Enthalpies= -3476.835764
Sum of electronic and thermal Free Energies= -3476.904554
Number of Imaginary Frequencies = 0

E (Single Point Energy) [IEFPCM$_{(DCM)}$M06-2X/6-311++G(2d,2p)] = -3479.870718

C	-0.37093700	1.05413800	-1.61610700
H	-0.13909400	0.19171400	-2.23287200
C	-1.40742000	0.99533400	-0.74977800
N	-2.11186800	-0.18820000	-0.52519600
C	-3.45987100	-0.20908400	0.03087000
H	-4.08438800	0.62163200	-0.31088100
C	-1.84513600	-1.37438500	-1.33956700
H	-0.79626000	-1.66897600	-1.23974000
H	-2.05255800	-1.17324700	-2.40317200
C	-2.81206100	-2.40391700	-0.76753200
H	-2.39412900	-2.83935200	0.14688900
H	-3.02437500	-3.21490100	-1.46637600
C	-4.04839700	-1.55687600	-0.45389000
H	-4.61988200	-1.37529300	-1.36820400
H	-4.72018100	-2.00325800	0.28228600
C	-1.80379200	2.18256200	0.10396500
H	-2.89032200	2.31540100	0.08118900
H	-1.54877300	1.97236600	1.15405900
C	0.35095700	3.25656000	-0.64236600
C	0.72014000	-0.24423100	1.00879800
H	0.36365000	0.64200200	1.56935000
C	2.05272800	-0.03306900	0.33897200
O	0.10216700	-1.28565400	1.04111700
C	-3.46328600	-0.14917000	1.55963900
O	-2.36898400	-0.58358500	2.18333700
O	-4.42810100	0.23097100	2.18645100
C	3.16331300	-0.11771400	1.38867700

H	2.88135600	0.54733400	2.21733600
C	4.53539000	0.29681400	0.86249700
H	4.83533800	-0.38620300	0.06094900
H	4.46095200	1.29703300	0.41812500
H	2.05342900	0.94606700	-0.14343400
H	3.19332400	-1.13867600	1.78707000
C	5.58310400	0.29399600	1.97290700
H	5.67184100	-0.70071700	2.42124600
H	6.56563800	0.57831500	1.58782700
H	5.31453500	0.99778000	2.76708200
H	-1.66343800	-0.83949000	1.54832900
C	-1.13061200	3.48032800	-0.34931200
C	0.50134000	2.26979300	-1.79957300
H	-1.62197200	3.84241600	-1.26122900
H	-1.26960200	4.24737400	0.41807900
H	1.54805600	1.95294300	-1.91003300
H	0.25159600	2.77547000	-2.74335500
H	0.83724900	2.85572100	0.25940400
H	0.84805700	4.20228300	-0.87848600
Br	2.31284600	-1.33339000	-1.07604300

(*R*)-TS1$_{G}$-Br

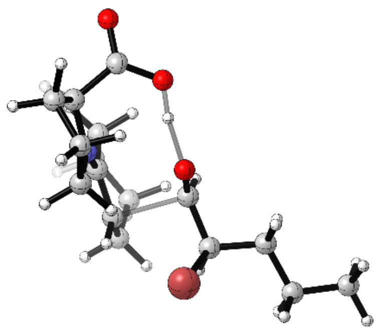

- Thermochemistry -

Zero-point correction=	0.408659 (Hartree/Particle)
Thermal correction to Energy=	0.426827
Thermal correction to Enthalpy=	0.427692
Thermal correction to Gibbs Free Energy=	0.363361
Sum of electronic and zero-point Energies=	-3476.848987
Sum of electronic and thermal Energies=	-3476.830819
Sum of electronic and thermal Enthalpies=	-3476.829954
Sum of electronic and thermal Free Energies=	-3476.894285

Number of Imaginary Frequencies = 1

E (Single Point Energy) [IEFPCM$_{(DCM)}$M06-2X/6-311++G(2d,2p)] = -3479.861538

C	-0.26856900	1.17811400	-1.23258300
H	0.13560100	0.41556100	-1.89157800
C	-1.51904800	0.95688300	-0.67546800
N	-2.05600700	-0.27818600	-0.62625100
C	-3.30732300	-0.61014300	0.05652400
H	-4.11601200	0.06537500	-0.23148600
C	-1.51677800	-1.42798700	-1.37617100
H	-0.43695200	-1.49182100	-1.25942000
H	-1.76381400	-1.31217400	-2.44061600
C	-2.23528600	-2.61593500	-0.74835500
H	-1.71212600	-2.91453500	0.16655600
H	-2.27999800	-3.47572100	-1.41870500
C	-3.61513500	-2.04555800	-0.41847200
H	-4.22833400	-1.99274300	-1.32253500
H	-4.16482600	-2.61157500	0.33532600
C	-2.23344100	2.06042000	0.07306300
H	-3.31522700	1.93738100	-0.01933200
H	-2.00162500	1.95246100	1.14412000
C	-0.31390600	3.59594700	-0.46504200

C	0.70105400	0.33102400	0.61196500
H	0.50458100	1.27663900	1.14340000
C	2.12229400	0.25397800	0.08673900
O	0.10721600	-0.71708100	0.95907400
C	-3.23513700	-0.54595800	1.59021000
O	-2.06366900	-0.59173100	2.18301900
O	-4.27325100	-0.48627000	2.22361600
C	3.06943300	0.06180600	1.27130400
H	2.82693400	0.84513600	2.00386600
C	4.54879700	0.16819900	0.90918700
H	4.82302000	-0.66259300	0.25055300
H	4.71415600	1.09152500	0.34006900
H	2.39069700	1.14177700	-0.48429000
H	2.85105500	-0.90179200	1.74515700
C	5.43162200	0.15959400	2.15511600
H	5.27709000	-0.75622500	2.73480200
H	6.49062600	0.21451400	1.88978600
H	5.20147100	1.01007000	2.80481600
H	-1.21559700	-0.63356600	1.58930100
C	-1.83115600	3.45667800	-0.40464200
C	0.26111200	2.57933300	-1.45034700
H	-2.25098600	3.63185200	-1.40304000
H	-2.26901200	4.20227900	0.26494400
H	1.35605800	2.58304200	-1.40024700
H	0.01956000	2.89470300	-2.47510300
H	0.10662700	3.43636800	0.53648500
H	-0.03195000	4.60863600	-0.76841200
Br	2.31335600	-1.24528500	-1.15070000

(*R*)-TS1$_G$-Br-P

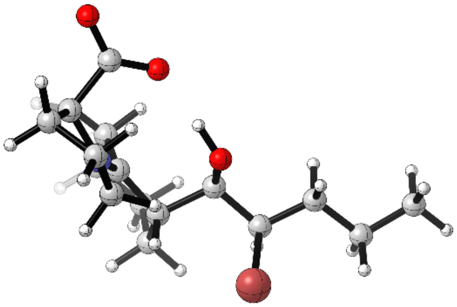

- Thermochemistry -

Zero-point correction=	0.413168 (Hartree/Particle)
Thermal correction to Energy=	0.431291
Thermal correction to Enthalpy=	0.432156
Thermal correction to Gibbs Free Energy=	0.367485
Sum of electronic and zero-point Energies=	-3476.879029
Sum of electronic and thermal Energies=	-3476.860906
Sum of electronic and thermal Enthalpies=	-3476.860041
Sum of electronic and thermal Free Energies=	-3476.924713

Number of Imaginary Frequencies = 0

E (Single Point Energy) [IEFPCM$_{(DCM)}$M06-2X/6-311++G(2d,2p)] = -3479.895319

C	-0.06913900	0.95080700	-0.79336200
H	0.07693500	0.25904300	-1.62310100
C	-1.50663200	0.92681200	-0.36279600
N	-2.23800800	-0.10623000	-0.61635400
C	-3.54720100	-0.36679900	0.02170300
H	-4.19628100	0.50408300	-0.07211800
C	-1.80524400	-1.29192600	-1.42106400
H	-0.78020800	-1.55575200	-1.17011000
H	-1.87982700	-1.01035200	-2.47609600
C	-2.81644800	-2.37139100	-1.04717900
H	-2.47713100	-2.90310000	-0.15269800
H	-2.94095400	-3.09526400	-1.85344500

C	-4.08579000	-1.57617300	-0.74227200
H	-4.56884000	-1.25156400	-1.66926400
H	-4.81249300	-2.11823500	-0.13706800
C	-1.99176100	2.05911900	0.49405900
H	-3.07449000	2.03880300	0.61311900
H	-1.56811800	1.87959700	1.49252800
C	-0.01609700	3.45317900	-0.26537500
C	0.73202700	0.38864600	0.42988300
H	0.65471800	1.11198900	1.25821800
C	2.22702100	0.27564200	0.15180700
O	0.25653800	-0.86724200	0.83368800
C	-3.34448700	-0.67236900	1.54881000
O	-2.16831800	-0.64333400	2.00151000
O	-4.39897100	-0.90977200	2.15688200
C	2.99053400	-0.31959300	1.32374700
H	2.66359600	0.22935700	2.21915500
C	4.50799100	-0.20863600	1.19820200
H	4.84740800	-0.80498500	0.34416600
H	4.77995200	0.83268400	0.98386100
H	2.63943900	1.24873600	-0.11858000
H	2.68510300	-1.36080700	1.46323600
C	5.21222600	-0.67725100	2.46961500
H	4.95704600	-1.71817000	2.69333100
H	6.29879300	-0.61175500	2.36877300
H	4.91545500	-0.06829400	3.32964800
H	-0.61766900	-0.78215000	1.28962300
C	-1.52442000	3.42625400	-0.03008400
C	0.37185100	2.35186400	-1.25019000

H	-2.04449400	3.65270800	-0.96863100
H	-1.82282400	4.18856200	0.69400600
H	1.44768900	2.34485400	-1.44652400
H	-0.10877800	2.55501000	-2.21461600
H	0.51252500	3.32910000	0.68799100
H	0.28216100	4.42590500	-0.66672300
Br	2.52219300	-0.83722100	-1.45268200

(*S*)-TS1$_G$-Br-Pre

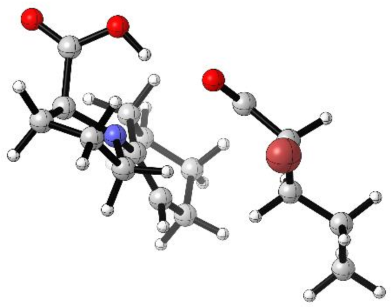

- Thermochemistry -

Zero-point correction=	0.408539 (Hartree/Particle)
Thermal correction to Energy=	0.428230
Thermal correction to Enthalpy=	0.429095
Thermal correction to Gibbs Free Energy=	0.359689
Sum of electronic and zero-point Energies=	-3476.854839
Sum of electronic and thermal Energies=	-3476.835147
Sum of electronic and thermal Enthalpies=	-3476.834282
Sum of electronic and thermal Free Energies=	-3476.903689

Number of Imaginary Frequencies = 0

E (Single Point Energy) [IEFPCM$_{(DCM)}$M06-2X/6-311++G(2d,2p)] = -3479.870083

C	-0.20317200	1.72486600	-1.17741000
H	0.24264800	1.11926300	-1.96135500
C	-1.27279100	1.25349200	-0.50388900
N	-1.75730900	-0.05030200	-0.67879400

C	-3.19593300	-0.32148900	-0.65849600
H	-3.79168500	0.51092800	-1.04818900
C	-1.07162400	-0.96268400	-1.59266900
H	-0.05263000	-1.15012100	-1.23820300
H	-1.01546900	-0.54556200	-2.61129600
C	-1.96255500	-2.19716900	-1.56600600
H	-1.77990200	-2.76819200	-0.64886900
H	-1.79936000	-2.85425600	-2.42209100
C	-3.36135800	-1.57411800	-1.55505500
H	-3.63215200	-1.24914700	-2.56340700
H	-4.14652500	-2.24008900	-1.19155400
C	-1.96037600	2.06360300	0.57490700
H	-3.04891400	1.99308000	0.46734500
H	-1.72524600	1.61384100	1.55158000
C	0.81371500	-0.27864000	1.45201400
H	0.62643400	0.73050000	1.86521600
C	2.24189500	-0.49122500	1.00888000
O	-0.06173400	-1.11184100	1.41976000
C	-3.72804300	-0.61109000	0.74531500
O	-2.83850500	-0.95800800	1.66960200
O	-4.91329200	-0.57112800	0.99895600
C	2.76003000	0.65709600	0.15144900
H	2.25355600	0.63940400	-0.81955900
C	4.27492700	0.65902200	-0.03467400
H	4.75850200	0.56935500	0.94595100
H	4.57117200	-0.21994400	-0.61678500
H	2.83565300	-0.56907300	1.92780900
H	2.45810900	1.58944800	0.65146200

C	4.74393200	1.93445900	-0.73076600
H	4.48542400	2.81939500	-0.14002800
H	5.82695600	1.93042500	-0.87733200
H	4.27194200	2.03777100	-1.71354300
H	-1.92099100	-0.89974900	1.32102800
C	-1.54261300	3.53512900	0.56089500
C	0.40875600	3.08171000	-0.93174900
H	-2.03017900	4.04317600	-0.28092800
H	-1.89220400	4.02112300	1.47652200
H	1.50255100	3.01044800	-0.98041200
H	0.12033300	3.76839600	-1.74065800
C	-0.02911400	3.66844100	0.40927300
H	0.27818600	4.71602700	0.48544500
H	0.46605000	3.12794700	1.22913300
Br	2.44151700	-2.20322500	0.11616000

(*S*)-TS1$_G$-Br

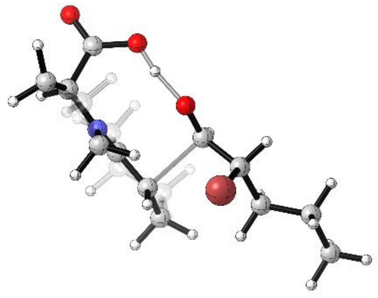

- Thermochemistry -

Zero-point correction=	0.408901 (Hartree/Particle)
Thermal correction to Energy=	0.426949
Thermal correction to Enthalpy=	0.427814
Thermal correction to Gibbs Free Energy=	0.363834
Sum of electronic and zero-point Energies=	-3476.842825
Sum of electronic and thermal Energies=	-3476.824777

Sum of electronic and thermal Enthalpies= -3476.823912
Sum of electronic and thermal Free Energies= -3476.887892

Number of Imaginary Frequencies = 1

E (Single Point Energy) [IEFPCM$_{(DCM)}$M06-2X/6-311++G(2d,2p)] = -3479.855148

C	0.12950200	0.96109300	-1.06832100
H	0.48893500	0.09613700	-1.61979600
C	-1.22164800	0.99513400	-0.72896600
N	-1.97328500	-0.11796400	-0.72126600
C	-3.36885300	-0.17827400	-0.27575200
H	-3.97108000	0.60221200	-0.74593200
C	-1.53198700	-1.39827500	-1.30614000
H	-0.52008600	-1.63591900	-0.98726500
H	-1.56278900	-1.32097400	-2.40159600
C	-2.55550000	-2.39342900	-0.77466900
H	-2.26887400	-2.70591500	0.23523500
H	-2.63470300	-3.28153000	-1.40358700
C	-3.84348200	-1.57060600	-0.74056200
H	-4.26167200	-1.48254200	-1.74728900
H	-4.61534400	-1.97301700	-0.08275800
C	-1.83207600	2.25021400	-0.14296000
H	-2.90022500	2.29501000	-0.36635300
H	-1.74960200	2.17610200	0.95270700
C	0.62614100	0.10614800	0.95182500
H	0.60644500	1.12448600	1.37129800
C	2.04403500	-0.42590500	0.77804900
O	-0.27018100	-0.72001200	1.25920300
C	-3.57251200	-0.00886800	1.23848600
O	-2.55213000	-0.16257800	2.04820900

O	-4.69227400	0.23695300	1.64972100
C	3.14026400	0.56513500	0.42409500
H	3.20183600	0.68137600	-0.66201400
C	4.50682100	0.15341000	0.97127200
H	4.44854100	0.07907600	2.06388300
H	4.75744300	-0.84681800	0.59813600
H	2.26145800	-0.86977100	1.75701600
H	2.86318800	1.54359300	0.84220700
C	5.59931000	1.14243900	0.57382000
H	5.37458000	2.14596100	0.94976600
H	6.57052700	0.84239500	0.97555600
H	5.68994500	1.20470500	-0.51540500
H	-1.61957800	-0.36407100	1.63068200
C	-1.15273600	3.53335100	-0.62254800
C	0.86497200	2.26143000	-1.34521200
H	-1.41570800	3.71159500	-1.67264800
H	-1.54105800	4.37735500	-0.04542000
H	1.93898100	2.13381700	-1.21126600
H	0.72935000	2.51760800	-2.40633800
C	0.36237100	3.42664100	-0.49660500
H	0.84124100	4.35614600	-0.81934100
H	0.63404600	3.27777900	0.55728700
Br	2.09760400	-1.97747900	-0.41342400

(S)-TS1$_G$-Br-P

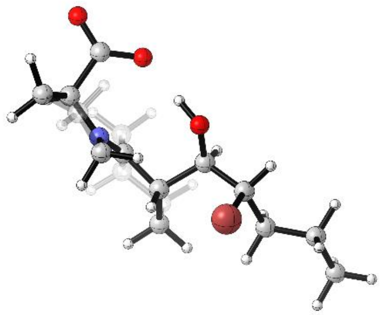

- Thermochemistry -

Zero-point correction=	0.413268 (Hartree/Particle)
Thermal correction to Energy=	0.431325
Thermal correction to Enthalpy=	0.432190
Thermal correction to Gibbs Free Energy=	0.367929
Sum of electronic and zero-point Energies=	-3476.874439
Sum of electronic and thermal Energies=	-3476.856382
Sum of electronic and thermal Enthalpies=	-3476.855517
Sum of electronic and thermal Free Energies=	-3476.919777

Number of Imaginary Frequencies = 0

E (Single Point Energy) [IEFPCM$_{(DCM)}$M06-2X/6-311++G(2d,2p)] = -3479.890591

C	0.16802000	0.63928600	-0.72811500
H	0.30319000	-0.24269400	-1.35593600
C	-1.30142800	0.89080400	-0.52872300
N	-2.15079400	-0.06799200	-0.68361700
C	-3.56974800	0.00782400	-0.26856200
H	-4.03493300	0.90267900	-0.68305500
C	-1.80585000	-1.45879700	-1.11209800
H	-0.88929200	-1.77804800	-0.61907600
H	-1.66814600	-1.44162400	-2.19773300
C	-3.02869000	-2.27538300	-0.70525400
H	-2.92454900	-2.60513500	0.33312600
H	-3.14674600	-3.15587200	-1.33794100

C	-4.17817200	-1.27587400	-0.83293200
H	-4.45054000	-1.13455200	-1.88359700
H	-5.07035700	-1.55413400	-0.27208300
C	-1.70702800	2.24074400	-0.01336300
H	-2.78842200	2.36935700	-0.02024600
H	-1.40295700	2.25634500	1.04328900
C	0.73426600	0.29070900	0.68997700
H	0.81407200	1.21907200	1.27817400
C	2.14264900	-0.30846400	0.69041400
O	-0.07578300	-0.64624000	1.35585600
C	-3.67293600	0.07633600	1.29679600
O	-2.59808300	0.09598600	1.95393900
O	-4.83786700	0.11360800	1.72130800
C	3.31698800	0.59631700	0.33539000
H	3.45955400	0.63763100	-0.74871300
C	4.62051400	0.14444800	0.99451700
H	4.49146000	0.14702300	2.08338600
H	4.83033800	-0.89126600	0.70178100
H	2.28000400	-0.70383400	1.69917000
H	3.07813500	1.61608100	0.66983300
C	5.79343900	1.04062400	0.60615400
H	5.60855000	2.07814300	0.90327800
H	6.71852100	0.71281100	1.08719800
H	5.95293700	1.02508300	-0.47685100
H	-0.97380000	-0.29643900	1.57992700
C	-1.01013000	3.38560500	-0.76368600
C	0.82985600	1.83763000	-1.43538700
H	-1.39481600	3.43736800	-1.78932700

H	-1.27415200	4.32585600	-0.27305700
H	1.90624300	1.67844600	-1.49439100
H	0.46763500	1.84670800	-2.47080200
C	0.50185200	3.18417400	-0.79397100
H	0.97715800	3.98582800	-1.36655400
H	0.90546400	3.24142000	0.22486800
Br	2.18209300	-1.93723000	-0.41900300

(*R*)-TS1$_{O}$-Br-Pre

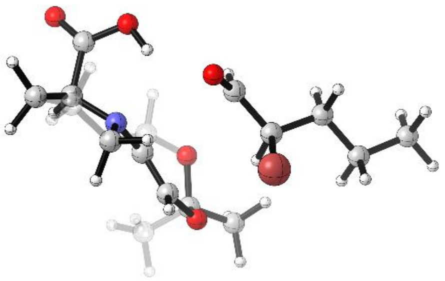

- Thermochemistry -

Zero-point correction= 0.416339 (Hartree/Particle)
Thermal correction to Energy= 0.437978
Thermal correction to Enthalpy= 0.438843
Thermal correction to Gibbs Free Energy= 0.366152
Sum of electronic and zero-point Energies= -3627.237810
Sum of electronic and thermal Energies= -3627.216171
Sum of electronic and thermal Enthalpies= -3627.215306
Sum of electronic and thermal Free Energies= -3627.287997

Number of Imaginary Frequencies = 0

E (Single Point Energy) [IEFPCM$_{(DCM)}$M06-2X/6-311++G(2d,2p)] = -3630.305534

C	-0.26603000	0.92528900	-1.36649000
H	-0.18480700	0.27856000	-2.22965100
C	-1.21355000	0.81405300	-0.41975700
N	-2.18955500	-0.18852300	-0.42804600

C	-3.51209100	0.07187200	0.14180600
H	-3.77981300	1.13377800	0.14321100
C	-2.27889800	-1.07134800	-1.59287700
H	-1.39325600	-1.71393100	-1.63643600
H	-2.33346900	-0.48377800	-2.52403200
C	-3.58049700	-1.82663400	-1.34982200
H	-3.41862700	-2.63688300	-0.63073600
H	-3.99084500	-2.25646500	-2.26494200
C	-4.47888800	-0.73838000	-0.75279000
H	-4.85130600	-0.08437600	-1.54598200
H	-5.33490500	-1.12007600	-0.19333300
C	-1.16059300	1.73298700	0.77601300
H	-1.93423200	2.51295900	0.73205700
H	-1.31175600	1.17340000	1.70556200
O	0.77158800	1.82529300	-1.29684000
O	0.13001900	2.31441300	0.87650200
C	0.59801400	2.87291700	-0.34018100
C	1.97474700	3.42904300	-0.05138700
H	2.44184300	3.75905300	-0.98124000
H	1.89389200	4.27717300	0.63159300
C	-0.35421100	3.92425600	-0.89922600
H	0.12850400	4.43232000	-1.73656700
H	-0.58686100	4.65834600	-0.12362100
H	-1.28195900	3.47630600	-1.26225500
C	0.59719900	-1.06628800	1.10937700
H	0.38971000	-0.43238900	1.99493400
C	1.88195600	-0.69191100	0.42248100
O	-0.15686900	-1.96008800	0.79816200

C	-3.61021600	-0.40795500	1.59360300
O	-2.65537800	-1.23382200	2.01749900
O	-4.52626700	-0.08210800	2.31580400
H	2.59490100	2.65650400	0.40967600
C	3.08111200	-0.91052900	1.34530500
H	2.84456100	-0.42477900	2.30224400
C	4.38458700	-0.33342600	0.79818100
H	4.61742900	-0.81735600	-0.15616900
H	4.24472700	0.73384100	0.58666700
H	3.19230500	-1.98338900	1.54341600
C	5.53975900	-0.52571500	1.77745400
H	5.33544200	-0.02478500	2.72913700
H	5.70033100	-1.58850500	1.98496100
H	6.46987900	-0.11750000	1.37397000
H	-1.98587800	-1.38198200	1.31285500
H	1.80726500	0.36827500	0.15680600
Br	2.05527600	-1.63353000	-1.26185600

(*R*)-TS1$_{O}$-Br

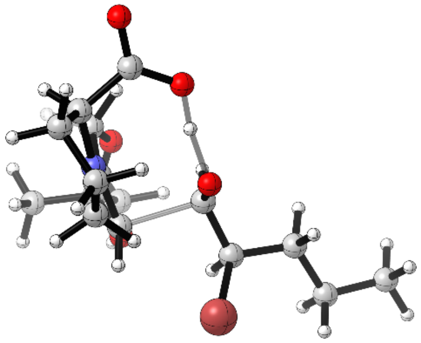

- Thermochemistry -

Zero-point correction= 0.415312 (Hartree/Particle)
Thermal correction to Energy= 0.435479

Thermal correction to Enthalpy= 0.436344
Thermal correction to Gibbs Free Energy= 0.367844
Sum of electronic and zero-point Energies= -3627.226893
Sum of electronic and thermal Energies= -3627.206726
Sum of electronic and thermal Enthalpies= -3627.205861
Sum of electronic and thermal Free Energies= -3627.274362

Number of Imaginary Frequencies = 1

E (Single Point Energy) [IEFPCM$_{(DCM)}$M06-2X/6-311++G(2d,2p)] = -3630.294358

C	-0.16823300	0.95889600	-0.89261600
H	0.18662200	0.38230700	-1.73912500
C	-1.43138900	0.75184700	-0.35868300
N	-2.09157100	-0.39170100	-0.55219600
C	-3.33929700	-0.74165100	0.12781200
H	-4.03000400	0.10634100	0.14474000
C	-1.70937500	-1.38160900	-1.57963400
H	-0.65078500	-1.63007200	-1.49379000
H	-1.90843300	-0.95643900	-2.57229400
C	-2.62225800	-2.56357900	-1.26659600
H	-2.16443600	-3.18714700	-0.49141500
H	-2.80624100	-3.18388500	-2.14469500
C	-3.89091100	-1.89186900	-0.73388200
H	-4.47288400	-1.47124500	-1.55891600
H	-4.53619800	-2.55262500	-0.15408300
C	-1.97093200	1.76163400	0.62314400
H	-2.89261100	2.21297800	0.22788500
H	-2.20987800	1.28282000	1.57859800
O	0.47780000	2.17327600	-0.77292100
O	-0.99614300	2.74045500	0.91593800
C	-0.29891900	3.23415500	-0.21489300
C	0.68500400	4.25929000	0.30196500

H	1.33116100	4.58950800	-0.51377800
H	0.14605300	5.11909800	0.70485500
C	-1.23922300	3.79904000	-1.27407900
H	-0.64616700	4.29016500	-2.04800500
H	-1.90880600	4.53260900	-0.81799200
H	-1.83785400	3.01913200	-1.75127100
C	0.67677400	-0.19923200	0.65748300
H	0.49112800	0.60141600	1.39555100
C	2.10562100	-0.14482500	0.14733200
O	0.10246900	-1.32084800	0.78541100
C	-3.17320900	-1.18409600	1.59608600
O	-1.97732700	-1.39826000	2.07795800
O	-4.18374700	-1.32123200	2.26354600
H	1.29519800	3.81173000	1.08956700
C	3.05231400	-0.60152400	1.25459400
H	2.81254000	-0.00006800	2.14325600
C	4.53137500	-0.42322900	0.92018800
H	4.79596200	-1.08033500	0.08505300
H	4.70199800	0.60559700	0.57947100
H	2.83230100	-1.64607100	1.50276600
C	5.41968400	-0.72788700	2.12421700
H	5.19819600	-0.05167500	2.95613300
H	5.26115400	-1.75296400	2.47462700
H	6.47791900	-0.61860700	1.87287500
H	-1.12331400	-1.33178200	1.44487600
H	2.35415000	0.85984500	-0.19676900
Br	2.30850400	-1.30365800	-1.41733800

(*R*)-TS1$_O$-Br-P

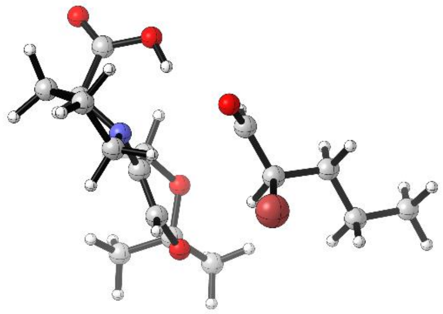

- Thermochemistry -

Zero-point correction=	0.416333 (Hartree/Particle)
Thermal correction to Energy=	0.437974
Thermal correction to Enthalpy=	0.438840
Thermal correction to Gibbs Free Energy=	0.366131
Sum of electronic and zero-point Energies=	-3627.237815
Sum of electronic and thermal Energies=	-3627.216174
Sum of electronic and thermal Enthalpies=	-3627.215309
Sum of electronic and thermal Free Energies=	-3627.288017

Number of Imaginary Frequencies = 0

E (Single Point Energy) [IEFPCM$_{(DCM)}$M06-2X/6-311++G(2d,2p)] = -3630.321486

C	-0.26631100	0.92433700	-1.36663700
H	-0.18509600	0.27734000	-2.22960400
C	-1.21395700	0.81350400	-0.41998300
N	-2.19017300	-0.18886100	-0.42832300
C	-3.51249300	0.07169800	0.14191000
H	-3.78030400	1.13359200	0.14293600
C	-2.27983400	-1.07157400	-1.59316500
H	-1.39417500	-1.71411300	-1.63704200
H	-2.33470700	-0.48396200	-2.52427800
C	-3.58127400	-1.82699500	-1.34963900
H	-3.41898500	-2.63732400	-0.63074100
H	-3.99199700	-2.25674700	-2.26462700

C	-4.47956300	-0.73894700	-0.75205900
H	-4.85269400	-0.08509800	-1.54503900
H	-5.33511700	-1.12089100	-0.19206300
C	-1.16101300	1.73277600	0.77552100
H	-1.93455200	2.51282700	0.73126300
H	-1.31231500	1.17349300	1.70522700
O	0.77143400	1.82422000	-1.29717000
O	0.12966600	2.31407900	0.87594600
C	0.59782600	2.87217600	-0.34086700
C	1.97453900	3.42837000	-0.05209200
H	2.44183000	3.75787700	-0.98202400
H	1.89358700	4.27685700	0.63043300
C	-0.35427200	3.92340200	-0.90034400
H	0.12859700	4.43122400	-1.73774300
H	-0.58700200	4.65771000	-0.12496800
H	-1.28198600	3.47540200	-1.26339700
C	0.59762500	-1.06463500	1.10952500
H	0.39006000	-0.42986700	1.99443400
C	1.88243600	-0.69103000	0.42234900
O	-0.15649500	-1.95864700	0.79906900
C	-3.60995400	-0.40740900	1.59400000
O	-2.65470200	-1.23279700	2.01789400
O	-4.52571800	-0.08132500	2.31645600
H	2.59459800	2.65607800	0.40951500
C	3.08153900	-0.90930100	1.34533300
H	2.84516900	-0.42266700	2.30186900
C	4.38520700	-0.33311300	0.79770200
H	4.61790800	-0.81800700	-0.15619200

H	4.24568000	0.73399700	0.58517900
H	3.19235800	-1.98203500	1.54433700
C	5.54030300	-0.52484400	1.77717400
H	5.33613800	-0.02294000	2.72837600
H	5.70052800	-1.58748800	1.98569200
H	6.47056100	-0.11731600	1.37331100
H	-1.98578900	-1.38142700	1.31281500
H	1.80800300	0.36895600	0.15586400
Br	2.05550400	-1.63394800	-1.26129200

(*S*)-TS1$_{O}$-Br-Pre

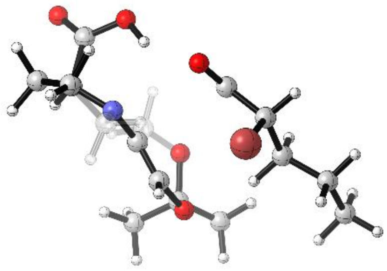

- Thermochemistry -

Zero-point correction=	0.416308 (Hartree/Particle)
Thermal correction to Energy=	0.437945
Thermal correction to Enthalpy=	0.438810
Thermal correction to Gibbs Free Energy=	0.366172
Sum of electronic and zero-point Energies=	-3627.235237
Sum of electronic and thermal Energies=	-3627.213599
Sum of electronic and thermal Enthalpies=	-3627.212734
Sum of electronic and thermal Free Energies=	-3627.285373

Number of Imaginary Frequencies = 0

E (Single Point Energy) [IEFPCM$_{(DCM)}$M06-2X/6-311++G(2d,2p)] = -3630.304491

C	0.08543300	0.85898000	-1.23927800
H	0.31327000	0.09356000	-1.97070000

C	-1.00915200	0.86297300	-0.45653100
N	-1.98368200	-0.13869900	-0.48366800
C	-3.38425100	0.19991800	-0.22654200
H	-3.62817200	1.23928400	-0.47181300
C	-1.87191500	-1.20007300	-1.48483500
H	-0.99754400	-1.82127500	-1.26734100
H	-1.75993600	-0.77452000	-2.49627300
C	-3.20144100	-1.93206800	-1.35108000
H	-3.17756300	-2.60014600	-0.48322500
H	-3.44501800	-2.52345100	-2.23520500
C	-4.18131600	-0.77634300	-1.12454600
H	-4.39774800	-0.27935500	-2.07417500
H	-5.12841000	-1.07291900	-0.67006800
C	-1.15238600	1.94276800	0.58671800
H	-1.93434700	2.67069400	0.32567700
H	-1.41841000	1.50425900	1.55497700
O	1.06263300	1.82370300	-1.17720200
O	0.09150800	2.59313000	0.78539800
C	0.72754400	2.98630700	-0.41659800
C	2.03677000	3.63032800	-0.01544100
H	2.63530100	3.83341300	-0.90595200
H	2.58812900	2.95584200	0.64406500
C	-0.14989100	3.90738900	-1.25838000
H	-0.50747900	4.73729300	-0.64334900
H	-1.00689000	3.37516700	-1.67772400
H	0.44088300	4.30642300	-2.08549500
C	0.53018200	-1.04070100	1.44078600
H	0.45023400	-0.08707300	1.99746400

C	1.93378000	-1.38085500	0.98559600
O	-0.43201900	-1.76058800	1.29385200
C	-3.77057500	-0.00516200	1.24135000
O	-2.94537900	-0.73404000	1.98681300
O	-4.80028400	0.44299300	1.69721100
H	1.84226200	4.56839100	0.50879900
C	2.82356300	-0.15855200	0.83246000
H	2.45156800	0.47157000	0.01757200
C	4.29996500	-0.48798600	0.62886500
H	4.62730000	-1.18369100	1.41207000
H	4.42869100	-1.00634300	-0.32719100
H	2.71250700	0.42308200	1.75998800
C	5.16158200	0.77213900	0.65770000
H	5.06631500	1.29033200	1.61761500
H	6.21747200	0.53398400	0.50573500
H	4.85663300	1.46824700	-0.13088900
H	-2.14383700	-1.00250800	1.48103100
H	2.34281400	-2.05336300	1.74980700
Br	1.86796300	-2.45977000	-0.63542200

(*S*)-TS1$_O$-Br

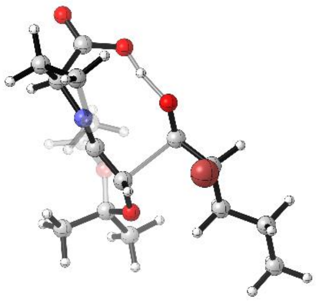

- Thermochemistry -

Zero-point correction= 0.415169 (Hartree/Particle)
Thermal correction to Energy= 0.435309
Thermal correction to Enthalpy= 0.436174
Thermal correction to Gibbs Free Energy= 0.367855
Sum of electronic and zero-point Energies= -3627.223513
Sum of electronic and thermal Energies= -3627.203372
Sum of electronic and thermal Enthalpies= -3627.202507
Sum of electronic and thermal Free Energies= -3627.270827

Number of Imaginary Frequencies = 1

E (Single Point Energy) [IEFPCM$_{(DCM)}$M06-2X/6-311++G(2d,2p)] = -3630.290158

C	0.21606100	0.68827800	-0.75369000
H	0.54808900	-0.07475000	-1.45123700
C	-1.13182500	0.85673100	-0.45831600
N	-2.02213000	-0.11993900	-0.63298200
C	-3.41876500	-0.04091300	-0.19764600
H	-3.85077300	0.93417500	-0.44126100
C	-1.75192100	-1.32547700	-1.44049100
H	-0.82124800	-1.79508800	-1.12287400
H	-1.67397900	-1.03249200	-2.49615900
C	-2.97816400	-2.19300900	-1.17573600
H	-2.83395100	-2.76268000	-0.25158300
H	-3.16760900	-2.89485300	-1.98888400
C	-4.09828000	-1.16402600	-1.00230100
H	-4.40516700	-0.76981700	-1.97516600
H	-4.98055400	-1.54608300	-0.48782300
C	-1.56124400	2.10861000	0.26704900
H	-2.28817600	2.66365500	-0.34393600
H	-2.04160700	1.85499200	1.21797700
O	1.09039300	1.75807400	-0.68510200
O	-0.44671900	2.90739900	0.59853400

C	0.50386400	3.03442000	-0.44433800
C	1.61104000	3.91651700	0.08815800
H	2.43363300	3.94527500	-0.62919000
H	1.97192700	3.51070300	1.03587700
C	-0.11602900	3.57537800	-1.72925200
H	-0.67108900	4.49181900	-1.51289500
H	-0.79002800	2.85386000	-2.19772200
H	0.68121200	3.80153600	-2.44001600
C	0.51539100	-0.41544400	1.04136000
H	0.56023300	0.52769500	1.61221400
C	1.90382200	-1.00058400	0.79733500
O	-0.42997800	-1.23527800	1.23699300
C	-3.63968200	-0.23450600	1.31637600
O	-2.64210500	-0.60565400	2.07454700
O	-4.75751700	-0.02904500	1.75587700
H	1.23549700	4.92957100	0.24628800
C	3.04699600	-0.03329100	0.54391900
H	3.01715600	0.33049600	-0.48711900
C	4.41795100	-0.62946100	0.85754500
H	4.42469200	-0.99864700	1.89063500
H	4.59692700	-1.49625400	0.21086800
H	2.87802600	0.84359500	1.18398700
C	5.53164700	0.39770100	0.66758000
H	5.38310900	1.26082500	1.32476000
H	6.51142500	-0.03215100	0.89177600
H	5.55218000	0.76249400	-0.36454000
H	-1.70031600	-0.82687100	1.63534800
H	2.10386100	-1.57301700	1.71086800

Br	1.84753400	-2.39079800	-0.58432700

(*S*)-TS1$_O$-Br-P

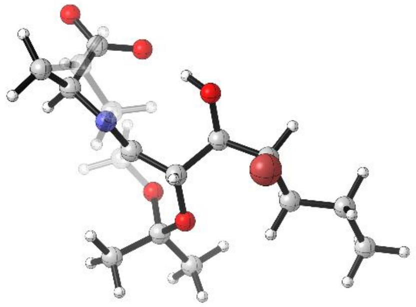

- Thermochemistry -

Zero-point correction=	0.419788 (Hartree/Particle)
Thermal correction to Energy=	0.439918
Thermal correction to Enthalpy=	0.440783
Thermal correction to Gibbs Free Energy=	0.372541
Sum of electronic and zero-point Energies=	-3627.248201
Sum of electronic and thermal Energies=	-3627.228071
Sum of electronic and thermal Enthalpies=	-3627.227206
Sum of electronic and thermal Free Energies=	-3627.295449

Number of Imaginary Frequencies = 0

E (Single Point Energy) [IEFPCM$_{(DCM)}$M06-2X/6-311++G(2d,2p)] = -3630.318696

C	0.11814200	0.31288600	-0.53694700
H	0.18102700	-0.41755500	-1.34710900
C	-1.30566500	0.72350000	-0.30339800
N	-2.28075000	-0.06599300	-0.57466700
C	-3.64807500	0.14091300	-0.06064800
H	-3.94153200	1.18623000	-0.18326300
C	-2.17858200	-1.36968400	-1.30325200
H	-1.34003100	-1.94393500	-0.91312300
H	-2.02050400	-1.12939500	-2.35909500
C	-3.53979400	-2.01879100	-1.05999800

H	-3.51091200	-2.60951400	-0.13938400
H	-3.81581800	-2.67709500	-1.88420300
C	-4.48504200	-0.82578100	-0.89500900
H	-4.72211100	-0.38135000	-1.86642000
H	-5.41302900	-1.07041700	-0.37902300
C	-1.52432800	2.03710800	0.39565800
H	-2.25575500	2.64277700	-0.15741300
H	-1.91628300	1.84585300	1.40009200
O	0.98125200	1.38020300	-0.86003300
O	-0.29535800	2.70898100	0.54693100
C	0.51362600	2.70926200	-0.61740100
C	1.73234100	3.54445300	-0.29230700
H	2.46040300	3.45719500	-1.10135700
H	2.18060200	3.18562500	0.63660200
C	-0.23241200	3.21445400	-1.84898400
H	-0.69273900	4.18372900	-1.64073900
H	-1.00594100	2.51714300	-2.18390200
H	0.48262900	3.32668800	-2.66605500
C	0.57900800	-0.36942600	0.79630300
H	0.58622900	0.40893200	1.57576100
C	2.00692400	-0.91225000	0.72587700
O	-0.27000200	-1.42994100	1.13440600
C	-3.69481100	-0.20302600	1.47975600
O	-2.59909600	-0.44351600	2.05348100
O	-4.83417900	-0.17476900	1.96064300
H	1.44552100	4.59178700	-0.17717300
C	3.13532400	0.10747200	0.70947200
H	3.17846300	0.61667200	-0.25674400

C	4.49538400	-0.49124600	1.06247600
H	4.41868900	-1.03177100	2.01409200
H	4.77744600	-1.22809600	0.30198900
H	2.87854800	0.87195600	1.45695600
C	5.57153100	0.58738600	1.16381400
H	5.32238600	1.31666100	1.94161300
H	6.54581900	0.15527200	1.40672000
H	5.67009300	1.12838800	0.21687100
H	-1.11628500	-1.08650800	1.51510600
H	2.11206300	-1.56963400	1.59208400
Br	2.18154000	-2.15082100	-0.79874600

(*R*)-TS1$_T$-Br-Pre

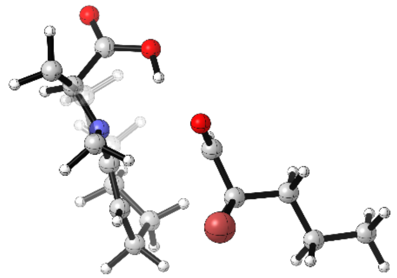

- Thermochemistry -

Zero-point correction= 0.381352 (Hartree/Particle)
Thermal correction to Energy= 0.401336
Thermal correction to Enthalpy= 0.402201
Thermal correction to Gibbs Free Energy= 0.332629
Sum of electronic and zero-point Energies= -3835.742971

Sum of electronic and thermal Energies= -3835.722988
Sum of electronic and thermal Enthalpies= -3835.722123
Sum of electronic and thermal Free Energies= -3835.791695

Number of Imaginary Frequencies = 0

E (Single Point Energy) [IEFPCM$_{(DCM)}$M06-2X/6-311++G(2d,2p)] = -3838.753211

C	-0.39407900	0.97374400	-1.58880000
H	-0.20526600	0.12554800	-2.23879700
C	-1.42515800	0.91616700	-0.71585300
N	-2.15492000	-0.25780600	-0.54134500
C	-3.49913800	-0.28228000	0.02504600
H	-4.11558700	0.56696100	-0.28431900
C	-1.90105200	-1.42740700	-1.38454200
H	-0.85586600	-1.73631300	-1.29024300
H	-2.10589100	-1.19819200	-2.44262000
C	-2.88097200	-2.45893300	-0.83918500
H	-2.46885100	-2.92340600	0.06345800
H	-3.10287500	-3.24822400	-1.55939000
C	-4.10603200	-1.60472800	-0.50363900
H	-4.67132200	-1.38582200	-1.41362200
H	-4.78673100	-2.06320800	0.21660000
C	-1.80864200	2.05657200	0.20436900
H	-2.89216400	2.21429300	0.15728900
H	-1.58256200	1.77671400	1.24251100
C	0.68820200	-0.41027700	1.02309600
H	0.35998400	0.46483200	1.61657400
C	2.02717900	-0.21296200	0.36394300
O	0.03564000	-1.43161000	1.00488800
C	-3.49801200	-0.27684800	1.55540500

O	-2.41163300	-0.74808800	2.16505300
O	-4.45743400	0.09689200	2.19393500
C	3.13474800	-0.39017300	1.40482700
H	2.87195200	0.23445200	2.27038100
C	4.51705000	0.01688500	0.89997300
H	4.79401200	-0.62098100	0.05399600
H	4.47034400	1.04600700	0.52284200
H	2.05678800	0.79277700	-0.05825900
H	3.13648800	-1.43288000	1.74356900
C	5.56767800	-0.08908700	2.00252400
H	5.32278000	0.56942400	2.84189200
H	5.62797700	-1.11301200	2.38510800
H	6.55709300	0.19041500	1.63175200
H	-1.70331200	-0.99694800	1.52974400
C	-1.14095400	3.38504600	-0.12846400
C	0.56018900	2.12116600	-1.77486000
H	-1.55024000	3.80371700	-1.05307600
H	-1.31261700	4.10497000	0.67354100
H	1.57216500	1.73506600	-1.94398800
H	0.30530800	2.72315500	-2.65511100
S	0.65691900	3.22205400	-0.32883400
Br	2.24210000	-1.44618400	-1.11808900

(*R*)-TS1$_{T}$-Br

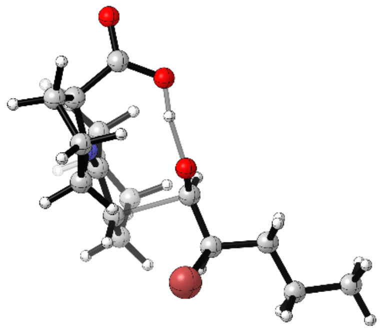

- Thermochemistry -

Zero-point correction=	0.381254 (Hartree/Particle)
Thermal correction to Energy=	0.399604
Thermal correction to Enthalpy=	0.400469
Thermal correction to Gibbs Free Energy=	0.335333
Sum of electronic and zero-point Energies=	-3835.733688
Sum of electronic and thermal Energies=	-3835.715338
Sum of electronic and thermal Enthalpies=	-3835.714473
Sum of electronic and thermal Free Energies=	-3835.779609

Number of Imaginary Frequencies = 1

E (Single Point Energy) [IEFPCM$_{(DCM)}$M06-2X/6-311++G(2d,2p)] = -3838.741514

C	-0.25432600	1.00616200	-1.20608000
H	0.10022000	0.24048900	-1.88925400
C	-1.51923800	0.82501200	-0.65342700
N	-2.07996000	-0.39461200	-0.63564500
C	-3.32519000	-0.72861800	0.06197500
H	-4.12106600	-0.02068000	-0.17848000
C	-1.56347000	-1.54017700	-1.41500400
H	-0.48617100	-1.63529900	-1.29422300
H	-1.80371000	-1.38461100	-2.47525100
C	-2.31492300	-2.72735000	-0.82538100
H	-1.79894100	-3.07268700	0.07688800
H	-2.38374600	-3.56104200	-1.52570300
C	-3.67720600	-2.13149300	-0.47118800

H	-4.28941800	-2.02335000	-1.37098700
H	-4.24206900	-2.70992500	0.26136300
C	-2.21085300	1.90945600	0.14492800
H	-3.29351600	1.82183700	0.01761300
H	-2.00476400	1.73160800	1.21012000
C	0.67681900	0.15826300	0.59483600
H	0.46827100	1.08220800	1.15741900
C	2.11400000	0.10218600	0.11337700
O	0.09173000	-0.91347000	0.90586200
C	-3.21694800	-0.73601300	1.59656900
O	-2.03566800	-0.80554700	2.16055700
O	-4.24542900	-0.70340400	2.24833900
C	3.01772400	-0.19491200	1.30947600
H	2.75227800	0.52660500	2.09562100
C	4.50908500	-0.06972000	1.00833900
H	4.79987400	-0.83727900	0.28321100
H	4.70102000	0.90175300	0.53595700
H	2.41379800	1.02931500	-0.37343200
H	2.77773100	-1.19273300	1.69301400
C	5.34872400	-0.20594100	2.27654700
H	5.10159000	0.58067400	2.99657700
H	5.16829500	-1.17149900	2.76004200
H	6.41652300	-0.13604200	2.05337700
H	-1.18194000	-0.83953800	1.55194900
C	-1.81066100	3.33572300	-0.21975400
C	0.33320200	2.36929500	-1.48077700
H	-2.17591800	3.59750500	-1.21749600
H	-2.25177600	4.03334100	0.49401300

H	1.41986500	2.30680600	-1.57887800
H	-0.03742100	2.76637700	-2.43314600
S	-0.01365800	3.58871200	-0.17915800
Br	2.33153600	-1.30122300	-1.23025600

(*R*)-**TS1**$_{T}$-Br-P

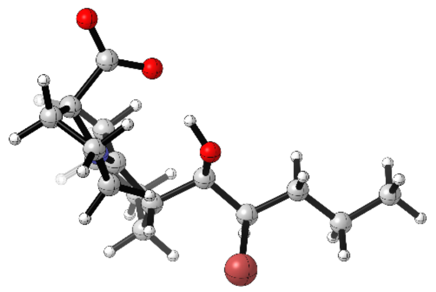

- Thermochemistry -

Zero-point correction=	0.385715 (Hartree/Particle)
Thermal correction to Energy=	0.404101
Thermal correction to Enthalpy=	0.404966
Thermal correction to Gibbs Free Energy=	0.339578
Sum of electronic and zero-point Energies=	-3835.762069
Sum of electronic and thermal Energies=	-3835.743683
Sum of electronic and thermal Enthalpies=	-3835.742818
Sum of electronic and thermal Free Energies=	-3835.808206

Number of Imaginary Frequencies = 0

E (Single Point Energy) [IEFPCM$_{(DCM)}$M06-2X/6-311++G(2d,2p)] = -3838.773806

C	-0.07263300	0.78156100	-0.80078200
H	0.05461100	0.07685100	-1.62356300
C	-1.51250700	0.79325800	-0.37155500
N	-2.25365900	-0.23414900	-0.62029000
C	-3.56646100	-0.48352900	0.01747400
H	-4.20889600	0.39200900	-0.07697600
C	-1.83315500	-1.42867400	-1.42191600

H	-0.81181300	-1.70424800	-1.16917800
H	-1.90501300	-1.14786900	-2.47729900
C	-2.85529300	-2.49656000	-1.04591400
H	-2.52214800	-3.02932500	-0.14980700
H	-2.98513600	-3.22081100	-1.85090000
C	-4.11639200	-1.68792500	-0.74535900
H	-4.59485300	-1.36015500	-1.67352000
H	-4.84935500	-2.22029000	-0.13920500
C	-2.00066200	1.91835700	0.49926300
H	-3.08650800	1.89829100	0.59316800
H	-1.59666200	1.72407000	1.50129800
C	0.72039100	0.22256200	0.42941900
H	0.63792000	0.95458500	1.24798100
C	2.21573600	0.10519800	0.15875400
O	0.23305100	-1.02960700	0.82798800
C	-3.36427800	-0.79058200	1.54618700
O	-2.18826100	-0.75869700	1.99832300
O	-4.41919800	-1.02902200	2.15147300
C	2.96813700	-0.49857700	1.33320900
H	2.63808000	0.04964500	2.22779900
C	4.48697500	-0.39612200	1.21821500
H	4.82905500	-0.99241500	0.36516400
H	4.76638800	0.64414600	1.00884800
H	2.63437500	1.07833100	-0.10304800
H	2.65541200	-1.53840400	1.46648400
C	5.17884100	-0.87196500	2.49376100
H	4.87969800	-0.26263800	3.35265900
H	4.91540400	-1.91166800	2.71352800

H	6.26647600	-0.81315600	2.40086300
H	-0.63366800	-0.93480200	1.29504200
C	-1.56953900	3.31197100	0.02926200
C	0.42229900	2.13921900	-1.31385900
H	-2.03017900	3.55951500	-0.93170500
H	-1.90451800	4.04824700	0.76143500
H	1.47868500	2.07299500	-1.58160400
H	-0.12023500	2.40193300	-2.22721300
S	0.23102400	3.48769100	-0.11554600
Br	2.51310300	-1.00284000	-1.44910700

(*S*)-**TS1**$_{T}$-Br-Pre

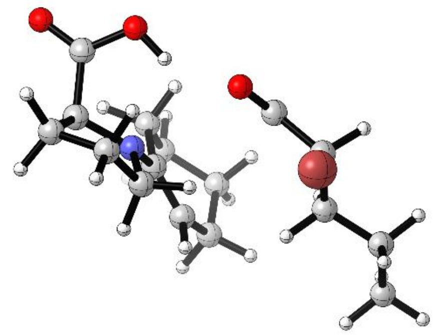

- Thermochemistry -

Zero-point correction= 0.381503 (Hartree/Particle)
Thermal correction to Energy= 0.401362
Thermal correction to Enthalpy= 0.402227
Thermal correction to Gibbs Free Energy= 0.332670
Sum of electronic and zero-point Energies= -3835.741089
Sum of electronic and thermal Energies= -3835.721230
Sum of electronic and thermal Enthalpies= -3835.720365
Sum of electronic and thermal Free Energies= -3835.789922

Number of Imaginary Frequencies = 0

E (Single Point Energy) [IEFPCM$_{(DCM)}$M06-2X/6-311++G(2d,2p)] = -3838.752357

C	-0.17379100	1.52103000	-1.29296600
H	0.22121900	0.87010400	-2.06746100
C	-1.25275300	1.12012400	-0.58931600
N	-1.78372300	-0.16799300	-0.72537800
C	-3.23018400	-0.39744500	-0.69166600
H	-3.80236400	0.44201100	-1.10046400
C	-1.12134700	-1.14576500	-1.58923500
H	-0.11595400	-1.35573300	-1.20940800
H	-1.03602100	-0.77782900	-2.62412000
C	-2.06035300	-2.34275700	-1.52576900
H	-1.91160300	-2.88558100	-0.58570900
H	-1.91259400	-3.03749300	-2.35432100
C	-3.43303400	-1.66557200	-1.55661600
H	-3.67385700	-1.35772300	-2.57790400
H	-4.25030700	-2.28817800	-1.18756600
C	-1.90760200	1.95466500	0.49102900
H	-2.99746100	1.93263800	0.36642600
H	-1.69877300	1.48850800	1.46369100
C	0.80563300	-0.27996400	1.42640800
H	0.68046800	0.77607200	1.73305900
C	2.21459600	-0.61909200	0.99983200
O	-0.11482800	-1.06377100	1.47579500
C	-3.77017300	-0.64090600	0.71874200
O	-2.89126100	-0.97098000	1.65875900
O	-4.95621600	-0.57909300	0.96279100
C	2.79630100	0.42067100	0.05011100
H	2.27870600	0.35603400	-0.91373900
C	4.30548500	0.30733200	-0.14752600

H	4.79380300	0.25892600	0.83365500
H	4.53607100	-0.63065400	-0.66336200
H	2.81011500	-0.65983800	1.91993800
H	2.55919800	1.40576500	0.47887600
C	4.85024800	1.49269100	-0.94077600
H	4.37379800	1.55467200	-1.92502000
H	4.66031900	2.43443200	-0.41553100
H	5.92845800	1.40390900	-1.09570400
H	-1.96886200	-0.92600800	1.32252600
C	-1.47407900	3.41482400	0.51672600
C	0.54941500	2.83250900	-1.13925200
H	-1.87597000	3.95759000	-0.34455100
H	-1.83829700	3.90145100	1.42313300
H	1.62542600	2.69088200	-1.27745500
H	0.22691400	3.55532600	-1.89817500
S	0.33493100	3.58615800	0.50119500
Br	2.30351400	-2.40671900	0.24573100

(*S*)-**TS1**$_T$-Br

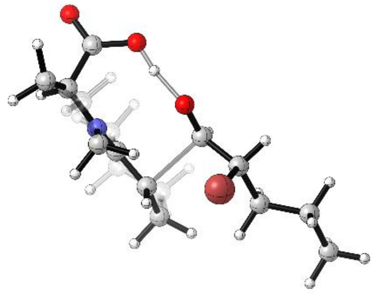

- Thermochemistry -

Zero-point correction=	0.383176 (Hartree/Particle)
Thermal correction to Energy=	0.401127
Thermal correction to Enthalpy=	0.401992
Thermal correction to Gibbs Free Energy=	0.338653
Sum of electronic and zero-point Energies=	-1363.732980
Sum of electronic and thermal Energies=	-1363.715029
Sum of electronic and thermal Enthalpies=	-1363.714164
Sum of electronic and thermal Free Energies=	-1363.777503

Number of Imaginary Frequencies = 1

E (Single Point Energy) [IEFPCM$_{(DCM)}$M06-2X/6-311++G(2d,2p)] = -1364.414632

C	0.08134300	1.15049800	-1.12315300
H	0.47068800	0.53827400	-1.93191900
C	-1.17308000	0.81690100	-0.61974100
N	-1.65918300	-0.42377600	-0.78693100
C	-2.89563400	-0.92522500	-0.17905400
H	-3.73096300	-0.24254200	-0.34946000
C	-1.06125700	-1.41343900	-1.70624400
H	0.01943000	-1.44754400	-1.58071600
H	-1.30333400	-1.12994500	-2.73939800
C	-1.73773400	-2.71670300	-1.29975600
H	-1.21359100	-3.14554600	-0.43913300
H	-1.73896700	-3.45058000	-2.10708500
C	-3.14291400	-2.26348500	-0.90369900
H	-3.74615300	-2.07541100	-1.79640200
H	-3.67962300	-2.97032200	-0.26923700
C	-1.94727500	1.72926500	0.30776900
H	-3.01866300	1.59578800	0.13182700
H	-1.75437800	1.41449200	1.34250600
C	1.11866300	0.03795800	0.45709000

H	1.05665500	0.91720100	1.11645500
C	2.44888500	-0.12043500	-0.25974100
O	0.48587200	-1.01705400	0.73270200
C	-2.82984700	-1.13132200	1.34463700
O	-1.66554200	-1.17314300	1.94492400
O	-3.87626200	-1.27480500	1.95136400
C	3.49203000	-0.68926900	0.69002900
H	3.55520000	-0.02728000	1.56341900
C	4.86895100	-0.82826300	0.04205500
H	4.78741300	-1.46727500	-0.84369000
H	5.20507900	0.15636700	-0.30618700
H	2.79229400	0.82726600	-0.68577200
H	3.13031000	-1.66146600	1.04574500
C	5.89537600	-1.41292100	1.01018500
H	6.00568700	-0.77665000	1.89432400
H	5.58744900	-2.40706700	1.34967300
H	6.87677800	-1.50730000	0.53787500
H	-0.79970500	-1.06031100	1.37065800
C	-1.62818200	3.21180600	0.15934000
C	0.63339100	2.55659000	-1.11490700
H	-1.96564100	3.58896200	-0.81093900
H	-2.14067600	3.77847900	0.93845100
H	1.72668600	2.53770900	-1.11144200
H	0.33644400	3.09660000	-2.02139800
S	0.14583900	3.54310000	0.33037200
F	2.28151100	-1.01075400	-1.32626200

(*S*)-**TS1**$_{T}$-Br-P

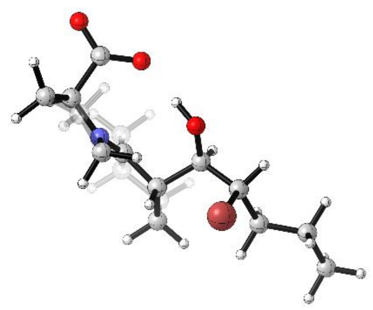

- Thermochemistry -

Zero-point correction=	0.385628 (Hartree/Particle)
Thermal correction to Energy=	0.404013
Thermal correction to Enthalpy=	0.404878
Thermal correction to Gibbs Free Energy=	0.339134
Sum of electronic and zero-point Energies=	-3835.757857
Sum of electronic and thermal Energies=	-3835.739472
Sum of electronic and thermal Enthalpies=	-3835.738607
Sum of electronic and thermal Free Energies=	-3835.804351

Number of Imaginary Frequencies = 0

E (Single Point Energy) [IEFPCM$_{(DCM)}$M06-2X/6-311++G(2d,2p)] = -3838.769222

C	0.12902500	0.44852500	-0.71182100
H	0.20502200	-0.43613700	-1.34625900
C	-1.32322000	0.78237100	-0.49936500
N	-2.21734600	-0.13327400	-0.66366200
C	-3.62700700	-0.00610500	-0.22645300
H	-4.05930500	0.91797700	-0.61090800
C	-1.94395400	-1.53037600	-1.12948500
H	-1.03717300	-1.90484900	-0.65860800
H	-1.82269400	-1.49133100	-2.21637300
C	-3.19685200	-2.29927200	-0.72301400
H	-3.09394700	-2.65716000	0.30606300
H	-3.36225400	-3.15868000	-1.37373000
C	-4.30047600	-1.24634200	-0.81249700

H	-4.57941500	-1.06760000	-1.85558500
H	-5.19719900	-1.49599500	-0.24549100
C	-1.67670800	2.13226500	0.06316700
H	-2.75404100	2.29545600	0.05069400
H	-1.37822700	2.10667100	1.11934700
C	0.68649400	0.06476200	0.69960000
H	0.76298100	0.98221200	1.30328400
C	2.09176900	-0.53866700	0.69391900
O	-0.14264700	-0.88218300	1.32567700
C	-3.70128200	0.02432600	1.34365400
O	-2.61483500	-0.01494000	1.98030500
O	-4.85622200	0.09576100	1.78799100
C	3.27447300	0.38591600	0.42222700
H	3.47167000	0.46545400	-0.65113700
C	4.54970300	-0.08346800	1.12358700
H	4.37122000	-0.11883300	2.20480600
H	4.77850000	-1.10781400	0.80548000
H	2.20563100	-0.99022300	1.68162100
H	3.01234400	1.39185600	0.77985800
C	5.73315600	0.83141500	0.82043600
H	5.94313700	0.85351700	-0.25381700
H	5.52692400	1.85706400	1.14332400
H	6.63703500	0.49240800	1.33297800
H	-1.01930500	-0.50627800	1.58735700
C	-0.98461400	3.30855900	-0.63413600
C	0.87336500	1.57132100	-1.44729800
H	-1.31994700	3.40117900	-1.67148300
H	-1.24795600	4.22973800	-0.11220900

H	1.91813400	1.29884300	-1.58778400
H	0.44089800	1.69192100	-2.44557200
S	0.82459800	3.17450200	-0.60182600
Br	2.15234900	-2.10197100	-0.50451200

Supplementary Table 7. Energies for enamine addition to 2-chloropentanal. Reported energies for structures optimized at the IEFPCM$_{(DCM)}$M06-2X/6-311++G(2d,2p)//IEFPCM$_{(DCM)}$M06-2X/6-31+G(d,p) level of theory represent the sum of the thermal correction to Gibbs Free Energy computed at the IEFPCM$_{(DCM)}$M06-2X/6-31+G(d,p) level of theory and single point energies computed at the IEFPCM$_{(DCM)}$M06-2X/6-311++G(2d,2p). All energies are reported in Hartrees.

**Structure**	**Single Point Energies, E IEFPCM$_{(DCM)}$M06-2X/6-311++G(2d,2p)**	**Thermal Corrections to Gibbs Free Energies, IEFPCM$_{(DCM)}$M06-2X/6-31+G(d,p)**	**Gibbs Free Energies (G), IEFPCM$_{(DCM)}$M06-2X/6-31+G(d,p)**	**Gibbs Free Energies (G), IEFPCM$_{(DCM)}$M06-2X/6-311++G(2d,2p)// IEFPCM$_{(DCM)}$M06-2X/6-31+G(d,p)**
Chloropentanal	-731.33393130	0.102879	-731.121809	-731.2310523
Enamine of Cyclohexanone (G)	-634.55147535	0.238392	-634.142903	-634.3130834
Enamine of Dioxane (O)	-784.98483791	0.244077	-784.522619	-784.7407609
Enamine of Tetrahydro-4H-thiopyranone (T)	-993.43210840	0.210205	-993.028735	-993.2219034
Enamine of Tetrahydro-4H-pyranone (P)	-670.45469021	0.214793	-670.055312	-670.2398972
(*R*)-**TS2**$_P$-Cl-Pre	-1401.805196	0.337854	-1401.173571	-1401.467342
(*R*)-**TS2**$_P$-Cl	-1401.790092	0.341689	-1401.156596	-1401.448403
(*R*)-**TS2**$_P$-Cl-P	-1401.820368	0.345347	-1401.183637	-1401.475021
(*S*)-**TS2**$_P$-Cl-Pre	-1401.806134	0.33645	-1401.17629	-1401.469684
(*S*)-**TS2**$_P$-Cl	-1401.796294	0.340244	-1401.164151	-1401.45605
(*S*)-**TS2**$_P$-Cl-P	-1401.825541	0.344533	-1401.189579	-1401.481008
(*R*)-**TS2**$_G$-Cl-Pre	-1365.902257	0.361551	-1365.261681	-1365.540706
(*R*)-**TS2**$_G$-Cl	-1365.887212	0.365714	-1365.244059	-1365.521498
(*R*)-**TS2**$_G$-Cl-P	-1365.918402	0.369157	-1365.272168	-1365.549245
(*S*)-**TS2**$_G$-Cl-Pre	-1365.903542	0.361695	-1365.262706	-1365.541847
(*S*)-**TS2**$_G$-Cl	-1365.893626	0.364656	-1365.251485	-1365.52897
(*S*)-**TS2**$_G$-Cl-P	-1365.924651	0.368381	-1365.279269	-1365.55627
(*R*)-**TS2**$_O$-Cl-Pre	-1516.336462	0.365492	-1515.644382	-1515.97097
(*R*)-**TS2**$_O$-Cl	-1516.319381	0.369078	-1515.62514	-1515.950303

(*R*)-**TS2**$_O$-Cl-P	-1516.346706	0.372289	-1515.64974	-1515.974417
(*S*)-**TS2**$_O$-Cl-Pre	-1516.338534	0.366524	-1515.645123	-1515.97201
(*S*)-**TS2**$_O$-Cl	-1516.32557	0.369297	-1515.630967	-1515.956273
(*S*)-**TS2**$_O$-Cl-P	-1516.350766	0.37202	-1515.653866	-1515.978746
(*R*)-**TS2**$_T$-Cl-Pre	-1724.781246	0.33334	-1724.145143	-1724.447906
(*R*)-**TS2**$_T$-Cl	-1724.766523	0.337245	-1724.128796	-1724.429278
(*R*)-**TS2**$_T$-Cl-P	-1724.794106	0.341075	-1724.153172	-1724.453031
(*S*)-**TS2**$_T$-Cl-Pre	-1724.783329	0.334442	-1724.146124	-1724.448887
(*S*)-**TS2**$_T$-Cl	-1724.773577	0.336784	-1724.136217	-1724.436793
(*S*)-**TS2**$_T$-Cl-P	-1724.801604	0.34048	-1724.161179	-1724.461124

Pre – Precomplex

P – Product

(*R*)-**TS2**$_P$-Cl-Pre

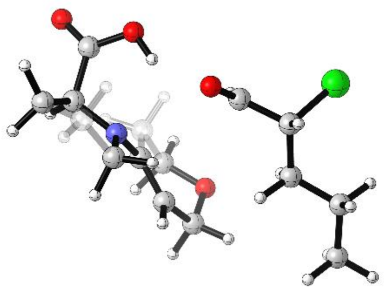

- Thermochemistry -

Zero-point correction= 0.385733 (Hartree/Particle)
Thermal correction to Energy= 0.405077
Thermal correction to Enthalpy= 0.405942
Thermal correction to Gibbs Free Energy= 0.337854
Sum of electronic and zero-point Energies= -1401.125691
Sum of electronic and thermal Energies= -1401.106347
Sum of electronic and thermal Enthalpies= -1401.105482
Sum of electronic and thermal Free Energies= -1401.173571

Number of Imaginary Frequencies = 0

E (Single Point Energy) [IEFPCM$_{(DCM)}$M06-2X/6-311++G(2d,2p)] = -1401.805196

C	-0.03542800	1.86595000	0.80552100
H	-0.13136300	1.72320300	1.87713800
C	0.96057200	1.28564400	0.10718100
N	1.85065000	0.37096200	0.66795300
C	3.21554300	0.22601600	0.16702100
H	3.64447800	1.16706500	-0.19124700
C	1.76801900	0.05131700	2.09160700
H	0.79330200	-0.39399000	2.31486700
H	1.88812100	0.95629900	2.70874100
C	2.93456500	-0.90873700	2.29095200
H	2.65580100	-1.91125500	1.94828500
H	3.25001500	-0.97722500	3.33342300
C	4.01153200	-0.30159500	1.38679500
H	4.48397100	0.54838300	1.88647400
H	4.79639200	-1.00281700	1.09597800
C	1.09607800	1.53051800	-1.37911300
H	2.12789100	1.79261800	-1.63908700
H	0.86005400	0.60947300	-1.92927100
C	-0.89008100	-1.45774700	-0.23728900
H	-0.69290800	-0.86891600	-1.15269800
C	-2.34149900	-1.56443000	0.16569600
O	0.00916500	-1.97356800	0.39096400
C	3.31194200	-0.76453300	-0.99344600
O	2.35831600	-1.68932200	-1.07562000
O	4.23545100	-0.74653700	-1.77782800
C	-2.90514700	-0.18909700	0.49699700

H	-2.29874400	0.19997300	1.32734300
C	-4.37831400	-0.19343900	0.89842800
H	-4.98220900	-0.57610600	0.06933400
H	-4.52062000	-0.88026700	1.74146700
H	-2.44565200	-2.26937500	0.99231000
H	-2.74042800	0.47826300	-0.35994500
C	-4.85212700	1.20790300	1.27862700
H	-4.27459300	1.60299700	2.12102900
H	-5.90653800	1.20313500	1.56608800
H	-4.73620100	1.89877800	0.43692800
H	1.66373300	-1.56230900	-0.39015400
Cl	-3.18226900	-2.29924500	-1.25843700
C	0.17973500	2.66580500	-1.82724700
C	-1.06519700	2.73940600	0.13848900
H	0.60798000	3.63472600	-1.52927800
H	0.05685000	2.65987000	-2.91158000
H	-2.06733700	2.51817800	0.51913700
H	-0.86868300	3.80572800	0.34072800
O	-1.11298600	2.53144200	-1.26246000

(*R*)-**TS2**$_{P}$-Cl

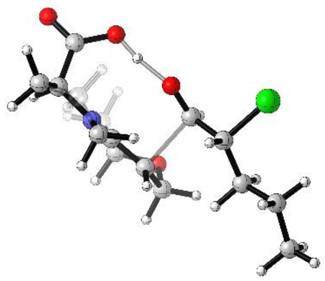

- Thermochemistry -

Zero-point correction=	0.385675 (Hartree/Particle)
Thermal correction to Energy=	0.403307
Thermal correction to Enthalpy=	0.404172
Thermal correction to Gibbs Free Energy=	0.341689
Sum of electronic and zero-point Energies=	-1401.112610
Sum of electronic and thermal Energies=	-1401.094979
Sum of electronic and thermal Enthalpies=	-1401.094113
Sum of electronic and thermal Free Energies=	-1401.156596

Number of Imaginary Frequencies = 1

E (Single Point Energy) [IEFPCM$_{(DCM)}$M06-2X/6-311++G(2d,2p)] = -1401.790092

C	-0.35156100	1.19807000	0.75817100
H	-0.76364900	0.67329000	1.61569300
C	1.01853800	1.08759800	0.52163100
N	1.71434700	0.02228500	0.92678100
C	3.12860700	-0.21718100	0.61863100
H	3.73484300	0.66194900	0.84827000
C	1.17181000	-0.99783100	1.84237200
H	0.17169000	-1.29125500	1.52668000
H	1.12637000	-0.58206500	2.85723000
C	2.18188800	-2.13361400	1.73288700
H	1.95167900	-2.74241700	0.85240400
H	2.17701400	-2.77758300	2.61334600
C	3.50579100	-1.39131300	1.54376900
H	3.85509800	-0.99079200	2.49958700
H	4.30208100	-2.00044800	1.11436300
C	1.69793200	2.06805200	-0.40241200
H	2.72810300	2.25023300	-0.08448700
H	1.73883700	1.63088300	-1.40933400
C	-0.85562600	-0.27027600	-0.78545000
H	-0.97727800	0.56491400	-1.49257600

C	-2.15670500	-0.87777200	-0.24757200
O	0.10063600	-1.08389500	-0.90819600
C	3.42741700	-0.55699000	-0.85297000
O	2.44812300	-0.87875700	-1.66174300
O	4.58851900	-0.53463500	-1.21837500
C	-3.27930300	0.03619400	0.21410800
H	-2.90660000	0.61356400	1.06734400
C	-4.53928300	-0.70356300	0.66760400
H	-4.98479100	-1.23097900	-0.18066100
H	-4.26066300	-1.46715200	1.40499400
H	-1.89873200	-1.60939400	0.52293600
H	-3.53049000	0.74607200	-0.58412600
C	-5.56197800	0.25548100	1.27366300
H	-5.15246400	0.76538400	2.15173500
H	-6.46529700	-0.27618000	1.58361800
H	-5.85511400	1.02092800	0.54771600
H	1.47615200	-0.88777800	-1.29005500
Cl	-2.76064400	-1.88680000	-1.65305200
C	0.93500300	3.38523800	-0.45339400
C	-1.04968600	2.49278900	0.38345000
H	1.04480100	3.93075200	0.49552400
H	1.30941200	4.01399800	-1.26234900
H	-2.08036800	2.31376400	0.07481600
H	-1.07743000	3.17481300	1.24888000
O	-0.43349600	3.14301200	-0.71094400

(*R*)-**TS2**$_{P}$-Cl-P

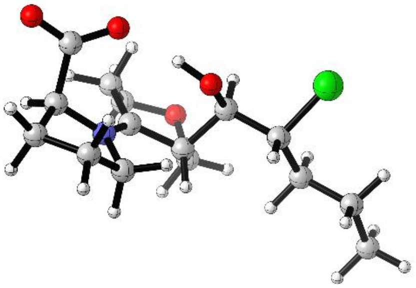

- Thermochemistry -

Zero-point correction=	0.389864 (Hartree/Particle)
Thermal correction to Energy=	0.407526
Thermal correction to Enthalpy=	0.408391
Thermal correction to Gibbs Free Energy=	0.345347
Sum of electronic and zero-point Energies=	-1401.139120
Sum of electronic and thermal Energies=	-1401.121458
Sum of electronic and thermal Enthalpies=	-1401.120593
Sum of electronic and thermal Free Energies=	-1401.183637

Number of Imaginary Frequencies = 0

E (Single Point Energy) [IEFPCM$_{(DCM)}$M06-2X/6-311++G(2d,2p)] = -1401.820368

C	-0.42826200	0.78308600	0.55196000
H	-0.61517200	0.22567200	1.47451500
C	1.06180600	0.89722400	0.33872000
N	1.86676800	0.03941200	0.86896300
C	3.31069900	-0.05899100	0.54981600
H	3.78097500	0.92014000	0.64868800
C	1.44839200	-1.10342300	1.73531200
H	0.55751800	-1.56636500	1.31583800
H	1.23921200	-0.70525200	2.73279600
C	2.66417100	-2.02603400	1.73260600

H	2.61435900	-2.70149100	0.87297100
H	2.71019700	-2.62544300	2.64254000
C	3.83654000	-1.05785200	1.57910400
H	4.04471100	-0.55157700	2.52696700
H	4.75239200	-1.52612200	1.21928200
C	1.55006200	1.95000000	-0.61309900
H	2.63648200	2.00989400	-0.62695000
H	1.25007900	1.60453500	-1.61340600
C	-0.98062700	-0.06670200	-0.65385100
H	-1.15548000	0.61467800	-1.49944600
C	-2.29359000	-0.79331500	-0.31147800
O	-0.10381900	-1.10950100	-1.00718900
C	3.51629400	-0.55633700	-0.92729900
O	2.49241100	-0.69056500	-1.64646800
O	4.70483900	-0.74878100	-1.22756600
C	-3.39976900	-0.02777900	0.39765700
H	-2.98589000	0.34329100	1.34361500
C	-4.62690300	-0.87887500	0.72844200
H	-5.10991200	-1.20543100	-0.19706000
H	-4.30288600	-1.78637900	1.25363900
H	-2.01956000	-1.67519600	0.27674700
H	-3.69993600	0.84300400	-0.19875400
C	-5.62665900	-0.10916000	1.58924400
H	-5.17783300	0.19130700	2.54166700
H	-6.50765700	-0.71752600	1.80937400
H	-5.96429900	0.79765600	1.07683500
H	0.78803300	-0.82066100	-1.31333500
C	0.91508700	3.31882900	-0.34401600

C	-1.06041700	2.18846800	0.69557900
H	1.25550800	3.70278900	0.62507400
H	1.26397900	4.01618400	-1.10969900
H	-2.14609800	2.10686100	0.68037700
H	-0.79275600	2.56903800	1.68885800
C	-0.60253000	3.19958000	-0.35399900
H	-1.06503600	4.16731900	-0.13944900
H	-0.94180400	2.90216600	-1.35410000
Cl	-2.93704300	-1.46885400	-1.87522000

(*S*)-**TS2**$_{P}$-Cl-Pre

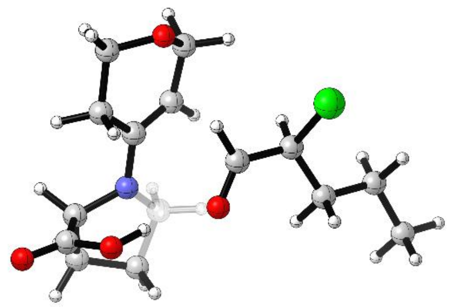

- Thermochemistry -

Zero-point correction=	0.385207 (Hartree/Particle)
Thermal correction to Energy=	0.404644
Thermal correction to Enthalpy=	0.405509
Thermal correction to Gibbs Free Energy=	0.336450
Sum of electronic and zero-point Energies=	-1401.127532
Sum of electronic and thermal Energies=	-1401.108095
Sum of electronic and thermal Enthalpies=	-1401.107230
Sum of electronic and thermal Free Energies=	-1401.176290

Number of Imaginary Frequencies = 0

E (Single Point Energy) [IEFPCM$_{(DCM)}$M06-2X/6-311++G(2d,2p)] = -1401.806134

C	-0.23490100	1.27332000	-1.56444800
H	0.22284600	0.57226900	-2.25558100

C	-1.26920500	0.90865700	-0.77840400
N	-1.70676000	-0.40503700	-0.66546700
C	-3.08073100	-0.74480800	-0.30248500
H	-3.81164600	-0.02412600	-0.68215500
C	-1.08892600	-1.45736100	-1.47114700
H	-0.02377000	-1.53334600	-1.22784400
H	-1.18430800	-1.23861600	-2.54632400
C	-1.89019700	-2.69627700	-1.08861700
H	-1.53256100	-3.09215000	-0.13188700
H	-1.82242600	-3.48757300	-1.83692600
C	-3.30661700	-2.13241100	-0.94773800
H	-3.75199600	-1.98893800	-1.93602100
H	-3.98056700	-2.75469300	-0.35635300
C	-1.91068500	1.91560700	0.14810900
H	-3.00340300	1.86377900	0.08889600
H	-1.63704200	1.68328600	1.18688100
C	1.14972200	0.31321600	0.95643300
H	0.82672700	1.35036100	1.15097100
C	2.49203400	0.15632400	0.25966000
H	2.37871400	0.50350600	-0.77345500
O	0.48361400	-0.64235200	1.29528200
C	-3.30277500	-0.82453200	1.21052000
O	-2.22652900	-0.88961600	1.98775000
O	-4.41853700	-0.86662300	1.68180500
H	-1.39436700	-0.79815200	1.47376700
C	3.04396300	-1.25611000	0.30505100
H	2.25985700	-1.90983800	-0.09775100
C	4.33207400	-1.44291900	-0.49305700

H	4.16863400	-1.11156300	-1.52632400
H	3.19079600	-1.55080300	1.35082900
C	4.79479200	-2.89838600	-0.48259400
H	5.72078000	-3.02223500	-1.04994900
H	4.97852000	-3.24059100	0.54088800
H	4.03744400	-3.55395600	-0.92430100
H	5.11713100	-0.80273600	-0.07674200
Cl	3.60461200	1.35406200	1.03857500
C	0.35544500	2.65943100	-1.51111800
C	-1.46082900	3.32789600	-0.20657600
H	1.44982600	2.61258900	-1.46553800
H	0.09285000	3.23703900	-2.41274800
H	-1.94250200	3.65860500	-1.13881400
H	-1.71949600	4.03026000	0.58740300
O	-0.05230900	3.37408500	-0.35733100

(*S*)-**TS2**$_{P}$-Cl

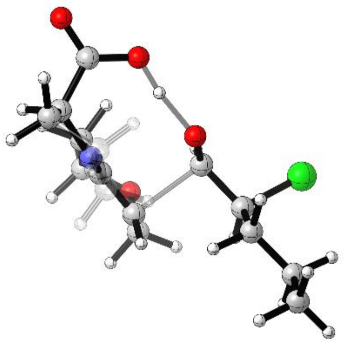

- Thermochemistry -

Zero-point correction=	0.385109 (Hartree/Particle)
Thermal correction to Energy=	0.402973
Thermal correction to Enthalpy=	0.403838
Thermal correction to Gibbs Free Energy=	0.340244
Sum of electronic and zero-point Energies=	-1401.119287

Sum of electronic and thermal Energies= -1401.101422
Sum of electronic and thermal Enthalpies= -1401.100557
Sum of electronic and thermal Free Energies= -1401.164151

Number of Imaginary Frequencies = 1

E (Single Point Energy) [IEFPCM$_{(DCM)}$M06-2X/6-311++G(2d,2p)] = -1401.796294

C	0.14636200	1.08200700	-1.24340000
H	0.68652100	0.36224800	-1.85227600
C	-1.16385600	0.79473300	-0.86835500
N	-1.62341200	-0.45948500	-0.80907800
C	-2.93871300	-0.83788600	-0.28401800
H	-3.72950700	-0.21799900	-0.71186400
C	-0.90015800	-1.61626000	-1.37034900
H	0.14150600	-1.59463900	-1.05576400
H	-0.95597900	-1.57756900	-2.46593100
C	-1.65574900	-2.81065800	-0.79832900
H	-1.28014000	-3.03447800	0.20570500
H	-1.54584400	-3.70266600	-1.41656800
C	-3.09719500	-2.30357300	-0.73279900
H	-3.55080200	-2.32158800	-1.72779400
H	-3.73767900	-2.86509400	-0.05152500
C	-2.02226100	1.89545900	-0.29386700
H	-3.07013300	1.75475700	-0.57167300
H	-1.96844400	1.84724200	0.80289300
C	0.88677800	0.50725600	0.72427800
H	0.58968500	1.49229700	1.11373500
C	2.36785400	0.39917300	0.36981700
H	2.68052000	1.20982000	-0.28820800
O	0.29766800	-0.53644400	1.11774900

C	-3.08426700	-0.71169800	1.24327900
O	-2.01667000	-0.57804800	1.99077800
O	-4.20504300	-0.76505400	1.71669100
H	-1.07349500	-0.54248800	1.53749900
C	2.79114900	-0.95200500	-0.17531000
H	2.21561100	-1.11772800	-1.09588800
C	4.27844200	-1.05630000	-0.50836100
H	4.55963000	-0.23165400	-1.17556900
H	2.49323100	-1.73370900	0.53155800
C	4.61451900	-2.39294300	-1.16665000
H	5.68159300	-2.46536300	-1.39230900
H	4.35225200	-3.22705600	-0.50784000
H	4.06231500	-2.51950400	-2.10370200
H	4.86735300	-0.93691100	0.40672000
Cl	3.23446600	0.74968300	1.94199900
C	0.55884300	2.52768900	-1.42864300
C	-1.55007500	3.26494200	-0.76989300
H	1.61599600	2.67266200	-1.19059000
H	0.41721000	2.83535900	-2.47715700
H	-1.79315100	3.40412500	-1.83355800
H	-2.03361700	4.05630300	-0.19576100
O	-0.15554400	3.40277100	-0.57759000

(S)-**TS2**$_P$-Cl-P

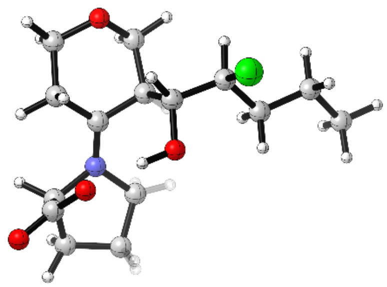

- Thermochemistry -

Zero-point correction=	0.389466 (Hartree/Particle)
Thermal correction to Energy=	0.407306
Thermal correction to Enthalpy=	0.408171
Thermal correction to Gibbs Free Energy=	0.344533
Sum of electronic and zero-point Energies=	-1401.144646
Sum of electronic and thermal Energies=	-1401.126806
Sum of electronic and thermal Enthalpies=	-1401.125941
Sum of electronic and thermal Free Energies=	-1401.189579

Number of Imaginary Frequencies = 0

E (Single Point Energy) [IEFPCM$_{(DCM)}$M06-2X/6-311++G(2d,2p)] = -1401.825541

C	0.32444300	0.79186100	-0.93165500
H	0.62815300	-0.00506000	-1.61354500
C	-1.15576600	0.73412400	-0.68581600
N	-1.80566500	-0.37406100	-0.79267000
C	-3.18367900	-0.57170400	-0.28786600
H	-3.84241900	0.20604900	-0.67531700
C	-1.22411100	-1.67240200	-1.24956200
H	-0.22827600	-1.79585400	-0.82922100
H	-1.17882200	-1.64223000	-2.34259900
C	-2.22560200	-2.70944100	-0.74984600

H	-1.98523100	-2.98887200	0.28056500
H	-2.20758000	-3.60835700	-1.36702700
C	-3.55826600	-1.96169900	-0.80249600
H	-3.92361100	-1.89821600	-1.83215500
H	-4.33636000	-2.40217400	-0.17912700
C	-1.79495900	1.98660100	-0.16562900
H	-2.88129600	1.96201500	-0.25375100
H	-1.55959400	2.03296200	0.90565400
C	0.98879400	0.56886600	0.47765700
H	0.81138700	1.47991800	1.06531200
C	2.50309700	0.37660600	0.34298200
H	2.93305200	1.18767800	-0.24759800
O	0.47033900	-0.56443400	1.11790100
C	-3.20104100	-0.49700800	1.28270000
O	-2.10968600	-0.27730400	1.87177700
O	-4.32675100	-0.66281300	1.77467900
H	-0.45295200	-0.41537000	1.43759500
C	2.91234300	-0.97569000	-0.22472600
H	2.37724100	-1.11463500	-1.17444100
C	4.41057400	-1.11181100	-0.49154600
H	4.73906200	-0.29175100	-1.14265300
H	2.56652600	-1.76354700	0.45217400
C	4.74847200	-2.45366100	-1.13811100
H	5.82233800	-2.54632900	-1.32003100
H	4.44344100	-3.28348000	-0.49247700
H	4.23294100	-2.56864500	-2.09737900
H	4.95954600	-1.00696000	0.44995600
Cl	3.23517300	0.59852300	1.99274700

C	0.69830800	2.14226500	-1.56875800
C	-1.23290300	3.23038100	-0.86360700
H	1.78119200	2.26648500	-1.60485900
H	0.32178200	2.17110800	-2.60126800
H	-1.58404200	3.28065900	-1.90405300
H	-1.56591300	4.12601900	-0.33898600
O	0.18021500	3.22935000	-0.83499500

(*R*)-**TS2**$_{G}$-Cl-Pre

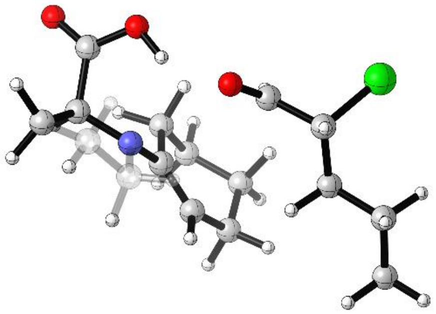

- Thermochemistry -

Zero-point correction=	0.409337 (Hartree/Particle)
Thermal correction to Energy=	0.428876
Thermal correction to Enthalpy=	0.429741
Thermal correction to Gibbs Free Energy=	0.361551
Sum of electronic and zero-point Energies=	-1365.213895
Sum of electronic and thermal Energies=	-1365.194356
Sum of electronic and thermal Enthalpies=	-1365.193491
Sum of electronic and thermal Free Energies=	-1365.261681

Number of Imaginary Frequencies = 0

E (Single Point Energy) [IEFPCM$_{(DCM)}$M06-2X/6-311++G(2d,2p)] = -1365.902257

C	-0.03981000	1.76907700	0.89258900
H	-0.20765700	1.46156200	1.92091300
C	0.99608500	1.24716500	0.20280500

N	1.80003600	0.22183000	0.71828500
C	3.21448000	0.11522900	0.36344200
H	3.69290500	1.08657800	0.20239900
C	1.55939500	-0.28703800	2.06685000
H	0.55687900	-0.72219200	2.12686900
H	1.63457800	0.51770600	2.81634500
C	2.67597700	-1.30718300	2.24907600
H	2.41991200	-2.23721400	1.72975000
H	2.86827900	-1.53905300	3.29811400
C	3.85865300	-0.61001700	1.57116700
H	4.28350900	0.14159300	2.24203300
H	4.66138900	-1.28509000	1.26754200
C	1.29957100	1.66163400	-1.22238500
H	2.37481500	1.82968500	-1.34787100
H	1.04645800	0.82772500	-1.89452200
C	-0.97292500	-1.33683700	-0.56875700
H	-0.82854300	-0.60460400	-1.38506100
C	-2.39530900	-1.51315400	-0.08806000
O	-0.04279000	-1.95673100	-0.10104100
C	3.43761700	-0.69583400	-0.91315500
O	2.47012800	-1.53851600	-1.26431600
O	4.46416300	-0.61736900	-1.55326200
C	-2.94455400	-0.21648100	0.49074300
H	-2.26885900	0.07425900	1.30676600
C	-4.37248500	-0.32034000	1.02157700
H	-5.04650600	-0.60739900	0.20791800
H	-4.42014700	-1.11796600	1.77263300
H	-2.44011700	-2.33919500	0.62409200

H	-2.87898900	0.57004800	-0.27277600
C	-4.83193800	1.00190300	1.63279700
H	-4.17928700	1.29937800	2.46019300
H	-5.85178700	0.92502600	2.01801500
H	-4.81349800	1.80260900	0.88608400
H	1.69218100	-1.46008700	-0.66668400
C	0.54033900	2.92145000	-1.64266700
C	-0.99479100	2.78843600	0.32203600
H	1.00830500	3.79895400	-1.17842000
H	0.62079700	3.04880100	-2.72620600
H	-2.01727700	2.55857700	0.64711600
H	-0.76932700	3.78228900	0.73537800
C	-0.91959400	2.84753700	-1.20265100
H	-1.48367800	3.70616600	-1.57981400
H	-1.37959900	1.94563200	-1.63220800
Cl	-3.33360400	-2.02993900	-1.54765100

(*R*)-**TS2**$_{G}$-Cl

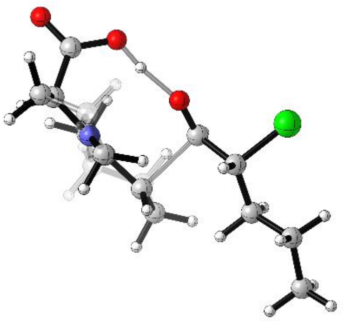

- Thermochemistry -

Zero-point correction= 0.381847 (Hartree/Particle)
Thermal correction to Energy= 0.399956
Thermal correction to Enthalpy= 0.400821
Thermal correction to Gibbs Free Energy= 0.337245

Sum of electronic and zero-point Energies= -1724.084194
Sum of electronic and thermal Energies= -1724.066085
Sum of electronic and thermal Enthalpies= -1724.065220
Sum of electronic and thermal Free Energies= -1724.128796

Number of Imaginary Frequencies = 1

E (Single Point Energy) [IEFPCM$_{(DCM)}$M06-2X/6-311++G(2d,2p)] = -1724.766523

C	0.30963300	0.91748400	-0.88416900
H	0.73216900	0.24609600	-1.62800000
C	-1.06897400	0.80494900	-0.66539500
N	-1.69478400	-0.34157400	-0.94909500
C	-3.06228100	-0.68739000	-0.53327800
H	-3.79416700	-0.07397300	-1.06971400
C	-1.11659000	-1.37666600	-1.83033900
H	-0.35526700	-1.93880000	-1.28178800
H	-0.65815300	-0.89954000	-2.70018100
C	-2.31652600	-2.24626400	-2.19777400
H	-2.02149500	-3.26747400	-2.44290500
H	-2.84146600	-1.81904000	-3.05757100
C	-3.19261300	-2.16360100	-0.95004700
H	-4.23515700	-2.43535100	-1.11756300
H	-2.78410000	-2.80313100	-0.15975700
C	-1.87406900	1.85371700	0.07089500
H	-2.90620500	1.82741400	-0.29491700
H	-1.90253900	1.59317900	1.13611300
C	0.83832700	-0.33001800	0.81223000
H	0.95804000	0.57956000	1.42019900
C	2.14061100	-0.99620000	0.35116200
O	-0.10912100	-1.13637600	1.04001800
C	-3.37242000	-0.55087400	0.96142600

O	-2.40704600	-0.73198200	1.82821900
O	-4.52402600	-0.34529600	1.29960400
C	3.25298000	-0.14749300	-0.24033200
H	2.87458700	0.28631900	-1.17251900
C	4.52582800	-0.92695300	-0.57635400
H	4.98050800	-1.30835400	0.34223700
H	4.26081600	-1.80064400	-1.18534500
H	1.88110300	-1.82585800	-0.31189200
H	3.49087600	0.67955800	0.44113400
C	5.53145300	-0.05586600	-1.32671000
H	5.81017600	0.81851100	-0.72954600
H	6.44444800	-0.61304700	-1.55179900
H	5.11374900	0.30356500	-2.27277900
H	-1.43289100	-0.86203100	1.45631400
C	-1.33824900	3.27219900	-0.07091400
C	1.03071400	2.24705200	-0.79157000
H	-1.40463100	3.62228100	-1.10549200
H	-1.92029600	3.95014200	0.55532000
H	2.08156100	2.10272500	-0.53922200
H	1.00672500	2.76477700	-1.75773900
S	0.38515000	3.36980700	0.47893200
Cl	2.75984100	-1.80164700	1.87602600

(*R*)-**TS2**$_{G}$-Cl-P

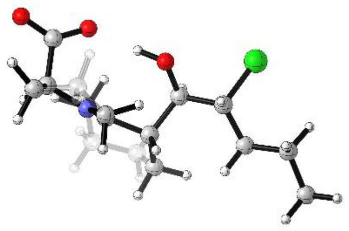

- Thermochemistry -

Zero-point correction=	0.413776 (Hartree/Particle)
Thermal correction to Energy=	0.431619
Thermal correction to Enthalpy=	0.432484
Thermal correction to Gibbs Free Energy=	0.369157
Sum of electronic and zero-point Energies=	-1365.227549
Sum of electronic and thermal Energies=	-1365.209706
Sum of electronic and thermal Enthalpies=	-1365.208841
Sum of electronic and thermal Free Energies=	-1365.272168

Number of Imaginary Frequencies = 0

E (Single Point Energy) [IEFPCM$_{(DCM)}$M06-2X/6-311++G(2d,2p)] = -1365.918402

C	-0.42826200	0.78308600	0.55196000
H	-0.61517200	0.22567200	1.47451500
C	1.06180600	0.89722400	0.33872000
N	1.86676800	0.03941200	0.86896300
C	3.31069900	-0.05899100	0.54981600
H	3.78097500	0.92014000	0.64868800
C	1.44839200	-1.10342300	1.73531200
H	0.55751800	-1.56636500	1.31583800
H	1.23921200	-0.70525200	2.73279600
C	2.66417100	-2.02603400	1.73260600
H	2.61435900	-2.70149100	0.87297100

H	2.71019700	-2.62544300	2.64254000
C	3.83654000	-1.05785200	1.57910400
H	4.04471100	-0.55157700	2.52696700
H	4.75239200	-1.52612200	1.21928200
C	1.55006200	1.95000000	-0.61309900
H	2.63648200	2.00989400	-0.62695000
H	1.25007900	1.60453500	-1.61340600
C	-0.98062700	-0.06670200	-0.65385100
H	-1.15548000	0.61467800	-1.49944600
C	-2.29359000	-0.79331500	-0.31147800
O	-0.10381900	-1.10950100	-1.00718900
C	3.51629400	-0.55633700	-0.92729900
O	2.49241100	-0.69056500	-1.64646800
O	4.70483900	-0.74878100	-1.22756600
C	-3.39976900	-0.02777900	0.39765700
H	-2.98589000	0.34329100	1.34361500
C	-4.62690300	-0.87887500	0.72844200
H	-5.10991200	-1.20543100	-0.19706000
H	-4.30288600	-1.78637900	1.25363900
H	-2.01956000	-1.67519600	0.27674700
H	-3.69993600	0.84300400	-0.19875400
C	-5.62665900	-0.10916000	1.58924400
H	-5.17783300	0.19130700	2.54166700
H	-6.50765700	-0.71752600	1.80937400
H	-5.96429900	0.79765600	1.07683500
H	0.78803300	-0.82066100	-1.31333500
C	0.91508700	3.31882900	-0.34401600
C	-1.06041700	2.18846800	0.69557900

H	1.25550800	3.70278900	0.62507400
H	1.26397900	4.01618400	-1.10969900
H	-2.14609800	2.10686100	0.68037700
H	-0.79275600	2.56903800	1.68885800
C	-0.60253000	3.19958000	-0.35399900
H	-1.06503600	4.16731900	-0.13944900
H	-0.94180400	2.90216600	-1.35410000
Cl	-2.93704300	-1.46885400	-1.87522000

(*S*)-**TS2**$_{G}$-Cl-Pre

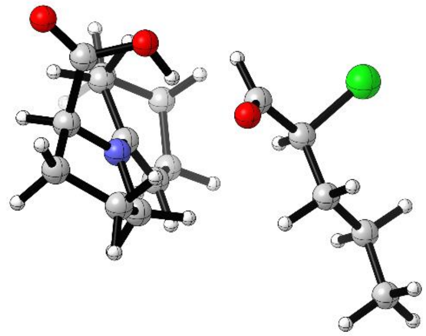

- Thermochemistry -

Zero-point correction= 0.409261 (Hartree/Particle)
Thermal correction to Energy= 0.428770
Thermal correction to Enthalpy= 0.429635
Thermal correction to Gibbs Free Energy= 0.361695
Sum of electronic and zero-point Energies= -1365.215140
Sum of electronic and thermal Energies= -1365.195630
Sum of electronic and thermal Enthalpies= -1365.194765
Sum of electronic and thermal Free Energies= -1365.262706

Number of Imaginary Frequencies = 0

E (Single Point Energy) [IEFPCM$_{(DCM)}$M06-2X/6-311++G(2d,2p)] = -1365.903542

C	0.05237000	0.97010300	-1.63577100
H	0.42964500	0.11032800	-2.18258300

C	-1.07187900	0.84898500	-0.89288200
N	-1.69974700	-0.38047800	-0.69187100
C	-3.11052600	-0.49027100	-0.33219900
H	-3.73533700	0.27834600	-0.79605400
C	-1.23261200	-1.57474500	-1.39529700
H	-0.19377300	-1.78556100	-1.12567600
H	-1.28874400	-1.43454500	-2.48670700
C	-2.19911500	-2.65387600	-0.92208400
H	-1.89674900	-3.01784700	0.06603100
H	-2.24311100	-3.50567300	-1.60291800
C	-3.52419300	-1.89243600	-0.84033500
H	-3.95652600	-1.78497600	-1.83888300
H	-4.27020900	-2.36144900	-0.19567500
C	-1.66536200	2.02561900	-0.14444000
H	-2.74979000	2.05666600	-0.29041200
H	-1.51250300	1.87399200	0.93482000
C	0.45413500	3.27130900	-0.70105300
C	0.95957400	-0.06020800	1.21981700
H	0.52403700	0.85409400	1.66086900
C	2.36011300	0.08756600	0.66036200
H	2.38361800	0.96257600	0.00483500
O	0.35908500	-1.11375700	1.23711400
C	-3.34632200	-0.38336900	1.17588500
O	-2.31637300	-0.66010800	1.97260400
O	-4.43281200	-0.10433800	1.63447100
H	-1.49441000	-0.83823400	1.46310400
C	2.88597600	-1.15023400	-0.03531100
H	2.14242900	-1.41478100	-0.79931500

C	4.24714900	-0.94957200	-0.69729600
H	4.18794200	-0.10114500	-1.39114100
H	2.91643700	-1.98246100	0.67694000
C	4.69940500	-2.20120600	-1.44639000
H	5.67516300	-2.05014900	-1.91507500
H	4.78102600	-3.05378400	-0.76463700
H	3.98471200	-2.46719500	-2.23192000
H	4.98927600	-0.68503800	0.06381200
Cl	3.37394100	0.55429200	2.09388900
C	0.81529100	2.26308100	-1.79048400
C	-1.06415400	3.36500900	-0.57576900
H	1.89451100	2.06046300	-1.78549300
H	0.60853300	2.70478300	-2.77605400
H	-1.48294700	3.65255400	-1.54844100
H	-1.35440700	4.13852200	0.14110600
H	0.87846800	2.95149800	0.26206400
H	0.88563800	4.25048500	-0.92964000

(*S*)-**TS2**$_{G}$-Cl

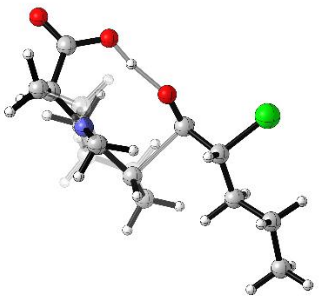

- Thermochemistry -

Zero-point correction= 0.409224 (Hartree/Particle)
Thermal correction to Energy= 0.427192
Thermal correction to Enthalpy= 0.428057

Thermal correction to Gibbs Free Energy=	0.364656
Sum of electronic and zero-point Energies=	-1365.206916
Sum of electronic and thermal Energies=	-1365.188948
Sum of electronic and thermal Enthalpies=	-1365.188083
Sum of electronic and thermal Free Energies=	-1365.251485

Number of Imaginary Frequencies = 1

E (Single Point Energy) [IEFPCM$_{(DCM)}$M06-2X/6-311++G(2d,2p)] = -1365.893626

C	0.14526000	1.09648300	-1.21944200
H	0.67265100	0.35042900	-1.80779400
C	-1.16406300	0.80656800	-0.84168300
N	-1.61902500	-0.45642300	-0.81262600
C	-2.93278200	-0.85481800	-0.29910500
H	-3.73017900	-0.23975700	-0.72123900
C	-0.89742600	-1.59586200	-1.41048000
H	0.14841500	-1.58079200	-1.11196800
H	-0.96859400	-1.53312800	-2.50448100
C	-1.63557800	-2.80826900	-0.85530900
H	-1.24740100	-3.04688400	0.14056300
H	-1.52329600	-3.68746100	-1.49147000
C	-3.08035000	-2.31643500	-0.76627700
H	-3.54486200	-2.32514300	-1.75639900
H	-3.70819800	-2.89482500	-0.08700800
C	-2.04454600	1.88426900	-0.25011900
H	-3.09446000	1.67165900	-0.46289200
H	-1.93690600	1.84519600	0.84514100
C	-0.19205100	3.54277500	-0.61084100
C	0.87600500	0.43927200	0.75861000
H	0.55956200	1.39377200	1.20298700
C	2.35879300	0.37992400	0.40862300

H	2.65512300	1.22761200	-0.20834700
O	0.29933900	-0.63026300	1.09270300
C	-3.08409600	-0.74900800	1.22784200
O	-2.01423000	-0.65945000	1.98016900
O	-4.20644700	-0.78119300	1.70007000
H	-1.07514300	-0.63364000	1.52179900
C	2.81532700	-0.93639500	-0.19189900
H	2.23489600	-1.08567600	-1.11207000
C	4.30162900	-0.98144700	-0.54208300
H	4.54696100	-0.13013000	-1.18975200
H	2.54884300	-1.75264300	0.48806600
C	4.67626000	-2.28767300	-1.23937800
H	5.74236600	-2.31644700	-1.47912500
H	4.45153700	-3.14747400	-0.59998800
H	4.11725800	-2.40916100	-2.17304600
H	4.89608900	-0.86620000	0.37006200
Cl	3.21211800	0.67755200	2.00151100
C	0.59511500	2.52791100	-1.43942500
C	-1.68705600	3.28307200	-0.75431800
H	1.66597100	2.62651700	-1.23065200
H	0.48297700	2.77255200	-2.50486800
H	-1.97385900	3.37193000	-1.80956000
H	-2.27060900	4.02144100	-0.19755100
H	0.08946300	3.47299200	0.44768700
H	0.05661500	4.55689600	-0.93723900

(*S*)-**TS2**$_{G}$-Cl-P

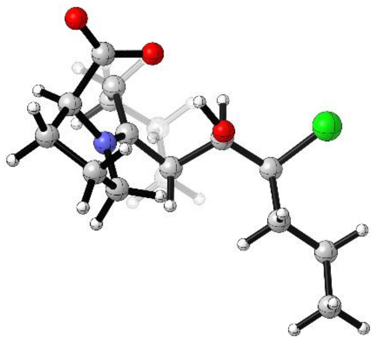

- Thermochemistry -

Zero-point correction=	0.413637 (Hartree/Particle)
Thermal correction to Energy=	0.431676
Thermal correction to Enthalpy=	0.432541
Thermal correction to Gibbs Free Energy=	0.368381
Sum of electronic and zero-point Energies=	-1365.234013
Sum of electronic and thermal Energies=	-1365.215975
Sum of electronic and thermal Enthalpies=	-1365.215110
Sum of electronic and thermal Free Energies=	-1365.279269

Number of Imaginary Frequencies = 0

E (Single Point Energy) [IEFPCM$_{(DCM)}$M06-2X/6-311++G(2d,2p)] = -1365.924651

C	0.34700600	0.72331500	-0.88219400
H	0.58558500	-0.11246900	-1.54112600
C	-1.13672900	0.76342700	-0.63658600
N	-1.86200400	-0.28923200	-0.81569500
C	-3.24627400	-0.42931200	-0.31017400
H	-3.85215300	0.42266200	-0.61868100
C	-1.37505500	-1.59396900	-1.36203700
H	-0.39943800	-1.82720900	-0.94169800
H	-1.30753000	-1.48661700	-2.44891600

C	-2.45826000	-2.58784100	-0.95188600
H	-2.24573100	-2.97406100	0.04971900
H	-2.50767400	-3.42904800	-1.64432200
C	-3.72842800	-1.73788300	-0.93497500
H	-4.08698800	-1.56010400	-1.95368900
H	-4.53898200	-2.16705900	-0.34593200
C	-1.69727500	2.02153600	-0.03834200
H	-2.78625000	2.01540100	-0.03841500
H	-1.39064400	2.01755400	1.01671100
C	0.36414800	3.28020600	-0.80762000
C	0.96673800	0.41211100	0.52849800
H	0.73411600	1.24583300	1.20446000
C	2.49196700	0.29914900	0.45503600
H	2.91066900	1.22554000	0.05697300
O	0.45371100	-0.79191200	1.03439700
C	-3.23393000	-0.49179200	1.26000700
O	-2.11854800	-0.41326700	1.84046800
O	-4.36113100	-0.61158300	1.76382400
H	-0.46345500	-0.65958100	1.37538900
C	2.99571700	-0.90125800	-0.33262400
H	2.56210400	-0.83717800	-1.34012900
C	4.51604000	-0.96994500	-0.46910100
H	4.88450300	-0.02179700	-0.88147100
H	2.60595700	-1.81642900	0.12635800
C	4.95045800	-2.12830200	-1.36485100
H	6.03922100	-2.17599800	-1.44996500
H	4.60245800	-3.08404800	-0.95976000
H	4.53786200	-2.02174400	-2.37353500

H	4.96768900	-1.08002900	0.52190900
Cl	3.11740600	0.23623500	2.16162100
C	0.83915100	2.02612800	-1.53976600
C	-1.16044500	3.28442500	-0.73095000
H	1.92857400	2.00124200	-1.62797000
H	0.45238200	2.04921600	-2.56571300
H	-1.57645300	3.34685600	-1.74369600
H	-1.52419000	4.15527600	-0.17997400
H	0.79074400	3.32782100	0.20203300
H	0.71453300	4.16874300	-1.34037300

(*R*)-**TS2**$_{O}$-Cl-Pre

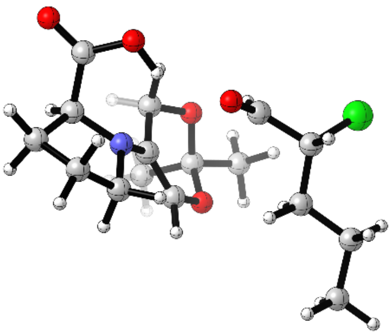

- Thermochemistry -

Zero-point correction=	0.416671 (Hartree/Particle)
Thermal correction to Energy=	0.438339
Thermal correction to Enthalpy=	0.439204
Thermal correction to Gibbs Free Energy=	0.365492
Sum of electronic and zero-point Energies=	-1515.593203
Sum of electronic and thermal Energies=	-1515.571536
Sum of electronic and thermal Enthalpies=	-1515.570671
Sum of electronic and thermal Free Energies=	-1515.644382

Number of Imaginary Frequencies = 0

E (Single Point Energy) [IEFPCM$_{(DCM)}$M06-2X/6-311++G(2d,2p)] = -1516.336462

C	-0.09864200	0.87494200	1.29663200
H	-0.30189600	0.26091000	2.16499600
C	0.96349300	0.72154000	0.48844600
N	1.91222100	-0.29654400	0.64097400
C	3.31194700	-0.04735700	0.28690900
H	3.57718800	1.01497900	0.31430500
C	1.81667600	-1.17651000	1.80666100
H	0.93624000	-1.82135000	1.71571300
H	1.72628500	-0.58939300	2.73521400
C	3.13966000	-1.93337500	1.77756200
H	3.09740100	-2.74093000	1.03884800
H	3.39460200	-2.36500500	2.74674900
C	4.12004600	-0.84261200	1.33670100
H	4.34749900	-0.18259400	2.17841000
H	5.06048500	-1.21949500	0.93119400
C	1.07565900	1.59227500	-0.73878900
H	1.86455800	2.35169900	-0.63805500
H	1.31243900	0.98210800	-1.61791700
O	-1.09479700	1.79524400	1.07070100
O	-0.17363900	2.20213900	-1.02065500
C	-0.79098000	2.80181400	0.10390300
C	-2.11893100	3.34220200	-0.37869800
H	-2.68159500	2.53895400	-0.86085800
H	-1.95223200	4.14925500	-1.09510900
C	0.09127300	3.87092100	0.73956900
H	-0.48266200	4.40324900	1.50087200
H	0.41468000	4.58192000	-0.02504100
H	0.97040400	3.43389500	1.21894000

C	-0.92989500	-1.15476600	-1.20622500
H	-0.84814700	-0.09463600	-1.50872300
C	-2.32101900	-1.64287100	-0.86545400
O	0.03734000	-1.88140700	-1.12407000
C	3.64571600	-0.54598000	-1.12361900
O	2.72731500	-1.28724700	-1.73489800
O	4.70979500	-0.30029900	-1.64885000
H	-2.69037800	3.72601000	0.46884200
C	-2.68519700	-1.24808100	0.56428000
H	-1.90753300	-1.67912400	1.21008200
C	-4.05485300	-1.75232400	1.01416100
H	-4.83185000	-1.31468500	0.37890100
H	-4.10301200	-2.83922900	0.87466200
H	-2.62231700	-0.15683600	0.66457300
C	-4.32358400	-1.40055800	2.47606600
H	-3.57463700	-1.85483200	3.13274500
H	-4.29019600	-0.31680400	2.62728900
H	-5.30821500	-1.75469700	2.79186100
H	1.91190100	-1.36373900	-1.18858000
H	-2.37187800	-2.72430000	-1.00767300
Cl	-3.45464700	-0.90191300	-2.06150100

(*R*)-**TS2**$_{O}$-Cl

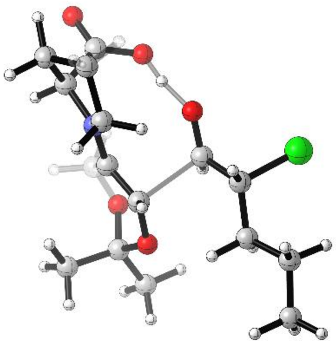

- Thermochemistry -

Zero-point correction=	0.415722 (Hartree/Particle)
Thermal correction to Energy=	0.435649
Thermal correction to Enthalpy=	0.436514
Thermal correction to Gibbs Free Energy=	0.369078
Sum of electronic and zero-point Energies=	-1515.578496
Sum of electronic and thermal Energies=	-1515.558569
Sum of electronic and thermal Enthalpies=	-1515.557704
Sum of electronic and thermal Free Energies=	-1515.625140

Number of Imaginary Frequencies = 1

E (Single Point Energy) [IEFPCM$_{(DCM)}$M06-2X/6-311++G(2d,2p)] = -1516.319381

C	-0.38108800	0.72799200	0.74598100
H	-0.65594100	0.23631800	1.67469400
C	0.95904600	0.84558900	0.38393800
N	1.88182000	-0.00350800	0.82618300
C	3.27547200	-0.01025900	0.37040700
H	3.66339100	1.00959100	0.29358500
C	1.65558500	-0.93250700	1.95102700
H	0.75547000	-1.52231600	1.77731200
H	1.54325300	-0.34887200	2.87391300

C	2.92479500	-1.77978400	1.95598400
H	2.81790600	-2.60683200	1.24633200
H	3.13624200	-2.19473100	2.94216700
C	3.99733300	-0.79823700	1.47674600
H	4.27217500	-0.11232500	2.28301200
H	4.90241700	-1.27509100	1.09987900
C	1.33540400	1.82887900	-0.69695600
H	2.04880200	2.56410300	-0.29631200
H	1.81415200	1.31117900	-1.53445100
O	-1.29348100	1.69830300	0.36824000
O	0.19099100	2.45526200	-1.23066400
C	-0.75386000	2.86175100	-0.25710700
C	-1.89509900	3.50672400	-1.01121900
H	-2.25335000	2.81909700	-1.78044700
H	-1.55288800	4.43126600	-1.48066300
C	-0.14240000	3.78452900	0.79280000
H	-0.94088900	4.19068800	1.41676100
H	0.37785600	4.60933600	0.29912900
H	0.56143800	3.26031100	1.44423600
C	-0.67784800	-0.88232600	-0.60364300
H	-0.84338700	-0.16083300	-1.42006700
C	-1.95618600	-1.49424800	-0.02418500
O	0.33481700	-1.64821700	-0.61457900
C	3.50507400	-0.65438800	-1.01377900
O	2.51071300	-1.22643200	-1.63673600
O	4.62842000	-0.59122500	-1.48175700
H	-2.70971600	3.73370300	-0.32068100
C	-3.14288400	-0.58244800	0.24086300

H	-2.84875100	0.12948700	1.01774400
C	-4.40186500	-1.31181600	0.71126300
H	-4.77645100	-1.96208200	-0.08454700
H	-4.14727700	-1.96299600	1.55757600
H	-3.35706900	0.01022400	-0.65738200
C	-5.49263400	-0.32610900	1.12641400
H	-5.15752200	0.30623500	1.95499300
H	-5.76031500	0.33045600	0.29195300
H	-6.39785000	-0.84919300	1.44580800
H	1.56697500	-1.32910600	-1.15019900
H	-1.68927100	-2.07878100	0.86094800
Cl	-2.43334800	-2.74799200	-1.27446000

(*R*)-**TS2**$_{O}$-Cl-P

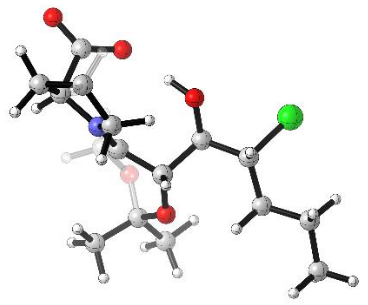

- Thermochemistry -

Zero-point correction= 0.419797 (Hartree/Particle)
Thermal correction to Energy= 0.439872
Thermal correction to Enthalpy= 0.440737
Thermal correction to Gibbs Free Energy= 0.372289
Sum of electronic and zero-point Energies= -1515.602231
Sum of electronic and thermal Energies= -1515.582156
Sum of electronic and thermal Enthalpies= -1515.581291
Sum of electronic and thermal Free Energies= -1515.649740

Number of Imaginary Frequencies = 0

E (Single Point Energy) [IEFPCM$_{(DCM)}$M06-2X/6-311++G(2d,2p)] = -1516.346706

C	-0.35892000	0.39160300	0.58381700
H	-0.42741400	0.01744300	1.61086700
C	1.07215400	0.69592900	0.22688700
N	2.05737200	0.09097300	0.78319000
C	3.43924200	0.15236800	0.26618200
H	3.69616100	1.18270300	0.00877300
C	1.96192300	-0.86940300	1.92529000
H	1.16279300	-1.58211100	1.73079300
H	1.74327400	-0.28092700	2.82135600
C	3.35243000	-1.50112000	1.97748600
H	3.38370100	-2.38468700	1.33298600
H	3.61030600	-1.80421900	2.99262000
C	4.26659100	-0.40693400	1.42035200
H	4.44575000	0.36903400	2.17084200
H	5.22338500	-0.77951700	1.05605100
C	1.28983300	1.65099600	-0.91596200
H	1.99070900	2.44328100	-0.61877500
H	1.72013400	1.10051600	-1.75861800
O	-1.20069900	1.52041400	0.49305700
O	0.05687500	2.17467800	-1.34546800
C	-0.76858300	2.63730700	-0.29389100
C	-2.00790200	3.21265500	-0.94334600
H	-2.42544600	2.47759200	-1.63495600
H	-1.75358500	4.12343900	-1.48962100
C	-0.05843800	3.63820800	0.61266000
H	-0.78852600	4.06221700	1.30454600

H	0.37720900	4.44392300	0.01607400
H	0.72866000	3.17011200	1.21103600
C	-0.81285600	-0.74082200	-0.41227300
H	-0.94270500	-0.26546200	-1.39547000
C	-2.13453600	-1.38001400	0.03153400
O	0.13101400	-1.77622000	-0.43360200
C	3.56685600	-0.72161700	-1.04418700
O	2.50272600	-1.16012000	-1.55431700
O	4.73189000	-0.85399600	-1.43875700
H	-2.74788500	3.44728400	-0.17562900
C	-3.28897000	-0.44923100	0.37112600
H	-2.95937400	0.19208500	1.19552300
C	-4.56597400	-1.17290900	0.79783500
H	-4.95951800	-1.75878200	-0.03811100
H	-4.32659100	-1.88583600	1.59756700
H	-3.49015400	0.21587600	-0.47796000
C	-5.62893300	-0.18862900	1.28250900
H	-5.27302800	0.38099300	2.14720300
H	-5.88462700	0.52633000	0.49347400
H	-6.54555000	-0.70773800	1.57478000
H	0.95205900	-1.53129800	-0.92529400
H	-1.91063800	-2.03633400	0.87913700
Cl	-2.63051000	-2.51183000	-1.30558500

(*S*)-**TS2**$_{O}$-Cl-Pre

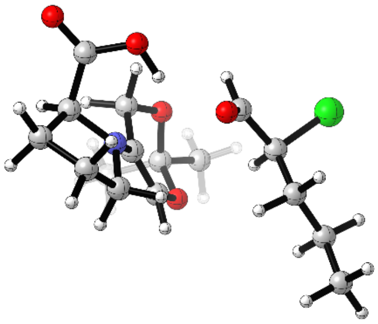

- Thermochemistry -

Zero-point correction=	0.416848 (Hartree/Particle)
Thermal correction to Energy=	0.438380
Thermal correction to Enthalpy=	0.439245
Thermal correction to Gibbs Free Energy=	0.366524
Sum of electronic and zero-point Energies=	-1515.594798
Sum of electronic and thermal Energies=	-1515.573266
Sum of electronic and thermal Enthalpies=	-1515.572401
Sum of electronic and thermal Free Energies=	-1515.645123

Number of Imaginary Frequencies = 0

E (Single Point Energy) [IEFPCM$_{(DCM)}$M06-2X/6-311++G(2d,2p)] = -1516.338534

C	0.14966400	0.78820100	-1.35516700
H	0.39091100	0.07899600	-2.13742500
C	-0.93330400	0.70623900	-0.56073200
N	-1.85252200	-0.34282800	-0.62145500
C	-3.24854800	-0.12755800	-0.23937900
H	-3.55891900	0.91915400	-0.32271400
C	-1.74196200	-1.31898800	-1.70685200
H	-0.84533700	-1.93190500	-1.56636900
H	-1.67047100	-0.80939800	-2.68144800

C	-3.04320300	-2.10709000	-1.59853800
H	-2.96466800	-2.86069500	-0.80755200
H	-3.30198700	-2.61269500	-2.53013200
C	-4.04973300	-1.01837800	-1.21444000
H	-4.31319100	-0.42481900	-2.09427200
H	-4.96994800	-1.39557900	-0.76515600
C	-1.10190300	1.72234400	0.54199200
H	-1.88129500	2.46100100	0.30736600
H	-1.38351600	1.23243500	1.48051800
O	1.12182700	1.75196500	-1.21540400
O	0.13759000	2.36352500	0.79456700
C	0.77295200	2.85407600	-0.37254200
C	2.07473700	3.47785500	0.07985000
H	2.66795400	3.76418300	-0.79080200
H	1.86889200	4.36496200	0.68234100
C	-0.11037900	3.82499400	-1.14832700
H	0.48071100	4.29115000	-1.93916100
H	-0.48120600	4.60241600	-0.47538000
H	-0.95809400	3.31571400	-1.61258600
C	0.80744200	-0.89323400	1.29708100
H	0.49216600	-0.11426200	2.01484500
C	2.18866900	-0.70100300	0.70701400
O	0.08963100	-1.83245200	1.02639300
C	-3.52426600	-0.54833300	1.20877900
O	-2.58177900	-1.25587000	1.82620100
O	-4.56645500	-0.27328300	1.76150200
H	2.63704300	2.75830600	0.68024700
C	2.61360100	-1.79542000	-0.25003200

H	2.66863700	-2.74921200	0.28772800
C	3.92728200	-1.49833900	-0.96902600
H	3.84104100	-0.53443700	-1.48679200
H	4.73151500	-1.39115500	-0.23280900
H	1.80471400	-1.89676700	-0.98595300
C	4.28624900	-2.59495500	-1.96931000
H	4.39405000	-3.56085000	-1.46543500
H	3.50765100	-2.69974700	-2.73191000
H	5.22826300	-2.37355700	-2.47758000
H	-1.79385600	-1.38475200	1.25194400
H	2.21523200	0.28567400	0.23109500
Cl	3.30300400	-0.56399500	2.13348700

(*S*)-**TS2**$_{\text{O}}$-Cl

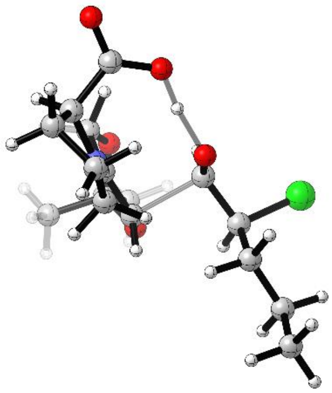

- Thermochemistry -

Zero-point correction=	0.415745 (Hartree/Particle)
Thermal correction to Energy=	0.435649
Thermal correction to Enthalpy=	0.436514
Thermal correction to Gibbs Free Energy=	0.369297
Sum of electronic and zero-point Energies=	-1515.584519
Sum of electronic and thermal Energies=	-1515.564615
Sum of electronic and thermal Enthalpies=	-1515.563750
Sum of electronic and thermal Free Energies=	-1515.630967

Number of Imaginary Frequencies = 1

E (Single Point Energy) [IEFPCM$_{(DCM)}$M06-2X/6-311++G(2d,2p)] = -1516.325570

C	0.26088900	0.82518300	-0.89223600
H	0.71619200	0.20962600	-1.66116800
C	-1.07694100	0.65062100	-0.54521700
N	-1.71001300	-0.50069700	-0.75226100
C	-3.05111500	-0.79936600	-0.24239700
H	-3.71623600	0.05974800	-0.36643800
C	-1.19512600	-1.56577100	-1.63731900
H	-0.17599100	-1.83123900	-1.35516900
H	-1.21044200	-1.20181200	-2.67263100
C	-2.18246200	-2.70828300	-1.41163400
H	-1.87098700	-3.29877600	-0.54377700
H	-2.24451500	-3.37099900	-2.27557900
C	-3.49747000	-1.98085400	-1.11982500
H	-3.93189900	-1.59389000	-2.04596700
H	-4.24309200	-2.59426000	-0.61343500
C	-1.75121700	1.72277100	0.27469000
H	-2.60937200	2.12955000	-0.27985500
H	-2.12123800	1.30863700	1.21860300
O	0.87303200	2.05950500	-0.77766400
O	-0.83372300	2.73384600	0.62882900
C	0.01761200	3.14928300	-0.42404600
C	0.91173000	4.22571800	0.14827500
H	1.66661100	4.50674800	-0.58857800
H	0.31491700	5.10334800	0.40450300
C	-0.76197100	3.61480000	-1.64934000

H	-0.06783800	4.06229400	-2.36329100
H	-1.49977400	4.36451700	-1.35229900
H	-1.27561500	2.78998900	-2.14968200
C	0.85791400	-0.17840000	0.83740300
H	0.58766500	0.68338900	1.46784800
C	2.34089700	-0.22067000	0.48330500
O	0.24888000	-1.28599900	0.98091700
C	-3.10506100	-1.16912400	1.25567700
O	-1.99725500	-1.32438400	1.92637700
O	-4.20747300	-1.30043800	1.75982100
H	1.40464600	3.84620800	1.04592800
C	2.74547400	-1.43356800	-0.33451700
H	2.50061000	-2.34013600	0.22893700
C	4.21203100	-1.43610800	-0.76073800
H	4.43452500	-0.50599800	-1.29873100
H	4.85008500	-1.44660900	0.12932400
H	2.11423000	-1.44655000	-1.23397100
C	4.54091700	-2.63744300	-1.64468300
H	4.34053900	-3.57505300	-1.11621100
H	3.93502300	-2.63099200	-2.55677700
H	5.59348400	-2.63586800	-1.93963700
H	-1.03995400	-1.26992300	1.43736300
H	2.64930400	0.71746400	0.01935000
Cl	3.21205400	-0.23220600	2.09156100

(*S*)-**TS2**$_{O}$-Cl-P

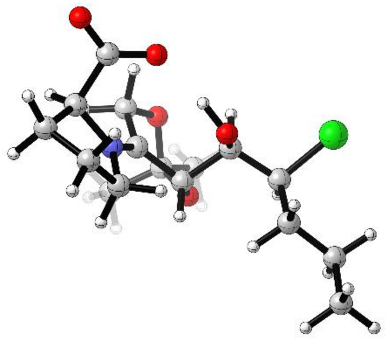

- Thermochemistry -

Zero-point correction=	0.419829 (Hartree/Particle)
Thermal correction to Energy=	0.440024
Thermal correction to Enthalpy=	0.440889
Thermal correction to Gibbs Free Energy=	0.372020
Sum of electronic and zero-point Energies=	-1515.606058
Sum of electronic and thermal Energies=	-1515.585863
Sum of electronic and thermal Enthalpies=	-1515.584998
Sum of electronic and thermal Free Energies=	-1515.653866

Number of Imaginary Frequencies = 0

E (Single Point Energy) [IEFPCM$_{(DCM)}$M06-2X/6-311++G(2d,2p)] = -1516.350766

C	0.34353600	0.55156400	-0.63126600
H	0.55899100	-0.04605800	-1.52027600
C	-1.13398700	0.61065300	-0.37573900
N	-1.92385500	-0.32858600	-0.75237300
C	-3.29487000	-0.48201100	-0.22776600
H	-3.80497100	0.48420300	-0.21762400
C	-1.56270700	-1.48181900	-1.63458400
H	-0.61941800	-1.91283400	-1.30498400
H	-1.47090700	-1.08823000	-2.65138200

C	-2.75414000	-2.42754800	-1.48991600
H	-2.58764200	-3.10945300	-0.65060900
H	-2.89885400	-3.02055900	-2.39334800
C	-3.92493900	-1.48926200	-1.18626900
H	-4.26594400	-0.98669700	-2.09640700
H	-4.77238800	-1.98526000	-0.71408600
C	-1.61833100	1.74508700	0.48554700
H	-2.46362100	2.25242000	0.00039400
H	-1.95625100	1.34275900	1.44630600
O	0.95176600	1.81200400	-0.80710800
O	-0.55756600	2.63096800	0.75558900
C	0.22491600	2.96983600	-0.37491500
C	1.25466200	3.96973000	0.10190800
H	1.96956900	4.16911500	-0.69857300
H	0.76288300	4.90229600	0.38619400
C	-0.61715300	3.49466100	-1.53322800
H	0.05124000	3.86395200	-2.31309600
H	-1.25580700	4.31389400	-1.19313400
H	-1.24334700	2.71597600	-1.97790500
C	0.93657700	-0.15652300	0.64488300
H	0.71914600	0.50222600	1.49727600
C	2.45498600	-0.26568000	0.49613100
O	0.38590100	-1.42930100	0.81183500
C	-3.23769900	-1.02549200	1.25500500
O	-2.10573100	-1.09831100	1.80196400
O	-4.34833500	-1.30532400	1.72265600
H	1.78277100	3.55778000	0.96474600
C	2.90868500	-1.34096300	-0.48038100

H	2.57650300	-2.31816700	-0.11440500
C	4.41395500	-1.34569600	-0.74238100
H	4.72552700	-0.34827500	-1.07731800
H	4.94612300	-1.54574100	0.19344400
H	2.39065800	-1.16569800	-1.43355700
C	4.80240100	-2.38855700	-1.78841500
H	4.51358200	-3.39278000	-1.46171800
H	4.30500200	-2.19098400	-2.74373600
H	5.88125600	-2.38982100	-1.96470600
H	-0.50701500	-1.35638800	1.22799300
H	2.85115700	0.71283700	0.21787600
Cl	3.14677100	-0.59815200	2.14518100

(*R*)-**TS2**$_{T}$-Cl-Pre

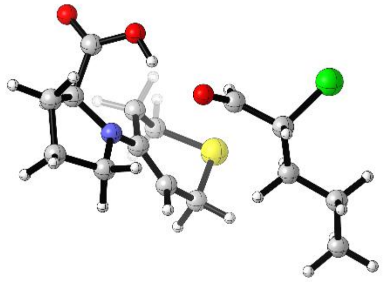

- Thermochemistry -

Zero-point correction= 0.381924 (Hartree/Particle)
Thermal correction to Energy= 0.401833
Thermal correction to Enthalpy= 0.402698
Thermal correction to Gibbs Free Energy= 0.333340
Sum of electronic and zero-point Energies= -1724.096560
Sum of electronic and thermal Energies= -1724.076651
Sum of electronic and thermal Enthalpies= -1724.075786
Sum of electronic and thermal Free Energies= -1724.145143

Number of Imaginary Frequencies = 0

E (Single Point Energy) [IEFPCM$_{(DCM)}$M06-2X/6-311++G(2d,2p)] = -1724.781246

C	0.01618300	1.42209700	1.14834400
H	-0.16972900	0.93081700	2.09797400
C	1.10800000	1.07820100	0.42630200
N	1.97370200	0.06833600	0.84449100
C	3.28682800	-0.16071000	0.25397300
H	3.93055200	0.72878800	0.27091700
C	1.90196600	-0.44169900	2.21956500
H	1.15055600	-1.23895300	2.28783000
H	1.61187300	0.36055300	2.90643700
C	3.31809900	-0.93850500	2.52001600
H	3.32659000	-1.79341600	3.19838500
H	3.90376500	-0.13357700	2.97440300
C	3.88094500	-1.27227900	1.14061100
H	4.97072200	-1.28161000	1.09350400
H	3.50898200	-2.24754300	0.80645400
C	1.43995200	1.69534200	-0.91864200
H	2.50794400	1.94192900	-0.95144000
H	1.26465300	0.95240600	-1.70776900
C	-0.90634500	-1.41360700	-0.49387900
H	-0.75150300	-0.60526000	-1.23255700
C	-2.33891400	-1.67097000	-0.08809700
O	0.02097000	-2.05939600	-0.05074300
C	3.24744400	-0.65726500	-1.18636400
O	2.23255100	-1.45247100	-1.51676700
O	4.12422800	-0.39988200	-1.98136600
C	-2.96155500	-0.44210300	0.55788600
H	-2.34491400	-0.20911400	1.43705600

C	-4.41432900	-0.62557500	0.99049900
H	-5.03151700	-0.85043600	0.11469100
H	-4.48460200	-1.48988000	1.66193200
H	-2.38386800	-2.55035600	0.55680500
H	-2.87117000	0.40442800	-0.13583200
C	-4.94399100	0.62480800	1.68984100
H	-4.89818300	1.49205400	1.02275100
H	-5.98404100	0.49351800	1.99875800
H	-4.35310500	0.85521400	2.58251000
H	1.58052400	-1.56669500	-0.78520900
C	0.66417600	2.96359300	-1.25096900
C	-0.99555100	2.47344900	0.77676500
H	0.99373300	3.79865300	-0.62477500
H	0.82640600	3.23837100	-2.29461500
H	-1.99140400	2.17690900	1.11807900
H	-0.77390800	3.43546900	1.25486700
S	-1.12413300	2.75086800	-1.01525000
Cl	-3.18868100	-2.10877500	-1.62604100

(*R*)-**TS2**$_{T}$-Cl

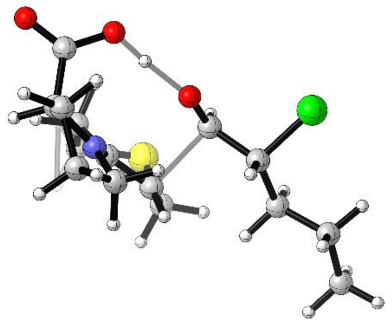

- Thermochemistry -

Zero-point correction= 0.381847 (Hartree/Particle)
Thermal correction to Energy= 0.399956
Thermal correction to Enthalpy= 0.400821
Thermal correction to Gibbs Free Energy= 0.337245
Sum of electronic and zero-point Energies= -1724.084194
Sum of electronic and thermal Energies= -1724.066085
Sum of electronic and thermal Enthalpies= -1724.065220
Sum of electronic and thermal Free Energies= -1724.128796

Number of Imaginary Frequencies = 1

E (Single Point Energy) [IEFPCM$_{(DCM)}$M06-2X/6-311++G(2d,2p)] = -1724.766523

C	0.30963300	0.91748400	-0.88416900
H	0.73216900	0.24609600	-1.62800000
C	-1.06897400	0.80494900	-0.66539500
N	-1.69478400	-0.34157400	-0.94909500
C	-3.06228100	-0.68739000	-0.53327800
H	-3.79416700	-0.07397300	-1.06971400
C	-1.11659000	-1.37666600	-1.83033900
H	-0.35526700	-1.93880000	-1.28178800
H	-0.65815300	-0.89954000	-2.70018100
C	-2.31652600	-2.24626400	-2.19777400
H	-2.02149500	-3.26747400	-2.44290500
H	-2.84146600	-1.81904000	-3.05757100
C	-3.19261300	-2.16360100	-0.95004700
H	-4.23515700	-2.43535100	-1.11756300
H	-2.78410000	-2.80313100	-0.15975700
C	-1.87406900	1.85371700	0.07089500
H	-2.90620500	1.82741400	-0.29491700
H	-1.90253900	1.59317900	1.13611300
C	0.83832700	-0.33001800	0.81223000
H	0.95804000	0.57956000	1.42019900

C	2.14061100	-0.99620000	0.35116200
O	-0.10912100	-1.13637600	1.04001800
C	-3.37242000	-0.55087400	0.96142600
O	-2.40704600	-0.73198200	1.82821900
O	-4.52402600	-0.34529600	1.29960400
C	3.25298000	-0.14749300	-0.24033200
H	2.87458700	0.28631900	-1.17251900
C	4.52582800	-0.92695300	-0.57635400
H	4.98050800	-1.30835400	0.34223700
H	4.26081600	-1.80064400	-1.18534500
H	1.88110300	-1.82585800	-0.31189200
H	3.49087600	0.67955800	0.44113400
C	5.53145300	-0.05586600	-1.32671000
H	5.81017600	0.81851100	-0.72954600
H	6.44444800	-0.61304700	-1.55179900
H	5.11374900	0.30356500	-2.27277900
H	-1.43289100	-0.86203100	1.45631400
C	-1.33824900	3.27219900	-0.07091400
C	1.03071400	2.24705200	-0.79157000
H	-1.40463100	3.62228100	-1.10549200
H	-1.92029600	3.95014200	0.55532000
H	2.08156100	2.10272500	-0.53922200
H	1.00672500	2.76477700	-1.75773900
S	0.38515000	3.36980700	0.47893200
Cl	2.75984100	-1.80164700	1.87602600

(*R*)-**TS2**$_{T}$-Cl-P

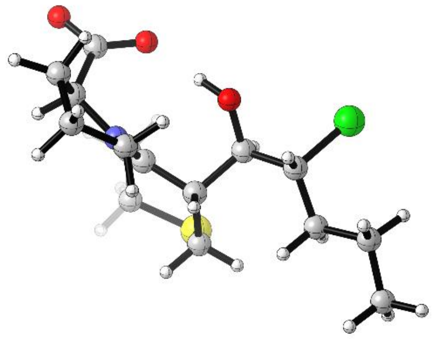

- Thermochemistry -

Zero-point correction=	0.386187 (Hartree/Particle)
Thermal correction to Energy=	0.404305
Thermal correction to Enthalpy=	0.405170
Thermal correction to Gibbs Free Energy=	0.341075
Sum of electronic and zero-point Energies=	-1724.108059
Sum of electronic and thermal Energies=	-1724.089941
Sum of electronic and thermal Enthalpies=	-1724.089076
Sum of electronic and thermal Free Energies=	-1724.153172

Number of Imaginary Frequencies = 0

E (Single Point Energy) [IEFPCM$_{(DCM)}$M06-2X/6-311++G(2d,2p)] = -1724.794106

C	-0.38251300	0.50377500	0.68483500
H	-0.55863200	-0.17159800	1.52673300
C	1.10523100	0.65497200	0.46826700
N	1.91503000	-0.26057000	0.88508900
C	3.33213300	-0.38310600	0.43851000
H	3.90202700	0.45229900	0.85286600
C	1.54339800	-1.40811900	1.75989400
H	1.02267300	-2.14188800	1.13721900
H	0.88873600	-1.06683200	2.56040300

C	2.89180700	-1.91345800	2.25541400
H	2.83129800	-2.95144800	2.58492900
H	3.23564300	-1.29978200	3.09361700
C	3.79271700	-1.72090900	1.03848200
H	4.85552800	-1.69466300	1.27769000
H	3.62128100	-2.52376400	0.31379000
C	1.61736000	1.79090200	-0.37723600
H	2.70570300	1.84464400	-0.32550800
H	1.36010800	1.55399900	-1.41733900
C	-0.92616300	-0.21670600	-0.61048400
H	-1.06930800	0.54948700	-1.38553900
C	-2.25313900	-0.95859100	-0.36720200
O	-0.04832600	-1.23058100	-1.02769000
C	3.47984800	-0.40636400	-1.11225200
O	2.44852200	-0.64662900	-1.79441400
O	4.64257700	-0.22651400	-1.50661300
C	-3.36892800	-0.25318200	0.38751900
H	-2.98704100	-0.02858800	1.39149100
C	-4.63510900	-1.09429400	0.55904100
H	-5.09496200	-1.27193900	-0.41712800
H	-4.36040400	-2.07691800	0.96322300
H	-1.99924100	-1.89559900	0.13865700
H	-3.61647800	0.69850600	-0.10047200
C	-5.63818500	-0.41138500	1.48658900
H	-5.92543700	0.56965300	1.09428500
H	-6.54707500	-1.00917600	1.59244100
H	-5.21451100	-0.26234800	2.48514100
H	0.82440400	-0.91147200	-1.36751300

C	1.03439200	3.15813200	-0.01348700
C	-1.04196600	1.85274800	1.03579800
H	1.31582300	3.45395100	1.00169300
H	1.43119200	3.90267600	-0.70508700
H	-2.11881400	1.74684300	1.13293700
H	-0.66459900	2.18650800	2.00783800
S	-0.76840900	3.17731400	-0.16923400
Cl	-2.85859100	-1.47072300	-2.00568500

(*S*)-**TS2**$_{T}$-Cl-Pre

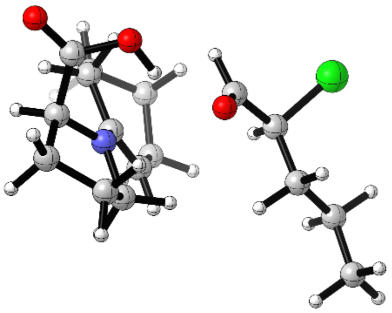

- Thermochemistry -

Zero-point correction= 0.381949 (Hartree/Particle)
Thermal correction to Energy= 0.401722
Thermal correction to Enthalpy= 0.402587
Thermal correction to Gibbs Free Energy= 0.334442
Sum of electronic and zero-point Energies= -1724.098616
Sum of electronic and thermal Energies= -1724.078843
Sum of electronic and thermal Enthalpies= -1724.077978
Sum of electronic and thermal Free Energies= -1724.146124

Number of Imaginary Frequencies = 0

E (Single Point Energy) [IEFPCM$_{(DCM)}$M06-2X/6-311++G(2d,2p)] = -1724.783329

C	0.02828800	0.71964900	-1.59488600
H	0.44654900	-0.16789900	-2.05916400
C	-1.13370400	0.62161000	-0.90273900

N	-1.76560700	-0.60091200	-0.71715000
C	-3.09755300	-0.75081700	-0.14363100
H	-3.86521700	-0.18577300	-0.68803000
C	-1.38083100	-1.76955200	-1.51900600
H	-0.52843400	-2.28279100	-1.05499400
H	-1.08606700	-1.45488600	-2.52562600
C	-2.64098700	-2.63622000	-1.55292600
H	-2.41422700	-3.70160200	-1.62011400
H	-3.25694900	-2.35973900	-2.41412100
C	-3.36180900	-2.26517800	-0.25899900
H	-4.43032200	-2.48432300	-0.26446600
H	-2.90360800	-2.78500200	0.59002300
C	-1.78982400	1.80182600	-0.21305900
H	-2.86530400	1.79173900	-0.42654500
H	-1.68514600	1.68763300	0.87392800
C	0.92283500	-0.20822800	1.19250500
H	0.46831100	0.71535700	1.59150400
C	2.33816500	-0.06827500	0.67466200
O	0.33359500	-1.27055700	1.22359400
C	-3.20596400	-0.34610600	1.32249600
O	-2.14826500	-0.58176000	2.09560500
O	-4.22699000	0.11149700	1.78626800
C	2.87098400	-1.30369900	-0.02043100
H	2.87994500	-2.14086800	0.68657800
C	4.24945600	-1.10733000	-0.64686400
H	4.21213600	-0.25780900	-1.34073900
H	4.97247800	-0.84651500	0.13372600
H	2.14290900	-1.56017600	-0.80216600

C	4.71697400	-2.35934700	-1.38585100
H	5.70447500	-2.21032200	-1.82996900
H	4.77943700	-3.21280800	-0.70319600
H	4.02139200	-2.62273300	-2.18925100
H	-1.37202000	-0.94041100	1.60224400
H	2.39064500	0.82098200	0.04163300
C	-1.24698400	3.16547500	-0.62202500
C	0.80980200	1.98362500	-1.83651200
H	1.88203300	1.76602300	-1.86819200
H	0.55926800	2.43917200	-2.80232700
H	-1.55090800	3.41691200	-1.64293200
H	-1.63377200	3.93713200	0.04567100
S	0.56684700	3.23522700	-0.53981600
Cl	3.31072200	0.36137700	2.14844100

(*S*)-**TS2**$_{T}$-Cl

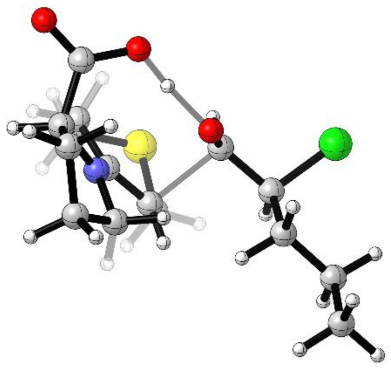

- Thermochemistry -

Zero-point correction= 0.381630 (Hartree/Particle)
Thermal correction to Energy= 0.399823
Thermal correction to Enthalpy= 0.400688
Thermal correction to Gibbs Free Energy= 0.336784
Sum of electronic and zero-point Energies= -1724.091371

Sum of electronic and thermal Energies= -1724.073178
Sum of electronic and thermal Enthalpies= -1724.072313
Sum of electronic and thermal Free Energies= -1724.136217

Number of Imaginary Frequencies = 1

E (Single Point Energy) [IEFPCM$_{(DCM)}$M06-2X/6-311++G(2d,2p)] = -1724.773577

C	0.10934700	0.88658400	-1.17047900
H	0.69330200	0.14985400	-1.71636600
C	-1.18943600	0.51689300	-0.81099100
N	-1.52897600	-0.77679800	-0.75967200
C	-2.75129200	-1.30173500	-0.13462200
H	-3.63843600	-0.99073300	-0.69641700
C	-0.76287000	-1.84384200	-1.43691200
H	0.11750500	-2.09859500	-0.83984600
H	-0.44497200	-1.49393100	-2.42251800
C	-1.75643800	-3.00026600	-1.50863700
H	-1.25516800	-3.96744700	-1.56727200
H	-2.40273100	-2.88811600	-2.38452400
C	-2.56809700	-2.82786300	-0.22641800
H	-3.53024600	-3.34069800	-0.23425200
H	-1.98924100	-3.17994800	0.63465100
C	-2.19189100	1.51176600	-0.26820200
H	-3.20313500	1.16369700	-0.50266700
H	-2.10666600	1.53527800	0.82627400
C	0.88254500	0.37143300	0.78475300
H	0.54032200	1.33455100	1.18963400
C	2.36288500	0.33484500	0.41686600
O	0.35417900	-0.70685500	1.18093500
C	-2.97806600	-0.89725300	1.32576200

O	-1.93500000	-0.68181400	2.08810900
O	-4.11912400	-0.85196300	1.74942800
C	2.84150200	-1.00026700	-0.12335000
H	2.62780700	-1.78062600	0.61473200
C	4.31494600	-1.01854800	-0.52526400
H	4.50742800	-0.20382800	-1.23486500
H	4.93651600	-0.82407400	0.35495300
H	2.23059400	-1.22729000	-1.00746300
C	4.71144200	-2.35434500	-1.15083100
H	5.76881300	-2.36334300	-1.42780900
H	4.53987000	-3.17760600	-0.44987500
H	4.12466700	-2.55493600	-2.05332800
H	-0.97498300	-0.70379900	1.64541800
H	2.63042800	1.15522400	-0.24875600
C	-2.02539600	2.92704600	-0.80952800
C	0.49316100	2.31232800	-1.49913200
H	1.56189900	2.47354400	-1.33734500
H	0.30532200	2.52522900	-2.55785500
H	-2.22777300	2.96478100	-1.88416300
H	-2.72618900	3.59730900	-0.30926200
S	-0.35718100	3.56658400	-0.49792500
Cl	3.23401700	0.73483200	1.97456200

(*S*)-**TS2**$_{T}$-Cl-P

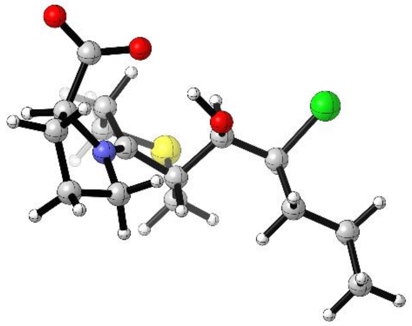

- Thermochemistry -

Zero-point correction=	0.385949 (Hartree/Particle)
Thermal correction to Energy=	0.404276
Thermal correction to Enthalpy=	0.405141
Thermal correction to Gibbs Free Energy=	0.340480
Sum of electronic and zero-point Energies=	-1724.115711
Sum of electronic and thermal Energies=	-1724.097383
Sum of electronic and thermal Enthalpies=	-1724.096518
Sum of electronic and thermal Free Energies=	-1724.161179

Number of Imaginary Frequencies = 0

E (Single Point Energy) [IEFPCM$_{(DCM)}$M06-2X/6-311++G(2d,2p)] = -1724.801604

C	0.32359000	0.47851400	-0.84354000
H	0.54565800	-0.38552900	-1.47169000
C	-1.15912700	0.55972100	-0.60423100
N	-1.89944600	-0.48326300	-0.78235700
C	-3.24980100	-0.63919000	-0.17934600
H	-3.93386500	0.08017500	-0.63534400
C	-1.48566300	-1.74048200	-1.46709400
H	-0.87772700	-2.32151200	-0.76755000
H	-0.90266300	-1.50126300	-2.35527400

C	-2.82019400	-2.40869800	-1.76999800
H	-2.70086200	-3.47989100	-1.93733500
H	-3.26862600	-1.96263300	-2.66295200
C	-3.64723700	-2.08376500	-0.52766000
H	-4.72273200	-2.16687100	-0.68224800
H	-3.36435000	-2.74925600	0.29454100
C	-1.72747700	1.80962500	0.01128600
H	-2.81819300	1.79117100	-0.02536600
H	-1.44603900	1.81048200	1.07106000
C	0.91327100	0.15517400	0.58015000
H	0.64182500	0.96816900	1.26497700
C	2.44264800	0.08852300	0.54067300
O	0.40560400	-1.07123500	1.02717700
C	-3.21092200	-0.43662400	1.36611800
O	-2.08681400	-0.51538600	1.93146300
O	-4.32581400	-0.26399100	1.88182700
C	3.00362800	-1.02915700	-0.32650800
H	2.60171900	-1.98702000	0.02263200
C	4.52975900	-1.07482800	-0.38877500
H	4.90923400	-0.08678900	-0.67959100
H	4.93007400	-1.28553500	0.60775900
H	2.62423700	-0.87406900	-1.34641300
C	5.02241600	-2.13099700	-1.37603600
H	6.11455300	-2.16561500	-1.40543100
H	4.66185300	-3.12528000	-1.09309300
H	4.66451100	-1.92050000	-2.38924900
H	-0.49331200	-0.93087300	1.41264600
H	2.84022800	1.05816600	0.23156400

C	-1.23056700	3.09624000	-0.65873900
C	0.89479000	1.72398200	-1.53261400
H	1.97532700	1.62777600	-1.65402000
H	0.46719200	1.81499700	-2.53570400
H	-1.57297100	3.15397300	-1.69635100
H	-1.64050700	3.95261800	-0.12142200
S	0.57669900	3.26146100	-0.62217800
Cl	3.01740500	-0.08933900	2.25407900

Supplementary Table 8. Energies for enamine addition to 2-fluoropentanal. Reported Gibbs free energies for structures optimized at the IEFPCM$_{(DCM)}$M06-2X/6-311++G(2d,2p)//IEFPCM$_{(DCM)}$M06-2X/6-31+G(d,p) level of theory represent the sum of the thermal correction to Gibbs free energy computed at the IEFPCM$_{(DCM)}$M06-2X/6-31+G(d,p) level of theory and single point electronic energies computed at the IEFPCM$_{(DCM)}$M06-2X/6-311++G(2d,2p). All energies are reported in Hartrees.

**Structure**	**Single Point Energies, E IEFPCM$_{(DCM)}$M06-2X/6-311++G(2d,2p)**	**Thermal Corrections to Gibbs Free Energies, IEFPCM$_{(DCM)}$M06-2X/6-31+G(d,p)**	**Gibbs Free Energies (G), IEFPCM$_{(DCM)}$M06-2X/6-31+G(d,p)**	**Gibbs Free Energies (G), IEFPCM$_{(DCM)}$M06-2X/6-311++G(2d,2p)// IEFPCM$_{(DCM)}$M06-2X/6-31+G(d,p)**
2-Fluoropentanal	-370.97333955	0.104800	-370.761392	-370.8685396
Enamine of Cyclohexanone (G)	-634.55147535	0.238392	-634.142903	-634.3130834
Enamine of Dioxane (O)	-784.98483791	0.244077	-784.522619	-784.7407609
Enamine of Tetrahydro-4H-thiopyranone (T)	-993.43210840	0.210205	-993.028735	-993.2219034
Enamine of Tetrahydro-4H-pyranone (P)	-670.45469021	0.214793	-670.055312	-670.2398972
(*R*)-**TS2**$_{P}$-F-Pre	-1041.446105	0.339912	-1040.814983	-1041.106193
(*R*)-**TS2**$_{P}$-F	-1041.429464	0.343633	-1040.796259	-1041.085831
(*R*)-**TS2**$_{P}$-F-P	-1041.461821	0.34743	-1040.824973	-1041.114382
(*S*)-**TS2**$_{P}$-F-Pre	-1041.445581	0.338546	-1040.815816	-1041.107035
(*S*)-**TS2**$_{P}$-F	-1041.434367	0.34289	-1040.801762	-1041.091477
(*S*)-**TS2**$_{P}$-F-P	-1041.465939	0.347487	-1040.82909	-1041.118452
(*R*)-**TS2**$_{G}$-F-Pre	-1005.542966	0.364056	-1004.901994	-1005.17891
(*R*)-**TS2**$_{G}$-F	-1005.526459	0.367328	-1004.883971	-1005.159131
(*R*)-**TS2**$_{G}$-F-P	-1005.55985	0.371357	-1004.913474	-1005.188493
(*S*)-**TS2**$_{G}$-F-Pre	-1005.542429	0.363048	-1004.902509	-1005.179381
(*S*)-**TS2**$_{G}$-F	-1005.5316	0.366632	-1004.889686	-1005.164968
(*S*)-**TS2**$_{G}$-F-P	-1005.56497	0.370935	-1004.919076	-1005.194035
(*R*)-**TS2**$_{O}$-F-Pre	-1155.977111	0.368522	-1155.283743	-1155.608589

(*R*)-**TS2**$_O$-F	-1155.958868	0.371067	-1155.264945	-1155.587801
(*R*)-**TS2**$_O$-F-P	-1155.987905	0.374936	-1155.290267	-1155.612969
(*S*)-**TS2**$_O$-F-Pre	-1155.97545	0.368391	-1155.28244	-1155.607059
(*S*)-**TS2**$_O$-F	-1155.963577	0.371738	-1155.268666	-1155.591839
(*S*)-**TS2**$_O$-F-P	-1155.990832	0.373799	-1155.294111	-1155.617033
(*R*)-**TS2**$_T$-F-Pre	-1364.422147	0.335876	-1363.786031	-1364.086271
(*R*)-**TS2**$_T$-F	-1364.406058	0.339298	-1363.768554	-1364.06676
(*R*)-**TS2**$_T$-F-P	-1364.406058	0.339298	-1363.768554	-1364.06676
(*S*)-**TS2**$_T$-F-Pre	-1364.421894	0.335488	-1363.786192	-1364.086406
(*S*)-**TS2**$_T$-F	-1364.411596	0.338699	-1363.774512	-1364.072897
(*S*)-**TS2**$_T$-F-P	-1364.441868	0.342929	-1363.80105	-1364.098939

Pre – Precomplex

P – Product

(*R*)-**TS2**$_P$-F-Pre

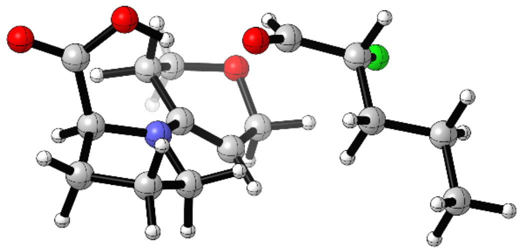

- Thermochemistry -

Zero-point correction= 0.386791 (Hartree/Particle)
Thermal correction to Energy= 0.405842
Thermal correction to Enthalpy= 0.406707
Thermal correction to Gibbs Free Energy= 0.339912
Sum of electronic and zero-point Energies= -1040.768103
Sum of electronic and thermal Energies= -1040.749052
Sum of electronic and thermal Enthalpies= -1040.748187
Sum of electronic and thermal Free Energies= -1040.814983

Number of Imaginary Frequencies = 0

E (Single Point Energy) [IEFPCM$_{(DCM)}$M06-2X/6-311++G(2d,2p)] = -1041.446105

C	0.31310000	1.12235000	-1.39447400
H	0.83282500	0.35114700	-1.95381800
C	-0.86092700	0.86958400	-0.77940900
N	-1.39573900	-0.40785000	-0.66350500
C	-2.82857100	-0.64769400	-0.51612700
H	-3.44097400	0.08202000	-1.05505600
C	-0.72088000	-1.54700700	-1.28372300
H	0.28024900	-1.66168700	-0.85604500
H	-0.62035300	-1.40164700	-2.37104800
C	-1.65381200	-2.71072000	-0.96903200
H	-1.48726200	-3.05707200	0.05681600
H	-1.51412400	-3.55548800	-1.64556700
C	-3.03418300	-2.06289500	-1.10569700
H	-3.29816600	-1.96505300	-2.16237800
H	-3.83647300	-2.60481600	-0.60180500
C	-1.58830200	1.97455300	-0.04835200
H	-2.65522500	1.97448400	-0.29758400
H	-1.51664300	1.80812100	1.03577200
C	1.16020500	0.30706500	1.47249900
H	0.81884100	1.35781300	1.47493300
C	2.64674600	0.10019400	1.27330800
O	0.40642600	-0.62516800	1.66917800
C	-3.29867700	-0.60911700	0.94094800
O	-2.37023700	-0.67508100	1.88927400
O	-4.47709600	-0.55922600	1.22115100

C	2.98743000	-0.97308800	0.25765400
H	2.43118700	-1.87594500	0.53772000
C	4.48429800	-1.26938300	0.18648600
H	5.02496400	-0.34766800	-0.05426600
H	4.83302700	-1.59364200	1.17455300
H	3.08381500	-0.13622000	2.25345100
H	2.61580400	-0.64965400	-0.72292000
C	4.79940100	-2.34274700	-0.85290000
H	4.28332400	-3.27878400	-0.61591800
H	5.87173600	-2.55011100	-0.89498800
H	4.47830500	-2.02567300	-1.85036900
H	-1.45809500	-0.66404400	1.52089500
C	-0.99591500	3.33191500	-0.40957500
C	0.97044700	2.47762800	-1.33366200
H	-1.29124800	3.61584000	-1.43089500
H	-1.34247900	4.10372500	0.27983100
H	2.03302800	2.37442800	-1.09500300
H	0.89272300	2.99875500	-2.30246200
O	0.41707600	3.29610800	-0.31596900
F	3.18443100	1.32658200	0.87599400

(*R*)-**TS2**P-F

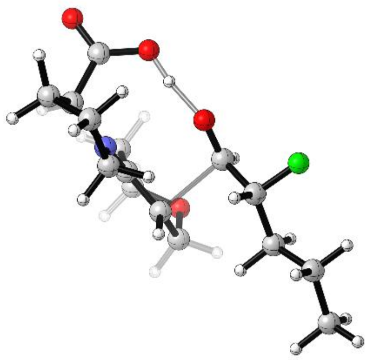

- Thermochemistry -

Zero-point correction=	0.387182 (Hartree/Particle)
Thermal correction to Energy=	0.404578
Thermal correction to Enthalpy=	0.405443
Thermal correction to Gibbs Free Energy=	0.343633
Sum of electronic and zero-point Energies=	-1040.752710
Sum of electronic and thermal Energies=	-1040.735314
Sum of electronic and thermal Enthalpies=	-1040.734449
Sum of electronic and thermal Free Energies=	-1040.796259

Number of Imaginary Frequencies = 1

E (Single Point Energy) [IEFPCM$_{(DCM)}$M06-2X/6-311++G(2d,2p)] = -1041.429464

C	0.55685300	0.96400700	-0.86463000
H	0.96146500	0.28563000	-1.61103800
C	-0.81418400	0.95421300	-0.63043800
N	-1.56393300	-0.12715200	-0.87509300
C	-2.99431400	-0.23610000	-0.56995700
H	-3.54469900	0.62799900	-0.94918400
C	-1.07155500	-1.29652600	-1.62358400
H	-0.09125900	-1.59421800	-1.25403500
H	-0.99528100	-1.03889200	-2.68813100
C	-2.14245700	-2.34878000	-1.36351000

H	-1.95351300	-2.82900200	-0.39776800
H	-2.16456100	-3.11734600	-2.13735500
C	-3.42569300	-1.51811400	-1.31134200
H	-3.74480300	-1.25125000	-2.32270500
H	-4.25750500	-2.01241300	-0.80793000
C	-1.44833200	2.09364800	0.12988700
H	-2.46308100	2.28307500	-0.23080900
H	-1.52614300	1.80890900	1.18804700
C	0.94534500	-0.29622800	0.92011500
H	1.08895800	0.63255400	1.49388900
C	2.21589500	-1.03852800	0.49047500
O	-0.04554000	-1.03953300	1.13937400
C	-3.34083900	-0.32549600	0.92669500
O	-2.40057500	-0.59135000	1.80150700
O	-4.50190000	-0.16751100	1.25581600
C	3.42540000	-0.24619300	0.04136800
H	3.20113600	0.22292700	-0.92260600
C	4.66783800	-1.12510200	-0.11904800
H	4.91907300	-1.57867900	0.84423800
H	4.43881300	-1.94930600	-0.80632200
H	1.94108100	-1.80473000	-0.24399500
H	3.62794600	0.55500300	0.76407400
C	5.85883500	-0.32733900	-0.64549600
H	5.63765200	0.10846200	-1.62538700
H	6.74383100	-0.96032300	-0.75063600
H	6.11052800	0.49176000	0.03605300
H	-1.43219400	-0.71378000	1.45801000
C	-0.61632100	3.36324100	0.00537900

C	1.32272900	2.25856500	-0.66790200
H	-0.69095700	3.77337800	-1.01260100
H	-0.96231700	4.11914700	0.71166000
H	2.34021900	2.06969500	-0.32331200
H	1.39368700	2.80764300	-1.62107400
O	0.73623100	3.09126700	0.31359600
F	2.58094800	-1.73786400	1.66046000

(*R*)-**TS2**$_{P}$-F-P

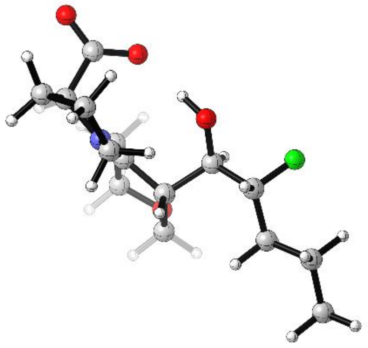

- Thermochemistry -

Zero-point correction= 0.391106 (Hartree/Particle)
Thermal correction to Energy= 0.408479
Thermal correction to Enthalpy= 0.409344
Thermal correction to Gibbs Free Energy= 0.347439
Sum of electronic and zero-point Energies= -1040.781306
Sum of electronic and thermal Energies= -1040.763933
Sum of electronic and thermal Enthalpies= -1040.763068
Sum of electronic and thermal Free Energies= -1040.824973

Number of Imaginary Frequencies = 0

E (Single Point Energy) [IEFPCM$_{(DCM)}$M06-2X/6-311++G(2d,2p)] = -1041.461821

C	0.60109000	0.57670600	-0.59108100
H	0.79864300	-0.09624500	-1.43168300
C	-0.88120300	0.79748500	-0.44720200

N	-1.72573000	-0.09226300	-0.84143400
C	-3.17412600	-0.05763900	-0.53312200
H	-3.59046800	0.91217000	-0.80850600
C	-1.36149100	-1.38165700	-1.50084500
H	-0.49132100	-1.80732800	-1.00507100
H	-1.13775300	-1.16413300	-2.54981200
C	-2.62045500	-2.23070700	-1.34952600
H	-2.60412100	-2.74771300	-0.38519500
H	-2.69460600	-2.97605500	-2.14215100
C	-3.74475700	-1.19533400	-1.37915400
H	-3.92509100	-0.85241500	-2.40275700
H	-4.68357400	-1.55044100	-0.95481900
C	-1.31118700	2.02457700	0.29886400
H	-2.37137700	2.23946000	0.16732000
H	-1.15883200	1.80224900	1.36327600
C	1.08720900	-0.09916100	0.74110300
H	1.27103000	0.69627300	1.47893600
C	2.37406900	-0.90701000	0.55031600
O	0.16535300	-1.04791600	1.22202700
C	-3.41614800	-0.27522800	1.00599300
O	-2.40576800	-0.36349600	1.75067300
O	-4.61593900	-0.32105400	1.31703600
C	3.57049500	-0.24411200	-0.10397100
H	3.31266900	0.00372400	-1.14079300
C	4.79767800	-1.15799600	-0.11395400
H	5.07692600	-1.39963400	0.91592400
H	4.53504400	-2.10550000	-0.60100100
H	2.10706500	-1.82638000	0.01157100

H	3.80962900	0.69206900	0.41701800
C	5.97814300	-0.51291300	-0.83646100
H	5.72712700	-0.29116000	-1.87901400
H	6.85125000	-1.17062800	-0.83314900
H	6.26381000	0.42721500	-0.35348500
H	-0.71235000	-0.67621300	1.47375000
C	-0.46071400	3.23848100	-0.08074300
C	1.28655600	1.92888200	-0.88534400
H	-0.68578000	3.56917900	-1.10466700
H	-0.66692100	4.06033300	0.60516600
H	2.36816600	1.83877900	-0.82090600
H	1.02986400	2.24308000	-1.90751900
O	0.91221900	2.92887800	0.03469800
F	2.76146700	-1.30804500	1.83698800

(*S*)-**TS2**$_{\mathrm{P}}$-F-Pre

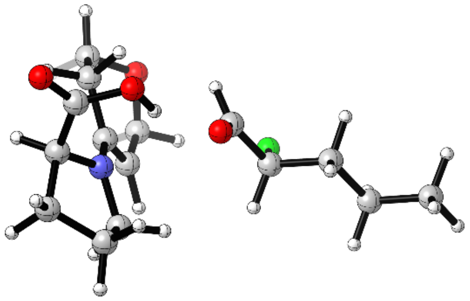

- Thermochemistry -

Zero-point correction=	0.386567 (Hartree/Particle)
Thermal correction to Energy=	0.405753
Thermal correction to Enthalpy=	0.406618
Thermal correction to Gibbs Free Energy=	0.338546
Sum of electronic and zero-point Energies=	-1040.767795
Sum of electronic and thermal Energies=	-1040.748609
Sum of electronic and thermal Enthalpies=	-1040.747744
Sum of electronic and thermal Free Energies=	-1040.815816

Number of Imaginary Frequencies = 0

E (Single Point Energy) [IEFPCM$_{(DCM)}$M06-2X/6-311++G(2d,2p)] = -1041.445581

C	-0.22542200	1.57588300	-1.27323400
H	0.16865300	1.06030300	-2.14366500
C	-1.20257800	1.03258300	-0.51706900
N	-1.65813000	-0.26974700	-0.68534700
C	-3.00617900	-0.68041500	-0.29863600
H	-3.74585000	0.11649400	-0.42302200
C	-1.14065700	-1.09328900	-1.77780300
H	-0.06260800	-1.23865800	-1.65620800
H	-1.31928400	-0.61175200	-2.75210600
C	-1.94302600	-2.38247200	-1.64562000
H	-1.51826500	-3.00772900	-0.85280700
H	-1.96108500	-2.96195600	-2.57008800
C	-3.32546100	-1.86166900	-1.24450500
H	-3.84796600	-1.47318100	-2.12304100
H	-3.96527700	-2.60686600	-0.76858000
C	-1.74537800	1.78590700	0.67566100
H	-2.83936400	1.73980200	0.70812900
H	-1.37940500	1.31527700	1.59912200
C	1.33661000	-0.01218900	0.75615400
H	1.10725100	0.95667200	1.23569800
C	2.59320100	-0.06051300	-0.08554100
H	2.35170500	-0.43506600	-1.08759400
O	0.66027900	-1.00606700	0.93357200
C	-3.10347100	-1.12996100	1.16206800
O	-1.96878800	-1.38608800	1.80435600

O	-4.17732900	-1.28341400	1.70309100
H	-1.17869400	-1.17491200	1.25893500
C	3.66924200	-0.91001400	0.57353200
H	3.24546900	-1.90878200	0.73117600
C	4.94247300	-0.99736300	-0.26627300
H	4.69594400	-1.42107200	-1.24759700
H	3.89345700	-0.49011400	1.56249300
C	6.01043500	-1.85032700	0.41489100
H	6.91610700	-1.90965300	-0.19420700
H	6.28477100	-1.42757300	1.38664900
H	5.64822400	-2.86966200	0.58272200
H	5.32937700	0.01155900	-0.44368900
C	0.36922900	2.92521700	-0.95665800
C	-1.31296000	3.24662500	0.62888800
H	1.45918900	2.88644600	-1.02822600
H	0.01148300	3.68822700	-1.66812300
H	-1.87477100	3.78478600	-0.14942400
H	-1.49216600	3.73567700	1.58796000
O	0.07519200	3.34459400	0.36584100
F	3.05209200	1.24997200	-0.23078700

(*S*)-**TS2**P-F

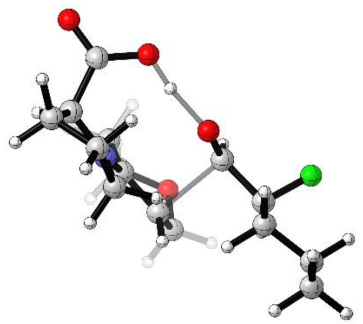

- Thermochemistry -

Zero-point correction=	0.386859 (Hartree/Particle)
Thermal correction to Energy=	0.404387
Thermal correction to Enthalpy=	0.405252
Thermal correction to Gibbs Free Energy=	0.342890
Sum of electronic and zero-point Energies=	-1040.757793
Sum of electronic and thermal Energies=	-1040.740265
Sum of electronic and thermal Enthalpies=	-1040.739400
Sum of electronic and thermal Free Energies=	-1040.801762

Number of Imaginary Frequencies = 1

E (Single Point Energy) [IEFPCM$_{(DCM)}$M06-2X/6-311++G(2d,2p)] = -1041.434367

C	0.29619200	1.25786300	-1.04989500
H	0.92793900	0.62419400	-1.66593900
C	-1.00172400	0.85316000	-0.77002400
N	-1.37244800	-0.43406900	-0.82779900
C	-2.69218700	-0.93631500	-0.43738400
H	-3.49268900	-0.34142200	-0.88267900
C	-0.53479500	-1.48874300	-1.42657500
H	0.48235900	-1.42202800	-1.04386600
H	-0.52525500	-1.36739000	-2.51776400
C	-1.23509400	-2.77313400	-0.99754100

H	-0.91021700	-3.04685900	0.01185400
H	-1.02016100	-3.60554100	-1.66911400
C	-2.71135200	-2.37284400	-0.99754700
H	-3.09692200	-2.34763000	-2.02063600
H	-3.35148200	-3.02754800	-0.40465100
C	-1.97226400	1.84378400	-0.17267000
H	-2.98533600	1.66264000	-0.54212400
H	-1.99477800	1.70450000	0.91712100
C	0.96782900	0.53108900	0.94244100
H	0.59796000	1.46745000	1.38779700
C	2.46859900	0.53084300	0.65216600
H	2.76679400	1.41236600	0.07868500
O	0.41241000	-0.56310200	1.21141900
C	-2.96281400	-0.93607500	1.07705600
O	-1.96303100	-0.80991600	1.91728400
O	-4.10992200	-1.08222300	1.45716000
H	-1.00525300	-0.68493500	1.53744900
C	3.00776900	-0.74178400	0.03772600
H	2.59040800	-0.83750000	-0.97273900
C	4.53413200	-0.75140100	-0.04962400
H	4.87120100	0.12703500	-0.61428100
H	2.64686700	-1.59668400	0.62027500
C	5.05498700	-2.02407900	-0.71381400
H	6.14687000	-2.02555300	-0.76743500
H	4.74415200	-2.91079000	-0.15191000
H	4.66703200	-2.12098100	-1.73324000
H	4.95348800	-0.65908300	0.95726100
C	0.62774100	2.73465700	-1.07210200

C	-1.56420700	3.27587400	-0.49966400
H	1.65486800	2.91540200	-0.74383700
H	0.53927400	3.13300300	-2.09578300
H	-1.74752900	3.49111400	-1.56274600
H	-2.13580900	3.98346000	0.10234400
O	-0.19734000	3.48294500	-0.20030000
F	3.04994900	0.69691800	1.92786200

(*S*)-**TS2**$_{P}$-F-P

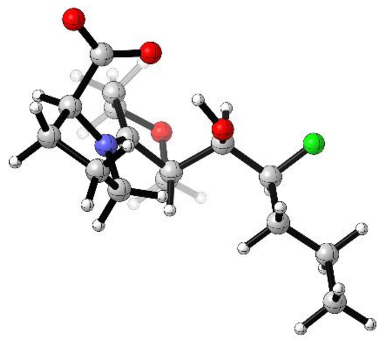

- Thermochemistry -

Zero-point correction=	0.391410 (Hartree/Particle)
Thermal correction to Energy=	0.408817
Thermal correction to Enthalpy=	0.409682
Thermal correction to Gibbs Free Energy=	0.347487
Sum of electronic and zero-point Energies=	-1040.785166
Sum of electronic and thermal Energies=	-1040.767760
Sum of electronic and thermal Enthalpies=	-1040.766895
Sum of electronic and thermal Free Energies=	-1040.829090

Number of Imaginary Frequencies = 0

E (Single Point Energy) [IEFPCM$_{(DCM)}$M06-2X/6-311++G(2d,2p)] = -1041.465939

C	0.50378500	0.89830600	-0.73559700
H	0.86430000	0.16376800	-1.45933800
C	-0.98511000	0.78669500	-0.57601300

N	-1.59811600	-0.32274800	-0.80992300
C	-2.99538600	-0.59238600	-0.40203000
H	-3.65252400	0.20122000	-0.75909600
C	-0.95703500	-1.56460200	-1.33856400
H	0.01528600	-1.70136700	-0.87004200
H	-0.84807400	-1.43984900	-2.42042100
C	-1.95805200	-2.66187500	-0.98875500
H	-1.76831500	-3.02583700	0.02571400
H	-1.88273500	-3.50276500	-1.67907300
C	-3.30488400	-1.94079200	-1.05286900
H	-3.61558900	-1.79694600	-2.09236500
H	-4.10339100	-2.45028100	-0.51376900
C	-1.68346400	1.97777900	0.00773100
H	-2.76300800	1.93454600	-0.13718900
H	-1.50170800	1.94572600	1.08976400
C	1.10168800	0.57424900	0.67886100
H	0.87724800	1.42300700	1.34080000
C	2.62269000	0.43839900	0.62544700
H	3.06637800	1.32495200	0.15982300
O	0.58757400	-0.62459300	1.19654600
C	-3.09907800	-0.65245300	1.16587700
O	-2.04732100	-0.45885700	1.83058200
O	-4.24502200	-0.88501500	1.57876400
H	-0.35331200	-0.52534200	1.47891600
C	3.13195900	-0.82898100	-0.03395300
H	2.76694300	-0.85977600	-1.06934400
C	4.65838200	-0.91532800	-0.03959700
H	5.06975500	-0.03734000	-0.55366400

H	2.69974100	-1.68936000	0.48790600
C	5.15390700	-2.19046300	-0.71807000
H	6.24582200	-2.24406400	-0.71434700
H	4.77142800	-3.07800500	-0.20374100
H	4.81765800	-2.23617000	-1.75931800
H	5.02423900	-0.87735300	0.99166000
C	0.87300700	2.30255700	-1.24786500
C	-1.12247800	3.28471800	-0.56434700
H	1.95223000	2.45477800	-1.21572600
H	0.54925300	2.40199200	-2.29400900
H	-1.42295600	3.40467700	-1.61494700
H	-1.50683700	4.12930100	0.00766800
O	0.28694100	3.31688700	-0.46256400
F	3.06533500	0.45082300	1.95727200

(*R*)-**TS2**$_G$-F-Pre

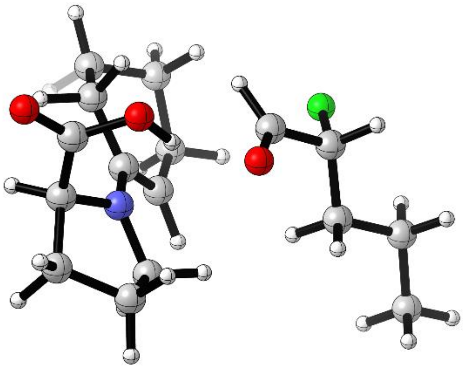

- Thermochemistry -

Zero-point correction= 0.410574 (Hartree/Particle)
Thermal correction to Energy= 0.429753
Thermal correction to Enthalpy= 0.430618
Thermal correction to Gibbs Free Energy= 0.364056
Sum of electronic and zero-point Energies= -1004.855476
Sum of electronic and thermal Energies= -1004.836297

Sum of electronic and thermal Enthalpies= -1004.835432
Sum of electronic and thermal Free Energies= -1004.901994

Number of Imaginary Frequencies = 0

E (Single Point Energy) [IEFPCM$_{(DCM)}$M06-2X/6-311++G(2d,2p)] = -1005.542966

C	-0.40906400	1.04771600	1.37340500
H	-0.84477700	0.21978700	1.92489500
C	0.78892000	0.88270300	0.77040500
N	1.41621600	-0.36326700	0.69425600
C	2.86232900	-0.50128200	0.55241900
H	3.42471100	0.26983500	1.08779300
C	0.83275000	-1.52527700	1.36205200
H	-0.16729400	-1.72186200	0.96318900
H	0.74633500	-1.35590100	2.44764100
C	1.83167700	-2.63419200	1.05418700
H	1.66426800	-3.01603300	0.04104500
H	1.76149500	-3.46960600	1.75297200
C	3.17087100	-1.89795200	1.14469700
H	3.45975400	-1.77453700	2.19203900
H	3.99082300	-2.39569200	0.62309100
C	1.47765600	2.01877900	0.04317000
H	2.54400900	2.03444700	0.29204300
H	1.42547700	1.83554300	-1.04112600
C	-1.00884300	-0.14119300	-1.55337600
H	-0.56926000	0.85349800	-1.74806500
C	-2.51235300	-0.16944900	-1.38848700
O	-0.33315600	-1.14966500	-1.53798800
C	3.32035600	-0.43459200	-0.90576300
O	2.41133900	-0.70385400	-1.83912000

O	4.47034800	-0.19356400	-1.20417400
C	-2.99205800	-1.01057700	-0.22206700
H	-2.52154200	-1.99712300	-0.31425000
C	-4.51303400	-1.14514100	-0.17603300
H	-4.96279500	-0.14733800	-0.12971500
H	-4.86186400	-1.60810300	-1.10717700
H	-2.94124300	-0.53075600	-2.33390000
H	-2.62046100	-0.55533500	0.70384200
C	-4.97101100	-1.97609600	1.02036700
H	-4.54677500	-2.98460200	0.98078400
H	-6.05983700	-2.06888300	1.04423600
H	-4.65146700	-1.51439100	1.96019000
H	1.51769700	-0.84748500	-1.45212200
C	0.86167700	3.37960400	0.37382600
C	-1.19026500	2.33843000	1.35611400
H	1.16435700	3.67674100	1.38613100
H	1.25640000	4.13252900	-0.31483800
H	-2.24795500	2.12002200	1.16469000
H	-1.15107900	2.80949300	2.34916900
F	-2.92987500	1.15420400	-1.23505700
C	-0.66193200	3.31535100	0.30640100
H	-1.09873800	4.30735200	0.45835600
H	-0.96918200	2.97911000	-0.69283400

(*R*)-**TS2**$_{G}$-F

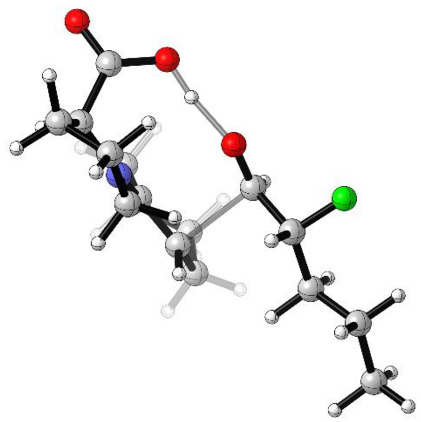

- Thermochemistry -

Zero-point correction=	0.410946 (Hartree/Particle)
Thermal correction to Energy=	0.428577
Thermal correction to Enthalpy=	0.429442
Thermal correction to Gibbs Free Energy=	0.367328
Sum of electronic and zero-point Energies=	-1004.840353
Sum of electronic and thermal Energies=	-1004.822722
Sum of electronic and thermal Enthalpies=	-1004.821857
Sum of electronic and thermal Free Energies=	-1004.883971

Number of Imaginary Frequencies = 1

E (Single Point Energy) [IEFPCM$_{(DCM)}$M06-2X/6-311++G(2d,2p)] = -1005.526459

C	0.55239900	0.99127700	-0.84644800
H	0.95716100	0.28228700	-1.56464700
C	-0.82046800	0.95678200	-0.62171300
N	-1.53965800	-0.15157800	-0.86836700
C	-2.97219800	-0.29617000	-0.59082000
H	-3.54196700	0.54212700	-0.99799100
C	-1.00900100	-1.31163300	-1.60394400
H	-0.02419500	-1.58158800	-1.22727100

H	-0.93097400	-1.06088800	-2.67043100
C	-2.04846300	-2.39411800	-1.34352900
H	-1.85405000	-2.85674700	-0.37025400
H	-2.03835800	-3.17189600	-2.10854500
C	-3.35676700	-1.60372700	-1.31401100
H	-3.67613300	-1.36341100	-2.33195700
H	-4.17722900	-2.11603600	-0.80957900
C	-1.51745800	2.08376100	0.10749000
H	-2.55181800	2.16804300	-0.23438600
H	-1.56318700	1.81780800	1.17439200
C	0.95059500	-0.29991300	0.94230700
H	1.10362100	0.62130800	1.52489400
C	2.22015700	-1.03955400	0.50605700
O	-0.04081400	-1.04043600	1.16147600
C	-3.34494800	-0.36583200	0.89948000
O	-2.41596400	-0.61382100	1.79290100
O	-4.51192500	-0.21399300	1.21048800
C	3.42372900	-0.24741600	0.04288700
H	3.18710800	0.22057700	-0.91834100
C	4.66558300	-1.12517200	-0.12722800
H	4.92600000	-1.57838900	0.83387700
H	4.43169800	-1.94989800	-0.81230900
H	1.94338700	-1.81206300	-0.22083600
H	3.63154800	0.55659800	0.76107400
C	5.85126400	-0.32659600	-0.66445900
H	5.62050800	0.10940200	-1.64204400
H	6.73587500	-0.95872700	-0.77801700
H	6.10861600	0.49260400	0.01492500

H	-1.44589500	-0.72448000	1.46066500
C	-0.80433600	3.42436000	-0.05516300
C	1.33116500	2.28434300	-0.68485700
H	-0.91452500	3.77537900	-1.08874800
H	-1.28315400	4.16445100	0.59199300
H	2.34834600	2.07371500	-0.34479300
H	1.43798500	2.76125200	-1.66934700
F	2.59596900	-1.73087500	1.67875100
C	0.67385200	3.27273300	0.27802100
H	1.18821200	4.23668700	0.22164300
H	0.77428900	2.91806600	1.31250100

(*R*)-**TS2**$_{G}$-F-P

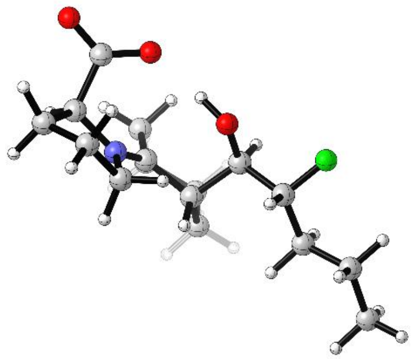

- Thermochemistry -

Zero-point correction= 0.415141 (Hartree/Particle)
Thermal correction to Energy= 0.432677
Thermal correction to Enthalpy= 0.433542
Thermal correction to Gibbs Free Energy= 0.371357
Sum of electronic and zero-point Energies= -1004.869691
Sum of electronic and thermal Energies= -1004.852154
Sum of electronic and thermal Enthalpies= -1004.851289
Sum of electronic and thermal Free Energies= -1004.913474

Number of Imaginary Frequencies = 0

E (Single Point Energy) [IEFPCM$_{(DCM)}$M06-2X/6-311++G(2d,2p)] = -1005.559850

C	0.60192900	0.58886300	-0.58080000
H	0.77552600	-0.10664900	-1.40748700
C	-0.88292500	0.80297500	-0.41811100
N	-1.72170800	-0.08617600	-0.83133400
C	-3.17177700	-0.07086500	-0.52538700
H	-3.59488500	0.90236600	-0.77648300
C	-1.34942500	-1.36002000	-1.51762700
H	-0.48288400	-1.79629600	-1.02518800
H	-1.11687600	-1.11926700	-2.55954100
C	-2.60533700	-2.21817400	-1.39623700
H	-2.59210400	-2.76083800	-0.44600200
H	-2.67155800	-2.94261500	-2.20882600
C	-3.73381300	-1.18784000	-1.40366700
H	-3.91049700	-0.81844000	-2.41872800
H	-4.67333200	-1.55817200	-0.99399000
C	-1.33165100	2.00571000	0.35999800
H	-2.41287600	2.12652300	0.32831700
H	-1.07855900	1.78732500	1.40783700
C	1.09350600	-0.10356700	0.74182000
H	1.28684100	0.67229000	1.49808800
C	2.37743600	-0.91539100	0.54288400
O	0.17316900	-1.05883000	1.21682600
C	-3.41597700	-0.33106500	1.00534400
O	-2.40683600	-0.42663800	1.75105200
O	-4.61617500	-0.39843500	1.31366700
C	3.57783100	-0.26147200	-0.11241000
H	3.32244800	-0.02336500	-1.15171000

C	4.80305600	-1.17804500	-0.10895600
H	5.08027600	-1.40930200	0.92383900
H	4.53971900	-2.13043600	-0.58611400
H	2.10320200	-1.83174300	0.00280300
H	3.81703400	0.68036600	0.39849900
C	5.98618200	-0.54323700	-0.83635300
H	5.73724300	-0.33164300	-1.88150900
H	6.85814000	-1.20244200	-0.82485200
H	6.27299600	0.40138800	-0.36276100
H	-0.70986800	-0.69606800	1.46324400
C	-0.61398100	3.28884000	-0.07518000
C	1.30419500	1.92526800	-0.91709400
H	-0.91947100	3.55749100	-1.09356300
H	-0.93324100	4.10045700	0.58339600
H	2.38426900	1.79059600	-0.88592800
H	1.05433900	2.17630500	-1.95517800
F	2.76569900	-1.32350200	1.82850000
C	0.89491200	3.09240400	-0.02110800
H	1.41076600	3.99705400	-0.35569500
H	1.20779500	2.91477800	1.01530300

(*S*)-**TS2**$_G$-F-Pre

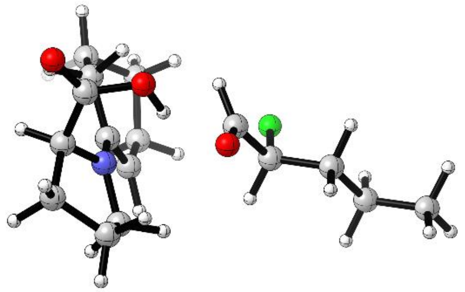

- Thermochemistry -

Zero-point correction= 0.410432 (Hartree/Particle)
Thermal correction to Energy= 0.429731
Thermal correction to Enthalpy= 0.430596
Thermal correction to Gibbs Free Energy= 0.363048
Sum of electronic and zero-point Energies= -1004.855125
Sum of electronic and thermal Energies= -1004.835826
Sum of electronic and thermal Enthalpies= -1004.834961
Sum of electronic and thermal Free Energies= -1004.902509

Number of Imaginary Frequencies = 0

E (Single Point Energy) [IEFPCM$_{(DCM)}$M06-2X/6-311++G(2d,2p)] = -1005.542429

C	-0.15411400	1.48435100	-1.35311400
H	0.19304400	0.87779500	-2.18502400
C	-1.15807500	1.02800500	-0.57146900
N	-1.67666200	-0.26218000	-0.70504000
C	-3.02474300	-0.61108900	-0.26708300
H	-3.74809500	0.19761100	-0.40815300
C	-1.22963700	-1.12828600	-1.79502400
H	-0.15251300	-1.30303800	-1.71766400
H	-1.43744400	-0.66789000	-2.77447500
C	-2.05582000	-2.39233100	-1.58980600
H	-1.60888400	-3.00512800	-0.79920900
H	-2.12937700	-2.99807200	-2.49469100

C	-3.40705100	-1.82812000	-1.14385600
H	-3.96683000	-1.46821500	-2.01143300
H	-4.03498500	-2.54217400	-0.60727300
C	-1.70579800	1.84741100	0.57877900
H	-2.79920900	1.79338900	0.59556900
H	-1.36752300	1.40088700	1.52653200
C	0.21761600	3.42651400	0.20840800
C	1.24062400	-0.30113600	0.79459300
H	0.89044700	0.51603300	1.45123300
C	2.54687500	-0.05368200	0.07270800
H	2.37549400	-0.09132100	-1.01021200
O	0.64051200	-1.35243200	0.70101900
C	-3.08538800	-0.98784600	1.21476300
O	-1.94855100	-1.36796100	1.79206900
O	-4.12913500	-0.98631000	1.83131700
H	-1.18144800	-1.28791700	1.18137400
C	3.62451900	-1.03871300	0.49548000
H	3.23579600	-2.04631700	0.30667800
C	4.93994900	-0.82479900	-0.25116500
H	4.76032400	-0.92158500	-1.32898100
H	3.78126400	-0.94722400	1.57775300
C	6.00852600	-1.82289800	0.18925400
H	6.94475300	-1.66368700	-0.35194000
H	6.21671400	-1.72351000	1.25934500
H	5.68220800	-2.85153300	0.00521300
H	5.29231500	0.19764800	-0.07879700
C	0.52162000	2.82019200	-1.16072400
C	-1.27459900	3.31341700	0.50857700

H	1.60431700	2.70126000	-1.28558900
H	0.19879200	3.51546500	-1.94942900
H	-1.83979200	3.81852300	-0.28522000
H	-1.52715100	3.81010700	1.45012700
H	0.78648300	2.89093800	0.98031200
H	0.54118100	4.47161900	0.24030700
F	2.94412400	1.24769600	0.38575000

(*S*)-**TS2**$_G$-F

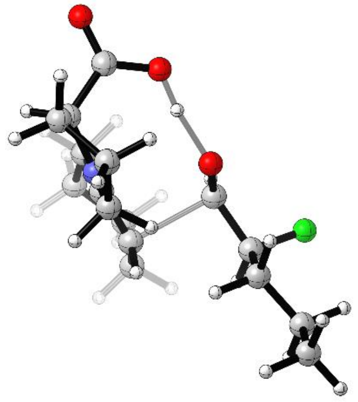

- Thermochemistry -

Zero-point correction=	0.410676 (Hartree/Particle)
Thermal correction to Energy=	0.428420
Thermal correction to Enthalpy=	0.429285
Thermal correction to Gibbs Free Energy=	0.366632
Sum of electronic and zero-point Energies=	-1004.845643
Sum of electronic and thermal Energies=	-1004.827898
Sum of electronic and thermal Enthalpies=	-1004.827033
Sum of electronic and thermal Free Energies=	-1004.889686

Number of Imaginary Frequencies = 1

E (Single Point Energy) [IEFPCM$_{(DCM)}$M06-2X/6-311++G(2d,2p)] = -1005.531600

C	0.28422100	1.27242500	-1.02612100
H	0.91427500	0.61335000	-1.61735400

C	-1.00974000	0.85275400	-0.74805300
N	-1.35835600	-0.44603700	-0.82479700
C	-2.67109600	-0.97698600	-0.44992700
H	-3.48147200	-0.40319900	-0.90452400
C	-0.50633100	-1.47812800	-1.44191400
H	0.51347500	-1.40013900	-1.07048800
H	-0.50958300	-1.34742600	-2.53257800
C	-1.17364700	-2.78071700	-1.01731100
H	-0.83638200	-3.05092200	-0.01098000
H	-0.94285200	-3.60470900	-1.69421800
C	-2.65812100	-2.41517700	-1.00777000
H	-3.05057100	-2.40045600	-2.02845800
H	-3.27918900	-3.08522600	-0.41136600
C	-2.01922900	1.81152800	-0.15586600
H	-3.02725400	1.53936000	-0.47759600
H	-2.00155500	1.69291800	0.93850700
C	-0.28161300	3.62279100	-0.24185100
C	0.96165300	0.47960900	0.97319000
H	0.57695800	1.38833700	1.45976200
C	2.45993200	0.52136700	0.68025000
H	2.73398700	1.42314600	0.12733600
O	0.42115700	-0.63009300	1.19834300
C	-2.95702200	-0.97954900	1.06050500
O	-1.95969200	-0.87431500	1.90807900
O	-4.10781700	-1.11229800	1.43488500
H	-1.00421000	-0.75456200	1.52727500
C	3.02702400	-0.72405600	0.03603200
H	2.60270000	-0.81188600	-0.97206200

C	4.55218700	-0.69039000	-0.06491800
H	4.85931000	0.20438300	-0.62092700
H	2.69471100	-1.59962400	0.60483200
C	5.10198300	-1.93926800	-0.75061200
H	6.19288400	-1.90926300	-0.81505500
H	4.82192200	-2.84146200	-0.19720900
H	4.70645100	-2.03419700	-1.76730100
H	4.97843600	-0.59955200	0.93925400
C	0.64183200	2.74389400	-1.08505100
C	-1.73694400	3.26872500	-0.52298100
H	1.68186400	2.89453300	-0.77507500
H	0.59760000	3.07784500	-2.13115400
H	-1.95078900	3.42191100	-1.58811800
H	-2.41598200	3.91562600	0.03937800
H	-0.07313200	3.48061900	0.82630400
H	-0.09038100	4.67699200	-0.46320100
F	3.04194900	0.67073200	1.95916900

(*S*)-**TS2**$_{G}$-F-P

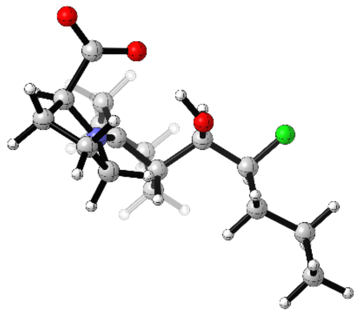

- Thermochemistry -

Zero-point correction= 0.415255 (Hartree/Particle)
Thermal correction to Energy= 0.432945

Thermal correction to Enthalpy= 0.433810
Thermal correction to Gibbs Free Energy= 0.370935
Sum of electronic and zero-point Energies= -1004.874756
Sum of electronic and thermal Energies= -1004.857066
Sum of electronic and thermal Enthalpies= -1004.856201
Sum of electronic and thermal Free Energies= -1004.919076

Number of Imaginary Frequencies = 0

E (Single Point Energy) [IEFPCM$_{(DCM)}$M06-2X/6-311++G(2d,2p)] = -1005.564970

C	0.53459300	0.79357500	-0.70059700
H	0.82562400	-0.00447800	-1.38553400
C	-0.96108900	0.80227000	-0.53835800
N	-1.65908600	-0.24830600	-0.81256600
C	-3.06582500	-0.43561600	-0.39218400
H	-3.66676300	0.42828300	-0.67541200
C	-1.12245000	-1.51461800	-1.40189100
H	-0.17194700	-1.76217400	-0.93475300
H	-0.98846600	-1.34335400	-2.47434700
C	-2.21607300	-2.54144200	-1.11955300
H	-2.05900300	-2.98817300	-0.13293800
H	-2.21399900	-3.33812100	-1.86445900
C	-3.49517800	-1.70490200	-1.12580900
H	-3.79415100	-1.46534700	-2.15116000
H	-4.33432200	-2.17651600	-0.61461900
C	-1.57274600	2.02061400	0.09124400
H	-2.65971300	2.00230900	0.02848800
H	-1.32565400	1.96546700	1.16032300
C	0.50962500	3.34250000	-0.49465300
C	1.08265600	0.41300700	0.71762900
H	0.81160000	1.20051900	1.43501000
C	2.60695000	0.31736100	0.73018600

H	3.04548500	1.28153500	0.45001800
O	0.56632100	-0.82619000	1.13261600
C	-3.13518500	-0.59946800	1.16977000
O	-2.05571600	-0.52169900	1.81390900
O	-4.28292000	-0.78689600	1.60196800
H	-0.36884400	-0.72500300	1.43034300
C	3.18529300	-0.80441100	-0.11015800
H	2.93049200	-0.61487800	-1.16148600
C	4.70404100	-0.91871500	0.02100800
H	5.16367900	0.04024300	-0.25037600
H	2.70261700	-1.74521700	0.17841900
C	5.26873200	-2.03083800	-0.86006300
H	6.35434100	-2.10766300	-0.75646800
H	4.83545200	-2.99899500	-0.58868500
H	5.04277000	-1.84732800	-1.91578200
H	4.96183700	-1.10685000	1.06804700
C	1.04550900	2.13436900	-1.26110300
C	-1.01672400	3.32344000	-0.50554800
H	2.13848000	2.12686600	-1.28396300
H	0.71974200	2.20641200	-2.30592700
H	-1.37562500	3.43146900	-1.53620300
H	-1.42455000	4.16084300	0.06608800
H	0.87739100	3.34240200	0.53887000
H	0.87581000	4.26205300	-0.95998500
F	2.97522800	0.11215200	2.06855400

(*R*)-**TS2**$_{O}$-F-Pre

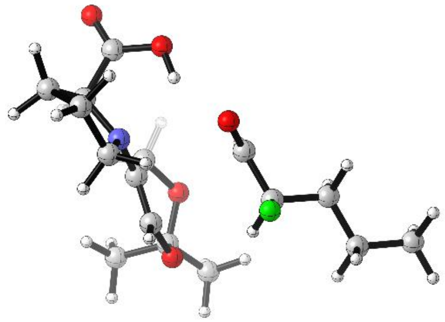

- Thermochemistry -

Zero-point correction=	0.418212 (Hartree/Particle)
Thermal correction to Energy=	0.439452
Thermal correction to Enthalpy=	0.440317
Thermal correction to Gibbs Free Energy=	0.368522
Sum of electronic and zero-point Energies=	-1155.234053
Sum of electronic and thermal Energies=	-1155.212813
Sum of electronic and thermal Enthalpies=	-1155.211948
Sum of electronic and thermal Free Energies=	-1155.283743

Number of Imaginary Frequencies = 0

E (Single Point Energy) [IEFPCM$_{(DCM)}$M06-2X/6-311++G(2d,2p)] = -1155.977111

C	0.00221100	1.04271500	1.30629100
H	-0.08023000	0.53402500	2.25870400
C	0.96054200	0.79872200	0.39391400
N	1.93688400	-0.18493000	0.55474000
C	3.23481000	-0.06282800	-0.10705000
H	3.50327200	0.97366900	-0.33689800
C	2.05596400	-0.86240700	1.84700800
H	1.18084500	-1.49796100	2.01750500
H	2.12065600	-0.12675400	2.66527900
C	3.35908000	-1.64282200	1.70916600
H	3.18667300	-2.57085800	1.15345700
H	3.79557600	-1.89775800	2.67612100

C	4.23158400	-0.67846300	0.89992600
H	4.61031700	0.11715600	1.54759700
H	5.08032300	-1.15041600	0.40198300
C	0.89899900	1.51557300	-0.93251300
H	1.66212000	2.30245500	-1.01386200
H	1.05931400	0.81248200	-1.75746200
O	-1.03763300	1.91903700	1.09715000
O	-0.39887600	2.05227100	-1.12990900
C	-0.88797900	2.79102900	-0.02493100
C	-2.28102800	3.24656500	-0.40000300
H	-2.87735300	2.38140600	-0.70085800
H	-2.22853400	3.95529500	-1.22902200
C	0.03322200	3.94731500	0.34876600
H	-0.46204500	4.57204900	1.09481400
H	0.24429500	4.55064300	-0.53791900
H	0.97425000	3.59087400	0.77416200
C	-0.97613700	-1.25112400	-0.52540300
H	-0.87769700	-0.54864800	-1.37466200
C	-2.25355600	-1.09814600	0.26005500
O	-0.13402700	-2.09384200	-0.29463500
C	3.28356600	-0.82752800	-1.43519600
O	2.28679100	-1.66984300	-1.69058400
O	4.20475300	-0.69125900	-2.21055200
H	-2.75360000	3.73001200	0.45725800
C	-3.46045600	-1.49570500	-0.58062800
H	-3.43826300	-0.91247100	-1.51015600
C	-4.78091100	-1.25473700	0.15005500
H	-4.77783900	-1.81207600	1.09265600

H	-4.85652300	-0.19134000	0.40897400
H	-3.36208800	-2.55314600	-0.85636800
C	-5.98143100	-1.67140500	-0.69639500
H	-6.01224000	-1.10914000	-1.63530800
H	-5.93190800	-2.73639800	-0.94468100
H	-6.91999300	-1.49301000	-0.16529000
H	1.60799100	-1.66076600	-0.97740600
H	-2.33557500	-0.05093700	0.58360600
F	-2.18543100	-1.89170100	1.39716200

(*R*)-**TS2**$_{O}$-F

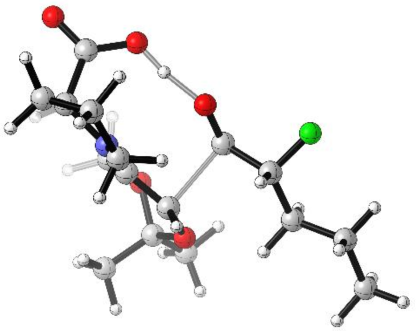

- Thermochemistry -

Zero-point correction=	0.417192 (Hartree/Particle)
Thermal correction to Energy=	0.436878
Thermal correction to Enthalpy=	0.437743
Thermal correction to Gibbs Free Energy=	0.371067
Sum of electronic and zero-point Energies=	-1155.218820
Sum of electronic and thermal Energies=	-1155.199133
Sum of electronic and thermal Enthalpies=	-1155.198268
Sum of electronic and thermal Free Energies=	-1155.264945

Number of Imaginary Frequencies = 1

E (Single Point Energy) [IEFPCM$_{(DCM)}$M06-2X/6-311++G(2d,2p)] = -1155.958868

C	-0.53501600	0.50533100	0.77410600
H	-0.77827000	-0.10096400	1.64194000
C	0.78928600	0.74849300	0.42600600
N	1.77377500	-0.07480600	0.78241900
C	3.16333700	0.06784600	0.33747800
H	3.47792300	1.11490300	0.37159900
C	1.61958300	-1.11717700	1.81542700
H	0.75974000	-1.74807100	1.58927300
H	1.47547000	-0.63224300	2.78981500
C	2.94269600	-1.87297700	1.73816600
H	2.88764500	-2.63153400	0.95036100
H	3.18809800	-2.36747200	2.67876500
C	3.94332000	-0.77819400	1.35941000
H	4.17733200	-0.15970600	2.23053300
H	4.87613000	-1.15294500	0.93734200
C	1.08929600	1.85577300	-0.55447600
H	1.74593100	2.60122000	-0.08229900
H	1.60390700	1.45823800	-1.43533100
O	-1.51918100	1.43351400	0.48422500
O	-0.09992600	2.44286200	-1.03281500
C	-1.07019900	2.68647100	-0.03032900
C	-2.25855200	3.31017200	-0.72724800
H	-2.56834300	2.66893000	-1.55531900
H	-1.98678600	4.29528500	-1.11185700
C	-0.52702000	3.55185600	1.10267100
H	-1.35206500	3.83810100	1.75784200
H	-0.07089300	4.45517400	0.68945000
H	0.21531600	3.02322700	1.70599300

C	-0.69447700	-0.99404200	-0.73662100
H	-0.90270000	-0.21367400	-1.48637800
C	-1.92000000	-1.74149000	-0.20178300
O	0.36085400	-1.69190800	-0.81290300
C	3.44092900	-0.40749800	-1.10425600
O	2.49974600	-1.00486900	-1.78515000
O	4.55021000	-0.19533400	-1.56159800
H	-3.08504500	3.41463500	-0.02163900
C	-3.21249400	-0.96886300	-0.04175800
H	-3.09155000	-0.23139400	0.75660500
C	-4.39769200	-1.88050600	0.28065900
H	-4.53802400	-2.60125300	-0.53063300
H	-4.16975000	-2.46217700	1.18311800
H	-3.40842700	-0.40345100	-0.96249100
C	-5.68192800	-1.08144800	0.49235900
H	-5.57218300	-0.37489500	1.32166700
H	-5.93418100	-0.50659800	-0.40480800
H	-6.52607600	-1.73843400	0.71845400
H	1.57453500	-1.23086200	-1.31472100
H	-1.63965300	-2.27043000	0.71750200
F	-2.13939300	-2.74406300	-1.16940600

(*R*)-**TS2**$_{O}$-F-P

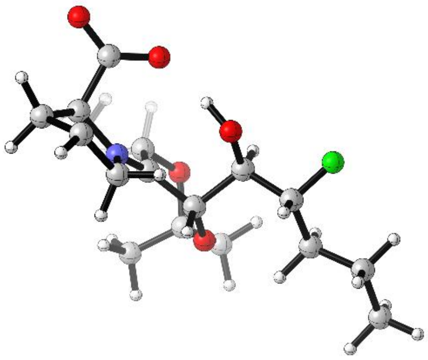

- Thermochemistry -

Zero-point correction=	0.421340 (Hartree/Particle)
Thermal correction to Energy=	0.441228
Thermal correction to Enthalpy=	0.442093
Thermal correction to Gibbs Free Energy=	0.374432
Sum of electronic and zero-point Energies=	-1155.249207
Sum of electronic and thermal Energies=	-1155.229319
Sum of electronic and thermal Enthalpies=	-1155.228454
Sum of electronic and thermal Free Energies=	-1155.296115

Number of Imaginary Frequencies = 0

E (Single Point Energy) [IEFPCM$_{(DCM)}$M06-2X/6-311++G(2d,2p)] = -1155.987905

C	-0.30733000	0.68729300	0.73125200
H	-0.35126700	0.31882900	1.75863800
C	1.09009900	0.64137000	0.19238700
N	1.94855600	-0.21250100	0.61963900
C	3.19791300	-0.52479100	-0.10052300
H	3.68180200	0.39735800	-0.43089700
C	1.77370000	-1.11996900	1.79636400
H	0.79255200	-1.58883100	1.75325800
H	1.86236600	-0.49918300	2.69275200
C	2.93389800	-2.10519300	1.66160800
H	2.62640400	-2.96082200	1.05293700

H	3.25454000	-2.47275200	2.63678600
C	4.01203100	-1.29794400	0.93390700
H	4.50862400	-0.60561900	1.62064300
H	4.76422800	-1.91468300	0.44308300
C	1.38722300	1.52920100	-0.98522400
H	2.30344400	2.10691900	-0.80087900
H	1.53947400	0.90485200	-1.87174400
O	-0.89810800	1.96946500	0.70804600
O	0.28475300	2.36496300	-1.24781600
C	-0.27086400	2.97404200	-0.09512000
C	-1.37446700	3.88593700	-0.58355200
H	-1.92683400	4.28249200	0.27036000
H	-0.94762000	4.71432600	-1.15272800
C	0.77469400	3.70955200	0.73830100
H	0.26416300	4.27258100	1.52171800
H	1.33724400	4.40368500	0.10860200
H	1.47497000	3.02657200	1.22805000
C	-1.13370800	-0.28499000	-0.18558500
H	-1.15708000	0.15683800	-1.19362000
C	-2.56701100	-0.34055700	0.32596000
O	-0.58892200	-1.57254600	-0.18279700
C	2.87393300	-1.39414800	-1.37797600
O	1.66087100	-1.55773800	-1.67552800
O	3.88129900	-1.80836300	-1.96517900
H	-2.05457900	3.31735800	-1.22156600
C	-3.44458100	-1.30111200	-0.44922100
H	-3.40125600	-1.01708600	-1.50886900
C	-4.89526300	-1.29464700	0.03119200

H	-4.92321000	-1.54752900	1.09669000
H	-5.30446800	-0.28096800	-0.06399100
H	-3.01540300	-2.30550100	-0.36777800
C	-5.76028400	-2.27682600	-0.75589000
H	-5.76183400	-2.02840900	-1.82234100
H	-5.38099200	-3.29854200	-0.65177300
H	-6.79583100	-2.26466400	-0.40538000
H	0.21117300	-1.60662400	-0.76154000
H	-2.98579200	0.67123500	0.32902600
F	-2.52374900	-0.75376700	1.67133500

(*S*)-**TS2**$_{O}$-F-Pre

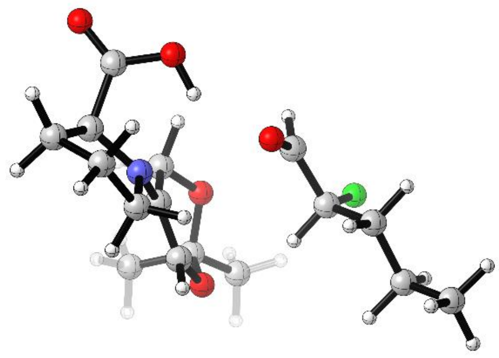

- Thermochemistry -

Zero-point correction=	0.418091 (Hartree/Particle)
Thermal correction to Energy=	0.439372
Thermal correction to Enthalpy=	0.440237
Thermal correction to Gibbs Free Energy=	0.368391
Sum of electronic and zero-point Energies=	-1155.232740
Sum of electronic and thermal Energies=	-1155.211459
Sum of electronic and thermal Enthalpies=	-1155.210594
Sum of electronic and thermal Free Energies=	-1155.282440

Number of Imaginary Frequencies = 0

E (Single Point Energy) [IEFPCM$_{(DCM)}$M06-2X/6-311++G(2d,2p)] = -1155.975450

C	0.06648800	0.85315900	-1.46064900
H	0.18301600	0.27708700	-2.36962200
C	-0.91010200	0.66406000	-0.55888400
N	-1.87778100	-0.34451800	-0.66201000
C	-3.23188200	-0.10270800	-0.15497300
H	-3.47673500	0.96258400	-0.08343400
C	-1.91699200	-1.15381000	-1.88198000
H	-1.04518900	-1.81465600	-1.91906900
H	-1.90716600	-0.51107500	-2.77734800
C	-3.24733100	-1.88981800	-1.76870000
H	-3.15278300	-2.74270400	-1.08808700
H	-3.60428500	-2.25590600	-2.73267800
C	-4.15590700	-0.81304700	-1.16831700
H	-4.44700300	-0.09632100	-1.94126700
H	-5.06161800	-1.19851700	-0.69721000
C	-0.88392900	1.46522000	0.71886100
H	-1.64136700	2.26177800	0.73025100
H	-1.07430900	0.81688700	1.58069900
O	1.09771800	1.74936600	-1.28510900
O	0.41343800	2.00487400	0.91772000
C	0.92323500	2.68387800	-0.21742200
C	2.30513400	3.16991800	0.16140500
H	2.79937300	3.59092400	-0.71642500
H	2.22603500	3.93943900	0.93231400
C	0.00582100	3.81200100	-0.67685700
H	0.52003400	4.39973200	-1.44004800
H	-0.23370700	4.46020300	0.17011400
H	-0.92025100	3.42740000	-1.11030800

C	1.10736600	-0.95019700	1.41142500
H	0.92988600	-0.43670300	2.37483100
C	2.42426600	-0.63896300	0.73681100
O	0.28959100	-1.70184600	0.92615600
C	-3.43293200	-0.69324600	1.24569500
O	-2.45646100	-1.46062700	1.72229400
O	-4.44407400	-0.49283200	1.88218600
H	2.89554200	2.33513800	0.54695100
C	3.14190700	-1.86887200	0.21523100
H	3.36517000	-2.53419200	1.05872500
C	4.42084800	-1.52251000	-0.54481200
H	4.17091500	-0.85944800	-1.38247100
H	5.09269800	-0.96102200	0.11328300
H	2.43950100	-2.39878000	-0.43904200
C	5.12900900	-2.77185000	-1.06391900
H	5.40619300	-3.43394000	-0.23730400
H	4.47978900	-3.33582000	-1.74148800
H	6.04113200	-2.51292900	-1.60790700
H	-1.70070800	-1.48753400	1.09410400
H	2.22468500	0.07918300	-0.07085100
F	3.21821500	0.02111600	1.68043100

(S)-**TS2**$_{O}$-F

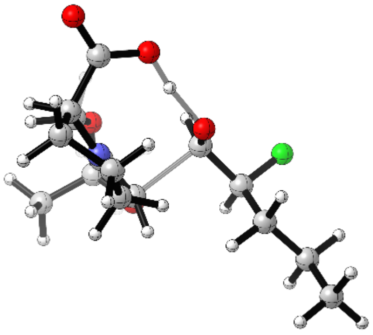

- Thermochemistry -

Zero-point correction=	0.417489 (Hartree/Particle)
Thermal correction to Energy=	0.437086
Thermal correction to Enthalpy=	0.437951
Thermal correction to Gibbs Free Energy=	0.371738
Sum of electronic and zero-point Energies=	-1155.222914
Sum of electronic and thermal Energies=	-1155.203318
Sum of electronic and thermal Enthalpies=	-1155.202453
Sum of electronic and thermal Free Energies=	-1155.268666

Number of Imaginary Frequencies = 1

E (Single Point Energy) [IEFPCM$_{(DCM)}$M06-2X/6-311++G(2d,2p)] = -1155.963577

C	0.44378400	0.85084500	-0.77208200
H	0.96887400	0.24542400	-1.50340000
C	-0.90285700	0.64028600	-0.50408100
N	-1.49683500	-0.52654900	-0.75546200
C	-2.86687800	-0.85039000	-0.35052300
H	-3.53598800	-0.00117700	-0.51574000
C	-0.90413200	-1.56734000	-1.61863700
H	0.09817200	-1.82011400	-1.27165900
H	-0.85437800	-1.18830900	-2.64774800
C	-1.88373300	-2.72989900	-1.47784800
H	-1.62372200	-3.32684600	-0.59747500

H	-1.87333100	-3.38196600	-2.35204800
C	-3.22890100	-2.02902900	-1.27047200
H	-3.60440000	-1.63759100	-2.22022700
H	-3.99721600	-2.66186500	-0.82549400
C	-1.65504100	1.69210700	0.27282400
H	-2.48235100	2.08302700	-0.33746700
H	-2.07945200	1.26519400	1.18763000
O	1.02321400	2.09339100	-0.60075500
O	-0.78732900	2.72214900	0.69225800
C	0.12228700	3.16218300	-0.30017400
C	0.95183900	4.25549300	0.33406200
H	1.74874100	4.55540100	-0.34913500
H	0.31987500	5.11913000	0.55066700
C	-0.58465600	3.61732500	-1.57266200
H	0.14461500	4.08572200	-2.23639700
H	-1.35816100	4.34761000	-1.32173900
H	-1.04391100	2.78395700	-2.11019400
C	0.94706400	-0.18601100	1.00197600
H	0.63126900	0.66717700	1.62458100
C	2.45082700	-0.20038500	0.73416500
O	0.34173900	-1.29640700	1.09411700
C	-3.02991900	-1.23626700	1.13466900
O	-1.97308200	-1.38644900	1.88711400
O	-4.16370000	-1.38652600	1.55589200
H	1.39050800	3.88403200	1.26252900
C	2.93994100	-1.40593800	-0.03769900
H	2.60986200	-2.31034400	0.48513800
C	4.45716200	-1.41077400	-0.22205200

H	4.76219600	-0.49596600	-0.74545400
H	4.93863700	-1.38721100	0.76122200
H	2.45265700	-1.41254300	-1.02153000
C	4.93173100	-2.63538700	-1.00126500
H	4.65521200	-3.55808300	-0.48085900
H	4.47877200	-2.66491100	-1.99786100
H	6.01793600	-2.63144200	-1.12511900
H	-0.99613800	-1.30785700	1.46738500
H	2.77290300	0.74392800	0.28602800
F	3.03098000	-0.23096800	2.02137700

(*S*)-**TS2**$_O$-F-P

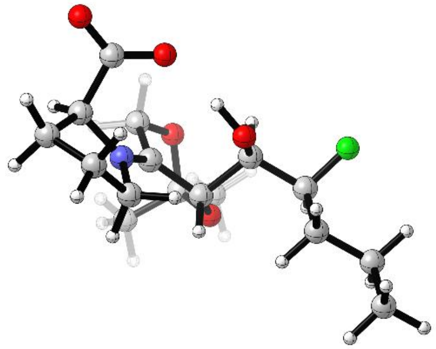

- Thermochemistry -

Zero-point correction=	0.421309 (Hartree/Particle)
Thermal correction to Energy=	0.441246
Thermal correction to Enthalpy=	0.442111
Thermal correction to Gibbs Free Energy=	0.373799
Sum of electronic and zero-point Energies=	-1155.246602
Sum of electronic and thermal Energies=	-1155.226664
Sum of electronic and thermal Enthalpies=	-1155.225799
Sum of electronic and thermal Free Energies=	-1155.294111

Number of Imaginary Frequencies = 0

E (Single Point Energy) [IEFPCM$_{(DCM)}$M06-2X/6-311++G(2d,2p)] = -1155.990832

C	0.52673700	0.52889100	-0.50086700
H	0.78759500	-0.07684300	-1.37282300
C	-0.96307700	0.59825400	-0.32965500
N	-1.73365100	-0.34143600	-0.74335800
C	-3.13474100	-0.48414100	-0.30219100
H	-3.63943300	0.48441800	-0.33088000
C	-1.32483800	-1.50394200	-1.59227200
H	-0.40786400	-1.93857900	-1.19898500
H	-1.16406700	-1.11837800	-2.60356100
C	-2.52953000	-2.44112300	-1.51737200
H	-2.41950600	-3.11983200	-0.66625500
H	-2.62229800	-3.03779700	-2.42522700
C	-3.71123200	-1.49484900	-1.29022200
H	-3.99568500	-0.99674100	-2.22210900
H	-4.58742400	-1.98377100	-0.86560100
C	-1.49166400	1.74331000	0.49061000
H	-2.31734200	2.23707600	-0.03989500
H	-1.86959200	1.35387500	1.44182300
O	1.15351700	1.78527800	-0.64643500
O	-0.45085600	2.64336500	0.79010300
C	0.38884200	2.94834500	-0.30911400
C	1.37675000	3.98402400	0.17931500
H	2.13246700	4.15997100	-0.58851800
H	0.85702500	4.92000100	0.39414000
C	-0.39460800	3.41270400	-1.53353000
H	0.31194300	3.76292600	-2.28812900
H	-1.06376000	4.23356600	-1.26302600
H	-0.98407200	2.60817500	-1.98303900

C	1.04333300	-0.17576600	0.80444000
H	0.80062900	0.48669400	1.64856200
C	2.56298400	-0.30814300	0.75440700
O	0.46997700	-1.44273000	0.95405200
C	-3.17252300	-1.01400300	1.18576600
O	-2.07716600	-1.08550100	1.80245900
O	-4.31178900	-1.28456900	1.58589100
H	1.86123400	3.61889800	1.08737600
C	3.07885200	-1.38441200	-0.18007600
H	2.64422500	-2.34423300	0.11981100
C	4.60535100	-1.46737300	-0.19434700
H	5.01798300	-0.49433300	-0.48913800
H	4.96411300	-1.66817200	0.82056500
H	2.72082200	-1.16935300	-1.19575600
C	5.10831400	-2.55159000	-1.14486200
H	4.72488200	-3.53420300	-0.85140600
H	4.77843200	-2.35654800	-2.17075500
H	6.20035200	-2.60358000	-1.14668600
H	-0.44207100	-1.35705700	1.32172500
H	3.00005000	0.66783300	0.52024200
F	2.97375000	-0.62353500	2.06025800

(*R*)-**TS2**$_T$-F-Pre

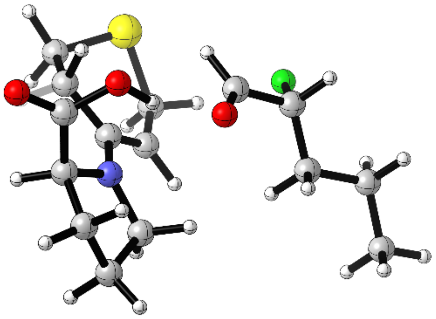

- Thermochemistry -

Zero-point correction=	0.383025 (Hartree/Particle)
Thermal correction to Energy=	0.402527
Thermal correction to Enthalpy=	0.403392
Thermal correction to Gibbs Free Energy=	0.335876
Sum of electronic and zero-point Energies=	-1363.738883
Sum of electronic and thermal Energies=	-1363.719381
Sum of electronic and thermal Enthalpies=	-1363.718516
Sum of electronic and thermal Free Energies=	-1363.786031

Number of Imaginary Frequencies = 0

E (Single Point Energy) [IEFPCM$_{(DCM)}$M06-2X/6-311++G(2d,2p)] = -1364.422147

C	-0.25200600	1.00649200	1.23364700
H	-0.93651700	0.26620600	1.63475100
C	0.95936600	0.60619500	0.77653100
N	1.30224400	-0.73878700	0.71542100
C	2.63668600	-1.22780000	0.39583200
H	3.40773400	-0.85535100	1.08323300
C	0.50933700	-1.74357400	1.43400300
H	-0.35627500	-2.04622200	0.83015500
H	0.13964900	-1.32852200	2.37809800
C	1.48663400	-2.89591800	1.67528000
H	0.99156800	-3.86846700	1.68721300

H	1.99466400	-2.75636000	2.63439500
C	2.48955600	-2.75593200	0.53223500
H	3.45127500	-3.23508000	0.72022600
H	2.06814000	-3.16742500	-0.39202000
C	1.99498800	1.56698900	0.22656600
H	2.98228800	1.29270500	0.61761200
H	2.04580800	1.46286900	-0.86497200
C	-1.10073800	0.04495200	-1.49278100
H	-0.61659900	1.02947800	-1.61358200
C	-2.59777700	0.06331900	-1.27076700
O	-0.48681800	-0.99999500	-1.60544900
C	3.11447600	-0.90941200	-1.01700100
O	2.19585500	-0.86449400	-1.97683300
O	4.29083300	-0.76947100	-1.27266300
C	-3.10155000	-0.97710700	-0.29128400
H	-2.69107700	-1.94498000	-0.60276000
C	-4.62658700	-1.03880700	-0.22500100
H	-5.01762900	-0.05217100	0.04620100
H	-5.02016300	-1.27327600	-1.22162600
H	-3.06950600	-0.06997200	-2.25525900
H	-2.68699700	-0.75258300	0.69917400
C	-5.10777400	-2.08178200	0.78111600
H	-4.74380100	-1.84966600	1.78720800
H	-6.19942700	-2.12069700	0.81916400
H	-4.74315300	-3.07901600	0.51432300
H	1.26918400	-0.95918400	-1.64694100
C	1.74223400	3.02816100	0.57309300
C	-0.76947800	2.41976000	1.28092400

H	1.88157800	3.20673900	1.64397000
H	2.43816500	3.66878100	0.02864200
H	-1.82735700	2.43105300	1.00644700
H	-0.68816600	2.84508100	2.28866900
S	0.06149600	3.54044000	0.11633300
F	-2.93735400	1.34655400	-0.83626500

(*R*)-**TS2**$_T$-F

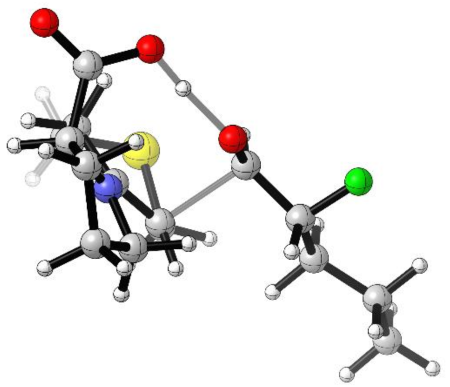

- Thermochemistry -

Zero-point correction=	0.383288 (Hartree/Particle)
Thermal correction to Energy=	0.401144
Thermal correction to Enthalpy=	0.402009
Thermal correction to Gibbs Free Energy=	0.339298
Sum of electronic and zero-point Energies=	-1363.724564
Sum of electronic and thermal Energies=	-1363.706709
Sum of electronic and thermal Enthalpies=	-1363.705844
Sum of electronic and thermal Free Energies=	-1363.768554

Number of Imaginary Frequencies = 1

E (Single Point Energy) [IEFPCM$_{(DCM)}$M06-2X/6-311++G(2d,2p)] = -1364.406058

C	0.50748500	0.70863200	-0.90677700
H	0.90627800	-0.07505100	-1.54728800
C	-0.87307200	0.70703700	-0.69778800
N	-1.57512300	-0.42155500	-0.86580600

C	-2.97063400	-0.62318000	-0.45102200
H	-3.64813400	-0.02088900	-1.06611900
C	-1.06218900	-1.57714700	-1.62860900
H	-0.34882900	-2.13401500	-1.01412800
H	-0.56095900	-1.22331300	-2.53327700
C	-2.31629900	-2.39432200	-1.92978500
H	-2.09237100	-3.45354800	-2.06375400
H	-2.79874700	-2.02227800	-2.83873400
C	-3.20030600	-2.12224000	-0.71491900
H	-4.25722600	-2.33755900	-0.87444200
H	-2.84786600	-2.70247900	0.14509100
C	-1.61405700	1.87911200	-0.09018800
H	-2.63845500	1.88783400	-0.47816400
H	-1.68155800	1.73018000	0.99464900
C	0.90645800	-0.40722500	0.95074200
H	1.06198500	0.55268800	1.46599600
C	2.16648100	-1.19195800	0.56945300
O	-0.08702300	-1.13177700	1.22958400
C	-3.29952700	-0.30760200	1.01176200
O	-2.37005700	-0.47489500	1.92170400
O	-4.43584200	0.02344100	1.29617200
C	3.37866600	-0.44806100	0.05053200
H	3.15907000	-0.08394800	-0.95886300
C	4.62330900	-1.33617300	-0.01302600
H	4.87323800	-1.68479100	0.99321800
H	4.39732100	-2.22890500	-0.60973500
H	1.87843800	-2.00859600	-0.10294800
H	3.57638800	0.42486600	0.68688900

C	5.81362900	-0.59541600	-0.61841700
H	6.06118700	0.29217900	-0.02704700
H	6.70057700	-1.23342000	-0.65334700
H	5.59411100	-0.26681700	-1.63961100
H	-1.41049000	-0.71952300	1.58983600
C	-0.97849000	3.23642900	-0.35982000
C	1.31966400	1.98658700	-0.93386000
H	-1.00762100	3.48490700	-1.42514000
H	-1.51947000	4.01176500	0.18503000
H	2.35540300	1.79487900	-0.65242400
H	1.34066700	2.40682100	-1.94637900
S	0.74173800	3.27240300	0.20842500
F	2.53353300	-1.80217100	1.78746700

(*R*)-**TS2**$_{T}$-F-P

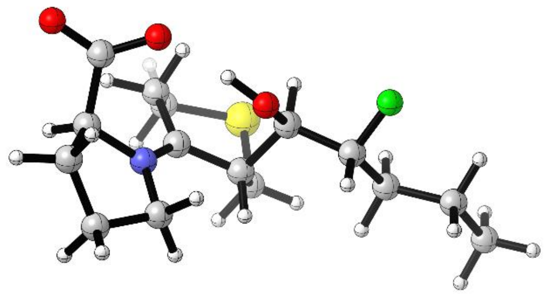

- Thermochemistry -

Zero-point correction= 0.387521 (Hartree/Particle)
Thermal correction to Energy= 0.405381
Thermal correction to Enthalpy= 0.406246
Thermal correction to Gibbs Free Energy= 0.343042
Sum of electronic and zero-point Energies= -1363.750405
Sum of electronic and thermal Energies= -1363.732545
Sum of electronic and thermal Enthalpies= -1363.731680
Sum of electronic and thermal Free Energies= -1363.794884

Number of Imaginary Frequencies = 0

E (Single Point Energy) [IEFPCM$_{(DCM)}$M06-2X/6-311++G(2d,2p)] = -1364.435765

C	0.52554800	0.28872800	-0.67187300
H	0.64263400	-0.48033500	-1.44022600
C	-0.94312100	0.57465900	-0.46314800
N	-1.83035000	-0.29518000	-0.81307400
C	-3.24806500	-0.26741300	-0.35381700
H	-3.74792000	0.58864500	-0.81378400
C	-1.56472200	-1.52766500	-1.60702900
H	-1.11173500	-2.26214800	-0.93409200
H	-0.88295500	-1.30184400	-2.42531300
C	-2.95439900	-1.94138200	-2.07210600
H	-2.98769600	-2.99946600	-2.33486600
H	-3.24547500	-1.35436000	-2.94844000
C	-3.82939900	-1.59319000	-0.87093500
H	-4.88659700	-1.48657700	-1.11260400
H	-3.72804000	-2.36194100	-0.09772600
C	-1.34444800	1.80908800	0.30001400
H	-2.42595500	1.94643400	0.26576800
H	-1.07982700	1.63525900	1.35049000
C	1.01835100	-0.34129700	0.68273400
H	1.18057500	0.46935100	1.40779800
C	2.31932700	-1.13998000	0.55170900
O	0.09393500	-1.28985200	1.15718500
C	-3.37802600	-0.18772700	1.19676700
O	-2.35805500	-0.45981600	1.88356400
O	-4.52068700	0.09749500	1.58816800
C	3.52535900	-0.49670700	-0.10527800

H	3.31829900	-0.38961900	-1.17681000
C	4.78872200	-1.34519300	0.05545100
H	5.02785400	-1.44122700	1.11839200
H	4.58959800	-2.35828800	-0.31620100
H	2.07673200	-2.08958700	0.05669200
H	3.69501800	0.50550100	0.31054800
C	5.97432600	-0.74057600	-0.69322100
H	6.19425500	0.26688600	-0.32507900
H	6.87405900	-1.34806400	-0.56532900
H	5.76660700	-0.66744400	-1.76584400
H	-0.75757400	-0.90077400	1.47468500
C	-0.66520600	3.09204900	-0.18745400
C	1.27316800	1.54260800	-1.16587200
H	-0.95173800	3.32584000	-1.21725800
H	-0.98324900	3.91968400	0.44816600
H	2.33385200	1.34069400	-1.28762800
H	0.88555500	1.81640500	-2.15253600
S	1.13928500	2.98576100	-0.07832400
F	2.67573200	-1.46861700	1.86876600

(*S*)-**TS2**$_{T}$-F-Pre

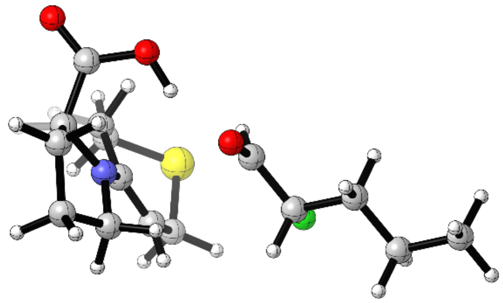

- Thermochemistry -

Zero-point correction=	0.383092 (Hartree/Particle)
Thermal correction to Energy=	0.402640
Thermal correction to Enthalpy=	0.403505
Thermal correction to Gibbs Free Energy=	0.335488
Sum of electronic and zero-point Energies=	-1363.738588
Sum of electronic and thermal Energies=	-1363.719040
Sum of electronic and thermal Enthalpies=	-1363.718175
Sum of electronic and thermal Free Energies=	-1363.786192

Number of Imaginary Frequencies = 0

E (Single Point Energy) [IEFPCM$_{(DCM)}$M06-2X/6-311++G(2d,2p)] = -1364.421894

C	-0.21774400	1.22389200	-1.34343900
H	0.26776300	0.58209500	-2.07251200
C	-1.27136900	0.74341100	-0.63841100
N	-1.68309900	-0.57676500	-0.76329400
C	-2.88614400	-1.12028800	-0.14735400
H	-3.80138600	-0.59836400	-0.45666800
C	-1.22858700	-1.39649900	-1.89333800
H	-0.24620400	-1.83445300	-1.67459100
H	-1.13557600	-0.77794300	-2.79270200
C	-2.31940200	-2.45758500	-2.05301900
H	-1.92960000	-3.40422400	-2.43118100
H	-3.08645600	-2.09942300	-2.74645200

C	-2.90605000	-2.57765700	-0.64822800
H	-3.91275300	-2.99661800	-0.61894300
H	-2.25298000	-3.19151300	-0.01745600
C	-2.02439100	1.56542500	0.38896800
H	-3.10051700	1.39174800	0.26936700
H	-1.75733300	1.22156700	1.39670600
C	1.30138100	-0.30882800	0.68882300
H	0.97463300	0.58529000	1.24849000
C	2.59786700	-0.18401700	-0.07873600
O	0.69833800	-1.36548700	0.73559200
C	-2.87841100	-1.12755300	1.37778300
O	-1.70856100	-1.30774200	1.98314100
O	-3.90405800	-1.04010400	2.01748800
C	3.69903700	-1.01732000	0.56002100
H	3.83882800	-0.68283800	1.59586100
C	5.01719400	-0.93105900	-0.20760900
H	4.85506100	-1.27265800	-1.23734400
H	5.33474700	0.11528500	-0.26591900
H	3.34425100	-2.05397900	0.59640300
C	6.11050700	-1.76886600	0.45186800
H	7.04849600	-1.70254200	-0.10541300
H	6.30072700	-1.42571300	1.47379600
H	5.81978700	-2.82315900	0.50051000
H	-0.93826000	-1.33706900	1.36455400
H	2.43903500	-0.47372200	-1.12412800
C	-1.77824500	3.06547600	0.29666800
C	0.35858200	2.61245800	-1.25475300
H	1.44814000	2.56392300	-1.32444300

H	0.00689800	3.24745500	-2.07708400
H	-2.21580300	3.47869900	-0.61761400
H	-2.23204900	3.57311400	1.14964300
S	-0.00492800	3.45383300	0.31453200
F	2.96222300	1.16353400	-0.06750700

(*S*)-**TS2**$_{T}$-F

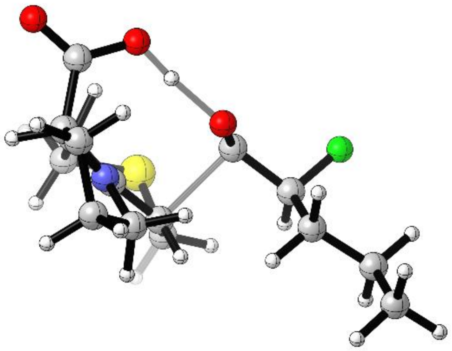

- Thermochemistry -

Zero-point correction=	0.383158 (Hartree/Particle)
Thermal correction to Energy=	0.401128
Thermal correction to Enthalpy=	0.401993
Thermal correction to Gibbs Free Energy=	0.338699
Sum of electronic and zero-point Energies=	-1363.730053
Sum of electronic and thermal Energies=	-1363.712083
Sum of electronic and thermal Enthalpies=	-1363.711218
Sum of electronic and thermal Free Energies=	-1363.774512

Number of Imaginary Frequencies = 1

E (Single Point Energy) [IEFPCM$_{(DCM)}$M06-2X/6-311++G(2d,2p)] = -1364.411596

C	0.24414800	1.03470300	-1.01614000
H	0.93100900	0.38412300	-1.55143200
C	-1.02865900	0.53400400	-0.75380500
N	-1.25443600	-0.78833300	-0.79273800
C	-2.46775900	-1.44955700	-0.29557200

H	-3.33638700	-1.18056700	-0.90626700
C	-0.35861000	-1.73969300	-1.48145100
H	0.50583500	-1.95696400	-0.84720300
H	-0.01609100	-1.30151700	-2.42280400
C	-1.23853300	-2.97028100	-1.68611900
H	-0.65098200	-3.88629600	-1.76296200
H	-1.83444900	-2.86027000	-2.59734200
C	-2.14480500	-2.94651000	-0.45700500
H	-3.05490200	-3.53789100	-0.56127300
H	-1.59485900	-3.29968900	0.42244000
C	-2.14999800	1.40271700	-0.22582600
H	-3.10782700	0.99033000	-0.56018600
H	-2.14652400	1.35587000	0.87101900
C	0.95316700	0.42161200	0.98173900
H	0.52061800	1.33293800	1.42070900
C	2.44892400	0.51718200	0.68647000
O	0.47930900	-0.70832100	1.27245300
C	-2.83722800	-1.15840600	1.16195600
O	-1.87445800	-0.91360400	2.01871900
O	-4.00533600	-1.22658800	1.49787400
C	3.06902500	-0.74053400	0.11944900
H	2.81410000	-1.58093000	0.77454900
C	4.58381600	-0.62445400	-0.04846300
H	4.81257500	0.22755500	-0.70108100
H	5.03876600	-0.40836800	0.92370800
H	2.60872700	-0.94367500	-0.85586300
C	5.18923400	-1.89934800	-0.63176200
H	6.27250300	-1.80808600	-0.74703800

H	4.99077600	-2.75682000	0.01936600
H	4.76277900	-2.12075600	-1.61581800
H	-0.89732200	-0.83728900	1.64877900
H	2.68410600	1.39400900	0.07760300
C	-2.07051300	2.86132000	-0.66029000
C	0.52945600	2.50579800	-1.21529900
H	1.56788500	2.73682600	-0.96504900
H	0.39459400	2.78414100	-2.26705000
H	-2.20421700	2.95873000	-1.74192600
H	-2.85703400	3.43597100	-0.16848400
S	-0.48628100	3.60949300	-0.19140400
F	3.02575000	0.77131700	1.94951000

(*S*)-**TS2**$_T$-F-P

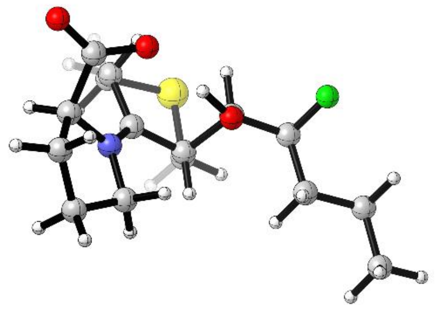

- Thermochemistry -

Zero-point correction= 0.415255 (Hartree/Particle)
Thermal correction to Energy= 0.432945
Thermal correction to Enthalpy= 0.433810
Thermal correction to Gibbs Free Energy= 0.370935
Sum of electronic and zero-point Energies= -1004.874756
Sum of electronic and thermal Energies= -1004.857066
Sum of electronic and thermal Enthalpies= -1004.856201
Sum of electronic and thermal Free Energies= -1004.919076

Number of Imaginary Frequencies = 0

E (Single Point Energy) [IEFPCM$_{(DCM)}$M06-2X/6-311++G(2d,2p)] = -1005.564970

C	0.53459300	0.79357500	-0.70059700
H	0.82562400	-0.00447800	-1.38553400
C	-0.96108900	0.80227000	-0.53835800
N	-1.65908600	-0.24830600	-0.81256600
C	-3.06582500	-0.43561600	-0.39218400
H	-3.66676300	0.42828300	-0.67541200
C	-1.12245000	-1.51461800	-1.40189100
H	-0.17194700	-1.76217400	-0.93475300
H	-0.98846600	-1.34335400	-2.47434700
C	-2.21607300	-2.54144200	-1.11955300
H	-2.05900300	-2.98817300	-0.13293800
H	-2.21399900	-3.33812100	-1.86445900
C	-3.49517800	-1.70490200	-1.12580900
H	-3.79415100	-1.46534700	-2.15116000
H	-4.33432200	-2.17651600	-0.61461900
C	-1.57274600	2.02061400	0.09124400
H	-2.65971300	2.00230900	0.02848800
H	-1.32565400	1.96546700	1.16032300
C	0.50962500	3.34250000	-0.49465300
C	1.08265600	0.41300700	0.71762900
H	0.81160000	1.20051900	1.43501000
C	2.60695000	0.31736100	0.73018600
H	3.04548500	1.28153500	0.45001800
O	0.56632100	-0.82619000	1.13261600
C	-3.13518500	-0.59946800	1.16977000
O	-2.05571600	-0.52169900	1.81390900

O	-4.28292000	-0.78689600	1.60196800
H	-0.36884400	-0.72500300	1.43034300
C	3.18529300	-0.80441100	-0.11015800
H	2.93049200	-0.61487800	-1.16148600
C	4.70404100	-0.91871500	0.02100800
H	5.16367900	0.04024300	-0.25037600
H	2.70261700	-1.74521700	0.17841900
C	5.26873200	-2.03083800	-0.86006300
H	6.35434100	-2.10766300	-0.75646800
H	4.83545200	-2.99899500	-0.58868500
H	5.04277000	-1.84732800	-1.91578200
H	4.96183700	-1.10685000	1.06804700
C	1.04550900	2.13436900	-1.26110300
C	-1.01672400	3.32344000	-0.50554800
H	2.13848000	2.12686600	-1.28396300
H	0.71974200	2.20641200	-2.30592700
H	-1.37562500	3.43146900	-1.53620300
H	-1.42455000	4.16084300	0.06608800
H	0.87739100	3.34240200	0.53887000
H	0.87581000	4.26205300	-0.95998500
F	2.97522800	0.11215200	2.06855400

Supplementary Table 9. Energies for enamine addition to 2-bromopentanal. Reported energies for structures optimized at the IEFPCM$_{(DCM)}$M06-2X/6-311++G(2d,2p)//IEFPCM$_{(DCM)}$M06-2X/6-31+G(d,p) level of theory represent the sum of the thermal correction to Gibbs Free Energy computed at the IEFPCM$_{(DCM)}$M06-2X/6-31+G(d,p) level of theory and single point energies computed at the IEFPCM$_{(DCM)}$M06-2X/6-311++G(2d,2p). All energies are reported in Hartrees.

**Structure**	**Single Point Energies, E IEFPCM$_{(DCM)}$M06-2X/6-311++G(2d,2p)**	**Thermal Corrections to Gibbs Free Energies, IEFPCM$_{(DCM)}$M06-2X/6-31+G(d,p)**	**Gibbs Free Energies (G), IEFPCM$_{(DCM)}$M06-2X/6-31+G(d,p)**	**Gibbs Free Energies (G), IEFPCM$_{(DCM)}$M06-2X/6-311++G(2d,2p)// IEFPCM$_{(DCM)}$M06-2X/6-31+G(d,p)**
2-Bromopentanal	-2845.302023	0.102041	-2842.756584	-2845.199982
Enamine of Cyclohexanone (G)	-634.55147535	0.238392	-634.142903	-634.3130834
Enamine of Dioxane (O)	-784.98483791	0.244077	-784.522619	-784.7407609
Enamine of Tetrahydro-4H-thiopyranone (T)	-993.43210840	0.210205	-993.028735	-993.2219034
Enamine of Tetrahydro-4H-pyranone (P)	-670.45469021	0.214793	-670.055312	-670.2398972
(*R*)-**TS2**$_P$-Br-Pre	-3515.773643	0.337623	-3512.813022	-3515.43602
(*R*)-**TS2**$_P$-Br	-3515.7581	0.340676	-3512.79657	-3515.417424
(*R*)-**TS2**$_P$-Br-P	-3515.787444	0.34455	-3512.822467	-3515.442891
(*S*)-**TS2**$_P$-Br-Pre	-3515.7753	0.337211	-3512.815113	-3515.438089
(*S*)-**TS2**$_P$-Br	-3515.764418	0.339866	-3512.804072	-3515.424552
(*S*)-**TS2**$_P$-Br P	-3515.792756	0.343886	-3512.829062	-3515.44887
(*R*)-**TS2**$_G$ Br-Pre	-3479.870764	0.360822	-3476.901401	-3479.509942
(*R*)-**TS2**$_G$-Br	-3479.855265	0.364163	-3476.884545	-3479.491102
(*R*)-**TS2**$_G$-Br-P	-3479.885376	0.368128	-3476.911154	-3479.517248
(*S*)-**TS2**$_G$-Br-Pre	-3479.871179	0.360308	-3476.904008	-3479.510871
(*S*)-**TS2**$_G$-Br	-3479.861757	0.363804	-3476.892016	-3479.497953
(*S*)-**TS2**$_G$-Br-P	-3479.891844	0.36803	-3476.918369	-3479.523814
(*R*)-**TS2**$_O$-Br-Pre	-3630.304563	0.366796	-3627.281363	-3629.937767

(*R*)-**TS2**O-Br	-3630.289223	0.369445	-3627.266059	-3629.919778
(*R*)-**TS2**O-Br-P	-3630.315195	0.372588	-3627.289573	-3629.942607
(*S*)-**TS2**O-Br-Pre	-3630.306273	0.365643	-3627.287761	-3629.94063
(*S*)-**TS2**O-Br	-3630.293793	0.368103	-3627.272308	-3629.92569
(*S*)-**TS2**O-Br-P	-3630.31805	0.370977	-3627.293836	-3629.947073
(*R*)-**TS2**T-Br-Pre	-3838.749773	0.332897	-3835.784701	-3838.416876
(*R*)-**TS2**T-Br	-3838.734498	0.335789	-3835.769038	-3838.398709
(*R*)-**TS2**T-Br-P	-3838.761015	0.339331	-3835.793023	-3838.421684
(*S*)-**TS2**T-Br-Pre	-3838.752143	0.332851	-3835.787543	-3838.419292
(*S*)-**TS2**T-Br	-3838.741723	0.335558	-3835.777067	-3838.406165
(*S*)-**TS2**T-Br-P	-3838.768722	0.339318	-3835.80117	-3838.429404

Pre – Precomplex

P – Product

(*R*)-**TS2**P-Br-Pre

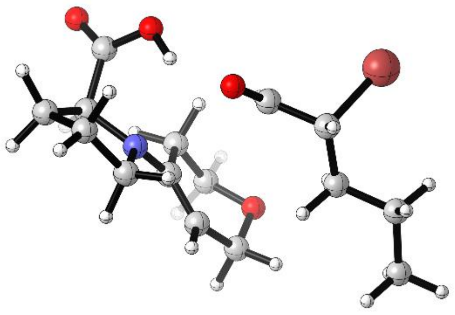

- Thermochemistry -

Zero-point correction= 0.385643 (Hartree/Particle)
Thermal correction to Energy= 0.404980
Thermal correction to Enthalpy= 0.405845
Thermal correction to Gibbs Free Energy= 0.337623
Sum of electronic and zero-point Energies= -3512.765002
Sum of electronic and thermal Energies= -3512.745665
Sum of electronic and thermal Enthalpies= -3512.744800

Sum of electronic and thermal Free Energies= -3512.813022

Number of Imaginary Frequencies = 0

E (Single Point Energy) [IEFPCM$_{(DCM)}$M06-2X/6-311++G(2d,2p)] = -3515.773643

C	0.60890900	2.19325600	0.57120500
H	0.58811100	2.24278500	1.65521600
C	1.44777000	1.36068500	-0.07714800
N	2.24220400	0.42071600	0.57744300
C	3.51685300	-0.02589200	0.01900000
H	4.04472200	0.75960900	-0.53026200
C	2.25204700	0.36617300	2.03796800
H	1.24901800	0.13131400	2.40771200
H	2.56488600	1.33130400	2.46795300
C	3.27484800	-0.72488000	2.32908900
H	2.81565600	-1.71077300	2.19697000
H	3.67657000	-0.66605000	3.34203800
C	4.33779900	-0.46701600	1.25681200
H	4.98089700	0.36366900	1.55961500
H	4.97485900	-1.32845400	1.04558300
C	1.49049600	1.34252600	-1.58886700
H	2.52353700	1.39468900	-1.95030800
H	1.07761900	0.39524800	-1.96204700
C	-0.68081200	-1.09972500	0.26286100
H	-0.40866700	-0.67701100	-0.72186300
C	-2.11316300	-0.90240000	0.67846900
O	0.13124100	-1.69011800	0.94411500
C	3.35391100	-1.19743500	-0.94851400
O	2.30230100	-1.99158500	-0.75854800
O	4.16446500	-1.43121100	-1.81819400

C	-2.50486400	0.56433200	0.66725300
H	-1.85296300	1.05442000	1.40503700
C	-3.96488400	0.82569100	1.02895700
H	-4.61170000	0.42699700	0.24072800
H	-4.21118100	0.27875000	1.94728700
H	-2.30121500	-1.38760100	1.63696700
H	-2.26120000	1.00148900	-0.31052200
C	-4.23084200	2.31706200	1.21811300
H	-3.61513700	2.72541600	2.02637100
H	-5.27894900	2.50319800	1.46566900
H	-3.99860600	2.87288400	0.30342800
H	1.72179700	-1.66882000	-0.03259700
C	0.71434400	2.52257600	-2.16707100
C	-0.33716400	3.09966600	-0.17150100
H	1.30259000	3.44673400	-2.06306900
H	0.50074000	2.36501200	-3.22557500
H	-1.32558100	3.09886400	0.29867900
H	0.02610200	4.14101200	-0.16333600
O	-0.53168200	2.68368700	-1.51199500
Br	-3.12569800	-1.93296000	-0.64641600

(*R*)-**TS2**$_{P}$-Br

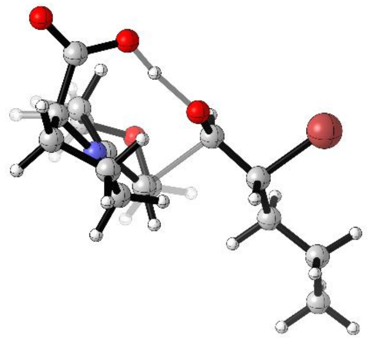

- Thermochemistry -

Zero-point correction=	0.385310 (Hartree/Particle)
Thermal correction to Energy=	0.403073
Thermal correction to Enthalpy=	0.403938
Thermal correction to Gibbs Free Energy=	0.340676
Sum of electronic and zero-point Energies=	-3512.751936
Sum of electronic and thermal Energies=	-3512.734173
Sum of electronic and thermal Enthalpies=	-3512.733308
Sum of electronic and thermal Free Energies=	-3512.796570

Number of Imaginary Frequencies = 1

E (Single Point Energy) [IEFPCM$_{(DCM)}$M06-2X/6-311++G(2d,2p)] = -3515.758121

C	0.13728100	1.55925900	0.59966900
H	-0.25719300	1.30177300	1.57841900
C	1.46579200	1.23617900	0.32622200
N	2.07367300	0.21462800	0.93421700
C	3.42732800	-0.25696000	0.62210300
H	4.14176200	0.56947000	0.62953300
C	1.48943100	-0.51034600	2.07669100
H	0.44048000	-0.73098500	1.88402500
H	1.57097600	0.11089100	2.97805000
C	2.35028200	-1.76434400	2.16511600

H	1.98689100	-2.50583200	1.44600400
H	2.33572800	-2.20714100	3.16196000
C	3.73332100	-1.25124900	1.76067200
H	4.19344100	-0.70874300	2.59130600
H	4.42226800	-2.03213800	1.43633500
C	2.17069700	1.89121500	-0.83561400
H	3.23337800	2.02759900	-0.61629100
H	2.09978500	1.22778100	-1.70812100
C	-0.62747000	-0.14362200	-0.53669700
H	-0.71824700	0.53000900	-1.40268100
C	-1.93388300	-0.46478500	0.18747000
O	0.22992200	-1.07027700	-0.52797500
C	3.58363800	-0.93377400	-0.75185300
O	2.52171900	-1.31599000	-1.41687100
O	4.71196700	-1.11668800	-1.17123900
C	-2.90833400	0.64620000	0.53033100
H	-2.39983600	1.32911700	1.22097500
C	-4.18619000	0.16201400	1.21893600
H	-4.80474100	-0.38699000	0.50320200
H	-3.91921200	-0.54771800	2.01214100
H	-1.71391100	-1.09948900	1.04860800
H	-3.16253000	1.21521500	-0.37204500
C	-4.97753300	1.32933500	1.80397600
H	-4.39529500	1.85753200	2.56589100
H	-5.90533700	0.98457700	2.26772900
H	-5.24063800	2.05056100	1.02320600
H	1.58201800	-1.12920400	-1.00754500
C	1.53538500	3.23450100	-1.17041700

C	-0.46069100	2.79340300	-0.04956900
H	1.76383100	3.97488300	-0.38969900
H	1.90901000	3.60785500	-2.12485300
H	-1.52289800	2.65700600	-0.25659600
H	-0.36361100	3.66306600	0.62062200
O	0.13477800	3.08939100	-1.29756600
Br	-2.83923600	-1.68900300	-1.08579200

(*R*)-**TS2**$_{P}$-Br-P

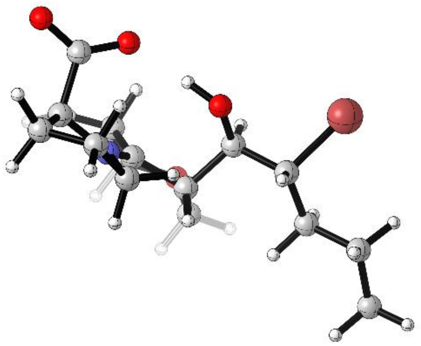

- Thermochemistry -

Zero-point correction=	0.389470 (Hartree/Particle)
Thermal correction to Energy=	0.407248
Thermal correction to Enthalpy=	0.408113
Thermal correction to Gibbs Free Energy=	0.344553
Sum of electronic and zero-point Energies=	-3512.777551
Sum of electronic and thermal Energies=	-3512.759773
Sum of electronic and thermal Enthalpies=	-3512.758908
Sum of electronic and thermal Free Energies=	-3512.822467

Number of Imaginary Frequencies = 0

E (Single Point Energy) [IEFPCM$_{(DCM)}$M06-2X/6-311++G(2d,2p)] = -3515.787444

C	-0.00803000	1.15655900	0.39302100
H	-0.18533600	1.01914300	1.46398300
C	1.46705900	1.06416900	0.10742800

N	2.23423800	0.33488000	0.84346700
C	3.63507800	-0.00621400	0.50194700
H	4.19706800	0.90216800	0.28080700
C	1.78881400	-0.42849600	2.04726400
H	0.81830300	-0.87943900	1.85267300
H	1.72430200	0.27873200	2.88009500
C	2.90186100	-1.45095200	2.25409800
H	2.71095600	-2.33513400	1.63802500
H	2.96762400	-1.76142100	3.29743300
C	4.14530100	-0.71351200	1.75736500
H	4.47854600	0.02050700	2.49765900
H	4.97927600	-1.36976800	1.50990100
C	1.96938400	1.72784600	-1.13936500
H	3.05345100	1.84147400	-1.13619300
H	1.72037400	1.05147300	-1.96759500
C	-0.70168000	-0.00700300	-0.41421100
H	-0.88737500	0.37551700	-1.42856100
C	-2.02627900	-0.44289100	0.22944000
O	0.07377100	-1.17764900	-0.43437400
C	3.68813900	-0.93422300	-0.76736800
O	2.60367900	-1.19330500	-1.35143700
O	4.83455800	-1.30356300	-1.06210200
C	-3.01716200	0.62591400	0.65712900
H	-2.50478200	1.27971000	1.37592500
C	-4.26262600	0.07397400	1.35272100
H	-4.87907700	-0.46348300	0.62648800
H	-3.95568000	-0.66027100	2.10817100
H	-1.78482000	-1.10212900	1.06823200

H	-3.31162500	1.24110600	-0.20190400
C	-5.07714200	1.18970900	2.00297400
H	-4.49474700	1.70648500	2.77262800
H	-5.98163900	0.79545900	2.47324400
H	-5.38287600	1.93228800	1.25859200
H	0.95107200	-1.08871400	-0.87876200
C	1.28324500	3.07316200	-1.37572300
C	-0.53012000	2.55204000	-0.01495600
H	1.61295800	3.81800700	-0.63761400
H	1.52506000	3.43908100	-2.37372900
H	-1.61704100	2.57164300	-0.02369300
H	-0.17836000	3.29474600	0.71589800
O	-0.11876300	2.91948500	-1.31158600
Br	-2.91132300	-1.63118800	-1.06715900

(*S*)-**TS2**$_{P}$-Br-Pre

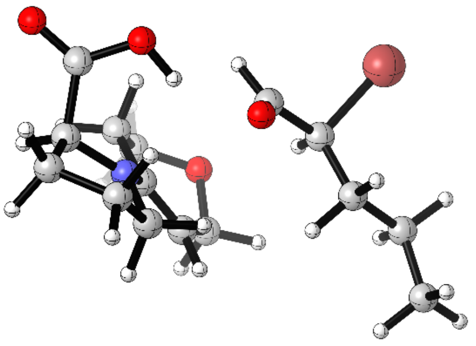

- Thermochemistry -

Zero-point correction=	0.385313 (Hartree/Particle)
Thermal correction to Energy=	0.404752
Thermal correction to Enthalpy=	0.405617
Thermal correction to Gibbs Free Energy=	0.337211
Sum of electronic and zero-point Energies=	-3512.767010
Sum of electronic and thermal Energies=	-3512.747572

Sum of electronic and thermal Enthalpies= -3512.746707
Sum of electronic and thermal Free Energies= -3512.815113

Number of Imaginary Frequencies = 0

E (Single Point Energy) [IEFPCM$_{(DCM)}$M06-2X/6-311++G(2d,2p)] = -3515.775255

C	-0.43663000	2.02210600	0.50541800
H	-0.31358800	2.53604100	-0.44215200
C	-1.41689500	1.11187900	0.68613000
N	-2.22287700	0.66243200	-0.35188100
C	-3.52886500	0.05172600	-0.13254500
H	-4.07068100	0.48399700	0.71353700
C	-2.11148600	1.25491900	-1.68465500
H	-1.09666000	1.11195300	-2.06977500
H	-2.32468600	2.33495900	-1.65176600
C	-3.17093200	0.50317900	-2.48358200
H	-2.77659700	-0.46472600	-2.81207800
H	-3.49942700	1.05462000	-3.36602000
C	-4.28917900	0.31453100	-1.45430200
H	-4.85978000	1.24103100	-1.34629800
H	-4.98727500	-0.48931300	-1.69611600
C	-1.61516300	0.45772600	2.03540300
H	-2.65990600	0.54524700	2.35347800
H	-1.39885800	-0.61686700	1.97049900
C	0.64151500	-0.96824500	-0.18526600
H	0.22860000	-1.47248000	0.70721800
C	1.97039700	-0.28513600	0.01476800
H	1.97764600	0.22682200	0.97933500
O	0.06032100	-1.00874800	-1.24987600
C	-3.43938800	-1.45204100	0.13719200

O	-2.35684100	-2.08640800	-0.31177800
O	-4.32829000	-2.05593600	0.69561000
H	-1.71189800	-1.47149900	-0.72578400
C	2.39942900	0.56968500	-1.15565800
H	1.59693700	1.30764100	-1.29990200
C	3.72475900	1.29963300	-0.95272700
H	3.72965400	1.78150700	0.03365800
H	2.42683100	-0.04236300	-2.06337700
C	3.95067900	2.34713700	-2.04076800
H	4.91340700	2.84906500	-1.91460100
H	3.93947600	1.88509800	-3.03333000
H	3.16578600	3.11004400	-2.01708200
H	4.54732200	0.57643900	-0.95114900
C	0.55247000	2.34848100	1.59157900
C	-0.73185400	1.11661000	3.09313900
H	1.56808700	2.39968200	1.18083200
H	0.34230000	3.32656800	2.05419800
H	-1.17369000	2.07291600	3.40956300
H	-0.63150700	0.47414000	3.96922900
O	0.57660500	1.34923300	2.59774300
Br	3.20060400	-1.80388000	0.25093500

(*S*)-**TS2**$_{P}$-Br

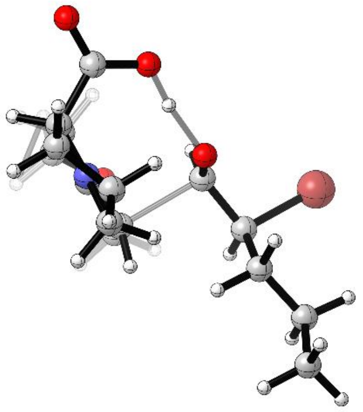

- Thermochemistry -

Zero-point correction=	0.385009 (Hartree/Particle)
Thermal correction to Energy=	0.402922
Thermal correction to Enthalpy=	0.403787
Thermal correction to Gibbs Free Energy=	0.339866
Sum of electronic and zero-point Energies=	-3512.758929
Sum of electronic and thermal Energies=	-3512.741016
Sum of electronic and thermal Enthalpies=	-3512.740151
Sum of electronic and thermal Free Energies=	-3512.804072

Number of Imaginary Frequencies = 1

E (Single Point Energy) [IEFPCM$_{(DCM)}$M06-2X/6-311++G(2d,2p)] = -3515.764418

C	-0.28413000	0.88104600	-1.51833600
H	0.14414600	0.07534300	-2.10797900
C	-1.55656000	0.71166700	-0.97697200
N	-2.06623300	-0.50046900	-0.73621900
C	-3.32666900	-0.74569700	-0.02868300
H	-4.13376400	-0.13079900	-0.43263300
C	-1.46687400	-1.74924400	-1.24372100
H	-0.39504600	-1.75380600	-1.05589900
H	-1.65245000	-1.82383400	-2.32305500
C	-2.19975600	-2.83104900	-0.45849300

H	-1.71758800	-2.96527800	0.51559500
H	-2.20214900	-3.78928000	-0.97977600
C	-3.60018900	-2.23983700	-0.28922900
H	-4.16816300	-2.33906800	-1.21863800
H	-4.18010800	-2.68856700	0.51829800
C	-2.29148700	1.91345700	-0.43514400
H	-3.36844300	1.81049500	-0.59314300
H	-2.12711400	1.96734700	0.65017800
C	0.64449600	0.45604500	0.40340600
H	0.44514200	1.49062100	0.71868600
C	2.05577100	0.21526300	-0.11082700
H	2.36610100	0.95436700	-0.84710900
O	0.05360200	-0.50676300	0.96737400
C	-3.28744600	-0.45098600	1.48206600
O	-2.13557100	-0.30574800	2.08764200
O	-4.34768800	-0.38654100	2.07826400
H	-1.24706700	-0.37929800	1.53284200
C	2.33015300	-1.20535500	-0.55968000
H	1.67568200	-1.39229100	-1.42290300
C	3.77273800	-1.46647800	-0.99085000
H	4.11310700	-0.64854700	-1.63869700
H	2.02439800	-1.89922700	0.22971000
C	3.90348400	-2.80049900	-1.72216100
H	4.94210500	-3.00108700	-1.99764200
H	3.55859700	-3.62516400	-1.08969500
H	3.30369000	-2.80734000	-2.63801500
H	4.42320600	-1.45850200	-0.11077300
C	0.17882200	2.27380700	-1.89210100

C	-1.80313300	3.19954400	-1.09331800
H	1.26201900	2.37455300	-1.78455400
H	-0.06372100	2.48838200	-2.94533800
H	-2.15503200	3.25287100	-2.13401600
H	-2.17861000	4.07020300	-0.55419400
O	-0.39062500	3.26812000	-1.06313100
Br	3.19833200	0.63424400	1.45443800

(*S*)-**TS2**$_{P}$-Br-P

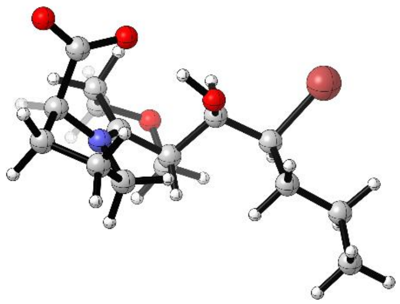

- Thermochemistry -

Zero-point correction= 0.389332 (Hartree/Particle)
Thermal correction to Energy= 0.407242
Thermal correction to Enthalpy= 0.408107
Thermal correction to Gibbs Free Energy= 0.343886
Sum of electronic and zero-point Energies= -3512.783616
Sum of electronic and thermal Energies= -3512.765706
Sum of electronic and thermal Enthalpies= -3512.764841
Sum of electronic and thermal Free Energies= -3512.829062

Number of Imaginary Frequencies = 0

E (Single Point Energy) [IEFPCM$_{(DCM)}$M06-2X/6-311++G(2d,2p)] = -3515.792756

C	-0.09034800	0.60876300	-1.21463200
H	0.10510400	-0.27463300	-1.82569500
C	-1.53425200	0.65338300	-0.81037100
N	-2.23025500	-0.42759000	-0.71698700

C	-3.55132100	-0.50268800	-0.05401000
H	-4.21781300	0.26296600	-0.45237500
C	-1.74522500	-1.79481200	-1.07614200
H	-0.71915100	-1.91945700	-0.73644500
H	-1.80317100	-1.88581500	-2.16520900
C	-2.73203200	-2.72223300	-0.37260100
H	-2.39894700	-2.90596900	0.65353400
H	-2.81328300	-3.67995300	-0.88784600
C	-4.03216300	-1.91760700	-0.37505800
H	-4.49880400	-1.94301600	-1.36457700
H	-4.75867600	-2.25030000	0.36609800
C	-2.06388400	1.98700700	-0.37741700
H	-3.15329200	2.00833600	-0.34778700
H	-1.71032300	2.14144800	0.65040900
C	0.71561200	0.51335500	0.13376600
H	0.63795800	1.49379300	0.62247000
C	2.19213900	0.22437100	-0.14315400
H	2.61148900	0.94876800	-0.84159300
O	0.22925800	-0.51113000	0.95638000
C	-3.39438200	-0.26560900	1.49271900
O	-2.23992000	-0.02281800	1.93425000
O	-4.46243900	-0.34023400	2.11749300
H	-0.64899400	-0.28209700	1.34834000
C	2.47591600	-1.20370300	-0.58066600
H	1.85435500	-1.40568000	-1.46539700
C	3.93341200	-1.46610500	-0.95707000
H	4.28425600	-0.67132700	-1.62776300
H	2.14643800	-1.89194800	0.20338000

C	4.10339800	-2.82685700	-1.62873000
H	5.15195500	-3.02233500	-1.86800200
H	3.75361900	-3.63014100	-0.97196400
H	3.52924000	-2.88109000	-2.55942700
H	4.55571700	-1.41399000	-0.05806900
C	0.26773100	1.85646000	-2.04065400
C	-1.53385200	3.11332700	-1.27235200
H	1.34511500	1.91888400	-2.19881500
H	-0.21427900	1.78730300	-3.02644500
H	-1.99537400	3.06267300	-2.26867700
H	-1.77274000	4.07849800	-0.82557800
O	-0.12698300	3.04473300	-1.39161900
Br	3.17364500	0.58404300	1.52550100

(*R*)-**TS2**$_G$-Br-Pre

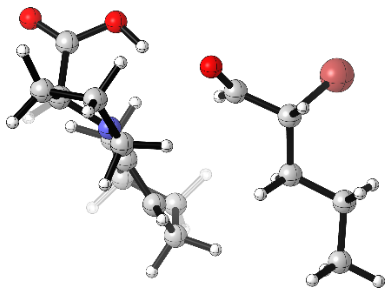

- Thermochemistry -

Zero-point correction=	0.409212 (Hartree/Particle)
Thermal correction to Energy=	0.428825
Thermal correction to Enthalpy=	0.429690
Thermal correction to Gibbs Free Energy=	0.360822
Sum of electronic and zero-point Energies=	-3476.853011
Sum of electronic and thermal Energies=	-3476.833398
Sum of electronic and thermal Enthalpies=	-3476.832533
Sum of electronic and thermal Free Energies=	-3476.901401

Number of Imaginary Frequencies = 0

E (Single Point Energy) [IEFPCM$_{(DCM)}$M06-2X/6-311++G(2d,2p)] = -3479.870764

C	0.60238200	2.11157700	0.56659800
H	0.48381500	2.05927400	1.64521100
C	1.50311900	1.31214700	-0.04342900
N	2.20782200	0.31647800	0.64404000
C	3.55822900	-0.07545700	0.24554800
H	4.14935300	0.75708100	-0.14916700
C	2.01490200	0.14492100	2.08206200
H	0.97109400	-0.11037600	2.29018800
H	2.26203500	1.06824700	2.63115000
C	2.99202900	-0.97231600	2.42448400
H	2.56663800	-1.94014500	2.13713600
H	3.23963900	-1.00549300	3.48694900
C	4.19689400	-0.62154800	1.54727400
H	4.77680100	0.18015100	2.01259000
H	4.87032500	-1.45989300	1.35718300
C	1.74019600	1.36797100	-1.53857400
H	2.81430700	1.34799500	-1.75293800
H	1.32632600	0.45712700	-1.99747900
C	-0.77158300	-1.06330000	-0.05215800
H	-0.58007300	-0.51994200	-0.99592600
C	-2.15557900	-0.91267300	0.52404400
O	0.08342100	-1.74607200	0.47119900
C	3.56487100	-1.15987800	-0.83175900
O	2.48511800	-1.93177400	-0.91762400
O	4.52725300	-1.34875900	-1.54465100
C	-2.53378700	0.54443200	0.71932600

H	-1.79137900	0.96367200	1.41298400
C	-3.93456900	0.76156400	1.28567900
H	-4.67880200	0.43042900	0.55407600
H	-4.06377100	0.13376200	2.17563200
H	-2.25037900	-1.50428500	1.43549300
H	-2.41818200	1.08256500	-0.23059600
C	-4.16377200	2.22889800	1.63958800
H	-3.44464900	2.56648300	2.39301100
H	-5.16945600	2.38745000	2.03690600
H	-4.04759600	2.86512100	0.75583300
H	1.77434300	-1.63954600	-0.30232600
C	1.11974200	2.60743500	-2.18583100
C	-0.25555500	3.11440500	-0.16472600
H	1.73367900	3.48587700	-1.94956600
H	1.12835900	2.49116300	-3.27355100
H	-1.26968200	3.10852300	0.25430400
H	0.12980100	4.12983300	0.00779000
C	-0.29715800	2.83923400	-1.66678900
H	-0.77340700	3.67161200	-2.19406500
H	-0.90424300	1.94369200	-1.86367700
Br	-3.31823300	-1.78049600	-0.79328100

(R)-**TS2**$_G$-Br

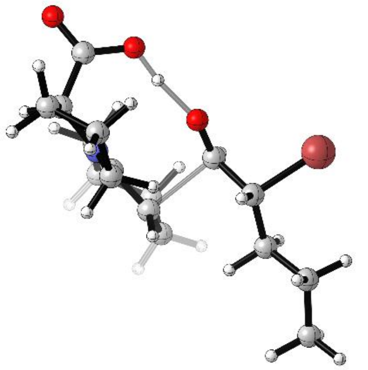

- Thermochemistry -

Zero-point correction= 0.409040 (Hartree/Particle)
Thermal correction to Energy= 0.427059
Thermal correction to Enthalpy= 0.427924
Thermal correction to Gibbs Free Energy= 0.364163
Sum of electronic and zero-point Energies= -3476.839668
Sum of electronic and thermal Energies= -3476.821649
Sum of electronic and thermal Enthalpies= -3476.820784
Sum of electronic and thermal Free Energies= -3476.884545

Number of Imaginary Frequencies = 1

E (Single Point Energy) [IEFPCM$_{(DCM)}$M06-2X/6-311++G(2d,2p)] = -3479.855265

C	0.15184500	1.56017600	0.62176600
H	-0.25228800	1.23448100	1.57667400
C	1.47856600	1.21960900	0.35550800
N	2.04712700	0.15663300	0.94152700
C	3.39825300	-0.33526400	0.65143900
H	4.13351200	0.47016300	0.71357200
C	1.42203900	-0.59099900	2.04674700
H	0.37287900	-0.78095800	1.82900100
H	1.49893800	-0.00237600	2.97060600
C	2.24652500	-1.87010800	2.11118400

H	1.87990900	-2.57535500	1.35797200
H	2.19654400	-2.34693700	3.09115100
C	3.65106800	-1.38140300	1.75626000
H	4.10714200	-0.88423800	2.61702400
H	4.32600100	-2.16850900	1.41732000
C	2.24944600	1.89633200	-0.75495600
H	3.31462300	1.91646900	-0.51134500
H	2.14876000	1.27782400	-1.65947000
C	-0.63564600	-0.12700800	-0.56011200
H	-0.74027000	0.56604600	-1.40813900
C	-1.93685900	-0.46050400	0.16696400
O	0.21844500	-1.05475500	-0.58726200
C	3.57946400	-0.95844800	-0.74364400
O	2.52715500	-1.30292300	-1.44512400
O	4.71361700	-1.13929600	-1.14840700
C	-2.90455300	0.64787800	0.53289300
H	-2.38989000	1.31096500	1.23770100
C	-4.18806200	0.15987400	1.20761000
H	-4.81278500	-0.36371200	0.47827400
H	-3.93011100	-0.57277600	1.98285100
H	-1.71536400	-1.11131500	1.01539200
H	-3.14999500	1.24012800	-0.35695300
C	-4.96600700	1.32132500	1.82180700
H	-4.37736200	1.82370600	2.59611700
H	-5.89795200	0.97642600	2.27715700
H	-5.22040200	2.06482000	1.05916500
H	1.58710300	-1.11458900	-1.04588000
C	1.75069900	3.31043900	-1.04557800

C	-0.44550100	2.83449200	0.05235400
H	2.01590900	3.97214900	-0.21168800
H	2.25804200	3.69126300	-1.93623300
H	-1.51277600	2.69867200	-0.14024300
H	-0.37593400	3.63085600	0.80650200
C	0.23935200	3.30259400	-1.23122300
H	-0.12629200	4.29835100	-1.49888300
H	-0.01807000	2.63502700	-2.06428200
Br	-2.85335000	-1.66231800	-1.12205200

(*R*)-**TS2**$_{G}$-Br-P

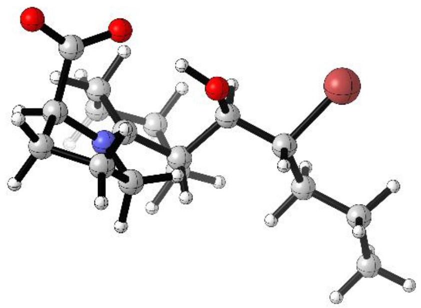

- Thermochemistry -

Zero-point correction= 0.413288 (Hartree/Particle)
Thermal correction to Energy= 0.431284
Thermal correction to Enthalpy= 0.432149
Thermal correction to Gibbs Free Energy= 0.368128
Sum of electronic and zero-point Energies= -3476.865993
Sum of electronic and thermal Energies= -3476.847998
Sum of electronic and thermal Enthalpies= -3476.847133
Sum of electronic and thermal Free Energies= -3476.911154

Number of Imaginary Frequencies = 0

E (Single Point Energy) [IEFPCM$_{(DCM)}$M06-2X/6-311++G(2d,2p)] = -3479.885376

C	-0.00853500	1.15004100	0.40904000
H	-0.17295500	0.93543900	1.46896800

C	1.46756900	1.04765800	0.11448400
N	2.22281800	0.29517300	0.84219500
C	3.62594900	-0.04937600	0.51197500
H	4.19409600	0.85847600	0.30560400
C	1.75905000	-0.49458800	2.02288100
H	0.79775700	-0.95120300	1.79839100
H	1.66786800	0.19512900	2.86770800
C	2.87362400	-1.51427600	2.23655100
H	2.69606200	-2.39309300	1.60886700
H	2.92443900	-1.83564500	3.27748700
C	4.12145800	-0.76851600	1.76573000
H	4.44229200	-0.04142700	2.51832500
H	4.96054500	-1.41985400	1.52243500
C	1.99022900	1.72219300	-1.12032700
H	3.07766300	1.69389000	-1.16284000
H	1.63558200	1.10882000	-1.96178300
C	-0.71324100	0.01926700	-0.43575500
H	-0.92007700	0.42746600	-1.43601200
C	-2.02897900	-0.44502300	0.20713100
O	0.05908000	-1.15311700	-0.51030800
C	3.68674400	-0.96451800	-0.76457100
O	2.60880800	-1.19519800	-1.37212800
O	4.83136400	-1.35331800	-1.04402800
C	-3.02112700	0.60161600	0.68128300
H	-2.50495400	1.22359500	1.42450500
C	-4.26437200	0.02032000	1.35635500
H	-4.88870400	-0.47794800	0.60922200
H	-3.95575900	-0.75211000	2.07213700

H	-1.77399900	-1.12901100	1.02162300
H	-3.31620200	1.25392500	-0.14984100
C	-5.06896300	1.10596300	2.06732500
H	-4.47892900	1.58021200	2.85822700
H	-5.97295700	0.69355700	2.52285800
H	-5.37484100	1.88687800	1.36325100
H	0.94645600	-1.04994800	-0.93038700
C	1.46090500	3.15094700	-1.28145300
C	-0.53144700	2.57495300	0.11223800
H	1.86879500	3.79050700	-0.48961600
H	1.82157900	3.54512400	-2.23479300
H	-1.61956900	2.58183600	0.15077800
H	-0.18754100	3.23014400	0.92165800
C	-0.05955300	3.15347100	-1.21977600
H	-0.44698400	4.17116500	-1.32243400
H	-0.46351700	2.57494800	-2.05990800
Br	-2.92130700	-1.60042300	-1.11505000

(*S*)-**TS2**$_G$-Br-Pre

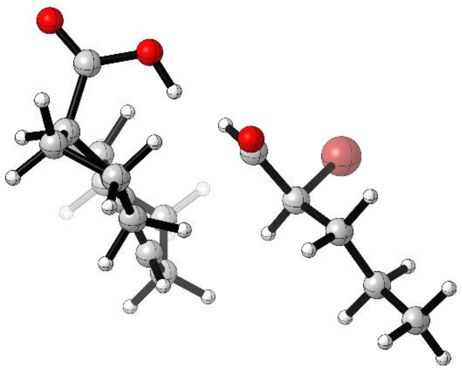

- Thermochemistry -

Zero-point correction= 0.408825 (Hartree/Particle)

Thermal correction to Energy= 0.428488
Thermal correction to Enthalpy= 0.429353
Thermal correction to Gibbs Free Energy= 0.360308
Sum of electronic and zero-point Energies= -3476.855490
Sum of electronic and thermal Energies= -3476.835827
Sum of electronic and thermal Enthalpies= -3476.834962
Sum of electronic and thermal Free Energies= -3476.904008

Number of Imaginary Frequencies = 0

E (Single Point Energy) [IEFPCM$_{(DCM)}$M06-2X/6-311++G(2d,2p)] = -3479.871179

C	-0.45443400	0.51994300	-2.02113400
H	-0.27509400	-0.44780000	-2.48087000
C	-1.45229600	0.65825100	-1.11881400
N	-2.18101600	-0.43512100	-0.64598100
C	-3.52107900	-0.29770100	-0.08337600
H	-4.11447900	0.48408200	-0.56609400
C	-1.97197100	-1.76495200	-1.21733600
H	-0.93446500	-2.07896600	-1.06554100
H	-2.17973600	-1.76528100	-2.29932000
C	-2.97388700	-2.62716200	-0.45842000
H	-2.56696600	-2.89927800	0.52174200
H	-3.22497200	-3.54589700	-0.99121500
C	-4.17087400	-1.68432100	-0.31028900
H	-4.73897900	-1.65098200	-1.24390700
H	-4.85667300	-1.95681100	0.49454000
C	-1.77710200	1.99299900	-0.47864100
H	-2.85226400	2.18837800	-0.55092800
H	-1.55732400	1.93518000	0.59811600
C	0.43778500	2.78175200	-1.39915000
C	0.63423800	-0.25328800	0.86346700
H	0.20265000	0.72944200	1.12075800

C	1.97743300	-0.21680000	0.17408000
H	1.88670800	0.35750700	-0.74929300
O	0.06136200	-1.28347500	1.15013000
C	-3.50075600	0.03673900	1.40905800
O	-2.41926700	-0.32762000	2.09714000
O	-4.44066500	0.56503800	1.96133200
H	-1.73038000	-0.72170700	1.51828800
C	2.59821200	-1.57705200	-0.05087600
H	1.84481700	-2.16998300	-0.58808200
C	3.88926800	-1.53745200	-0.86356800
H	3.71078700	-0.98461800	-1.79484100
H	2.75767000	-2.07047900	0.91448200
C	4.39503800	-2.94226800	-1.18236700
H	5.32720500	-2.90744800	-1.75198800
H	4.58302500	-3.50546400	-0.26259200
H	3.65921500	-3.49876800	-1.77157700
H	4.65279500	-0.98187000	-0.30805300
C	0.45803500	1.65204900	-2.42722900
C	-1.01084200	3.16224800	-1.10529200
H	1.47922300	1.27299400	-2.56786200
H	0.16167000	2.05059800	-3.40811400
H	-1.49565000	3.45026500	-2.04644200
H	-1.06699800	4.02717800	-0.43821500
H	0.92834200	2.45831700	-0.46940600
H	1.00055300	3.64421900	-1.76897300
Br	3.07755400	0.93096000	1.32650800

(*S*)-**TS2**$_{G}$-Br

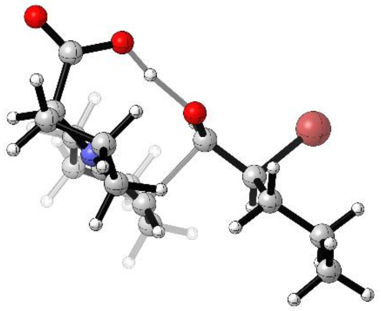

- Thermochemistry -

Zero-point correction=	0.408948 (Hartree/Particle)
Thermal correction to Energy=	0.427032
Thermal correction to Enthalpy=	0.427897
Thermal correction to Gibbs Free Energy=	0.363804
Sum of electronic and zero-point Energies=	-3476.846872
Sum of electronic and thermal Energies=	-3476.828788
Sum of electronic and thermal Enthalpies=	-3476.827923
Sum of electronic and thermal Free Energies=	-3476.892016

Number of Imaginary Frequencies = 1

E (Single Point Energy) [IEFPCM$_{(DCM)}$M06-2X/6-311++G(2d,2p)] = -3479.861757

C	-0.29237000	0.91811500	-1.49358600
H	0.13052400	0.09754900	-2.06656500
C	-1.55985600	0.72975600	-0.94659600
N	-2.05664600	-0.50083500	-0.74346100
C	-3.31403700	-0.78386200	-0.04529500
H	-4.13414300	-0.18030100	-0.43961500
C	-1.45098800	-1.72666200	-1.29729500
H	-0.37662000	-1.72894900	-1.12828700
H	-1.65364700	-1.77340100	-2.37557400
C	-2.15458200	-2.84032400	-0.53123000
H	-1.65731900	-2.98799600	0.43334800
H	-2.14651900	-3.78595400	-1.07529100

C	-3.56224300	-2.27848100	-0.33105300
H	-4.14122000	-2.37021900	-1.25442600
H	-4.12327900	-2.75478100	0.47432500
C	-2.32610700	1.89976200	-0.37177800
H	-3.40061100	1.72125300	-0.45411400
H	-2.10395000	1.95435600	0.70534000
C	-0.45748300	3.42192100	-1.08284700
C	0.63631300	0.40332300	0.44116300
H	0.41972100	1.41115800	0.82203900
C	2.05004100	0.22121300	-0.08437600
H	2.34083600	1.00358300	-0.78247400
O	0.05687300	-0.59723300	0.94442800
C	-3.28263800	-0.51467500	1.46893400
O	-2.13008200	-0.41108800	2.08406300
O	-4.34346600	-0.43620600	2.06272000
H	-1.24808800	-0.48284500	1.52383500
C	2.35682200	-1.17009500	-0.59884300
H	1.69919600	-1.33560700	-1.46384100
C	3.80169300	-1.37288100	-1.05289700
H	4.11389800	-0.51757000	-1.66562300
H	2.07637200	-1.90653600	0.16089400
C	3.96085600	-2.66817000	-1.84563500
H	5.00160500	-2.82737600	-2.13960500
H	3.64505500	-3.53000800	-1.24853400
H	3.35195000	-2.64984100	-2.75528900
H	4.46005800	-1.38728100	-0.17869900
C	0.18947800	2.29908600	-1.89346200
C	-1.96867700	3.22751400	-1.04117400

H	1.28023100	2.35982500	-1.81221300
H	-0.03217300	2.45459700	-2.95849400
H	-2.36699300	3.23671800	-2.06335800
H	-2.45575500	4.04169000	-0.49751200
H	-0.06378900	3.43119100	-0.05841000
H	-0.20484200	4.38942000	-1.52658500
Br	3.18748800	0.58817600	1.50100200

(*S*)-**TS2**$_{G}$-Br-P

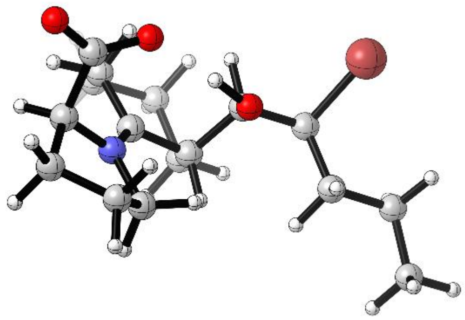

- Thermochemistry -

Zero-point correction=	0.413496 (Hartree/Particle)
Thermal correction to Energy=	0.431581
Thermal correction to Enthalpy=	0.432446
Thermal correction to Gibbs Free Energy=	0.368030
Sum of electronic and zero-point Energies=	-3476.872903
Sum of electronic and thermal Energies=	-3476.854818
Sum of electronic and thermal Enthalpies=	-3476.853953
Sum of electronic and thermal Free Energies=	-3476.918369

Number of Imaginary Frequencies = 0

E (Single Point Energy) [IEFPCM$_{(DCM)}$M06-2X/6-311++G(2d,2p)] = -3479.891844

C	-0.06948000	0.67345200	-1.12478800
H	0.07965500	-0.17879400	-1.78857400
C	-1.51537000	0.75681100	-0.72238300
N	-2.28125300	-0.27936600	-0.80187700

C	-3.59883900	-0.37968000	-0.13503900
H	-4.21617200	0.48550000	-0.37638900
C	-1.89404100	-1.60133300	-1.38587500
H	-0.88201500	-1.85652500	-1.08116300
H	-1.95369200	-1.50549500	-2.47439500
C	-2.94585800	-2.56280400	-0.83963100
H	-2.62699700	-2.94483400	0.13507000
H	-3.09681000	-3.40896200	-1.51101300
C	-4.18370200	-1.67976400	-0.68509500
H	-4.65317900	-1.50045900	-1.65741000
H	-4.93103300	-2.08275800	-0.00158600
C	-1.97501800	2.03734400	-0.08633700
H	-3.05889200	2.06652200	0.01615600
H	-1.57069000	2.03098700	0.93526300
C	0.03815900	3.22451500	-1.07692100
C	0.68458900	0.36553600	0.21962800
H	0.51564900	1.19806800	0.91499700
C	2.19166400	0.25584000	-0.00805300
H	2.59201700	1.19338300	-0.39529200
O	0.22293000	-0.83903700	0.77183900
C	-3.40071700	-0.43237300	1.42311100
O	-2.22205500	-0.38057200	1.86536000
O	-4.46217400	-0.51827100	2.05895300
H	-0.64648000	-0.69367000	1.21707100
C	2.61321600	-0.92838200	-0.86066300
H	2.11450400	-0.81478200	-1.83463800
C	4.11712700	-1.02577500	-1.11273800
H	4.50138200	-0.03555000	-1.38957600

H	2.23183300	-1.85087000	-0.41047600
C	4.43921000	-2.03703000	-2.21060200
H	5.51801200	-2.12578600	-2.36308400
H	4.05443300	-3.02845700	-1.94963800
H	3.98657400	-1.74207000	-3.16285300
H	4.62507600	-1.30867100	-0.18553000
C	0.39056700	1.95254400	-1.84635400
C	-1.46930900	3.27812400	-0.84027900
H	1.46282200	1.88892200	-2.05078000
H	-0.10383200	1.98293300	-2.82484600
H	-1.98612000	3.35072500	-1.80468400
H	-1.74510400	4.16297900	-0.26125700
H	0.57128400	3.26188700	-0.11871900
H	0.35771300	4.09964300	-1.64968200
Br	3.02817300	0.10950200	1.76768700

(*R*)-**TS2**$_{\mathrm{O}}$-Br-Pre

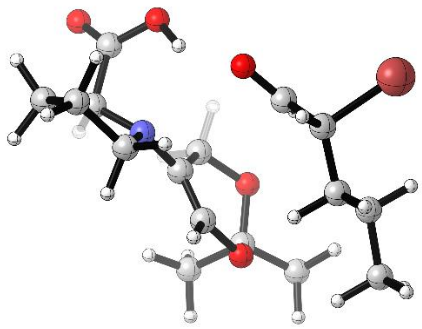

- Thermochemistry -

Zero-point correction= 0.416888 (Hartree/Particle)
Thermal correction to Energy= 0.438271
Thermal correction to Enthalpy= 0.439136
Thermal correction to Gibbs Free Energy= 0.366796
Sum of electronic and zero-point Energies= -3627.231271

Sum of electronic and thermal Energies= -3627.209888
Sum of electronic and thermal Enthalpies= -3627.209023
Sum of electronic and thermal Free Energies= -3627.281363

Number of Imaginary Frequencies = 0

E (Single Point Energy) [IEFPCM$_{(DCM)}$M06-2X/6-311++G(2d,2p)] = -3630.304563

C	0.56848300	1.42459900	1.15506900
H	0.42870000	1.14218500	2.19135500
C	1.38012700	0.78196600	0.29687600
N	2.11703400	-0.36007000	0.62963200
C	3.47602300	-0.51815000	0.10469500
H	3.96416200	0.43855400	-0.11162300
C	2.01936600	-0.88835800	1.98935900
H	1.01102000	-1.27920200	2.16362500
H	2.22678300	-0.10475900	2.73687800
C	3.10843600	-1.95345400	2.01572000
H	2.76123600	-2.85863900	1.50616000
H	3.41018300	-2.22018000	3.02990900
C	4.23098300	-1.27585200	1.22369500
H	4.74467600	-0.54616800	1.85548400
H	4.97636600	-1.96500400	0.82276300
C	1.43058900	1.23883700	-1.14041900
H	2.40021100	1.69417500	-1.39287000
H	1.27789300	0.38768000	-1.81317400
O	-0.18451800	2.51856000	0.80381400
O	0.36996700	2.14105100	-1.41026600
C	0.20992200	3.14142900	-0.42103600
C	-0.94591800	4.01117500	-0.86429000
H	-1.80737700	3.38748100	-1.10911900

H	-0.65575500	4.58529200	-1.74678300
C	1.48673800	3.94930600	-0.20328100
H	1.26376700	4.80066800	0.44322300
H	1.85094600	4.32011500	-1.16486500
H	2.27052000	3.35553500	0.27176900
C	-0.97716100	-1.20207000	-0.29251500
H	-0.97781600	-0.39582300	-1.04698700
C	-2.18142200	-1.29882100	0.61414600
O	-0.06678700	-2.00246700	-0.22369600
C	3.50920700	-1.32990800	-1.19330500
O	2.41458200	-2.01509200	-1.50666000
O	4.50217900	-1.37697600	-1.88673200
H	-1.21654700	4.69942300	-0.06061000
C	-2.67176300	0.01158600	1.21242400
H	-3.51385900	-0.21967100	1.87486600
C	-3.07938500	1.09653500	0.21817700
H	-2.23370000	1.33503100	-0.43759700
H	-3.88703300	0.71990800	-0.41867100
H	-1.86175900	0.38769900	1.85191400
C	-3.53364600	2.36256700	0.94037600
H	-3.87702300	3.12037700	0.23053400
H	-4.36099400	2.14422100	1.62397400
H	-2.70838700	2.79026600	1.51812200
H	1.68695500	-1.84671400	-0.86473100
H	-1.97313600	-2.03943100	1.38650700
Br	-3.55674700	-2.13547000	-0.51123700

(*R*)-**TS2**$_{O}$-Br

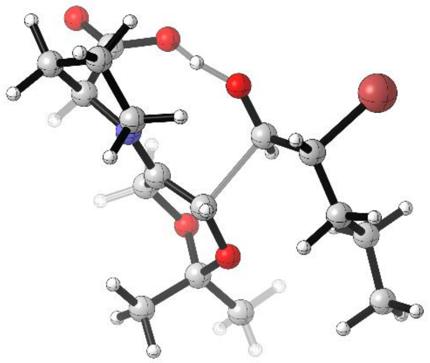

- Thermochemistry -

Zero-point correction=	0.415814 (Hartree/Particle)
Thermal correction to Energy=	0.435581
Thermal correction to Enthalpy=	0.436446
Thermal correction to Gibbs Free Energy=	0.369445
Sum of electronic and zero-point Energies=	-3627.219690
Sum of electronic and thermal Energies=	-3627.199924
Sum of electronic and thermal Enthalpies=	-3627.199059
Sum of electronic and thermal Free Energies=	-3627.266059

Number of Imaginary Frequencies = 1

E (Single Point Energy) [IEFPCM$_{(DCM)}$M06-2X/6-311++G(2d,2p)] = -3630.289223

C	-0.02963900	0.97221200	0.91302300
H	-0.17980400	0.58414600	1.91630200
C	1.24423500	0.96915000	0.34581000
N	2.17701700	0.10312400	0.72712400
C	3.48121200	-0.03875400	0.07116000
H	3.91513800	0.94210000	-0.14353900
C	2.07452500	-0.71380500	1.95331700
H	1.12209300	-1.24246200	1.97602000
H	2.15218700	-0.05194000	2.82554100
C	3.26919500	-1.65528000	1.83607300

H	2.99630900	-2.52054000	1.22299300
H	3.60628900	-2.01259100	2.80978300
C	4.31458700	-0.79817900	1.11814600
H	4.75895300	-0.07936700	1.81239200
H	5.11538900	-1.37082400	0.64966300
C	1.49993200	1.82805400	-0.86820000
H	2.31692800	2.53340900	-0.65672100
H	1.79798900	1.20668700	-1.71904800
O	-0.93426300	1.97099400	0.59006300
O	0.32684000	2.50020000	-1.26840000
C	-0.42014600	3.04601000	-0.19568500
C	-1.61303300	3.74198700	-0.81177000
H	-2.15022300	3.04662300	-1.46042900
H	-1.27491100	4.59752900	-1.40006300
C	0.41442400	3.98132300	0.67490600
H	-0.24411800	4.48874200	1.38252300
H	0.90390500	4.72903600	0.04560900
H	1.17643000	3.44704900	1.24751700
C	-0.60094700	-0.72918000	-0.21475700
H	-0.84675300	-0.08799600	-1.07392400
C	-1.78213900	-1.17988200	0.63844400
O	0.35933300	-1.55898300	-0.29690900
C	3.45504200	-0.79432000	-1.27521400
O	2.34974600	-1.35381800	-1.68398500
O	4.49091800	-0.82898100	-1.91672000
H	-2.28276600	4.09114700	-0.02345500
C	-2.89193000	-0.19692900	0.97684300
H	-3.63859200	-0.73517100	1.57157700

C	-3.57334300	0.47792400	-0.21219100
H	-2.81581300	0.89740700	-0.88159400
H	-4.13108300	-0.26859500	-0.78760800
H	-2.47024000	0.57295500	1.63187100
C	-4.50945400	1.59249200	0.24699100
H	-4.99460700	2.07963200	-0.60342300
H	-5.29300000	1.20089500	0.90454200
H	-3.95328400	2.35459700	0.80320000
H	1.48786300	-1.36602300	-1.04656200
H	-1.39034900	-1.65284600	1.54070400
Br	-2.56167900	-2.71307700	-0.34878600

(*R*)-**TS2**$_{O}$-Br-P

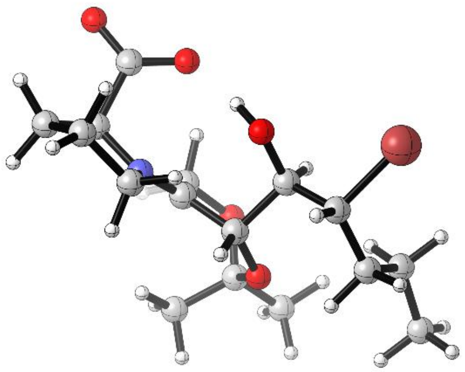

- Thermochemistry -

Zero-point correction= 0.419636 (Hartree/Particle)
Thermal correction to Energy= 0.439624
Thermal correction to Enthalpy= 0.440489
Thermal correction to Gibbs Free Energy= 0.372588
Sum of electronic and zero-point Energies= -3627.242525
Sum of electronic and thermal Energies= -3627.222537
Sum of electronic and thermal Enthalpies= -3627.221672
Sum of electronic and thermal Free Energies= -3627.289573

Number of Imaginary Frequencies = 0

E (Single Point Energy) [IEFPCM$_{(DCM)}$M06-2X/6-311++G(2d,2p)] = -3630.315195

C	-0.07186600	0.64358700	0.74179400
H	-0.00607400	0.41820800	1.81199400
C	1.30414900	0.81108500	0.15243100
N	2.32377800	0.20239900	0.64106400
C	3.61878100	0.09854100	-0.06186800
H	3.90055600	1.07079600	-0.47307300
C	2.34284000	-0.59859400	1.90439700
H	1.47937000	-1.25949800	1.92832200
H	2.30803200	0.11471100	2.73380100
C	3.67841800	-1.33631600	1.83741000
H	3.55190600	-2.28561800	1.30790900
H	4.06478400	-1.54554000	2.83531000
C	4.56905600	-0.39232600	1.02670700
H	4.90774100	0.44623600	1.64262800
H	5.43467400	-0.88091600	0.58079100
C	1.41714700	1.62400400	-1.10998700
H	2.25559000	2.32892300	-1.02548900
H	1.61258000	0.94680900	-1.94787000
O	-0.89524400	1.78310600	0.58431300
O	0.20795800	2.29390400	-1.37430700
C	-0.39454600	2.85117500	-0.22019600
C	-1.59342300	3.64156100	-0.69553200
H	-2.21432900	3.01903000	-1.34257400
H	-1.25528200	4.51706500	-1.25375200
C	0.57054600	3.71252500	0.59367000
H	0.00272200	4.23493800	1.36574400
H	1.05130600	4.44980100	-0.05441900

H	1.34621800	3.12816500	1.09756500
C	-0.69579400	-0.59532600	-0.01449000
H	-0.94716600	-0.25312600	-1.02862100
C	-1.94621400	-1.09101600	0.71862900
O	0.20487000	-1.66589600	-0.02105300
C	3.50420500	-0.91768800	-1.26587700
O	2.35083500	-1.31094400	-1.58471900
O	4.59151600	-1.19390100	-1.78667400
H	-2.18094100	3.96980700	0.16394100
C	-3.05164900	-0.09281800	1.02720400
H	-3.81850300	-0.61726000	1.60847000
C	-3.69429000	0.57828700	-0.18435500
H	-2.91132000	0.97011500	-0.84198300
H	-4.24926800	-0.16805600	-0.76235600
H	-2.62765700	0.67473200	1.68526300
C	-4.62759900	1.71152200	0.23280800
H	-5.07980000	2.19481500	-0.63779600
H	-5.43644800	1.33758800	0.86953300
H	-4.08220600	2.47436300	0.79853800
H	0.93954800	-1.53444400	-0.67046400
H	-1.61169100	-1.57258900	1.64115600
Br	-2.68453800	-2.59243400	-0.31877100

(*S*)-**TS2**$_{O}$-Br-Pre

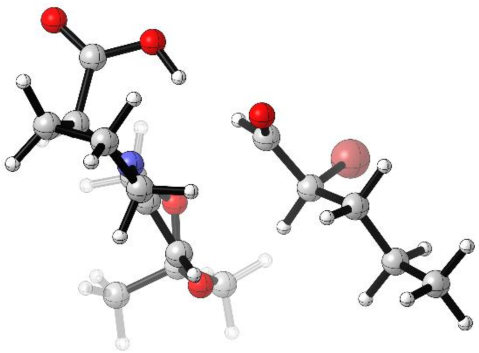

- Thermochemistry -

Zero-point correction= 0.416363 (Hartree/Particle)
Thermal correction to Energy= 0.438035
Thermal correction to Enthalpy= 0.438900
Thermal correction to Gibbs Free Energy= 0.365643
Sum of electronic and zero-point Energies= -3627.237041
Sum of electronic and thermal Energies= -3627.215369
Sum of electronic and thermal Enthalpies= -3627.214504
Sum of electronic and thermal Free Energies= -3627.287761

Number of Imaginary Frequencies = 0

E (Single Point Energy) [IEFPCM$_{(DCM)}$M06-2X/6-311++G(2d,2p)] = -3630.306273

C	0.39350900	0.85467000	1.59406200
H	0.30557600	0.19223300	2.44618200
C	1.34215700	0.75132700	0.64500900
N	2.29612400	-0.26840400	0.62732700
C	3.59241000	-0.06413100	-0.01815600
H	3.87580500	0.99116600	-0.08724000
C	2.41069400	-1.14998100	1.79082000
H	1.52739300	-1.79410500	1.85983900
H	2.48679800	-0.55895100	2.71780500
C	3.70308000	-1.91448200	1.52241700
H	3.51712400	-2.74092900	0.82805000

H	4.13979600	-2.32489300	2.43414000
C	4.58526500	-0.84491100	0.87110100
H	4.97798400	-0.16736600	1.63432000
H	5.42494600	-1.24316600	0.29892800
C	1.28840300	1.69431400	-0.53246000
H	2.02616000	2.50416500	-0.44992800
H	1.48686800	1.16395600	-1.46974100
O	-0.63954600	1.76126400	1.54198700
O	-0.02480000	2.21944800	-0.65264200
C	-0.52033000	2.78463700	0.54689400
C	-1.92709900	3.25738200	0.25102500
H	-2.40761100	3.58086100	1.17644500
H	-1.89602800	4.09288200	-0.45153400
C	0.37679900	3.89563300	1.07961100
H	-0.13446100	4.40522600	1.89875400
H	0.58253500	4.61696900	0.28451500
H	1.32097100	3.50080100	1.46223800
C	-0.52634000	-1.01427400	-0.80496900
H	-0.17805000	-0.26999000	-1.54214200
C	-1.85927800	-0.70892400	-0.16563000
O	0.12645600	-2.00530600	-0.55067600
C	3.61809400	-0.61632700	-1.44808400
O	2.61730000	-1.41669100	-1.80817600
O	4.52304600	-0.36073000	-2.21134200
H	-2.50583900	2.44151300	-0.19030200
C	-2.41262900	-1.82129100	0.69655800
H	-2.55710900	-2.72289400	0.09000000
C	-3.69765000	-1.44694700	1.43024700

H	-3.54326500	-0.50034000	1.96409300
H	-4.49405700	-1.26972100	0.69912700
H	-1.62566900	-2.05486300	1.42732200
C	-4.12496400	-2.53545500	2.41191500
H	-4.28571600	-3.48574000	1.89248600
H	-3.35793200	-2.69696700	3.17610500
H	-5.05551800	-2.26791400	2.91912700
H	1.96094300	-1.52099500	-1.08374300
H	-1.77249600	0.23166000	0.38673300
Br	-3.03326800	-0.24385900	-1.67109200

(*S*)-**TS2**$_O$-Br

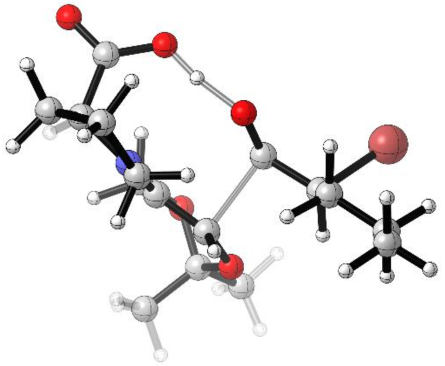

- Thermochemistry -

Zero-point correction= 0.415358 (Hartree/Particle)
Thermal correction to Energy= 0.435442
Thermal correction to Enthalpy= 0.436307
Thermal correction to Gibbs Free Energy= 0.368103
Sum of electronic and zero-point Energies= -3627.225054
Sum of electronic and thermal Energies= -3627.204970
Sum of electronic and thermal Enthalpies= -3627.204105
Sum of electronic and thermal Free Energies= -3627.272308

Number of Imaginary Frequencies = 1

E (Single Point Energy) [IEFPCM$_{(DCM)}$M06-2X/6-311++G(2d,2p)] = -3630.293793

C	0.18356100	0.85330300	1.06863300
H	-0.17421500	0.25519300	1.90038100
C	1.46354600	0.65354700	0.55412000
N	2.10126300	-0.50436000	0.69486800
C	3.35832100	-0.83491200	0.01741000
H	4.04897000	0.01245200	0.04131400
C	1.68702200	-1.55143500	1.65149200
H	0.63564000	-1.79993400	1.50675000
H	1.84277100	-1.17808800	2.67164300
C	2.61639800	-2.71441600	1.31317500
H	2.18470700	-3.30631500	0.49932900
H	2.77807600	-3.37060400	2.16917400
C	3.89479600	-2.01465200	0.84505600
H	4.45237700	-1.62634300	1.70215000
H	4.55794900	-2.64679500	0.25403200
C	2.04250700	1.70598300	-0.35905000
H	2.97057700	2.10453200	0.07625000
H	2.28272300	1.27660400	-1.33743500
O	-0.42134900	2.09531400	1.01543900
O	1.10185100	2.72785800	-0.60506100
C	0.39828000	3.16724800	0.54309600
C	-0.54466200	4.25318800	0.07719200
H	-1.19757600	4.55104300	0.89980200
H	0.02830700	5.11974200	-0.25881200
C	1.33458400	3.63156800	1.65370400
H	0.74312900	4.09176400	2.44753900
H	2.03548800	4.37081300	1.25781100

H	1.90013500	2.80461100	2.09067200
C	-0.64171400	-0.15006100	-0.55077400
H	-0.43993100	0.69907900	-1.22182600
C	-2.05647400	-0.16083600	0.00565000
O	-0.08004700	-1.27384000	-0.76237900
C	3.20720200	-1.22433500	-1.46954400
O	2.01888500	-1.36369300	-1.98676600
O	4.23230300	-1.38554500	-2.11044700
H	-1.15088900	3.87586400	-0.74925000
C	-2.37130600	-1.37640300	0.85388400
H	-2.19339200	-2.28210600	0.26572100
C	-3.77603600	-1.38173600	1.45269500
H	-3.97503400	-0.41077900	1.92355800
H	-4.51244600	-1.49861900	0.65086100
H	-1.64140000	-1.39071800	1.67668400
C	-3.94151700	-2.50114200	2.47815700
H	-3.74469600	-3.47718100	2.02257800
H	-3.24489300	-2.37500800	3.31336400
H	-4.95582800	-2.51941600	2.88538300
H	1.12880700	-1.28567900	-1.37796000
H	-2.30266900	0.78219100	0.49268500
Br	-3.22016300	-0.16502800	-1.59663300

(*S*)-**TS2**$_O$-Br-P

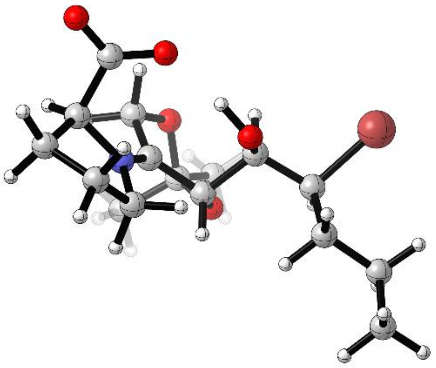

- Thermochemistry -

Zero-point correction=	0.419397 (Hartree/Particle)
Thermal correction to Energy=	0.439742
Thermal correction to Enthalpy =	0.440607
Thermal correction to Gibbs Free Energy=	0.370977
Sum of electronic and zero-point Energies=	-3627.245416
Sum of electronic and thermal Energies=	-3627.225071
Sum of electronic and thermal Enthalpies=	-3627.224206
Sum of electronic and thermal Free Energies=	-3627.293836

Number of Imaginary Frequencies = 0

E (Single Point Energy) [IEFPCM$_{(DCM)}$M06-2X/6-311++G(2d,2p)] = -3630.318050

C	0.06426900	0.69021800	0.78059800
H	-0.08317400	0.17235700	1.73166700
C	1.50231200	0.62742200	0.36307500
N	2.25785700	-0.35207000	0.70689900
C	3.55222300	-0.63429000	0.05802100
H	4.11902400	0.29106100	-0.06912600
C	1.90992400	-1.42845700	1.68613900
H	0.91471700	-1.81213500	1.46852300
H	1.93782100	-0.97503900	2.68159500
C	3.02111000	-2.45964800	1.49174100
H	2.72949000	-3.17902100	0.72065900

H	3.21927100	-3.00462300	2.41508600
C	4.21202300	-1.62483600	1.01337900
H	4.67691300	-1.09453300	1.84996600
H	4.97219100	-2.20621500	0.49264100
C	1.97478500	1.67961400	-0.60254800
H	2.90044400	2.14001100	-0.22941000
H	2.18204400	1.21067000	-1.57019900
O	-0.45120200	1.99612000	0.91572000
O	0.96023000	2.63366800	-0.81050200
C	0.32190600	3.07371200	0.37503800
C	-0.66602000	4.14088900	-0.04001300
H	-1.28117200	4.42333600	0.81634500
H	-0.13083700	5.02020700	-0.40428500
C	1.31364700	3.56316900	1.42616500
H	0.75698000	4.01922400	2.24682500
H	1.98396100	4.30907900	0.99145800
H	1.91284200	2.75006800	1.84677900
C	-0.71402900	-0.05293300	-0.37201400
H	-0.57769000	0.56148000	-1.27284400
C	-2.19786200	-0.08578400	-0.01337300
O	-0.25153100	-1.35786400	-0.54780700
C	3.30593400	-1.25726300	-1.37266200
O	2.12355500	-1.26116400	-1.80654800
O	4.33831000	-1.65979700	-1.92250600
H	-1.30658300	3.74926800	-0.83322400
C	-2.54868400	-1.11124600	1.05183100
H	-2.27389200	-2.10910400	0.69628700
C	-4.01022000	-1.07793700	1.49551200

H	-4.30302300	-0.04098800	1.70304500
H	-4.64608900	-1.42456500	0.67444900
H	-1.91900800	-0.90171500	1.92894600
C	-4.24107900	-1.94307600	2.73245000
H	-3.94877300	-2.98074900	2.54047000
H	-3.65198800	-1.58089200	3.58135600
H	-5.29368000	-1.94055500	3.02746500
H	0.59418200	-1.35366100	-1.06043300
H	-2.53898300	0.91462400	0.25351800
Br	-3.18018000	-0.51137500	-1.66407900

(*R*)-**TS2**$_{T}$-Br-Pre

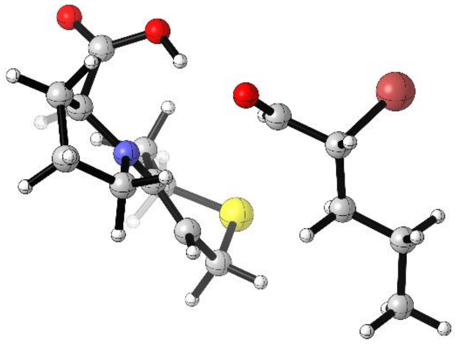

- Thermochemistry -

Zero-point correction=	0.381780 (Hartree/Particle)
Thermal correction to Energy=	0.401726
Thermal correction to Enthalpy=	0.402591
Thermal correction to Gibbs Free Energy=	0.332897
Sum of electronic and zero-point Energies=	-3835.735819
Sum of electronic and thermal Energies=	-3835.715872
Sum of electronic and thermal Enthalpies=	-3835.715007
Sum of electronic and thermal Free Energies=	-3835.784701

Number of Imaginary Frequencies = 0

E (Single Point Energy) [IEFPCM$_{(DCM)}$M06-2X/6-311++G(2d,2p)] = -3838.749773

C	0.61124100	1.84783300	0.82250100
H	0.46340800	1.65451400	1.88004200
C	1.58017000	1.17870000	0.15450100
N	2.35798800	0.20314100	0.77551200
C	3.57450300	-0.34889300	0.19126600
H	4.31427300	0.41897100	-0.07130400
C	2.35550500	0.07954800	2.23849900
H	1.52720800	-0.56418000	2.56170000
H	2.22396800	1.06324500	2.70158700
C	3.72698100	-0.50885100	2.57779400
H	3.69998900	-1.15217400	3.45899100
H	4.44020500	0.29975600	2.76451600
C	4.11825700	-1.26093700	1.30817600
H	5.19098300	-1.42739300	1.20096500
H	3.60971700	-2.23103800	1.27146300
C	1.84792400	1.36843200	-1.32607400
H	2.92857600	1.45584000	-1.49076500
H	1.52032500	0.47111000	-1.86784200
C	-0.74351700	-1.13372300	0.07756900
H	-0.53746400	-0.52755400	-0.82351200
C	-2.15295600	-1.07142800	0.60390600
O	0.11381400	-1.82011100	0.59718200
C	3.34637900	-1.19421400	-1.05637300
O	2.22864500	-1.91483000	-1.09664500
O	4.16722000	-1.26460200	-1.94446500
C	-2.58690500	0.35238500	0.90027200
H	-1.88941300	0.73179300	1.66086600
C	-4.01623400	0.48139000	1.41955200

H	-4.71740100	0.18574800	0.63239200
H	-4.16148400	-0.21687400	2.25273600
H	-2.26812800	-1.74057200	1.45745600
H	-2.44725300	0.96468100	0.00006300
C	-4.30922700	1.90950000	1.87306800
H	-4.17511100	2.61523600	1.04632900
H	-5.33625200	2.00742400	2.23359600
H	-3.63619600	2.20875100	2.68321700
H	1.63474800	-1.75489800	-0.32431500
C	1.18699300	2.59258500	-1.94716600
C	-0.30108200	2.89488700	0.24050000
H	1.66741800	3.51342800	-1.60179800
H	1.27415100	2.55304100	-3.03428100
H	-1.28362800	2.84886200	0.71832800
H	0.08282500	3.90806600	0.41100900
S	-0.58135600	2.69259400	-1.54457500
Br	-3.22386600	-1.85474900	-0.83866700

(*R*)-**TS2**$_{T}$-Br

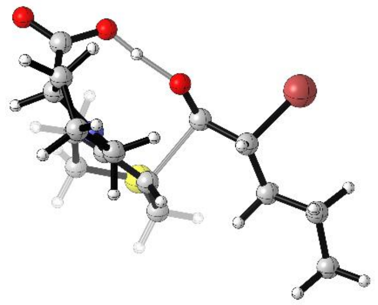

- Thermochemistry -

Zero-point correction= 0.381358 (Hartree/Particle)
Thermal correction to Energy= 0.399655

Thermal correction to Enthalpy=	0.400520
Thermal correction to Gibbs Free Energy=	0.335789
Sum of electronic and zero-point Energies=	-3835.723469
Sum of electronic and thermal Energies=	-3835.705172
Sum of electronic and thermal Enthalpies=	-3835.704307
Sum of electronic and thermal Free Energies=	-3835.769038

Number of Imaginary Frequencies = 1

E (Single Point Energy) [IEFPCM$_{(DCM)}$M06-2X/6-311++G(2d,2p)] = -3838.734498

C	0.17093800	1.26588700	0.85145000
H	-0.25600900	0.80089000	1.73689000
C	1.50186300	0.93797400	0.56416500
N	2.01369600	-0.21077500	1.01583700
C	3.28996900	-0.80012800	0.58401900
H	4.13209700	-0.20119200	0.94713300
C	1.39547400	-0.99238500	2.10673700
H	0.53209900	-1.54007900	1.71824200
H	1.06881200	-0.31550800	2.90000300
C	2.51450100	-1.92903400	2.55448800
H	2.12619000	-2.84374900	3.00449000
H	3.15629100	-1.42646100	3.28442500
C	3.28418900	-2.18301500	1.26073900
H	4.29887500	-2.55294600	1.41011000
H	2.73858300	-2.89470500	0.63129600
C	2.34951000	1.72203000	-0.41391400
H	3.40067000	1.63653000	-0.11762600
H	2.25628800	1.26318500	-1.40597400
C	-0.64463500	-0.18085500	-0.54076900
H	-0.72315900	0.61796900	-1.29352700
C	-1.95538100	-0.58359700	0.13259500
O	0.18395700	-1.12613000	-0.68833600

C	3.48348600	-0.97544400	-0.92643400
O	2.43315200	-1.18137600	-1.68112800
O	4.61836800	-0.98724900	-1.36799300
C	-2.90803700	0.48782800	0.62590600
H	-2.39934400	1.02520200	1.43498600
C	-4.22265800	-0.05059300	1.19472900
H	-4.84961500	-0.42835200	0.38230100
H	-4.00727000	-0.90595200	1.84771100
H	-1.74476000	-1.33147200	0.89987200
H	-3.11145700	1.20721900	-0.17704000
C	-4.97209600	1.02822100	1.97311800
H	-5.18041100	1.89384700	1.33557000
H	-5.92694900	0.65234100	2.34959200
H	-4.38496700	1.37610500	2.82914700
H	1.48520600	-1.11003800	-1.23105700
C	1.98711200	3.19725000	-0.52289500
C	-0.39936100	2.63762800	0.55368400
H	2.18684500	3.72707700	0.41339800
H	2.57986200	3.66429500	-1.31094900
H	-1.47977300	2.58842500	0.41625000
H	-0.21846700	3.31997300	1.39253300
S	0.24117200	3.40526100	-0.95985800
Br	-2.87906600	-1.61039800	-1.29248400

(*R*)-**TS2**$_T$-Br-P

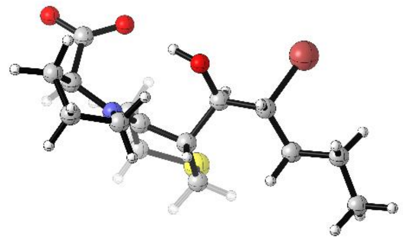

- Thermochemistry -

Zero-point correction=	0.385550 (Hartree/Particle)
Thermal correction to Energy=	0.403892
Thermal correction to Enthalpy=	0.404757
Thermal correction to Gibbs Free Energy=	0.339331
Sum of electronic and zero-point Energies=	-3835.746803
Sum of electronic and thermal Energies=	-3835.728461
Sum of electronic and thermal Enthalpies=	-3835.727596
Sum of electronic and thermal Free Energies=	-3835.793023

Number of Imaginary Frequencies = 0

E (Single Point Energy) [IEFPCM$_{(DCM)}$M06-2X/6-311++G(2d,2p)] = -3838.761015

C	0.02539100	0.89194000	0.67828500
H	-0.07438100	0.55998200	1.71527800
C	1.47782500	0.84062800	0.26472300
N	2.31169000	0.09104300	0.90432500
C	3.65650700	-0.28751500	0.38448100
H	4.28811700	0.60387300	0.36672100
C	2.02802900	-0.62598900	2.17882200
H	1.43698600	-1.51323000	1.93248400
H	1.46700300	0.01956000	2.85215100
C	3.41912300	-0.97438000	2.68983000
H	3.38752200	-1.80166200	3.39997600
H	3.86376600	-0.10541200	3.18442600
C	4.17389000	-1.31289000	1.40663100

H	5.25738900	-1.25167000	1.50527200
H	3.91441500	-2.32338000	1.07420900
C	1.89816900	1.53668800	-1.00337300
H	2.98403800	1.52365300	-1.10582400
H	1.49555700	0.94590600	-1.83590500
C	-0.68206700	-0.18904800	-0.22890200
H	-0.81350500	0.24998600	-1.22734800
C	-2.04571500	-0.64982700	0.30582300
O	0.08496700	-1.36585300	-0.26818000
C	3.59648100	-0.91823400	-1.03915400
O	2.47632600	-1.31587300	-1.45740900
O	4.70185800	-0.99905400	-1.59662800
C	-3.04121900	0.39670500	0.77002300
H	-2.58898300	0.89404100	1.63823900
C	-4.38475000	-0.16931100	1.23252100
H	-4.95245900	-0.52299800	0.36752500
H	-4.20298000	-1.04581000	1.86746600
H	-1.86677700	-1.38726300	1.09259500
H	-3.19800200	1.15331800	-0.00963300
C	-5.19468200	0.87641700	1.99539700
H	-5.36969300	1.76137700	1.37460700
H	-6.16829200	0.48007900	2.29490100
H	-4.66939800	1.19968800	2.89995200
H	0.92562300	-1.28705600	-0.78556700
C	1.40859900	2.98215800	-1.12440500
C	-0.51546800	2.33395600	0.59964200
H	1.84875800	3.61448900	-0.34762200
H	1.71648000	3.37498000	-2.09448100

H	-1.56118700	2.38020000	0.88830400
H	0.03785300	2.95542900	1.31113300
S	-0.39629300	3.09859200	-1.03803700
Br	-2.85785400	-1.70384000	-1.14756800

(*S*)-**TS2**$_{T}$-Br-Pre

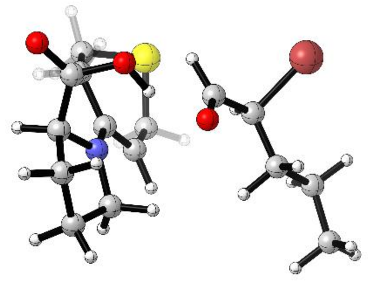

- Thermochemistry -

Zero-point correction= 0.381375 (Hartree/Particle)
Thermal correction to Energy= 0.401323
Thermal correction to Enthalpy= 0.402188
Thermal correction to Gibbs Free Energy= 0.332851
Sum of electronic and zero-point Energies= -3835.739020
Sum of electronic and thermal Energies= -3835.719072
Sum of electronic and thermal Enthalpies= -3835.718207
Sum of electronic and thermal Free Energies= -3835.787543

Number of Imaginary Frequencies = 0

E (Single Point Energy) [IEFPCM$_{(DCM)}$M06-2X/6-311++G(2d,2p)] = -3838.752143

C	-0.46214300	0.46966600	-1.84265900
H	-0.15975600	-0.48910300	-2.25172800
C	-1.52765100	0.52872500	-1.00527700
N	-2.19880500	-0.61893300	-0.60551700
C	-3.44975000	-0.61322200	0.14332400
H	-4.24875300	-0.05494300	-0.36149000
C	-1.98908600	-1.88934400	-1.31174600

H	-1.11212400	-2.40933400	-0.90420200
H	-1.81339500	-1.70343600	-2.37654700
C	-3.28903100	-2.66425800	-1.08930800
H	-3.13295400	-3.74400400	-1.06204000
H	-3.99627600	-2.43829100	-1.89312700
C	-3.81210400	-2.10890200	0.23347500
H	-4.88285300	-2.25123300	0.38502900
H	-3.27739300	-2.56797000	1.07267300
C	-2.01793800	1.81833900	-0.37636600
H	-3.10881100	1.87408300	-0.47473200
H	-1.80284400	1.79846300	0.70004700
C	0.67663700	-0.20166200	0.89473400
H	0.27857200	0.77800900	1.21073400
C	2.00907400	-0.16760000	0.19118000
O	0.09467300	-1.23784500	1.15384700
C	-3.34172900	-0.04953900	1.55622400
O	-2.21249100	-0.28621900	2.22013800
O	-4.26173300	0.53608800	2.08304200
C	2.43892500	-1.50455600	-0.36827600
H	2.50756700	-2.23321900	0.44649900
C	3.73631600	-1.46113500	-1.17054500
H	3.67206300	-0.66368600	-1.92183800
H	4.57011300	-1.20446400	-0.50797100
H	1.61963900	-1.84076400	-1.02050900
C	4.00717800	-2.79795100	-1.85723700
H	4.94469200	-2.77286800	-2.41856600
H	4.07693000	-3.60543800	-1.12119100
H	3.20169100	-3.04884100	-2.55475200

H	-1.53026200	-0.76191500	1.68770300
H	2.02374200	0.64442100	-0.53543000
C	-1.43035100	3.09137300	-0.97378800
C	0.35768400	1.64276700	-2.31122300
H	1.39805200	1.34066000	-2.46606400
H	0.00345200	2.03191700	-3.27350000
H	-1.84158200	3.28108800	-1.97014800
H	-1.67202000	3.94649900	-0.34042800
S	0.37950200	3.01611400	-1.11862700
Br	3.23435200	0.44333300	1.60448400

(*S*)-**TS2**$_{T}$-Br

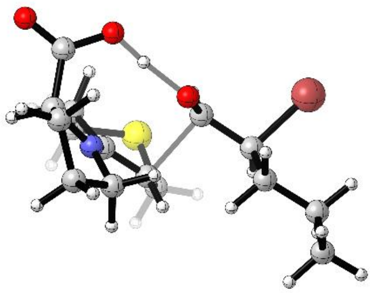

- Thermochemistry -

Zero-point correction=	0.381194 (Hartree/Particle)
Thermal correction to Energy=	0.399570
Thermal correction to Enthalpy=	0.400435
Thermal correction to Gibbs Free Energy=	0.335558
Sum of electronic and zero-point Energies=	-3835.731431
Sum of electronic and thermal Energies=	-3835.713055
Sum of electronic and thermal Enthalpies=	-3835.712190
Sum of electronic and thermal Free Energies=	-3835.777067

Number of Imaginary Frequencies = 1

E (Single Point Energy) [IEFPCM$_{(DCM)}$M06-2X/6-311++G(2d,2p)] = -3838.741723

C	-0.31215300	0.74983500	-1.39252500

H	0.15185900	-0.05779800	-1.95229800
C	-1.57707800	0.49315200	-0.85692600
N	-1.98350200	-0.76872600	-0.67424300
C	-3.14579100	-1.17077600	0.13043700
H	-4.07725300	-0.84370100	-0.34347400
C	-1.36753500	-1.92586900	-1.35685900
H	-0.43497700	-2.19519100	-0.85272400
H	-1.15603300	-1.66503500	-2.39720100
C	-2.42447800	-3.01843500	-1.22106300
H	-1.98903800	-4.01761700	-1.26764900
H	-3.16872500	-2.92422700	-2.01783600
C	-3.05782300	-2.70805500	0.13352300
H	-4.03933100	-3.15909800	0.28196000
H	-2.39449400	-3.03509100	0.94197900
C	-2.44400100	1.58689000	-0.27320200
H	-3.49573100	1.29618800	-0.36520500
H	-2.22821300	1.67269300	0.80000200
C	0.65906800	0.30580400	0.48782400
H	0.42737300	1.31298700	0.86142500
C	2.06670900	0.14109100	-0.06272700
O	0.12095600	-0.70840400	1.01968800
C	-3.16757000	-0.65412800	1.57289000
O	-2.02814000	-0.46603600	2.19031200
O	-4.24386100	-0.49789800	2.12150600
C	2.38231300	-1.25358100	-0.56468500
H	2.15256000	-1.97807800	0.22297900
C	3.81135100	-1.43365900	-1.07382900
H	4.07086400	-0.59626700	-1.73408300

H	4.50672500	-1.39293700	-0.22952100
H	1.68929900	-1.45673800	-1.39359100
C	3.97598400	-2.75566900	-1.82003800
H	5.00656200	-2.89491400	-2.15679600
H	3.71668500	-3.60092800	-1.17405500
H	3.32521200	-2.79431100	-2.69963100
H	-1.13123200	-0.58359700	1.63930900
H	2.33161000	0.91673100	-0.77899200
C	-2.25561800	2.95077900	-0.92796800
C	0.11475400	2.12128700	-1.86596100
H	1.20328200	2.21665600	-1.85080400
H	-0.19512500	2.28018300	-2.90536800
H	-2.58358700	2.93555200	-1.97176300
H	-2.84787800	3.69820800	-0.39764800
S	-0.52530500	3.49084700	-0.85930100
Br	3.23167200	0.54858700	1.48868900

(*S*)-**TS2**$_T$-Br-P

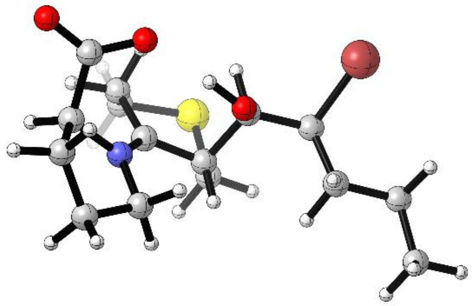

- Thermochemistry -

Zero-point correction= 0.385466 (Hartree/Particle)
Thermal correction to Energy= 0.403988
Thermal correction to Enthalpy= 0.404854
Thermal correction to Gibbs Free Energy= 0.339318

Sum of electronic and zero-point Energies= -3835.755023
Sum of electronic and thermal Energies= -3835.736500
Sum of electronic and thermal Enthalpies= -3835.735635
Sum of electronic and thermal Free Energies= -3835.801170

Number of Imaginary Frequencies = 0

E (Single Point Energy) [IEFPCM$_{(DCM)}$M06-2X/6-311++G(2d,2p)] = -3838.768722

C	-0.08690200	0.56808000	-1.03361100
H	0.06075600	-0.24281800	-1.74847100
C	-1.53000100	0.61962100	-0.61620900
N	-2.28847400	-0.41070400	-0.79140500
C	-3.55278500	-0.63133000	-0.04089600
H	-4.29017300	0.11432500	-0.34689800
C	-1.96333600	-1.59873700	-1.63023600
H	-1.26889000	-2.22981200	-1.06827200
H	-1.50091700	-1.27443200	-2.56148400
C	-3.32456100	-2.25581800	-1.81647700
H	-3.22609000	-3.30736000	-2.08903100
H	-3.88426000	-1.74082800	-2.60316400
C	-3.98684600	-2.04746200	-0.45612900
H	-5.07297300	-2.13251900	-0.47939400
H	-3.59918500	-2.77490600	0.26469600
C	-2.01728100	1.81037100	0.16384100
H	-3.10504700	1.78860600	0.25235800
H	-1.61545600	1.72516800	1.18035600
C	0.65827100	0.13589400	0.28495200
H	0.44916000	0.87761600	1.06519800
C	2.17112500	0.10748100	0.07284300
O	0.21738200	-1.13740700	0.66832700
C	-3.32228900	-0.55575900	1.49937400

O	-2.13633400	-0.66718000	1.91266100
O	-4.36444900	-0.43933800	2.16169300
C	2.64625700	-0.92473900	-0.93501600
H	2.29236200	-1.91510800	-0.62924700
C	4.15575600	-0.93291400	-1.17210400
H	4.50662500	0.09964500	-1.29730100
H	4.66243500	-1.32880900	-0.28659700
H	2.15692700	-0.69097700	-1.89227900
C	4.52807800	-1.76295400	-2.39857800
H	5.61115700	-1.79046300	-2.54370400
H	4.17771100	-2.79469400	-2.28985700
H	4.07626900	-1.34949100	-3.30613700
H	-0.62782000	-1.04497400	1.17193700
H	2.54375700	1.10664700	-0.15848300
C	-1.59625900	3.15058300	-0.45147000
C	0.40840400	1.86683000	-1.68026900
H	1.46802300	1.78479800	-1.93013100
H	-0.12890300	2.03665000	-2.61845600
H	-2.05899600	3.29298900	-1.43269300
H	-1.93540800	3.95761300	0.19959600
S	0.20380800	3.32302600	-0.61594100
Br	2.97739300	-0.28010900	1.82459400

Supplementary Table 10. Energies for enamine addition to 2-chloropentanal. Reported energies for structures optimized at the IEFPCM$_{(DCM)}$M06-2X/6-311++G(2d,2p)//IEFPCM$_{(DCM)}$M06-2X/6-31+G(d,p) level of theory represent the sum of the thermal correction to Gibbs Free Energy computed at the IEFPCM$_{(DCM)}$M06-2X/6-31+G(d,p) level of theory and single point energies computed at the IEFPCM$_{(DCM)}$M06-2X/6-311++G(2d,2p). All energies are reported in Hartrees.

**Structure**	**Single Point Energies, E IEFPCM$_{(DCM)}$M06-2X/6-311++G(2d,2p)**	**Thermal Corrections to Gibbs Free Energies, IEFPCM$_{(DCM)}$M06-2X/6-31+G(d,p)**	**Gibbs Free Energies (G), IEFPCM$_{(DCM)}$M06-2X/6-31+G(d,p)**	**Gibbs Free Energies (G), IEFPCM$_{(DCM)}$M06-2X/6-311++G(2d,2p)// IEFPCM$_{(DCM)}$M06-2X/6-31+G(d,p)**
2-Chloropentanal	-731.33393130	0.102879	-731.121809	-731.2310523
Enamine of Cyclohexanone (G)	-634.55147535	0.238392	-634.142903	-634.3130834
Enamine of Dioxane (O)	-784.98483791	0.244077	-784.522619	-784.7407609
Enamine of Tetrahydro-4H-thiopyranone (T)	-993.43210840	0.210205	-993.028735	-993.2219034
Enamine of Tetrahydro-4H-pyranone (P)	-670.45469021	0.214793	-670.055312	-670.2398972
(*R*)-**TS3**$_{P}$-Cl-Pre	-1401.805997	0.337891	-1401.17504	-1401.468106
(*R*)-**TS3**$_{P}$-Cl	-1401.792111	0.341323	-1401.159118	-1401.450788
(*R*)-**TS3**$_{P}$-Cl-P	-1401.825385	0.345243	-1401.188706	-1401.480142
(*S*)-**TS3**$_{P}$-Cl-Pre	-1401.805033	0.336807	-1401.175179	-1401.468226
(*S*)-**TS3**$_{P}$-Cl	-1401.791658	0.340366	-1401.159908	-1401.451292
(*S*)-**TS3**$_{P}$-Cl-P	-1401.82164	0.344016	-1401.186409	-1401.477624
(*R*)-**TS3**$_{G}$-Cl-Pre	-1365.903524	0.361325	-1365.263362	-1365.542199
(*R*)-**TS3**$_{G}$-Cl	-1365.888196	0.364421	-1365.246524	-1365.523775
(*R*)-**TS3**$_{G}$-Cl-P	-1365.918739	0.36881	-1365.27305	-1365.549929
(*S*)-**TS3**$_{G}$-Cl-Pre	-1365.901816	0.360176	-1365.262948	-1365.54164
(*S*)-**TS3**$_{G}$-Cl	-1365.88909	0.364491	-1365.247432	-1365.524599
(*S*)-**TS3**$_{G}$-Cl-P	-1365.9162	0.367159	-1365.27225	-1365.549041
(*R*)-**TS3**$_{O}$-Cl-Pre	-1516.336463	0.365498	-1515.644377	-1515.970965
(*R*)-**TS3**$_{O}$-Cl	-1516.319628	0.36887	-1515.625316	-1515.95075

(*R*)-**TS3**O-Cl-P	-1516.34889	0.373824	-1515.649894	-1515.975066
(*S*)-**TS3**O-Cl-Pre	-1516.33738	0.366707	-1515.643925	-1515.970673
(*S*)-**TS3**O-Cl	-1516.318913	0.369175	-1515.624654	-1515.949738
(*S*)-**TS3**O-Cl-P	-1516.346594	0.373427	-1515.648241	-1515.973167
(*R*)-**TS3**T-Cl-Pre	-1724.78465	0.335088	-1724.147822	-1724.449562
(*R*)-**TS3**T-Cl	-1724.769951	0.336882	-1724.132824	-1724.433069
(*R*)-**TS3**T-Cl-P	-1724.802661	0.341118	-1724.161485	-1724.461543
(*S*)-**TS3**T-Cl-Pre	-1724.781967	0.333162	-1724.146451	-1724.448805
(*S*)-**TS3**T-Cl	-1724.769924	0.336822	-1724.132677	-1724.433102
(*S*)-**TS3**T-Cl-P	-1724.799615	0.341308	-1724.158295	-1724.458307

(*R*)-**TS3**P-Cl-Pre

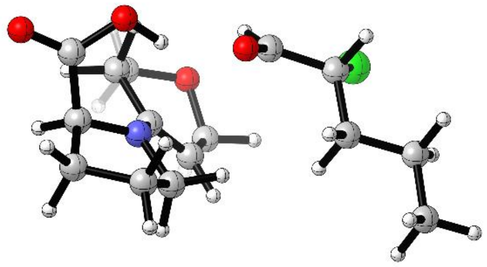

- Thermochemistry -

Zero-point correction= 0.385367 (Hartree/Particle)
Thermal correction to Energy= 0.404688
Thermal correction to Enthalpy= 0.405553
Thermal correction to Gibbs Free Energy= 0.337891
Sum of electronic and zero-point Energies= -1401.127565
Sum of electronic and thermal Energies= -1401.108243
Sum of electronic and thermal Enthalpies= -1401.107378
Sum of electronic and thermal Free Energies= -1401.175040

Number of Imaginary Frequencies = 0

E (Single Point Energy) [IEFPCM$_{(DCM)}$M06-2X/6-311++G(2d,2p)] = -1401.805997

C	0.11697200	1.00008100	-1.53762800
H	0.55220700	0.17987700	-2.09967300
C	-1.01985300	0.83922000	-0.83013700
N	-1.62363600	-0.39870500	-0.63107600
C	-3.06370200	-0.53475000	-0.42048200
H	-3.64646800	0.21827700	-0.96040700
C	-1.06121200	-1.59546700	-1.25501200
H	-1.01408400	-1.48522100	-2.35011800
H	-0.04670400	-1.76526400	-0.88092600
C	-2.04982100	-2.68513900	-0.85729900
H	-1.99910700	-3.55752700	-1.51090700
H	-1.85557300	-3.01000200	0.17067100
C	-3.39002400	-1.95084100	-0.95057800
H	-3.69942500	-1.86937500	-1.99621900
H	-4.19992700	-2.42173500	-0.39064400
C	-1.62299100	2.00770900	-0.08460600
H	-2.70490200	2.06408800	-0.24848200
H	-1.47415600	1.86789000	0.99543700
C	1.10069200	0.23014500	1.38805000
H	0.84676800	1.30354600	1.34076400
C	2.55788600	-0.15380800	1.18763000
O	0.27144700	-0.62183100	1.62793100
C	-3.46654500	-0.41700400	1.05197800
O	-2.49963700	-0.50961800	1.95874700
O	-4.62537500	-0.28372300	1.38167100
H	-1.61150600	-0.56539600	1.54054300
C	2.73354100	-1.23905900	0.13027000

H	2.42424400	-0.83921800	-0.84376100
C	4.15030400	-1.80149200	0.04965600
H	2.03152900	-2.03972800	0.39524400
C	4.24746100	-2.92806300	-0.97706300
H	5.26459700	-3.32412700	-1.03250300
H	3.57709900	-3.75369700	-0.71751800
H	3.97041400	-2.57098000	-1.97421200
H	4.44604400	-2.17104400	1.03923800
H	4.84900500	-0.99982600	-0.21219300
H	2.93614700	-0.50389800	2.15418000
C	-0.98615900	3.31713300	-0.53608300
C	0.84047100	2.32149200	-1.59127500
H	-1.35007500	3.59051300	-1.53797400
H	-1.22899300	4.12642500	0.15467600
H	1.91562900	2.17572600	-1.45336400
H	0.69267900	2.81154100	-2.56828700
O	0.42553200	3.20113700	-0.55954400
Cl	3.50542100	1.32831900	0.79582400

(*R*)-**TS3**$_{P}$-Cl

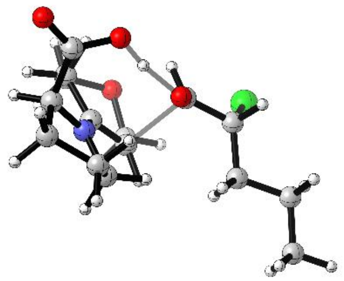

- Thermochemistry -

Zero-point correction= 0.385433 (Hartree/Particle)

Thermal correction to Energy= 0.403070
Thermal correction to Enthalpy= 0.403935
Thermal correction to Gibbs Free Energy= 0.341323
Sum of electronic and zero-point Energies= -1401.115009
Sum of electronic and thermal Energies= -1401.097372
Sum of electronic and thermal Enthalpies= -1401.096507
Sum of electronic and thermal Free Energies= -1401.159118

Number of Imaginary Frequencies = 1

E (Single Point Energy) [IEFPCM$_{(DCM)}$M06-2X/6-311++G(2d,2p)] = -1401.792111

C	-0.39198300	0.70901100	1.11490500
H	-0.80661900	-0.16339600	1.60844900
C	0.97656200	0.72845400	0.85844900
N	1.69718300	-0.39663400	0.79751800
C	3.09820600	-0.46514100	0.37324000
H	3.70559900	0.28500400	0.88415600
C	1.19465800	-1.70723000	1.25582900
H	1.16083000	-1.71319100	2.35299300
H	0.19678800	-1.88907800	0.86022000
C	2.22800400	-2.68590100	0.70908700
H	2.26308900	-3.61219500	1.28423100
H	1.98712900	-2.93104700	-0.33074100
C	3.52973900	-1.88625200	0.78528500
H	3.89842600	-1.85956600	1.81458100
H	4.32580400	-2.26656100	0.14378300
C	1.61638700	2.02004700	0.41133200
H	2.64608800	2.09234200	0.77120000
H	1.65882400	2.02487000	-0.68743400
C	-0.79471900	0.17720300	-0.97576100
H	-0.55884400	1.20579500	-1.28541500
C	-2.28714500	-0.17370400	-0.99105700

O	-0.02461500	-0.77814600	-1.27985500
C	3.32183500	-0.24110300	-1.13233000
O	2.30447300	-0.31919000	-1.95441000
O	4.45552100	-0.02320500	-1.52023300
H	1.34588500	-0.51064600	-1.57017300
C	-2.79552500	-1.08927200	0.11115800
H	-2.88175400	-0.52735700	1.04839000
C	-4.13093100	-1.75475400	-0.21609400
H	-2.02778800	-1.86198100	0.25529700
C	-4.59402700	-2.67759600	0.90901100
H	-5.54825100	-3.15112500	0.66433800
H	-3.86128900	-3.47035300	1.09172600
H	-4.72483700	-2.11960200	1.84187800
H	-4.02918400	-2.32422400	-1.14788100
H	-4.88676700	-0.98226500	-0.39686000
H	-2.42372300	-0.66945900	-1.95786800
C	0.82486700	3.23083700	0.90343500
C	-1.09574700	2.01771100	1.40527100
H	0.98188800	3.36951900	1.98341300
H	1.15415600	4.13342200	0.38672400
H	-2.15576900	1.95784100	1.15711300
H	-1.01617400	2.26373000	2.47711900
O	-0.55685100	3.08067800	0.64092900
Cl	-3.28900500	1.32947100	-1.12081700

(*R*)-**TS3**$_{P}$-Cl-P

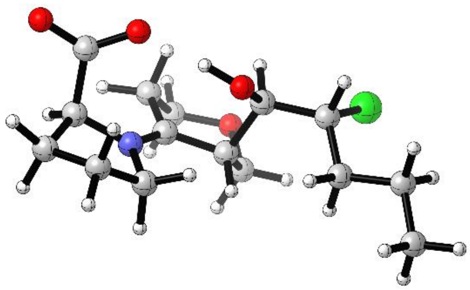

- Thermochemistry -

Zero-point correction=	0.389582 (Hartree/Particle)
Thermal correction to Energy=	0.407307
Thermal correction to Enthalpy=	0.408172
Thermal correction to Gibbs Free Energy=	0.345243
Sum of electronic and zero-point Energies=	-1401.144367
Sum of electronic and thermal Energies=	-1401.126642
Sum of electronic and thermal Enthalpies=	-1401.125777
Sum of electronic and thermal Free Energies=	-1401.188706

Number of Imaginary Frequencies = 0

E (Single Point Energy) [IEFPCM$_{(DCM)}$M06-2X/6-311++G(2d,2p)] = -1401.825385

C	0.44187200	0.49188700	-0.71616800
H	0.67756600	-0.40106700	-1.29874100
C	-1.04576400	0.67395200	-0.61359100
N	-1.85767500	-0.31518700	-0.76756900
C	-3.28386100	-0.27426800	-0.37519700
H	-3.77432200	0.59768300	-0.80844300
C	-1.46141100	-1.70420800	-1.15167500
H	-1.30064200	-1.70809700	-2.23392600
H	-0.54763000	-1.98114400	-0.63034700
C	-2.66720400	-2.54922000	-0.74939900
H	-2.74660800	-3.44548400	-1.36559800

H	-2.57340900	-2.85406000	0.29740100
C	-3.84377400	-1.58776500	-0.91991900
H	-4.10236300	-1.47849900	-1.97776000
H	-4.73616400	-1.88081400	-0.36706000
C	-1.51039500	2.03582400	-0.19376100
H	-2.57843000	2.17810600	-0.35706000
H	-1.33594200	2.11018700	0.88697800
C	0.92860100	0.26911300	0.75652500
H	0.75908600	1.19043600	1.32817700
C	2.41524900	-0.10418500	0.88945000
O	0.23541600	-0.81731700	1.32654300
C	-3.39898900	-0.18411700	1.19064400
O	-2.32871700	-0.13731500	1.85442500
O	-4.56724500	-0.16392200	1.60410800
H	-0.69329100	-0.56298400	1.55518200
C	2.91306400	-1.13566900	-0.11031000
H	2.96063000	-0.69590600	-1.11516400
C	4.26891000	-1.74082300	0.24874300
H	2.15696200	-1.93340000	-0.13724900
C	4.69786900	-2.80869800	-0.75512900
H	5.66866300	-3.23446900	-0.48874700
H	3.97031700	-3.62598300	-0.79282800
H	4.78094400	-2.38640700	-1.76193400
H	4.21070400	-2.17573900	1.25395600
H	5.02227400	-0.94666700	0.28999500
H	2.52601700	-0.48798700	1.90579100
C	-0.71420200	3.13266500	-0.91578500
C	1.05748300	1.71144300	-1.42334700

H	-0.99632000	3.17169200	-1.97774900
H	-0.93158700	4.09946300	-0.46127200
H	2.14525400	1.66103700	-1.39871800
H	0.73492900	1.71690500	-2.47496400
O	0.67706400	2.91724800	-0.79687100
Cl	3.45825000	1.38437500	0.86064300

(*S*)-**TS3**$_{P}$-Cl-Pre

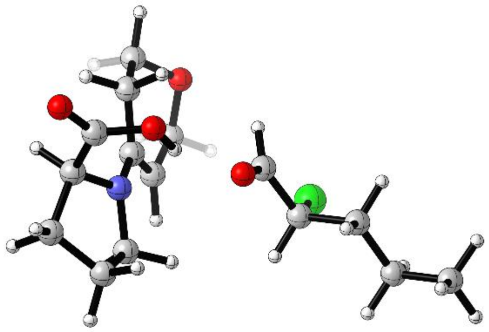

- Thermochemistry -

Zero-point correction=	0.385343 (Hartree/Particle)
Thermal correction to Energy=	0.404761
Thermal correction to Enthalpy=	0.405626
Thermal correction to Gibbs Free Energy=	0.336807
Sum of electronic and zero-point Energies=	-1401.126642
Sum of electronic and thermal Energies=	-1401.107224
Sum of electronic and thermal Enthalpies=	-1401.106359
Sum of electronic and thermal Free Energies=	-1401.175179

Number of Imaginary Frequencies = 0

E (Single Point Energy) [IEFPCM$_{(DCM)}$M06-2X/6-311++G(2d,2p)] = -1401.805033

C	0.52086100	1.70660200	1.15997200
H	0.10004200	1.28543700	2.06805200
C	1.42673200	1.03145600	0.42319600
N	1.78312400	-0.29134000	0.67330500

C	3.11346000	-0.80435800	0.34508000
H	3.89528600	-0.04330900	0.43285400
C	1.21643200	-0.99259200	1.82446200
H	1.43159200	-0.45333800	2.76052900
H	0.13027400	-1.07088700	1.71409500
C	1.93068200	-2.33829100	1.79164400
H	1.90358500	-2.85184100	2.75411200
H	1.47139200	-2.98759200	1.03840500
C	3.34734100	-1.93673800	1.37273900
H	3.88745300	-1.52500500	2.22967200
H	3.94057800	-2.75237100	0.95564300
C	1.99364300	1.64984400	-0.83450800
H	3.07579400	1.49281200	-0.90298300
H	1.54372000	1.15845800	-1.70864600
C	-1.26593100	0.00474100	-0.74890100
H	-1.07108900	0.99484300	-1.19730400
C	-2.52363100	-0.16326700	0.08578600
O	-0.54481600	-0.94963200	-0.94917000
C	3.20863700	-1.35537800	-1.08012800
O	2.06957100	-1.56664900	-1.73073200
O	4.27817100	-1.62294900	-1.58383200
H	1.29570100	-1.25844500	-1.21025700
C	-3.53834300	-1.03751100	-0.65339500
H	-3.00999400	-1.96171800	-0.91820200
C	-4.77913500	-1.37055800	0.17152600
H	-3.82420400	-0.54388900	-1.59063800
C	-5.72022700	-2.30529200	-0.58590600
H	-6.60714100	-2.53673400	0.00942000

H	-6.05338000	-1.84684400	-1.52245800
H	-5.22244300	-3.24858200	-0.83200900
H	-5.30554100	-0.44644700	0.43194600
H	-4.46773400	-1.83676600	1.11448300
H	-2.25265300	-0.62022900	1.04167000
Cl	-3.20460100	1.45907900	0.47088500
C	1.70782000	3.14601300	-0.87400000
C	0.06011100	3.09051900	0.77653200
H	2.34716200	3.67418600	-0.15044200
H	1.89928000	3.55244400	-1.86867200
H	-1.02331500	3.17822300	0.88732300
H	0.52560300	3.84885500	1.42840600
O	0.34653200	3.39956000	-0.57723700

(*S*)-**TS3**$_{P}$-Cl

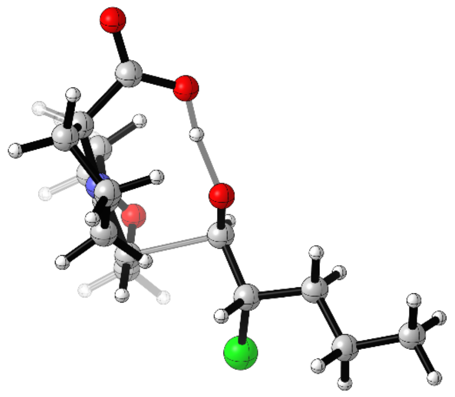

- Thermochemistry -

Zero-point correction=	0.385246 (Hartree/Particle)
Thermal correction to Energy=	0.403024
Thermal correction to Enthalpy=	0.403889
Thermal correction to Gibbs Free Energy=	0.340366
Sum of electronic and zero-point Energies=	-1401.115028
Sum of electronic and thermal Energies=	-1401.097250
Sum of electronic and thermal Enthalpies=	-1401.096385

Sum of electronic and thermal Free Energies= -1401.159908

Number of Imaginary Frequencies = 1

E (Single Point Energy) [$IEFPCM_{(DCM)}$M06-2X/6-311++G(2d,2p)] = -1401.791658

C	0.08379200	1.49870100	0.82749200
H	-0.36762800	1.12990200	1.74521600
C	1.33215400	0.99929600	0.45191800
N	1.74772300	-0.21384500	0.82498600
C	2.99471500	-0.84422200	0.37784500
H	3.84246500	-0.16495800	0.49155000
C	1.05900400	-1.02612800	1.84490300
H	1.24778100	-0.59442200	2.83621500
H	-0.01201400	-1.03788000	1.65344100
C	1.70014400	-2.39946400	1.68614000
H	1.62289900	-2.99909400	2.59412600
H	1.21109800	-2.93832000	0.86786600
C	3.14432400	-2.05667900	1.31800800
H	3.69847200	-1.74747300	2.20867200
H	3.68914000	-2.87076800	0.83856600
C	2.14219000	1.73036200	-0.59079800
H	3.21303700	1.60914100	-0.40715900
H	1.92689700	1.28644100	-1.57240700
C	-1.00032200	0.21838300	-0.54006600
H	-1.02415100	1.05330500	-1.25763600
C	-2.31242800	-0.07968900	0.19560900
O	-0.32558000	-0.82441900	-0.77707700
C	3.00812400	-1.28675400	-1.09644600
O	1.88771800	-1.33002500	-1.77605200
O	4.07445300	-1.60834000	-1.58724700

H	1.01131900	-1.04486200	-1.30205400
C	-3.18892200	-0.97523300	-0.68231900
H	-2.56793200	-1.83542600	-0.95534600
C	-4.46737000	-1.46140000	-0.00270500
H	-3.42992500	-0.43983000	-1.61018000
C	-5.23391100	-2.44163900	-0.88908500
H	-6.15060200	-2.78215900	-0.40038100
H	-5.51293500	-1.97150600	-1.83766000
H	-4.62535600	-3.32229900	-1.11804800
H	-5.10701100	-0.60731300	0.24160600
H	-4.20639800	-1.94480100	0.94744900
H	-2.09777100	-0.58093400	1.14257200
Cl	-3.22570000	1.41794800	0.63424700
C	1.79138300	3.21217000	-0.61872800
C	-0.24113300	2.94348800	0.48926200
H	2.17496900	3.71452800	0.28183000
H	2.22688700	3.69130800	-1.49683100
H	-1.30970000	3.07388800	0.32650800
H	0.05591700	3.60129300	1.32250000
O	0.39127500	3.37864600	-0.70008400

(*S*)-**TS3**$_{P}$-Cl-P

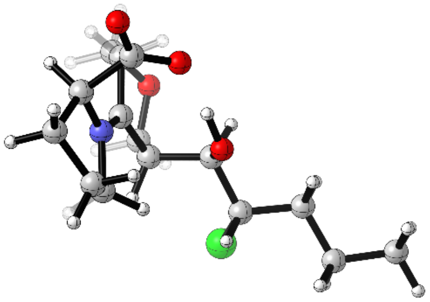

- Thermochemistry -

Zero-point correction=	0.389100 (Hartree/Particle)
Thermal correction to Energy=	0.406954
Thermal correction to Enthalpy=	0.407819
Thermal correction to Gibbs Free Energy=	0.344016
Sum of electronic and zero-point Energies=	-1401.141324
Sum of electronic and thermal Energies=	-1401.123470
Sum of electronic and thermal Enthalpies=	-1401.122605
Sum of electronic and thermal Free Energies=	-1401.186409

Number of Imaginary Frequencies = 0

E (Single Point Energy) [IEFPCM$_{(DCM)}$M06-2X/6-311++G(2d,2p)] = -1401.821640

C	-0.18747100	1.14499500	0.58691700
H	-0.33681100	0.85126500	1.63022400
C	1.25038300	0.93151800	0.19413800
N	1.95112500	0.00002500	0.74545300
C	3.28182700	-0.43683600	0.26262700
H	3.94424000	0.42397700	0.16364200
C	1.46358000	-0.90571400	1.82752100
H	1.51687600	-0.35188200	2.76988400
H	0.43799800	-1.20199200	1.61785000
C	2.44915300	-2.06973800	1.79265900
H	2.52125000	-2.55876600	2.76471200
H	2.12325300	-2.80843400	1.05380300

C	3.75254400	-1.40777300	1.34550900
H	4.20793500	-0.85761900	2.17481000
H	4.48505300	-2.10458500	0.93879300
C	1.77986400	1.74022300	-0.95160200
H	2.86851200	1.72111000	-1.00185000
H	1.40716700	1.25667700	-1.86435200
C	-1.06445300	0.22476200	-0.33995100
H	-1.29877200	0.80142300	-1.24803700
C	-2.37098000	-0.26928500	0.31584300
O	-0.43365100	-0.99516700	-0.65202900
C	3.16325000	-1.12459500	-1.14624500
O	2.02890300	-1.15458700	-1.69251500
O	4.24064200	-1.56065200	-1.57771400
H	0.43849900	-0.92058600	-1.11337600
C	-3.31053700	-0.88084100	-0.71496700
H	-2.70161100	-1.55382700	-1.32910100
C	-4.48139900	-1.65774700	-0.11674000
H	-3.67990200	-0.08277000	-1.37216200
C	-5.34339900	-2.30141800	-1.20123100
H	-6.17897100	-2.85590500	-0.76598900
H	-5.75692300	-1.54098600	-1.87167600
H	-4.75492200	-2.99767800	-1.80733900
H	-5.09656300	-0.98881300	0.49466300
H	-4.09086200	-2.43259100	0.55495600
H	-2.08966100	-1.01129400	1.06908600
Cl	-3.25475300	1.01057600	1.25247400
C	1.26338800	3.17845800	-0.90987900
C	-0.54345100	2.64269500	0.45555400

H	1.71763500	3.73443200	-0.07741600
H	1.50986200	3.68423200	-1.84381100
H	-1.61919900	2.78147200	0.52270400
H	-0.06730300	3.19649000	1.27780300
O	-0.14185100	3.18346200	-0.78386100

(*R*)-**TS3**$_{G}$-Cl-Pre

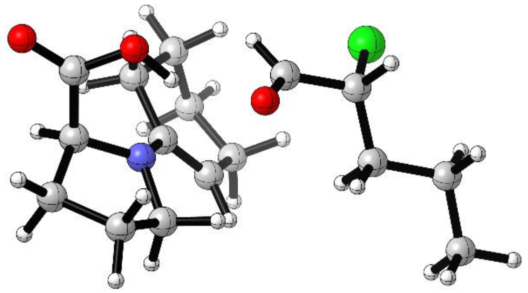

- Thermochemistry -

Zero-point correction=	0.408971 (Hartree/Particle)
Thermal correction to Energy=	0.428531
Thermal correction to Enthalpy=	0.429396
Thermal correction to Gibbs Free Energy=	0.361325
Sum of electronic and zero-point Energies=	-1365.215716
Sum of electronic and thermal Energies=	-1365.196156
Sum of electronic and thermal Enthalpies=	-1365.195291
Sum of electronic and thermal Free Energies=	-1365.263362

Number of Imaginary Frequencies = 0

E (Single Point Energy) [IEFPCM$_{(DCM)}$M06-2X/6-311++G(2d,2p)] = -1365.903524

C	0.25195000	0.84140400	-1.56047800
H	0.54593900	-0.06218300	-2.08649300
C	-0.87987600	0.84207800	-0.82356200
N	-1.67454800	-0.29907700	-0.66910400
C	-3.10373600	-0.19498500	-0.37779700
H	-3.55138800	0.72985500	-0.75247100

C	-1.37924000	-1.49909900	-1.45264000
H	-0.41427400	-1.91774900	-1.14916400
H	-1.33083600	-1.25941400	-2.52713500
C	-2.55843200	-2.41572600	-1.14713900
H	-2.41070600	-2.91258700	-0.18177400
H	-2.69949700	-3.18375700	-1.90952000
C	-3.72443200	-1.42637400	-1.07680500
H	-4.03042400	-1.13669500	-2.08597800
H	-4.60210700	-1.80271000	-0.54771900
C	-1.33133800	2.06649300	-0.05380000
H	-2.20069500	2.51346200	-0.55421300
H	-1.67487300	1.76537000	0.94489200
C	0.45330000	3.33838100	-1.27857700
C	0.86347900	-0.46617400	1.42613700
H	0.42451300	0.51917900	1.66585600
C	2.37365200	-0.54591000	1.29226900
O	0.17669500	-1.45951000	1.32296300
C	-3.40492100	-0.24288800	1.12197800
O	-2.45349300	-0.73259100	1.91315900
O	-4.47411300	0.10858400	1.57184200
C	2.81579300	-1.21090100	-0.00771200
H	2.22595600	-2.13180500	-0.09978900
C	4.30571700	-1.54053300	-0.05046700
H	4.88737300	-0.61863500	0.05811400
H	4.55783300	-2.17885700	0.80525500
H	2.54176200	-0.56202600	-0.84756900
C	4.68719500	-2.24120600	-1.35278500
H	5.75507700	-2.47268300	-1.37776800

H	4.13540900	-3.17953000	-1.46914900
H	4.45952400	-1.60806300	-2.21639000
H	-1.64097700	-0.95589500	1.40444700
H	2.74052300	-1.11686200	2.15217500
Cl	3.06220900	1.11009800	1.47173800
C	-0.22263700	3.11249300	0.07175800
H	-0.64320600	4.04306000	0.46438100
H	0.53063700	2.76828300	0.79309000
C	1.13593700	2.05115400	-1.74168900
H	2.07690200	1.91755300	-1.18765200
H	1.41755800	2.13388800	-2.79801300
H	-0.30539800	3.63746900	-2.01394700
H	1.18248300	4.15211700	-1.21514000

(*R*)-**TS3**$_{G}$-Cl

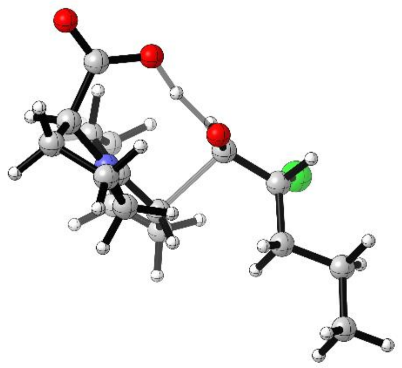

- Thermochemistry -

Zero-point correction= 0.409042 (Hartree/Particle)
Thermal correction to Energy= 0.426929
Thermal correction to Enthalpy= 0.427794
Thermal correction to Gibbs Free Energy= 0.364421
Sum of electronic and zero-point Energies= -1365.201903
Sum of electronic and thermal Energies= -1365.184016
Sum of electronic and thermal Enthalpies= -1365.183151
Sum of electronic and thermal Free Energies= -1365.246524

Number of Imaginary Frequencies = 1

E (Single Point Energy) [IEFPCM$_{(DCM)}$M06-2X/6-311++G(2d,2p)] = -1365.888196

C	0.43112200	0.65116100	-1.05208900
H	0.75151500	-0.28853100	-1.48791000
C	-0.93082400	0.76950500	-0.78013100
N	-1.72814600	-0.30787400	-0.75732100
C	-3.12899700	-0.28517900	-0.33455100
H	-3.64153300	0.60449700	-0.70722900
C	-1.34521900	-1.62074400	-1.31960700
H	-0.40602700	-1.96376600	-0.88834300
H	-1.23297100	-1.51893600	-2.40693700
C	-2.51925700	-2.52214200	-0.94621900
H	-2.36877900	-2.92869700	0.05962100
H	-2.63221900	-3.35624700	-1.64020400
C	-3.71118200	-1.56363200	-0.96043600
H	-4.01479000	-1.34885500	-1.98916300
H	-4.58066200	-1.92205600	-0.40820100
C	-1.52354600	2.08764800	-0.34084400
H	-2.32657000	2.34654700	-1.04315600
H	-1.99964900	1.96446000	0.64071100
C	0.41789400	3.14223400	-1.51850000
C	0.77819800	0.03877400	1.00622700
H	0.53877200	1.03649200	1.40272900
C	2.27454600	-0.31102700	1.02968000
O	0.00588300	-0.93668900	1.24122900
C	-3.32998700	-0.28413800	1.19232200
O	-2.30056200	-0.48112100	1.97753300

O	-4.45510500	-0.10903500	1.62609000
C	2.83920000	-1.06963700	-0.16054500
H	2.09686700	-1.83721000	-0.42100800
C	4.18159400	-1.74043000	0.12413100
H	4.91603500	-0.97856500	0.40818800
H	4.07251500	-2.41256600	0.98402400
H	2.93302800	-0.39473600	-1.01948900
C	4.68987200	-2.52256600	-1.08507600
H	5.65029900	-2.99915200	-0.87256500
H	3.98052600	-3.30608000	-1.37096900
H	4.82558900	-1.86180800	-1.94743600
H	-1.35000600	-0.65767200	1.55746400
H	2.37701500	-0.93004500	1.92652400
Cl	3.26010600	1.16493400	1.40362200
C	-0.49598900	3.22107400	-0.29949200
H	-1.02548000	4.17652900	-0.25495000
H	0.11121800	3.15114300	0.61200700
C	1.25864100	1.86687300	-1.44036000
H	2.07473700	2.02589900	-0.72852900
H	1.73362100	1.66775700	-2.40775100
H	-0.19565600	3.13220800	-2.42910600
H	1.06967900	4.01875100	-1.57452100

(*R*)-**TS3**$_G$-Cl-P

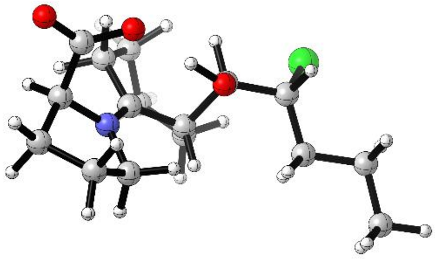

- Thermochemistry -

Zero-point correction=	0.413385 (Hartree/Particle)
Thermal correction to Energy=	0.431387
Thermal correction to Enthalpy=	0.432252
Thermal correction to Gibbs Free Energy=	0.368810
Sum of electronic and zero-point Energies=	-1365.228474
Sum of electronic and thermal Energies=	-1365.210473
Sum of electronic and thermal Enthalpies=	-1365.209608
Sum of electronic and thermal Free Energies=	-1365.273050

Number of Imaginary Frequencies = 0

E (Single Point Energy) [IEFPCM$_{(DCM)}$M06-2X/6-311++G(2d,2p)] = -1365.918739

C	0.45005100	0.39608100	-0.75346000
H	0.60032000	-0.56048200	-1.25512500
C	-1.01647700	0.67621400	-0.61874800
N	-1.89349500	-0.26271500	-0.74709200
C	-3.29718100	-0.12766800	-0.31311500
H	-3.70545000	0.83217200	-0.63186700
C	-1.61825200	-1.66395700	-1.18920500
H	-0.76092100	-2.05572400	-0.64560100
H	-1.40418200	-1.63112000	-2.26144400
C	-2.91863900	-2.40791500	-0.88564500
H	-2.88394300	-2.81912900	0.12766000
H	-3.07789700	-3.22915100	-1.58539200
C	-3.98789900	-1.31747300	-0.97602900

H	-4.21791100	-1.08537900	-2.02060600
H	-4.91327000	-1.56465700	-0.45590900
C	-1.44025200	2.05257400	-0.18025100
H	-2.28931900	2.35726100	-0.80298600
H	-1.81603700	1.97751900	0.84818500
C	0.43139700	2.83653700	-1.62113100
C	0.89653200	0.16043300	0.73951700
H	0.67817900	1.05300400	1.33986800
C	2.38831900	-0.17995500	0.92061300
O	0.21154900	-0.95840500	1.25067900
C	-3.37987600	-0.19870400	1.25601500
O	-2.29796800	-0.29386100	1.89685300
O	-4.53530700	-0.14564100	1.70031300
C	2.97050600	-1.10394900	-0.13604100
H	2.25454900	-1.93103200	-0.24710300
C	4.34159800	-1.67349500	0.22261500
H	5.05883000	-0.85343500	0.33587200
H	4.27714400	-2.17503600	1.19601200
H	3.02246400	-0.58688100	-1.10287900
C	4.84218000	-2.65501500	-0.83494900
H	5.82457200	-3.05456300	-0.57028900
H	4.15308400	-3.49881300	-0.94372000
H	4.92993700	-2.16533200	-1.81035700
H	-0.71541900	-0.71327700	1.50596400
H	2.45528700	-0.65133600	1.90312300
Cl	3.38746800	1.33204800	1.09758000
C	-0.32207500	3.09103900	-0.31938100
H	-0.77127000	4.08689200	-0.30614600

H	0.37122700	3.04534700	0.52795100
C	1.19090800	1.50448000	-1.54816000
H	2.16668500	1.67513200	-1.09318800
H	1.37757700	1.12885100	-2.55805300
H	-0.29183100	2.81520200	-2.44700800
H	1.13387900	3.64604000	-1.83481800

(*S*)-**TS3**$_G$-Cl-Pre

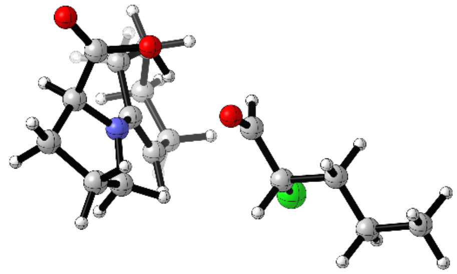

- Thermochemistry -

Zero-point correction=	0.408940 (Hartree/Particle)
Thermal correction to Energy=	0.428585
Thermal correction to Enthalpy=	0.429450
Thermal correction to Gibbs Free Energy=	0.360176
Sum of electronic and zero-point Energies=	-1365.214185
Sum of electronic and thermal Energies=	-1365.194539
Sum of electronic and thermal Enthalpies=	-1365.193674
Sum of electronic and thermal Free Energies=	-1365.262948

Number of Imaginary Frequencies = 0

E (Single Point Energy) [IEFPCM$_{(DCM)}$M06-2X/6-311++G(2d,2p)] = -1365.901816

C	0.48089200	1.69985200	0.98085800
H	-0.10405800	1.21341500	1.75734100
C	1.46561400	1.01847700	0.35402100
N	1.74128600	-0.32407600	0.62709700
C	3.05267700	-0.90956400	0.34765800

H	3.86073100	-0.17287800	0.36819400
C	1.10672700	-0.97818400	1.77239800
H	0.02854800	-1.06714600	1.61006900
H	1.26990500	-0.39073600	2.68995400
C	1.81193000	-2.32786000	1.84563100
H	1.37899900	-3.01895500	1.11382600
H	1.73924100	-2.78373900	2.83448300
C	3.24680800	-1.96128400	1.46101100
H	3.75123000	-1.48655200	2.30738200
H	3.85393300	-2.80454900	1.12723000
C	2.29566400	1.64541700	-0.74844300
H	3.30507000	1.85356700	-0.36895100
H	2.41491100	0.92858800	-1.56999200
C	1.25136200	3.83545200	-0.11801700
C	-1.32306000	-0.07308200	-0.78571500
H	-1.12955800	0.86799600	-1.32800700
C	-2.57027700	-0.14578700	0.07648600
O	-0.62130300	-1.05275500	-0.91986900
C	3.12380500	-1.57900800	-1.02904600
O	1.98081500	-1.73890500	-1.68795500
O	4.17441100	-1.97092900	-1.48975900
H	1.22738400	-1.34828300	-1.19218800
C	-3.65115300	-0.95508600	-0.64403100
H	-3.19337100	-1.91899600	-0.89908000
H	-2.31692900	-0.61392000	1.03152200
Cl	-3.13592700	1.52168200	0.45368900
C	-4.90697800	-1.18719300	0.19291600
H	-5.36593200	-0.22454800	0.44060600

H	-3.90796400	-0.45509300	-1.58646200
H	-4.62263400	-1.65875600	1.14166300
C	-5.91534400	-2.06604700	-0.54455200
H	-6.81263300	-2.22545900	0.05880100
H	-6.22138900	-1.60056400	-1.48689000
H	-5.48596200	-3.04559700	-0.77739700
C	1.67699800	2.93903000	-1.27765200
C	0.15179200	3.14399900	0.68651300
H	2.39483200	3.44567400	-1.92961200
H	0.79726000	2.70083400	-1.89044000
H	2.11975800	4.02541200	0.52669900
H	0.89974000	4.80516500	-0.48406500
H	-0.79620500	3.20680900	0.13267200
H	-0.01927200	3.67417100	1.63068000

(*S*)-**TS3**$_{G}$-Cl

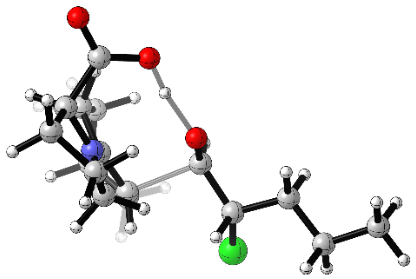

- Thermochemistry -

Zero-point correction= 0.408984 (Hartree/Particle)
Thermal correction to Energy= 0.426870
Thermal correction to Enthalpy= 0.427735
Thermal correction to Gibbs Free Energy= 0.364491
Sum of electronic and zero-point Energies= -1365.202940
Sum of electronic and thermal Energies= -1365.185053
Sum of electronic and thermal Enthalpies= -1365.184188
Sum of electronic and thermal Free Energies= -1365.247432

Number of Imaginary Frequencies = 1

E (Single Point Energy) [IEFPCM$_{(DCM)}$M06-2X/6-311++G(2d,2p)] = -1365.889090

C	0.03912200	1.48353800	0.73632400
H	-0.38577600	1.05747600	1.64113000
C	1.29739600	1.01375500	0.35036600
N	1.74766000	-0.17757800	0.76248900
C	3.01358100	-0.77668500	0.33205100
H	3.81429500	-0.03352500	0.31136000
C	1.11349300	-0.95763500	1.84273400
H	0.05132400	-1.08679700	1.64295000
H	1.24389600	-0.42230500	2.79239700
C	1.87715100	-2.27773400	1.82048700
H	1.43917500	-2.94456000	1.07003500
H	1.85403200	-2.78269900	2.78716100
C	3.28423100	-1.85056000	1.39995100
H	3.80860500	-1.39025700	2.24233500
H	3.90032600	-2.66024000	1.00751700
C	2.14781900	1.74672800	-0.66227500
H	3.09271500	2.00291300	-0.16420000
H	2.40542300	1.07256100	-1.48696200
C	0.80029000	3.76880100	-0.07360600
C	-1.01782000	0.13475900	-0.56287300
H	-1.05776500	0.92179500	-1.33200400
C	-2.32647500	-0.11998200	0.19346700
O	-0.34904300	-0.92327400	-0.75141100
C	2.98682300	-1.39837900	-1.07770900
O	1.85457400	-1.48090600	-1.73287300

O	4.03600500	-1.80065900	-1.54657600
H	0.98441000	-1.15526100	-1.26725100
C	-3.23215100	-1.01294200	-0.65616200
H	-2.63019900	-1.88734200	-0.92753000
H	-2.10347300	-0.60990300	1.14469400
Cl	-3.20224500	1.40309000	0.62042200
C	-4.50700000	-1.46906200	0.05071400
H	-5.12659000	-0.60081700	0.29687200
H	-3.48033600	-0.48656400	-1.58744300
H	-4.23782600	-1.94686000	1.00137900
C	-5.30649400	-2.44484700	-0.81109500
H	-6.21984600	-2.76451200	-0.30239300
H	-5.59476500	-1.97971600	-1.75941100
H	-4.71747900	-3.33826900	-1.04179200
C	1.49846300	3.02003900	-1.20278400
C	-0.36495300	2.91874400	0.42834900
H	2.26670500	3.63227500	-1.68277700
H	0.76216700	2.76587900	-1.97571300
H	1.51414000	3.95802900	0.73907100
H	0.43214200	4.73945500	-0.41902000
H	-1.15011700	2.93656900	-0.33886000
H	-0.81377600	3.36068000	1.32309800

(*S*)-**TS3**$_G$-Cl-P

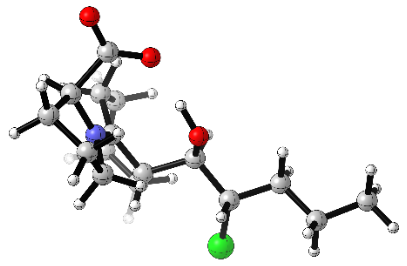

- Thermochemistry -

Zero-point correction=	0.412514 (Hartree/Particle)
Thermal correction to Energy=	0.430591
Thermal correction to Enthalpy=	0.431456
Thermal correction to Gibbs Free Energy=	0.367159
Sum of electronic and zero-point Energies=	-1365.226895
Sum of electronic and thermal Energies=	-1365.208818
Sum of electronic and thermal Enthalpies=	-1365.207953
Sum of electronic and thermal Free Energies=	-1365.272250

Number of Imaginary Frequencies = 0

E (Single Point Energy) [IEFPCM$_{(DCM)}$M06-2X/6-311++G(2d,2p)] = -1365.916168

C	-0.19960400	1.15668000	0.53203200
H	-0.30434100	0.86815600	1.58154400
C	1.23262600	0.95333400	0.11829300
N	1.94458400	0.03999300	0.69320600
C	3.26584600	-0.39863600	0.19914300
H	3.88510400	0.46827900	-0.03735600
C	1.51572500	-0.79706600	1.85301600
H	0.50875900	-1.17068700	1.68296900
H	1.54106300	-0.15915700	2.74206800
C	2.56886400	-1.90090200	1.91277000
H	2.27122900	-2.73244300	1.26644200
H	2.69170200	-2.27860700	2.92847400
C	3.82262600	-1.22240200	1.35933000

H	4.26779800	-0.56304900	2.11101600
H	4.58182200	-1.91904100	1.00469000
C	1.82663100	1.72791600	-1.02763900
H	2.79248100	2.12069900	-0.68618000
H	2.04374200	1.02081100	-1.83620100
C	0.34099200	3.56413500	-0.27241200
C	-1.04863900	0.13414500	-0.32521300
H	-1.25083800	0.61358800	-1.29601600
C	-2.38188600	-0.27942200	0.33499700
O	-0.42452300	-1.11170800	-0.49493000
C	3.11653300	-1.25304400	-1.11401400
O	1.96532100	-1.37420400	-1.61275100
O	4.18798700	-1.71496900	-1.53188300
H	0.43758700	-1.08774000	-0.98659100
C	-3.31606500	-0.93250000	-0.67495100
H	-2.70892400	-1.64877700	-1.24065100
H	-2.12653400	-0.98186700	1.13379500
Cl	-3.25214700	1.06806400	1.18242700
C	-4.50952100	-1.65568400	-0.05479500
H	-5.11988500	-0.94391200	0.51139600
H	-3.66163000	-0.16679300	-1.38196900
H	-4.14294600	-2.40061400	0.66265100
C	-5.36875300	-2.34010600	-1.11628000
H	-6.22057900	-2.85549700	-0.66474000
H	-5.75873200	-1.60881800	-1.83182000
H	-4.78547600	-3.07811800	-1.67630800
C	0.95378300	2.89140900	-1.49401200
C	-0.68869300	2.62269100	0.35067200

H	1.57225200	3.58028000	-2.07411200
H	0.15746800	2.53762400	-2.15976600
H	1.13472600	3.80931000	0.44597200
H	-0.15085200	4.50328000	-0.53939600
H	-1.56872600	2.61603100	-0.29951600
H	-1.02493100	3.00378500	1.31679400

(*R*)-**TS3**$_{O}$-Cl-Pre

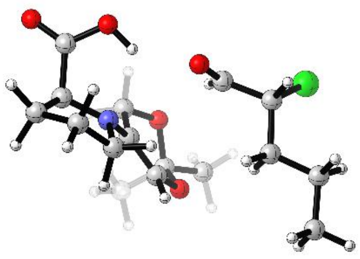

- Thermochemistry -

Zero-point correction= 0.416672 (Hartree/Particle)
Thermal correction to Energy= 0.438339
Thermal correction to Enthalpy= 0.439204
Thermal correction to Gibbs Free Energy= 0.365498
Sum of electronic and zero-point Energies= -1515.593202
Sum of electronic and thermal Energies= -1515.571535
Sum of electronic and thermal Enthalpies= -1515.570670
Sum of electronic and thermal Free Energies= -1515.644377

Number of Imaginary Frequencies = 0

E (Single Point Energy) [IEFPCM$_{(DCM)}$M06-2X/6-311++G(2d,2p)] = -1516.336463

C	-0.09905300	0.87518600	1.29666200
H	-0.30237300	0.26134000	2.16514400
C	0.96287400	0.72128300	0.48829100
N	1.91140100	-0.29701800	0.64081600
C	3.31128000	-0.04792700	0.28726000

H	3.57668700	1.01435400	0.31526000
C	1.81545900	-1.17712100	1.80637300
H	0.93483300	-1.82166600	1.71526000
H	1.72520800	-0.59013800	2.73502400
C	3.13819900	-1.93439600	1.77727800
H	3.09580200	-2.74170700	1.03830300
H	3.39284600	-2.36639500	2.74637900
C	4.11897200	-0.84378100	1.33692900
H	4.34647300	-0.18411600	2.17890000
H	5.05937300	-1.22078700	0.93145100
C	1.07526400	1.59199300	-0.73894500
H	1.86470300	2.35089000	-0.63839000
H	1.31145700	0.98170700	-1.61814700
O	-1.09485700	1.79589100	1.07085500
O	-0.17370000	2.20267400	-1.02050200
C	-0.79051000	2.80250500	0.10427200
C	-2.11817100	3.34387600	-0.37802000
H	-2.68141100	2.54112500	-0.86032800
H	-1.95104200	4.15101700	-1.09423300
C	0.09254000	3.87088900	0.74004900
H	0.41622700	4.58188900	-0.02444200
H	0.97150700	3.43318500	1.21910600
H	-0.48090100	4.40337400	1.50161600
C	-0.93000600	-1.15413900	-1.20752600
H	-0.84880100	-0.09394600	-1.50994600
C	-2.32075400	-1.64277300	-0.86594400
O	0.03755600	-1.88042700	-1.12607100
C	3.64550100	-0.54585100	-1.12339600

O	2.72725500	-1.28658600	-1.73555300
O	4.70986600	-0.30008700	-1.64801400
H	-2.68926100	3.72782500	0.46969600
C	-2.68419900	-1.24823100	0.56403600
H	-1.90587500	-1.67884700	1.20932400
C	-4.05326100	-1.75323600	1.01483900
H	-4.83092100	-1.31634400	0.37987600
H	-4.10074700	-2.84022000	0.87574100
H	-2.62183500	-0.15695800	0.66430700
C	-4.32138800	-1.40114000	2.47677500
H	-5.30552000	-1.75599300	2.79332200
H	-3.57164100	-1.85452500	3.13315400
H	-4.28882100	-0.31729900	2.62755100
H	1.91155900	-1.36338700	-1.18974800
H	-2.37131400	-2.72421500	-1.00821500
Cl	-3.45537900	-0.90209300	-2.06123400

(*R*)-**TS3**$_{O}$-Cl

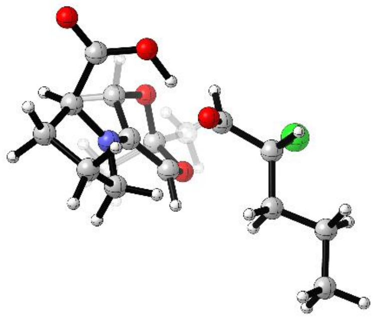

- Thermochemistry -

Zero-point correction= 0.415698 (Hartree/Particle)
Thermal correction to Energy= 0.435638
Thermal correction to Enthalpy= 0.436503

Thermal correction to Gibbs Free Energy= 0.368870
Sum of electronic and zero-point Energies= -1515.578488
Sum of electronic and thermal Energies= -1515.558548
Sum of electronic and thermal Enthalpies= -1515.557683
Sum of electronic and thermal Free Energies= -1515.625316

Number of Imaginary Frequencies = 1

E (Single Point Energy) [IEFPCM$_{(DCM)}$M06-2X/6-311++G(2d,2p)] = -1516.319628

C	-0.42402200	0.46841500	0.80107400
H	-0.73062000	-0.32460600	1.47282600
C	0.92458700	0.66601500	0.52654900
N	1.82996100	-0.29432900	0.72129400
C	3.22007200	-0.20703600	0.27076700
H	3.62165300	0.79811600	0.42661600
C	1.58735300	-1.48220000	1.56655300
H	0.69590200	-2.01302900	1.23296800
H	1.45325700	-1.15274400	2.60508500
C	2.86136300	-2.30376300	1.38184900
H	2.76656000	-2.93989000	0.49557500
H	3.06441500	-2.94161300	2.24297600
C	3.93673000	-1.23712500	1.16014400
H	4.20534000	-0.76342200	2.10868100
H	4.84478600	-1.61145100	0.68669100
C	1.32552300	1.92483500	-0.19905600
H	2.04502300	2.49735200	0.40434700
H	1.80416500	1.68199800	-1.15481100
O	-1.32139400	1.51613600	0.78439700
O	0.18630400	2.69322300	-0.51931100
C	-0.75836300	2.80379400	0.53272700
C	-1.88084200	3.67168200	0.01057700

H	-2.24343000	3.26055900	-0.93338700
H	-1.51857500	4.68995600	-0.14559400
C	-0.13297400	3.35262200	1.81212200
H	0.40945600	4.27544100	1.59042600
H	0.55352200	2.63960500	2.27564300
H	-0.92678600	3.57069400	2.52915500
C	-0.64691000	-0.54009600	-1.04847300
H	-0.47508400	0.37884500	-1.63074300
C	-2.11720000	-0.97737100	-0.99783100
O	0.19054700	-1.49553800	-1.09727900
C	3.43061400	-0.52769400	-1.22280800
O	2.43173200	-0.98251000	-1.93157800
O	4.54074200	-0.34534700	-1.69140500
H	-2.69817000	3.68720500	0.73437300
C	-2.59368000	-1.65116800	0.27970200
H	-1.77380400	-2.29867400	0.62166800
C	-3.85681400	-2.48908500	0.09070700
H	-4.66218600	-1.84991200	-0.28835100
H	-3.67017400	-3.25265100	-0.67417600
H	-2.76676000	-0.89051400	1.04996000
C	-4.29481300	-3.15501800	1.39322400
H	-5.19663700	-3.75511400	1.24711000
H	-3.51011700	-3.81420300	1.77870500
H	-4.51037500	-2.40488000	2.16099900
H	1.48544900	-1.17184800	-1.47539600
H	-2.19395400	-1.68879900	-1.82702300
Cl	-3.21116200	0.38031300	-1.47582000

(*R*)-**TS3**$_O$-Cl-P

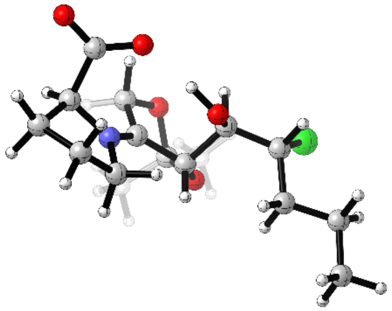

- Thermochemistry -

Zero-point correction=	0.420324 (Hartree/Particle)
Thermal correction to Energy=	0.440275
Thermal correction to Enthalpy=	0.441141
Thermal correction to Gibbs Free Energy=	0.373824
Sum of electronic and zero-point Energies=	-1515.603394
Sum of electronic and thermal Energies=	-1515.583443
Sum of electronic and thermal Enthalpies=	-1515.582578
Sum of electronic and thermal Free Energies=	-1515.649894

Number of Imaginary Frequencies = 0

E (Single Point Energy) [IEFPCM$_{(DCM)}$M06-2X/6-311++G(2d,2p)] = -1516.348890

C	-0.41014700	0.21455300	0.50441100
H	-0.55571800	-0.53783200	1.28320400
C	1.04632000	0.55844300	0.36344500
N	1.97710600	-0.25699300	0.70430100
C	3.37365800	-0.11200900	0.25134700
H	3.69542500	0.92776400	0.34386800
C	1.79638500	-1.53837900	1.45583200
H	0.97570200	-2.10491200	1.02062500
H	1.56814400	-1.26951800	2.49138800
C	3.15435600	-2.22734900	1.32255600
H	3.16557900	-2.85673800	0.42770600

H	3.36431700	-2.85565000	2.18860900
C	4.13904000	-1.06585500	1.16424000
H	4.33806000	-0.59000700	2.12924600
H	5.08496700	-1.35433100	0.70677100
C	1.35049500	1.84922400	-0.34729400
H	2.09431900	2.42682200	0.21834900
H	1.76233700	1.62383000	-1.33707700
O	-1.23098200	1.31620300	0.81782400
O	0.16123400	2.57791000	-0.54235300
C	-0.68561800	2.62100400	0.59444600
C	-1.84207700	3.52711200	0.23598000
H	-2.27526600	3.20115400	-0.71081300
H	-1.48992200	4.55709000	0.14701600
C	0.04809200	3.08187800	1.85189200
H	0.56991200	4.02311500	1.66009200
H	0.76939200	2.34265900	2.21279900
H	-0.68546900	3.23733600	2.64507800
C	-0.79294600	-0.41836100	-0.87838100
H	-0.60687400	0.32404700	-1.66573500
C	-2.26677200	-0.85205800	-0.94963000
O	-0.03347300	-1.58410300	-1.07456100
C	3.47683900	-0.52554000	-1.26929400
O	2.39917200	-0.75390900	-1.88101100
O	4.63677500	-0.55925000	-1.69755000
H	-2.60312000	3.47257100	1.01697400
C	-2.78481200	-1.54644800	0.29979600
H	-2.02541600	-2.28933400	0.58412300
C	-4.12507400	-2.25306700	0.10620800

H	-4.88491200	-1.51877800	-0.18229600
H	-4.03889500	-2.96391400	-0.72480900
H	-2.86034200	-0.81921800	1.11820400
C	-4.56805700	-2.98485400	1.37140100
H	-5.52878500	-3.48488300	1.22362300
H	-3.83445000	-3.74375300	1.66261900
H	-4.67765100	-2.28597700	2.20712700
H	0.86202400	-1.33978400	-1.41656900
H	-2.32641600	-1.52715100	-1.80658800
Cl	-3.33038800	0.54469200	-1.40083200

(*S*)-**TS3**$_{O}$-Cl-Pre

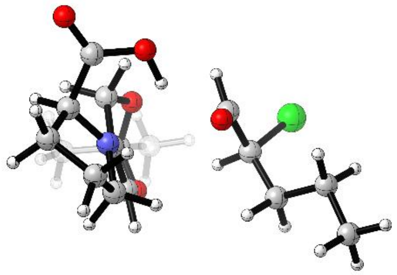

- Thermochemistry -

Zero-point correction=	0.416907 (Hartree/Particle)
Thermal correction to Energy=	0.438397
Thermal correction to Enthalpy=	0.439262
Thermal correction to Gibbs Free Energy=	0.366707
Sum of electronic and zero-point Energies=	-1515.593725
Sum of electronic and thermal Energies=	-1515.572234
Sum of electronic and thermal Enthalpies=	-1515.571369
Sum of electronic and thermal Free Energies=	-1515.643925

Number of Imaginary Frequencies = 0

E (Single Point Energy) [IEFPCM$_{(DCM)}$M06-2X/6-311++G(2d,2p)] = -1516.337380

C	0.12541100	1.02635500	1.50424600

H	-0.02816300	0.42583900	2.39197200
C	1.03381700	0.75790800	0.55064300
N	1.86254700	-0.36846600	0.56796900
C	3.20542200	-0.29768300	-0.01249200
H	3.60019300	0.72306900	-0.05316100
C	1.84191100	-1.24116600	1.74335400
H	0.88683600	-1.77467400	1.79277000
H	1.96499200	-0.65447700	2.66820800
C	3.04493000	-2.15089500	1.51947400
H	2.79149400	-2.94659900	0.81073100
H	3.39600000	-2.61220400	2.44386600
C	4.06761600	-1.18604900	0.91186200
H	4.50179900	-0.55907800	1.69560000
H	4.88053300	-1.67380900	0.37132600
C	1.07006000	1.62724700	-0.68204300
H	1.92759400	2.31485400	-0.68189300
H	1.14111200	1.01191600	-1.58534600
O	-0.78433900	2.05600900	1.41652000
O	-0.14865600	2.34486200	-0.79586400
C	-0.52270500	3.01992100	0.39204100
C	-1.84004600	3.70310700	0.09721900
H	-2.24527200	4.13324000	1.01519100
H	-2.54967600	2.97605000	-0.30641900
C	0.55191100	3.98920600	0.87161700
H	1.43330400	3.46193300	1.24387400
H	0.14840500	4.59397900	1.68625600
H	0.84554100	4.64821800	0.05048500
C	-1.16474800	-0.72289200	-1.05986400

H	-0.94030300	-0.09051000	-1.93793700
C	-2.38762100	-0.31957200	-0.26162300
O	-0.45309500	-1.65448300	-0.75222500
C	3.24294300	-0.83751200	-1.44690700
O	2.16195900	-1.48118000	-1.87974900
O	4.22192200	-0.70932100	-2.14867400
H	1.45228600	-1.47748200	-1.19903300
H	-1.68714100	4.49821100	-0.63545400
C	-2.85429100	-1.35621000	0.74633600
H	-3.62893500	-0.90272900	1.37410600
H	-2.13011900	0.62032400	0.23995900
Cl	-3.67383600	0.13417500	-1.45367000
C	-3.36395300	-2.66842400	0.15118500
H	-2.59885400	-3.09584200	-0.50573500
H	-1.99095500	-1.55552700	1.39535400
H	-4.24396500	-2.46733700	-0.46906700
C	-3.72433700	-3.67098800	1.24584900
H	-2.84963900	-3.91232400	1.85851000
H	-4.49608100	-3.26512500	1.90821200
H	-4.10384600	-4.60285800	0.81879500

(*S*)-**TS3**$_{O}$-Cl

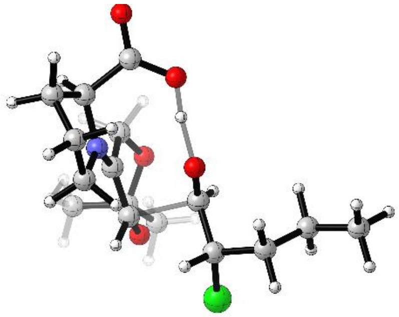

- Thermochemistry -

Zero-point correction=	0.415932 (Hartree/Particle)
Thermal correction to Energy=	0.435791
Thermal correction to Enthalpy=	0.436656
Thermal correction to Gibbs Free Energy=	0.369175
Sum of electronic and zero-point Energies=	-1515.577897
Sum of electronic and thermal Energies=	-1515.558038
Sum of electronic and thermal Enthalpies=	-1515.557173
Sum of electronic and thermal Free Energies=	-1515.624654

Number of Imaginary Frequencies = 1

E (Single Point Energy) [IEFPCM$_{(DCM)}$M06-2X/6-311++G(2d,2p)] = -1516.318913

C	-0.12290700	1.09566100	0.86396300
H	-0.30907500	0.82990900	1.90069500
C	1.10661300	0.80725000	0.26938200
N	1.87566600	-0.19213200	0.68999500
C	3.10446300	-0.62342600	0.01536900
H	3.70137600	0.23902700	-0.29488000
C	1.67811800	-0.87738000	1.98284600
H	0.64731000	-1.21776800	2.07526000
H	1.90939700	-0.17509300	2.79413000
C	2.67628900	-2.02907100	1.91628800
H	2.22674000	-2.87568600	1.38689900
H	2.98304600	-2.36339100	2.90803500

C	3.82907600	-1.43848800	1.10073300
H	4.42437400	-0.76106700	1.71942000
H	4.49278700	-2.18365900	0.66163200
C	1.47560500	1.51015800	-1.01442600
H	2.41575800	2.06374900	-0.87294000
H	1.62687500	0.78272000	-1.81848500
O	-0.82823300	2.22290500	0.47901600
O	0.43732000	2.35785400	-1.44716200
C	-0.15984300	3.11421600	-0.40725900
C	-1.23505400	3.95689100	-1.05643800
H	-1.80618800	4.47976900	-0.28692900
H	-1.90532100	3.30975800	-1.62619800
C	0.86306600	3.95089600	0.35656900
H	1.53503800	3.33437300	0.95909100
H	0.33485700	4.62874400	1.02991100
H	1.45740000	4.53960400	-0.34702500
C	-1.06729300	-0.53992500	-0.05060100
H	-1.30759600	0.10105200	-0.91318500
C	-2.22192100	-0.82600700	0.91456500
O	-0.27928300	-1.53293000	-0.17341600
C	2.88738200	-1.46502100	-1.26035000
O	1.67992800	-1.82510500	-1.60455700
O	3.87278500	-1.75766700	-1.91420100
H	0.86357400	-1.60995100	-0.96018100
H	-0.77806300	4.68855900	-1.72596200
C	-3.02308800	-2.04066900	0.43483500
H	-3.83611900	-2.21156400	1.14968400
H	-1.81819600	-1.02889500	1.90880100

Cl	-3.32316100	0.59204800	1.11535300
C	-3.58482200	-1.92662400	-0.98150000
H	-2.75928300	-1.82618300	-1.69604100
H	-2.34716800	-2.90112800	0.49344900
H	-4.19336400	-1.01913900	-1.06559200
C	-4.42681300	-3.14794600	-1.34652000
H	-3.83228100	-4.06511500	-1.28258200
H	-5.27786900	-3.25180600	-0.66553700
H	-4.81729900	-3.07026600	-2.36459800

(*S*)-**TS3**$_{\mathrm{O}}$-Cl-P

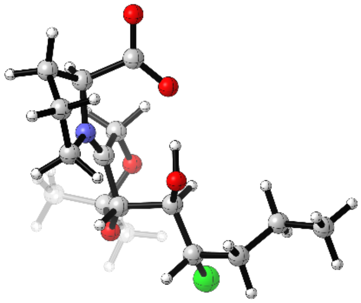

- Thermochemistry -

Zero-point correction=	0.420174 (Hartree/Particle)
Thermal correction to Energy=	0.440065
Thermal correction to Enthalpy=	0.440930
Thermal correction to Gibbs Free Energy=	0.373427
Sum of electronic and zero-point Energies=	-1515.601495
Sum of electronic and thermal Energies=	-1515.581603
Sum of electronic and thermal Enthalpies=	-1515.580738
Sum of electronic and thermal Free Energies=	-1515.648241

Number of Imaginary Frequencies = 0

E (Single Point Energy) [IEFPCM$_{\mathrm{(DCM)}}$M06-2X/6-311++G(2d,2p)] = -1516.346594

C	-0.24245700	0.77330400	0.86113700
H	-0.10667900	0.55895500	1.92513100

C	1.07458200	0.75068100	0.13175600
N	2.09646000	0.11025900	0.56996400
C	3.26004900	-0.21574800	-0.27690900
H	3.55519200	0.65611100	-0.86542000
C	2.22256300	-0.53896700	1.91213400
H	1.32391500	-1.11480800	2.12280000
H	2.34336400	0.26492800	2.64418500
C	3.48322400	-1.39303200	1.78035900
H	3.22044000	-2.39041600	1.41525800
H	3.98978700	-1.49932300	2.74004300
C	4.31774100	-0.65033400	0.73370500
H	4.80433500	0.22555600	1.17363500
H	5.07157300	-1.27323700	0.25319900
C	1.07625300	1.40182300	-1.22455700
H	1.94956200	2.05942900	-1.33042200
H	1.13101900	0.62289300	-1.99296100
O	-0.90802600	2.01054900	0.76826500
O	-0.13080200	2.10070600	-1.41756300
C	-0.53004900	2.88233300	-0.30402600
C	-1.77187700	3.63360700	-0.72938200
H	-2.21810400	4.12166100	0.13941100
H	-2.49054200	2.93254000	-1.15593700
C	0.57634600	3.81685300	0.18058100
H	1.40805400	3.27879800	0.64505300
H	0.15942400	4.48743800	0.93429300
H	0.95872100	4.41196100	-0.65286700
C	-1.05607700	-0.42067300	0.24329200
H	-1.20306900	-0.23877600	-0.83081100

C	-2.43046200	-0.57904900	0.92206700
O	-0.33361400	-1.60178700	0.47926900
C	2.87259600	-1.37550400	-1.27684500
O	1.66127800	-1.71996900	-1.32224200
O	3.82898200	-1.80829300	-1.93087500
H	0.38197900	-1.70000300	-0.19729000
H	-1.50831000	4.38999700	-1.47191900
C	-3.01659800	-1.97971200	0.76194900
H	-3.99408900	-1.98939100	1.25824400
H	-2.35083600	-0.33136200	1.98398000
Cl	-3.60281500	0.63240900	0.24838000
C	-3.15987000	-2.46383100	-0.67983600
H	-2.17310800	-2.48384000	-1.15579100
H	-2.36386800	-2.66482700	1.31268200
H	-3.77854200	-1.75862300	-1.24599300
C	-3.78387000	-3.85719600	-0.73901100
H	-3.16856100	-4.58350400	-0.19794200
H	-4.78046800	-3.85809700	-0.28506600
H	-3.88366300	-4.20382800	-1.77100800

(*R*)-**TS3**$_T$-Cl-Pre

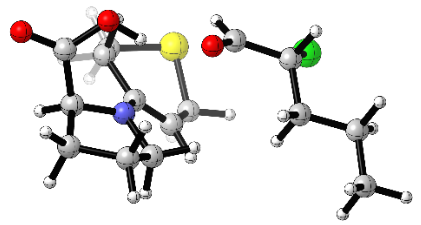

- Thermochemistry -

Zero-point correction= 0.381447 (Hartree/Particle)
Thermal correction to Energy= 0.400534

Thermal correction to Enthalpy= 0.401399
Thermal correction to Gibbs Free Energy= 0.335088
Sum of electronic and zero-point Energies= -1724.101463
Sum of electronic and thermal Energies= -1724.082376
Sum of electronic and thermal Enthalpies= -1724.081511
Sum of electronic and thermal Free Energies= -1724.147822

Number of Imaginary Frequencies = 0

E (Single Point Energy) [IEFPCM$_{(DCM)}$M06-2X/6-311++G(2d,2p)] = -1724.784650

C	0.09881400	0.95207300	-1.45101900
H	0.54963300	0.12747500	-1.99553000
C	-1.06036100	0.74551700	-0.78743600
N	-1.60614800	-0.53177700	-0.65180300
C	-3.03794200	-0.75838600	-0.46432200
H	-3.66100400	-0.03883500	-1.00422100
C	-0.96752400	-1.68203300	-1.29035400
H	-0.91553000	-1.54949300	-2.38291100
H	0.04998000	-1.80184600	-0.90768200
C	-1.89011300	-2.83687300	-0.92131300
H	-1.77966800	-3.69223900	-1.58995200
H	-1.68312000	-3.16785200	0.10231200
C	-3.27166700	-2.18568000	-1.01442800
H	-3.57930200	-2.10918300	-2.06094500
H	-4.05462000	-2.71361200	-0.46682200
C	-1.79541700	1.83440900	-0.03263000
H	-2.87082900	1.76034700	-0.22999400
H	-1.66524700	1.66723700	1.04574000
C	1.05323100	-0.06476500	1.44039600
H	0.77128400	0.99850200	1.52996000
C	2.52081300	-0.37953400	1.19814200
O	0.24681600	-0.96121200	1.57675000

C	-3.46747500	-0.68585500	1.00310600
O	-2.51546300	-0.80116400	1.92351900
O	-4.63261100	-0.57171100	1.31734500
H	-1.61758400	-0.83961800	1.52317900
C	2.73396900	-1.43766400	0.12237900
H	2.40632000	-1.03122800	-0.84306000
C	4.17203000	-1.94056800	0.02573200
H	2.06543800	-2.27103400	0.37242400
C	4.31153600	-3.03911700	-1.02602600
H	5.34368700	-3.39219900	-1.09309600
H	3.67611300	-3.89686600	-0.78329000
H	4.01663700	-2.67146000	-2.01419300
H	4.48559400	-2.31980700	1.00612200
H	4.83699500	-1.10558300	-0.21950100
H	2.93320700	-0.72335000	2.15369000
C	-1.35770800	3.24936100	-0.38733900
C	0.84381700	2.25617900	-1.56274700
H	-1.66132600	3.50748600	-1.40682800
H	-1.81590000	3.96767800	0.29483700
H	1.91962900	2.07654600	-1.48639000
H	0.66292600	2.73382300	-2.53333600
S	0.44133600	3.44585800	-0.24879500
Cl	3.39190600	1.15200700	0.81641600

(R)-**TS3**$_T$-Cl

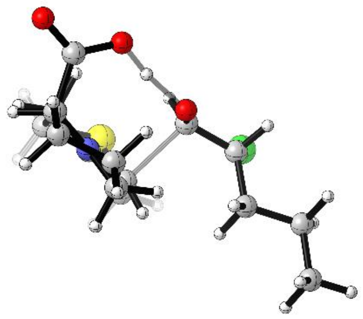

- Thermochemistry -

Zero-point correction=	0.381605 (Hartree/Particle)
Thermal correction to Energy=	0.399723
Thermal correction to Enthalpy=	0.400588
Thermal correction to Gibbs Free Energy=	0.336882
Sum of electronic and zero-point Energies=	-1724.088101
Sum of electronic and thermal Energies=	-1724.069983
Sum of electronic and thermal Enthalpies=	-1724.069118
Sum of electronic and thermal Free Energies=	-1724.132824

Number of Imaginary Frequencies = 1

E (Single Point Energy) [IEFPCM$_{(DCM)}$M06-2X/6-311++G(2d,2p)] = -1724.769951

C	-0.37664700	0.61019700	1.03803500
H	-0.74792700	-0.27619500	1.54239300
C	1.00017000	0.64904300	0.80228500
N	1.71633400	-0.48396200	0.79614000
C	3.12172700	-0.58204700	0.38547300
H	3.73481300	0.17624300	0.87602800
C	1.20793100	-1.77648300	1.30412500
H	1.17254000	-1.73704100	2.40054100
H	0.21256400	-1.97595200	0.91428400
C	2.23522700	-2.78202800	0.79869100
H	2.26052200	-3.68576600	1.40920100
H	1.99432300	-3.06447300	-0.23162900

C	3.54149400	-1.99032800	0.84865100
H	3.90904800	-1.92948800	1.87686600
H	4.33621800	-2.39821700	0.22253600
C	1.69333700	1.90600800	0.32267600
H	2.73448900	1.90284600	0.65547000
H	1.71437500	1.88267800	-0.77659800
C	-0.76509400	-0.03804100	-1.01277500
H	-0.55601300	0.97283800	-1.39035800
C	-2.24762100	-0.42754300	-1.00519500
O	0.02731300	-0.99298800	-1.26383400
C	3.35660300	-0.40897900	-1.12517400
O	2.34538400	-0.51785700	-1.95041700
O	4.49290100	-0.20040900	-1.51121300
H	1.38339500	-0.71036500	-1.56620700
C	-2.72005800	-1.33064800	0.12285900
H	-2.80671400	-0.75241100	1.05032500
C	-4.04367100	-2.03437300	-0.17114900
H	-1.93323900	-2.08285100	0.27113100
C	-4.46687200	-2.94676100	0.97796400
H	-5.41285500	-3.44767000	0.75695200
H	-3.71230000	-3.71800300	1.16383900
H	-4.59713900	-2.37463000	1.90230700
H	-3.94170400	-2.61885000	-1.09356100
H	-4.82105500	-1.28434700	-0.35472600
H	-2.37940300	-0.94998200	-1.95874500
C	1.05250400	3.20890900	0.79211900
C	-1.17783000	1.85223300	1.35970700
H	1.16426700	3.32717500	1.87438700

H	1.54641600	4.05321300	0.30830100
H	-2.23938700	1.67978700	1.17711000
H	-1.07086100	2.11352300	2.41945500
S	-0.70911800	3.30000900	0.36907900
Cl	-3.29440000	1.04238800	-1.16197600

(*R*)-**TS3**$_{T}$-Cl-P

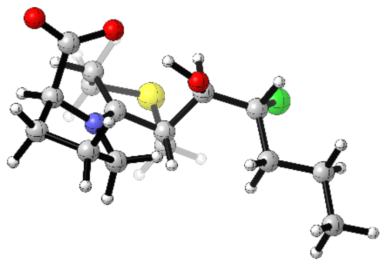

- Thermochemistry -

Zero-point correction=	0.386030 (Hartree/Particle)
Thermal correction to Energy=	0.404208
Thermal correction to Enthalpy=	0.405073
Thermal correction to Gibbs Free Energy=	0.341118
Sum of electronic and zero-point Energies=	-1724.116573
Sum of electronic and thermal Energies=	-1724.098395
Sum of electronic and thermal Enthalpies=	-1724.097530
Sum of electronic and thermal Free Energies=	-1724.161485

Number of Imaginary Frequencies = 0

E (Single Point Energy) [IEFPCM$_{(DCM)}$M06-2X/6-311++G(2d,2p)] = -1724.802661

C	0.42489400	0.40460100	-0.63807900
H	0.61700300	-0.48138200	-1.24651300
C	-1.06507600	0.61106500	-0.52660000
N	-1.87415700	-0.36933500	-0.75152500
C	-3.30992800	-0.35934100	-0.38577100
H	-3.79498700	0.53860200	-0.76794000

C	-1.47202100	-1.73547200	-1.21377800
H	-1.27900900	-1.67083300	-2.28868400
H	-0.57679700	-2.05213600	-0.68375800
C	-2.68833900	-2.60241600	-0.90120600
H	-2.75169700	-3.45399100	-1.57955800
H	-2.62101300	-2.97893600	0.12407400
C	-3.85807000	-1.62896200	-1.03422700
H	-4.08933200	-1.44495700	-2.08799100
H	-4.76479400	-1.95547200	-0.52512200
C	-1.56564400	1.92854100	-0.00054900
H	-2.65091500	1.99301500	-0.06465600
H	-1.31310800	1.95251300	1.06702200
C	0.91129600	0.08863200	0.81769400
H	0.79051600	0.98550700	1.43814700
C	2.37876700	-0.36434700	0.91383000
O	0.17713100	-0.99608100	1.33821700
C	-3.45594300	-0.37872200	1.18010000
O	-2.39866400	-0.35459900	1.86519500
O	-4.63153400	-0.40792600	1.57147200
H	-0.74844700	-0.73224200	1.56809500
C	2.80492700	-1.38960400	-0.12492800
H	2.85781000	-0.92323600	-1.11752900
C	4.13339100	-2.07466300	0.19087800
H	2.00826400	-2.14619400	-0.15998400
C	4.48663500	-3.13547400	-0.84928900
H	5.43865000	-3.61806600	-0.61378100
H	3.71675900	-3.91266800	-0.89367700
H	4.57287900	-2.69070000	-1.84609000

H	4.07048000	-2.53367700	1.18501900
H	4.92857800	-1.32282700	0.23840400
H	2.48481500	-0.78545200	1.91587500
C	-0.94876800	3.14663800	-0.69818800
C	1.12067300	1.57976100	-1.34331800
H	-1.22577300	3.17400000	-1.75641800
H	-1.33203700	4.05265700	-0.22627000
H	2.19643600	1.40916000	-1.38101100
H	0.75974700	1.64162200	-2.37496700
S	0.85726600	3.18572200	-0.54320000
Cl	3.50309200	1.06452500	0.91239400

(*S*)-**TS3**$_{T}$-Cl-Pre

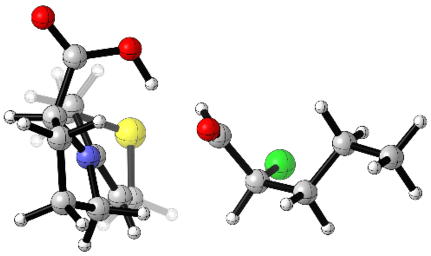

- Thermochemistry -

Zero-point correction=	0.381616 (Hartree/Particle)
Thermal correction to Energy=	0.401423
Thermal correction to Enthalpy=	0.402288
Thermal correction to Gibbs Free Energy=	0.333162
Sum of electronic and zero-point Energies=	-1724.097998
Sum of electronic and thermal Energies=	-1724.078191
Sum of electronic and thermal Enthalpies=	-1724.077326
Sum of electronic and thermal Free Energies=	-1724.146451

Number of Imaginary Frequencies = 0

E (Single Point Energy) [IEFPCM$_{(DCM)}$M06-2X/6-311++G(2d,2p)] = -1724.781967

C	0.49180400	1.34244300	1.40228400

H	0.11309100	0.75779600	2.23521800
C	1.43503200	0.80130800	0.59439300
N	1.85226200	-0.51475700	0.75248200
C	2.96648400	-1.11138000	0.02851000
H	3.91574800	-0.58211600	0.18471300
C	1.53871500	-1.25365200	1.98154900
H	0.53161800	-1.68659500	1.91818200
H	1.56708100	-0.57928100	2.84433400
C	2.63246300	-2.32008700	2.07144700
H	2.28509800	-3.23600700	2.55251500
H	3.48136600	-1.93199600	2.64251300
C	3.04180800	-2.53446600	0.61592800
H	4.03545100	-2.96653900	0.49056200
H	2.31402600	-3.17908200	0.11007900
C	2.04754100	1.54367700	-0.57642800
H	3.13048400	1.37053400	-0.58322900
H	1.65507100	1.13485900	-1.51657000
C	-1.30965100	-0.25199900	-0.40671700
H	-0.96647900	0.58011800	-1.04471600
C	-2.52339700	0.00341500	0.47391800
O	-0.75612600	-1.33373600	-0.40559400
C	2.76177900	-1.21784000	-1.47885200
O	1.52089800	-1.42487800	-1.91179600
O	3.69365100	-1.18373800	-2.25229300
H	0.84284900	-1.40114300	-1.19561200
C	-3.43297300	-1.21277300	0.61170900
H	-4.21605100	-0.97435900	1.33906100
H	-2.15145400	0.30581000	1.45739700

C	-4.05642900	-1.70161200	-0.69470700
H	-3.26484600	-1.92281100	-1.42024300
H	-2.81595300	-2.00769000	1.04815800
H	-4.67196700	-0.90505100	-1.12593100
C	-4.90964200	-2.94860500	-0.47068100
H	-4.30635100	-3.76809600	-0.06713700
H	-5.36097500	-3.29069000	-1.40545600
H	-5.71789400	-2.74501200	0.23920900
C	1.81195400	3.04801200	-0.55417800
C	-0.11342300	2.71597700	1.28028100
H	2.35911600	3.51863800	0.26866300
H	2.15317300	3.49557500	-1.48923300
H	-1.18885700	2.66972900	1.48269400
H	0.31585000	3.41328600	2.00998200
S	0.04841400	3.44224900	-0.37822000
Cl	-3.39643800	1.44598500	-0.17084000

(*S*)-**TS3**$_T$-Cl

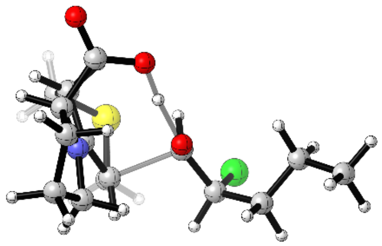

- Thermochemistry -

Zero-point correction= 0.381745 (Hartree/Particle)
Thermal correction to Energy= 0.399805
Thermal correction to Enthalpy= 0.400670
Thermal correction to Gibbs Free Energy= 0.336822
Sum of electronic and zero-point Energies= -1724.087754
Sum of electronic and thermal Energies= -1724.069694

Sum of electronic and thermal Enthalpies= -1724.068829
Sum of electronic and thermal Free Energies= -1724.132677

Number of Imaginary Frequencies = 1

E (Single Point Energy) [IEFPCM$_{(DCM)}$M06-2X/6-311++G(2d,2p)] = -1724.769924

C	-0.05294200	1.17320000	-1.15617600
H	0.13286400	0.61537900	-2.07035500
C	-1.25252400	0.89309300	-0.49664200
N	-1.91703400	-0.23371900	-0.77783100
C	-2.98838100	-0.80306800	0.05033700
H	-3.87371200	-0.15871500	0.03907700
C	-1.73233700	-0.99569900	-2.03286400
H	-0.82507500	-1.60262900	-1.96898200
H	-1.64144100	-0.29731600	-2.86844800
C	-2.99208700	-1.85428700	-2.10842500
H	-2.83593400	-2.75945600	-2.69705900
H	-3.81088300	-1.28327600	-2.55686300
C	-3.29377300	-2.14560900	-0.63947500
H	-4.31925500	-2.46325100	-0.44889100
H	-2.61205300	-2.91557600	-0.26144500
C	-1.72565900	1.70838500	0.68562200
H	-2.81536300	1.63635200	0.76163500
H	-1.31123300	1.26959400	1.60364100
C	1.05660400	-0.16419900	0.09362600
H	1.01799400	0.52246600	0.95207700
C	2.39663000	-0.16524000	-0.66973200
O	0.42521800	-1.26238300	0.13906100
C	-2.62040300	-1.04564300	1.51720000
O	-1.37659100	-1.33497500	1.80821300

O	-3.50323300	-1.02533400	2.35624900
H	-0.65193200	-1.30410700	1.03201400
C	3.07919700	-1.53107200	-0.63935400
H	3.97257000	-1.47477400	-1.27185200
H	2.26798100	0.14650400	-1.70706300
C	3.45352200	-2.05514000	0.74586100
H	2.56169300	-2.07682500	1.38144700
H	2.38261400	-2.22851800	-1.11939900
H	4.16805000	-1.37046600	1.21504400
C	4.06180900	-3.45421200	0.66258900
H	3.35089400	-4.16422900	0.22755000
H	4.34291800	-3.82316100	1.65247200
H	4.96014600	-3.45306200	0.03619400
C	-1.33569500	3.18232200	0.61573600
C	0.58547600	2.54280000	-1.15000600
H	-1.83726600	3.68011800	-0.21973400
H	-1.63384200	3.68520800	1.53722900
H	1.65148500	2.46830700	-1.37740900
H	0.13807000	3.17867500	-1.92284000
S	0.45868200	3.39989500	0.44651200
Cl	3.47654500	1.09871900	0.06203600

(*S*)-**TS3**$_{T}$-Cl-P

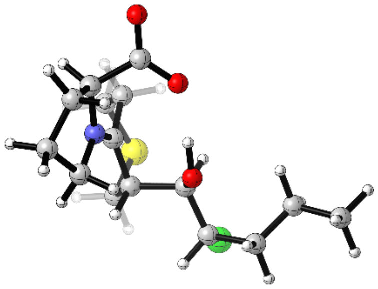

- Thermochemistry -

Zero-point correction=	0.386059 (Hartree/Particle)
Thermal correction to Energy=	0.404121
Thermal correction to Enthalpy=	0.404986
Thermal correction to Gibbs Free Energy=	0.341308
Sum of electronic and zero-point Energies=	-1724.113543
Sum of electronic and thermal Energies=	-1724.095482
Sum of electronic and thermal Enthalpies=	-1724.094617
Sum of electronic and thermal Free Energies=	-1724.158295

Number of Imaginary Frequencies = 0

E (Single Point Energy) [IEFPCM$_{(DCM)}$M06-2X/6-311++G(2d,2p)] = -1724.799615

C	-0.19148300	0.91422600	0.89448100
H	-0.07222800	0.58467900	1.92965100
C	1.13140800	0.82074800	0.17707000
N	2.08832000	0.10507100	0.66590300
C	3.24383800	-0.37707900	-0.13798600
H	3.87063800	0.47400800	-0.41343900
C	2.12354900	-0.48242000	2.03469000
H	1.47708100	-1.36477500	2.03834500
H	1.76170700	0.24742900	2.75754600
C	3.59383000	-0.83999400	2.20777700
H	3.73203300	-1.59560000	2.98217400
H	4.16630600	0.05130100	2.48217500

C	3.98641000	-1.32846100	0.81506800
H	5.05985500	-1.30864300	0.62839400
H	3.62892000	-2.35220800	0.66259900
C	1.25986400	1.45421800	-1.18079400
H	2.28972300	1.40147900	-1.53666100
H	0.64887900	0.86930700	-1.87825300
C	-1.08986100	-0.18554700	0.21792200
H	-1.27856600	0.08169300	-0.83081500
C	-2.44553300	-0.35798000	0.93322000
O	-0.42430100	-1.41967900	0.32149000
C	2.77786300	-1.13284400	-1.41861800
O	1.57748900	-1.51813500	-1.46141400
O	3.67653100	-1.31460900	-2.25357500
H	0.30119300	-1.48353300	-0.35168500
C	-3.09207000	-1.71849100	0.68686500
H	-4.04976400	-1.73255100	1.22036800
H	-2.32591300	-0.19592400	2.00822800
C	-3.30824800	-2.07885600	-0.78184400
H	-2.34095000	-2.09596000	-1.29587400
H	-2.44582100	-2.46799900	1.15460700
H	-3.91955600	-1.30851100	-1.26510100
C	-3.98918500	-3.43901500	-0.92555100
H	-3.38382000	-4.22875400	-0.46870500
H	-4.14076200	-3.69748200	-1.97689900
H	-4.96766000	-3.43970700	-0.43373200
C	0.80002800	2.91799400	-1.19614800
C	-0.72734500	2.35229000	0.93089000
H	1.42860200	3.53262000	-0.54451100

H	0.89118500	3.30321800	-2.21281600
H	-1.69378900	2.38342300	1.43219100
H	-0.03655100	2.97401700	1.50954600
S	-0.93774000	3.09747200	-0.70986100
Cl	-3.60536900	0.94000400	0.39440400

Supplementary Table 11. Energies for enamine addition to 2-fluoropentanal. Reported energies for structures optimized at the IEFPCM$_{(DCM)}$M06-2X/6-311++G(2d,2p)//IEFPCM$_{(DCM)}$M06-2X/6-31+G(d,p) level of theory represent the sum of the thermal correction to Gibbs Free Energy computed at the IEFPCM$_{(DCM)}$M06-2X/6-31+G(d,p) level of theory and single point energies computed at the IEFPCM$_{(DCM)}$M06-2X/6-311++G(2d,2p). All energies are reported in Hartrees.

**Structure**	**Single Point Energies, E IEFPCM$_{(DCM)}$M06-2X/6-311++G(2d,2p)**	**Thermal Corrections to Gibbs Free Energies, IEFPCM$_{(DCM)}$M06-2X/6-31+G(d,p)**	**Gibbs Free Energies (G), IEFPCM$_{(DCM)}$M06-2X/6-31+G(d,p)**	**Gibbs Free Energies (G), IEFPCM$_{(DCM)}$M06-2X/6-311++G(2d,2p)// IEFPCM$_{(DCM)}$M06-2X/6-31+G(d,p)**
2-Fluoropentanal	-731.33393130	0.102879	-370.761392	-731.2310523
Enamine of Cyclohexanone (G)	-634.55147535	0.238392	-634.142903	-634.3130834
Enamine of Dioxane (O)	-784.98483791	0.244077	-784.522619	-784.7407609
Enamine of Tetrahydro-4H-thiopyranone (T)	-993.43210840	0.210205	-993.028735	-993.2219034
Enamine of Tetrahydro-4H-pyranone (P)	-670.45469021	0.214793	-670.055312	-670.2398972
(*R*)-**TS3**$_{P}$-F-Pre	-1041.446106	0.339911	-1040.814983	-1041.106195
(*R*)-**TS3**$_{P}$-F	-1041.435017	0.343573	-1040.80187	-1041.091444
(*R*)-**TS3**$_{P}$-F-P	-1041.467264	0.347343	-1040.830554	-1041.119921
(*S*)-**TS3**$_{P}$-F-Pre	-1041.445581	0.338545	-1040.815817	-1041.107036
(*S*)-**TS3**$_{P}$-F	-1041.43582	0.342145	-1040.804044	-1041.093675
(*S*)-**TS3**$_{P}$-F-P	-1041.466319	0.346494	-1040.83068	-1041.119825
(*R*)-**TS3**$_{G}$-F-Pre	-1005.539197	0.362756	-1004.899267	-1005.176441
(*R*)-**TS3**$_{G}$-F	-1005.531517	0.367347	-1004.888828	-1005.16417
(*R*)-**TS3**$_{G}$-F-P	-1005.55939	0.370408	-1004.914209	-1005.188982
(*S*)-**TS3**$_{G}$-F-Pre	-1005.542809	0.363097	-1004.902711	-1005.179712
(*S*)-**TS3**$_{G}$-F	-1005.532406	0.366469	-1004.890519	-1005.165937
(*S*)-**TS3**$_{G}$-F-P	-1005.560329	0.369905	-1004.915721	-1005.190424
(*R*)-**TS3**$_{O}$-F-Pre	-1155.975514	0.367878	-1155.283126	-1155.607636
(*R*)-**TS3**$_{O}$-F	-1155.960428	0.370379	-1155.266603	-1155.590049

(*R*)-**TS3**O-F-P	-1155.989945	0.375586	-1155.291196	-1155.614359
(*S*)-**TS3**O-F-Pre	-1155.97491	0.368238	-1155.282047	-1155.606672
(*S*)-**TS3**O-F	-1155.961139	0.370957	-1155.266948	-1155.590182
(*S*)-**TS3**O-F-P	-1155.988743	0.375469	-1155.290286	-1155.613274
(*R*)-**TS3**T-F-Pre	-1005.539197	0.362756	-1004.899267	-1005.176441
(*R*)-**TS3**T-F	-1005.531517	0.367347	-1004.888828	-1005.16417
(*R*)-**TS3**T-F-P	-1005.55939	0.370408	-1004.914209	-1005.188982
(*S*)-**TS3**T-F-Pre	-1005.542809	0.363097	-1004.902711	-1005.179712
(*S*)-**TS3**T-F	-1005.532406	0.366469	-1004.890519	-1005.165937
(*S*)-**TS3**T-F-P	-1005.560329	0.369905	-1004.915721	-1005.190424

(*R*)-**TS3**P-F-Pre

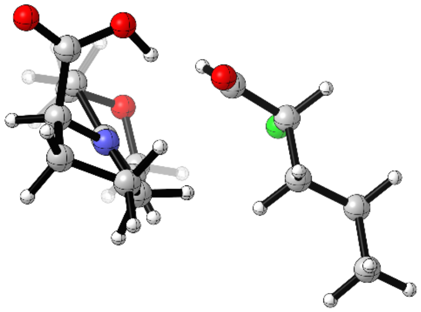

- Thermochemistry -

Zero-point correction= 0.386791 (Hartree/Particle)
Thermal correction to Energy= 0.405843
Thermal correction to Enthalpy= 0.406708
Thermal correction to Gibbs Free Energy= 0.339911
Sum of electronic and zero-point Energies= -1040.768103
Sum of electronic and thermal Energies= -1040.749052
Sum of electronic and thermal Enthalpies= -1040.748187
Sum of electronic and thermal Free Energies= -1040.814983

Number of Imaginary Frequencies = 0

E (Single Point Energy) [IEFPCM$_{(DCM)}$M06-2X/6-311++G(2d,2p)] = -1041.446106

C	0.31308500	1.12236100	-1.39452200
H	0.83281400	0.35115900	-1.95386300
C	-0.86093700	0.86959000	-0.77944900
N	-1.39572900	-0.40784800	-0.66351500
C	-2.82855300	-0.64772400	-0.51611600
H	-3.44098000	0.08196700	-1.05504800
C	-0.72084800	-1.54700500	-1.28371100
H	-0.62032600	-1.40166600	-2.37103900
H	0.28028600	-1.66165300	-0.85603300
C	-1.65375300	-2.71073100	-0.96899400
H	-1.51404800	-3.55551000	-1.64551000
H	-1.48719200	-3.05705800	0.05686200
C	-3.03413600	-2.06293700	-1.10566800
H	-3.29812000	-1.96511600	-2.16235000
H	-3.83641500	-2.60486600	-0.60176700
C	-1.58831600	1.97455900	-0.04839300
H	-2.65523200	1.97450900	-0.29765600
H	-1.51669000	1.80810400	1.03573000
C	1.16022100	0.30722000	1.47244800
H	0.81889800	1.35798200	1.47476000
C	2.64674800	0.10025200	1.27326600
O	0.40641100	-0.62496300	1.66925700
C	-3.29865900	-0.60915000	0.94096000
O	-2.37021800	-0.67500700	1.88929100
O	-4.47708200	-0.55934000	1.22115900
H	-1.45807500	-0.66394600	1.52091600
C	2.98736900	-0.97307400	0.25763700
H	2.61582400	-0.64961100	-0.72295800

C	4.48421200	-1.26951700	0.18654200
H	2.43102400	-1.87587300	0.53768600
C	4.79926600	-2.34289000	-0.85284800
H	5.87158000	-2.55037400	-0.89486900
H	4.28306800	-3.27887600	-0.61592500
H	4.47827300	-2.02575600	-1.85033200
H	4.83285400	-1.59383100	1.17462200
H	5.02498300	-0.34785100	-0.05416200
H	3.08379600	-0.13617300	2.25341500
C	-0.99590200	3.33192100	-0.40957100
C	0.97043800	2.47763600	-1.33369000
H	-1.29122200	3.61588400	-1.43088400
H	-1.34245700	4.10371600	0.27985700
H	2.03302100	2.37442200	-1.09505100
H	0.89269700	2.99879700	-2.30247000
F	3.18452500	1.32659100	0.87592600
O	0.41708800	3.29608300	-0.31595700

(*R*)-**TS3**$_{P}$-F

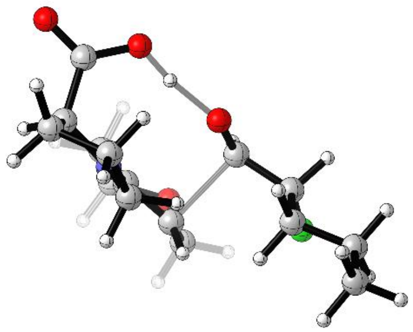

- Thermochemistry -

Zero-point correction= 0.386933 (Hartree/Particle)
Thermal correction to Energy= 0.404278

Thermal correction to Enthalpy= 0.405143
Thermal correction to Gibbs Free Energy= 0.343573
Sum of electronic and zero-point Energies= -1040.758510
Sum of electronic and thermal Energies= -1040.741164
Sum of electronic and thermal Enthalpies= -1040.740299
Sum of electronic and thermal Free Energies= -1040.801870

Number of Imaginary Frequencies = 1

E (Single Point Energy) [IEFPCM$_{(DCM)}$M06-2X/6-311++G(2d,2p)] = -1041.435017

C	-0.39683700	1.23415800	0.90330700
H	-1.05449900	0.60321600	1.49328300
C	0.92546100	0.84070500	0.72108700
N	1.30499500	-0.43982100	0.81333800
C	2.65207700	-0.93368100	0.51363100
H	3.41373100	-0.34224500	1.02688400
C	0.43637100	-1.50052600	1.35151900
H	0.36321200	-1.39006100	2.44146100
H	-0.55778800	-1.42834500	0.91411300
C	1.16318000	-2.77938600	0.95256600
H	0.90602800	-3.62055900	1.59790800
H	0.90524600	-3.03914000	-0.07966900
C	2.63563200	-2.37779800	1.05497700
H	2.95224200	-2.36665800	2.10174300
H	3.31475100	-3.02390800	0.49717100
C	1.92268100	1.83683000	0.18001000
H	2.91850700	1.65306600	0.59242800
H	1.99343500	1.70603800	-0.90889700
C	-0.94367900	0.51261500	-1.09509200
H	-0.62058400	1.48919300	-1.48687200
C	-2.45495000	0.34173200	-0.99059200
O	-0.29968200	-0.53919700	-1.37096800

C	3.03717500	-0.91072400	-0.97606600
O	2.10761000	-0.75934300	-1.88702700
O	4.21030000	-1.06168500	-1.26655300
H	1.11587400	-0.63184300	-1.58218200
C	-2.94723000	-0.82350200	-0.16020100
H	-2.72887200	-0.62759500	0.89735600
C	-4.44424600	-1.08102800	-0.33386300
H	-2.37426100	-1.70859600	-0.46047300
C	-4.92533000	-2.24227200	0.53312300
H	-5.99537500	-2.42110900	0.39910900
H	-4.39429400	-3.16536000	0.27862600
H	-4.74893100	-2.03577500	1.59386100
H	-4.65146400	-1.29465000	-1.38955200
H	-5.00055400	-0.17210000	-0.07958500
H	-2.79222200	0.22303600	-2.03136700
C	1.49696100	3.26646100	0.49464100
C	-0.72427700	2.71464600	0.93420000
H	1.62011600	3.47376400	1.56808200
H	2.10319000	3.97758200	-0.06847600
H	-1.72986100	2.89461700	0.55695100
H	-0.67552300	3.09091100	1.96924300
F	-3.04058600	1.52578900	-0.53246100
O	0.15055600	3.47444800	0.11931500

(*R*)-**TS3**$_{P}$-F-P

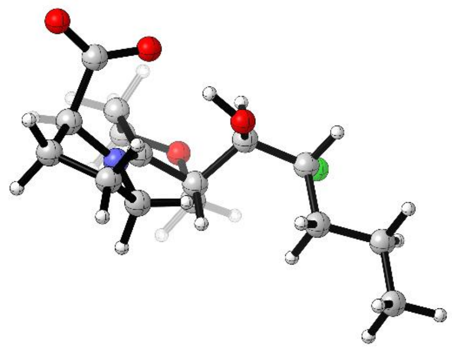

- Thermochemistry -

Zero-point correction=	0.391146 (Hartree/Particle)
Thermal correction to Energy=	0.408579
Thermal correction to Enthalpy=	0.409444
Thermal correction to Gibbs Free Energy=	0.347343
Sum of electronic and zero-point Energies=	-1040.786751
Sum of electronic and thermal Energies=	-1040.769318
Sum of electronic and thermal Enthalpies=	-1040.768453
Sum of electronic and thermal Free Energies=	-1040.830554

Number of Imaginary Frequencies = 0

E (Single Point Energy) [IEFPCM$_{(DCM)}$M06-2X/6-311++G(2d,2p)] = -1041.467264

C	0.59550100	0.67073000	-0.61610500
H	0.91915900	-0.15016900	-1.25912100
C	-0.90218500	0.73369500	-0.56103600
N	-1.62540700	-0.30817600	-0.79403700
C	-3.06168700	-0.40214900	-0.44997800
H	-3.60783600	0.44734200	-0.86040300
C	-1.10599700	-1.63686100	-1.23941300
H	-0.92172200	-1.57036600	-2.31597500
H	-0.18427800	-1.86676400	-0.70946800
C	-2.24625900	-2.59752300	-0.91346800

H	-2.23458300	-3.46598100	-1.57303300
H	-2.15397500	-2.94437300	0.12032200
C	-3.49432000	-1.72895400	-1.07393600
H	-3.73243600	-1.58827600	-2.13281400
H	-4.37359900	-2.12260500	-0.56412200
C	-1.49207700	2.02504900	-0.08027900
H	-2.55780000	2.09823600	-0.29724100
H	-1.38206700	2.03350900	1.01168100
C	1.05268800	0.37508900	0.85249800
H	0.82560200	1.25628900	1.46849700
C	2.55523200	0.10805600	0.98722000
O	0.42389900	-0.77995300	1.35091000
C	-3.23761900	-0.39752300	1.11225200
O	-2.19731900	-0.31903900	1.81920200
O	-4.41901700	-0.47049300	1.48070400
H	-0.52702300	-0.60449400	1.55963900
C	3.13506600	-0.89022100	0.00698600
H	3.12518200	-0.45427100	-1.00094600
C	4.55949500	-1.31449500	0.36408000
H	2.47503900	-1.76772600	-0.00703200
C	5.13540100	-2.29725300	-0.65269900
H	6.15129200	-2.59789300	-0.38343400
H	4.52072900	-3.20146600	-0.71233900
H	5.17050600	-1.84948200	-1.65132800
H	4.55712600	-1.76998700	1.36169000
H	5.19613100	-0.42471000	0.42367600
H	2.71712800	-0.22618600	2.01791500
C	-0.74575100	3.22581100	-0.67713900

C	1.16131200	1.98684700	-1.17796100
H	-0.96853500	3.31927300	-1.74964300
H	-1.06366500	4.13931900	-0.17411200
H	2.24488200	2.00834000	-1.06853300
H	0.91292800	2.06144900	-2.24677600
F	3.24085000	1.32755900	0.85490000
O	0.64858700	3.10553500	-0.48570000

(*S*)-**TS3**$_{P}$-F-Pre

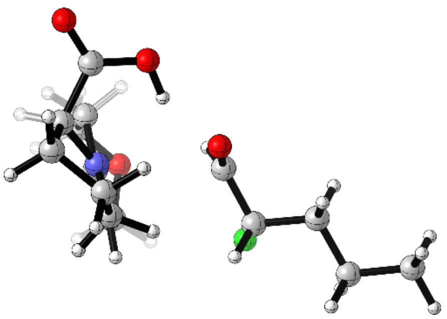

- Thermochemistry -

Zero-point correction=	0.386566 (Hartree/Particle)
Thermal correction to Energy=	0.405752
Thermal correction to Enthalpy=	0.406617
Thermal correction to Gibbs Free Energy=	0.338545
Sum of electronic and zero-point Energies=	-1040.767796
Sum of electronic and thermal Energies=	-1040.748610
Sum of electronic and thermal Enthalpies=	-1040.747745
Sum of electronic and thermal Free Energies=	-1040.815817

Number of Imaginary Frequencies = 0

E (Single Point Energy) [IEFPCM$_{(DCM)}$M06-2X/6-311++G(2d,2p)] = -1041.445581

C	0.22502800	1.57570100	1.27301800
H	-0.16960900	1.05985800	2.14304100
C	1.20251700	1.03252500	0.51718800

N	1.65779200	-0.26992000	0.68529800
C	3.00587400	-0.68078000	0.29893800
H	3.74566500	0.11596000	0.42367300
C	1.13971400	-1.09367500	1.77730800
H	1.31796500	-0.61241100	2.75181500
H	0.06169400	-1.23886800	1.65518900
C	1.94195200	-2.38294200	1.64514600
H	1.95954900	-2.96265200	2.56948100
H	1.51742000	-3.00795000	0.85201100
C	3.32462600	-1.86223700	1.24472100
H	3.84678300	-1.47395200	2.12355500
H	3.96456800	-2.60741900	0.76894400
C	1.74601000	1.78616800	-0.67502800
H	2.84000600	1.73996400	-0.70694800
H	1.38045200	1.31588300	-1.59882800
C	-1.33649400	-0.01197900	-0.75658300
H	-1.10704200	0.95672500	-1.23639900
C	-2.59304500	-0.05995500	0.08517900
O	-0.66025600	-1.00598100	-0.93371100
C	3.10353500	-1.13011300	-1.16181100
O	1.96899000	-1.38587800	-1.80449500
O	4.17752400	-1.28376700	-1.70251200
H	1.17879600	-1.17459900	-1.25927200
C	-3.66895700	-0.90988800	-0.57355700
H	-3.24495800	-1.90860900	-0.73091900
C	-4.94214300	-0.99722000	0.26630800
H	-3.89327800	-0.49035800	-1.56265400
C	-6.00999500	-1.85053400	-0.41459000

H	-6.91563800	-1.90984300	0.19455300
H	-6.28442900	-1.42807900	-1.38645100
H	-5.64762900	-2.86985500	-0.58216500
H	-5.32920200	0.01169100	0.44345900
H	-4.69552100	-1.42061900	1.24774200
H	-2.35154400	-0.43401000	1.08741100
C	1.31372400	3.24691800	-0.62802900
C	-0.36927800	2.92521900	0.95657500
H	1.87520500	3.78478400	0.15072600
H	1.49347000	3.73623500	-1.58686500
H	-1.45927700	2.88660300	1.02761700
H	-0.01176000	3.68796000	1.66844500
O	-0.07454600	3.34496400	-0.36565200
F	-3.05203800	1.25056200	0.22978000

(*S*)-**TS3**P-F

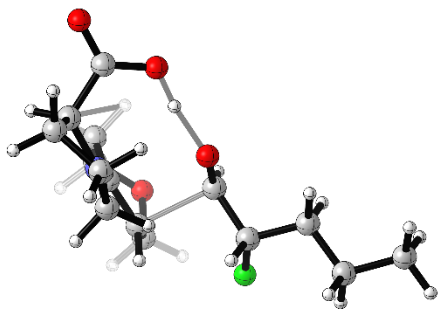

- Thermochemistry -

Zero-point correction= 0.386472 (Hartree/Particle)
Thermal correction to Energy= 0.404048
Thermal correction to Enthalpy= 0.404913
Thermal correction to Gibbs Free Energy= 0.342145
Sum of electronic and zero-point Energies= -1040.759717
Sum of electronic and thermal Energies= -1040.742141
Sum of electronic and thermal Enthalpies= -1040.741276

Sum of electronic and thermal Free Energies= -1040.804044

Number of Imaginary Frequencies = 1

E (Single Point Energy) [IEFPCM$_{(DCM)}$M06-2X/6-311++G(2d,2p)] = -1041.435820

C	-0.07609300	1.41960400	0.97632600
H	-0.48006600	0.96941400	1.87851800
C	1.16676700	1.00587200	0.51150400
N	1.66858700	-0.20051700	0.80752300
C	2.91790100	-0.73957800	0.26387300
H	3.73478500	-0.02033200	0.35810000
C	1.08439300	-1.08682600	1.82966500
H	1.30617600	-0.68507100	2.82710300
H	0.00534700	-1.15196200	1.69871500
C	1.78753500	-2.41530300	1.57764700
H	1.79768400	-3.05311800	2.46261700
H	1.27939300	-2.94896000	0.76761300
C	3.18705200	-1.97968300	1.14012500
H	3.77816600	-1.67711100	2.00912800
H	3.74309400	-2.74375900	0.59530400
C	1.87622200	1.82760200	-0.53733900
H	2.95981400	1.78062600	-0.39820600
H	1.65669100	1.40264800	-1.52653600
C	-1.12806700	0.14213000	-0.45258600
H	-1.12680500	0.98721400	-1.15956300
C	-2.42718700	-0.08882300	0.30599200
O	-0.46681100	-0.90629100	-0.69223200
C	2.86894200	-1.12170000	-1.22615100
O	1.71295300	-1.22639200	-1.83622700
O	3.92186400	-1.34153500	-1.79660000

H	0.84700800	-1.02869000	-1.29793300
C	-3.43996500	-0.78302000	-0.59246400
H	-2.98479500	-1.71576100	-0.94356100
C	-4.76012300	-1.07273500	0.12098400
H	-3.61547800	-0.15245700	-1.47445600
C	-5.75778300	-1.77472900	-0.79806600
H	-6.69781500	-1.98055800	-0.27927100
H	-5.98388300	-1.15532400	-1.67202800
H	-5.35465300	-2.72684500	-1.15802400
H	-5.18926600	-0.13446900	0.48784600
H	-4.56215100	-1.69712600	1.00120600
H	-2.23223200	-0.68403900	1.20555100
C	1.41799700	3.28033300	-0.49385100
C	-0.53774800	2.83573100	0.69530400
H	1.80693700	3.77691600	0.40761600
H	1.77668000	3.82099500	-1.37097200
H	-1.62101000	2.86935000	0.57989400
H	-0.26398800	3.49830400	1.53255400
O	0.00661100	3.35010900	-0.50689100
F	-2.97063500	1.12815100	0.72732700

(*S*)-**TS3**$_{P}$-F-P

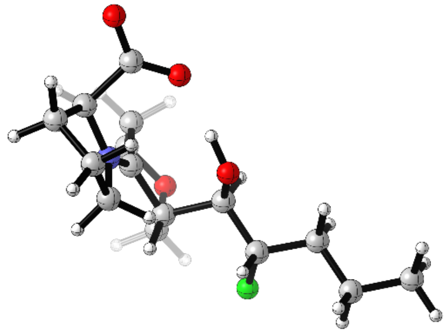

- Thermochemistry -

Zero-point correction=	0.390783 (Hartree/Particle)
Thermal correction to Energy=	0.408314
Thermal correction to Enthalpy=	0.409179
Thermal correction to Gibbs Free Energy=	0.346494
Sum of electronic and zero-point Energies=	-1040.786391
Sum of electronic and thermal Energies=	-1040.768859
Sum of electronic and thermal Enthalpies=	-1040.767994
Sum of electronic and thermal Free Energies=	-1040.830680

Number of Imaginary Frequencies = 0

E (Single Point Energy) [IEFPCM$_{(DCM)}$M06-2X/6-311++G(2d,2p)] = -1041.466319

C	-0.31482100	1.05173300	0.77221100
H	-0.40618700	0.66007500	1.78941000
C	1.09889500	0.91818900	0.27981400
N	1.87758500	-0.00562000	0.73044000
C	3.17430300	-0.36890800	0.11434800
H	3.79168400	0.52079200	-0.01384000
C	1.52212000	-0.97376600	1.81065800
H	1.63374800	-0.45409000	2.76716200
H	0.49565000	-1.30743300	1.67663400
C	2.54875900	-2.09049100	1.64472000
H	2.72688200	-2.60973000	2.58706000

H	2.18981000	-2.81677200	0.90891200
C	3.77947800	-1.35679700	1.11153400
H	4.28567200	-0.81771400	1.91836100
H	4.50005100	-2.00602500	0.61454000
C	1.50172800	1.80768900	-0.85756800
H	2.58210900	1.83367100	-0.99937800
H	1.06739700	1.36510700	-1.76365500
C	-1.19820000	0.18026600	-0.19066600
H	-1.38165100	0.76916400	-1.10248000
C	-2.53315200	-0.20698500	0.45460700
O	-0.60823400	-1.06194200	-0.48364100
C	2.94799300	-1.01443300	-1.30111300
O	1.76728300	-1.08199600	-1.73489700
O	3.99528300	-1.38483400	-1.85153900
H	0.23258900	-0.99188100	-1.00100800
C	-3.51582900	-0.80229000	-0.53191900
H	-3.01210300	-1.62597400	-1.04903500
C	-4.79874400	-1.30118600	0.13195800
H	-3.75194200	-0.03820400	-1.28471300
C	-5.77741700	-1.88289700	-0.88606200
H	-6.68986800	-2.24046600	-0.40144500
H	-6.06275800	-1.12848100	-1.62650000
H	-5.32764800	-2.72447500	-1.42287000
H	-5.27385500	-0.47563300	0.67310400
H	-4.54357700	-2.06399000	0.87822600
H	-2.32387100	-0.90458300	1.27631500
C	0.94765900	3.22370200	-0.67808200
C	-0.72349100	2.53965400	0.79149700

H	1.45895700	3.73802900	0.14824000
H	1.10076000	3.79574600	-1.59359900
H	-1.79314500	2.63069300	0.96233800
H	-0.19000200	3.04661100	1.60858500
O	-0.44154100	3.18238400	-0.43351500
F	-3.12331200	0.92390500	1.04107400

(*R*)-**TS3**$_{G}$-F-Pre

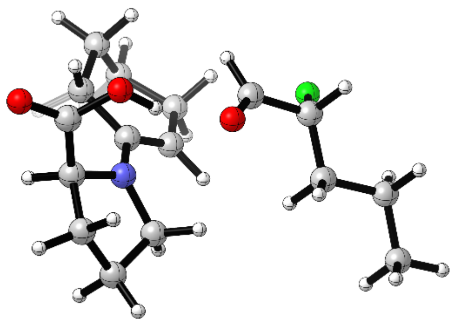

- Thermochemistry -

Zero-point correction=	0.410012 (Hartree/Particle)
Thermal correction to Energy=	0.429436
Thermal correction to Enthalpy=	0.430301
Thermal correction to Gibbs Free Energy=	0.362756
Sum of electronic and zero-point Energies=	-1004.852010
Sum of electronic and thermal Energies=	-1004.832586
Sum of electronic and thermal Enthalpies=	-1004.831721
Sum of electronic and thermal Free Energies=	-1004.899267

Number of Imaginary Frequencies = 0

E (Single Point Energy) [IEFPCM$_{(DCM)}$M06-2X/6-311++G(2d,2p)] = -1005.539197

C	0.20753400	1.35316400	-1.00903900
H	1.00020400	0.68466100	-1.33125400
C	-0.99067800	0.84123300	-0.64086200
N	-1.25082100	-0.52815900	-0.66054000

C	-2.57606900	-1.08903600	-0.43246600
H	-3.35164000	-0.62825700	-1.05784400
C	-0.40858000	-1.43007700	-1.45792200
H	0.41936000	-1.81958100	-0.85152000
H	0.01674400	-0.88682800	-2.30859200
C	-1.36301900	-2.53606000	-1.91134900
H	-0.85873000	-3.49310600	-2.05744800
H	-1.84036500	-2.24952100	-2.85372600
C	-2.40317300	-2.57828600	-0.79468400
H	-3.35248500	-3.03177900	-1.08254300
H	-2.00501600	-3.12291800	0.06930100
C	-2.11873900	1.73249100	-0.16077600
H	-2.91465200	1.73230700	-0.91815000
H	-2.56384800	1.32344000	0.75226500
C	-0.75562800	3.67702700	-0.99545700
C	1.17098200	0.16623100	1.55932400
H	0.68025500	1.12824700	1.78312600
C	2.65895300	0.22947000	1.28383100
O	0.57337800	-0.89130900	1.60943700
C	-3.06253200	-1.00128100	1.00945300
O	-2.13755400	-0.96503600	1.96399600
O	-4.24298300	-1.02697800	1.28490300
C	3.15672500	-0.77834600	0.26835000
H	2.79306500	-1.76422300	0.58117100
C	4.67919000	-0.78554800	0.14082200
H	5.02480400	0.21786700	-0.13038000
H	5.12028100	-1.02007500	1.11738700
H	2.69620000	-0.55774600	-0.70252400

C	5.15572500	-1.79656600	-0.89953100
H	6.24573400	-1.79678400	-0.98090000
H	4.83642400	-2.80969000	-0.63453400
H	4.74443200	-1.56273700	-1.88682200
H	-1.21660500	-0.93063000	1.61200300
H	3.16721700	0.08851200	2.24933400
C	-1.66264000	3.16571200	0.11932300
H	-2.54158800	3.80523300	0.24380700
H	-1.11014100	3.19177200	1.06808800
C	0.51459500	2.83038900	-1.04326600
H	1.16522800	3.09899300	-0.19786000
H	1.08948100	3.05903700	-1.94813800
H	-1.28488800	3.60445100	-1.95490500
H	-0.50572000	4.73118700	-0.83939000
F	2.94926700	1.52986300	0.86696000

(*R*)-**TS3**$_{G}$-F

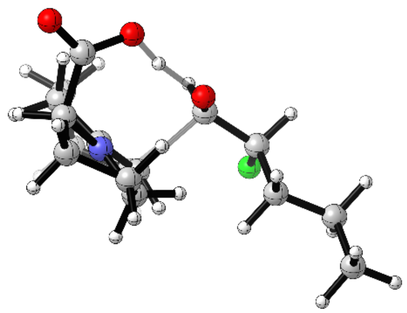

- Thermochemistry -

Zero-point correction= 0.410619 (Hartree/Particle)
Thermal correction to Energy= 0.428127
Thermal correction to Enthalpy= 0.428992
Thermal correction to Gibbs Free Energy= 0.367347
Sum of electronic and zero-point Energies= -1004.845557
Sum of electronic and thermal Energies= -1004.828049

Sum of electronic and thermal Enthalpies= -1004.827184
Sum of electronic and thermal Free Energies= -1004.888828

Number of Imaginary Frequencies = 1

E (Single Point Energy) [IEFPCM$_{(DCM)}$M06-2X/6-311++G(2d,2p)] = -1005.531517

C	0.41919200	1.27925000	-0.78392400
H	1.05812500	0.60641500	-1.34656300
C	-0.90915100	0.89944000	-0.61229000
N	-1.29631200	-0.37779500	-0.76060000
C	-2.65349300	-0.86379000	-0.50607000
H	-3.40258500	-0.17207000	-0.89882400
C	-0.44912200	-1.41363900	-1.37817200
H	0.52319400	-1.44929800	-0.88940600
H	-0.31218700	-1.17731300	-2.44204300
C	-1.25209400	-2.69471600	-1.17460700
H	-1.04216300	-3.10709900	-0.18169800
H	-1.01369400	-3.45371200	-1.92108400
C	-2.69972700	-2.20723100	-1.25513600
H	-2.98062500	-2.02390200	-2.29635600
H	-3.42775300	-2.88868900	-0.81363700
C	-1.95457100	1.87199100	-0.11362200
H	-2.73206100	1.93698800	-0.88654500
H	-2.44102000	1.46487500	0.78069200
C	-0.40138200	3.67434500	-0.89969400
C	0.96666500	0.45094300	1.15972700
H	0.65543000	1.39972400	1.62322100
C	2.47784800	0.29375100	1.01090000
O	0.33272700	-0.61637100	1.39463200
C	-3.00393600	-1.05387600	0.98205100

O	-2.06919500	-0.93322800	1.89243700
O	-4.15754100	-1.31824200	1.27076600
C	2.94928500	-0.86997000	0.16603000
H	2.39044100	-1.75826300	0.48195000
C	4.45250600	-1.11846500	0.29398000
H	4.99564800	-0.20696900	0.02099100
H	4.69350800	-1.32873800	1.34324300
H	2.69843100	-0.67535400	-0.88461100
C	4.91373300	-2.27894100	-0.58478200
H	5.98836800	-2.45168800	-0.48302400
H	4.39571500	-3.20434100	-0.31243000
H	4.70394100	-2.07579600	-1.64006400
H	-1.08792800	-0.74364400	1.58942300
H	2.84816800	0.17674000	2.04077000
C	-1.40647100	3.27048100	0.17347800
H	-2.24286600	3.97252200	0.23000700
H	-0.91169600	3.28557200	1.15281200
C	0.80977700	2.74802400	-0.80976500
H	1.37405700	3.00786900	0.09463300
H	1.49287400	2.91760900	-1.64792800
H	-0.86959000	3.59593000	-1.88984800
H	-0.08867000	4.71434700	-0.76658100
F	3.04520400	1.47777800	0.53286600

(*R*)-**TS3**$_G$-F-P

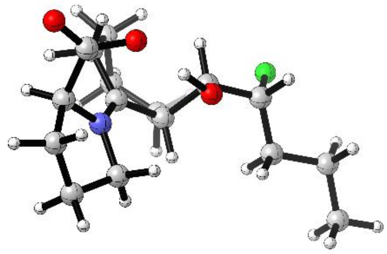

- Thermochemistry -

Zero-point correction= 0.414601 (Hartree/Particle)
Thermal correction to Energy= 0.432413
Thermal correction to Enthalpy= 0.433278
Thermal correction to Gibbs Free Energy= 0.370408
Sum of electronic and zero-point Energies= -1004.870016
Sum of electronic and thermal Energies= -1004.852205
Sum of electronic and thermal Enthalpies= -1004.851340
Sum of electronic and thermal Free Energies= -1004.914209

Number of Imaginary Frequencies = 0

E (Single Point Energy) [IEFPCM$_{(DCM)}$M06-2X/6-311++G(2d,2p)] = -1005.559390

C	0.60595200	0.52829800	-0.62900800
H	0.87852100	-0.39134200	-1.14715700
C	-0.88410500	0.64334100	-0.54477500
N	-1.64181600	-0.39129100	-0.71194400
C	-3.05538000	-0.43565200	-0.27051000
H	-3.61761500	0.34684500	-0.78583700
C	-1.22568400	-1.71138900	-1.25233500
H	-0.79764300	-2.29522900	-0.43193600
H	-0.48176900	-1.57601900	-2.03567800
C	-2.54508000	-2.28774800	-1.74987500
H	-2.49385100	-3.37176000	-1.86026200
H	-2.79928900	-1.84893600	-2.71954400
C	-3.53683700	-1.84237300	-0.67496000

H	-4.57100300	-1.82102600	-1.01785600
H	-3.47756400	-2.51188400	0.18902700
C	-1.48416400	1.95061000	-0.10566900
H	-2.39206400	2.11608900	-0.69706900
H	-1.80803900	1.84583200	0.93718400
C	0.32220800	2.92104400	-1.53594800
C	1.02221700	0.29043500	0.87059800
H	0.74359100	1.17099400	1.46881600
C	2.52948300	0.07545900	1.06622400
O	0.41474400	-0.87620200	1.36142000
C	-3.18443500	-0.24014000	1.27074400
O	-2.13768500	-0.36342900	1.96466000
O	-4.34282800	-0.02688900	1.65891800
C	3.20079200	-0.82731700	0.05325700
H	2.59414800	-1.73850400	-0.03480400
C	4.63395500	-1.19119500	0.44002400
H	5.21776200	-0.27263200	0.56556100
H	4.62433100	-1.69573000	1.41381200
H	3.19633700	-0.33316400	-0.92770300
C	5.29730400	-2.08903600	-0.60176200
H	6.31985000	-2.34535300	-0.31239300
H	4.73793700	-3.02221900	-0.72557500
H	5.33807600	-1.59119300	-1.57615000
H	-0.53359200	-0.70362500	1.59788400
H	2.64711600	-0.32607200	2.07853800
C	-0.52482800	3.13430800	-0.28532700
H	-1.11483000	4.05152700	-0.34933100
H	0.13081000	3.23836600	0.58693400

C	1.27978900	1.74695200	-1.31274400
H	2.11317800	2.08612600	-0.69408400
H	1.70400600	1.42017500	-2.26610400
H	-0.33942700	2.71902800	-2.38932700
H	0.89600400	3.81841600	-1.78111800
F	3.17741800	1.32321200	1.06557600

(*S*)-**TS3**$_{G}$-F-Pre

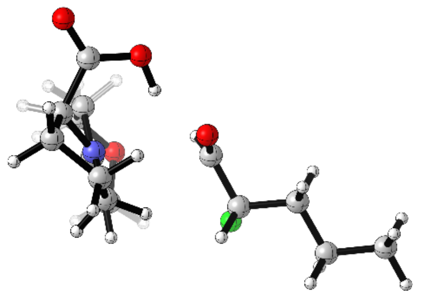

- Thermochemistry -

Zero-point correction=	0.410464 (Hartree/Particle)
Thermal correction to Energy=	0.429723
Thermal correction to Enthalpy=	0.430588
Thermal correction to Gibbs Free Energy=	0.363097
Sum of electronic and zero-point Energies=	-1004.855344
Sum of electronic and thermal Energies=	-1004.836085
Sum of electronic and thermal Enthalpies=	-1004.835220
Sum of electronic and thermal Free Energies=	-1004.902711

Number of Imaginary Frequencies = 0

E (Single Point Energy) [IEFPCM$_{(DCM)}$M06-2X/6-311++G(2d,2p)] = -1005.542809

C	0.11288900	1.51187000	1.31815800
H	-0.13540900	0.91451600	2.19109800
C	1.07772700	1.08682900	0.47304100
N	1.75351900	-0.12242200	0.65872400
C	3.06580500	-0.36920800	0.06578400

H	3.63914100	0.54648700	-0.10230800
C	1.58288900	-0.86730400	1.90772100
H	0.55696000	-1.23965300	1.98873900
H	1.78527500	-0.21696000	2.77375200
C	2.61751500	-1.98255200	1.79858000
H	2.22668000	-2.80243600	1.18561300
H	2.89769800	-2.38782300	2.77234000
C	3.77822900	-1.28102500	1.08911200
H	4.32377000	-0.65031700	1.79675100
H	4.48967300	-1.95858500	0.61322000
C	1.43803600	1.86038600	-0.77934900
H	2.41222300	2.34760500	-0.63991700
H	1.56218400	1.16283800	-1.61851100
C	0.00420100	3.71989500	0.10819600
C	-1.18050400	-0.59072400	-0.61991500
H	-0.73957200	0.05534400	-1.40273300
C	-2.52703900	-0.15191500	-0.08818700
O	-0.63478000	-1.60942000	-0.24977400
C	2.97858700	-1.07477600	-1.29060300
O	1.82725300	-1.66995000	-1.59328100
O	3.92505900	-1.12410900	-2.04611800
H	1.15138100	-1.53534100	-0.89111700
C	-3.60042000	-1.20560800	-0.30228800
H	-3.24995800	-2.12795800	0.17553000
H	-2.42717700	0.10618400	0.97362600
C	-4.95377800	-0.78806100	0.27006600
H	-5.26830400	0.15316200	-0.19307400
H	-3.68647800	-1.40791500	-1.37730100

H	-4.84387500	-0.59283900	1.34389700
C	-6.01844600	-1.85895300	0.04283900
H	-6.98198000	-1.55221400	0.45789900
H	-6.15719800	-2.04936200	-1.02619300
H	-5.73088700	-2.80278600	0.51712200
C	0.38735300	2.91484900	-1.13107800
C	-0.64997700	2.80164900	1.14038900
H	0.77506600	3.56418100	-1.92160500
H	-0.51184400	2.42477300	-1.52782500
H	0.90842500	4.17132700	0.53745600
H	-0.67371900	4.53757700	-0.15542000
H	-1.68458900	2.58770900	0.83274700
H	-0.72274500	3.31272400	2.10741600
F	-2.86755900	1.01916000	-0.76929800

(*S*)-**TS3**$_{G}$-F

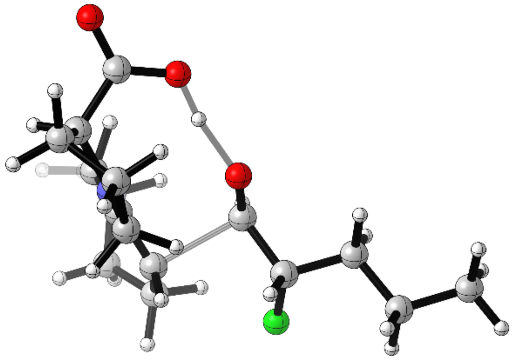

- Thermochemistry -

Zero-point correction= 0.410416 (Hartree/Particle)
Thermal correction to Energy= 0.428066
Thermal correction to Enthalpy= 0.428931
Thermal correction to Gibbs Free Energy= 0.366469
Sum of electronic and zero-point Energies= -1004.846572
Sum of electronic and thermal Energies= -1004.828922

Sum of electronic and thermal Enthalpies= -1004.828057
Sum of electronic and thermal Free Energies= -1004.890519

Number of Imaginary Frequencies = 1

E (Single Point Energy) [$IEFPCM_{(DCM)}$M06-2X/6-311++G(2d,2p)] = -1005.532406

C	-0.12518000	1.41565400	0.88284200
H	-0.49712100	0.91313700	1.77049300
C	1.12333800	1.02849500	0.40612100
N	1.66413200	-0.15206900	0.74797400
C	2.93126300	-0.66257900	0.22262500
H	3.68748500	0.12497600	0.17661000
C	1.14345300	-0.99420100	1.84085000
H	0.07904100	-1.17962400	1.70388500
H	1.30289600	-0.47918800	2.79762700
C	1.97650600	-2.26739500	1.73135000
H	1.52945600	-2.93755700	0.98922400
H	2.04319800	-2.79931700	2.68148800
C	3.32821200	-1.74897500	1.23781900
H	3.87836000	-1.28246600	2.06017100
H	3.96254600	-2.51116900	0.78384100
C	1.86465800	1.84248300	-0.63047100
H	2.81710900	2.15214000	-0.17947500
H	2.11988400	1.20802800	-1.48719000
C	0.41421800	3.75852400	0.08075900
C	-1.14392900	0.05753300	-0.47555000
H	-1.15701300	0.84648400	-1.24467600
C	-2.44206900	-0.12441000	0.29904200
O	-0.48555700	-1.00623400	-0.65205600
C	2.85282200	-1.24627300	-1.20100300

O	1.68690700	-1.39863200	-1.78064400
O	3.89340000	-1.55650100	-1.75278200
H	0.82653500	-1.15208700	-1.24927500
C	-3.46938700	-0.84267600	-0.56298000
H	-3.02555500	-1.79140900	-0.88480100
H	-2.24214600	-0.68779500	1.21811700
C	-4.78465900	-1.09587300	0.17309400
H	-5.20250300	-0.14146500	0.51022100
H	-3.64940700	-0.24308700	-1.46558200
H	-4.58267300	-1.68975900	1.07325900
C	-5.79728100	-1.82235600	-0.70987200
H	-6.73368000	-2.00140300	-0.17478400
H	-6.02728500	-1.23324700	-1.60357700
H	-5.40543200	-2.79023900	-1.03892700
C	1.09745500	3.07879100	-1.10099700
C	-0.65224000	2.81997300	0.64182500
H	1.79182800	3.75342200	-1.60928700
H	0.33772700	2.78886100	-1.83794100
H	1.16090700	3.99018500	0.85180800
H	-0.04255000	4.70400100	-0.22668200
H	-1.49495300	2.79242800	-0.06148400
H	-1.05952600	3.21090400	1.57935600
F	-2.96968200	1.11226300	0.67809900

(*S*)-**TS3**$_{G}$-F-P

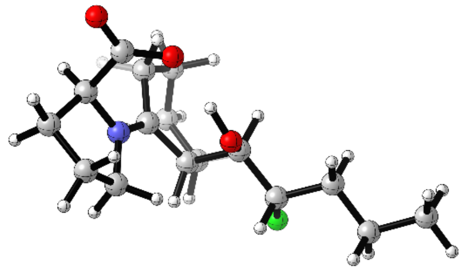

- Thermochemistry -

Zero-point correction=	0.414296 (Hartree/Particle)
Thermal correction to Energy=	0.432052
Thermal correction to Enthalpy=	0.432917
Thermal correction to Gibbs Free Energy=	0.369905
Sum of electronic and zero-point Energies=	-1004.871330
Sum of electronic and thermal Energies=	-1004.853574
Sum of electronic and thermal Enthalpies=	-1004.852709
Sum of electronic and thermal Free Energies=	-1004.915721

Number of Imaginary Frequencies = 0

E (Single Point Energy) [IEFPCM$_{(DCM)}$M06-2X/6-311++G(2d,2p)] = -1005.560329

C	-0.32904200	1.08728600	0.70014900
H	-0.38890100	0.70942000	1.72397300
C	1.08415500	0.95009300	0.21182200
N	1.84398700	0.01714100	0.68640400
C	3.13573000	-0.36889900	0.08382300
H	3.72671700	0.51910400	-0.14534900
C	1.50324900	-0.89534400	1.81947300
H	0.49439700	-1.27941100	1.69125900
H	1.57449200	-0.30931300	2.74125800
C	2.57651800	-1.97899300	1.74818700
H	2.25168500	-2.77888800	1.07550500
H	2.77097900	-2.41089800	2.73061300

C	3.78065900	-1.24566400	1.15597100
H	4.26299300	-0.62100000	1.91436800
H	4.52673700	-1.90557500	0.71375800
C	1.59722700	1.81555700	-0.90764600
H	2.58550600	2.18363400	-0.60666200
H	1.75525700	1.17537000	-1.78374500
C	0.12305900	3.55715700	0.08571700
C	-1.18163000	0.11647100	-0.20668000
H	-1.35050200	0.62740300	-1.16826000
C	-2.52607400	-0.21744000	0.45414800
O	-0.59697600	-1.14321900	-0.39502400
C	2.90808700	-1.15291000	-1.26110200
O	1.72592300	-1.27522800	-1.68158100
O	3.95484900	-1.56630800	-1.78028300
H	0.24080300	-1.11516300	-0.92667700
C	-3.52939000	-0.80306200	-0.51726600
H	-3.03988500	-1.63051200	-1.04290400
H	-2.32001900	-0.91119900	1.27982000
C	-4.80743200	-1.29229000	0.16247400
H	-5.26598000	-0.46419000	0.71408900
H	-3.76925100	-0.03579400	-1.26590000
H	-4.54889300	-2.06064500	0.90175200
C	-5.80673000	-1.85985400	-0.84343400
H	-6.71533400	-2.21112500	-0.34712000
H	-6.09579700	-1.09938500	-1.57625500
H	-5.37295200	-2.70314500	-1.39065900
C	0.69433000	3.01055100	-1.21777300
C	-0.86936400	2.54364300	0.65211100

H	1.28216900	3.76034700	-1.75250100
H	-0.12647100	2.71453600	-1.88178300
H	0.94050500	3.74535200	0.79517400
H	-0.38792500	4.51019300	-0.07526700
H	-1.76376600	2.56201800	0.02280000
H	-1.19326300	2.83167000	1.65431000
F	-3.08574300	0.92860400	1.03720400

(*R*)-**TS3**$_{O}$-F-Pre

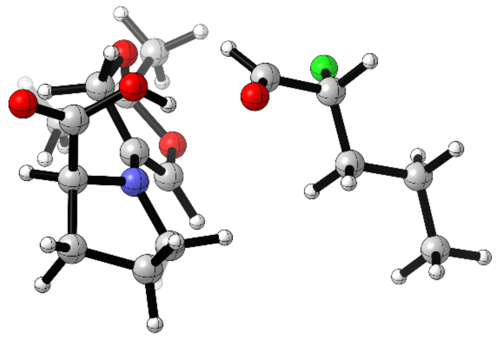

- Thermochemistry -

Zero-point correction= 0.417855 (Hartree/Particle)
Thermal correction to Energy= 0.439184
Thermal correction to Enthalpy= 0.440049
Thermal correction to Gibbs Free Energy= 0.367878
Sum of electronic and zero-point Energies= -1155.233150
Sum of electronic and thermal Energies= -1155.211821
Sum of electronic and thermal Enthalpies= -1155.210956
Sum of electronic and thermal Free Energies= -1155.283126

Number of Imaginary Frequencies = 0

E (Single Point Energy) [IEFPCM$_{(DCM)}$M06-2X/6-311++G(2d,2p)] = -1155.975514

C	-0.33927800	0.88358900	1.20561900
H	-0.77088400	0.19694300	1.92290800
C	0.78360700	0.64355600	0.50633000

N	1.49199900	-0.56120900	0.56771500
C	2.95122600	-0.55650100	0.43964900
H	3.40150000	0.40147000	0.72129100
C	1.05220700	-1.58922700	1.51146500
H	0.08903900	-2.00051400	1.19033400
H	0.93394600	-1.17001000	2.52412400
C	2.19526000	-2.59710800	1.47835900
H	2.11489700	-3.23044400	0.58835300
H	2.21116400	-3.23992100	2.35993400
C	3.42093800	-1.68333300	1.38763200
H	3.63646500	-1.24991000	2.36816700
H	4.32404300	-2.17612300	1.02362300
C	1.23623600	1.65811900	-0.51432900
H	2.14071900	2.19452800	-0.19211300
H	1.46472100	1.16290300	-1.46502100
O	-1.09217100	2.02266300	1.05949000
O	0.18719400	2.56897000	-0.79814600
C	-0.45376500	3.08757000	0.35350200
C	-1.55834900	3.99282800	-0.14497300
H	-2.19898100	3.43598600	-0.83238500
H	-1.12668800	4.85145000	-0.66356500
C	0.52457400	3.80153800	1.28086900
H	1.09361500	4.54235400	0.71327100
H	1.21772600	3.10106000	1.75264800
H	-0.03487700	4.31002300	2.06880500
C	-1.12846500	-0.55550600	-1.58944000
H	-0.86035700	0.50142600	-1.77298800
C	-2.59829800	-0.82905100	-1.35410000

O	-0.30780600	-1.44991000	-1.60717700
C	3.40862800	-0.85056500	-0.99331800
O	2.48545400	-1.27634000	-1.84918300
O	4.56781200	-0.72998100	-1.32563200
H	-2.15441100	4.34539500	0.69909800
C	-2.86638400	-1.62036600	-0.08678800
H	-2.23999800	-2.51984000	-0.12492500
C	-4.33723100	-2.00013300	0.07363900
H	-4.94816500	-1.09114000	0.06699300
H	-4.64919500	-2.59964200	-0.79028600
H	-2.53251200	-1.02288500	0.77066000
C	-4.58209300	-2.78044100	1.36315400
H	-5.63592300	-3.05109500	1.46763100
H	-3.99304200	-3.70315700	1.37901400
H	-4.29877000	-2.18596300	2.23770700
H	1.59100200	-1.28733100	-1.43764700
H	-2.99063800	-1.35549000	-2.23467100
F	-3.24501500	0.40615100	-1.29482200

(*R*)-**TS3**$_{O}$-F

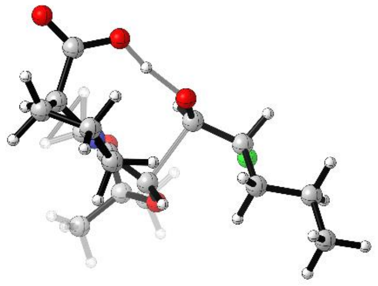

- Thermochemistry -

Zero-point correction= 0.416872 (Hartree/Particle)

Thermal correction to Energy= 0.436577
Thermal correction to Enthalpy= 0.437442
Thermal correction to Gibbs Free Energy= 0.370379
Sum of electronic and zero-point Energies= -1155.220110
Sum of electronic and thermal Energies= -1155.200405
Sum of electronic and thermal Enthalpies= -1155.199540
Sum of electronic and thermal Free Energies= -1155.266603

Number of Imaginary Frequencies = 1

E (Single Point Energy) [IEFPCM$_{(DCM)}$M06-2X/6-311++G(2d,2p)] = -1155.960428

C	-0.58837000	0.53064500	0.70857300
H	-0.95541000	-0.23292700	1.38400600
C	0.77547700	0.67331200	0.48910900
N	1.63781200	-0.31175000	0.75123800
C	3.04938200	-0.28378100	0.36534400
H	3.48325500	0.70695900	0.52787200
C	1.31078100	-1.47136500	1.60583800
H	0.41350200	-1.97228600	1.24214600
H	1.14441800	-1.11796200	2.63186600
C	2.55705100	-2.34633500	1.49139700
H	2.47503200	-2.99357700	0.61192500
H	2.69640100	-2.97644300	2.37071600
C	3.68356800	-1.32742800	1.30080500
H	3.93007100	-0.84939300	2.25321300
H	4.59543200	-1.74602400	0.87392500
C	1.25310800	1.89677100	-0.25067100
H	1.95180400	2.46945600	0.37611800
H	1.77870600	1.61088100	-1.16889900
O	-1.45191000	1.60055200	0.60498600
O	0.15631300	2.68408200	-0.66083100
C	-0.84115800	2.86138400	0.33149900

C	-1.91113600	3.73030000	-0.28982400
H	-2.24362000	3.27662700	-1.22564600
H	-1.51042600	4.72684800	-0.48670000
C	-0.27231000	3.44913400	1.61964400
H	0.30287500	4.35031100	1.39171800
H	0.37177400	2.74012900	2.14616400
H	-1.09735000	3.71255500	2.28429700
C	-0.75155600	-0.51822100	-1.12881500
H	-0.52522400	0.38481900	-1.71935800
C	-2.23701400	-0.86746500	-1.13576000
O	0.05204100	-1.50446200	-1.12679900
C	3.31764000	-0.63352300	-1.11261100
O	2.33999400	-1.07167300	-1.85914600
O	4.45337400	-0.48920500	-1.53194200
H	-2.75924100	3.81160800	0.39278500
C	-2.79727200	-1.57150900	0.08267300
H	-2.05193000	-2.30098900	0.42648500
C	-4.12671200	-2.27134000	-0.19907700
H	-4.83555800	-1.54284500	-0.60914500
H	-3.97457600	-3.03506500	-0.97127100
H	-2.93600600	-0.82852900	0.87714600
C	-4.71203900	-2.91272800	1.05665700
H	-5.65851100	-3.41557900	0.84161500
H	-4.02287900	-3.65479400	1.47316300
H	-4.89887100	-2.15874400	1.82823600
H	1.36295800	-1.22835500	-1.44634600
H	-2.35092900	-1.50890800	-2.02201200
F	-2.97839000	0.28589100	-1.39572500

(*R*)-**TS3**$_O$-F-P

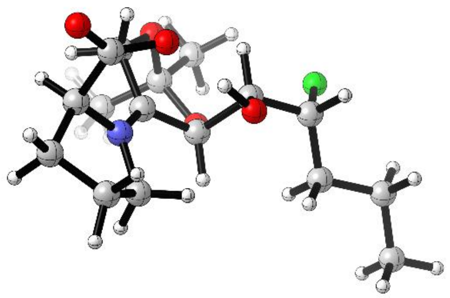

- Thermochemistry -

Zero-point correction=	0.421605 (Hartree/Particle)
Thermal correction to Energy=	0.441332
Thermal correction to Enthalpy=	0.442197
Thermal correction to Gibbs Free Energy=	0.375586
Sum of electronic and zero-point Energies=	-1155.245176
Sum of electronic and thermal Energies=	-1155.225449
Sum of electronic and thermal Enthalpies=	-1155.224584
Sum of electronic and thermal Free Energies=	-1155.291196

Number of Imaginary Frequencies = 0

E (Single Point Energy) [IEFPCM$_{(DCM)}$M06-2X/6-311++G(2d,2p)] = -1155.989945

C	-0.55515500	0.22780100	0.44184300
H	-0.74068000	-0.51153300	1.22456300
C	0.90513000	0.56374700	0.35529200
N	1.82011800	-0.24814000	0.74339200
C	3.23231400	-0.11487300	0.33871800
H	3.55443600	0.92529400	0.42563100
C	1.60624300	-1.51877500	1.50364100
H	0.79937500	-2.08732200	1.04523300
H	1.34111700	-1.23661300	2.52668900
C	2.96581000	-2.21391200	1.43033600
H	3.00848200	-2.85482900	0.54468700

H	3.14110200	-2.83143600	2.31170400
C	3.95972000	-1.05725600	1.29383000
H	4.12313100	-0.56807300	2.25892200
H	4.92154200	-1.35464100	0.87707000
C	1.23673200	1.83731400	-0.37367500
H	1.92147000	2.45205500	0.22653500
H	1.72874200	1.58980900	-1.32041700
O	-1.37974500	1.33730200	0.71152900
O	0.04964100	2.52517100	-0.69107700
C	-0.87122500	2.63682600	0.38092700
C	-2.03821300	3.44379000	-0.14272100
H	-2.41498700	2.97599800	-1.05407900
H	-1.71816000	4.46634400	-0.35428300
C	-0.23848300	3.24876200	1.62692600
H	0.24315500	4.19804700	1.37816300
H	0.49836200	2.58332400	2.08610500
H	-1.02225700	3.42939900	2.36486000
C	-0.89013500	-0.41882300	-0.94497100
H	-0.66611600	0.31175000	-1.73443800
C	-2.37122700	-0.78859900	-1.07423100
O	-0.15334400	-1.60373700	-1.10134500
C	3.38892200	-0.55788000	-1.16909300
O	2.33471600	-0.80744100	-1.81174200
O	4.56365000	-0.59249700	-1.55623500
H	-2.83295000	3.46276800	0.60559600
C	-2.98646100	-1.46129900	0.13507800
H	-2.31827600	-2.27729300	0.44292100
C	-4.38417300	-2.01359200	-0.14316900

H	-5.02956800	-1.19914400	-0.49029700
H	-4.32572100	-2.74204800	-0.96115300
H	-3.02984500	-0.73695100	0.95861000
C	-4.99711500	-2.67000500	1.09167500
H	-5.99454600	-3.06370900	0.87893700
H	-4.37474400	-3.50006200	1.44209000
H	-5.08729700	-1.94981600	1.91146900
H	0.76028700	-1.38227900	-1.40384700
H	-2.44547900	-1.43612900	-1.95589800
F	-3.09742800	0.37159100	-1.37228800

(*S*)-**TS3**$_{O}$-F-Pre

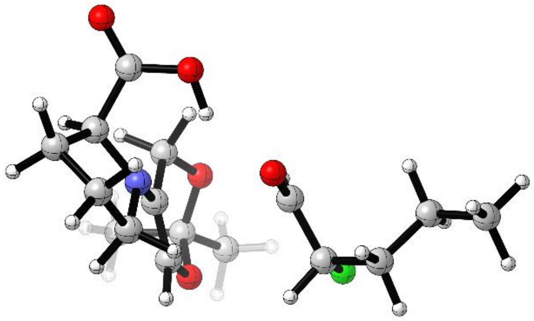

- Thermochemistry -

Zero-point correction=	0.418012 (Hartree/Particle)
Thermal correction to Energy=	0.439302
Thermal correction to Enthalpy=	0.440167
Thermal correction to Gibbs Free Energy=	0.368238
Sum of electronic and zero-point Energies=	-1155.232273
Sum of electronic and thermal Energies=	-1155.210983
Sum of electronic and thermal Enthalpies=	-1155.210118
Sum of electronic and thermal Free Energies=	-1155.282047

Number of Imaginary Frequencies = 0

E (Single Point Energy) [IEFPCM$_{(DCM)}$M06-2X/6-311++G(2d,2p)] = -1155.974910

C	-0.46937300	1.35697900	-1.26947400
H	-0.25359500	1.01451800	-2.27405600
C	-1.17112700	0.65536200	-0.36186800
N	-1.65252400	-0.63734400	-0.58993800
C	-2.89212400	-1.08243000	0.04906600
H	-3.55660100	-0.25392000	0.31642300
C	-1.51147200	-1.21623700	-1.92657400
H	-0.45544700	-1.41641600	-2.13664700
H	-1.89684800	-0.52602300	-2.69485900
C	-2.37060900	-2.47344400	-1.84993900
H	-1.82371300	-3.27398800	-1.34027900
H	-2.67178600	-2.83370700	-2.83486600
C	-3.55540100	-1.99929000	-1.00329500
H	-4.23417500	-1.39788900	-1.61432900
H	-4.12973100	-2.80458100	-0.54227200
C	-1.32289800	1.21488600	1.03124800
H	-2.34881500	1.55719000	1.23040800
H	-1.07660400	0.44968600	1.77627700
O	0.09982900	2.57872900	-1.00532800
O	-0.39467800	2.26595900	1.23977600
C	-0.35372600	3.21558600	0.18998800
C	0.70961800	4.22282600	0.56784700
H	0.86116800	4.92435200	-0.25495800
H	1.64578900	3.69941900	0.77403600
C	-1.71573500	3.85639400	-0.05753700
H	-2.42701500	3.14249000	-0.47951700
H	-1.60041800	4.68061000	-0.76443400
H	-2.11368400	4.24666900	0.88274200

C	1.56882500	-0.45911300	0.27338600
H	1.29933900	0.33042000	0.99921500
C	2.69005800	-0.11805400	-0.68908000
O	1.01591400	-1.53983500	0.25129800
C	-2.63004500	-1.86567900	1.34046800
O	-1.37442200	-2.22359700	1.58955900
O	-3.53234800	-2.17155100	2.08941000
H	-0.75829300	-1.87621300	0.90490800
H	0.39711800	4.77417300	1.45712400
C	3.78176000	-1.17493800	-0.72342800
H	4.51175800	-0.88344400	-1.48660000
H	2.25536600	0.03078500	-1.68478600
C	4.47683800	-1.37990900	0.62303400
H	3.74002700	-1.69109800	1.37397900
H	3.31613700	-2.10941900	-1.05793500
H	4.89559500	-0.42705900	0.96367600
C	5.58311300	-2.42847000	0.52918200
H	5.17883300	-3.39469100	0.21075900
H	6.34390400	-2.12675500	-0.19800000
H	6.07687800	-2.57051900	1.49389200
F	3.21910100	1.10804100	-0.28291100

(*S*)-**TS3**$_O$-F

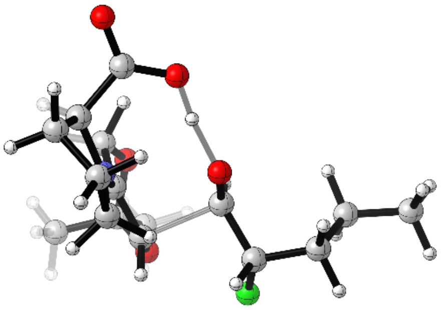

- Thermochemistry -

Zero-point correction=	0.417146 (Hartree/Particle)
Thermal correction to Energy=	0.436769
Thermal correction to Enthalpy=	0.437634
Thermal correction to Gibbs Free Energy=	0.370957
Sum of electronic and zero-point Energies=	-1155.220759
Sum of electronic and thermal Energies=	-1155.201136
Sum of electronic and thermal Enthalpies=	-1155.200271
Sum of electronic and thermal Free Energies=	-1155.266948

Number of Imaginary Frequencies = 1

E (Single Point Energy) [IEFPCM$_{(DCM)}$M06-2X/6-311++G(2d,2p)] = -1155.961139

C	-0.24249000	1.02548600	0.98382400
H	-0.34976300	0.71814500	2.01927400
C	0.95172700	0.81855000	0.29817300
N	1.80888100	-0.14052700	0.64350000
C	3.00642700	-0.48554200	-0.12759000
H	3.52892800	0.41583200	-0.46086800
C	1.74445100	-0.85724700	1.93162300
H	0.74486400	-1.26291300	2.08815600
H	1.98938800	-0.15683800	2.74067700
C	2.80248600	-1.94505800	1.77093700
H	2.36638600	-2.80996000	1.26017400
H	3.20127700	-2.27444900	2.73115000

C	3.85531300	-1.27472300	0.88463100
H	4.45518700	-0.57345900	1.47152100
H	4.52633300	-1.97290400	0.38359200
C	1.18384000	1.56148900	-0.99466200
H	2.09184100	2.17664100	-0.90767200
H	1.32852100	0.85745500	-1.82067600
O	-1.05492400	2.09867300	0.66689400
O	0.06198300	2.34110700	-1.34031100
C	-0.51244500	3.04370200	-0.25068500
C	-1.68453100	3.81666500	-0.81287500
H	-2.23600900	4.29139500	0.00090000
H	-2.34643900	3.13111000	-1.34597300
C	0.50092800	3.94176900	0.45350400
H	1.25619600	3.36755700	0.99605600
H	-0.02417200	4.57202500	1.17389300
H	0.99990000	4.58003800	-0.28029600
C	-1.13047000	-0.64412400	0.06440900
H	-1.41163900	-0.00673900	-0.78864100
C	-2.23557800	-0.91775300	1.07675000
O	-0.32259500	-1.61716300	-0.08633600
C	2.74592300	-1.31666300	-1.40189300
O	1.54463500	-1.76162700	-1.65477800
O	3.69312700	-1.52275700	-2.14073300
H	0.76335700	-1.61614300	-0.94451500
H	-1.32524000	4.58479500	-1.50081400
C	-3.18508500	-1.99997400	0.57713400
H	-3.95435400	-2.13612300	1.34717300
H	-1.78428500	-1.22174800	2.02768200

C	-3.84270500	-1.68708000	-0.76751900
H	-3.07883000	-1.65442500	-1.55336800
H	-2.61835400	-2.93547600	0.51340500
H	-4.30127800	-0.69241300	-0.72743200
C	-4.89778400	-2.73011900	-1.13082100
H	-4.45441600	-3.72988400	-1.18382300
H	-5.69407500	-2.75643300	-0.37983000
H	-5.35490700	-2.51257400	-2.09971800
F	-2.96871500	0.24433900	1.31643800

(*S*)-**TS3**$_{O}$-F-P

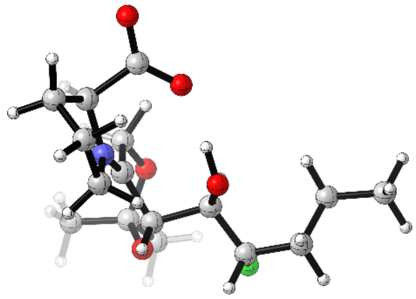

- Thermochemistry -

Zero-point correction= 0.421632 (Hartree/Particle)
Thermal correction to Energy= 0.441327
Thermal correction to Enthalpy= 0.442192
Thermal correction to Gibbs Free Energy= 0.375469
Sum of electronic and zero-point Energies= -1155.244123
Sum of electronic and thermal Energies= -1155.224428
Sum of electronic and thermal Enthalpies= -1155.223563
Sum of electronic and thermal Free Energies= -1155.290286

Number of Imaginary Frequencies = 0

E (Single Point Energy) [IEFPCM$_{(DCM)}$M06-2X/6-311++G(2d,2p)] = -1155.988743

C	0.32315100	0.78873200	-0.93549700
H	0.14652200	0.59261300	-1.99656400

C	-0.95823600	0.76098900	-0.15077300
N	-2.00423300	0.13348500	-0.54769200
C	-3.11908200	-0.21103900	0.35497500
H	-3.37939500	0.64695200	0.97925100
C	-2.20105700	-0.49157100	-1.89217200
H	-1.31552200	-1.06435000	-2.15991800
H	-2.35755500	0.32434500	-2.60365100
C	-3.45545700	-1.34635100	-1.71023300
H	-3.17647300	-2.35141600	-1.38016300
H	-4.01301400	-1.43232200	-2.64331500
C	-4.23110100	-0.62369200	-0.60538300
H	-4.73899500	0.26111400	-1.00097100
H	-4.95916100	-1.25572700	-0.09777600
C	-0.88083700	1.38004700	1.21849600
H	-1.70494900	2.09167000	1.36316000
H	-0.96529100	0.59032100	1.97275400
O	0.98971600	2.02559500	-0.85743200
O	0.37831900	1.98594100	1.39254600
C	0.78461700	2.82010800	0.32099100
C	2.12819600	3.39323000	0.71360000
H	2.54565200	3.94935700	-0.12817300
H	2.80362700	2.57635900	0.97302500
C	-0.24751500	3.89719300	0.00344400
H	-1.16558400	3.47632800	-0.41615100
H	0.17236300	4.57737600	-0.74000700
H	-0.49248700	4.46233700	0.90650700
C	1.16099800	-0.40766500	-0.36509000
H	1.32093400	-0.25251900	0.71131400

C	2.53702700	-0.48158000	-1.03592200
O	0.47510500	-1.60384500	-0.62914500
C	-2.67677300	-1.39755300	1.29982000
O	-1.47099200	-1.75802800	1.24946500
O	-3.59143300	-1.83403200	2.00968600
H	-0.22686400	-1.73516400	0.05283400
H	2.01059400	4.06546500	1.56642400
C	3.24744900	-1.80498700	-0.81162000
H	4.23840800	-1.71889200	-1.27462700
H	2.45270900	-0.26560500	-2.10782100
C	3.39249400	-2.19422400	0.65976900
H	2.39917700	-2.36757600	1.08936800
H	2.70105500	-2.58333200	-1.35364000
H	3.84323600	-1.36332700	1.21484800
C	4.24616500	-3.44972300	0.82812100
H	3.80520100	-4.29511800	0.28968900
H	5.25525900	-3.29130900	0.43350200
H	4.33615600	-3.73150700	1.88065200
F	3.32756300	0.53811300	-0.48586900

(*R*)-**TS3**$_{T}$-F-Pre

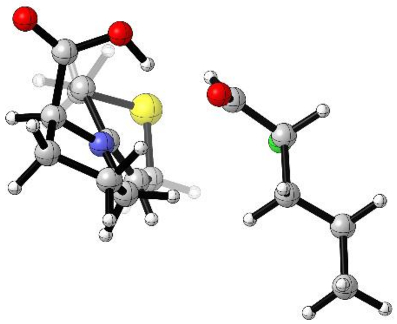

- Thermochemistry -

Zero-point correction=	0.383228 (Hartree/Particle)
Thermal correction to Energy=	0.402669
Thermal correction to Enthalpy=	0.403534
Thermal correction to Gibbs Free Energy=	0.336396
Sum of electronic and zero-point Energies=	-1363.741265
Sum of electronic and thermal Energies=	-1363.721824
Sum of electronic and thermal Enthalpies=	-1363.720959
Sum of electronic and thermal Free Energies=	-1363.788097

Number of Imaginary Frequencies = 0

E (Single Point Energy) [IEFPCM$_{(DCM)}$M06-2X/6-311++G(2d,2p)] = -1364.424353

C	0.33604700	0.95459000	-1.34826300
H	0.81108400	0.13582300	-1.88040400
C	-0.86648300	0.74578400	-0.76698300
N	-1.42003300	-0.53091700	-0.68351100
C	-2.85563100	-0.76539000	-0.55322300
H	-3.46143500	-0.04312300	-1.10872100
C	-0.74485100	-1.67680200	-1.29225800
H	-0.63459000	-1.53805700	-2.37961800
H	0.25111900	-1.79693500	-0.85572800
C	-1.68224400	-2.83616400	-0.97760000
H	-1.53896400	-3.68496300	-1.64837000

H	-1.52227200	-3.17699300	0.05119800
C	-3.05977900	-2.18725100	-1.12798400
H	-3.31889500	-2.10009500	-2.18676700
H	-3.86573500	-2.72282500	-0.62278700
C	-1.64912200	1.83138100	-0.05751200
H	-2.70748400	1.76162100	-0.33312300
H	-1.59896200	1.66000700	1.02691800
C	1.07487400	-0.05558100	1.52006000
H	0.71396100	0.98292000	1.62422200
C	2.56633100	-0.21187500	1.31010600
O	0.33699300	-1.01585900	1.62334800
C	-3.34310500	-0.71067400	0.89703200
O	-2.43278500	-0.84872800	1.85581400
O	-4.51936700	-0.59003900	1.16426000
H	-1.51564000	-0.89523300	1.50132700
C	2.94412500	-1.22532600	0.24791900
H	2.58008000	-0.85984800	-0.72064300
C	4.44829500	-1.48476700	0.18411300
H	2.40444400	-2.15337100	0.47253600
C	4.80371000	-2.48866300	-0.91017800
H	5.88094200	-2.67024600	-0.94578300
H	4.30496500	-3.44779500	-0.73716300
H	4.49161600	-2.12070400	-1.89292500
H	4.78943700	-1.85815100	1.15739200
H	4.97159400	-0.53907000	0.00583100
H	3.00782400	-0.48063900	2.28030300
C	-1.18049600	3.24515400	-0.37550300
C	1.11287600	2.24443700	-1.37084600

H	-1.40346600	3.50482500	-1.41516900
H	-1.68709100	3.96487900	0.26995900
H	2.17048500	2.04078100	-1.17921500
H	1.04726500	2.72969400	-2.35203700
S	0.60276100	3.43522600	-0.09565800
F	3.06886300	1.04713200	0.97369000

(*R*)-**TS3**$_{T}$-F

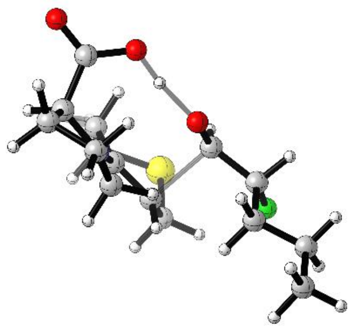

- Thermochemistry -

Zero-point correction= 0.383005 (Hartree/Particle)
Thermal correction to Energy= 0.400864
Thermal correction to Enthalpy= 0.401729
Thermal correction to Gibbs Free Energy= 0.338235
Sum of electronic and zero-point Energies= -1363.730697
Sum of electronic and thermal Energies= -1363.712837
Sum of electronic and thermal Enthalpies= -1363.711972
Sum of electronic and thermal Free Energies= -1363.775467

Number of Imaginary Frequencies = 1

E (Single Point Energy) [IEFPCM$_{(DCM)}$M06-2X/6-311++G(2d,2p)] = -1364.411768

C	-0.44261900	0.95073500	0.91582400
H	-1.01752600	0.22219000	1.47924800
C	0.91311900	0.66559100	0.73466800
N	1.35650200	-0.59635500	0.81961800
C	2.72263200	-1.02838300	0.50119200

H	3.46354600	-0.40812000	1.00973900
C	0.54415700	-1.70871900	1.35177900
H	0.47318600	-1.60890500	2.44281500
H	-0.45462100	-1.69042600	0.92044700
C	1.33086500	-2.94610400	0.93812200
H	1.11881300	-3.80316900	1.57897500
H	1.07777900	-3.21026700	-0.09414400
C	2.78155400	-2.47402700	1.03278700
H	3.10587600	-2.45403600	2.07709100
H	3.48757000	-3.08172400	0.46515200
C	1.88813300	1.69700900	0.20818300
H	2.88717100	1.48862700	0.60071200
H	1.94776700	1.57963900	-0.88320500
C	-0.92301900	0.23017600	-1.08584300
H	-0.60334400	1.19810500	-1.49842900
C	-2.43385500	0.04810300	-1.03213500
O	-0.26070900	-0.82336300	-1.32722300
C	3.08392200	-0.97747600	-0.99417900
O	2.13173600	-0.88456500	-1.88709100
O	4.26079100	-1.05210000	-1.30129500
H	1.12729500	-0.82949500	-1.56694300
C	-2.95772600	-1.04727800	-0.12947900
H	-2.79069000	-0.76303900	0.91703400
C	-4.44276800	-1.33582100	-0.35088800
H	-2.36528900	-1.94826800	-0.32933100
C	-4.95684000	-2.42484100	0.58780600
H	-6.01743800	-2.62746200	0.41749700
H	-4.40648300	-3.35970100	0.43949600

H	-4.83506600	-2.12666100	1.63430200
H	-4.59777500	-1.63975700	-1.39325800
H	-5.01678100	-0.41473600	-0.20103900
H	-2.72229900	-0.16167400	-2.07301700
C	1.54153800	3.14354500	0.54411900
C	-0.95651800	2.36447500	1.08830200
H	1.63456200	3.32617000	1.61910100
H	2.23084400	3.81355600	0.02762900
H	-2.01900700	2.40778300	0.85224900
H	-0.83634200	2.68940300	2.12868500
S	-0.13777300	3.58275800	0.01986900
F	-3.04143300	1.26562600	-0.70696900

(*R*)-**TS3**$_{T}$-F-P

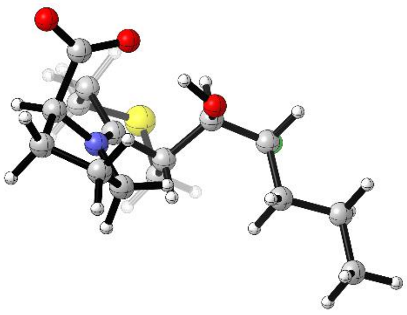

- Thermochemistry -

Zero-point correction= 0.387574 (Hartree/Particle)
Thermal correction to Energy= 0.405468
Thermal correction to Enthalpy= 0.406333
Thermal correction to Gibbs Free Energy= 0.343195
Sum of electronic and zero-point Energies= -1363.758758
Sum of electronic and thermal Energies= -1363.740865
Sum of electronic and thermal Enthalpies= -1363.740000
Sum of electronic and thermal Free Energies= -1363.803138

Number of Imaginary Frequencies = 0

E (Single Point Energy) [IEFPCM$_{(DCM)}$M06-2X/6-311++G(2d,2p)] = -1364.444298

C	0.57750600	0.50839400	-0.56689200
H	0.84112100	-0.33444200	-1.20879900
C	-0.92223500	0.62901500	-0.50208100
N	-1.66420200	-0.39052800	-0.78116000
C	-3.10706200	-0.47809400	-0.45542100
H	-3.63584800	0.39840600	-0.82935800
C	-1.16829700	-1.71284800	-1.27871200
H	-0.96201000	-1.60137400	-2.34742200
H	-0.26403000	-1.99163900	-0.74293000
C	-2.33340000	-2.66283600	-1.01760500
H	-2.32860900	-3.49732500	-1.71976000
H	-2.26425300	-3.06297200	-0.00141400
C	-3.55889500	-1.76073000	-1.15167000
H	-3.77743000	-1.56111900	-2.20528100
H	-4.45465400	-2.15834900	-0.67483300
C	-1.51694800	1.89382200	0.05439500
H	-2.59980600	1.90524500	-0.06385900
H	-1.31973400	1.87902400	1.13384500
C	1.02804300	0.15435500	0.89032700
H	0.83683000	1.02091300	1.53729100
C	2.51991600	-0.17220300	1.00707500
O	0.35901600	-0.99395800	1.35158500
C	-3.29632200	-0.54879300	1.10403100
O	-2.26136000	-0.49929500	1.82111000
O	-4.48022400	-0.64100200	1.45975200
H	-0.58607200	-0.79703000	1.56535500
C	3.05193700	-1.18308600	0.01233100

H	3.03542100	-0.74654000	-0.99538600
C	4.47057100	-1.64762800	0.34207900
H	2.36756900	-2.04163900	0.01041100
C	4.99877100	-2.64673600	-0.68457700
H	6.01103200	-2.97512100	-0.43475400
H	4.35825300	-3.53359700	-0.73099400
H	5.02675300	-2.20082000	-1.68425100
H	4.47445500	-2.10256100	1.33996100
H	5.13324600	-0.77633100	0.38880500
H	2.68040900	-0.52286500	2.03255100
C	-0.93290300	3.17269600	-0.55609800
C	1.25495100	1.74990800	-1.16910800
H	-1.16198900	3.23614000	-1.62415900
H	-1.38503200	4.03511000	-0.06384600
H	2.33794000	1.63184300	-1.12994900
H	0.96511000	1.84578300	-2.22028500
S	0.85926000	3.29900100	-0.31268700
F	3.24881200	1.02261900	0.87912600

(*S*)-**TS3**$_T$-F-Pre

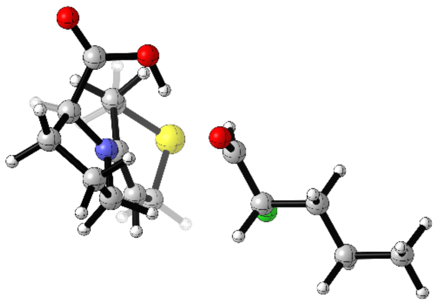

- Thermochemistry -

Zero-point correction= 0.383259 (Hartree/Particle)

Thermal correction to Energy=	0.402762
Thermal correction to Enthalpy=	0.403627
Thermal correction to Gibbs Free Energy=	0.335820
Sum of electronic and zero-point Energies=	-1363.740781
Sum of electronic and thermal Energies=	-1363.721278
Sum of electronic and thermal Enthalpies=	-1363.720413
Sum of electronic and thermal Free Energies=	-1363.788219

Number of Imaginary Frequencies = 0

E (Single Point Energy) [IEFPCM$_{(DCM)}$M06-2X/6-311++G(2d,2p)] = -1364.423886

C	0.20464500	1.31886400	1.37946600
H	-0.17067100	0.69462200	2.18547700
C	1.21094800	0.85696000	0.60324500
N	1.66758900	-0.45632100	0.70912300
C	3.01601300	-0.85741700	0.31339500
H	3.76782100	-0.08763900	0.51133500
C	1.12649800	-1.35035000	1.73328600
H	1.30149600	-0.94488800	2.74245000
H	0.04876100	-1.47321400	1.59153800
C	1.90846900	-2.63935200	1.51358600
H	1.90541500	-3.28645500	2.39229600
H	1.48152200	-3.19544800	0.67183400
C	3.30331600	-2.11471000	1.16772300
H	3.82315500	-1.80481500	2.07844500
H	3.93546000	-2.83281400	0.64215700
C	1.82898700	1.65800700	-0.52436800
H	2.91919600	1.54995200	-0.50031800
H	1.49554000	1.23537800	-1.48243600
C	-1.28876400	-0.27220300	-0.76738200
H	-1.04143100	0.67432100	-1.28038100
C	-2.55683100	-0.27061800	0.05729500

O	-0.61899500	-1.27884000	-0.89328700
C	3.13026800	-1.19694300	-1.17519900
O	2.00545700	-1.45609100	-1.83449400
O	4.20805100	-1.27008200	-1.72513000
H	1.20550100	-1.31762300	-1.27922700
C	-3.64033200	-1.11914200	-0.59088000
H	-3.23519700	-2.13106600	-0.70918900
C	-4.92770100	-1.15282400	0.23109700
H	-3.84082200	-0.72741900	-1.59637100
C	-6.00257800	-2.00662100	-0.43804000
H	-6.91849200	-2.02680400	0.15815800
H	-6.25313300	-1.61161900	-1.42773100
H	-5.65916100	-3.03820600	-0.56564700
H	-5.29642000	-0.13097000	0.36859600
H	-4.70482900	-1.54813300	1.22979200
H	-2.33690200	-0.61811800	1.07406800
C	1.51245400	3.14726500	-0.48467300
C	-0.46253300	2.66514400	1.27190900
H	2.01046000	3.62908900	0.36263500
H	1.85857000	3.63010600	-1.40026600
H	-1.53883700	2.55662900	1.42745800
H	-0.08923800	3.35515900	2.03819100
S	-0.27253800	3.45065400	-0.35624400
F	-2.99039300	1.05305400	0.15374600

(*S*)-**TS3**$_{T}$-F

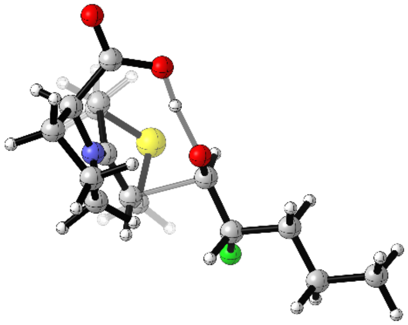

- Thermochemistry -

Zero-point correction=	0.382864 (Hartree/Particle)
Thermal correction to Energy=	0.400862
Thermal correction to Enthalpy=	0.401727
Thermal correction to Gibbs Free Energy=	0.337760
Sum of electronic and zero-point Energies=	-1363.731314
Sum of electronic and thermal Energies=	-1363.713316
Sum of electronic and thermal Enthalpies=	-1363.712451
Sum of electronic and thermal Free Energies=	-1363.776417

Number of Imaginary Frequencies = 1

E (Single Point Energy) [IEFPCM$_{(DCM)}$M06-2X/6-311++G(2d,2p)] = -1364.412197

C	-0.05927400	1.18178800	1.08340400
H	-0.44005500	0.64011100	1.94564000
C	1.19452300	0.79823000	0.60101000
N	1.66001100	-0.43742300	0.83262300
C	2.89349600	-0.98964200	0.25999200
H	3.73690900	-0.31127100	0.40485400
C	1.04167300	-1.37280400	1.79361600
H	1.27444300	-1.04301400	2.81456700
H	-0.03726200	-1.39978900	1.65771900
C	1.69890900	-2.70515100	1.45691800
H	1.68040400	-3.39887600	2.29867000

H	1.17560500	-3.16505100	0.61204300
C	3.11409400	-2.29257500	1.05345800
H	3.71162100	-2.06928100	1.94176600
H	3.64553300	-3.03803700	0.46038300
C	1.97454000	1.63759200	-0.38863000
H	3.04537300	1.48039400	-0.23126000
H	1.74589000	1.27031800	-1.39862800
C	-1.11900400	0.01724300	-0.40124700
H	-1.11028900	0.88651900	-1.07675300
C	-2.43335600	-0.23575500	0.32363800
O	-0.47067800	-1.03296700	-0.68707100
C	2.83718300	-1.27143200	-1.25244200
O	1.68019800	-1.30922900	-1.86492900
O	3.88707600	-1.47656100	-1.83501600
H	0.80960000	-1.13248500	-1.31051500
C	-3.41791900	-0.93288400	-0.60299800
H	-2.94861100	-1.86269000	-0.94251600
C	-4.75580400	-1.22884200	0.07375500
H	-3.57153700	-0.30200500	-1.48883000
C	-5.72447400	-1.93619300	-0.87184500
H	-6.67737300	-2.14647200	-0.37890800
H	-5.92964700	-1.31836200	-1.75207300
H	-5.30691300	-2.88644000	-1.22010800
H	-5.19977000	-0.29272000	0.42829400
H	-4.57934400	-1.85194500	0.95951800
H	-2.26050000	-0.83230300	1.22686100
C	1.70185200	3.13533600	-0.32288200
C	-0.54802600	2.61406500	1.02514200

H	2.06929700	3.55916100	0.61677300
H	2.21664800	3.63738500	-1.14367900
H	-1.63681400	2.63523500	1.05865600
H	-0.18200000	3.17995000	1.88988700
S	-0.06505200	3.50425400	-0.48091400
F	-2.99707700	0.97686300	0.73385400

(*S*)-**TS3**$_{T}$-F-P

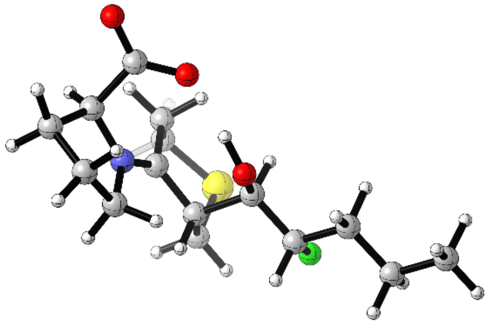

- Thermochemistry -

Zero-point correction=	0.387679 (Hartree/Particle)
Thermal correction to Energy=	0.405559
Thermal correction to Enthalpy=	0.406424
Thermal correction to Gibbs Free Energy=	0.343139
Sum of electronic and zero-point Energies=	-1363.757525
Sum of electronic and thermal Energies=	-1363.739645
Sum of electronic and thermal Enthalpies=	-1363.738780
Sum of electronic and thermal Free Energies=	-1363.802065

Number of Imaginary Frequencies = 0

E (Single Point Energy) [IEFPCM$_{(DCM)}$M06-2X/6-311++G(2d,2p)] = -1364.442999

C	-0.27833800	0.78098200	0.88311200
H	-0.28149500	0.25921400	1.84340500
C	1.11367100	0.76798500	0.30581500
N	1.98137500	-0.09455300	0.71800200
C	3.25165200	-0.39304700	0.01648000

H	3.79938700	0.52600200	-0.19046300
C	1.76683600	-1.07438500	1.82959200
H	1.88431300	-0.52882800	2.77070700
H	0.76778800	-1.49694400	1.75634300
C	2.86988200	-2.10919200	1.62620900
H	3.14897900	-2.58035400	2.56935500
H	2.52586600	-2.88716600	0.93778200
C	3.99957200	-1.30003300	0.99136300
H	4.51562600	-0.69868700	1.74629900
H	4.73324200	-1.90636300	0.46035900
C	1.40449400	1.66798300	-0.86421700
H	2.46041300	1.64285900	-1.12987000
H	0.85632300	1.25260500	-1.71907200
C	-1.14602600	-0.07970300	-0.09886500
H	-1.23163600	0.44274500	-1.06251700
C	-2.56786300	-0.29159100	0.42892000
O	-0.57341800	-1.35492400	-0.24768400
C	2.94414900	-1.11021200	-1.34910300
O	1.73485000	-1.27454900	-1.66230600
O	3.95883400	-1.43351400	-1.98355300
H	0.24634500	-1.31882100	-0.79917200
C	-3.36111300	-1.30531900	-0.37009300
H	-2.84109000	-2.26561400	-0.30553400
C	-4.79960100	-1.44840400	0.12622500
H	-3.35098000	-1.00329100	-1.42605200
C	-5.57400900	-2.49270500	-0.67545000
H	-6.60146800	-2.58985000	-0.31451700
H	-5.61375400	-2.21854200	-1.73471500

H	-5.09568300	-3.47487900	-0.60277100
H	-5.30754900	-0.48041100	0.06255200
H	-4.78812200	-1.72999600	1.18700600
H	-2.54802100	-0.56198200	1.49366300
C	0.97843000	3.12401400	-0.64171400
C	-0.78142500	2.20713800	1.15859600
H	1.54772900	3.57640600	0.17595400
H	1.18441500	3.69276400	-1.54978800
H	-1.79958500	2.17060800	1.54361000
H	-0.14901100	2.66731500	1.92428000
S	-0.79576500	3.28116000	-0.30267800
F	-3.23148900	0.94637200	0.34745700

Supplementary Table 12. Energies for enamine addition to 2-bromopentanal. Reported energies for structures optimized at the IEFPCM$_{(DCM)}$M06-2X/6-311++G(2d,2p)//IEFPCM$_{(DCM)}$M06-2X/6-31+G(d,p) level of theory represent the sum of the thermal correction to Gibbs Free Energy computed at the IEFPCM$_{(DCM)}$M06-2X/6-31+G(d,p) level of theory and single point energies computed at the IEFPCM$_{(DCM)}$M06-2X/6-311++G(2d,2p). All energies are reported in Hartrees.

**Structure**	**Single Point Energies, E IEFPCM$_{(DCM)}$M06-2X/6-311++G(2d,2p)**	**Thermal Corrections to Gibbs Free Energies, IEFPCM$_{(DCM)}$M06-2X/6-31+G(d,p)**	**Gibbs Free Energies (G), IEFPCM$_{(DCM)}$M06-2X/6-31+G(d,p)**	**Gibbs Free Energies (G), IEFPCM$_{(DCM)}$M06-2X/6-311++G(2d,2p)// IEFPCM$_{(DCM)}$M06-2X/6-31+G(d,p)**
2-Bromopentanal	-2845.302023	0.102041	-2842.756584	-2845.199982
Enamine of Cyclohexanone (G)	-634.55147535	0.238392	-634.142903	-634.3130834
Enamine of Dioxane (O)	-784.98483791	0.244077	-784.522619	-784.7407609
Enamine of Tetrahydro-4H-thiopyranone (T)	-993.43210840	0.210205	-993.028735	-993.2219034
Enamine ofTetrahydro-4H-pyranone (P)	-670.45469021	0.214793	-670.055312	-670.2398972
(*R*)-**TS3**$_P$-Br-Pre	-3515.772666	0.336557	-3512.81573	-3515.436109
(*R*)-**TS3**$_P$-Br	-3515.75725	0.339631	-3512.79908	-3515.417619
(*R*)-**TS3**$_P$-Br-P	-3515.792008	0.344841	-3512.828515	-3515.447167
(*S*)-**TS3**$_P$-Br-Pre	-3515.773353	0.336644	-3512.81529	-3515.436709
(*S*)-**TS3**$_P$-Br	-3515.757188	0.339054	-3512.798467	-3515.418134
(*S*)-**TS3**$_P$-Br-P	-3515.788684	0.343919	-3512.825136	-3515.444765
(*R*)-**TS3**$_G$-Br-Pre	-3479.870365	0.360779	-3476.903726	-3479.509586
(*R*)-**TS3**$_G$-Br	-3479.854643	0.364378	-3476.886609	-3479.490265
(*R*)-**TS3**$_G$-Br-P	-3479.885557	0.368487	-3476.913388	-3479.51707
(*S*)-**TS3**$_G$-Br-Pre	-3479.868737	0.358896	-3476.903929	-3479.509841
(*S*)-**TS3**$_G$-Br	-3479.854911	0.363619	-3476.886337	-3479.491292
(*S*)-**TS3**$_G$-Br-P	-3479.88184	0.366119	-3476.910799	-3479.515721
(*R*)-**TS3**$_O$-Br-Pre	-3630.303915	0.365694	-3627.284499	-3629.938221
(*R*)-**TS3**$_O$-Br	-3630.286244	0.36793	-3627.26738	-3629.918314

(*R*)-**TS3**$_{O}$-Br-P	-3630.315445	0.37229	-3627.291794	-3629.943146
(*S*)-**TS3**$_{O}$-Br-Pre	-3630.301967	0.36445	-3627.284609	-3629.937517
(*S*)-**TS3**$_{O}$-Br	-3630.285366	0.36805	-3627.265946	-3629.917315
(*S*)-**TS3**$_{O}$-Br-P	-3630.313357	0.373188	-3627.290085	-3629.940169
(*R*)-**TS3**$_{T}$-Br-Pre	-3838.751494	0.33315	-3835.789332	-3838.418344
(*R*)-**TS3**$_{T}$-Br	-3838.736601	0.33612	-3835.773702	-3838.400481
(*R*)-**TS3**$_{T}$-Br-P	-3838.769308	0.340446	-3835.801764	-3838.428862
(*S*)-**TS3**$_{T}$-Br-Pre	-3838.74956	0.333301	-3835.787541	-3838.416259
(*S*)-**TS3**$_{T}$-Br	-3838.734863	0.335584	-3835.772793	-3838.399279
(*S*)-**TS3**$_{T}$-Br-P	-3838.765953	0.33997	-3835.798642	-3838.425983

Pre – Precomplex

P – Product

(*R*)-**TS3**$_{P}$-Br-Pre

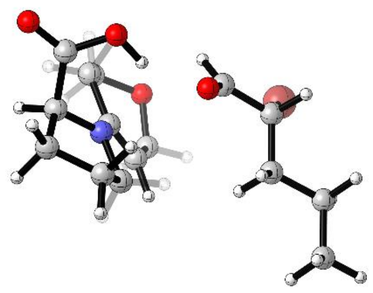

- Thermochemistry -

Zero-point correction=	0.385072 (Hartree/Particle)
Thermal correction to Energy=	0.404566
Thermal correction to Enthalpy=	0.405431
Thermal correction to Gibbs Free Energy=	0.336557
Sum of electronic and zero-point Energies=	-3512.767216
Sum of electronic and thermal Energies=	-3512.747721
Sum of electronic and thermal Enthalpies=	-3512.746856
Sum of electronic and thermal Free Energies=	-3512.81573

Number of Imaginary Frequencies = 0

E (Single Point Energy) [IEFPCM$_{(DCM)}$M06-2X/6-311++G(2d,2p)] = -3515.772666

C	0.13692600	-0.69470600	-1.67490500
H	-0.16286800	0.21915500	-2.17761300
C	1.26288700	-0.74685400	-0.93307200
N	2.02254100	0.37716900	-0.62748600
C	3.45168500	0.29604200	-0.33825900
H	3.96263300	-0.47891800	-0.91812600
C	1.66114500	1.67911600	-1.18508600
H	1.67034900	1.65320200	-2.28659600
H	0.65555800	1.95733300	-0.85465700
C	2.75382000	2.59443400	-0.64604100
H	2.86212000	3.50991900	-1.23004600
H	2.53215000	2.86926400	0.39096700
C	3.99189300	1.69620700	-0.71863100
H	4.36590700	1.65735700	-1.74531700
H	4.81152500	2.01116500	-0.06998000
C	1.68699100	-2.04635900	-0.28682700
H	2.75486400	-2.23265500	-0.44624300
H	1.53749200	-1.98443900	0.80010000
C	-0.70030000	0.14391800	1.33706800
H	-0.48572700	-0.93732900	1.38127400
C	-2.13367600	0.56585700	1.07487600
O	0.15655500	0.97857700	1.53889700
C	3.74961300	-0.00210000	1.13292000
O	2.78381600	0.23995200	2.01351100
O	4.83741600	-0.40015700	1.49035700
H	1.94409700	0.51633100	1.58037600

C	-2.25893000	1.60578100	-0.02991000
H	-1.94648400	1.15773900	-0.98045600
C	-3.65538900	2.20797000	-0.15770700
H	-1.53789100	2.39723600	0.21390900
C	-3.67950600	3.35659900	-1.16339300
H	-4.68546100	3.77224100	-1.26261400
H	-3.00904600	4.16421700	-0.85316800
H	-3.35852600	3.01371000	-2.15245800
H	-3.98302700	2.56566500	0.82635200
H	-4.36156900	1.42826900	-0.46192400
H	-2.53138400	0.95790400	2.01634100
C	0.89543400	-3.21608500	-0.86390300
C	-0.74065600	-1.90243500	-1.87697300
H	1.25937000	-3.45392400	-1.87505400
H	1.00420800	-4.10389000	-0.23849500
H	-1.79484100	-1.63314100	-1.76999800
H	-0.60394000	-2.31990300	-2.88906800
O	-0.48679900	-2.91319800	-0.91706800
Br	-3.19762400	-1.02321100	0.69623900

(*R*)-**TS3**$_{P}$-Br

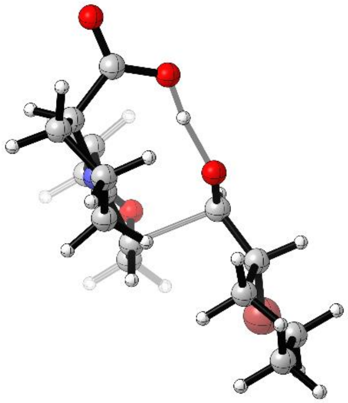

- Thermochemistry -

Zero-point correction=	0.385067 (Hartree/Particle)
Thermal correction to Energy=	0.402946
Thermal correction to Enthalpy=	0.403811
Thermal correction to Gibbs Free Energy=	0.339631
Sum of electronic and zero-point Energies=	-3512.753644
Sum of electronic and thermal Energies=	-3512.735766
Sum of electronic and thermal Enthalpies=	-3512.734901
Sum of electronic and thermal Free Energies=	-3512.799080

Number of Imaginary Frequencies = 1

E (Single Point Energy) [IEFPCM$_{(DCM)}$M06-2X/6-311++G(2d,2p)] = -3515.757250

C	-0.05382400	1.04388800	0.98471500
H	-0.55911400	0.30890800	1.60601300
C	1.31270700	0.88607700	0.73561000
N	1.91427400	-0.30382700	0.79226800
C	3.31260200	-0.55510500	0.42306400
H	3.98144900	0.15715100	0.91129600
C	1.27799800	-1.50069500	1.36813500
H	1.25436500	-1.40322300	2.46140400
H	0.26233500	-1.59891400	0.99451100

C	2.18960500	-2.63308000	0.91287200
H	2.11620300	-3.50903500	1.55884900
H	1.92443300	-2.92780100	-0.10817300
C	3.57377600	-1.98301000	0.94423600
H	3.93944900	-1.92300300	1.97309500
H	4.32350800	-2.49808700	0.34247200
C	2.09495200	2.03977900	0.15420700
H	3.13654000	2.01236900	0.48442500
H	2.09813400	1.93453000	-0.93972500
C	-0.60298800	0.25128900	-0.96790600
H	-0.56569600	1.28039800	-1.35323400
C	-2.00683600	-0.34638700	-0.82556800
O	0.25776900	-0.60633500	-1.32243000
C	3.61998800	-0.45481800	-1.08172600
O	2.63824100	-0.43309700	-1.94854100
O	4.78802700	-0.42238800	-1.42416100
H	1.65621900	-0.45761200	-1.59738300
C	-2.11313000	-1.60355500	0.01786800
H	-1.97653900	-1.35369400	1.07758700
C	-3.42021400	-2.37246000	-0.16710200
H	-1.27682800	-2.24409600	-0.28955800
C	-3.38441800	-3.71905800	0.55207800
H	-4.32705300	-4.25851100	0.42847200
H	-2.57992000	-4.35057200	0.16185100
H	-3.21421500	-3.58260100	1.62519500
H	-3.59428300	-2.52665000	-1.23935500
H	-4.25638300	-1.77163000	0.20535200
H	-2.29885400	-0.57521500	-1.85622200

C	1.47703800	3.38126000	0.53004100
C	-0.57597600	2.46465800	1.12714400
H	1.65444500	3.59966000	1.59359100
H	1.91549200	4.18237000	-0.06687500
H	-1.63068600	2.52178600	0.87227100
H	-0.46137100	2.80056300	2.17118200
O	0.08910800	3.37354600	0.26998500
Br	-3.35183900	0.96066300	-0.26879300

(*R*)-**TS3**$_P$-Br-P

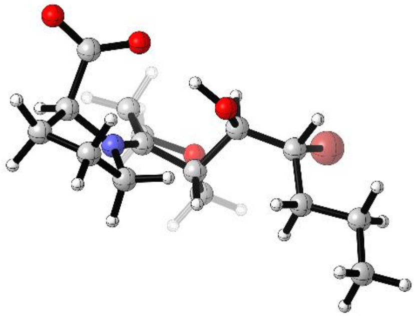

- Thermochemistry -

Zero-point correction= 0.389626 (Hartree/Particle)
Thermal correction to Energy= 0.407435
Thermal correction to Enthalpy= 0.408300
Thermal correction to Gibbs Free Energy= 0.344841
Sum of electronic and zero-point Energies= -3512.783729
Sum of electronic and thermal Energies= -3512.765921
Sum of electronic and thermal Enthalpies= -3512.765056
Sum of electronic and thermal Free Energies= -3512.828515

Number of Imaginary Frequencies = 0

E (Single Point Energy) [IEFPCM$_{(DCM)}$M06-2X/6-311++G(2d,2p)] = -3515.792008

C	-0.09166900	-0.22464300	-0.83228300
H	-0.20428800	0.73685300	-1.33664300

C	1.36038900	-0.58353200	-0.70352700
N	2.28279500	0.31605000	-0.74149000
C	3.67494100	0.08004000	-0.29915500
H	4.08681700	-0.80407000	-0.78590500
C	2.06245500	1.76875100	-1.01770200
H	1.95788200	1.88062500	-2.10112300
H	1.16052000	2.10537700	-0.51152300
C	3.33327300	2.43320300	-0.49506000
H	3.54144400	3.36320500	-1.02521900
H	3.22314600	2.65737400	0.57034000
C	4.40290600	1.36097800	-0.70499000
H	4.69747000	1.31050900	-1.75784700
H	5.29524700	1.50320600	-0.09551700
C	1.65215200	-2.02187600	-0.39751800
H	2.70160300	-2.27346000	-0.55004800
H	1.43509600	-2.17060200	0.66768700
C	-0.60385300	-0.07173200	0.64145400
H	-0.52492500	-1.04215900	1.14647500
C	-2.05707200	0.41424400	0.75353100
O	0.15986500	0.90698000	1.30761400
C	3.70059300	-0.14662000	1.25690000
O	2.60115100	-0.10787700	1.87203600
O	4.83552500	-0.34562200	1.71335400
H	1.04670100	0.54282100	1.55440700
C	-2.45114900	1.50463700	-0.22489500
H	-2.50622300	1.09700200	-1.24214800
C	-3.75782500	2.21645900	0.11601900
H	-1.63349700	2.24101400	-0.21224500

C	-4.01090000	3.39912800	-0.81611800
H	-4.95402800	3.89704400	-0.57674000
H	-3.20875000	4.13984200	-0.73688600
H	-4.06130600	3.06772700	-1.85859800
H	-3.71543000	2.56130100	1.15635800
H	-4.58737000	1.50408200	0.05190100
H	-2.17858900	0.75976500	1.78107800
C	0.76184500	-2.95177200	-1.23582700
C	-0.81334300	-1.29570000	-1.66575600
H	1.07906500	-2.93245300	-2.28826600
H	0.84979500	-3.97255900	-0.86343400
H	-1.88750800	-1.11502200	-1.67218500
H	-0.44889300	-1.24876700	-2.70275900
O	-0.59976600	-2.58678300	-1.13890400
Br	-3.31697800	-1.09268500	0.63702900

(*S*)-**TS3**$_{P}$-Br-Pre

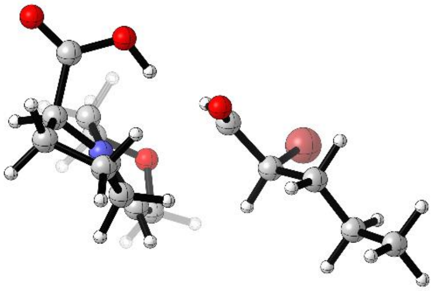

- Thermochemistry -

Zero-point correction= 0.385021 (Hartree/Particle)
Thermal correction to Energy= 0.404512
Thermal correction to Enthalpy= 0.405377
Thermal correction to Gibbs Free Energy= 0.336644
Sum of electronic and zero-point Energies= -3512.766914
Sum of electronic and thermal Energies= -3512.747422

Sum of electronic and thermal Enthalpies= -3512.746557
Sum of electronic and thermal Free Energies= -3512.815290

Number of Imaginary Frequencies = 0

E (Single Point Energy) [IEFPCM$_{(DCM)}$M06-2X/6-311++G(2d,2p)] = -3515.773353

C	0.44403000	0.91209100	1.75795300
H	0.16444900	0.07887200	2.39544400
C	1.49619900	0.82657500	0.91612700
N	2.17554800	-0.36006500	0.67649900
C	3.55105900	-0.39728300	0.19007200
H	4.16677900	0.41547900	0.58695900
C	1.82721600	-1.57378600	1.41395800
H	1.96158000	-1.42818200	2.49732200
H	0.78011700	-1.83478300	1.22726900
C	2.81219600	-2.60010900	0.86583700
H	2.95748800	-3.44482400	1.54119200
H	2.45502100	-2.98419500	-0.09593100
C	4.08248100	-1.76551000	0.68019600
H	4.57914800	-1.62008800	1.64344600
H	4.80490200	-2.19926900	-0.01373500
C	1.90008200	2.01826700	0.07848300
H	2.97865400	2.19640200	0.15155300
H	1.68422200	1.81878600	-0.98035100
C	-0.72891500	-0.15189200	-0.84556600
H	-0.44013600	0.88506100	-1.08421700
C	-2.03883400	-0.32178200	-0.10191100
O	-0.04026800	-1.09867600	-1.16634400
C	3.65510700	-0.30660800	-1.33479800
O	2.57085200	-0.60053300	-2.04678900

O	4.69965800	-0.02127300	-1.87896500
H	1.78414000	-0.77515100	-1.48397000
C	-2.63296300	-1.71218600	-0.21236600
H	-1.84404800	-2.40900400	0.09940900
C	-3.87368200	-1.92051900	0.65140600
H	-2.85187500	-1.93070000	-1.26413000
C	-4.34670400	-3.37169800	0.61724900
H	-5.24546100	-3.50897100	1.22392300
H	-4.58121700	-3.68012100	-0.40668000
H	-3.57356800	-4.04495700	1.00108200
H	-4.67449400	-1.25901600	0.30374500
H	-3.64781500	-1.62551200	1.68406600
H	-1.87857700	-0.04055800	0.94167900
C	1.16117200	3.27040500	0.53908400
C	-0.42138800	2.14457900	1.82615500
H	1.58705000	3.63655700	1.48516700
H	1.23814200	4.06309100	-0.20686600
H	-1.48498500	1.87795700	1.81214200
H	-0.24325200	2.70651000	2.75804600
O	-0.21853600	2.99850100	0.71466500
Br	-3.23774500	1.05999000	-0.79382500

(*S*)-**TS3**P-Br

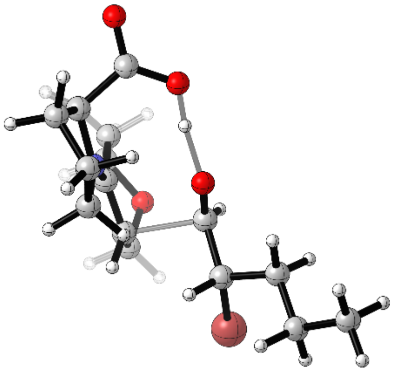

- Thermochemistry -

Zero-point correction=	0.384719 (Hartree/Particle)
Thermal correction to Energy=	0.402687
Thermal correction to Enthalpy=	0.403553
Thermal correction to Gibbs Free Energy=	0.339054
Sum of electronic and zero-point Energies=	-3512.752802
Sum of electronic and thermal Energies=	-3512.734833
Sum of electronic and thermal Enthalpies=	-3512.733968
Sum of electronic and thermal Free Energies=	-3512.798467

Number of Imaginary Frequencies = 1

E (Single Point Energy) [IEFPCM$_{(DCM)}$M06-2X/6-311++G(2d,2p)] = -3515.757188

C	0.23501800	1.38801200	0.79532000
H	-0.20565900	0.96697600	1.69586100
C	1.54173900	1.01980300	0.46163200
N	2.06407400	-0.14716400	0.84180600
C	3.37867400	-0.64965400	0.42539100
H	4.15438600	0.10354600	0.58050500
C	1.42903700	-1.03426500	1.83484600
H	1.55725600	-0.60197900	2.83549900
H	0.36826800	-1.14140000	1.61899900
C	2.19584900	-2.34053400	1.67166800

H	2.14979600	-2.96192500	2.56701900
H	1.77859000	-2.90461600	0.83092800
C	3.61203600	-1.86204000	1.34900900
H	4.11300800	-1.52220400	2.25972000
H	4.24027200	-2.61464400	0.87108500
C	2.30613500	1.83873300	-0.55046900
H	3.37919600	1.81400200	-0.34314700
H	2.15533000	1.39047600	-1.54204200
C	-0.66072600	0.01034100	-0.60775200
H	-0.74950300	0.82913000	-1.33757100
C	-1.95583300	-0.41524100	0.09463200
O	0.12838300	-0.95785800	-0.81165300
C	3.48177200	-1.05669200	-1.05589100
O	2.39360800	-1.20002300	-1.77173500
O	4.59099800	-1.25485700	-1.51657600
H	1.47548000	-1.02289700	-1.31679100
C	-2.64444600	-1.51271900	-0.71511100
H	-1.89294900	-2.29749200	-0.85805400
C	-3.87767900	-2.10996100	-0.04120100
H	-2.89884300	-1.12060100	-1.70790300
C	-4.36926400	-3.35344500	-0.77952100
H	-5.26485700	-3.76585900	-0.30727800
H	-4.61700100	-3.11534200	-1.81916400
H	-3.60176500	-4.13384200	-0.78904900
H	-4.67670700	-1.36291600	0.00132400
H	-3.63039100	-2.36465900	0.99741500
H	-1.76106800	-0.75359700	1.11406600
C	1.82265900	3.28356000	-0.57154600

C	-0.19484300	2.81342500	0.49017300
H	2.14278300	3.80811900	0.34103900
H	2.23090900	3.80908000	-1.43606900
H	-1.26813500	2.86694700	0.32204800
H	0.04682900	3.46652700	1.34504800
O	0.41525900	3.32668100	-0.67873000
Br	-3.19144200	1.09233800	0.27446700

(*S*)-**TS3**$_{P}$-Br-P

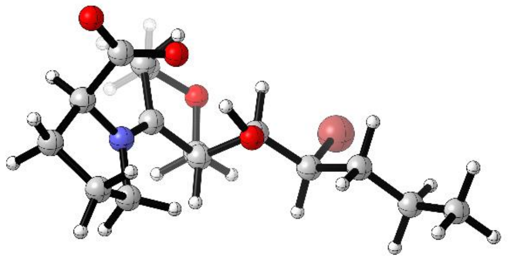

- Thermochemistry -

Zero-point correction=	0.389305 (Hartree/Particle)
Thermal correction to Energy=	0.407158
Thermal correction to Enthalpy=	0.408023
Thermal correction to Gibbs Free Energy=	0.343919
Sum of electronic and zero-point Energies=	-3512.779751
Sum of electronic and thermal Energies=	-3512.761897
Sum of electronic and thermal Enthalpies=	-3512.761032
Sum of electronic and thermal Free Energies=	-3512.825136

Number of Imaginary Frequencies = 0

E (Single Point Energy) [IEFPCM$_{(DCM)}$M06-2X/6-311++G(2d,2p)] = -3515.788684

C	0.11611700	0.70990800	0.93043700
H	0.11314800	0.13979100	1.86300100
C	1.50863000	0.84279200	0.38486700
N	2.45328100	0.03878600	0.73371500

C	3.73530900	-0.08247200	0.00603900
H	4.19471100	0.89718700	-0.12571200
C	2.33300700	-1.03993000	1.76135600
H	2.41811000	-0.56407800	2.74289800
H	1.36949900	-1.53439000	1.66070300
C	3.51776400	-1.95621200	1.46591200
H	3.84762000	-2.47986000	2.36386800
H	3.23332700	-2.69951000	0.71483800
C	4.56852000	-1.00195800	0.89737500
H	5.04003300	-0.42501400	1.69896400
H	5.34513900	-1.49763300	0.31510000
C	1.69072200	1.88865900	-0.67401500
H	2.74110900	2.09502200	-0.87821100
H	1.25469200	1.48674100	-1.59682900
C	-0.66756400	-0.13313500	-0.13587500
H	-0.71469200	0.42289800	-1.08063600
C	-2.11132200	-0.43020400	0.32272600
O	-0.00272300	-1.36047300	-0.31592500
C	3.47457200	-0.70711100	-1.41439600
O	2.28181000	-0.96283200	-1.73023300
O	4.50542300	-0.87506300	-2.08198100
H	0.80954500	-1.23270700	-0.86467300
C	-2.68381100	-1.70966500	-0.26719200
H	-1.98894500	-2.51204300	0.00172600
C	-4.07828300	-2.06400600	0.24442400
H	-2.67637100	-1.63927400	-1.36178500
C	-4.49849700	-3.46252400	-0.20312400
H	-5.50487900	-3.70558400	0.14813500

H	-4.49684000	-3.53678400	-1.29556700
H	-3.81224300	-4.22251100	0.18397700
H	-4.80514700	-1.32726300	-0.11222400
H	-4.08431900	-2.00471800	1.34068700
H	-2.18996900	-0.44218100	1.41403300
C	0.96433500	3.18682100	-0.28880200
C	-0.44018800	2.11274100	1.22069000
H	1.49069300	3.68377200	0.53866100
H	0.94484400	3.86082500	-1.14547100
H	-1.48191400	2.05837700	1.53437500
H	0.14059500	2.56255600	2.04002100
O	-0.37698700	2.93645100	0.07769500
Br	-3.26276500	1.07194300	-0.23813900

(*R*)-**TS3**$_{G}$-Br-Pre

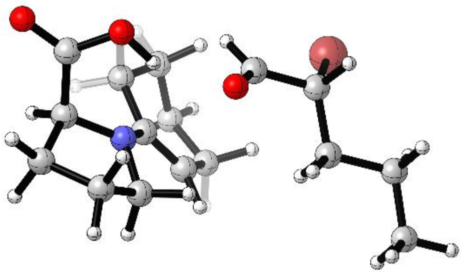

- Thermochemistry -

Zero-point correction= 0.408765 (Hartree/Particle)
Thermal correction to Energy= 0.428420
Thermal correction to Enthalpy= 0.429285
Thermal correction to Gibbs Free Energy= 0.360779
Sum of electronic and zero-point Energies= -3476.855740
Sum of electronic and thermal Energies= -3476.836085
Sum of electronic and thermal Enthalpies= -3476.835220
Sum of electronic and thermal Free Energies= -3476.903726

Number of Imaginary Frequencies = 0

E (Single Point Energy) [IEFPCM$_{(DCM)}$M06-2X/6-311++G(2d,2p)] = -3479.870365

C	-0.09151900	0.43628500	-1.78543900
H	0.07844400	-0.56205100	-2.17786500
C	-1.16161800	0.66312500	-0.99300200
N	-2.04631500	-0.35436400	-0.61926100
C	-3.43380200	-0.05993100	-0.26379100
H	-3.81763300	0.84339700	-0.74605500
C	-1.92455000	-1.67876800	-1.22968000
H	-0.98486200	-2.14943200	-0.92300200
H	-1.93084100	-1.60155700	-2.32891100
C	-3.16091800	-2.41316600	-0.72359900
H	-2.99215100	-2.78147200	0.29445700
H	-3.42997000	-3.26213600	-1.35436500
C	-4.21873400	-1.30673400	-0.73304900
H	-4.56961800	-1.13335300	-1.75422400
H	-5.08690500	-1.50971700	-0.10294300
C	-1.43790800	2.02825600	-0.39708400
H	-2.29372400	2.48605100	-0.91085400
H	-1.73691300	1.91571100	0.65387300
C	0.36805400	2.91322700	-1.90155400
C	0.57234700	-0.46312200	1.32676100
H	0.22510300	0.57734300	1.45748800
C	2.05387500	-0.69392200	1.10175200
O	-0.18955800	-1.40200200	1.41282600
C	-3.62972800	0.13921200	1.24078700
O	-2.68694100	-0.34939100	2.04301400
O	-4.61798100	0.67711800	1.69147900

H	-1.93806300	-0.73199700	1.53162100
C	2.35220100	-1.55298000	-0.12003400
H	2.10050600	-0.98571800	-1.02248500
H	2.44263100	-1.17050000	2.00711500
C	3.79385400	-2.04886400	-0.18522500
H	4.03810100	-2.56986000	0.74879200
H	1.66707900	-2.40977700	-0.07000000
H	4.47058700	-1.19029100	-0.25624700
C	4.01081500	-2.98173000	-1.37431900
H	5.04776700	-3.32367200	-1.42306600
H	3.36701700	-3.86414200	-1.30361700
H	3.78012800	-2.47097900	-2.31486300
C	-0.22657500	2.95684700	-0.49559300
C	0.88235500	1.50687300	-2.21217700
H	-0.52471500	3.97410000	-0.22453600
H	0.53888200	2.63994800	0.22426800
H	-0.40852000	3.18871900	-2.62745000
H	1.17798200	3.64267800	-2.00176100
H	1.85304300	1.35270100	-1.71955100
H	1.07306000	1.40726700	-3.28740100
Br	2.96127800	1.03130900	1.00741000

(*R*)-**TS3**$_G$-Br

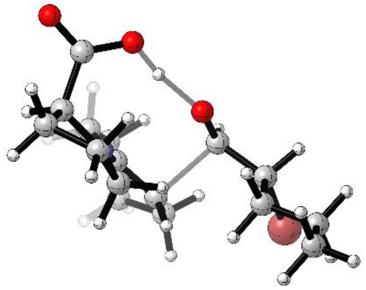

- Thermochemistry -

Zero-point correction=	0.408923 (Hartree/Particle)
Thermal correction to Energy=	0.426840
Thermal correction to Enthalpy=	0.427705
Thermal correction to Gibbs Free Energy=	0.364378
Sum of electronic and zero-point Energies=	-3476.842064
Sum of electronic and thermal Energies=	-3476.824147
Sum of electronic and thermal Enthalpies=	-3476.823282
Sum of electronic and thermal Free Energies=	-3476.886609

Number of Imaginary Frequencies = 1

E (Single Point Energy) [IEFPCM$_{(DCM)}$M06-2X/6-311++G(2d,2p)] = -3479.854643

C	0.07725800	1.12921400	-0.81403500
H	0.58536200	0.38797900	-1.42542300
C	-1.29402000	0.95166500	-0.60534600
N	-1.87300400	-0.24761900	-0.74321600
C	-3.27879100	-0.52789000	-0.43863900
H	-3.92925800	0.26760000	-0.80976100
C	-1.21762200	-1.39440000	-1.39580800
H	-0.24134700	-1.57181300	-0.95316600
H	-1.09633300	-1.17408300	-2.46488100
C	-2.18929200	-2.54440900	-1.15388800
H	-2.00116300	-2.98382400	-0.16808700

H	-2.09526500	-3.32904500	-1.90587100
C	-3.55147700	-1.84942700	-1.17784800
H	-3.84505600	-1.62658800	-2.20770600
H	-4.35316200	-2.41609000	-0.70310900
C	-2.17731500	2.06484900	-0.08647700
H	-2.97487500	2.20879500	-0.82737100
H	-2.67002800	1.74442800	0.83899700
C	-0.43048200	3.61369200	-0.98079100
C	0.62015200	0.19460000	1.05354000
H	0.58960700	1.18808400	1.52427300
C	2.03185000	-0.36988600	0.84652500
O	-0.22679000	-0.69313900	1.36403800
C	-3.60023200	-0.65886300	1.06277300
O	-2.62861700	-0.65250300	1.94184000
O	-4.76820900	-0.76647400	1.39019900
H	-1.64410600	-0.59776100	1.60535700
C	2.12508200	-1.63289700	0.00930700
H	1.95110400	-1.39181600	-1.04731700
H	2.37974000	-0.58215600	1.86368100
C	3.45003500	-2.37965700	0.15594800
H	3.65883400	-2.52798400	1.22287100
H	1.30763400	-2.28273000	0.34535800
H	4.26479500	-1.76634600	-0.24273500
C	3.41666100	-3.72922900	-0.55770900
H	4.37108600	-4.25282500	-0.45839400
H	2.63351500	-4.37238600	-0.14377700
H	3.21477500	-3.59936300	-1.62616100
C	-1.44872200	3.39064900	0.13077000

C	0.64830200	2.54043800	-0.86198700
H	-2.18723100	4.19564100	0.17630700
H	-0.92741900	3.37948700	1.09609300
H	-0.92726100	3.55437200	-1.95836000
H	0.02155600	4.60685300	-0.90094500
H	1.22399700	2.75323100	0.04730500
H	1.36198600	2.60521300	-1.68873400
Br	3.32400400	0.95493400	0.20935500

(*R*)-**TS3**$_{G}$-Br-P

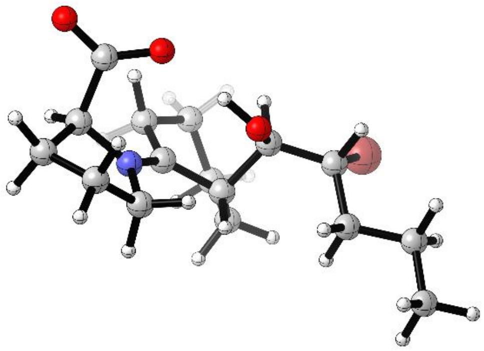

- Thermochemistry -

Zero-point correction=	0.413638 (Hartree/Particle)
Thermal correction to Energy=	0.431757
Thermal correction to Enthalpy=	0.432622
Thermal correction to Gibbs Free Energy=	0.368487
Sum of electronic and zero-point Energies=	-3476.868238
Sum of electronic and thermal Energies=	-3476.850119
Sum of electronic and thermal Enthalpies=	-3476.849254
Sum of electronic and thermal Free Energies=	-3476.913388

Number of Imaginary Frequencies = 0

E (Single Point Energy) [IEFPCM$_{(DCM)}$M06-2X/6-311++G(2d,2p)] = -3479.885557

C	0.09029200	0.11009300	-0.86990900
H	0.13206800	-0.89892100	-1.28561600

C	-1.33288300	0.55209000	-0.74166400
N	-2.30703000	-0.29347200	-0.74743600
C	-3.66781000	0.04678600	-0.29412000
H	-3.98595600	1.00155500	-0.71539400
C	-2.20150100	-1.75637900	-1.03181100
H	-1.35925900	-2.17756700	-0.48723300
H	-2.04788300	-1.86498100	-2.10962200
C	-3.55384800	-2.31340400	-0.58841500
H	-3.51036100	-2.59831900	0.46712500
H	-3.83118600	-3.19232600	-1.17151500
C	-4.50893200	-1.13270000	-0.77730400
H	-4.76558400	-1.00635800	-1.83377700
H	-5.42763200	-1.21375600	-0.19646300
C	-1.60000200	2.01761800	-0.52458900
H	-2.22408700	2.35123800	-1.36641300
H	-2.20270700	2.14043000	0.38141100
C	0.63901700	2.56113100	-1.59623900
C	0.56773900	-0.04153500	0.62009700
H	0.41488000	0.89079700	1.17195600
C	2.03848100	-0.44391900	0.77091900
O	-0.17114600	-1.07831400	1.22731900
C	-3.67369300	0.16229900	1.27734800
O	-2.57680900	0.00801900	1.87871000
O	-4.79146100	0.40193400	1.75709300
H	-1.04890100	-0.72452700	1.51286000
C	2.50301800	-1.55108400	-0.15592000
H	2.52978600	-1.19343200	-1.19186700
H	2.17090300	-0.73691200	1.81363000

C	3.85295400	-2.15853500	0.21681300
H	3.83688800	-2.44413400	1.27577300
H	1.73155300	-2.33364500	-0.10338600
H	4.63614700	-1.40060600	0.10561900
C	4.17837100	-3.37619500	-0.64531900
H	5.15304200	-3.79689600	-0.38487100
H	3.42609000	-4.16070400	-0.51512200
H	4.20183100	-3.10557000	-1.70617000
C	-0.33027900	2.87023200	-0.45235800
C	0.84831300	1.04766900	-1.83315200
H	-0.62533800	3.92203500	-0.48831800
H	0.16525600	2.72925800	0.51204500
H	0.25050700	3.01271100	-2.51503100
H	1.59788800	3.04440400	-1.38984100
H	1.90830300	0.79305300	-1.81371000
H	0.49481800	0.78747100	-2.83602400
Br	3.19280100	1.14319700	0.59859400

(*S*)-**TS3**$_{G}$-Br-Pre

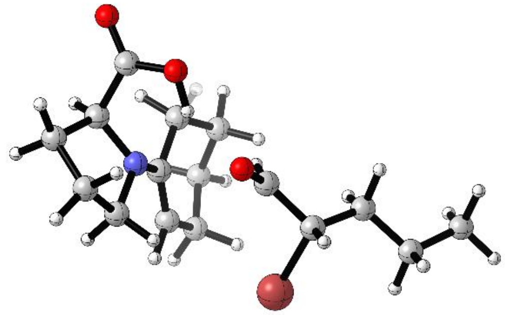

- Thermochemistry -

Zero-point correction= 0.408526 (Hartree/Particle)
Thermal correction to Energy= 0.428447

Thermal correction to Enthalpy= 0.429312
Thermal correction to Gibbs Free Energy= 0.358896
Sum of electronic and zero-point Energies= -3476.854299
Sum of electronic and thermal Energies= -3476.834378
Sum of electronic and thermal Enthalpies= -3476.833513
Sum of electronic and thermal Free Energies= -3476.903929

Number of Imaginary Frequencies = 0

E (Single Point Energy) [IEFPCM$_{(DCM)}$M06-2X/6-311++G(2d,2p)] = -3479.868737

C	0.52666300	1.66788800	1.30629400
H	0.39858800	1.11356600	2.23075600
C	1.35301500	1.19932400	0.35356900
N	2.04314900	-0.02274500	0.47703800
C	3.40994100	-0.12228400	-0.04490700
H	3.93672600	0.83724300	-0.04640100
C	1.91380600	-0.79169600	1.71528200
H	0.88862300	-1.16139100	1.82116600
H	2.15050200	-0.17224600	2.59584300
C	2.95061300	-1.89338800	1.54224800
H	2.56796700	-2.66904100	0.86960700
H	3.22727400	-2.36347200	2.48762800
C	4.11296200	-1.13223100	0.89793400
H	4.66110400	-0.57325000	1.66118800
H	4.82499100	-1.76868000	0.36816400
C	1.53458500	1.90516100	-0.97519900
H	2.51209300	2.40597800	-0.99764200
H	1.55342900	1.16008800	-1.78144700
C	0.17697100	3.78811800	-0.00777400
C	-0.76712700	-1.15056800	-0.65468700
H	-0.45195100	-0.12446100	-0.91956100
C	-2.21945500	-1.30488700	-0.28800900

O	0.01949400	-2.07327000	-0.65699300
C	3.45853500	-0.64484800	-1.47887100
O	2.44211600	-1.40637400	-1.87737800
O	4.40227000	-0.43179700	-2.20948500
H	1.73635500	-1.45859300	-1.19428500
C	-3.13099200	-0.60553200	-1.28118400
H	-2.94492300	-1.07172200	-2.25921200
H	-2.46894000	-2.35450600	-0.12965400
C	-4.61778300	-0.70798600	-0.95123400
H	-4.81353100	-0.19272300	-0.00520100
H	-2.83151800	0.44719800	-1.36324500
H	-4.87969100	-1.76199600	-0.79842400
C	-5.48073300	-0.11026300	-2.05989100
H	-6.54225900	-0.17138600	-1.80725700
H	-5.23281700	0.94403900	-2.21962600
H	-5.32769700	-0.63887100	-3.00597900
C	0.42774400	2.92734900	-1.24370400
C	-0.30765900	2.91254200	1.14667800
H	0.69931200	3.54290300	-2.10655800
H	-0.50105100	2.40135100	-1.50573200
H	1.11254500	4.28674700	0.27792200
H	-0.55560000	4.57194000	-0.22481200
H	-1.35820700	2.62794000	0.98059000
H	-0.29597900	3.48126900	2.08361300
Br	-2.32357000	-0.44306800	1.47831000

(*S*)-**TS3**$_G$-Br

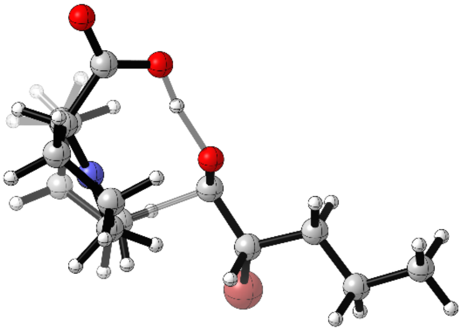

- Thermochemistry -

Zero-point correction=	0.408667 (Hartree/Particle)
Thermal correction to Energy=	0.426697
Thermal correction to Enthalpy=	0.427562
Thermal correction to Gibbs Free Energy=	0.363619
Sum of electronic and zero-point Energies=	-3476.841290
Sum of electronic and thermal Energies=	-3476.823260
Sum of electronic and thermal Enthalpies=	-3476.822395
Sum of electronic and thermal Free Energies=	-3476.886337

Number of Imaginary Frequencies = 1

E (Single Point Energy) [IEFPCM$_{(DCM)}$M06-2X/6-311++G(2d,2p)] = -3479.854911

C	0.20711100	1.38656600	0.66043000
H	-0.21875300	0.93684400	1.55328500
C	1.51950900	1.02726800	0.34070800
N	2.05004900	-0.12083100	0.77922600
C	3.37780800	-0.61325300	0.40288800
H	4.11811000	0.19004200	0.42819300
C	1.43929800	-0.95061500	1.83545800
H	0.39663900	-1.15594000	1.60221200
H	1.50076900	-0.41275400	2.79068300
C	2.29690200	-2.21127000	1.83246200

H	1.93696700	-2.89833900	1.05907400
H	2.27376500	-2.72760400	2.79310800
C	3.68388300	-1.67797100	1.47080500
H	4.14068400	-1.19142200	2.33741100
H	4.37210800	-2.43615000	1.09569200
C	2.36177700	1.83758900	-0.61898600
H	3.25466100	2.15760400	-0.06498900
H	2.71693600	1.19835900	-1.43513100
C	0.81866700	3.73473900	-0.09259000
C	-0.68054900	-0.07745200	-0.64917400
H	-0.77841700	0.69481100	-1.42745000
C	-1.97337300	-0.45627400	0.07984400
O	0.09930500	-1.06152100	-0.80422000
C	3.46561400	-1.21176800	-1.01482200
O	2.37476100	-1.37979300	-1.72189400
O	4.56544700	-1.51285300	-1.44157500
H	1.46012600	-1.15094500	-1.28472900
C	-2.72862500	-1.50693800	-0.73219900
H	-2.01121500	-2.31399200	-0.92155200
H	-1.75560100	-0.82801800	1.08302900
C	-3.95926100	-2.07889100	-0.03158700
H	-4.72721100	-1.30429600	0.06077300
H	-3.00315900	-1.07927900	-1.70497000
H	-3.68443000	-2.37255900	0.98966800
C	-4.52286100	-3.28207900	-0.78498300
H	-5.41509400	-3.67553100	-0.29069500
H	-4.79975700	-3.00536900	-1.80760200
H	-3.78553400	-4.08886700	-0.84477100

C	1.64937600	3.06679900	-1.18205800
C	-0.30039800	2.77918400	0.31301600
H	2.39635400	3.74695900	-1.60024000
H	0.98805300	2.77073600	-2.00609100
H	1.45704900	3.96792400	0.77003600
H	0.39331500	4.67705800	-0.45064300
H	-1.01222300	2.73035700	-0.52099500
H	-0.86448100	3.17095400	1.16458700
Br	-3.14129600	1.09385100	0.34125100

(*S*)-**TS3**$_{G}$-Br-P

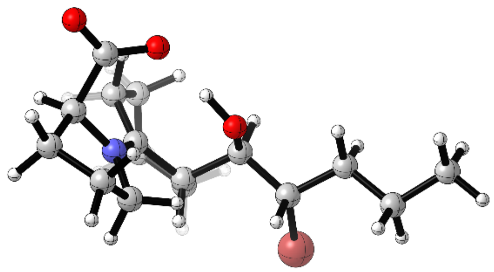

- Thermochemistry -

Zero-point correction=	0.412288 (Hartree/Particle)
Thermal correction to Energy=	0.430494
Thermal correction to Enthalpy=	0.431359
Thermal correction to Gibbs Free Energy=	0.366119
Sum of electronic and zero-point Energies=	-3476.864630
Sum of electronic and thermal Energies=	-3476.846425
Sum of electronic and thermal Enthalpies=	-3476.845559
Sum of electronic and thermal Free Energies=	-3476.910799

Number of Imaginary Frequencies = 0

E (Single Point Energy) [IEFPCM$_{(DCM)}$M06-2X/6-311++G(2d,2p)] = -3479.881840

C	0.03355600	1.11122400	0.33191400
H	-0.13500000	0.88367700	1.38857200

C	1.50384600	0.95554800	0.04605500
N	2.19512300	0.08801500	0.71031100
C	3.57198100	-0.30950300	0.35304100
H	4.17452000	0.57507500	0.13946000
C	1.69503500	-0.73122700	1.85387800
H	0.72546600	-1.15528000	1.60437600
H	1.60983400	-0.06585700	2.71886200
C	2.78307600	-1.78407900	2.04940000
H	2.58158100	-2.64849300	1.40891900
H	2.82675500	-2.12328900	3.08495200
C	4.05323200	-1.06838100	1.58807900
H	4.39942400	-0.36583400	2.35258600
H	4.86933800	-1.74172000	1.32795600
C	2.17680300	1.72869800	-1.05699700
H	3.07212400	2.19210500	-0.62269500
H	2.52576200	1.01310300	-1.80918400
C	0.53413500	3.50801000	-0.53759600
C	-0.70982900	-0.00243000	-0.51761100
H	-0.93383900	0.44061400	-1.50048100
C	-2.01602000	-0.51348900	0.11771300
O	0.01648000	-1.19691000	-0.64868000
C	3.57781600	-1.21155700	-0.93816400
O	2.49305000	-1.35487000	-1.56358100
O	4.69235100	-1.67758600	-1.21428900
H	0.90884600	-1.11799000	-1.07659100
C	-2.83024300	-1.33890600	-0.86889200
H	-2.12917600	-2.04173600	-1.33405100
H	-1.75478200	-1.09823300	1.00363600

C	-3.98174400	-2.12007400	-0.23978200
H	-4.72182900	-1.42229200	0.16620500
H	-3.20595600	-0.67648300	-1.65914600
H	-3.59805300	-2.70194600	0.60781800
C	-4.64726500	-3.05221400	-1.25001800
H	-5.48010300	-3.59722900	-0.79780900
H	-5.03899900	-2.48611700	-2.10161100
H	-3.93297700	-3.78610900	-1.63658300
C	1.29772200	2.82033000	-1.66118000
C	-0.50395800	2.53910500	0.02503400
H	1.93488300	3.51690700	-2.21114300
H	0.59080200	2.39325000	-2.38275600
H	1.23896300	3.82599400	0.24251600
H	0.02347900	4.40679700	-0.89352300
H	-1.30856400	2.45454900	-0.71176400
H	-0.95576200	2.94603000	0.93122600
Br	-3.15704300	0.92608600	0.81232100

(*R*)-**TS3**$_{O}$-Br-Pre

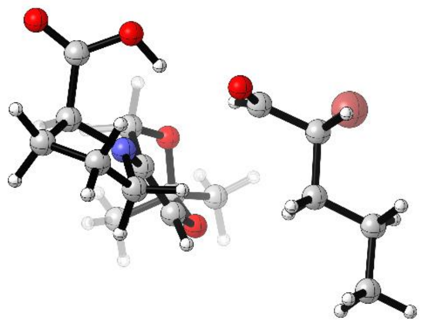

- Thermochemistry -

Zero-point correction= 0.416746 (Hartree/Particle)
Thermal correction to Energy= 0.438418

Thermal correction to Enthalpy= 0.439283
Thermal correction to Gibbs Free Energy= 0.365694
Sum of electronic and zero-point Energies= -3627.233448
Sum of electronic and thermal Energies= -3627.211775
Sum of electronic and thermal Enthalpies= -3627.210910
Sum of electronic and thermal Free Energies= -3627.284499

Number of Imaginary Frequencies = 0

E (Single Point Energy) [IEFPCM$_{(DCM)}$M06-2X/6-311++G(2d,2p)] = -3630.303915

C	0.37610200	0.92446500	1.43158000
H	0.26364900	0.32643500	2.32713000
C	1.34951100	0.75411600	0.52133600
N	2.30336000	-0.26717100	0.59309700
C	3.65090900	-0.04915800	0.06228300
H	3.91978300	1.01081200	0.00301400
C	2.34154600	-1.10864200	1.79056900
H	1.45814600	-1.75521100	1.82158500
H	2.35379100	-0.48943000	2.70235100
C	3.65501100	-1.86819100	1.64004300
H	3.53180200	-2.70424500	0.94327900
H	4.01961400	-2.26188500	2.59013600
C	4.57569600	-0.79756200	1.04715500
H	4.89239600	-0.10199500	1.82938500
H	5.46625300	-1.19156200	0.55472000
C	1.33525300	1.60376600	-0.72539200
H	2.11836900	2.37520400	-0.71103000
H	1.49901300	0.98151500	-1.61280600
O	-0.64558400	1.83336300	1.28680400
O	0.05547500	2.19059500	-0.89908500
C	-0.45924800	2.81327100	0.26441900
C	-1.83993600	3.31352400	-0.09924900

H	-2.43180100	2.48280900	-0.49201200
H	-1.76315300	4.09557000	-0.85759500
C	0.46081600	3.91486300	0.77828300
H	0.69349600	4.60827600	-0.03393600
H	1.39111500	3.50582200	1.17967400
H	-0.04463100	4.45989300	1.57815400
C	-0.69637600	-1.11196000	-0.91528100
H	-0.56629600	-0.07298900	-1.26419300
C	-2.07534600	-1.48790000	-0.42470100
O	0.21551000	-1.91195300	-0.90271400
C	3.81447000	-0.62251700	-1.35019400
O	2.82676400	-1.38031200	-1.81525000
O	4.81328900	-0.41764700	-2.00523800
H	-2.33025800	3.71894400	0.78816300
C	-2.26014400	-1.13415400	1.04687800
H	-1.42985200	-1.61596500	1.58320000
C	-3.58564400	-1.61852300	1.62951300
H	-4.41060300	-1.08459100	1.14621400
H	-3.71522600	-2.68206700	1.39416700
H	-2.15043100	-0.05129500	1.17734900
C	-3.64044700	-1.40734500	3.14078700
H	-4.59972900	-1.73483900	3.54969400
H	-2.84862400	-1.97107200	3.64421800
H	-3.51132500	-0.34928800	3.39090200
H	2.07651700	-1.42299000	-1.17940500
H	-2.24380900	-2.55160700	-0.60191100
Br	-3.36852500	-0.53927800	-1.54132700

(*R*)-**TS3**$_{O}$-Br

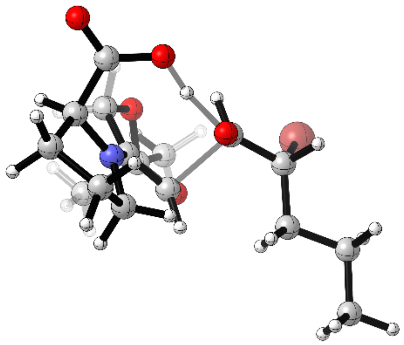

- Thermochemistry -

Zero-point correction=	0.415436 (Hartree/Particle)
Thermal correction to Energy=	0.435545
Thermal correction to Enthalpy=	0.436410
Thermal correction to Gibbs Free Energy=	0.367930
Sum of electronic and zero-point Energies=	-3627.219874
Sum of electronic and thermal Energies=	-3627.199766
Sum of electronic and thermal Enthalpies=	-3627.198901
Sum of electronic and thermal Free Energies=	-3627.267380

Number of Imaginary Frequencies = 1

E (Single Point Energy) [IEFPCM$_{(DCM)}$M06-2X/6-311++G(2d,2p)] = -3630.286244

C	-0.07179100	0.33890500	0.96471900
H	-0.23622100	-0.48738900	1.64603300
C	1.22054000	0.66260600	0.56689200
N	2.22884400	-0.20364400	0.68489000
C	3.54833000	-0.00524900	0.08356100
H	3.85697300	1.04190800	0.14314900
C	2.19477900	-1.37718200	1.58615800
H	1.34245200	-2.01529300	1.35555500
H	2.11789900	-1.01888800	2.62080500
C	3.53046400	-2.06522100	1.31075900
H	3.42630600	-2.75368100	0.46563500

H	3.88189200	-2.63222100	2.17367100
C	4.45585100	-0.90357100	0.94019100
H	4.75673100	-0.35395400	1.83674600
H	5.35196100	-1.20348300	0.39613000
C	1.42754200	1.95235800	-0.18436600
H	2.16270500	2.57974200	0.34040300
H	1.80981200	1.75455800	-1.19277100
O	-1.05814100	1.29968900	1.05892500
O	0.19937900	2.62606500	-0.35656200
C	-0.62524500	2.63549200	0.79762200
C	-1.85806300	3.43512200	0.44216400
H	-2.30120000	3.03048800	-0.46865100
H	-1.58438700	4.48078700	0.28601900
C	0.09915300	3.19277000	2.02067500
H	0.54183500	4.16186600	1.77601100
H	0.88575400	2.52445100	2.37969100
H	-0.62344300	3.32696200	2.82790000
C	-0.33777900	-0.70592300	-0.85469700
H	-0.20688400	0.18378700	-1.49012400
C	-1.79569400	-1.15737300	-0.71516200
O	0.52084400	-1.64256300	-0.89617000
C	3.63542600	-0.39996400	-1.40449000
O	2.62634900	-1.00881800	-1.96933200
O	4.66237000	-0.13462200	-2.00454500
H	-2.58268700	3.37225800	1.25665800
C	-2.23783500	-1.72626000	0.62086900
H	-1.41530100	-2.36366000	0.97809600
C	-3.51404900	-2.56083900	0.54360100

H	-4.34034900	-1.92591600	0.20491400
H	-3.38659000	-3.34711400	-0.21048400
H	-2.37151900	-0.91182300	1.34153700
C	-3.85670900	-3.18467300	1.89448000
H	-4.77594300	-3.77332500	1.83725500
H	-3.05352300	-3.84538300	2.23638900
H	-4.00047100	-2.40912900	2.65396800
H	1.75420300	-1.25534000	-1.40617300
H	-1.90973500	-1.91889600	-1.49242300
Br	-3.00566000	0.26514900	-1.30361600

(*R*)-**TS3**$_{O}$-Br-P

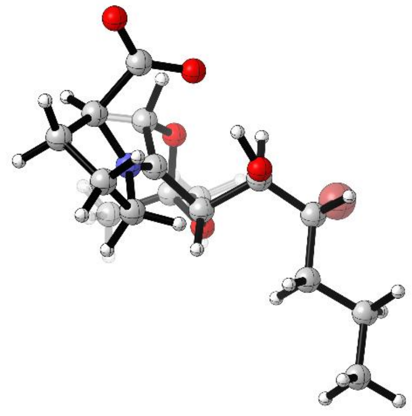

- Thermochemistry -

Zero-point correction=	0.419557 (Hartree/Particle)
Thermal correction to Energy=	0.439800
Thermal correction to Enthalpy=	0.440665
Thermal correction to Gibbs Free Energy=	0.372299
Sum of electronic and zero-point Energies=	-3627.244536
Sum of electronic and thermal Energies=	-3627.224293
Sum of electronic and thermal Enthalpies=	-3627.223428
Sum of electronic and thermal Free Energies=	-3627.291794

Number of Imaginary Frequencies = 0

E (Single Point Energy) [IEFPCM$_{(DCM)}$M06-2X/6-311++G(2d,2p)] = -3630.315445

C	-0.06243100	0.09287700	0.66117900
H	-0.10425600	-0.68987200	1.42247900
C	1.35275400	0.53931800	0.43104700
N	2.35634500	-0.22456800	0.67127200
C	3.69821300	0.01743800	0.10775100
H	3.96460000	1.07264500	0.20188300
C	2.31842900	-1.53084000	1.40117200
H	1.49637100	-2.13726400	1.02693900
H	2.17057600	-1.29656500	2.45961900
C	3.69696400	-2.13257500	1.13058400
H	3.66677800	-2.73645400	0.21867300
H	4.01811700	-2.76928900	1.95541900
C	4.59411700	-0.90926700	0.92489200
H	4.84584100	-0.44558500	1.88354700
H	5.51339800	-1.12796600	0.38236600
C	1.52062700	1.87044500	-0.25029400
H	2.28298200	2.46556500	0.27048900
H	1.85070000	1.70632200	-1.28200000
O	-0.94012800	1.12463800	1.05486000
O	0.28377700	2.54280800	-0.30357300
C	-0.46835500	2.46499500	0.89707300
C	-1.68588900	3.34149600	0.70720000
H	-2.19290800	3.06107100	-0.21658300
H	-1.38005400	4.38885800	0.65911600
C	0.34571000	2.86819400	2.12537600
H	0.80784500	3.84590400	1.96579200

H	1.12740900	2.14462000	2.37487300
H	-0.32760900	2.92932800	2.98227600
C	-0.48778200	-0.53339700	-0.71360900
H	-0.36220800	0.22267100	-1.49934800
C	-1.94778500	-1.00979900	-0.70869300
O	0.30419300	-1.66608700	-0.96236900
C	3.69452300	-0.35332600	-1.42760400
O	2.58405100	-0.63661000	-1.95170700
O	4.81260700	-0.30236300	-1.95407500
H	-2.36781000	3.20524900	1.54899200
C	-2.40630600	-1.67324900	0.57671100
H	-1.62670100	-2.40413900	0.84131400
C	-3.74203700	-2.40425900	0.46576700
H	-4.54004300	-1.67586000	0.28680700
H	-3.71714700	-3.06751300	-0.40777600
H	-2.45018000	-0.93099300	1.38275000
C	-4.04499000	-3.21281000	1.72531200
H	-5.01087400	-3.71886700	1.64894900
H	-3.27726500	-3.97428600	1.89623800
H	-4.07506200	-2.56223100	2.60572300
H	1.15478400	-1.37342700	-1.37448300
H	-2.03938000	-1.69925100	-1.54982500
Br	-3.15808300	0.45796500	-1.19346900

(*S*)-**TS3**$_{O}$-Br-Pre

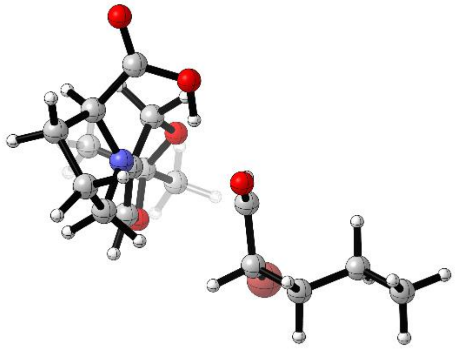

- Thermochemistry -

Zero-point correction=	0.416072 (Hartree/Particle)
Thermal correction to Energy=	0.437835
Thermal correction to Enthalpy=	0.438700
Thermal correction to Gibbs Free Energy=	0.364450
Sum of electronic and zero-point Energies=	-3627.232987
Sum of electronic and thermal Energies=	-3627.211224
Sum of electronic and thermal Enthalpies=	-3627.210359
Sum of electronic and thermal Free Energies=	-3627.284609

Number of Imaginary Frequencies = 0

E (Single Point Energy) [IEFPCM$_{(DCM)}$M06-2X/6-311++G(2d,2p)] = -3630.301967

C	0.57325200	1.32810300	1.19135000
H	0.34710500	0.97042500	2.18885400
C	1.40313300	0.70979900	0.33163300
N	2.04207300	-0.50988800	0.59671400
C	3.42198800	-0.70322000	0.13754700
H	3.95692900	0.24141200	-0.00821300
C	1.83727000	-1.13953300	1.90274700
H	0.81212500	-1.51761800	1.97819700
H	2.00886500	-0.42121000	2.72075000
C	2.89649200	-2.23510900	1.92605500
H	2.56270000	-3.09515800	1.33563700

H	3.12217600	-2.57664900	2.93749200
C	4.08519400	-1.54138900	1.25459800
H	4.56941100	-0.86255100	1.96197300
H	4.84098600	-2.22400500	0.86241500
C	1.55825300	1.27344300	-1.05927000
H	2.54858200	1.72536400	-1.21744700
H	1.43253100	0.47779800	-1.80241600
O	-0.09797400	2.48462600	0.89492900
O	0.52955700	2.21285600	-1.32244800
C	0.33879600	3.15863000	-0.28645700
C	-0.80658900	4.04454800	-0.72351300
H	-1.07650400	4.72381600	0.08767600
H	-1.66746000	3.42172500	-0.97581700
C	1.61195500	3.94213500	0.01753300
H	2.37367500	3.31066000	0.48098700
H	1.37625500	4.75407900	0.70852300
H	2.01152100	4.36622500	-0.90732200
C	-0.98826600	-0.86596100	-0.51354400
H	-0.89003300	0.00916500	-1.17956600
C	-2.07712500	-0.83182700	0.54693300
O	-0.26664100	-1.83544800	-0.61223000
C	3.49108600	-1.45382300	-1.19651200
O	2.37132600	-2.02512700	-1.62941000
O	4.52762400	-1.55030500	-1.81654100
H	1.61957800	-1.82632400	-1.02783800
H	-0.50914700	4.62834900	-1.59721800
C	-2.95434700	-2.07987900	0.52942200
H	-3.65137900	-2.02636400	1.37204800

H	-1.59375200	-0.72644300	1.52071300
C	-3.71335200	-2.30596300	-0.77662400
H	-3.00785000	-2.30065200	-1.61734500
H	-2.27947200	-2.92463200	0.71759700
H	-4.40787700	-1.47554000	-0.94142400
C	-4.47783200	-3.62755700	-0.76129700
H	-3.79506200	-4.47362700	-0.63432900
H	-5.19814100	-3.65024200	0.06300000
H	-5.02878200	-3.77483500	-1.69362900
Br	-3.09948800	0.81396700	0.32147700

(*S*)-**TS3**$_{O}$-Br

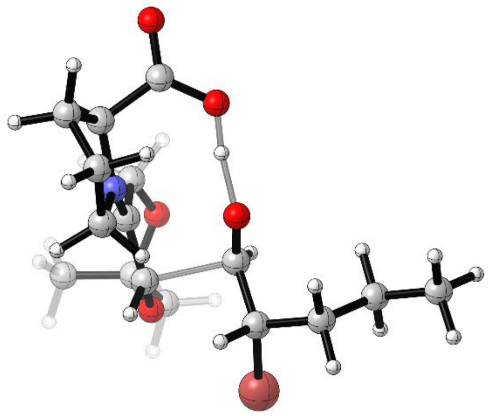

- Thermochemistry -

Zero-point correction=	0.415374 (Hartree/Particle)
Thermal correction to Energy=	0.435431
Thermal correction to Enthalpy=	0.436296
Thermal correction to Gibbs Free Energy=	0.368051
Sum of electronic and zero-point Energies=	-3627.218624
Sum of electronic and thermal Energies=	-3627.198567
Sum of electronic and thermal Enthalpies=	-3627.197702
Sum of electronic and thermal Free Energies=	-3627.265946

Number of Imaginary Frequencies = 1

E (Single Point Energy) [IEFPCM$_{(DCM)}$M06-2X/6-311++G(2d,2p)] = -3630.285366

C	0.05893600	1.06216400	0.77796900
H	-0.16604600	0.78683800	1.80432100
C	1.34190100	0.87039500	0.26164400
N	2.15738700	-0.06519000	0.73609600
C	3.45253600	-0.40595600	0.13799500
H	4.00285200	0.49812800	-0.13847000
C	1.93211700	-0.76286900	2.01825400
H	0.92521000	-1.17744800	2.04943800
H	2.06276200	-0.04537100	2.83849600
C	3.01373800	-1.83871000	2.01637000
H	2.65910000	-2.71544700	1.46447700
H	3.28409200	-2.15006600	3.02603200
C	4.16750300	-1.16613900	1.26856100
H	4.67393000	-0.44720600	1.91877100
H	4.90869400	-1.86135500	0.87372300
C	1.72795800	1.59375500	-1.00541000
H	2.60288500	2.23238400	-0.81315800
H	1.99477800	0.87735000	-1.78877600
O	-0.70710200	2.13062100	0.34791800
O	0.64484000	2.33786100	-1.51303300
C	-0.07277300	3.05815200	-0.52522600
C	-1.18490100	3.78344200	-1.24973500
H	-1.83667200	4.27356600	-0.52390400
H	-1.76702000	3.06184800	-1.82685900
C	0.82829700	3.99815800	0.27054000
H	1.51759100	3.45559500	0.92274300
H	0.20592600	4.63856500	0.89857500

H	1.40619600	4.62422700	-0.41434000
C	-0.69451200	-0.64521800	-0.17824900
H	-0.92503900	-0.03752600	-1.06697400
C	-1.88413200	-1.00150000	0.71842600
O	0.17453900	-1.57522300	-0.23202200
C	3.37351300	-1.25890200	-1.14668500
O	2.21776300	-1.70270200	-1.56279400
O	4.41518300	-1.47746900	-1.73946000
H	1.35286300	-1.55961100	-0.96019000
H	-0.76362600	4.53440200	-1.92130200
C	-2.50343200	-2.33633900	0.29978200
H	-3.32906100	-2.55927600	0.98481700
H	-1.56495100	-1.06613500	1.75989200
C	-2.98760100	-2.40128700	-1.14698000
H	-2.15476000	-2.16959900	-1.82184400
H	-1.72763600	-3.09425800	0.45981700
H	-3.75391500	-1.63582600	-1.31155200
C	-3.54887600	-3.77959700	-1.49006000
H	-2.78675700	-4.55480100	-1.36013600
H	-4.39486500	-4.02870300	-0.84085300
H	-3.89640900	-3.81893400	-2.52573500
Br	-3.24900700	0.40208600	0.70263600

(*S*)-**TS3**$_{O}$-Br-P

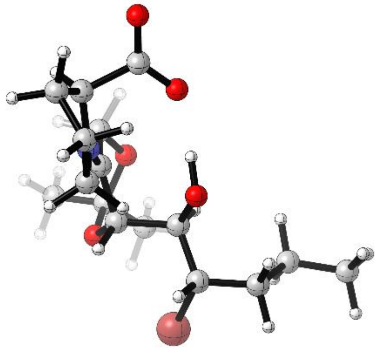

- Thermochemistry -

Zero-point correction=	0.420095 (Hartree/Particle)
Thermal correction to Energy=	0.440117
Thermal correction to Enthalpy=	0.440982
Thermal correction to Gibbs Free Energy=	0.373188
Sum of electronic and zero-point Energies=	-3627.243178
Sum of electronic and thermal Energies=	-3627.223155
Sum of electronic and thermal Enthalpies=	-3627.222290
Sum of electronic and thermal Free Energies=	-3627.290085

Number of Imaginary Frequencies = 0

E (Single Point Energy) [IEFPCM$_{(DCM)}$M06-2X/6-311++G(2d,2p)] = -3630.313357

C	0.00981300	0.65707300	0.71609500
H	0.01494100	0.45437100	1.79214100
C	1.42009900	0.77764100	0.19882400
N	2.41188400	0.15625300	0.71816300
C	3.73735200	0.07407600	0.07497300
H	4.04750300	1.06102500	-0.27600800
C	2.35423300	-0.71133100	1.93269000
H	1.48578900	-1.36428700	1.86945200
H	2.27384100	-0.04660100	2.79797500
C	3.68940000	-1.45423500	1.90268900
H	3.58826200	-2.37783100	1.32483700

H	4.02212900	-1.71060500	2.90888900
C	4.62777600	-0.48103400	1.18368000
H	4.94357700	0.32252800	1.85596100
H	5.50977600	-0.95884100	0.75862000
C	1.57038200	1.66123600	-1.01146000
H	2.09309300	2.58582300	-0.72569100
H	2.15068600	1.15187500	-1.78273000
O	-0.60958800	1.91886400	0.58799900
O	0.29754800	1.90100600	-1.56236900
C	-0.61586100	2.55830000	-0.69893200
C	-1.97883000	2.45022500	-1.35917000
H	-2.73458900	2.88708100	-0.70338000
H	-2.24568600	1.40961600	-1.55012700
C	-0.22928300	4.00910300	-0.45063900
H	0.73892500	4.08692100	0.04848600
H	-0.97754300	4.47269600	0.19585400
H	-0.19468400	4.54876900	-1.39952200
C	-0.67932800	-0.54695400	-0.01979900
H	-1.01835900	-0.19437100	-0.99950000
C	-1.84966000	-1.13554900	0.77093200
O	0.21521000	-1.62736000	-0.13888200
C	3.65502400	-0.89608700	-1.17157200
O	2.51067300	-1.26904000	-1.54040500
O	4.75854600	-1.16271800	-1.66522600
H	0.97470300	-1.44370800	-0.74307900
H	-1.96299900	2.99530900	-2.30615300
C	-2.50374500	-2.32660500	0.07946700
H	-3.35140800	-2.65192100	0.69269800

H	-1.49295100	-1.43094700	1.76070300
C	-2.96532800	-2.07360300	-1.35461000
H	-2.09608400	-1.86178500	-1.98817600
H	-1.76710800	-3.13768900	0.08635800
H	-3.60879900	-1.18560600	-1.38426200
C	-3.71671300	-3.27597800	-1.92263100
H	-3.08543400	-4.17043200	-1.90965700
H	-4.61348800	-3.48980800	-1.33202300
H	-4.02679100	-3.09775900	-2.95553600
Br	-3.20233100	0.23567800	1.15041700

(*R*)-**TS3**$_{T}$-Br-Pre

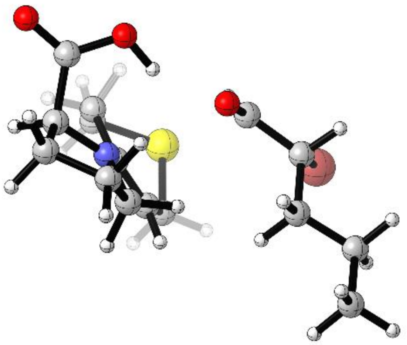

- Thermochemistry -

Zero-point correction= 0.381545 (Hartree/Particle)
Thermal correction to Energy= 0.401448
Thermal correction to Enthalpy= 0.402313
Thermal correction to Gibbs Free Energy= 0.333150
Sum of electronic and zero-point Energies= -3835.740937
Sum of electronic and thermal Energies= -3835.721034
Sum of electronic and thermal Enthalpies= -3835.720169
Sum of electronic and thermal Free Energies= -3835.789332

Number of Imaginary Frequencies = 0

E (Single Point Energy) [IEFPCM$_{(DCM)}$M06-2X/6-311++G(2d,2p)] = -3838.751494

C	0.16611900	-0.73447000	-1.56665300
H	-0.13813400	0.17306800	-2.07953700
C	1.31562900	-0.73116800	-0.85489900
N	2.02960100	0.44574900	-0.62923100
C	3.46648800	0.45713400	-0.36385800
H	4.01195700	-0.31267200	-0.91817900
C	1.59154300	1.70444500	-1.23101100
H	1.58211200	1.63406000	-2.33062600
H	0.58048700	1.94644200	-0.89221400
C	2.64240900	2.69822900	-0.75285500
H	2.68869700	3.59273800	-1.37626300
H	2.42585200	3.00390800	0.27661800
C	3.92428000	1.86487000	-0.81575200
H	4.27839600	1.80054800	-1.84823100
H	4.73956500	2.24909000	-0.19980900
C	1.86142400	-1.95437400	-0.14651900
H	2.94292500	-2.02342200	-0.30942100
H	1.71735300	-1.83245100	0.93606100
C	-0.71738800	0.27859400	1.35976500
H	-0.55785600	-0.80929900	1.45018200
C	-2.12390700	0.76618200	1.06119900
O	0.17728000	1.07889000	1.53845400
C	3.80629200	0.24527200	1.11340900
O	2.84275600	0.46822200	2.00136400
O	4.92234000	-0.06950500	1.46674600
H	1.97992000	0.67161100	1.57380500
C	-2.18208600	1.78966700	-0.06376200
H	-1.87611200	1.31123400	-1.00193400

C	-3.54486400	2.45703300	-0.22739600
H	-1.42895700	2.55069000	0.17990800
C	-3.48864100	3.59960300	-1.23885800
H	-4.47055000	4.06255800	-1.36557600
H	-2.78799800	4.37573400	-0.91526600
H	-3.15978600	3.23622000	-2.21800200
H	-3.87778600	2.83607500	0.74688300
H	-4.28063800	1.71048300	-0.54421800
H	-2.51653400	1.19588900	1.98849600
C	1.24166800	-3.26947300	-0.60103900
C	-0.74790500	-1.91156500	-1.78203800
H	1.54407600	-3.50939200	-1.62534400
H	1.56815400	-4.08395400	0.04809600
H	-1.79097400	-1.58591700	-1.73824800
H	-0.59101400	-2.35918000	-2.77108200
S	-0.57091500	-3.21217000	-0.52499700
Br	-3.27031700	-0.76972300	0.69564500

(*R*)-**TS3**$_T$-Br

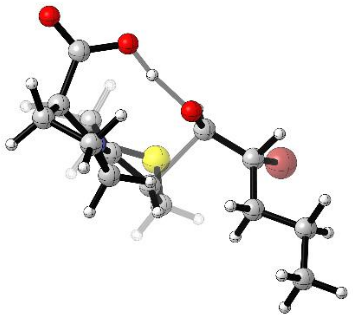

- Thermochemistry -

Zero-point correction= 0.381398 (Hartree/Particle)
Thermal correction to Energy= 0.399667

Thermal correction to Enthalpy= 0.400532
Thermal correction to Gibbs Free Energy= 0.336120
Sum of electronic and zero-point Energies= -3835.728424
Sum of electronic and thermal Energies= -3835.710155
Sum of electronic and thermal Enthalpies= -3835.709290
Sum of electronic and thermal Free Energies= -3835.773702

Number of Imaginary Frequencies = 1

E (Single Point Energy) [IEFPCM$_{(DCM)}$M06-2X/6-311++G(2d,2p)] = -3838.736601

C	-0.03723100	0.38538900	1.18289100
H	-0.26996500	-0.57105900	1.63951300
C	1.30865800	0.60493300	0.87877200
N	2.14925400	-0.43154000	0.75390200
C	3.52606500	-0.33480200	0.25621000
H	4.07488600	0.46803500	0.75164600
C	1.82801600	-1.80509500	1.20077000
H	1.85064400	-1.83421300	2.29776700
H	0.84358500	-2.10271900	0.84721000
C	2.93795800	-2.64556700	0.58092500
H	3.10812300	-3.57166500	1.13187200
H	2.67287000	-2.89858100	-0.45108600
C	4.13987400	-1.70219500	0.61174400
H	4.55597400	-1.65086600	1.62188500
H	4.94023600	-1.97445500	-0.07759600
C	1.82024300	1.96576200	0.45921500
H	2.87098300	2.06720900	0.74274700
H	1.78390200	2.01840300	-0.63878000
C	-0.47081800	-0.17020200	-0.88168000
H	-0.37695000	0.87444100	-1.20930900
C	-1.90568800	-0.70268100	-0.80780000
O	0.39061400	-1.02931100	-1.23071600

C	3.63701800	-0.06029300	-1.25371000
O	2.59441000	-0.25754600	-2.02174500
O	4.71065800	0.30803000	-1.69545000
H	1.68987700	-0.58248200	-1.59229000
C	-2.25140000	-1.64019700	0.33462600
H	-2.36638200	-1.07177100	1.26432500
C	-3.50089100	-2.48283000	0.09041000
H	-1.38832500	-2.31004600	0.45864800
C	-3.73265200	-3.47816600	1.22499200
H	-4.63244600	-4.07385300	1.05129000
H	-2.88628100	-4.16567200	1.32208900
H	-3.85406000	-2.95661400	2.18005600
H	-3.39361600	-3.01774900	-0.86100700
H	-4.36950700	-1.82289700	-0.01184100
H	-2.04327100	-1.22361100	-1.75964100
C	1.05633200	3.14628900	1.05301700
C	-0.95767700	1.49566100	1.63661700
H	1.21664400	3.20629200	2.13397500
H	1.41743100	4.07478500	0.60761100
H	-2.00041300	1.20287500	1.50557400
H	-0.81396700	1.70105700	2.70441300
S	-0.72527800	3.04868000	0.72482000
Br	-3.20064600	0.76661800	-0.92434900

(*R*)-**TS3**$_T$-Br-P

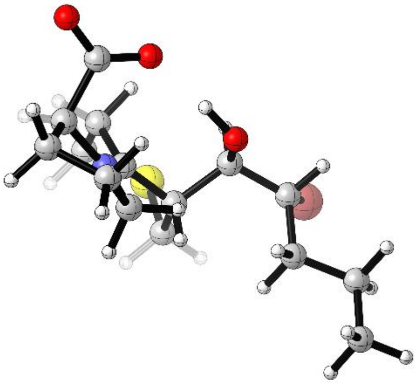

- Thermochemistry -

Zero-point correction=	0.385865 (Hartree/Particle)
Thermal correction to Energy=	0.404170
Thermal correction to Enthalpy=	0.405035
Thermal correction to Gibbs Free Energy=	0.340446
Sum of electronic and zero-point Energies=	-3835.756345
Sum of electronic and thermal Energies=	-3835.738040
Sum of electronic and thermal Enthalpies=	-3835.737175
Sum of electronic and thermal Free Energies=	-3835.801764

Number of Imaginary Frequencies = 0

E (Single Point Energy) [IEFPCM$_{(DCM)}$M06-2X/6-311++G(2d,2p)] = -3838.769308

C	-0.07624000	-0.22802700	-0.73926600
H	-0.15478700	0.70454200	-1.30101000
C	1.38024000	-0.58510000	-0.58481800
N	2.28735300	0.32335600	-0.72052700
C	3.69597300	0.15647800	-0.29385400
H	4.11147800	-0.76623600	-0.69788900
C	2.03867500	1.74464000	-1.12216800
H	1.88474500	1.75390400	-2.20522100
H	1.15747400	2.12112800	-0.60812500
C	3.31887700	2.47106500	-0.71902400

H	3.49447900	3.34248000	-1.35082700
H	3.24350600	2.80516500	0.32030500
C	4.39372500	1.39402000	-0.85373000
H	4.65469700	1.23711700	-1.90490700
H	5.30337400	1.60499300	-0.29167200
C	1.72890900	-1.97367200	-0.12385900
H	2.80470100	-2.14141200	-0.15155400
H	1.42814700	-2.03691500	0.92949500
C	-0.59226700	0.05989300	0.71289000
H	-0.56814500	-0.87035100	1.29361000
C	-2.01773300	0.63311100	0.76448500
O	0.20789700	1.05421700	1.31002300
C	3.76458000	0.09133600	1.27659900
O	2.67573400	0.12869100	1.90994200
O	4.91683200	-0.00110800	1.72318100
H	1.09435100	0.69561700	1.56624800
C	-2.33163300	1.67807900	-0.29006700
H	-2.40032400	1.20925300	-1.27965700
C	-3.59558800	2.48968700	-0.01753600
H	-1.47022600	2.36228900	-0.31122600
C	-3.75656800	3.62856700	-1.02190200
H	-4.67109300	4.19656000	-0.83288900
H	-2.91132500	4.32204600	-0.96742500
H	-3.80791800	3.23993100	-2.04438100
H	-3.54841900	2.89230000	1.00160200
H	-4.46798000	1.82861500	-0.05701100
H	-2.13304500	1.05338900	1.76453300
C	1.02749400	-3.07904600	-0.92285700

C	-0.85485600	-1.28309200	-1.53952400
H	1.34455500	-3.06650600	-1.97011400
H	1.30241500	-4.04633900	-0.49938200
H	-1.90530500	-0.99952500	-1.60459000
H	-0.46038600	-1.31991700	-2.56010400
S	-0.77902600	-2.95095500	-0.83151100
Br	-3.37192200	-0.79463900	0.72607600

(*S*)-**TS3**$_{T}$-Br-Pre

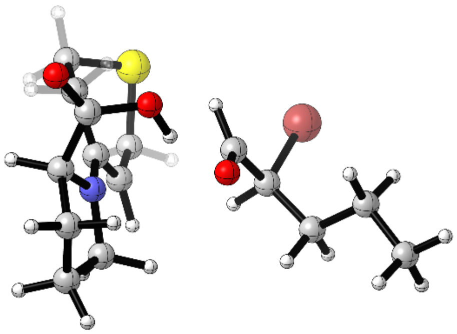

- Thermochemistry -

Zero-point correction=	0.381486 (Hartree/Particle)
Thermal correction to Energy=	0.401339
Thermal correction to Enthalpy=	0.402204
Thermal correction to Gibbs Free Energy=	0.333301
Sum of electronic and zero-point Energies=	-3835.739356
Sum of electronic and thermal Energies=	-3835.719503
Sum of electronic and thermal Enthalpies=	-3835.718638
Sum of electronic and thermal Free Energies=	-3835.787541

Number of Imaginary Frequencies = 0

E (Single Point Energy) [IEFPCM$_{(DCM)}$M06-2X/6-311++G(2d,2p)] = -3838.749560

C	0.61431800	1.18659700	1.51918800
H	0.31943600	0.48499200	2.29374500
C	1.62646200	0.85395000	0.68227400

N	2.22009200	-0.40125800	0.72909600
C	3.41035700	-0.77213100	-0.02469600
H	4.27597500	-0.13365300	0.19534200
C	1.99904700	-1.28824300	1.87752800
H	1.06231800	-1.84744500	1.75021100
H	1.92424700	-0.70119100	2.79921400
C	3.22967400	-2.19734000	1.89499700
H	3.00912900	-3.19264300	2.28450500
H	4.01096900	-1.74958400	2.51668100
C	3.67789100	-2.21886400	0.43535800
H	4.72332500	-2.49602600	0.29373400
H	3.05242800	-2.90945800	-0.14157400
C	2.13268800	1.77347500	-0.41064900
H	3.22926300	1.75643000	-0.41256700
H	1.80776900	1.39475300	-1.38854000
C	-0.90017600	-0.46139700	-0.47176800
H	-0.64780300	0.44880400	-1.04059500
C	-2.11291000	-0.38422800	0.43661000
O	-0.24120700	-1.47907100	-0.56134500
C	3.23349600	-0.76899200	-1.53973200
O	2.04126600	-1.11670200	-2.01712500
O	4.15589000	-0.52715900	-2.28699100
H	1.35968400	-1.26133500	-1.31895800
C	-2.76592100	-1.72351800	0.73391400
H	-3.52773200	-1.57472100	1.50589100
H	-1.79576800	0.09587200	1.36437300
C	-3.37121400	-2.42820300	-0.47872100
H	-2.63532000	-2.45868100	-1.29063000

H	-1.97860200	-2.35150000	1.17104900
H	-4.22402400	-1.84747600	-0.84570500
C	-3.81774700	-3.84825500	-0.13945600
H	-2.96835100	-4.45769000	0.18522000
H	-4.27220500	-4.33587100	-1.00563900
H	-4.55574000	-3.84260300	0.66954400
C	1.68386800	3.22141000	-0.26623200
C	-0.18231400	2.46458000	1.49968300
H	2.15238800	3.69319700	0.60319700
H	1.96273200	3.79142400	-1.15416300
H	-1.24200300	2.25246200	1.68327600
H	0.13816400	3.15293200	2.29108000
S	-0.11935900	3.34192300	-0.09139100
Br	-3.34868300	0.91307100	-0.34903500

(*S*)-**TS3**$_T$-Br

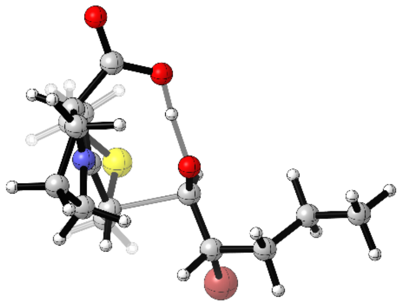

- Thermochemistry -

Zero-point correction=	0.381390 (Hartree/Particle)
Thermal correction to Energy=	0.399623
Thermal correction to Enthalpy=	0.400488
Thermal correction to Gibbs Free Energy=	0.335584
Sum of electronic and zero-point Energies=	-3835.726988
Sum of electronic and thermal Energies=	-3835.708754
Sum of electronic and thermal Enthalpies=	-3835.707889
Sum of electronic and thermal Free Energies=	-3835.772793

Number of Imaginary Frequencies = 1

E (Single Point Energy) [$IEFPCM_{(DCM)}$M06-2X/6-311++G(2d,2p)] = -3838.734863

C	0.15960300	1.24443200	0.89491700
H	-0.11313200	0.81527300	1.85575800
C	1.45265600	0.96146400	0.42908300
N	2.09999900	-0.11732400	0.87365600
C	3.33121800	-0.67037600	0.28789500
H	4.17250400	0.01107900	0.45290600
C	1.71588900	-0.83593700	2.10863900
H	0.85366000	-1.47672100	1.90419500
H	1.45570100	-0.11225500	2.88463500
C	2.96275500	-1.64718300	2.44902300
H	2.72505900	-2.53621900	3.03482300
H	3.66778600	-1.03318500	3.01762600
C	3.53970900	-1.97740300	1.07430000
H	4.59128000	-2.26498000	1.08867600
H	2.96126800	-2.78175600	0.60639200
C	2.07013900	1.69728700	-0.74082200
H	3.16064200	1.67616100	-0.64182300
H	1.82100800	1.15142200	-1.65990700
C	-0.74661200	-0.31801900	-0.26217800
H	-0.95615500	0.39130600	-1.07556000
C	-1.93210900	-0.72554600	0.62179700
O	0.08787600	-1.26050900	-0.42681800
C	3.29134400	-0.97729600	-1.21340300
O	2.14820500	-1.30104800	-1.76415200
O	4.33963000	-0.97604500	-1.83333000

H	1.28160400	-1.23676100	-1.16754400
C	-2.52322700	-2.06455500	0.17957700
H	-3.33979400	-2.32046300	0.86403100
H	-1.63016700	-0.79135800	1.66725400
C	-3.01215400	-2.11222600	-1.26627400
H	-2.19735700	-1.81409900	-1.93671600
H	-1.72893100	-2.80656500	0.32114400
H	-3.82036600	-1.38576800	-1.40397600
C	-3.49986900	-3.50785600	-1.64921400
H	-2.69410100	-4.24280800	-1.55358200
H	-3.85776300	-3.53238600	-2.68179600
H	-4.32248100	-3.82559500	-0.99982200
C	1.62291400	3.14633500	-0.89624900
C	-0.46989100	2.60387000	0.66242500
H	1.97524500	3.76045000	-0.06186200
H	2.03581100	3.55866100	-1.81827000
H	-1.54869800	2.54795400	0.79242800
H	-0.09073700	3.32837300	1.39328300
S	-0.18330100	3.27642400	-0.99709900
Br	-3.32996900	0.64907400	0.60208600

(*S*)-**TS3**$_T$-Br-P

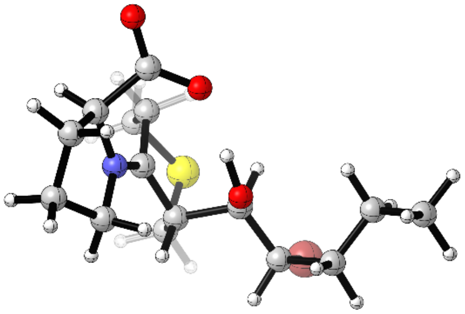

- Thermochemistry -

Zero-point correction=	0.385686 (Hartree/Particle)
Thermal correction to Energy=	0.403933
Thermal correction to Enthalpy=	0.404798
Thermal correction to Gibbs Free Energy=	0.339970
Sum of electronic and zero-point Energies=	-3835.752925
Sum of electronic and thermal Energies=	-3835.734678
Sum of electronic and thermal Enthalpies=	-3835.733813
Sum of electronic and thermal Free Energies=	-3835.798642

Number of Imaginary Frequencies = 0

E (Single Point Energy) [IEFPCM$_{(DCM)}$M06-2X/6-311++G(2d,2p)] = -3838.765953

C	0.06751100	0.82466300	0.86564900
H	0.18700900	0.49091200	1.89930200
C	1.40991400	0.84505200	0.18105900
N	2.40849200	0.19946900	0.68367100
C	3.61784800	-0.17506300	-0.09764500
H	4.17475500	0.72875800	-0.35456100
C	2.45730300	-0.41063300	2.04203200
H	1.89306300	-1.34732000	2.00950800
H	2.01075200	0.26721400	2.76778900
C	3.94832500	-0.63866000	2.25151400
H	4.13279300	-1.39133500	3.01908700
H	4.43112900	0.29552200	2.55410700

C	4.41997600	-1.06812900	0.86379600
H	5.49156200	-0.94706200	0.70704900
H	4.16273700	-2.11797200	0.68905200
C	1.52097800	1.50786300	-1.16447000
H	2.55933300	1.53780200	-1.49712700
H	0.97108100	0.88983800	-1.88394600
C	-0.72707800	-0.33160300	0.15496900
H	-0.91227400	-0.06704100	-0.89471400
C	-2.07564600	-0.61379300	0.84886700
O	0.03249500	-1.51080300	0.25676900
C	3.24498300	-0.95572100	-1.39415300
O	2.07753800	-1.42628300	-1.47510900
O	4.17922600	-1.06671000	-2.20197500
H	0.77550100	-1.50570400	-0.40053700
C	-2.58431500	-2.03690100	0.64874900
H	-3.52871700	-2.13889800	1.19545800
H	-2.00754800	-0.39806600	1.91801700
C	-2.77300800	-2.46165000	-0.80604700
H	-1.84208100	-2.28767800	-1.35711300
H	-1.85475700	-2.69865800	1.12726900
H	-3.54506900	-1.84018700	-1.27256100
C	-3.15947400	-3.93507200	-0.91614100
H	-2.37854800	-4.57527200	-0.49304400
H	-3.30696700	-4.22824100	-1.95891100
H	-4.08957700	-4.13757300	-0.37433100
C	0.95139100	2.93271000	-1.16992200
C	-0.58165000	2.21463400	0.90518400
H	1.51391700	3.58201700	-0.49205800

H	1.03915800	3.34165400	-2.17765200
H	-1.55901400	2.16126100	1.38371300
H	0.04319600	2.87982700	1.51004200
S	-0.80718000	2.96750300	-0.73009300
Br	-3.45835500	0.64211600	0.21010900

Summary of Transition State Energies

Supplementary Table 13. Energies of all transition states for enamine addition to 2-chloropentanal. Reported relative Gibbs free energies for structures optimized at the IEFPCM$_{(DCM)}$M06-2X/6-311++G(2d,2p)//IEFPCM$_{(DCM)}$M06-2X/6-31+G(d,p) level of theory represent the sum of the thermal correction to Gibbs free energy computed at the IEFPCM$_{(DCM)}$M06-2X/6-31+G(d,p) level of theory and single point energies computed at the IEFPCM$_{(DCM)}$M06-2X/6-311++G(2d,2p). All energies are reported in Hartree except the relative energies.

Structure	Gibbs Free Energies (G), IEFPCM$_{(DCM)}$M06-2X/6-31+G(d,p)	Thermal Corrections to Gibbs Free Energies, IEFPCM$_{(DCM)}$M06-2X/6-31+G(d,p)	Single Point Energies, E IEFPCM$_{(DCM)}$M06-2X/6-311++G(2d,2p)	Gibbs Free Energies (G), IEFPCM$_{(DCM)}$M06-2X/6-311++G(2d,2p)// IEFPCM$_{(DCM)}$M06-2X/6-31+G(d,p)	Relative Energies to (*R*)-**TS1**$_{P}$-Cl (Kcal/mol)
(*R*)-**TS1**$_{P}$-Cl	-1401.164861	0.340985	-1401.797805	-1401.45682	0
(*S*)-**TS1**$_{P}$-Cl	-1401.159001	0.341063	-1401.791734	-1401.450671	3.85855776
(*R*)-**TS2**$_{P}$-Cl	-1401.156596	0.341689	-1401.790092	-1401.448403	5.281749987
(*S*)-**TS2**$_{P}$-Cl	-1401.164151	0.340244	-1401.796294	-1401.45605	0.483182546
(*R*)-**TS3**$_{P}$-Cl	-1401.159118	0.341323	-1401.792111	0.341323	3.785139114
(*S*)-**TS3**$_{P}$-Cl	-1401.159908	0.340366	-1401.791658	-1401.451292	3.468874174

Supplementary Table 14. Energies of all transition states for enamine addition to 2-chloropentanal. Reported relative Gibbs free energies for structures optimized at the IEFPCM$_{(DCM)}$M06-2X/6-311++G(2d,2p)//IEFPCM$_{(DCM)}$M06-2X/6-31+G(d,p) level of theory represent the sum of the thermal correction to Gibbs free energy computed at the IEFPCM$_{(DCM)}$M06-2X/6-31+G(d,p) level of theory and single point energies computed at the IEFPCM$_{(DCM)}$M06-2X/6-311++G(2d,2p). All energies are reported in Hartree except the relative energies.

Structure	Gibbs Free Energies (G), IEFPCM$_{(DCM)}$M06-2X/6-31+G(d,p)	Thermal Corrections to Gibbs Free Energies, IEFPCM$_{(DCM)}$M06-2X/6-31+G(d,p)	Single Point Energies, E IEFPCM$_{(DCM)}$M06-2X/6-311++G(2d,2p)	Gibbs Free Energies (G), IEFPCM$_{(DCM)}$M06-2X/6-311++G(2d,2p)// IEFPCM$_{(DCM)}$M06-2X/6-31+G(d,p)	Relative Energies to (*R*)-**TS1**$_{G}$-Cl (Kcal/mol)
(*R*)-**TS1**$_{G}$-Cl	-1365.252009	0.36506	-1365.894619	-1365.529559	0
(*S*)-**TS1**$_{G}$-Cl	-1365.245974	0.364994	-1365.888191	-1365.523197	3.992217348

(*R*)-**TS2**$_{G}$-Cl	-1365.244059	0.365714	-1365.887212	-1365.521498	1.06613915
(*S*)-**TS2**$_{G}$-Cl	-1365.251485	0.364656	-1365.893626	-1365.52897	0.369603272
(*R*)-**TS3**$_{G}$-Cl	-1365.246524	0.364421	-1365.888196	-1365.523775	3.629516683
(*S*)-**TS3**$_{G}$-Cl	-1365.247432	0.364491	-1365.88909	-1365.524599	3.112448608

Supplementary Table 15. Energies of all transition states for enamine addition to 2-chloropentanal. Reported relative Gibbs free energies for structures optimized at the IEFPCM$_{(DCM)}$M06-2X/6-311++G(2d,2p)//IEFPCM$_{(DCM)}$M06-2X/6-31+G(d,p) level of theory represent the sum of the thermal correction to Gibbs free energy computed at the IEFPCM$_{(DCM)}$M06-2X/6-31+G(d,p) level of theory and single point energies computed at the IEFPCM$_{(DCM)}$M06-2X/6-311++G(2d,2p). All energies are reported in Hartree except the relative energies.

**Structure**	**Gibbs Free Energies (G), IEFPCM$_{(DCM)}$M06-2X/6-31+G(d,p)**	**Thermal Corrections to Gibbs Free Energies, IEFPCM$_{(DCM)}$M06-2X/6-31+G(d,p)**	**Single Point Energies, E IEFPCM$_{(DCM)}$M06-2X/6-311++G(2d,2p)**	**Gibbs Free Energies (G), IEFPCM$_{(DCM)}$M06-2X/6-311++G(2d,2p)// IEFPCM$_{(DCM)}$M06-2X/6-31+G(d,p)**	**Relative Energies to** (*R*)-**TS1**$_{O}$-Cl **(Kcal/mol)**
(*R*)-**TS1**$_{O}$-Cl	-1515.632299	0.369496	-1516.327244	-1515.957748	0
(*S*)-**TS1**$_{O}$-Cl	-1515.628417	0.369358	-1516.322975	-1515.953617	2.592242984
(*R*)-**TS2**$_{O}$-Cl	-1515.62514	0.369078	-1516.319381	-1515.950303	4.671810461
(*S*)-**TS2**$_{O}$-Cl	-1515.630967	0.369297	-1516.32557	-1515.956273	0.925576955
(*R*)-**TS3**$_{O}$-Cl	-1515.625316	0.36887	-1516.319628	-1515.950758	4.386293502
(*S*)-**TS3**$_{O}$-Cl	-1515.624654	0.369175	-1516.318913	-1515.949738	5.026353498

Supplementary Table 16. Energies of all transition states for enamine addition to 2-chloropentanal. Reported relative Gibbs free energies for structures optimized at the IEFPCM$_{(DCM)}$M06-2X/6-311++G(2d,2p)//IEFPCM$_{(DCM)}$M06-2X/6-31+G(d,p) level of theory represent the sum of the thermal correction to Gibbs free energy computed at the IEFPCM$_{(DCM)}$M06-2X/6-31+G(d,p) level of theory and single point energies computed at the IEFPCM$_{(DCM)}$M06-2X/6-311++G(2d,2p). All energies are reported in Hartree except the relative energies.

**Structure**	**Gibbs Free Energies (G), IEFPCM$_{(DCM)}$M06-2X/6-31+G(d,p)**	**Thermal Corrections to Gibbs Free Energies, IEFPCM$_{(DCM)}$M06-2X/6-31+G(d,p)**	**Single Point Energies, E IEFPCM$_{(DCM)}$M06-2X/6-311++G(2d,2p)**	**Gibbs Free Energies (G), IEFPCM$_{(DCM)}$M06-2X/6-311++G(2d,2p)// IEFPCM$_{(DCM)}$M06-2X/6-31+G(d,p)**	**Relative Energies to (*R*)-TS1$_T$-Cl (Kcal/mol)**
(*R*)-**TS1**$_T$-Cl	-1724.137252	0.336803	-1724.774436	-1724.437633	0
(*S*)-**TS1**$_T$-Cl	-1724.131053	0.337056	-1724.768234	-1724.431178	4.050575759
(*R*)-**TS2**$_T$-Cl	-1724.128796	0.337245	-1724.766523	-1724.429278	5.242844379
(*S*)-**TS2**$_T$-Cl	-1724.136217	0.336784	-1724.773577	-1724.436793	0.527108232
(*R*)-**TS3**$_T$-Cl	-1724.132824	0.336882	-1724.769951	-1724.433069	2.863954727
(*S*)-**TS3**$_T$-Cl	-1724.132677	0.336822	-1724.769924	-1724.433102	2.843246904

Supplementary Table 17. Energies of all transition states for enamine addition to 2-fluoropentanal. Reported relative Gibbs free energies for structures optimized at the IEFPCM$_{(DCM)}$M06-2X/6-311++G(2d,2p)//IEFPCM$_{(DCM)}$M06-2X/6-31+G(d,p) level of theory represent the sum of the thermal correction to Gibbs free energy computed at the IEFPCM$_{(DCM)}$M06-2X/6-31+G(d,p) level of theory and single point energies computed at the IEFPCM$_{(DCM)}$M06-2X/6-311++G(2d,2p). All energies are reported in Hartree except the relative energies.

**Structure**	**Gibbs Free Energies (G), IEFPCM$_{(DCM)}$M06-2X/6-31+G(d,p)**	**Thermal Corrections to Gibbs Free Energies, IEFPCM$_{(DCM)}$M06-2X/6-31+G(d,p)**	**Single Point Energies, E IEFPCM$_{(DCM)}$M06-2X/6-311++G(2d,2p)**	**Gibbs Free Energies (G), IEFPCM$_{(DCM)}$M06-2X/6-311++G(2d,2p)// IEFPCM$_{(DCM)}$M06-2X/6-31+G(d,p)**	**Relative Energies to (*R*)-TS1$_P$-F (Kcal/mol)**
(*R*)-**TS1**$_P$-F	-1040.805151	0.342953	-1041.438205	-1041.095252	0
(*S*)-**TS1**$_P$-F	-1040.79923	0.343526	-1041.432614	-1041.089088	3.867970407
(*R*)-**TS2**$_P$-F	-1040.796259	0.343633	-1041.429464	-1041.085831	5.911769826
(*S*)-**TS2**$_P$-F	-1040.801762	0.34289	-1041.434367	-1041.091477	2.368849495

(*R*)-**TS3**$_{P}$-F	-1040.80187	0.343573	-1041.435017	-1041.091444	2.389557318
(*S*)-**TS3**$_{P}$-F	-1040.804044	0.342145	-1041.43582	-1041.093675	0.989582955

Supplementary Table 18. Energies of all transition states for enamine addition to 2-fluoropentanal. Reported relative Gibbs free energies for structures optimized at the IEFPCM$_{(DCM)}$M06-2X/6-311++G(2d,2p)//IEFPCM$_{(DCM)}$M06-2X/6-31+G(d,p) level of theory represent the sum of the thermal correction to Gibbs free energy computed at the IEFPCM$_{(DCM)}$M06-2X/6-31+G(d,p) level of theory and single point energies computed at the IEFPCM$_{(DCM)}$M06-2X/6-311++G(2d,2p). All energies are reported in Hartree except the relative energies.

**Structure**	**Gibbs Free Energies (G), IEFPCM$_{(DCM)}$M06-2X/6-31+G(d,p)**	**Thermal Corrections to Gibbs Free Energies, IEFPCM$_{(DCM)}$M06-2X/6-31+G(d,p)**	**Single Point Energies, E IEFPCM$_{(DCM)}$M06-2X/6-311++G(2d,2p)**	**Gibbs Free Energies (G), IEFPCM$_{(DCM)}$M06-2X/6-311++G(2d,2p)// IEFPCM$_{(DCM)}$M06-2X/6-31+G(d,p)**	**Relative Energies to** (*R*)-**TS1**$_{G}$-F **(Kcal/mol)**
(*R*)-**TS1**$_{G}$-F	-1004.892614	0.366742	-1005.535067	-1005.168325	0
(*S*)-**TS1**$_{G}$-F	-1004.886483	0.36724	-1005.52915	-1005.16191	4.025475367
(*R*)-**TS2**$_{G}$-F	-1004.883971	0.367328	-1005.526459	-1005.159131	5.769325101
(*S*)-**TS2**$_{G}$-F	-1004.889686	0.366632	-1005.5316	-1005.164968	2.106550399
(*R*)-**TS3**$_{G}$-F	-1004.888828	0.367347	-1005.531517	-1005.16417	2.607303219
(*S*)-**TS3**$_{G}$-F	-1004.890519	0.366469	-1005.532406	-1005.165937	1.498493402

Supplementary Table 19. Energies of all transition states for enamine addition to 2-fluoropentanal. Reported relative Gibbs free energies for structures optimized at the IEFPCM$_{(DCM)}$M06-2X/6-311++G(2d,2p)//IEFPCM$_{(DCM)}$M06-2X/6-31+G(d,p) level of theory represent the sum of the thermal correction to Gibbs free energy computed at the IEFPCM$_{(DCM)}$M06-2X/6-31+G(d,p) level of theory and single point energies computed at the IEFPCM$_{(DCM)}$M06-2X/6-311++G(2d,2p). All energies are reported in Hartree except the relative energies.

**Structure**	**Gibbs Free Energies (G), IEFPCM$_{(DCM)}$M06-2X/6-31+G(d,p)**	**Thermal Corrections to Gibbs Free Energies, IEFPCM$_{(DCM)}$M06-2X/6-31+G(d,p)**	**Single Point Energies, E IEFPCM$_{(DCM)}$M06-2X/6-311++G(2d,2p)**	**Gibbs Free Energies (G), IEFPCM$_{(DCM)}$M06-2X/6-311++G(2d,2p)// IEFPCM$_{(DCM)}$M06-2X/6-31+G(d,p)**	**Relative Energies to** (*R*)-**TS1**$_{O}$-F **(Kcal/mol)**
(*R*)-**TS1**$_{O}$-F	-1155.272322	0.371031	-1155.966865	-1155.595834	0

(*S*)-**TS1**$_O$-F	-1155.267711	0.371627	-1155.962553	-1155.590926	3.079818098
(*R*)-**TS2**$_O$-F	-1155.264945	0.371067	-1155.958868	-1155.587801	5.040786224
(*S*)-**TS2**$_O$-F	-1155.268666	0.371738	-1155.963577	-1155.591839	2.506901651
(*R*)-**TS3**$_O$-F	-1155.266603	0.370379	-1155.960428	-1155.590049	3.630144193
(*S*)-**TS3**$_O$-F	-1155.266948	0.370957	-1155.961139	-1155.590182	3.54668539

Supplementary Table 20. Energies of all transition states for enamine addition to 2-fluoropentanal. Reported relative Gibbs free energies for structures optimized at the IEFPCM$_{(DCM)}$M06-2X/6-311++G(2d,2p)//IEFPCM$_{(DCM)}$M06-2X/6-31+G(d,p) level of theory represent the sum of the thermal correction to Gibbs free energy computed at the IEFPCM$_{(DCM)}$M06-2X/6-31+G(d,p) level of theory and single point energies computed at the IEFPCM$_{(DCM)}$M06-2X/6-311++G(2d,2p). All energies are reported in Hartree except the relative energies.

**Structure**	**Gibbs Free Energies (G), IEFPCM$_{(DCM)}$M06-2X/6-31+G(d,p)**	**Thermal Corrections to Gibbs Free Energies, IEFPCM$_{(DCM)}$M06-2X/6-31+G(d,p)**	**Single Point Energies, E IEFPCM$_{(DCM)}$M06-2X/6-311++G(2d,2p)**	**Gibbs Free Energies (G), IEFPCM$_{(DCM)}$M06-2X/6-311++G(2d,2p)// IEFPCM$_{(DCM)}$M06-2X/6-31+G(d,p)**	**Relative Energies to** (*R*)-**TS1**$_T$-F **(Kcal/mol)**
(*R*)-**TS1**$_T$-F	-1363.777503	0.338653	-1364.414632	-1364.075979	0
(*S*)-**TS1**$_T$-F	-1363.771275	0.339218	-1364.408701	-1364.069483	4.076303661
(*R*)-**TS2**$_T$-F	-1363.768554	0.339298	-1364.406058	-1364.06676	5.785012846
(*S*)-**TS2**$_T$-F	-1363.774512	0.338699	-1364.411596	-1364.072897	1.933985204
(*R*)-**TS3**$_T$-F	-1363.775467	0.338235	-1364.411768	-1364.073533	1.534888971
(*S*)-**TS3**$_T$-F	-1363.776417	0.33776	-1364.412197	-1364.074437	0.967620111

Supplementary Table 21. Energies of all transition states for enamine addition to 2-bromopentanal. Reported relative Gibbs free energies for structures optimized at the $IEFPCM_{(DCM)}$M06-2X/6-311++G(2d,2p)//$IEFPCM_{(DCM)}$M06-2X/6-31+G(d,p) level of theory represent the sum of the thermal correction to Gibbs free energy computed at the $IEFPCM_{(DCM)}$M06-2X/6-31+G(d,p) level of theory and single point energies computed at the $IEFPCM_{(DCM)}$M06-2X/6-311++G(2d,2p). All energies are reported in Hartree except the relative energies.

**Structure**	**Gibbs Free Energies (G), $IEFPCM_{(DCM)}$M06-2X/6-31+G(d,p)**	**Thermal Corrections to Gibbs Free Energies, $IEFPCM_{(DCM)}$M06-2X/6-31+G(d,p)**	**Single Point Energies, E $IEFPCM_{(DCM)}$M06-2X/6-311++G(2d,2p)**	**Gibbs Free Energies (G), $IEFPCM_{(DCM)}$M06-2X/6-311++G(2d,2p)// $IEFPCM_{(DCM)}$M06-2X/6-31+G(d,p)**	**Relative Energies to** (*R*)-**TS1**$_P$-Br **(Kcal/mol)**
(*R*)-**TS1**$_P$-Br	-3512.807029	0.339202	-3515.764676	-3515.425474	0
(*S*)-**TS1**$_P$-Br	-3512.797441	0.339792	-3515.755215	-3515.415423	6.307101
(*R*)-**TS2**$_P$-Br	-3512.79657	0.340676	-3515.7581	-3515.417424	5.05145389
(*S*)-**TS2**$_P$-Br	-3512.804072	0.339866	-3515.764418	-3515.424552	0.578564035
(*R*)-**TS3**$_P$-Br	-3512.79908	0.339631	-3515.75725	-3515.417619	4.929089479
(*S*)-**TS3**$_P$-Br	-3512.798467	0.339054	-3515.757188	-3515.418134	4.605921932

Supplementary Table 22. Energies of all transition states for enamine addition to 2-bromopentanal. Reported relative Gibbs free energies for structures optimized at the $IEFPCM_{(DCM)}$M06-2X/6-311++G(2d,2p)//$IEFPCM_{(DCM)}$M06-2X/6-31+G(d,p) level of theory represent the sum of the thermal correction to Gibbs free energy computed at the $IEFPCM_{(DCM)}$M06-2X/6-31+G(d,p) level of theory and single point electronic energies computed at the $IEFPCM_{(DCM)}$M06-2X/6-311++G(2d,2p). All energies are reported in Hartree except the relative energies.

**Structure**	**Gibbs Free Energies (G), $IEFPCM_{(DCM)}$M06-2X/6-31+G(d,p)**	**Thermal Corrections to Gibbs Free Energies, $IEFPCM_{(DCM)}$M06-2X/6-31+G(d,p)**	**Single Point Energies, E $IEFPCM_{(DCM)}$M06-2X/6-311++G(2d,2p)**	**Gibbs Free Energies (G), $IEFPCM_{(DCM)}$M06-2X/6-311++G(2d,2p)// $IEFPCM_{(DCM)}$M06-2X/6-31+G(d,p)**	**Relative Energies to** (*R*)-**TS1**$_G$-Br **(Kcal/mol)**
(*R*)-**TS1**$_G$-Br	-3476.894285	0.363361	-3479.861538	-3479.498177	0
(*S*)-**TS1**$_G$-Br	-3476.887892	0.363834	-3479.855148	-3479.491314	4.306599757
(*R*)-**TS2**$_G$-Br	-3476.884545	0.364163	-3479.855265	-3479.491102	4.439631835
(*S*)-**TS2**$_G$-Br	-3476.892016	0.363804	-3479.861757	-3479.497953	0.140562195

(*R*)-**TS3**$_G$-Br	-3476.886609	0.364378	-3479.854643	-3479.490265	4.964857538
(*S*)-**TS3**$_G$-Br	-3476.886337	0.363619	-3479.854911	-3479.491292	4.320404973

Supplementary Table 23. Energies of all transition states for enamine addition to 2-bromopentanal. Reported relative Gibbs free energies for structures optimized at the IEFPCM$_{(DCM)}$M06-2X/6-311++G(2d,2p)//IEFPCM$_{(DCM)}$M06-2X/6-31+G(d,p) level of theory represent the sum of the thermal correction to Gibbs free energy computed at the IEFPCM$_{(DCM)}$M06-2X/6-31+G(d,p) level of theory and single point energies computed at the IEFPCM$_{(DCM)}$M06-2X/6-311++G(2d,2p). All energies are reported in Hartree except the relative energies.

**Structure**	**Gibbs Free Energies (G), IEFPCM$_{(DCM)}$M06-2X/6-31+G(d,p)**	**Thermal Corrections to Gibbs Free Energies, IEFPCM$_{(DCM)}$M06-2X/6-31+G(d,p)**	**Single Point Energies, E IEFPCM$_{(DCM)}$M06-2X/6-311++G(2d,2p)**	**Gibbs Free Energies (G), IEFPCM$_{(DCM)}$M06-2X/6-311++G(2d,2p)// IEFPCM$_{(DCM)}$M06-2X/6-31+G(d,p)**	**Relative Energies to** (*R*)-**TS1**$_O$-Br **(Kcal/mol)**
(*R*)-**TS1**$_O$-Br	-3627.274362	0.367844	-3630.294358	-3629.926514	0
(*S*)-**TS1**$_O$-Br	-3627.270827	0.367855	-3630.290158	-3629.922303	2.642443768
(*R*)-**TS2**$_O$-Br	-3627.266059	0.369445	-3630.289223	-3629.919778	4.226906013
(*S*)-**TS2**$_O$-Br	-3627.272308	0.368103	-3630.293793	-3629.92569	0.517068075
(*R*)-**TS3**$_O$-Br	-3627.26738	0.36793	-3630.286244	-3629.918314	5.14558036
(*S*)-**TS3**$_O$-Br	-3627.265946	0.368051	-3630.285366	-3629.917315	5.77246265

Supplementary Table 24. Energies of all transition states for enamine addition to 2-bromopentanal. Reported relative Gibbs free energies for structures optimized at the IEFPCM$_{(DCM)}$M06-2X/6-311++G(2d,2p)//IEFPCM$_{(DCM)}$M06-2X/6-31+G(d,p) level of theory represent the sum of the thermal correction to Gibbs free energy computed at the IEFPCM$_{(DCM)}$M06-2X/6-31+G(d,p) level of theory and single point energies computed at the IEFPCM$_{(DCM)}$M06-2X/6-311++G(2d,2p). All energies are reported in Hartree except the relative energies.

**Structure**	**Gibbs Free Energies (G), IEFPCM$_{(DCM)}$M06-2X/6-31+G(d,p)**	**Thermal Corrections to Gibbs Free Energies, IEFPCM$_{(DCM)}$M06-2X/6-31+G(d,p)**	**Single Point Energies, E IEFPCM$_{(DCM)}$M06-2X/6-311++G(2d,2p)**	**Gibbs Free Energies (G), IEFPCM$_{(DCM)}$M06-2X/6-311++G(2d,2p)// IEFPCM$_{(DCM)}$M06-2X/6-31+G(d,p)**	**Relative Energies to** (*R*)-**TS1**$_T$-Br **(Kcal/mol)**
(*R*)-**TS1**$_T$-Br	-3835.779609	0.335333	-3838.741514	-3838.406181	0

(*S*)-**TS1**$_{T}$-Br	-3835.77367	0.335793	-3838.735267	-3838.399474	4.208708229
(*R*)-**TS2**$_{T}$-Br	-3835.769038	0.335789	-3838.734498	-3838.398709	4.688753226
(*S*)-**TS2**$_{T}$-Br	-3835.777067	0.335558	-3838.741723	-3838.406165	0.010040157
(*R*)-**TS3**$_{T}$-Br	-3835.773702	0.33612	-3838.736601	-3838.400481	3.57680586
(*S*)-**TS3**$_{T}$-Br	-3835.772793	0.335584	-3838.734863	-3838.399279	4.331072639

Noncovalent Interaction (NCI) Surfaces

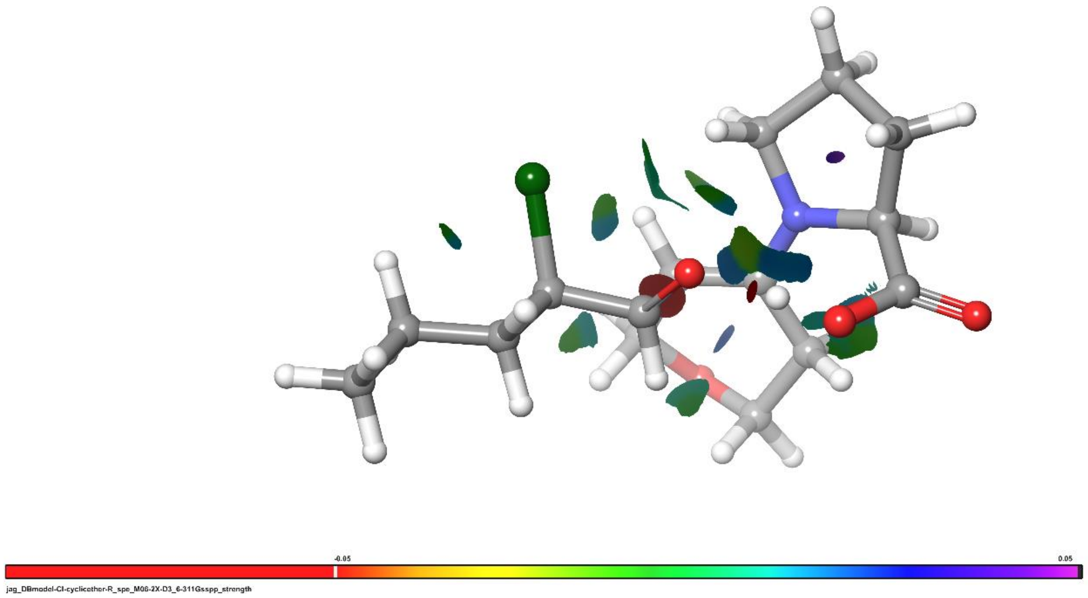

Supplementary Figure 87. Noncovalent Interaction (NCI) Surfaces of transition states (*R*)-**TS1**$_{P}$-Cl (isovalue = 0.3, min = -0.5 and max = 0.5) computed at the M06-2X-D3/LACV3P++** level of theory.

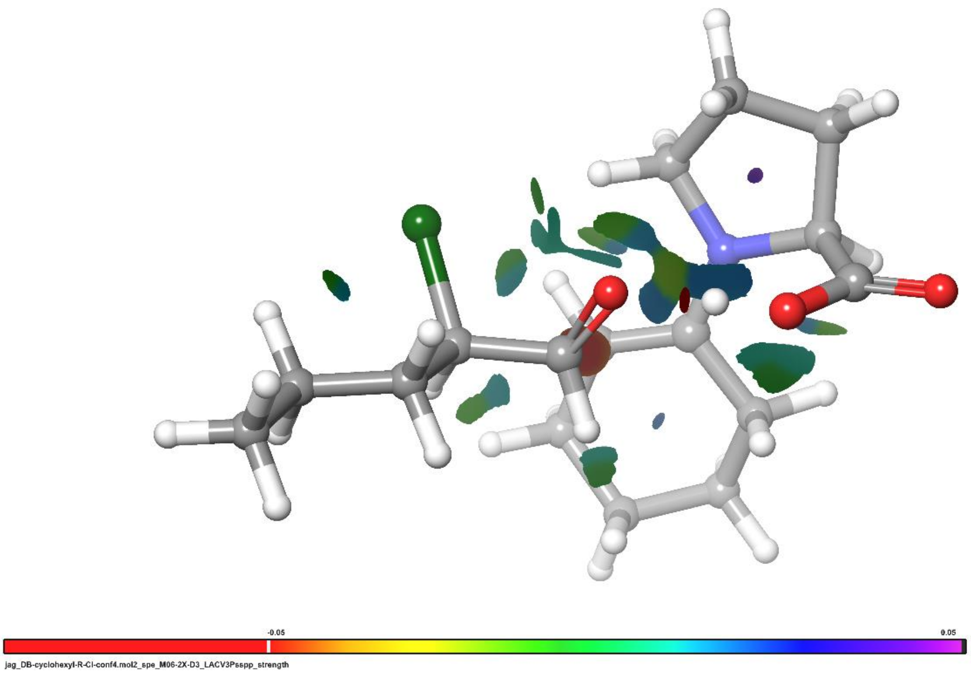

Supplementary Figure 88. Noncovalent Interaction (NCI) Surfaces of transition states (*R*)-**TS1**$_{G}$-Cl (isovalue = 0.3, min = -0.5 and max = 0.5) computed at the M06-2X-D3/LACV3P++** level of theory.

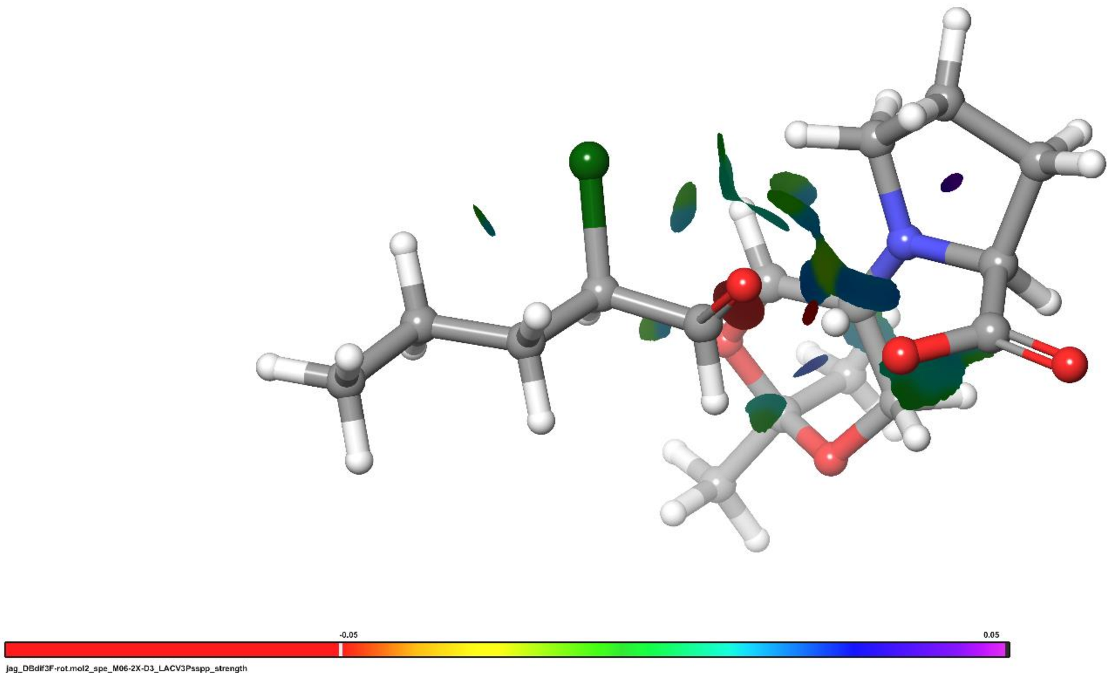

Supplementary Figure 89. Noncovalent Interaction (NCI) Surfaces of transition states (*R*)-**TS1**$_{O}$-Cl (isovalue = 0.3, min = -0.5 and max = 0.5) computed at the M06-2X-D3/LACV3P++** level of theory.

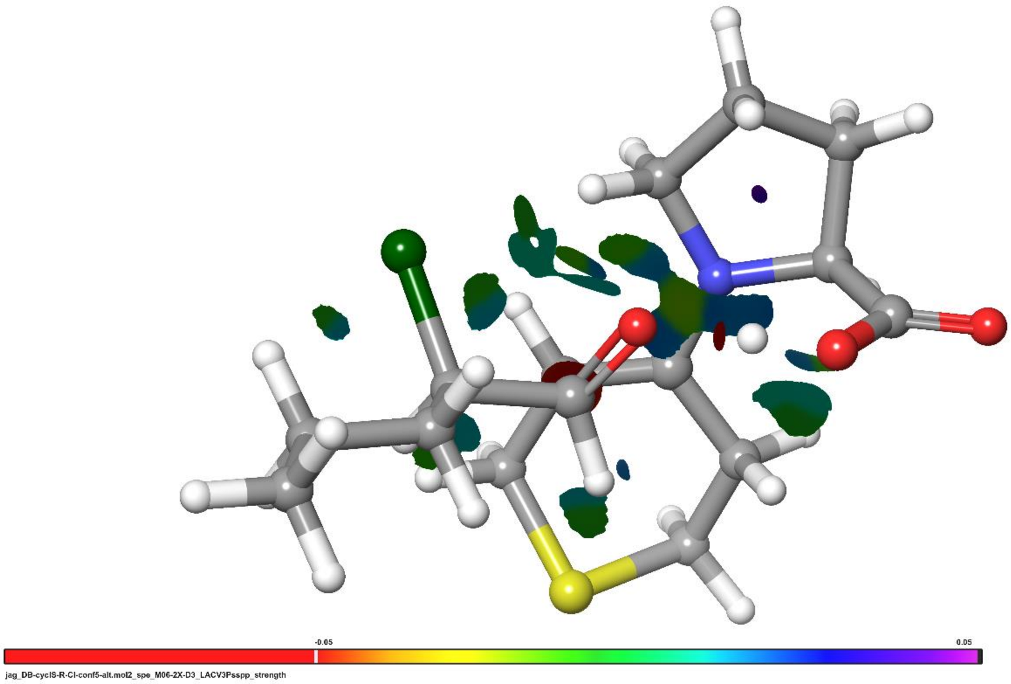

Supplementary Figure 90. Noncovalent Interaction (NCI) Surfaces of transition states (*R*)-**TS1**$_{T}$-Cl (isovalue = 0.3, min = -0.5 and max = 0.5) computed at the M06-2X-D3/LACV3P++** level of theory.

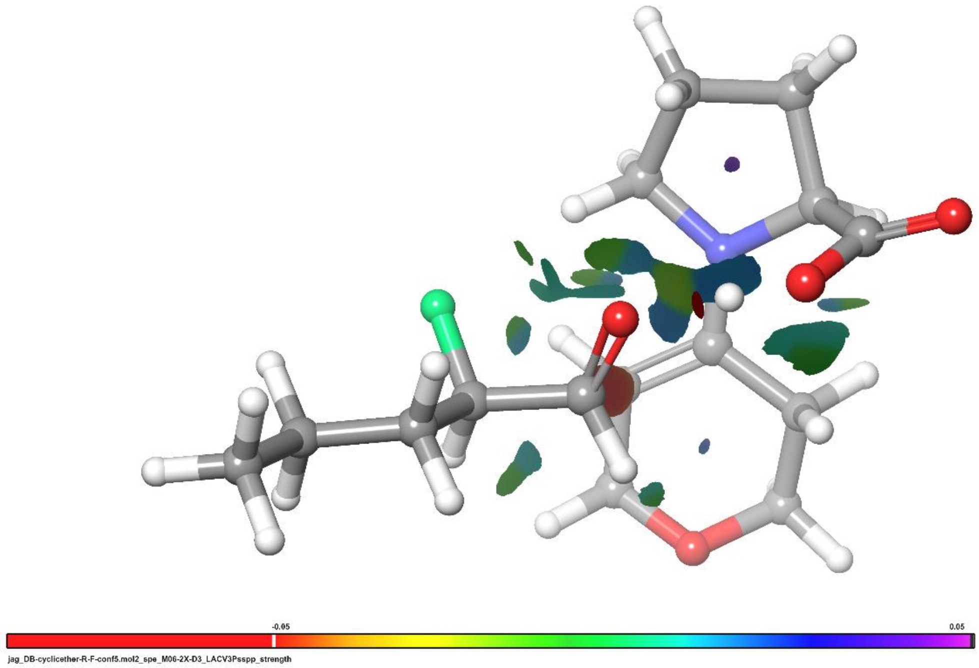

Supplementary Figure 91. Noncovalent Interaction (NCI) Surfaces of transition states (*R*)-**TS1**$_{P}$**-F** (isovalue = 0.3, min = -0.5 and max = 0.5) computed at the M06-2X-D3/LACV3P++** level of theory.

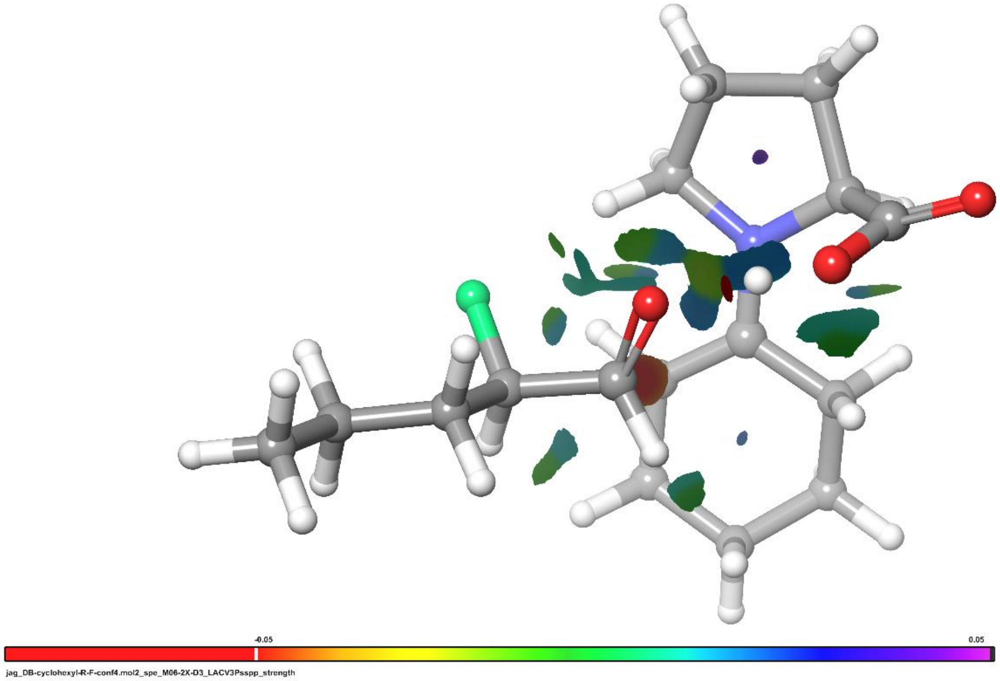

Supplementary Figure 92. Noncovalent Interaction (NCI) Surfaces of transition states (*R*)-**TS1**$_{G}$**-F** (isovalue = 0.3, min = -0.5 and max = 0.5) computed at the M06-2X-D3/LACV3P++** level of theory.

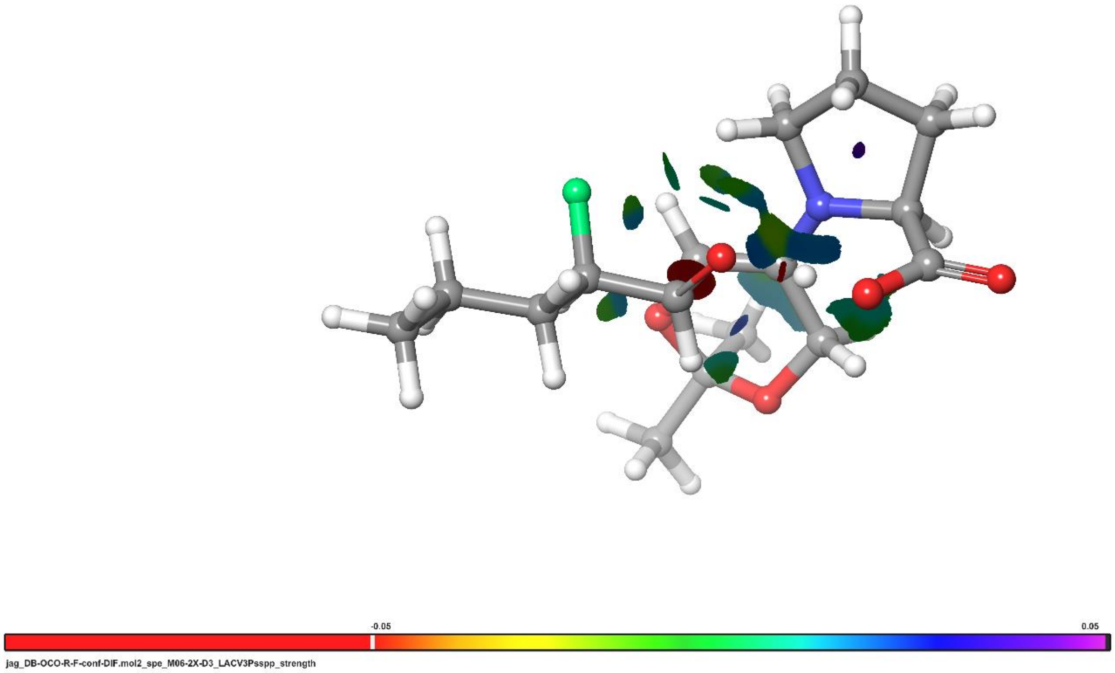

Supplementary Figure 93. Noncovalent Interaction (NCI) Surfaces of transition states (*R*)-**TS1**$_{O}$-F (isovalue = 0.3, min = -0.5 and max = 0.5) computed at the M06-2X-D3/LACV3P++** level of theory.

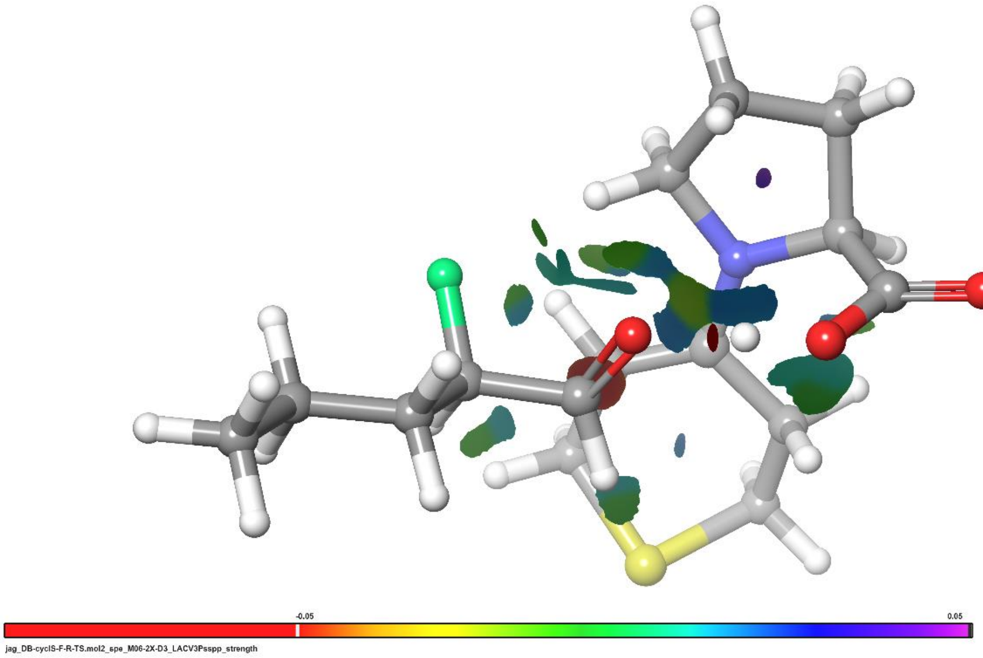

Supplementary Figure 94. Noncovalent Interaction (NCI) Surfaces of transition states (*R*)-**TS1**$_{T}$-F (isovalue = 0.3, min = -0.5 and max = 0.5) computed at the M06-2X-D3/LACV3P++** level of theory.

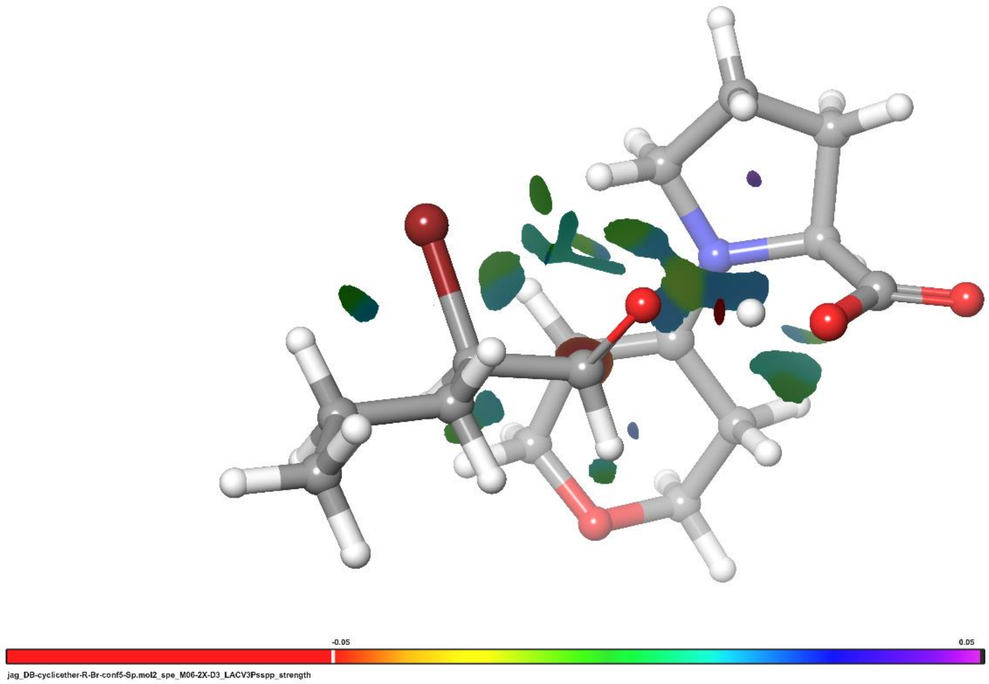

Supplementary Figure 95. Noncovalent Interaction (NCI) Surfaces of transition states (*R*)-**TS1**$_{P}$-Br (isovalue = 0.3, min = -0.5 and max = 0.5) computed at the M06-2X-D3/LACV3P++** level of theory.

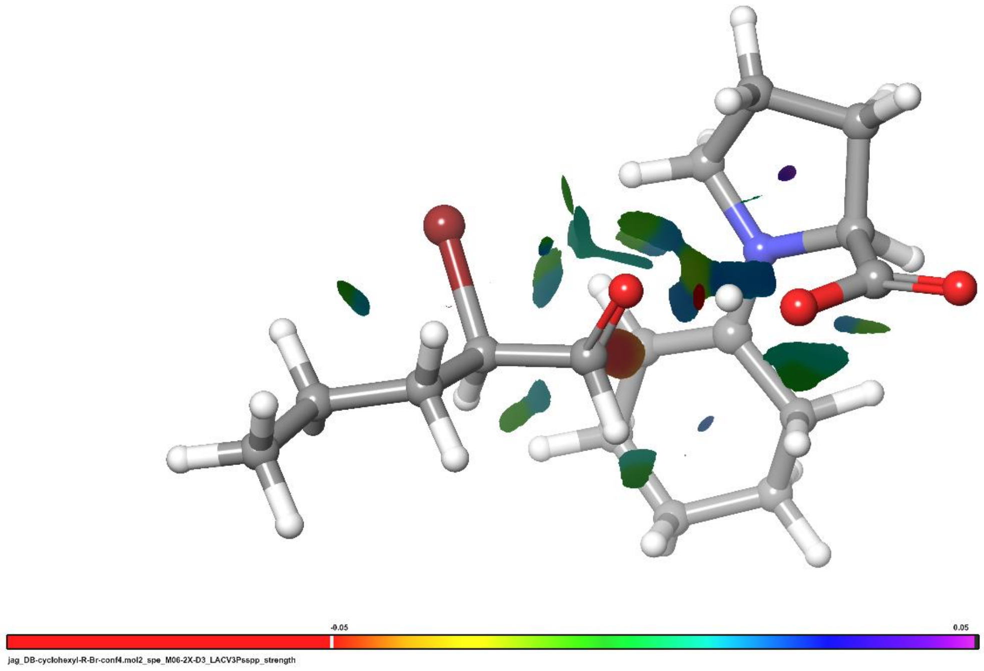

Supplementary Figure 96. Noncovalent Interaction (NCI) Surfaces of transition states (*R*)-**TS1**$_{G}$-Br (isovalue = 0.3, min = -0.5 and max = 0.5) computed at the M06-2X-D3/LACV3P++** level of theory.

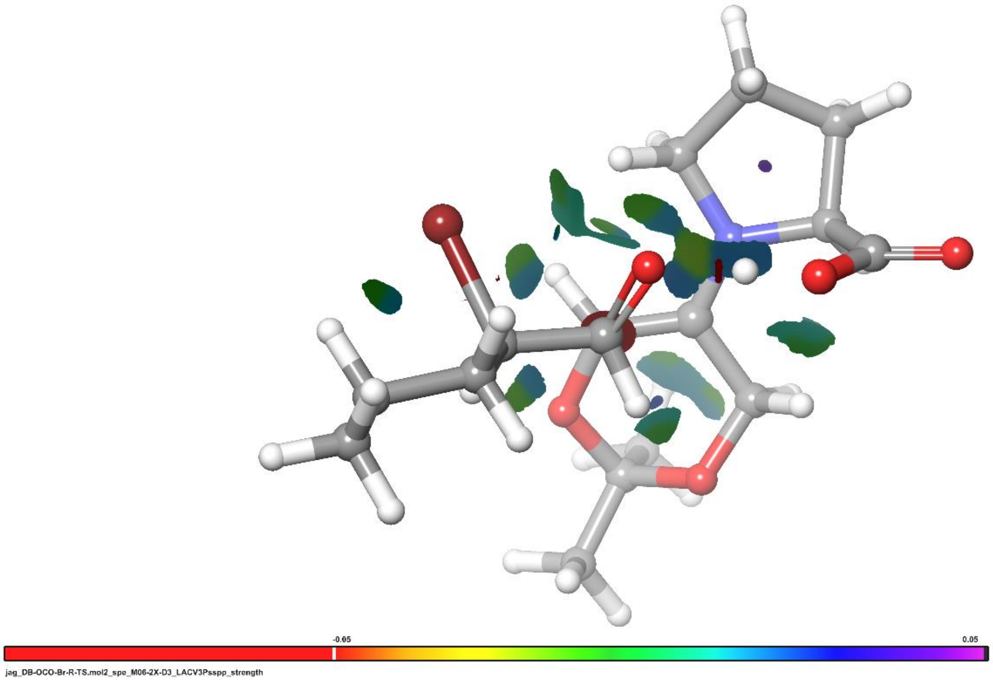

Supplementary Figure 97. Noncovalent Interaction (NCI) Surfaces of transition states (*R*)-**TS1**$_{O}$-Br (isovalue = 0.3, min = -0.5 and max = 0.5) computed at the M06-2X-D3/LACV3P++** level of theory.

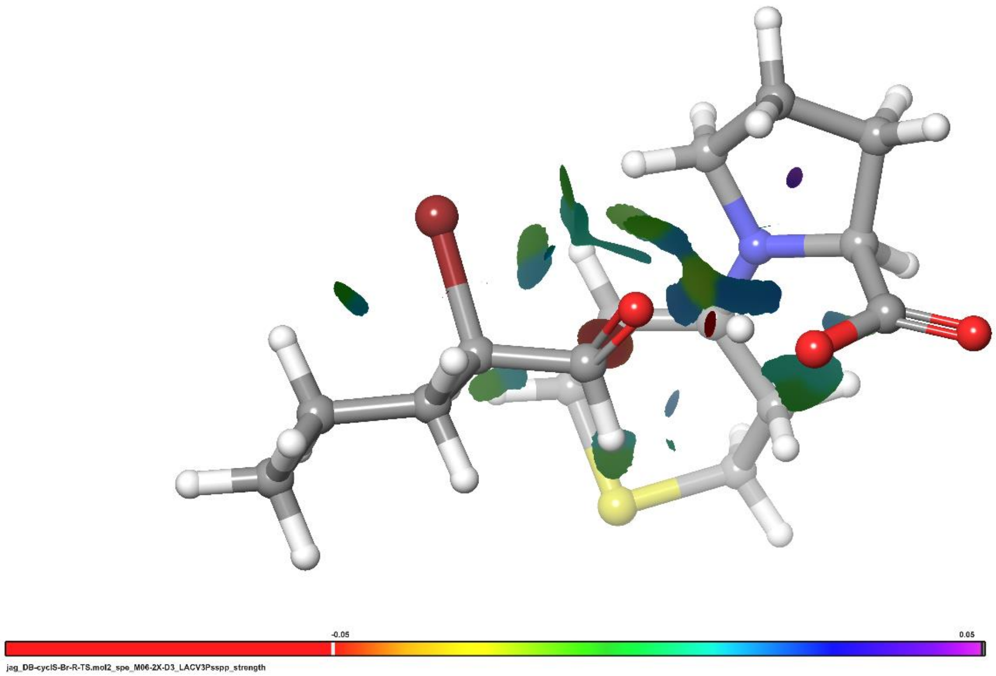

Supplementary Figure 98. Noncovalent Interaction (NCI) Surfaces of transition states (R)-**TS1**$_T$-Br (isovalue = 0.3, min = -0.5 and max = 0.5) computed at the M06-2X-D3/LACV3P++** level of theory.

Figures of Fixed Internal Coordinates used for Conformational Searches

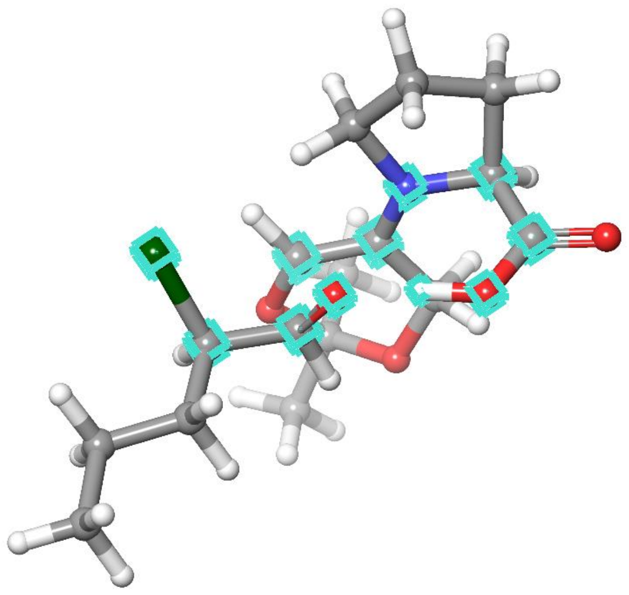

Supplementary Figure 99. Fixed internal coordinate of Dudding-Britton-type transition state model for addition of enamine derived cyclohexanone to (*R*)-2-chloropentanal.

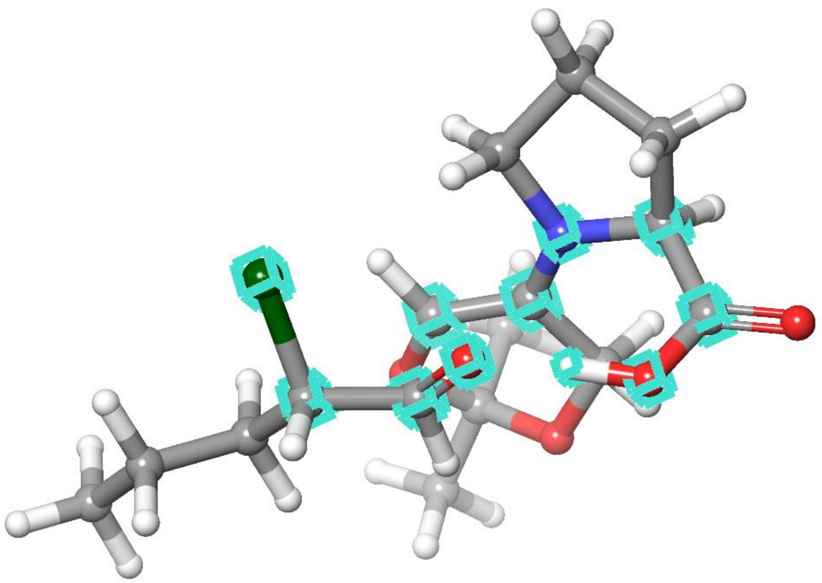

Supplementary Figure 100. Fixed internal coordinate of Dudding-Britton-type transition state model for addition of enamine derived cyclohexanone to (*S*)-2-chloropentanal.

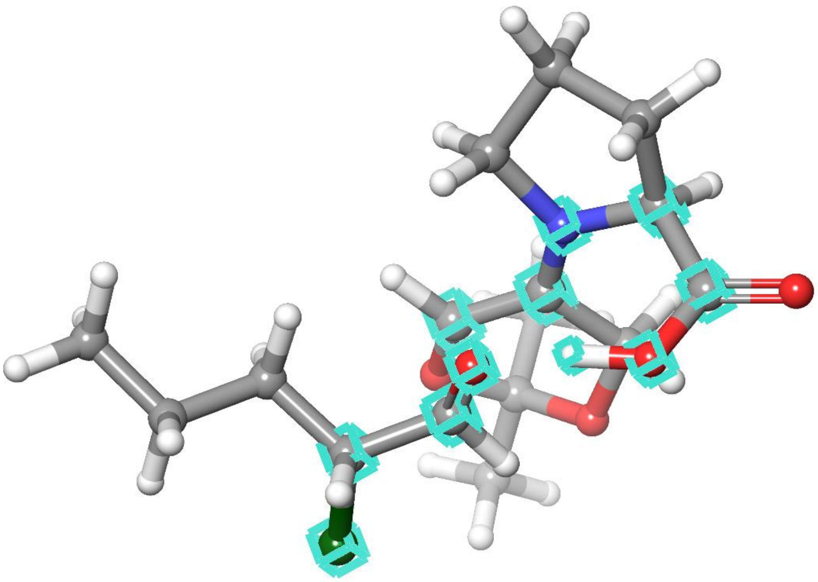

Supplementary Figure 101. Fixed internal coordinate of Evans-Cornforth-type transition state model for addition of enamine derived cyclohexanone to (*R*)-2-chloropentanal.

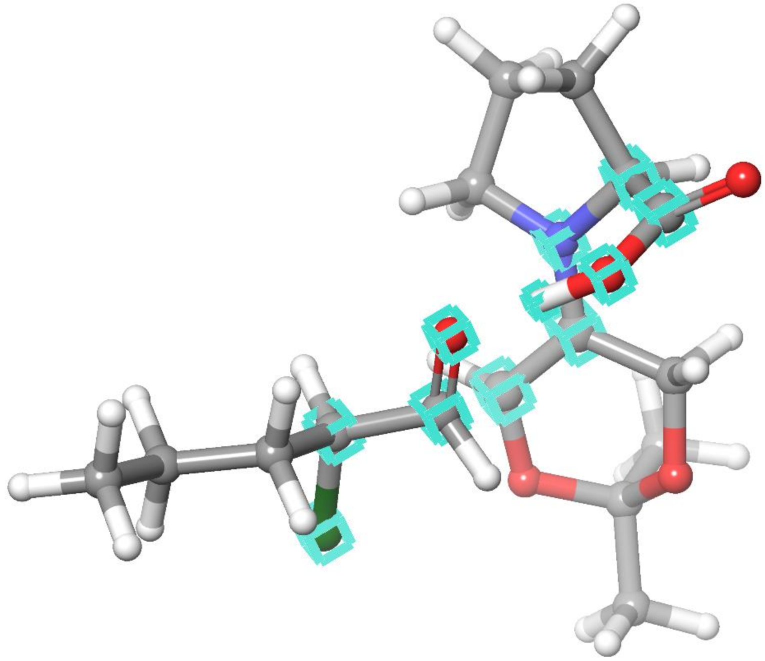

Supplementary Figure 102. Fixed internal coordinate of Evans-Cornforth-type transition state model for addition of enamine derived cyclohexanone to (*S*)-2-chloropentanal.

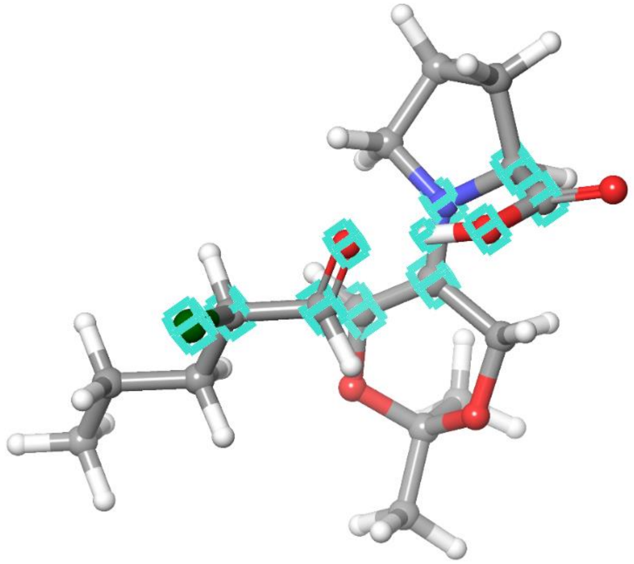

Supplementary Figure 103. Fixed internal coordinate of Felkin-Anh-type transition state model for addition of enamine derived cyclohexanone to (*R*)-2-chloropentanal.

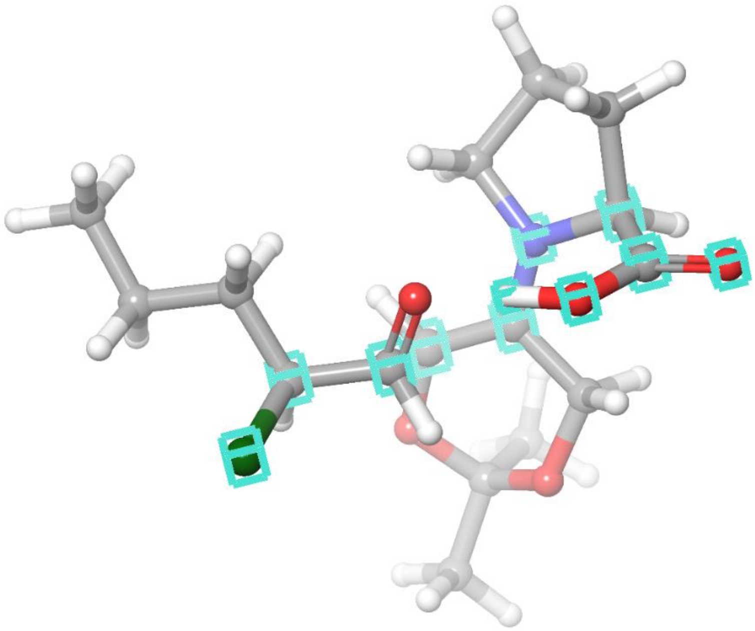

Supplementary Figure 104. Fixed internal coordinate of Felkin-Anh-type transition state model for addition of enamine derived cyclohexanone to (*S*)-2-chloropentanal.

Supplementary Data for Natural Bond Orbital Analysis

Computed natural bond orbital (NBO) energies for fluoride atom lone pair (*n*) donation into a H-C(*sp*^{2}) anti-bonding orbital (*) of the enamine ($\eta_F \rightarrow \sigma^*_{(C\text{-}H)}$) of (*R*)-**TS1**$_O$-F.

Second Order Perturbation Theory Analysis of Fock Matrix in NBO Basis.

Donor NBO (i)	Acceptor NBO (j)	E(2) kcal/mol	E(j)-E(i) a.u.	F(i,j) a.u.
87. LP (1) F 49	/516. BD*(1) C 1 - H 2	0.22	1.68	0.017
88. LP (2) F 49	/516. BD*(1) C 1 - H 2	0.38	1.02	0.018
89. LP (3) F 49	/516. BD*(1) C 1 - H 2	0.47	1.02	0.020

Overall energetic contribution = 1.07 kcal/mol
